# The Evolution of the Secreted Regulatory Protein Progranulin

**DOI:** 10.1371/journal.pone.0133749

**Published:** 2015-08-06

**Authors:** Roger G. E. Palfree, Hugh P. J. Bennett, Andrew Bateman

**Affiliations:** Endocrine Research Laboratory, Experimental Therapeutics and Metabolism, Research Institute of the McGill University Health Centre and Department of Medicine, McGill University, Montreal, Quebec, Canada; University of Lausanne, SWITZERLAND

## Abstract

Progranulin is a secreted growth factor that is active in tumorigenesis, wound repair, and inflammation. Haploinsufficiency of the human progranulin gene, *GRN*, causes frontotemporal dementia. Progranulins are composed of chains of cysteine-rich granulin modules. Modules may be released from progranulin by proteolysis as 6kDa granulin polypeptides. Both intact progranulin and some of the granulin polypeptides are biologically active. The granulin module occurs in certain plant proteases and progranulins are present in early diverging metazoan clades such as the sponges, indicating their ancient evolutionary origin. There is only one *Grn* gene in mammalian genomes. More gene-rich *Grn* families occur in teleost fish with between 3 and 6 members per species including short-form *Grn*s that have no tetrapod counterparts. Our goals are to elucidate progranulin and granulin module evolution by investigating (i): the origins of metazoan progranulins (ii): the evolutionary relationships between the single *Grn* of tetrapods and the multiple *Grn* genes of fish (iii): the evolution of granulin module architectures of vertebrate progranulins (iv): the conservation of mammalian granulin polypeptide sequences and how the conserved granulin amino acid sequences map to the known three dimensional structures of granulin modules. We report that progranulin-like proteins are present in unicellular eukaryotes that are closely related to metazoa suggesting that progranulin is among the earliest extracellular regulatory proteins still employed by multicellular animals. From the genomes of the elephant shark and coelacanth we identified contemporary representatives of a precursor for short-from *Grn* genes of ray-finned fish that is lost in tetrapods. In vertebrate *Grn*s pathways of exon duplication resulted in a conserved module architecture at the amino-terminus that is frequently accompanied by an unusual pattern of tandem nearly identical module repeats near the carboxyl-terminus. Polypeptide sequence conservation of mammalian granulin modules identified potential structure-activity relationships that may be informative in designing progranulin based therapeutics.

## Introduction

**Fig 1:**
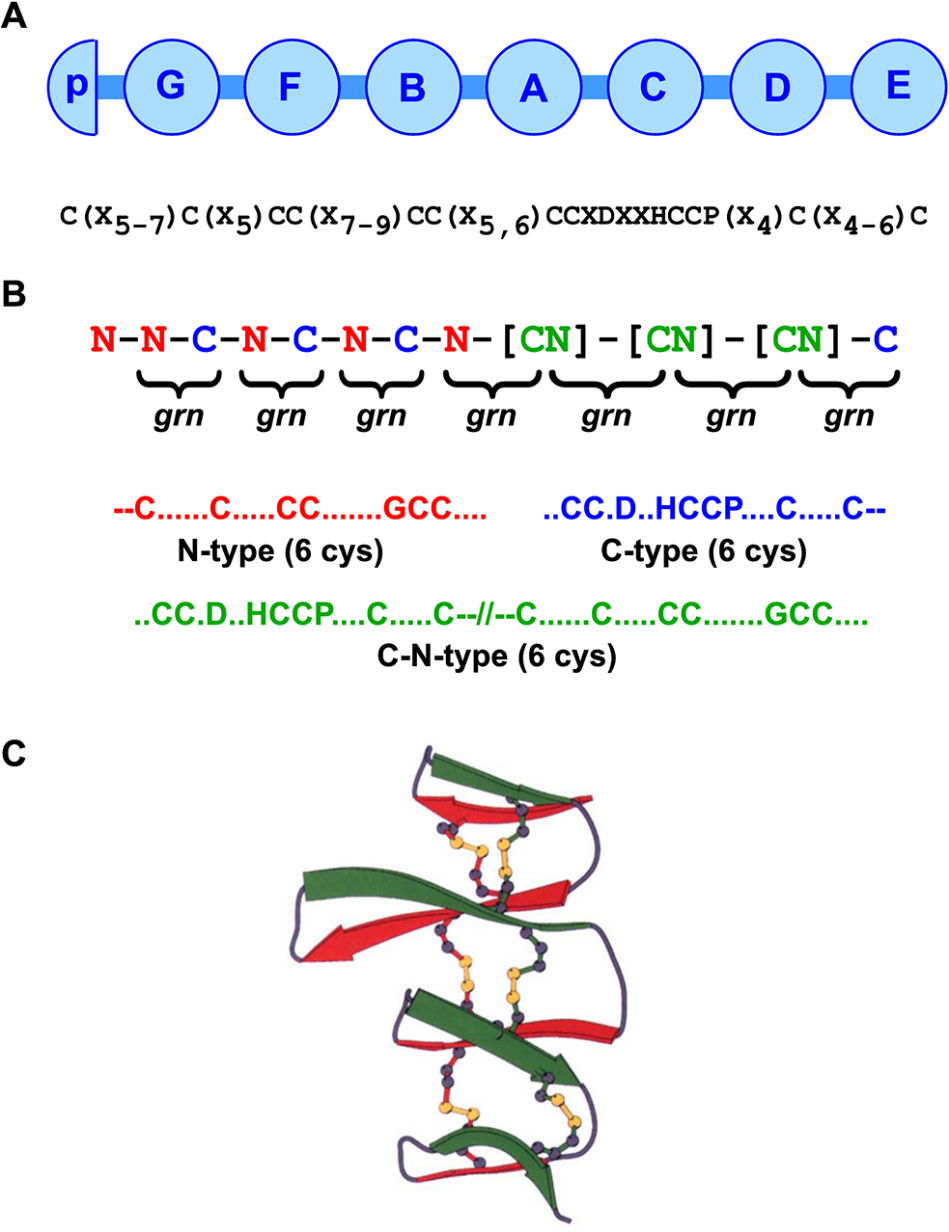
The protein and gene structure of a mammalian progranulin. (A). Progranulin is composed of multiple repeats of a highly conserved 12-cysteine granulin motif [1, 39]. Mammalian progranulins possess 7.5 granulin modules labeled A to G (circles) as well as a half-granulin motif called paragranulin (half circle, p) that contains the N-terminal six cyteines of the full granulin motif. The modules are separated by short joining sequences and can be released as individual granulin peptides of about 6kDa by proteolysis of PGRN (B) Each granulin module is encoded by two exons [40]. These are referred to as N-type exons (red), C-type exons (blue) that encode the first six N-terminal and last six C-terminal cysteines respectively or CN exons (green) that span the final C-terminally located six cysteines and the first N-terminally located six cysteines of adjacent granulin modules. The corresponding granulin polypeptide modules are bracketed. (C) The spatial conformation of carp granulin, determined by 2-dimension nuclear magnetic resonance spectroscopy [41] reveals that the granulin modules adopt a compact fold of beta turns (red and green ribbons) with the 6 disulfide bridges shown as yellow bonds.

Progranulin (which is also known as PC cell-derived growth factor, acrogranin, proepithelin, granulin-epithelin precursor or epithelial transforming growth factor) is a growth factor-like, immunomodulatory and neurotrophic extracellular regulatory protein. It is composed of tandem repeats of the granulin/epithelin module, a protein motif that is defined by a conserved pattern of 12 cysteine residues (Fig 1A) [1, 2]. In some instances progranulin is processed to liberate individual granulin modules as 6kDa peptides, called granulins (reviewed in [3]). Both the granulins and the intact progranulin have growth modulatory activity [2, 4]. Whether it be precursor or cleavage product, activity will be influenced by the structure of individual granulin domains. Mutation causing functional loss of one copy of *Grn*, the single gene encoding progranulin in humans, results in a form of a fatal neurodegenerative disease called frontotemporal dementia [5, 6]. Consistent with its role in dementia, progranulin is neuroactive, favouring neuronal survival and neurite extension [7–9]. Lentiviral delivery of progranulin to the brain limits the progression of Parkinsonian [10] and Alzheimer-like conditions [11] in mice. Progranulin is widely expressed in epithelial and inflammatory cells [12] and regulates cell proliferation, migration and survival [13–18]. Cancers often over-express progranulin [19] and several reports have demonstrated that progranulin promotes tumorigenesis [14, 20–24]. Loss of progranulin results in lysosome storage dysfunction, suggesting roles in regulating lysosomal function [25]. The physiological roles of progranulin include wound repair [18, 26], the regulation of inflammation [26–30], angiogenesis [18, 31], bone growth [32], sexual differentiation of the hypothalamus [33] and early embryogenesis [34].

The evolutionary history of the *Grn* gene family extends from premetazoan organisms, including slime molds and green plants, to mammalian genomes, making it among the earliest extracellular regulatory proteins still present in contemporary organisms [35]. The details of progranulin evolution have not, however, been investigated. Polypeptide motifs are often used in many different proteins and contexts, as is for example the epidermal growth factor motif, [36]. The granulin motif, in contrast, has a very restricted incidence and occurs in only a small number of genes per genome. Tetrapods, for example, possess only one *Grn* gene, whereas ray-finned fish (the Actinopterygii**)** possess up to six *Grn* genes. Typically these include genes for two proteins, GrnA and GrnB, that are long multi-modular progranulins similar to those found in land vertebrates (long-form *Grn* genes), as well as up to four smaller polypeptides with between 1.5 and 3 granulin modules (short-form *Grn* genes). The short-form granulin polypeptides of fish are themselves variable, with structures that may be summarized as, among others, gp, pg, gg, and ggg, where g is a 12-cysteine granulin module and p is a half granulin domain having the first 6 cysteines of a full module. It is unclear if they arose from one, or multiple ancestral genes. Indeed the presence of a family of short-form *Grn* genes in fish genomes that have no obvious counterpart in mammals has remained puzzling since their existence was first reported [37, 38]. Here we adopted an evolutionary approach to gain some insight into the origins and nature of the multiple *Grn* genes of fish.

Granulin modules are typically encoded by two non-identical exons (Fig 1B) [40, 42]. Three common variants of these exons occur that will be referred to here as N-exons, C-exons and CN-exons. N-type exons encode the N-terminal half of the granulin module including the first six cysteines of the 12-cysteine granulin motif. C-type exons encode C-terminal half of a granulin module, spanning the final six cysteines. Splicing of an N-type exon with a C-type exon generates the sequence encoding a complete granulin module. A CN exons sequence encodes a C-terminal half-granulin domain that is separated by an intervening, short, cysteine-free joining region from an N-terminal half-granulin domain (Fig 1B). Complete granulin modules (N/C) can be constructed from CN exons either by sequential splicing with another CN exon, or by appropriately placed N and C exons; that is, CN/CN; N/CN, or CN/C, where the resultant granulin module is underlined (Fig 1B). The spatial conformation has been determined for granulin modules from a teleost fish, the carp [41], and from human [4]. The granulin folds were found to consist of four β-hairpins stacked one above the other in a twisted ladder-like formation with six disulfide bridges that pin the four β-hairpins in place (Fig 1C). Determining which residues are most conserved in the granulin fold, and which are more variable, and how this is related to the spatial conformation of the granulin module, may identify the sequences that control folding or biological activity and assist the development of new therapies based on the structure of progranulin and its modules.

This study focuses upon unanswered questions in the evolution and structure of progranulins and their granulin domains, notably (a): How are granulin module-containing proteins in unicellular eukaryotes related to the progranulins of multicellular animals? (b): What evolutionary pathways led to the multiple *Grn* genes of ray-finned fish as opposed to the single *Gr*n gene of the tetrapod genomes? (c): How might the granulin module architectures of vertebrate *Grn* genes have arisen by conservation, duplication or deletion of exons? (d): Does the conservation of polypeptide sequences in mammalian granulin modules provide insight into the structure/activity relationships governing progranulin or granulin peptide activity?

## Methods

### Sequence data

The primary sources of sequence data were the databases of the National Center for Biotechnology Information (NCBI, at http://www.ncbi.nlm.nih.gov/). Other sources were the Joint Genome Institute (JGI, http://genome.jgi.doe.gov/), the Wellcome Trust Sanger Institute (https://www.sanger.ac.uk/resources/databases/), and Ensembl (http://www.ensembl.org/). To search for pre-metazoan *Grn* genes we used (i): the NCBI database using NCBI database Basic Local Alignment Search Tools (BLAST) [43] with blastp restricted to 42 unicellular eukaryote genomes and blastx restricted to 102 unicellular eukaryote genomes, (ii) the Taxonomically Broad EST database (http://tbestdb.bcm.umontreal.ca/searches/welcome.php) which stores EST data from 49 diverse unicellular eukaryotes, and (iii) the Origins of Multicellularity Database (http://www.broadinstitute.org/annotation/genome/multicellularity_project) which is a collection of data from nine unicellular organisms closely related to metazoa. NCBI protein blast analyses were performed using the NCBI default parameters (maximum sequences displayed 100, automatically adjust parameters for short input sequences, expect 10, word size 3, BLOSUM62 matrix, gap cost existence: 11, extension: 1). The presence of a granulin motif (Fig 1) was always confirmed by inspection and all putative granulin modules were positively identified as members of the granulin superfamily by the NCBI Conserved Domain Database. Blastp e value cutoffs were determined experimentally. Values smaller than 1 e^-12^ were considered strong. For example, GrnA (281–336 of human *GRN* NP_002078) returns an e value of 3e^-32^ against an expression construct for GrnA (PDP: 2JYE_A), 8e^-12^ against a Granulin F expression construct (PDB: 2JYV_A) and 1e^-12^ against the distant granulin of *C*. *elegans* (NP_492982.1). Blast results for individual granulin modules were considered possible between 1e^-12^ and 5e^-06^ provided the presence of the granulin motif was independently confirmed. This range was chosen empirically from blastp analyses that compared the evolutionarily very distant granulin modules from the plant *Aribidopsis thaliana* with human granulin modules.

The granulin superfamily conserved domain accession number is cl02546 (pfam00396, smart00277), through which annotated granulin-related sequences can be found for several species including plants, which have a distinct variation on the granulin cysteine motif. Ensembl has been a useful source of assembled data on identified granulin genes which are found easily through the "granulin" keyword search. The routine search strategy outlined below, however, provided data for granulin genes in most species ahead of their appearance in Ensembl, and allowed more reliable assembly of data for several granulin gene predictions.

Much of this work relied on mining NCBI databases via BLAST [43], usually initiated via tblastn, followed by blastn searches with retrieved and assembled nucleotide sequences. NCBI default blast settings were used. Selection of relevant tblastn hits was based on matching the characteristic cysteine motif. Assembly of short genomic (wgs) or cDNA (est) sequences was accomplished mostly with the aid of Cap3 [44], followed by inspection for possible sequence or assembly errors when a translated result showed an unusual character for a progranulin. In making predictions based on genomic data, Genescan (http://genes.mit.edu/GENSCAN.html) [45], and Augustus (http://bioinf.uni-greifswald.de/augustus/) [46, 47] were useful tools, but a good prediction required additional manual discovery of exons by searching for the characteristic cysteine motifs in the sequence translations, and testing splice predictions against any available cDNA data from the same, or a closely-related species.

For the subset of progranulin sequences presented in this work, current database accession numbers are given, and additional information is presented to explain where any prediction differs from ours.

The mammalian sequences analyzed are listed in Table 1. Amended sequences differ from NCBI curated sequences as follows: *American pika*: Our prediction is from genomic sequences represented by ALIT01101371.1, AAYZ01605896.1, AAYZ01106259.1; *Amur tiger*: XM_007093809.1 has an error in the N-half of module 6. Sequence Read Archive (SRA) data, NCBI, correct this (e.g. SRR836354.382753965.1 and SRR836354.358741544.2; *Dog*: XM_005624397.1 misses 2 granulin modules, but their presence is confirmed by EST sequence data; *Domestic Guinea Pig*: The missing 5' coding sequence and correct exon splicing was obtained from analysis of the AAKN02045624.1 genomic entry. *Giant Panda*: has an incorrect splice in module 6, but the correct sequence and splice donor is in SRA data SRR504859.58680058.1; *Goat*: XM_005693928 has minor sequence errors, one of which affects one Cys codon in module 2, but an assembly of 24 Transcriptome Shotgun Assembly (TSA) entries confirms a normal Cys motif. Sequences from the other species used in assessing progranulin evolution at the half-module level require more explanatory notes, and are provided as S1 Table.

**Table 1:** Mammalian progranulin sequences analyzed and their NCBI or Ensembl accession codes.

Common Name	Species	Sequence source*
Alpaca	Vicugna pacos	XM_006217455.1
Olive Baboon	Papio anubis	XM_003913166.1
Big Brown Bat	Eptesicus fuscus	XM_008149260.1
Wild Bactrian Camel	Camelus ferus	XM_006173088
Dog	Canis lupus familiaris	XM_005624397.1 Amended
Yangtze River dolphin	Lipotes vexillifer	XM_007455114.1
Bottlenosed Dolphin	Tursiops truncatus	XM_004311155.1
African Savanna Elephant	Loxodonta africana	XM_003414497.1
Black Flying Fox	Pteropus alecto	XM_006910127.1
Goat	Capra hircus	XM_005693928 Amended
Domestic Guinea Pig	Cavia porcellus	M86735.1 Amended
Small Madagascar Hedgehog	Echinops telfairi	XM_004707222
Horse	Equus caballus	XM_001489741.3
Human	Homo sapiens	XM_005257253.1
Sunda Flying Lemur	Galeopterus variegatus	XM_008590834.1
Crab-eating Macaque	Macaca fascicularis	AB169715.1
Florida Manatee	Trichechus manatus latirostris	XM_004374295.1
Star-nosed Mole	Condylura cristata	XM_004684086
House Mouse	Mus musculus	NM_008175.4
Aardvark	Orycteropus afer afer	XM_007955233.1
Gray Short-tailed Opossum	Monodelphis domestica	ENSMODT00000014710
Sumatran Orangutan	Pongo abelii	NM_001133217.1
Giant Panda	Ailuropoda melanoleuca	XM_002919741.1 Amended
Pig	Sus scrofa	AY642268.1
American Pika	Ochotona princeps	Our prediction
Rabbit	Oryctolagus cuniculus	XM_008271579.1
Norway Rat	Rattus norvegicus	M97750.1
Southern White Rhinoceros	Ceratotherium simum simum	XM_004432561.1
Weddell Seal	Leptonychotes weddellii	XM_006749411.1
Chinese Tree Shrew	Tupaia chinensis	XM_006167349.1
Thirteen-lined Ground Squirrel	Ictidomys tridecemlineatus	XM_005328118.1
Amur Tiger	Panthera tigris altaica	XM_007093809.1 Amended
Polar Bear	Ursus maritimus	XM_008693545.1
Pacific Walrus	Odobenus rosmarus divergens	XM_004407257.1
Common Minke Whale	Balaenoptera acutorostrata scammoni	XM_007180193
Killer Whale	Orcinus orca	XM_004285981.1
Sperm Whale	Physeter catodon	XM_007127930.1

* See text for information on manually predicted and amended sequences.

### Sequence Alignments

DNA sequence alignments were based upon encoded amino acid alignments. Strict alignment of the characteristic cysteine motif produced subsets based on mostly single residue gaps between the first and second, fourth and fifth, or eleventh and twelfth cysteines. The results of alignments with Clustal W2 [48, 49] were used as an aid to set the positions of these gaps, and then, in order to keep a compact alignment with the minimum of gaps, the gap position of the majority was adopted and a simple gap extension was applied for the few instances of shorter inter-cysteine sequence. In unusual cases a gap was needed elsewhere in the sequence, or a residue (codon) had to be removed. The DNA alignments used in the phylogenetic analysis are provided as S2 Table.

### Consensus Sequences

For generating consensus sequences, a stand-alone version of Mview (ver. 1.56) was used [50]. For the consensus-histogram representation of mammalian progranulin, major gaps in protein sequence alignments were closed by removal of PSAPLVRGP from J7 (joining sequence between modules 6 and 7) of the Star-nosed Mole, GAEGTPVVSPGLLPAALPT from J3 and QPSPPGPPGPPSP from J5 of the Guinea pig, and shorter sequences of between 4 and 7 amino acids in J3 of 17 other species. Three residues were removed from J1 of Elephant (SLS), Aardvark (TLT), Manatee & Hedgehog (SLP). A few other single residue gaps were closed in joining sequences. There were no gaps to close in the granulin modules except for a single residue removed from within the progranulin of Pika. Mview was called to provide 6 consensus sequences with identities from 50% to 100% in intervals of 10%. The Mview output provides the following notation where the conservation threshold is not reached by a single amino acid but by a residue-type: o, alcohol; l, aliphatic; a, aromatic; h, hydrophobic;-, negative; +, positive; c, charged; p, polar; s, small; u, tiny; t, turnlike; a dot is any residue. For the figure, the html output was edited and some colour style adjustments were made prior to image capture.

### Mammalian module and half-module distance analysis

Sequence distance calculations utilized components of the Phylip phylogenetic analysis package [51]. The inter-species polypeptide variability of each mammalian granulin module was investigated by analysis of the set of 666 pairwise distances between the sequences from 37 species calculated via PROTDIST using the Jones-Taylor-Thornton (JTT) matrix model. Box plots were prepared from the data. Values for the distances between DNA sequences encoding corresponding pairs of mammalian progranulin N- or C-half modules were calculated with the LogDet distance model [52] in DNADIST. In this work, the beginning and end of a module sequence was taken to include two amino acid residues flanking the first and the last Cys in the granulin motif. The junction between the N-half and C-half of any module is naturally defined by exon boundaries. This includes four residues beyond the second double Cys in the N-half of the motif. Five residues were included in the case of paragranulin, because this gave the best sequence alignment when paragranulins were compared with other N-half modules. By removing the fourth codon from N-half coding sequences of modules 2 and 3, the sequences in all eight sets (p, n1 to n7) were 90 bases in length. The sequences in all seven sets (c1 to c7) of C-half-encoding sequences were 75 bases in length after removal of codon 18 from all except c5. From the pairwise distances between each of the 37 sequences in each set, the mean and standard deviation were calculated.

### Maximum Likelihood Trees

For phylogenetic analysis we have employed programs within the Phylip package, and performed most of the final computations for the N-half and C-half trees in this paper using RaxML (version 8.0.22 running under Linux Ubuntu 12.04 LTS), which was faster and permitted more experimental approaches to searching and testing trees with the best likelihood scores [53]. Twenty four species were included, each providing data for from 1 to 5 genes encoding proteins composed primarily of one or more granulin modules. The analysis involved DNA sequence alignments of 307 distinct N-half modules and 300 distinct C-half modules. The sequences from the acorn worm and elephant shark were late additions. All others were included in the search for best maximum likelihood trees. The search with each N- or C-half module set proceeded via several approaches. First, following the method outlined in the RaxML manual, using the GTRCAT method, different rearrangement settings and rate categories were tested on several randomly generated parsimony trees for the full set of sequences. For either N-half or C-half input alignments the best rearrangement value was 20, while the best number for categories was 25 for the N-half set, although only slightly better than when numbers between 10 and 55 were chosen, and it was 40 or 55 for the C-half set. The best settings were then used to find the best trees from 50 inferences. Second, we took as an incomplete starting tree a previous result from running Phylip's DNAML on sequences from 17 of these species using the slower analysis with 20 jumbles and global rearrangement, and called on RaxML to perform 20 inferences for the complete set with the GTRCAT method. Third, to test order of species addition, we took four subsets of between 75 and 120 input sequences, one biased toward land/air vertebrates, one toward teleost fish, one for sea squirt, anemone, sponge and urchin, and one for the remaining species. Trees were found for each separately and used as incomplete starting trees for addition of other subsets. This was repeated until all species had been added.

These approaches gave 24 trees for each half-module set. Extended-majority-rule consensus N-half and C-half trees were generated from these tree sets, and provided as group models in the final search for best trees. The resulting likelihood scores for the best resulting N-half and C-half trees were slightly better than the best consensus input sequences. By visual comparison with the 24 input trees, the final N-half and C-half trees appeared to present largely stable topologies. In order to provide some measure of support, RaxML was called to start new searches from 100 random starting trees. When majority-rule consensus trees were obtained using the MR_DROP option to identify rogue taxa [54] there were nine from the N-half set (A_car_p, G_morC_p1, G_acuA_08z, D_rerB_09z, C_mil1_09z, P_marL_01, S_pur_27z, N_vecA_10, A_queL_02), and nine from the C-half set (X_tro_01, X_tro_14z, L_chaA_08, L_chaC_07, C_mil2_06, A_queL_01, S_kow_11, S_kow_12z*, N_vecA_11z). The rogue taxa were pruned from the 100 reference trees and from the best tree in order to obtain bipartition support values for the best tree. The bipartition support values for the positions of the rogue taxa were obtained from the non-pruned trees.

Sequence and tree file editing was facilitated by the following editors which accommodated the use of regular expressions: NoteTab, jEdit, and Gedit (with the advanced find/replace plugin). NJplot was used for regular tree visualization and organization [55]. Much use was made of TreeGraph 2 [56] in preparing the tree Figures, and Inkscape (http://www.inkscape.org/) was used in the final preparation of these and several figures in this report.

#### 3-D structure models

Currently the best 3-D model for a granulin module is the human granulin A structure 2JYE.pdb obtained from NMR studies [4]. To illustrate the spatial positions of conserved and variable residue sites in mammalian granulin modules, the pdb file was converted to a c3d file via the NCBI VAST search page (http://www.ncbi.nlm.nih.gov/Structure/VAST/vastsearch.html) to allow the powerful editing options in Cn3D [57]. Three-dimensional structure models of other granulin modules were developed using the on-line Swiss Model workspace via http://swissmodel.expasy.org/interactive [58]. 2JYE.pdb was the primary template. Model quality was assessed via ProQ2 [59] at http://bioinfo.ifm.liu.se/ProQ2/. In modeling the other 6 modules of human progranulin, a direct step from the 2JYE template often dropped a short sequence at the N terminal end. In general we found better results by stepping through several transition sequences presenting gradual changes between grn A and the target module sequence. At first transition sequences tested were hypothetical ancestral sequences generated from Phylip proml maximum likelihood analysis, but we found transition sequences prepared manually could produce as good or better results. This involved placing a residue with more intermediate properties at a site of most difference in the problematic section of sequence. The pdb files from the best Swiss Model predictions were converted to c3d format for preparing illustrations.

### Synteny and genomic context

Genome wide synteny was analyzed using the program Synteny Database (http://syntenydb.uoregon.edu/synteny_db/) set to a sliding window size of 200 genes [60]. Local synteny or genomic context, refers to the genes immediately flanking the target gene (in this case *Grn*) and provides a genetic signature for a gene and its immediate genomic environment that can be followed through evolutionary lineages. This was determined manually using the NCBI resource “Gene” (http://www.ncbi.nlm.nih.gov/gene) by walking along the chromosome in either direction from the target. Genes are identified by their assigned names in NCBI or, when recorded as an unknown, were subject to analysis by NCBI standard protein BLAST (blastp). Manual gene assignments were made using the top blastp hit with a named gene assignment, provided the gene name assignment was repeated in three or more species. If 5 or more of the top blastp hits failed to recognize an assigned gene we recorded the gene as “unknown.” The results are presented either in table form, or as screen shots from the NCBI Gene resource where transcripts are matched with chromosomal location.

## Results and Discussion

### The granulin module emerged before the transition from unicellular organisms to multicellular animals

**Fig 2:**
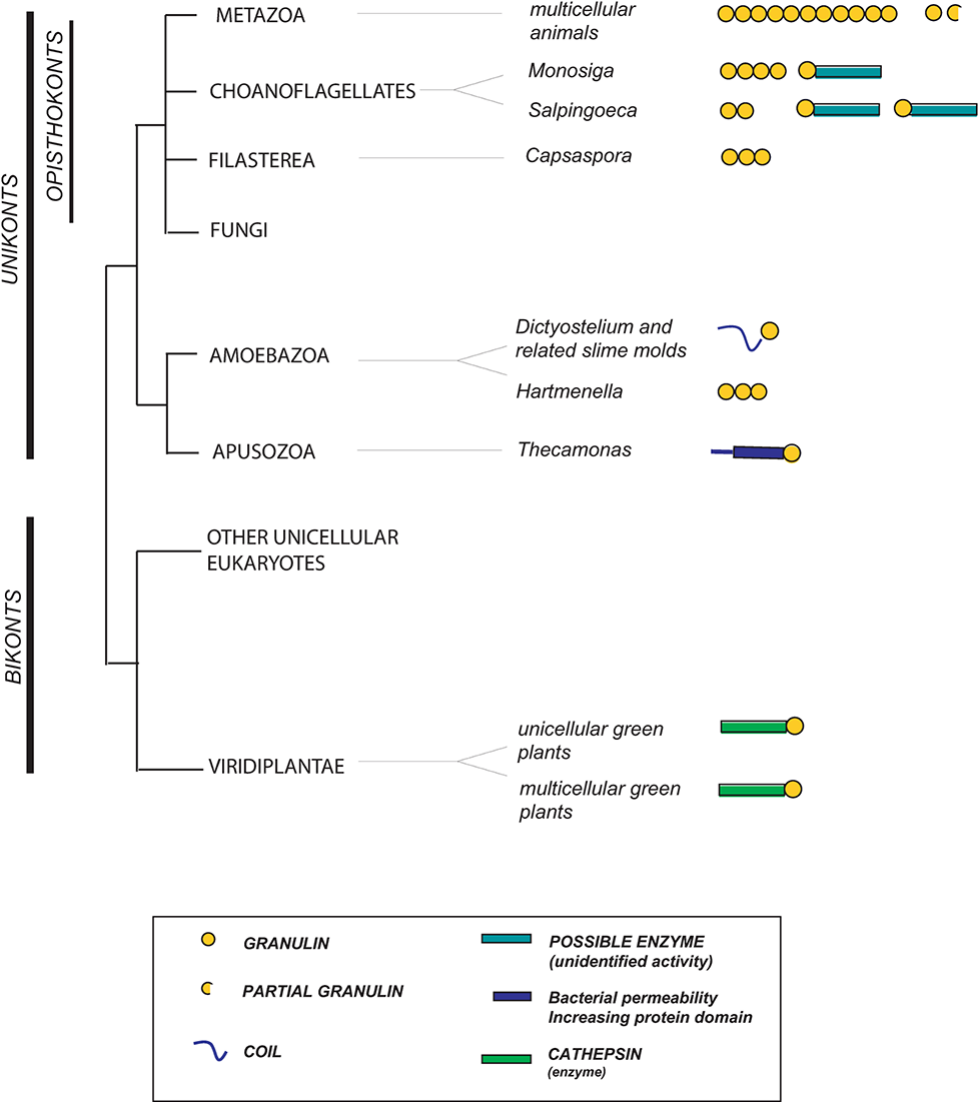
The nature and distribution of genes containing granulin modules in unicellular organisms, plants, fungi, and metazoa. Granulin modules are represented as yellow circles. Metazoans possess only progranulins, linear chains of granulin modules that may contain multiple modules or as few as 1.5. Similar progranulin like proteins are found in organisms closely related to metazoans including Choanoflagellates and Filasterea. Granulin-modules are encountered in other unikonts including slime molds and an Apusozoa but appear to be absent from fungi. In *Dictyostelium* and related slime molds the *Grn* genes contain one granulin module and a short N-terminal polypeptide tail. Among bikonts, genes containing granulin modules are reliably detected only in green plants (viridiplantae). In plants and some unikonts (Choanoflagellates and Apusozoa), but not in metazoa, the granulin modules are associated with non-granulin proteins (identified in the figure key). The distances between taxa are arbitrary.

To investigate when the granulin module emerged, and the nature of early granulin-containing proteins, we searched for the pre-metazoan distribution and evolution of *Grn* genes using databases outlined in the Methods. The results are summarized in Fig 2 and S3 Table. Choanoflagellates and filasterea are considered the closest unicellular organisms to metazoan animals [61] [62]. Genomic data from the unicellular choanoflagellate *Monosiga brevicollis*, the colonial choanoflagellate *Salpingoeca rosetta* and the filasterea *Capsaspora owczarzaki* revealed two classes of granulin module-containing proteins. The first class is composed of tandem granulin modules with 4.5, 2 and 3 modules in *Monosiga*, *Salpingoeca* and *Capsaspora* respectively (Fig 2 and S3 Table). This domain architecture resembles the metazoan progranulins. The second class is found in the choanoflagellates but not *Capsaspora* and is composed of proteins with a single granulin module that is linked to a protein sequence of unknown function (Fig 2 and S3 Table). These proteins may possess enzymatic activity since blastp searches reveal similarities with a putative Extradiol-Dioxygenase3-B-like molecule from the phytoplankton *Emiliana huxleyi* (EOD34296) and with the lipase-GDSL-2/SGNH hydrolase superfamily [63].

We investigated whether similar granulin-containing enzymes persisted into the metazoa. Blastp search of NCBI databases identified a protein (XP_001627333, blastp e value 2e^-27^) in the anemone *Nematostella vectensis* with homology to the granulin-module containing gene XP_004989953.1 of *Salpingoceca*. It possesses a SGNH/GDSL_hydrolase domain and a C-terminal region of 12 cysteines that was recognized by blastp as a granulin domain. The alignment of these cysteines is, however, very atypical of a granulin module (top e value for granulin-like was 5e^-05^) and may represent either a degenerate granulin or convergence on a granulin-like sequence. Further blastp searches using the *Nematostella vectensis* gene XP_001627333 as the search sequence revealed additional homologous genes in *Nematostella vectensis* (XP_001639667, blastp e value 4e-^40^), and *Hydra magnipapillata* (XP_002162489.2, blastp e value 6e^-37^) with the SGNH/GDSL hydrolase motif but no cysteine-rich region. No such genes were identified in other early diverging clades including sponges or *Trichoplax adhaerens*. It is possible, therefore, that remnants of the premetazoan granulin-enzyme proteins survive in the cnidarians but were extinguished elsewhere.

Metazoa, choanoflagellata and filasterea are often grouped together as opisthokonts, as are fungi, however we have not detected *Grn*-related genes in any fungal species. The NCBI database identifies a protein (XP_958881.1) in *Neurospora crassa* as containing a granulin module, but the alignment of this sequence with the canonical granulin motif is unconvincing (the highest Blastp e value against any granulin sequence is 0.007). Opisthokonts belong to a larger group, the unikonts, that includes taxa that are more distantly related to the metazoa such as *Amoebozoa* and *Apusozoa* [64]. Seven species of amoebozoan slime molds were found to encode granulin containing proteins (Fig 2 and S3 Table). These consist of a single C-terminal granulin-module, a short non-enzymatic N-terminal polypeptide sequence and a secretory signal peptide (Fig 2). The multiple granulin domains of choanoflagellate progranulins are encoded in a single large exon, whereas the granulin module of the slime mold genes is encoded by two exons, with the exon-intron boundaries located in the same position as the N and C-type exons of animals. Thus not only is the protein structure of the granulin module ancient, but so too, in slime molds, is the split exonic structure that encodes the module.

Other than slime molds genes with granulin modules were not identified in other *Amoebozoa* with the exception of *Hartmannella vermiformis*, a host for *Legionella pneumophila*, the bacteria causing Legionnaires' disease, where an EST that encodes for three sequential granulin modules was identified (S3 Table). The apusozoan, *Thecamonas trahens*, possesses a gene with a C-terminal granulin module linked to a protein that has homology with bacterial permeability-increasing proteins (Fig 2 and S3 Table). Fungi are thought to be evolutionarily closer to animals than are amoebozoa and apusozoa [65]. The presence of *Grn*-related genes in unikont genomes other than fungal suggests that the *Grn*-related genes are ancestral in unikonts but have been lost in the fungi (Fig 2).

Plant granulin-containing proteins differ from animal progranulins. The plant motif contains an additional two cysteines, and in all cases a single granulin module is linked to the C-terminus of a cathepsin-like cysteine protease. The secondary conformations of plant granulins are superimposable upon animal granulins [66] confirming their granulin-like identity. Granulin-containing cysteine proteases are represented in the genomes of unicellular green algae (Fig 2 and S3 Table) but not in the red algae. Plants are members of the bikonta, a very diverse group of organisms, but other than in the green plants we have found no evidence for any bikont granulin-containing molecules (for details see S1 Text). All unicellular granulin-module genes, have a signal peptide indicating that the encoded protein enters the endoplasmic reticulum either for secretion or packaging into vesicular organelles.

### Progranulin primary structures: Overview and plasticity in progranulin domain architecture

Automated annotation of genomes gives high error rates, with up to 50% of genes being incorrectly annotated in some draft genomes [67]. We therefore undertook independent manual interpretation of genomic data to evaluate the reliability of archived *Grn* sequences. *Grn* nucleotide sequences used in this report that were obtained by independent interpretation of genomic data, some of which differ significantly from sequences in public databases, are given in S1 Table. *Grn* genes are present throughout metazoan phylogeny including early diverging animal clades such as sponges *Amphimedon queenslandica* (XM_003382647.1), the basal eumetazoan *Trichoplax adhaerens* (XP_002110228.1), cnidaria such as *Hydra magnipapillata* (XP_002159371, XP_002165324) and the sea anemone *Nematostella vectensis* (XP_001619924.1). The only exception are certain insects such as the fruit fly and the honey bee although other insects possess *Grn* genes including *Locusta migratoria* (migratory locust, P80059)[68], *Zootermopsis nevadensis* (termite, KDR15531), *Manduca sexta* (tobacco hornworm moth, ABY61951.2) and *Aedes albopictus* (Asian tiger mosquito, ABY61952.1)[69]. Why *Grn* genes are lost from some insect genomes but not others is unknown. *Grn* genes may possess as few as one module (*Caenorhabditis elegans*, CCD72453) or greater than 30 (*Branchiostoma floridae*, Table 2 and S1 Table).

**Table 2:** Examples of granulin protein modular structure and gene exon structure.

Species	Form	Protein module structure^1^	Exon structure related to half modules^2^
Mammal		p-g*-g-g-g-g-g-g	sn-n*-c*-n-c-n-c-n-cn-cn-cn-c
*Anolis carolinensis* (green anole, iguana)		p-g*-g-g-g-g-g-g-g-g	sn-n*-c*-n-c-n-c-n-cn-cn-cn-cn-cn-c
*Xenopus tropicalis* (frog)		p-g*-g-g-g-g-g-g-g-g-g-g-g-g-p-g	sn-n*-c*-n-c-n-c-n-c-n-c-n-c-n-c-n-c-n-c-n-c-n-c-n-c-n-cn-n-c
*Danio rerio* (zebrafish)	GrnA	g-g*-g-g-g-g-g-g-g-g-g-g	sn-c-n*-c*-n-c-y-n-c-n-c-n-c-n-c-n-c-n-cn-cn-cn-c
	GrnB	g-g-g-g-g-g-g-g-g	sn-c-n-c-y-n-c-n-cn-cn-cn-cn-cn-c
	Grn1, 2	g-p	sn-c-n
*Petromyzon marinus* (sea lamprey)	L	g-g-g-g-g-g-g-g-g-g	sn-c-n-cn-cn-cn-cn-cn-cn-cn-cn-c
	S1	p-g"-x-p	sn-n"-c-xn
	S2	p-g"	sn-n"-c
	S3	p-g"-x	sn-n"-c-x
	S4	p-g”-x-ğ	sn-n”-c-xň-c
*Ciona intestinalis* (Sea Squirt)		g-g-g-g-g-g-g	s-n-c-n-c-n-c-n-c-n-c-n-c-n-c
*Branchiostoma floridae* (Florida lancelet)		g-g-g-g-g-g-g-g-[up to 31 repeated g]-g-g-g	sn-cn-cn-c-n-cn-c-n-cn-c'-|nc|-n'cnc[nc rpt exons]-nc-n-c-n-c-n-c
*C*. *elegans* (nematode)	1	g-g-g	s-ncnc-n-c
	2	G	(s)-(sn)-(n)c-t
*Monosiga brevicollis* (choanoflagellate)		g-g-g-g-p	s-ncncncncn
		g-x	sn-cx-x-x-x
*Dictystelium discoideum* (slime mold)	-	G	sn-c
Plant		x-ğ	sx-x-x-xňc-y

**1**. *Protein Module Structure*: Signal peptide not included; Hyphens indicate short intermodule sequence; g indicates a module with a typical 12 cysteine motif; g* indicates the alternative 10 cysteine motif often present in tetrapod progranulins; ğ represents the variant form of granulin module found in plants and the similar module in lamprey S4; g" represents a module missing the usual first double Cys; p indicates an N-half module (paragranulin form); q indicates a C-half module. Protein sequence recognized as a non-granulin component of the protein other than a simple intermodule sequernce is denoted by x, and in plants, the x represents a cysteine protease module.

**2**. *Exon Structure*: Hyphens indicate introns. Encoding of the N-terminal half or C-terminal half of a granulin module is indicated by n or c respectively, or by n* or c* in the case of the common alternative 5 cysteine half module forms, n" for a 4 cysteine form in lamprey, and by ň in the case of the plant N-half module or similar half module in lamprey. For *Branchiostoma*, c' and n' indicate that a single Cys belonging to the neighbouring module is also encoded in this exon, and |nc| indicates that the first and last single Cys in the motif are not encoded in this exon. The notation [nc rpt exons] indicates that the exon continues into highly repetitive granulin sequence, and it is uncertain precisely how many exons encode how many granulin modules. The end of the repeat contributes a C-half. For the single module *Caenorhabditis elegans* gene, the exon structure is unusual in that splicing occurs within the N-half sequence. The parentheses around s and n indicate parts of the signal or n sequence, and t represents a short terminal coding sequence. Exons encoding short amino acid sequences are represented by y, while x indicate exons encoding parts of non-granulin module.

The single tetrapod *Grn* gene demonstrates structural variation across taxa with respect to the number of granulin modules it encodes. Thus in placental mammals it has 7.5 modules, 9 in the lizard *Anolis carolinensis* (XP_003222565.1), and 12 (NP_001080678.1) and 10 (NP_001085281.1) modules in the two *Grn* genes of the tetraploid frog *Xenopus leavis*. Chicken progranulin (BX930405) has only 4 granulin domains raising the possibility that its phylogenetic origins might be related to the small forms such as are found in ray-finned fish. However, as determined using the Synteny Database, 168 genes including BX930405 located on chromosome 27 of the chicken (*Gallus gallus*) share synteny with human chromosome 17 where human *Grn* is located. The structurally simplified chicken *Grn* gene is therefore orthologous with the single *Grn* found in other tetrapods and is an example of module loss.

### Tandem duplication of near identical modules is a recurrent feature of progranulin evolution

An unexpected and recurrent feature of several *Grn*s is the presence of tandem repeats of multiple copies of identical or nearly identical granulin modules. For example, of the 13 granulin modules in the *Grn* of the California sea hare *Aplysia californica*, (XP_005090713.1), 11 encode identical polypeptide sequences, while a twelfth module differs from these at only 3 positions. Similar iterations of near-identical modules are encountered in *Grns* from *Nematostella vectensis*, the oyster *Crassostrea gigas* (CGI_10005248), the sea urchin (*Strongylocentrotus purpuratus*, Table 2 and S1 Table), and Amphioxus (*Branchiostoma floridae*, Table 2 and S1 Table), Xenopus leavis, and in *GrnA* (*D_rerA*, NP_001001949.2) of the zebrafish *Danio rerio*, where modules 4 to 7 are highly similar and differ by only five or fewer amino acid positions. Tandem duplication of identical and near identical modules may occur both within a single large exon, as for example in *Grn*s of amphioxus and *Strongylocentrotus purpuratus* (Table 2 and S1 Table) or be encoded by multiple N, C and CN split exons as are the repeats in the *Grn* genes of the *Aplysia* (unplaced scaffold NW_004797290.1 1504353–1556930, NCBI), and oyster (*Crassostrea gigas* unplaced genomic scaffold 41540, 108954–117117, NCBI) and zebrafish *GrnA* (Table 2).

A number of variants of the typical 12 cysteine granulin motif were identified (Table 2). These are discussed in S2 Text and are important as they provide information about the structural constraints governing the folding of the granulin module. CN exons occur often but the corresponding NC exons are rare, although in principle both should be equally likely to occur by loss of the intron separating C and N or N and C exons respectively. Possible reasons for the preponderance of CN over NC exons are discussed in S3 Text.

### Synteny reveals relationships between diverse forms of vertebrate *Grn* genes

To discriminate the evolutionary pathways that resulted in ray finned fish possessing multiple *Grn* genes but tetrapod genomes only a single *Grn* gene we first defined the orthology relationships of *Grn* genes. The orthology of long-form *GrnA* genes was confirmed (S1 Fig) as was that of the long-form *GrnB* genes (S2 Fig). The short-form *Grn*s of ray finned fish are the most diverse in module architecture (S4 Table) and the relatedness among themselves or with the long-form *Grn* genes was unclear. The genomic environments of three structurally divergent short-form *Grn* families in *Danio rerio* (zebrafish, 2 genes, *D_rer1*, *D_rer2*), *Takifugu rubripes* (Fugu fish, one gene, *T_rubC*), and *Oreochromis niloticus* (Nile tilapia, four genes, *O_nil1*, *2*, *3*, *4*) were investigated. There is conservation of the genomic regions flanking the short-form *Grn* loci in all three species (S3 Fig) with a characteristic flanking gene environment of HEPACAM2, CDK6, FAM113, GRN, SPP6RA, BIO, C9Orf23-like, SNX13 (incomplete in zebrafish). Thus despite differences in their modular architecture the short-form *Grn* genes in fish are orthologous and must originate from a common ancestral gene. To distinguish this gene family from the long-form *GrnA* and *GrnB* genes of fish we will refer to it as the *GrnC* orthology group. When more than one *GrnC* group gene occurs in a given genome they invariably lie immediately adjacent to one another along the chromosome, demonstrating that multiple short-form fish *GrnC* genes arose by local tandem duplication (S3 Fig).

Given that *GrnC* genes form a single orthology group much of the variability of their modular architecture would arise from exon deletions (S4 Fig and S4 Table). For example, the exonic structure of salmon *Sal_1* can be derived as SN-x-x-N-C where x indicates sites of exon deletion from the larger SN-CN-C-N-C structure of *S_salC* (salmon), *T_rubC* (fugu fish) and *G_acuC* (stickleback, *Gasterosteus aculeatus*). Fugu fish are an exception. They possess a short-form *Grn* (XP_003965070.2, *T_rubD*) with 2 granulin modules following an apparent remnant of a third module. The local genomic environment of this gene clearly corresponds to *GrnA*-group long-form *Grn* (S1 Fig). Thus, *GrnA* of fugu fish has lost modules to yield a novel short-form *Grn* but one that is not a *GrnC* ortholog.

**Fig 3:**
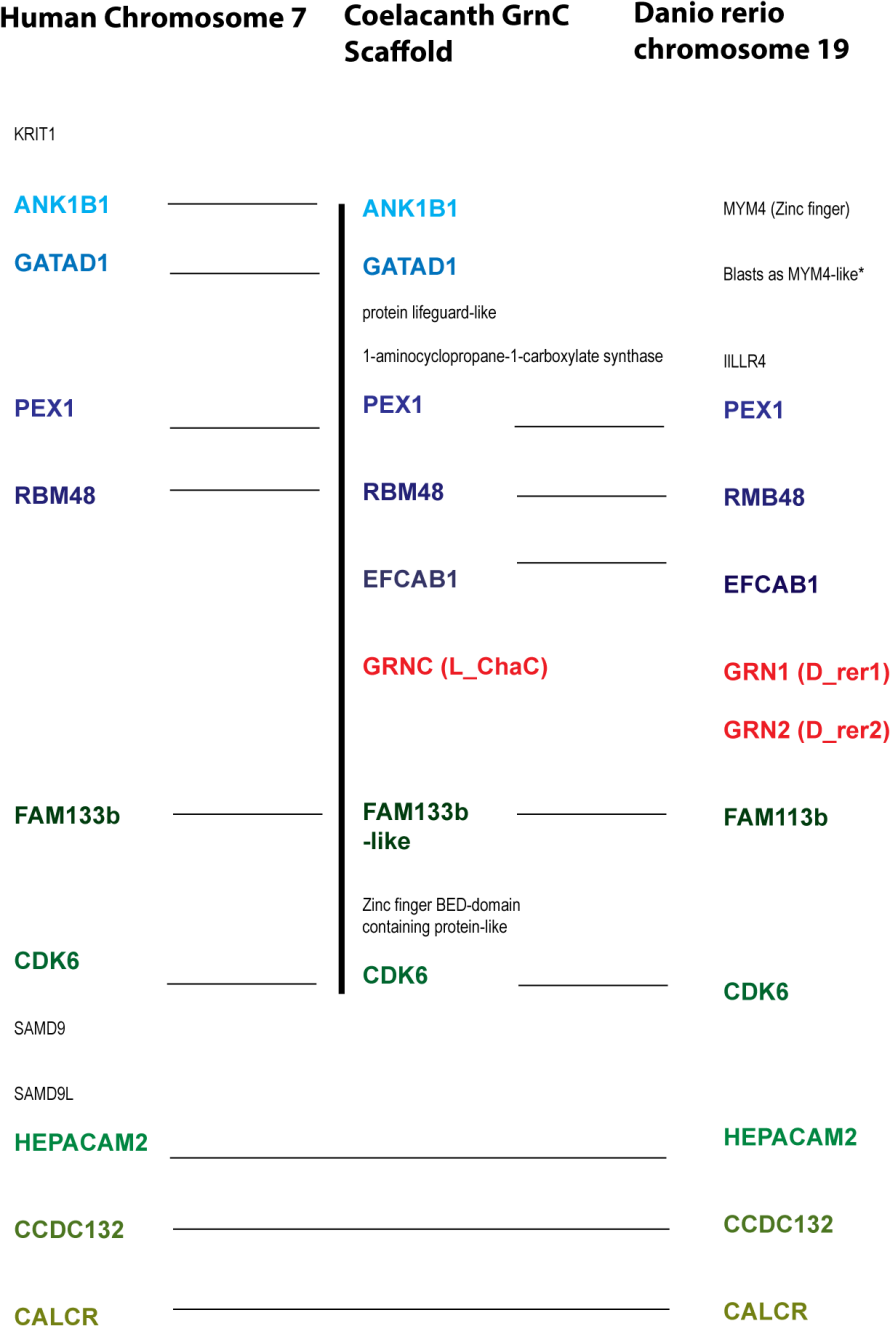
Syntenic conservation between the genomic regions around *GrnC* gene from the coelacanth, Zebrafish short-form *Grn* genes and a human Chr7 region. The genomic context of the Coelacanth *GrnC* (*L_ChaC*) based on an Ensembl scaffold (vertical line) aligns with the short-form *Grn* genes of the zebrafish, and a region of human chromosome 7. Genes in color are orthologues. Gene names are as assigned in the NCBI databases (Grn1 and Grn2 are *D_rer1* and *D_rer2* in Figs 5 and 6). *This gene was unassigned by NCBI and was identified by pBlast analysis (Ensembl:ENSDARG00000035821, NCBI accession for translated protein XP_005159758.1). An additional predicted protein coding sequence for piggyBac transposable element-derived protein 3-like (XP_005159764.1, not shown) is contained within the DNA sequence for XP_005159758.1.

**Fig 4:**
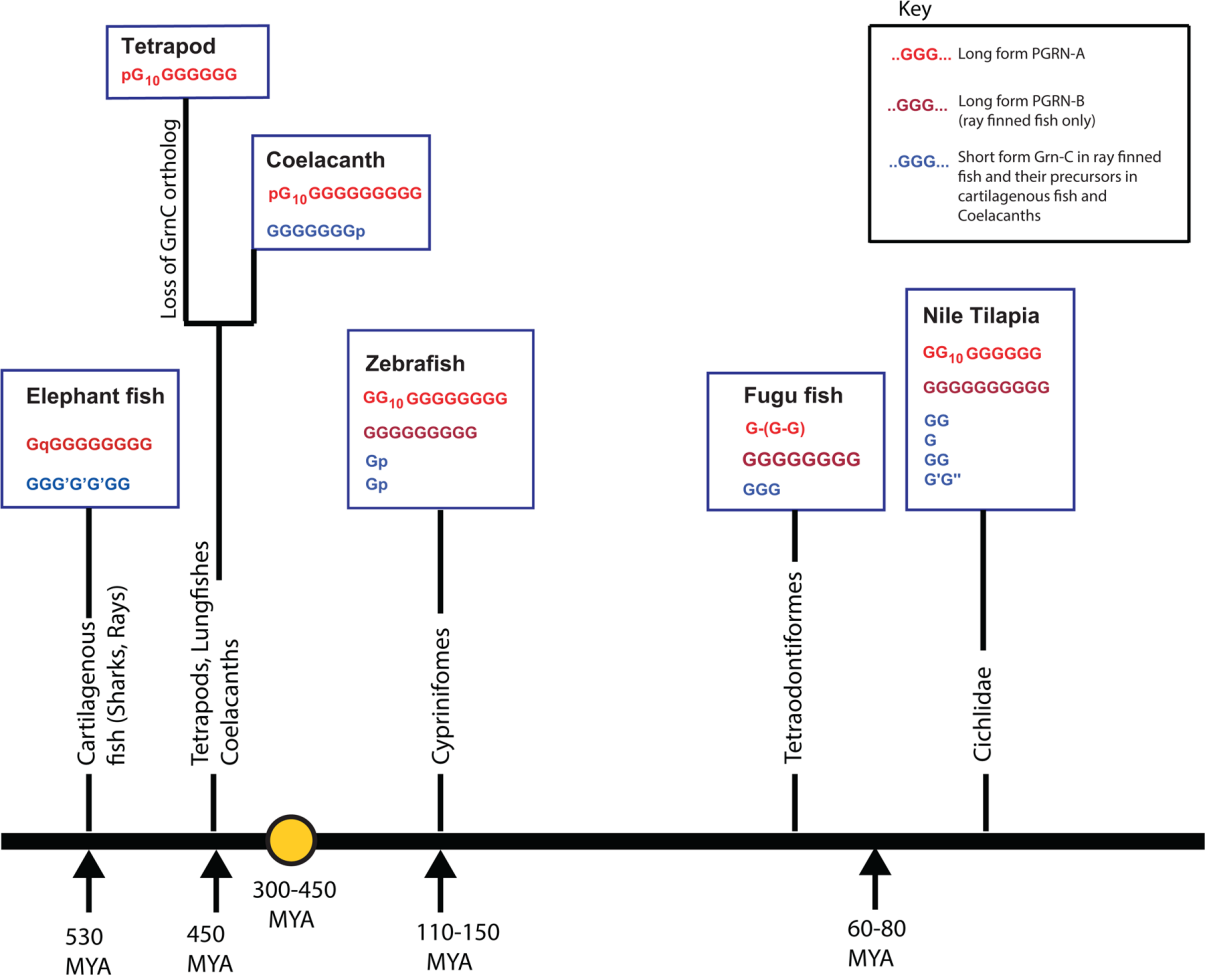
The structural variability of vertebrate GRN genes is built upon three conserved GRN orthology groups. Vertebrates have three *Grn* gene orthology groups which are represented here for the elephant shark *Callorhinchus milii*, the coelacanth *Latimeria chalumnae*, land vertebrates (tetrapods), and three representative modern ray-finned fish [*Danio rerio*, (zebrafish), *Takifugu rubripes* (fugu fish), and *Oreochromis niloticus* (Nile tilapia)]. *GrnA* orthologues (red) are characterized by a variant Cys10 granulin motif in the second granulin module. The coelacanth and tetrapod orthologues have lost part of the first granulin domain which is now represented by a six-cysteine paragranulin sequence (p). The gold circle represents a tetraploidization event in fish genomes between 450 and 300 Mya (gold circle,[71]). The *GrnB* orthology group (brown), found only in ray-finned fish probably arose during this genome duplication. The third orthology group, *GrnC* (blue) is absent in tetrapods, occurs in elephant sharks and coelacanths as long-form *Grn* genes, and in ray-finned fish as short-form *Grn* genes. In fugu fish the ‘long-form’ PGRN-A gene (red) has been reduced to a short-form with three modules (two modules are shown in parenthesis as they were identified from EST sequences). The positions along the horizontal axis represent the order of evolutionary precedence from early (elephant shark) to late (tilapia) with the time line based on reference [71]. Variations from the canonical 12-cysteine module are indicated as G’.

To identify the origins of the common precursor for the fish short-form *GrnC* genes we examined the genomic environments of the *Grn* genes of a cartilaginous fish, the elephant shark *Callorhinchus milii C_mil1* and *C_mil2* [70], and of a coelacanth, *L_ChaA* and *L_ChaC*. These are long-form *Grn* genes with between 10 and 7 modules (S1 Table). *C_mil2* and its flanking genes align with the region of the zebrafish genome that contains the short-form *GrnC* genes (see S5 Fig). The 11 protein-coding genes in the scaffold that includes *L_ChaC* show contiguous alignment with chromosome 19 of *Danio rerio* in the region surrounding the zebrafish small *Grn* genes (*Grn1* and *Grn 2*) and with human chromosome 7 (7Q21.2), even though this region of the human genome lacks a *Grn* gene (Fig 3). *C_mil2* and *L_ChaC* are therefore long-form orthologues of the short-form *GrnC* genes of ray-finned fish. The *GrnC* orthologue is lost in the tetrapod lineage (summarized in Fig 4). Local syntenic conservation around *C_mil1* was ambiguous (not shown) but coelacanth *L_ChaA* gene shows extensive synteny with genes surrounding human progranulin. 21 of the 24 protein-coding genes in the scaffold carrying *L_ChaA* align with contiguous genes surrounding human *Grn* on chromosome 17 (S5 Table). Thus tetrapod *Grn* and *L_ChaA* of the coelacanth are orthologous (summarized in Fig 4).

The *GrnA*/*GrnB* paralogous pair may have arisen during the well-established tetraploidization of the ray-finned genome or other duplication of large scale chromosomal regions [72]. Alternatively the two paralogs may have arisen by discrete duplication of a common precursor gene followed by translocation of one of the two genes to another chromosome. To address this question *GrnA* and *GrnB* genes from fish were screened using the Synteny Database for orthologous pairwise clusters with the human genome used as the outgroup. Results obtained as numbers of orthologous genes between fish and human genomes were *Danio rerio GrnA* 312, *GrnB*, 22; *Oryzias latipes* (medaka) *GrnA* 273, *GrnB* 173; *Xiphophorus maculatus* (southern platyfish) *GrnA* 333, *GrnB* 279; *Tetradon nigroviridis* (globe fish) *GrnA* 207, *GrnB* 200. This supports the hypothesis that the *GrnA*/*GrnB* paralogous pair arose following a large-scale or genome wide gene duplication event.

The Synteny Database [60] further revealed a possible syntenic relationship incorporating 43 orthologous gene pairs between human *GRN* on chromosome 17 and the *Grn-*like genes of the tunicate *Ciona intestinalis*. For comparison, 1099 orthologous pairs were detected in synteny with human *GRN* and mouse chromosome 11; and, as above, 312 genes between zebrafish *GrnA* and human chromosome 17. Vertebrate *Grn* is always located close to *mapt*, the gene for microtubule-associated protein Tau, that like *Grn*, causes frontotemporal dementia when mutated [73]. *Mapt* is included in the human/*Ciona Grn* syntenic cluster, suggesting that this unusual chromosomal apposition of two genes that cause the same clinical syndrome predates the origins of vertebrates.

### Loss of small-form Grn genes in the transition to land vertebrates

To investigate the disappearance of short-form *GrnC* genes in the transition from fish to tetrapods we compared the order and position of genes surrounding the short-from *D_rer1* and *D_rer2* locus in zebrafish with the human genome using manual inspection and the Synteny Database (S6 Table). Of 46 genes immediately surrounding the zebrafish short-form *Grn* locus, 40 were readily detected in the human genome, with most falling in one of two blocks of synteny on chromosome 7 and chromosome 1. Thus the loss of the short-form *Grn* gene from the tetrapod genome occurred by specific deletion of a *Grn* locus rather than by multiple gene deletion of a block of DNA. It is possible that the tetrapod short-form *Grn* gene has mutated beyond recognition however no unassigned genes were detected that might represent a relic short-from *Grn* gene in the human genome at or near the position predicted by synteny for the short-form *Grn*, and we have not been able to identify genes that might be candidates for a highly permutated *Grn* at other positions in the tetrapod genomes.

### Evolution of progranulin module architecture in vertebrate progranulins

**Fig 5:**
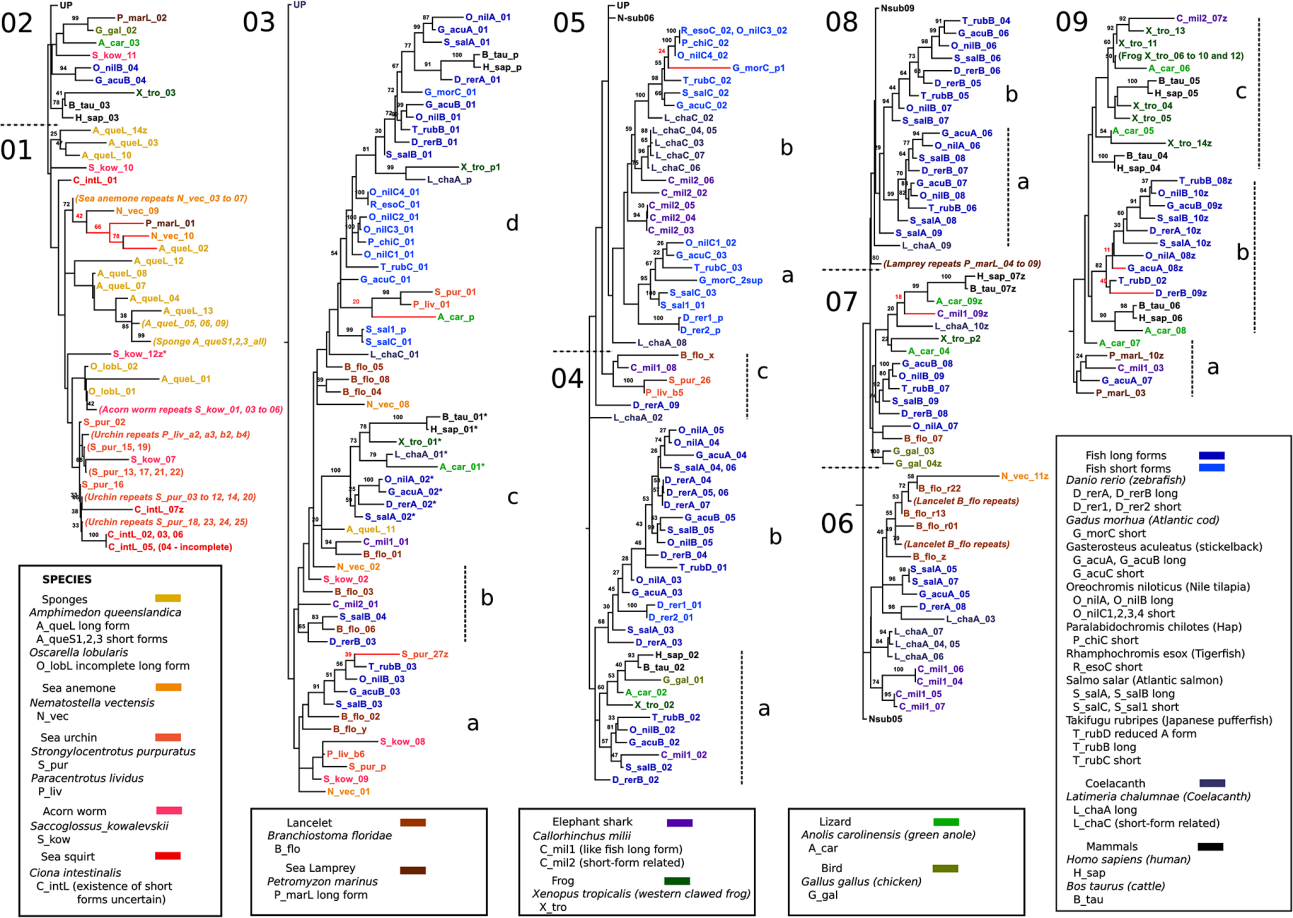
N-half module relationships illustrated by a DNA-based maximum likelihood tree. Letters and numbers are used to label some sub-trees and groups of sub-trees to facilitate reference from the text. Abbreviations for species and the specific Grn gene if there two or more are shown in the “species” boxes in the figure. In half-module labelling, this abbreviation is followed by a number based on the sequential numbering of whole modules encoded within the gene. For visual clarity, a "z" follows the number of the last module in a long-form of progranulin. When a repetitive module sequence or a lack of data make the number of modules uncertain, as in the lancelet (B_flo), the last letters of the alphabet replace numbering for the last sequential modules. N-half modules which are unpaired with C-half modules, paragranulins, are labeled "p", and followed by a number if there is more than one in the progranulin. An asterisk (*) indicates a 5-Cys form of half-module. (See also Fig 6 for a note on the cod module nomenclature). Bipartition support values of ≥20 are included. These were based upon data for trees from which rogue taxa had been pruned. The rogue taxa are indicated by red branches, and the support values for their placement in this topology are in red.

**Fig 6:**
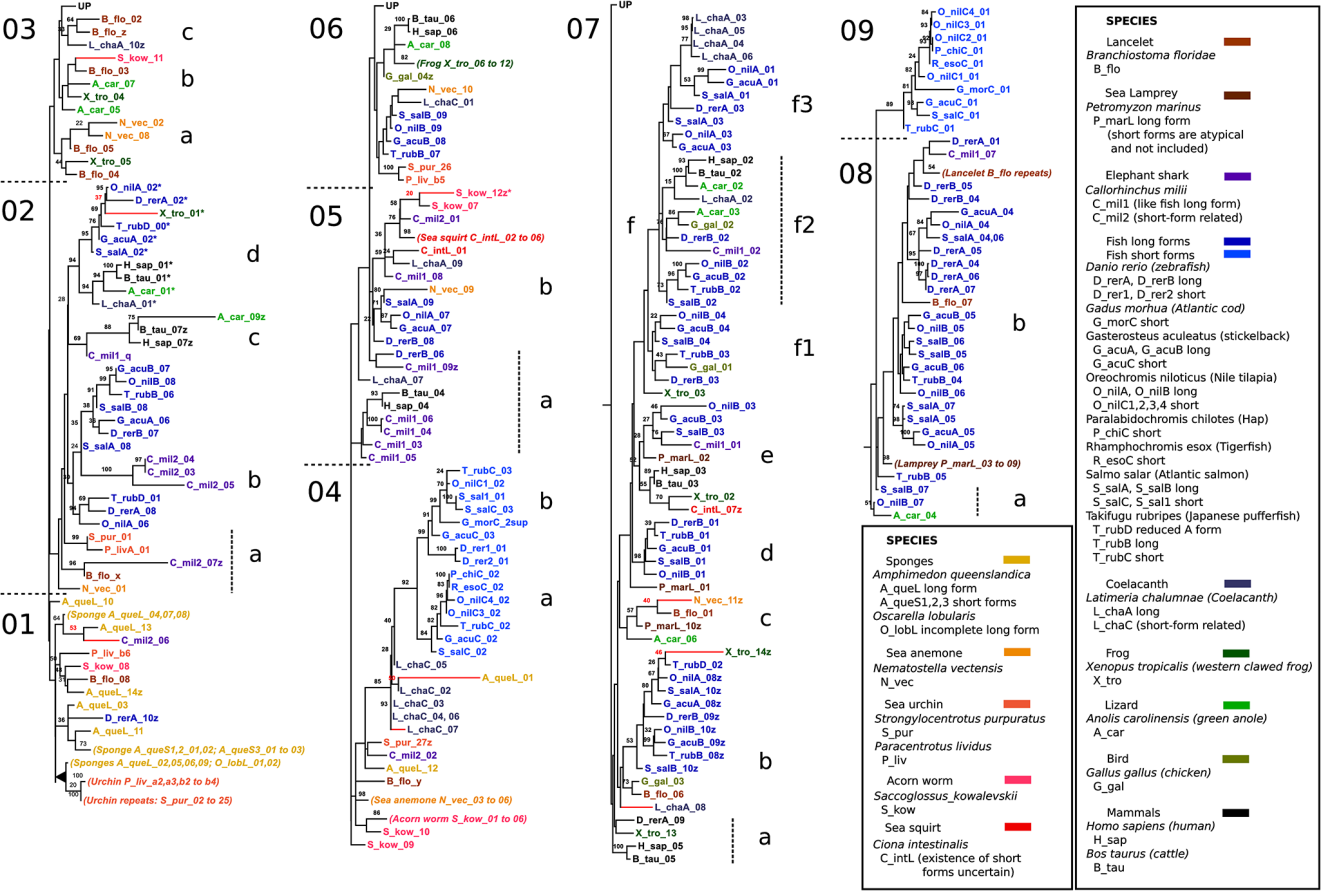
C-half module relationships illustrated by a DNA-based maximum likelihood tree. The Fig 5 legend provides most of the explanation. In addition, by analogy with labelling of paragranulins, a C-half with no paired N-half is labelled "q". The small form from Atlantic cod, G_morC, has the module form gpp, but if the second position stop codon in coding exon 4 is suppressed, the read completes an otherwise good C-half. This was included in the analysis and the virtual module called G_morC_2sup. Thus the third G_morC N-half module is properly a second paragranulin, G_morC_p2 in Fig 5, but is also the N-half of the G_morC_2sup virtual module.

The structure of vertebrate progranulins clearly resulted from successive module duplications. To identify pathways that may have led to their current modular architecture module relationships were investigated by DNA-based maximum likelihood tree analyses of evolutionary distances based on the N-terminal half of the various granulin modules (N-trees, Fig 5) or the corresponding C-terminal half modules (C-trees, Fig 6). Half module sequences were used in preference to whole module sequences because N- type, C-type and CN-type exons may be duplicated and rearranged independently. Moreover CN-type exons do not have a one to one correspondence with single granulin modules (Fig 1). Dendrograms were constructed using *Grn* sequences from the jawless fish *Petromyzon marinus* (the sea lamprey) which represents an early vertebrate; the cartilaginous elephant shark, the coelacanth, several long and short-form *Grns* from ray-finned fish, with tetrapods, represented by mammals *Homo sapiens* and *Bos Taurus*, the reptile *Anolis carolinensis*, the amphibian *Xenopus tropicalis*, and the bird *Gallus gallus*. In each figure the subtree containing the majority of sequences from the sponge *Amphimedon queenslandica* was taken as the out-group (sub-segment 01) from which the rest of the tree was ladderized up. Supporting bipartition values (≥20) are included at branch points. These were obtained by comparison of our best likelihood tree with 100 trees resulting from random starting trees.

The N-half sequences from vertebrate long-form progranulins (the GrnA and GrnB orthology groups) show a remarkable segregation of sequences with N-type exons located mostly in sub-segments Nsub02 to 04, while N-half modules from CN-type exons are segregated mostly in sub-segments Nsub06 to 09b (Fig 5). For C-type half modules (Fig 6) the exon types are more scattered, but predominantly C-type exon sequences are in Csub02c and d, Csub04a and b, and Csub07b,d,e and f, while C-half sequences from CN-type exons predominate in Csub02b, Csub05,06,08 & 09. The segregation of sequences derived from the CN type exons from sequences arising from the N and C type exons is consistent with a unique origin for the CN exons rather than multiple losses of introns between several C and N exons pairs. The half modules of the *GrnC* orthology group, (the short-form *Grn*s of fish), segregate apart from those of the long-form *GrnA* and *GrnB* orthology groups, and are located in subsections Nsub04d and Nsub05 (Fig 5) and Csub04a and b, and Csub09 (Fig 6). These branches include half modules of the elephant shark *C_mil2* and coelacanth *L_chaC* genes confirming their orthology with the small form *Grn*s of ray finned fish. The separation of half modules of the GrnC orthology group from the GrnA/GrnB type modules implies that the small form granulins of ray finned fish cannot be rationalized simply as reduced versions of the long-form progranulins.

Vertebrates belong to the deuterostoma. The relationship between module architecture of vertebrate and non-vertebrate deuterostome *Grn* genes was tested by including a tunicate, *Ciona intestinalis*, a cephalochordate *Branchiostoma floridae* (often called amphioxus), the hemichordate *Saccoglossus kowalevski* (an acorn worm) and two echinoderms, the sea urchins *Strongylocentrotus purpuratus* and *Paracentrotus lividus* (S1 Table). The half-module sequences of non-vertebrate deuterostomes were not consistently associated with any of the three vertebrate orthology groups. The tunicate sequences (*C_IntL*) and the highly repetitive sequences from the sea urchin *Grn*s (*S_pur* and *P_liv*) tend to align together in Nsub01 (Fig 5), the subtree chosen as the out-group. The *Saccoglossus* sequences (*S_kow*) align close to sea urchin sequences. In the C-tree most of the sea urchin sequences remained together and on a separate dendrogram subsection from other deuterostome sequences (Csub01, Fig 6) but the *Ciona* C half-module sequences merge with vertebrate sub-branches (C_intL_02 to 06 and C_intL_01 in Csub05, and C_intL_07 on Csub07). The *Branchiostoma* sequences (*B_flo*) were closer to vertebrate sequences than to those of other non-vertebrates both in the N- and C-trees. The segregation of non-vertebrate deuterostome granulin N-half module sequences with sequences from the out-group gene from the sponge *A*. *queenslandica* (long form *A_queL*, and short-form *A_queS1*, *2*, *3*, short-forms) was unexpected. We therefore included other *Grn* genes from early diverging clades, including those from another sponge, *Oscarella lobularis* and from the cnidarian *Nematostella vectensis*. These also branch in close association with non-vertebrate deuterostomes. This may reflect conservation of granulin module sequences across distant clades or the independent convergence of module sequences.

### Mapping N-tree and C-tree dendrogram coordinates onto the module architecture of long-form progranulins reveals patterns of evolution in vertebrate long-form progranulins

**Fig 7:**
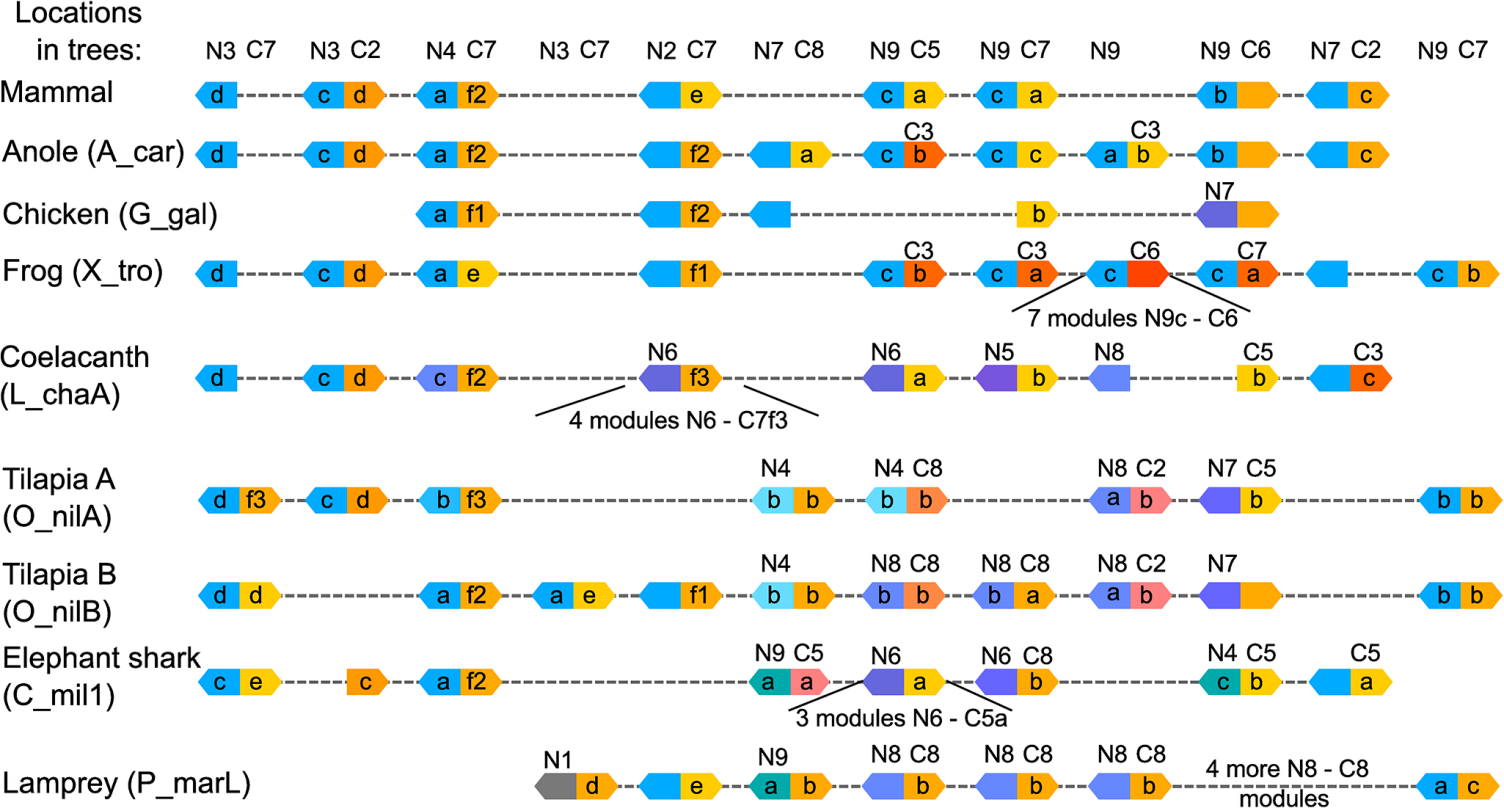
Schematic alignments of long-form progranulins from representative vertebrates summarizing positional half-module similarities and differences. The upper line, location in trees, provides the location of half-modules in the trees of Figs 5 and 6 and refers to the Nsub- or Csub- sections where they are placed. Thus, N3 is short for Nsub03, etc. Where the tree location differs from the column heading, it is written immediately above the half-module. The letters on half-module shapes refer to the labelled tree location within the sub-section in the trees of Figs 5 and 6. Colours are intended as a visual guide. Repeats of similar modules are shown as one module with the number of repeats written below (contraction of lamprey repeats was unnecessary).

To elucidate conservation and variation of vertebrate granulin modules we mapped the module architectures of vertebrate long-form progranulins (GrnA and GrnB orthologs) and the long-form *Grn* of the lamprey (*P_marL*) against the dendrogram coordinates of the corresponding N- and C-half sequences (Fig 7) which are shown in the upper lane of the figure. Consistent module conservation is evident in the amino terminal regions of long-form progranulins from elephant shark to tetrapods (Fig 7). Thus, the first amino-terminal modules locate to subsections N3 and C7 of the dendrograms with C7 lost in the progranulin of coelacanth and tetrapods (Fig 7). The second most N-terminal module locates to dendrogram subsections N3 and C2 for the elephant shark and all GrnA orthologs except for the reduced *Grn* of chickens (Fig 7). The third most N-terminal module locates to dendrogram subsections N4 and C7 and is conserved among all long-form progranulins from the elephant shark onwards. The dendrograms therefore predict an amino terminal modular architecture conserved from an ancestral vertebrate progranulin, based on sequences with the dendrogram coordinates N3 or N4 and C7or C2 (Fig 7).

Closer to the carboxyl-terminus the vertebrate progranulin modular architecture often features tandem repeats of closely similar modules. Thus in the lamprey (*P_marL*) a string of six adjacent highly similar modules locates to dendrogram subsections [N8,C8]; in elephant shark (*C_mil1*) and the coelacanth (*L_chaA*) strings of adjacent repeat modules occur at dendrogram coordinates [N6, C5a] and [N6, C7f3] respectively. Repetitive modular duplications occur in the *Xenopus Grn* (*X_tro*) with coordinates ([N9,C5], [N9,C7], 7x [N9,C6], [N9,C7]) and to a lesser extent in GrnA/GrnB paralogs of ray finned fish (Fig 7). Module evolution of vertebrate long-form progranulins can therefore be summarized as conservation of an ancestral architecture close to the amino terminus with frequent occurrence of tandem repeats of highly similar or identical modules closer to the carboxyl-terminus.

### Mammalian Granulin modules

**Fig 8:**
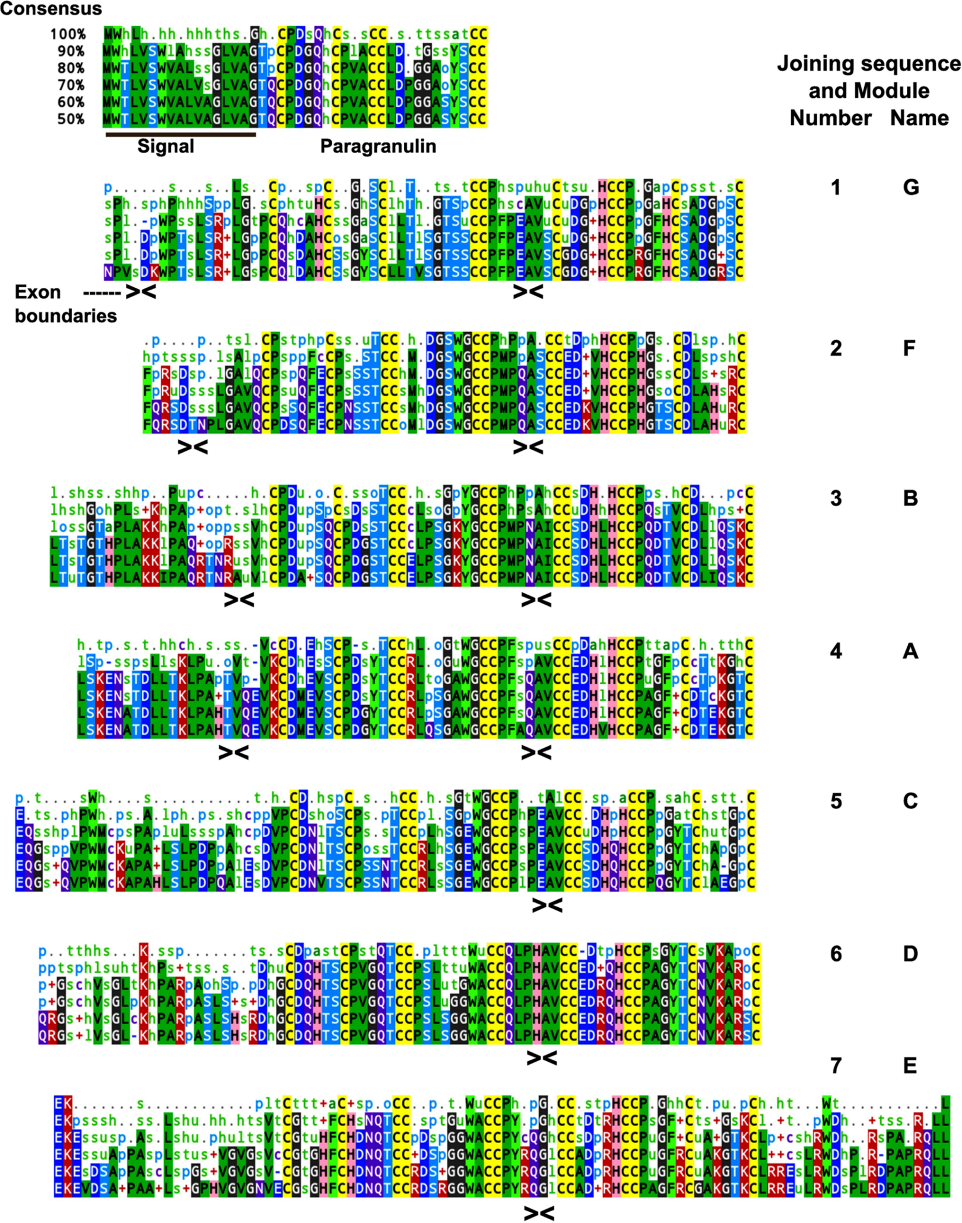
Consensus sequences for mammalian progranulin. The mammalian polypeptide sequences were aligned at five levels of identity form 100% to 50%. Cysteine residues are highlighted in yellow. Amino acids are in capital letters on a background colour. Other letters and symbols are the default Mview notation for residue categories, (see methods for details). The colour scheme was adjusted to facilitate coordination with colours used in 3D models. The signal peptide is underlined. J indicates a joining sequence between modules, the granulin modules are identified by number and letter designations and the positions of exon boundaries are indicated by arrowheads. Paragranulin is a half granulin module at the N-terminus of mammalian progranulin.

**Fig 9:**
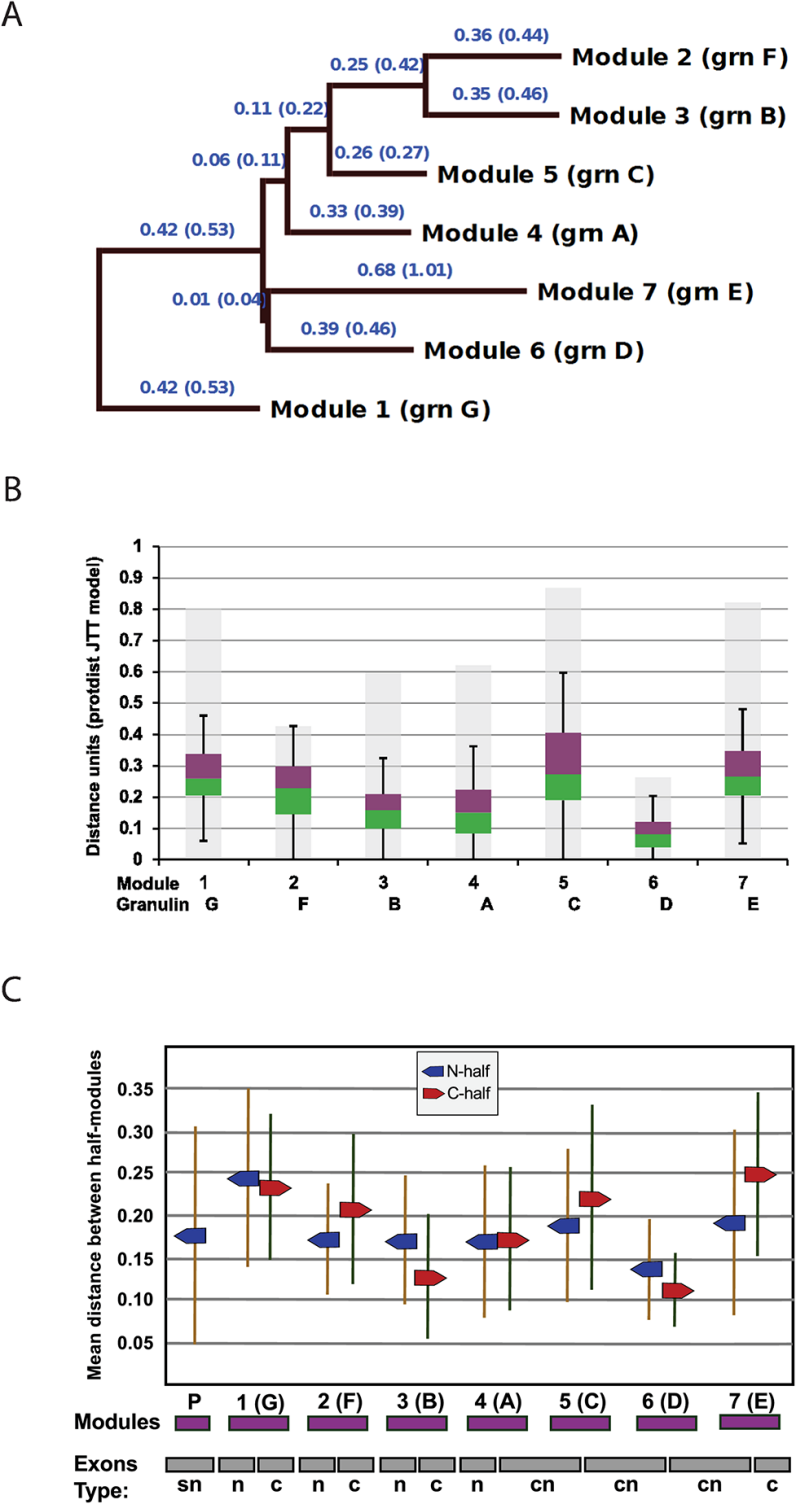
Interspecies variability within mammalian granulin modules. (A) To calculate distance relationships between mammalian consensus granulin modules the aligned consensus sequences for the 7 full modules in mammalian progranulin were analyzed with PROTDIST followed by FITCH. The JTT matrix was used, and global rearrangements applied. Branch lengths in parentheses result from removing the 12 conserved Cys residue positions from the analysis. (B) To determine the degrees of sequence variability for each granulin module the **s**equence distances between 37 species were calculated for each module using the Phylip Protdist program (JTT matrix). The data are represented by a box plot. The median divides the box with green down to the 1st quartile and purple up to the 3rd quartile. The background grey bars encompass all distances including outliers. (C). Diversity at the nucleotide level was determined by calculating the pairwise distances between DNA sequences from 37 species for each half module using DNAdist with the LogDet method. The means and standard deviations are plotted for each N-half, including the paragranulin, p, and each C-half in a way which shows their relationship to the modular structure of the progranulin and to its underlying exon structure.

Structurally conserved polypeptide features are likely to have important roles in the function and structural stability of progranulin and its modules. Polypeptide consensus sequences for the 37 mammalian progranulins listed in Table 1 are shown in Fig 8. The distance relationships between module consensus sequences are shown in the tree diagram in Fig 9A. Module 1 (granulin G) is used as the outgroup because it is the most distant from its closest module. This is not simply because it has the 10-cysteine version of the granulin motif. It is still scored as the most distant if the positions of the missing cysteines are removed from the analysis, or when all 12 cysteine positions are excluded (shown by parenthetical branch lengths). The next most distant from any other is module 7 (granulin E), which is at the opposite end of the progranulin molecule. Ignoring the possibility of differences in functional constraints acting upon the evolution of module structures, the tree supports the hypothesis that the first and last modules, 1 and 7, are the oldest, and that the others in between have diverged from each other over shorter time periods and result from duplication events.

The degree of interspecies amino acid variability within mammalian granulin modules calculated by protein distance analysis is shown in Fig 9B. To assess the variability of the corresponding nucleotide sequences the equivalent half modules, rather than full modules, were analyzed (Fig 9C) as each module is encoded by a combination of two N, C or CN type exons (Fig 1). A striking feature is the exceptionally tight conservation of module 6 (granulin D) suggesting it may play an important role either in progranulin or as a cleaved peptide. The high conservation of granulin D is evident again in the nucleotide sequences of its C-half and N-half modules. Interestingly, the C-half of granulin D is encoded in a CN-type exon which also encodes the N-half of the next module, granulin E (Fig 1), and there is a large difference in variability between the granulin D end of this exon and the downstream granulin E end of this exon. This shows clearly that evolution at the level of the granulin modules dominates over evolution at the level of the exon.

**Fig 10:**
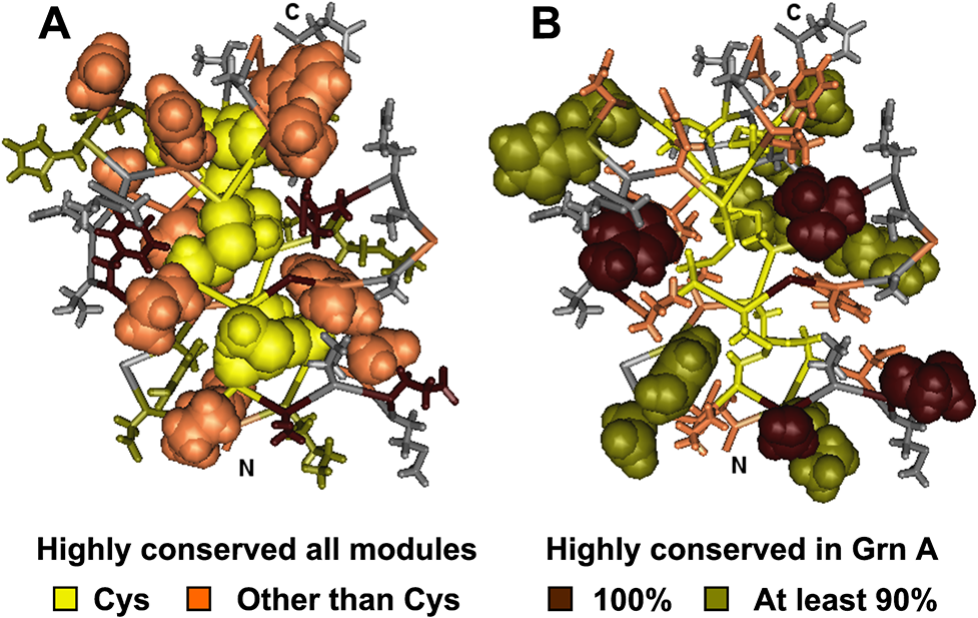
Mapping conserved and variable residues onto the granulin fold. The 3D structure of the well-folded granulin A (MMDB ID: 63884; PDB ID: 2JYE) was used as a model for illustrating conserved and variable residues in mammalian granulin modules. A: Highly conserved residues in all granulin modules are rendered with space-fill side chains. With no side chain, conserved glycine shows only as tube backbone. B: The residues highly conserved within granulin A in 37 mammalian species are shown with space fill rendition of side chains. Colouring is specified in the key. More variable residue positions are shown in grey. (Amino acids depicted as coloured tube backbones in A correspond to the space-filled residues in B, and vice-versa).

The well-defined, three-dimensional structure of human granulin A [4] was used to map the spatial positions of conserved and variable residues (Fig 8) in mammalian granulin modules (Fig 10). Residues of the core granulin motif that are conserved in all modules (Fig 10A) tend to be concentrated internally. Residues that are specifically conserved in granulin A but vary in other modules (Fig 10B) are found largely at the surface with greater potential to make contacts with other modules or progranulin binding proteins. The most variable residues, shown only as grey tube structures in the Fig 10 are predominantly at the surface.

Using the full data set of mammalian granulin module sequences and the known three dimensional structures of the GrnA module as a template, three dimensional folds were modelled for all granulin modules as described in the methods (S6 Fig). Based upon these models, all granulin modules are predicted to adopt similar three dimensional structures. Comparing the backbone structures, GrnF is most closely superimposable upon GrnB, while module GrnD is closely superimposable upon GrnE. The GrnF and GrnB modules are adjacent along the progranulin backbone near the N-terminus while GrnD and GrnE occur beside each other near the C-terminus (Fig 1). The presence of adjacent pairs of spatially highly similar module conformations in different regions of progranulin may have implications for functional specialization along the backbone of progranulin.

In previous research a recombinant polypeptide corresponding to GrnF promoted proliferation of breast cancer cells effectively replicating the activity of full length progranulin, whereas GrnA had opposite activity and was strongly anti-proliferative [4]. Other module peptides showed little activity in this assay. Residues specifically conserved either in GrnF or GrnA may have a major role in conferring these mitotic or antimitotic activities respectively. In GrnF 12 residues, excluding the conserved granulin core motif, are 100% conserved, 10 of which are in the amino terminal half domain. In GrnA, excluding the conserved granulin core motif, 11 residues are 100% conserved, and all are found in the amino-terminal half domain. Six of the fully conserved residues in GrnA differ from the equivalent position in GrnF, and may therefore be weighted towards conferring the A-like biological activity.

**Fig 11:**
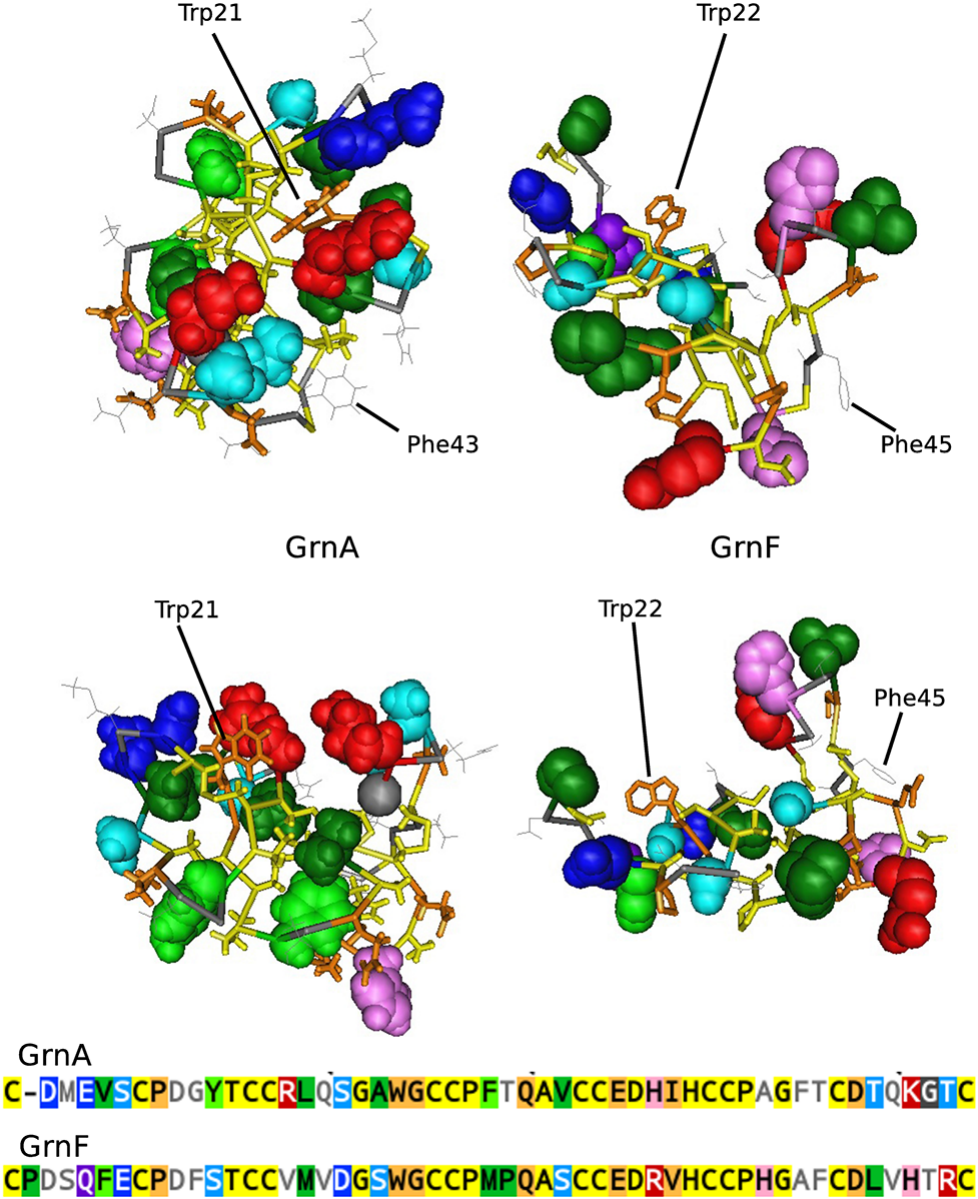
Comparison of the spatial placement of conserved residues in GrnA and GrnF. The GrnA (right) and GrnF (left) modules are shown in two orientations comparing the conformation around the common tryptophan (Trp21, GrnA and Trp22, GrnF) is shown in brown tube style. The space fill side chains are those which show module-specific high conservation. Residues conserved in all granulin modules are shown only as tube backbones coloured yellow for cysteine and black for those highly conserved in all granulin modules. The most variable residues are coloured grey. The colour coding for the space filling side chains follows the conventions in Fig 8. Red side-chains are basic, dark blue residues are acidic.

The concentration of module-specific conserved residues within the amino-termini suggests that this region is particularly important in determining the activity of the modules either as individual granulin peptides or in full length progranulin. In both modules the sequence spanning from Cys3Cys4 to Cys7Cys8 (residues 13 to 32 in GrnA) is highly conserved. Excluding the invariable cysteines, 11 and 9 out of 14 of these residues are conserved at the 90% cut-off in GrnF and GrnA respectively. Part of this sequence, notably GxWGC_5_C_6_P occurs in several modules (Fig 8), and may therefore be important in establishing the generic granulin fold. Using the easily recognizable Trp21 (GrnA) or Trp22 (GrnF) as a positional marker, comparison of GrnA and GrnF modules shows striking differences in the molecular environment surrounding this region which may contribute to the known differences in their biological activities (Fig 11). The Trp21 of GrnA is displaced relative to Trp22 of GrnF, and appears less exposed, with two highly conserved acidic groups (Asp2, Glu4, in blue) and a conserved basic residue, Arg15, (red) providing a more charged environment than in the GrnF module. It is hoped that future biochemical studies will define how the various conserved moieties identified here contribute to biological activity of their respective modules.

## Conclusions

The evolution of progranulin and granulin modules provides insight into many aspects of the biology of the *Grn* gene family from its pre-metazoan origins to the identification of key amino acid residues in mammalian granulin domains. The granulin module arose early in eukaryotic evolution. Whereas metazoa possess only one class of *Grn* gene, at least three categories of *Grn* genes were identified in unicellular eukaryotes. One class includes genes with a domain architecture of multiple granulin-repeats that strongly resembles the metazoan progranulins. These genes occur in the choanoflagellates and filasterea which are considered the closest extant single-celled organisms to the metazoa [64]. Metazoan multicellularity required the evolution of proteins needed for cell adhesion, for the formation of extracellular matrices and for extracellular signalling. Although choanoflagellates possess cadherins and other adhesion proteins [74, 75] the extracellular matrix proteins and secreted regulatory proteins that characterize even the earliest metazoan taxa are absent [76, 77]. The presence, therefore, in unicellular organisms of an extracellular protein, progranulin, that is fully metazoan-like in its module usage is highly unusual. The cadherin [74] and integrin-like proteins [75] of unicellular organisms close to the metazoa are taken as evidence that these genes contributed to the transition from unicellular to multicellular animals through a process of gene cooption [75]. We suggest that this applies also to progranulin. Choanoflagellates and apusozoa possess a second family of genes that encode a single granulin module linked to a larger protein, as seen also in plants. This type of module usage is absent from metazoan organisms. The occurrence of proteins with both pre-metazoan and metazoan-like granulin module usage in choanoflagellates points to a transitional stage of evolution, at least for granulin module-containing genes, in which proteins of both unicellular and metazoan module usage coexist within the same organism. The *Grn* genes of slime molds represent the third variation and are the simplest in structure, with a single granulin module linked only to a short N-terminal tail. The timing of major milestones in early eukaryotic evolution has been estimated using molecular data and fossil records [78]. Applying this chronology to *Grn* genes suggests that the granulin module appeared more than one billion years ago.

The roles of early *Grn* genes are not certain. Plant granulin-cysteine proteases have functions in programmed cell death [79] and in the plant immune response [80–82] in which the plant granulin modules may act as protein aggregation domains [81, 83]. Progranulin is present in neuronal and epithelial cells in the marine ragworm *Hediste diversicolor* [84], the nematode *Caenorhabditis elegans* [85] and vertebrates [12, 38] demonstrating that an association of *Grn* expression with epithelia and neurons was established early in animal evolution and before the separation of deuterostomes from protostomes. Roles for invertebrate progranulin have been postulated in the male reproductive system of flatworms [86], in the flatworm response to radiation damage [87], in the regulation of apoptosis in *C*. *elegans* [85, 88], and as a secreted mitogen in the carcinogenic liver fluke, *Opisthorchis viverrini* [89]. These activities are compatible with the actions of mammalian progranulins [3], and suggest a degree of conservation in the functions of progranulin.

While *Grn* genes have been conserved in almost all metazoan species examined, the size of the *Grn* gene family in each species is always small. This is in contrast to many other gene families based on compact protein modules where the module recurs in many genes. Why the *Grn* gene families are so circumscribed remains an open question. Although always based on repeats of granulin modules, the metazoan *Grn* genes show considerable variability between different taxa with increases or losses in the number of granulin modules per gene. The variability in module content of *Grn* genes is clearly due to exon duplication or loss. A recurrent feature of *Grn* evolution is the frequency with which multiple copies of identical or closely similar modules are repeated in the same gene. This occurs independently throughout metazoan pylogeny but its origins remain unclear.

The vertebrate *Grn* genes illustrate how gene families vary through evolution. Of the three vertebrate orthology groups only one, the *GrnA*-group genes, is present in cartilaginous fish, ray-finned fish and tetrapods. *GrnB* genes are found only in ray-finned fish and are paralogous to the *GrnA*-group genes. GrnC-like genes are present as short-form *Grn* genes in ray-finned fish, but as long-form *Grn* genes in elephant shark and coealacanth genomes. Comparison of the genomic sequences from zebrafish and coelacanth [90] has revealed the loss of 55 genes in land vertebrates. Many of the genes lost in the transition to land-based life have functions clearly specific to fish, such as fin development [90]. The specific loss of a tetrapod orthologue for fish short-form *GrnC* implies that it too proved redundant in land vertebrates, although for reasons that are unclear.

Combining sequence conservation data with polypeptide conformational analysis may help unravel structure and activity relationships of the granulin modules. Conserved residues of the core granulin motif, common to all modules, generally reside internally to the granulin fold and are likely to be important in maintaining the integrity of the granulin fold. In contrast residues that are conserved within a module but show intramodular variation are more often surface exposed. These module-specific conserved residues are likely to impart the individual biological ‘character’ of each module and their surface accessibility may facilitate interactions with other proteins or other regions of progranulin. In general module-specific conserved residues concentrate in the amino terminal region.

In summary, progranulin can be tracked to genes in ancestral unicellular organisms that were incorporated into the metazoan genome early in the development of the extracellular regulatory protein network of multicellular animals. In vertebrates, *Grn*s provides examples of tetraploidization, linear gene duplications, structural plasticity of gene structures due to duplications and deletions of exons, and gene loss. Human progranulin has important roles in disease. The patterns of conservation and variation among granulin modules, particularly when superimposed on the three dimensional structure of a granulin module, may provide a means to rationally predict structural features that govern the biological function of progranulin and granulin peptides and ultimately assist the development of new therapeutic agents based on the granulin structure.

## Supporting Information

S1 Fig Comparison of the genomic context for progranulin A-like genes (*GRNA*) from *Danio rerio* (chromosome 3 XP_001330864 GrnA, *D_rerA* in Figs 5 and 6), *Oreochromis niloticus* (unplaced genomic scaffold, Orenil1.0 scaffold00019 XP_003442564.1, *O_nilA* in Figs 5 and 6), *Takifugu rubripes* (Chromosome 1 XP_003961158, *T_rubA* in Figs 5 and 6). Images were obtained from NCBI Gene and are centered on the respective *Grn* genes. The bar across the top of each panel gives gene positions in Kb along the chromosome or scaffold. Genes that flank *GRNA* in two or more of the three genomes are in upper case. *60SRPL3*: 60S ribosomal protein L3-like, *60SRPL27*: ribosomal protein L27-like, *BCAAT*: branched-chain-amino-acid aminotransferase, cytosolic-like, *BHSD*: 17-beta-hydroxysteroid dehydrogenase 14-like, *CCG2*: voltage-dependent calcium channel gamma-2 subunit-like, *GRNA*: progranulin A, *grnaas*: granulin-a-antisense*GRN-like*: Short variant progranulin-A-like, *IFP 35*: interferon-induced 35 kDa protein homolog, *knca7*:potassium voltage-gated channel, shaker-related subfamily, member 7, *NMLI*: N-myc-interactor-like, *ppf1a3*: protein tyrosine phosphatase, receptor type, f polypeptide, *RUNX1*: RUN domain-containing protein 1-like, SCLC25: solute carrier family 25 member 39-like
(PDF)

S2 Fig Comparison of the genomic context for progranulin B-like genes (GRNB) from *Danio rerio* (chromosme 24 NP_997903, *D_rerB* in Figs 5 and 6), *Oreochromis niloticus* (unplaced genomic scaffold, Orenil1.0 scaffold00068 XP_003448753.1, *O_nilB* in Figs 5 and 6) and *Takifugu rubripes* (chromosome 1 XP_003961158.1, *T_rubD* in Figs 5 and 6). Images were obtained from NCBI Gene and are centered on the respective *Grn* genes. The bar across the top of each panel gives gene positions in Kb along the chromosome or scaffold. Genes that flank *GRNB* in two or more of the three genomes are in upper case. Pseudo genes are in shaded rectangles. *ARF-1*: ADP-ribosylation factor-1 like; *c18orf45*: transmembrane protein C18orf45 homolog, *CRFR-1*: Corticotropin-releasing factor receptor-1 like, *coasy*: Coenzyme A synthase, *FAM171A2*:Protein family 171A2 like, *FORM*: formin-like protein-1, *GRB7*: Growth factor receptor-bound protein-7 like, *GRNB*: Progranulin-b like, *kat8*: KAT8 regulatory NSL complex subunit-1 like, *MAPT*: Microtubule-associated protein Tau (Pseudo gene in *Oreochromis*), *MAPKK14*: Mitogen activated protein kinase kinase-14 like, *mll1*.*mll*: MLL1/MLL complex subunit KIAA1267-like, *naglu*:N-cetylglucosaminidase, alpha, *pus3*:pseudouridylate synthase 3, *roik3*: RIO kinase 3, *SRCKI*: SRC kinase signaling inhibitor 1-like, *thap4*: THAP domain-containing protein 4.
(PDF)

S3 Fig The genomic context of the short-form *Grn* genes from different ray-finned fish species suggests they arose from a single common ancestor. The genomic context for short-form granulin genes from *Danio rerio* (chromosome 19, NP_001018638 and NP_997921, *D_rer1* and *D_rer1* in Figs 5 and 6), *Oreochromis niloticus* (Nile Tilapia) unplaced genomic scaffold, Orenil1.0 scaffold00238 XP_003457316.1, XP_003457317.1, XP_003457303.1, XP_003457318.1, *O_nilC1*, *C2*, *C3* and *C4* in Figs 5 and 6) and *Takifugu rubripes* (Chromosome 12, XP_003969441.1, *T_rubC* in Figs 5 and 6) are compared. Images were obtained from NCBI Gene and are centered on the respective *Grn* genes. The bar across the top of each panel gives gene positions in Kb along the chromosome or scaffold. Genes that flank the short-form granulin genes in two or more of the three genomes are in upper case. Pseudo genes are in shaded rectangles. *BIO*: biotinidase-like, *C9Orf23-like*: transmembrane protein C9orf123 homolog, *CCDC132*: coiled-coil domain-containing protein 132-like, *CDK6*: cyclin-dependent kinase 6-like, *efcab1*: EF-hand calcium binding domain 1, *FAM133b*: family with sequence similarity 133, member B, *granas*: granulin antisense, *GRN*: short-form granulins, *HEPACAM2*: HEPACAM family member 2-like, *illr4*:immune-related, lectin-like receptor 4, *rbm48*: RNA binding motif protein 48, *sfpq*: splicing factor proline/glutamine rich, *SNX13*: sorting nexin-13-like, *SPP6RA*: serine/threonine-protein phosphatase 6 regulatory ankyrin repeat subunit A-like, *ZF-un*: zinc finger similar to zmym4 (Blast), *zmym4*: zinc finger, MYM-type 4. Note that the most recent versions of NCBI Gene do not identify *Oreochromis niloticus* LOC100693202 (*Grn2* in the figure) as a *Grn*, calling it instead “uncharacterized”. Previous version of NCBI Gene assign this gene as a *Grn*. As BLASTp analysis of the translated sequence clearly identified a complete granulin module in this sequence that is strongly homologous with other *Grns* from *Oreochromis niloticus* and *Oreochromis mossambicus* we have retained the older designation.
(PDF)

S4 Fig Multiple small form fish Grn genes (GrnC) arise by exon deletions. In the example shown a 3 module GrnC gene (ggg) can give rise to additional forms by loss of exons resulting in gg, which can be reached by two distinct exon loss pathways exist, as well as gp, and gpp where g is a full granulin module and p a half granulin module corresponding to the first six cysteines of g. Species abbreviations are as in Fig 5, and location in trees (top line) can be obtained from Figs 5 and 6.
(PDF)

S5 Fig The *C_mil2 Grn* gene from elephant shark belongs to the small form *Grn* gene orthology group. Genes on the Ensembl scaffold_4:14,537,823–14,565,096 that flank the *C_mil2 Grn* gene from the elephant shark *Callorhinchus milii* (identified as Grn in figure and highlighted in green) were compared with genes that flank the small form Grn genes of Danio rerio and the corresponding syntenic region on human chromosome 7. Genes circled pink are syntenic with small form (*GrnC*) genes from the zebrafish and/or the equivalent region of the human chromosome 7. Abbreviations not given in full in the figure are *CALM1*, calmodulin 1; CDK6: cyclin-dependent kinase 6-like; *efcab1*: EF-hand calcium binding domain 1; *FAM133b*: family with sequence similarity 133, member B; *IGF2BP3*, Insulin-Like Growth Factor 2 MRNA Binding Protein 3; *MGAT5*, Mannosyl (Alpha-1,6-)-Glycoprotein Beta-1,6-N-Acetyl-Glucosaminyltransferase; MPP6, Membrane Protein, Palmitoylated 6 (MAGUK P55 Subfamily Member 6; NPY, Neuropeptide Y; PEX1 peroxisome biogenesis factor 1; rbm48: RNA binding motif protein; SAMD9L Steril alpha motif domain-containing protein 9-Like; Tra2a, Transformer 2 Alpha Homolog (Drosophila)
(EPS)

S6 Fig 3D models for the 7 modules in human progranulin. Based upon the 3D structure of granulin A (module 4) provided in 2JYE.pdb, 3D models for the other 6 modules were developed using Swiss Model. The fold quality (by ProQ2) is illustrated in the top row with blue as high to red as low. Structures were prepared for presentation using Cn3D. Since the modeling failed to include flanking residues for some modules, only the fold from first to last cysteine is shown. In rows 2 to 4 all side chains are rendered as space fill except those most conserved in all modules. In rows 5 to 7 the space fill is used to display only those residues most conserved within the module based upon the data of Fig 8. Since glycine has no side chain, space fill style for glycine was applied to the backbone (dark grey sphere). Colouring of residues is consistent with the colouring in Fig 8. The 2D projections are as follows: R and L have the N-terminal at the right or left respectively, being rotated approximately 180 degrees about the vertical axis from each other. T is a "top" view, being a rotation relative to R of approximately 90 degrees forward about the horizontal axis.
(TIFF)

S1 Table *Grn* gene sequence predictions. *Grn* gene sequences from public databases were evaluated, and where necessary corrected.
(PDF)

S2 Table Alignments of granulin modules used to generate dendrograms in Fig 5 and Fig 6. See Methods for details of the alignment procedure.
(TXT)

S3 Table Premetazoan granulin genes. Polypeptide sequences of granulin modules obtained from the genomes of premetazoan unicellular organisms including choanoflagellates, filasterea, apusozoa, amoebazoa and unicellular plants.
(DOC)

S4 Table The fish *Grn* gene family. Schematic representations of granulin protein and gene structures from a representative selection of fish.
(DOC)

S5 Table The genomic environment immediately flanking human *GRN* and coelacanth *L_ChaA* demonstrate that the coelacanth and mammalian genes are orthologues. (DOCX)

S6 Table The loss of short-form *Grn* genes in tetrapods. Genes immediately flanking the short-form *Grn* genes of *Danio rerio* (*D_rer1* and *D_rer2* in Figs 5 and 6) were subjected to synteny analysis against the human genome using the Syntenty Database program. 40 of 46 *Danio* genes flanking *Grn1* and *Grn2* have orthologues in the human genome in two major synteny groups. Short-form Grns were probably lost from the tetrapod lineage in a discrete event rather than as a block deletion of multiple genes.
(DOC)

S1 Text Bikonts other than *Viridiplantae* lack easily identifiable genes encoding granulin modules. (DOCX)

S2 Text Variations on the granulin motif. (DOCX)

S3 Text Progranulin exon structures. (DOCX)
