# Supplementary material for: The Evolution of the Secreted Regulatory Protein Progranulin
Source: PLoS One. 2015 Aug 6;10(8):e0133749. doi: 10.1371/journal.pone.0133749 (PMC4527844; doi:10.1371/journal.pone.0133749)

LONG FORM PROGRANULIN_A of Danio rerio

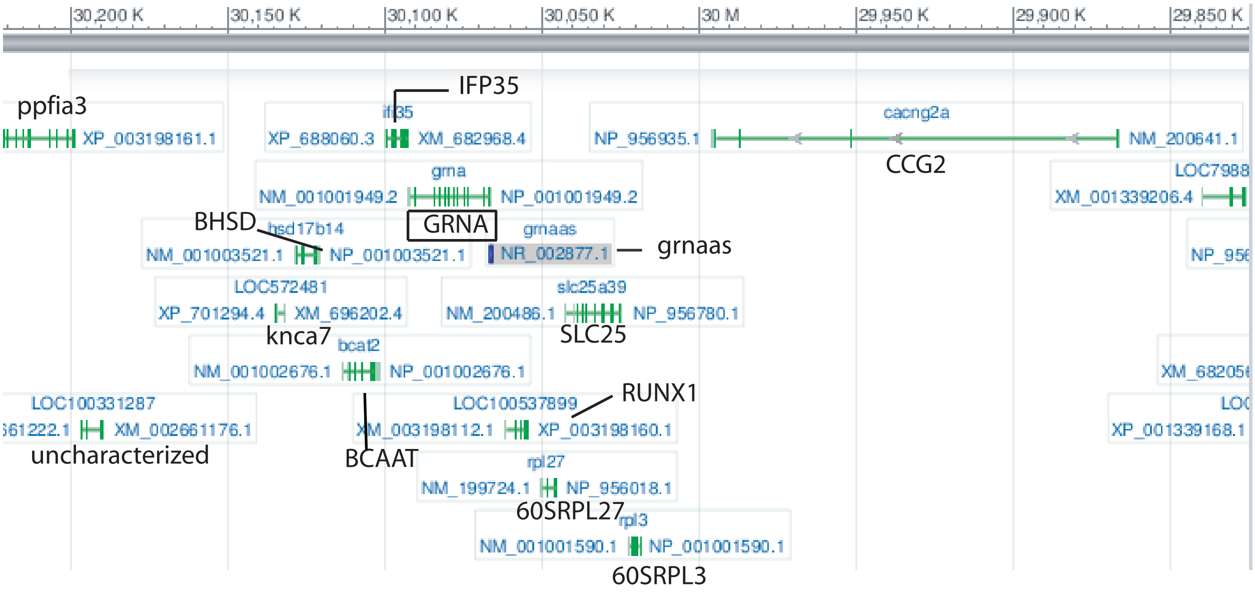

LONG FORM PROGRANULIN_A OF Oreochromis niloticus

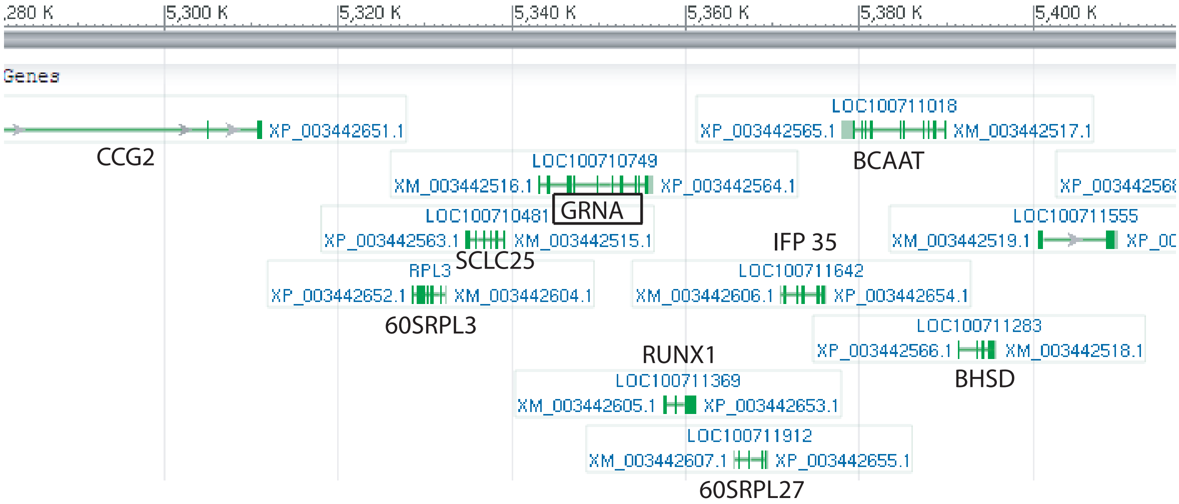

SHORT PROGRANULIN_A RELATED PROTEIN OF Takifugu rubripes

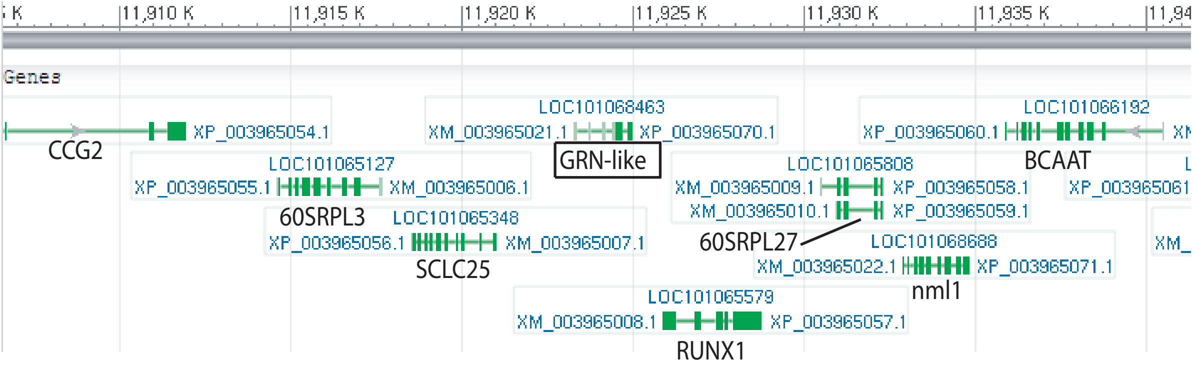

Supplement: S1 Fig — Images were obtained from NCBI Gene and are centered on the respective Grn genes. The bar across the top of each panel gives gene positions in Kb along the chromosome or scaffold. Genes that flank GRNA in two or more of the three genomes are in upper case. 60SRPL3: 60S ribosomal protein L3-like, 60SRPL27: ribosomal protein L27-like, BCAAT: branched-chain-amino-acid aminotransferase, cytosolic-like, BHSD: 17-beta-hydroxysteroid dehydrogenase 14-like, CCG2: voltage-dependent calcium channel gamma-2 subunit-like, GRNA: progranulin A, grnaas: granulin-a-antisenseGRN-like: Short variant progranulin-A-like, IFP 35: interferon-induced 35 kDa protein homolog, knca7:potassium voltage-gated channel, shaker-related subfamily, member 7, NMLI: N-myc-interactor-like, ppf1a3: protein tyrosine phosphatase, receptor type, f polypeptide, RUNX1: RUN domain-containing protein 1-like, SCLC25: solute carrier family 25 member 39-like (PDF) [file pone.0133749.s001.pdf]
