# Supplementary material for: The Evolution of the Secreted Regulatory Protein Progranulin
Source: PLoS One. 2015 Aug 6;10(8):e0133749. doi: 10.1371/journal.pone.0133749 (PMC4527844; doi:10.1371/journal.pone.0133749)

LONG FORM PROGRANULIN_B of Danio rerio

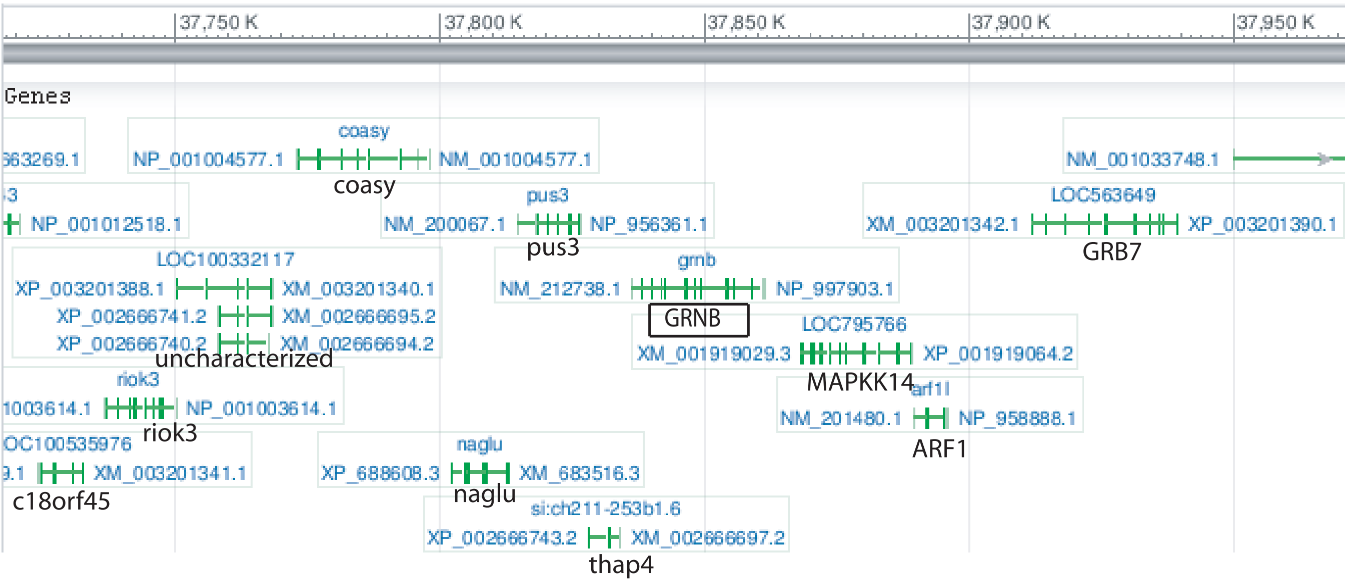

LONG FORM PROGRANULIN_B OF Oreochromis niloticus

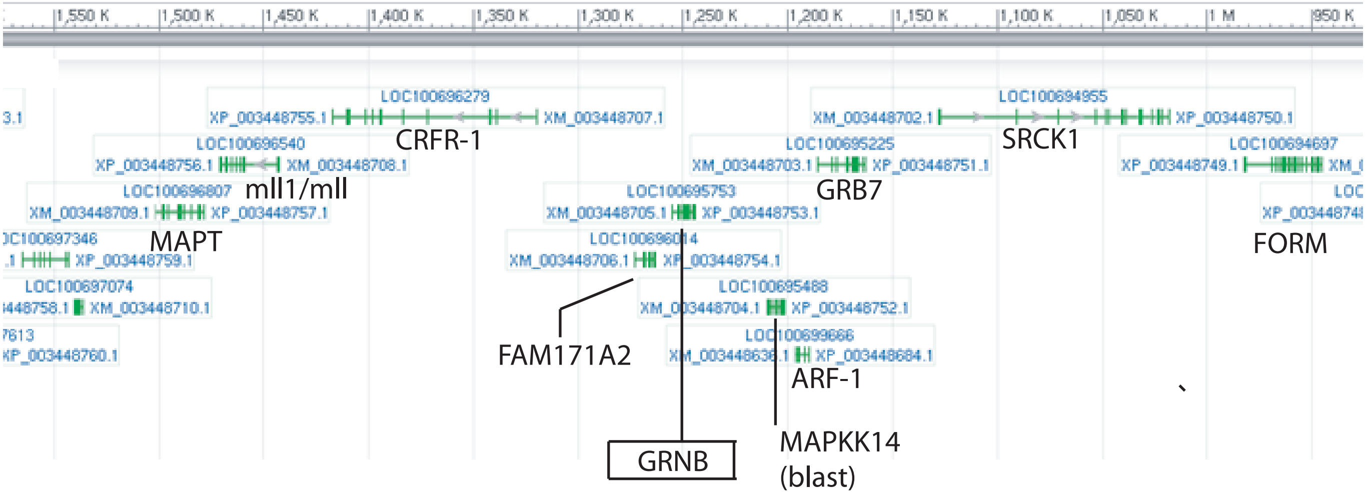

LONG FORM PROGRANULIN OF Takifugu rubripes -

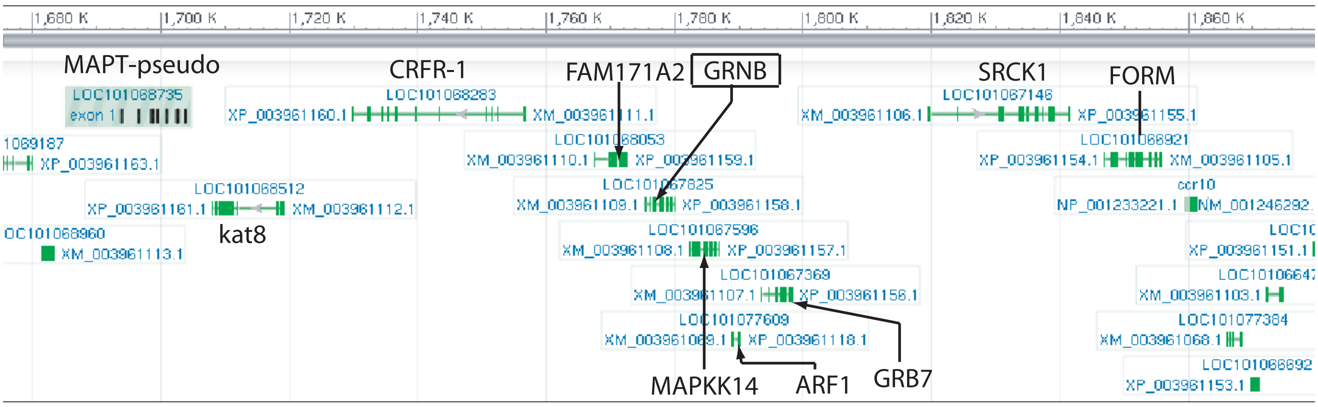

Supplement: S2 Fig — Images were obtained from NCBI Gene and are centered on the respective Grn genes. The bar across the top of each panel gives gene positions in Kb along the chromosome or scaffold. Genes that flank GRNB in two or more of the three genomes are in upper case. Pseudo genes are in shaded rectangles. ARF-1: ADP-ribosylation factor-1 like; c18orf45: transmembrane protein C18orf45 homolog, CRFR-1: Corticotropin-releasing factor receptor-1 like, coasy: Coenzyme A synthase, FAM171A2:Protein family 171A2 like, FORM: formin-like protein-1, GRB7: Growth factor receptor-bound protein-7 like, GRNB: Progranulin-b like, kat8: KAT8 regulatory NSL complex subunit-1 like, MAPT: Microtubule-associated protein Tau (Pseudo gene in Oreochromis), MAPKK14: Mitogen activated protein kinase kinase-14 like, mll1.mll: MLL1/MLL complex subunit KIAA1267-like, naglu:N-cetylglucosaminidase, alpha, pus3:pseudouridylate synthase 3, roik3: RIO kinase 3, SRCKI: SRC kinase signaling inhibitor 1-like, thap4: THAP domain-containing protein 4. (PDF) [file pone.0133749.s002.pdf]
