# Supplementary material for: The Evolution of the Secreted Regulatory Protein Progranulin
Source: PLoS One. 2015 Aug 6;10(8):e0133749. doi: 10.1371/journal.pone.0133749 (PMC4527844; doi:10.1371/journal.pone.0133749)

SHORT FORM GRANULINS OF *Danio rerio*

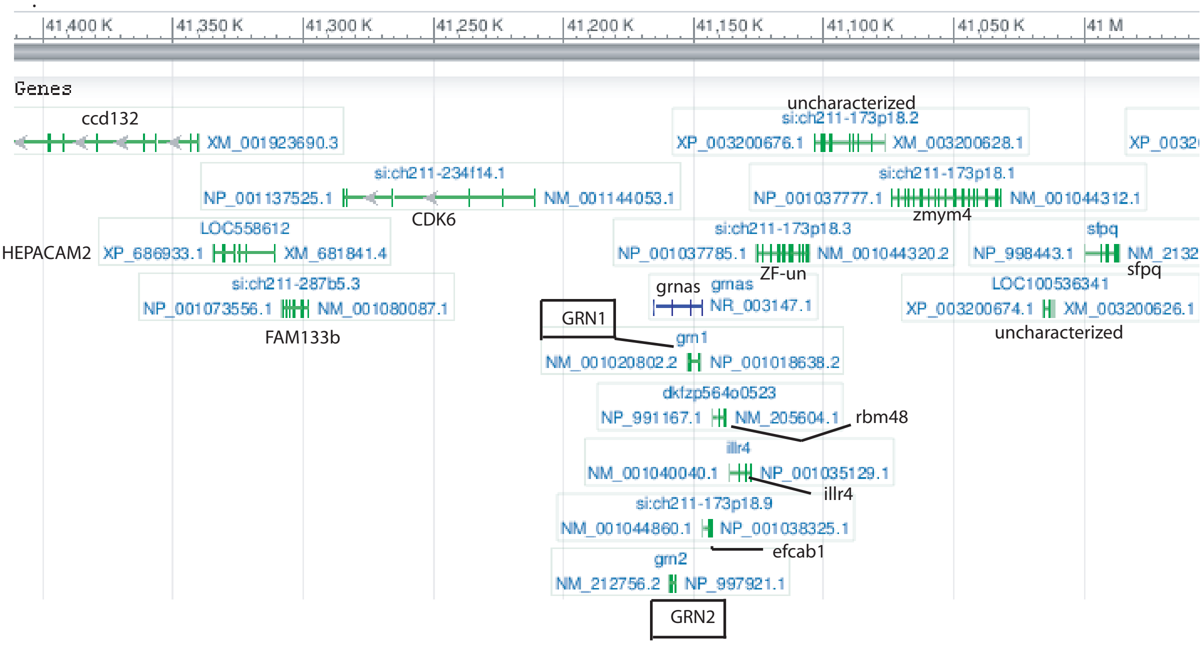


SHORT FORM GRANULINS OF *Oreochromis niloticus* (Nile Tilapia)

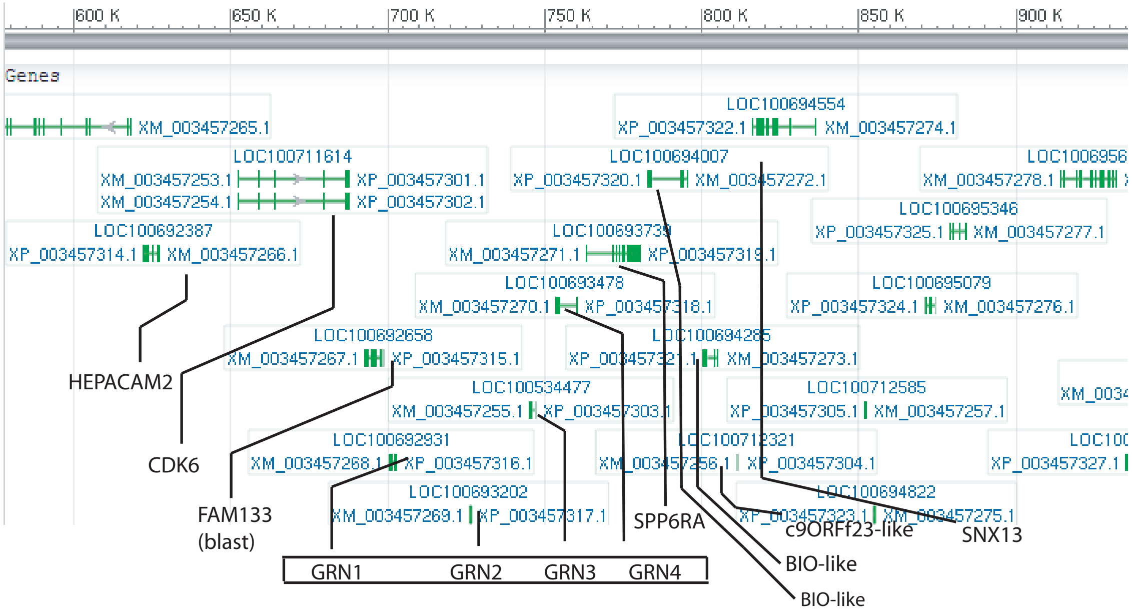


SHORT FORM GRANULIN OF *Takifugu rubripes* (Fugu fish)

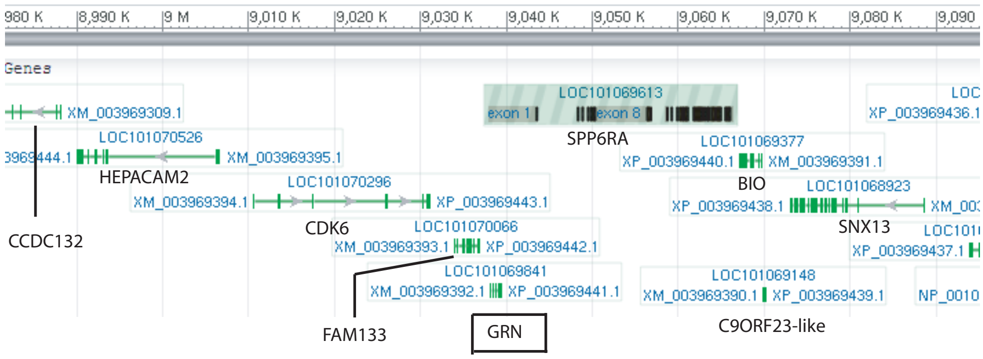

Supplement: S3 Fig — The genomic context for short-form granulin genes from Danio rerio (chromosome 19, NP_001018638 and NP_997921, D_rer1 and D_rer1 in Figs 5 and 6), Oreochromis niloticus (Nile Tilapia) unplaced genomic scaffold, Orenil1.0 scaffold00238 XP_003457316.1, XP_003457317.1, XP_003457303.1, XP_003457318.1, O_nilC1, C2, C3 and C4 in Figs 5 and 6) and Takifugu rubripes (Chromosome 12, XP_003969441.1, T_rubC in Figs 5 and 6) are compared. Images were obtained from NCBI Gene and are centered on the respective Grn genes. The bar across the top of each panel gives gene positions in Kb along the chromosome or scaffold. Genes that flank the short-form granulin genes in two or more of the three genomes are in upper case. Pseudo genes are in shaded rectangles. BIO: biotinidase-like, C9Orf23-like: transmembrane protein C9orf123 homolog, CCDC132: coiled-coil domain-containing protein 132-like, CDK6: cyclin-dependent kinase 6-like, efcab1: EF-hand calcium binding domain 1, FAM133b: family with sequence similarity 133, member B, granas: granulin antisense, GRN: short-form granulins, HEPACAM2: HEPACAM family member 2-like, illr4:immune-related, lectin-like receptor 4, rbm48: RNA binding motif protein 48, sfpq: splicing factor proline/glutamine rich, SNX13: sorting nexin-13-like, SPP6RA: serine/threonine-protein phosphatase 6 regulatory ankyrin repeat subunit A-like, ZF-un: zinc finger similar to zmym4 (Blast), zmym4: zinc finger, MYM-type 4. Note that the most recent versions of NCBI Gene do not identify Oreochromis niloticus LOC100693202 (Grn2 in the figure) as a Grn, calling it instead “uncharacterized”. Previous version of NCBI Gene assign this gene as a Grn. As BLASTp analysis of the translated sequence clearly identified a complete granulin module in this sequence that is strongly homologous with other Grns from Oreochromis niloticus and Oreochromis mossambicus we have retained the older designation. (PDF) [file pone.0133749.s003.pdf]
