# Supplementary figures and images for: The Evolution of the Secreted Regulatory Protein Progranulin
Source: PLoS One. 2015 Aug 6;10(8):e0133749. doi: 10.1371/journal.pone.0133749 (PMC4527844; doi:10.1371/journal.pone.0133749)

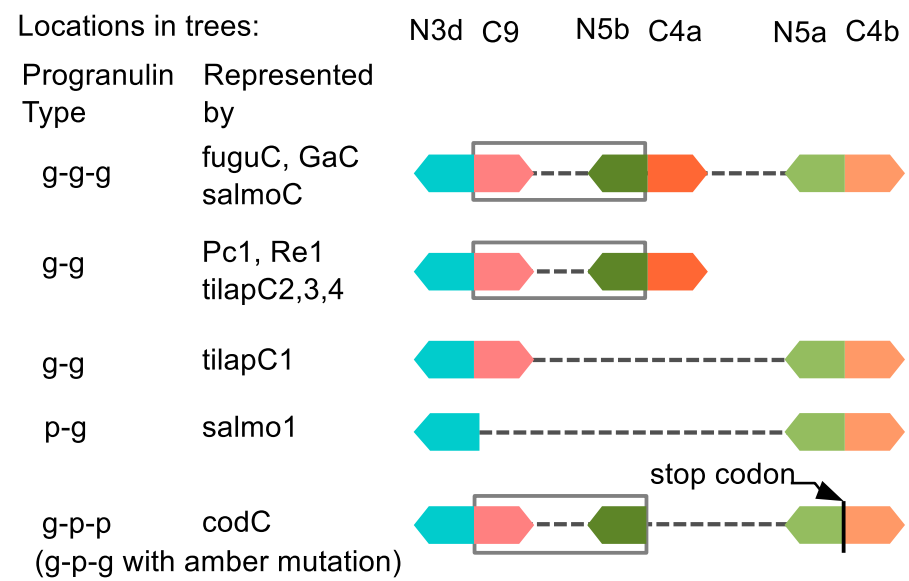

Supplement: S4 Fig — In the example shown a 3 module GrnC gene (ggg) can give rise to additional forms by loss of exons resulting in gg, which can be reached by two distinct exon loss pathways exist, as well as gp, and gpp where g is a full granulin module and p a half granulin module corresponding to the first six cysteines of g. Species abbreviations are as in Fig 5, and location in trees (top line) can be obtained from Figs 5 and 6. (PDF) [file pone.0133749.s004.pdf]

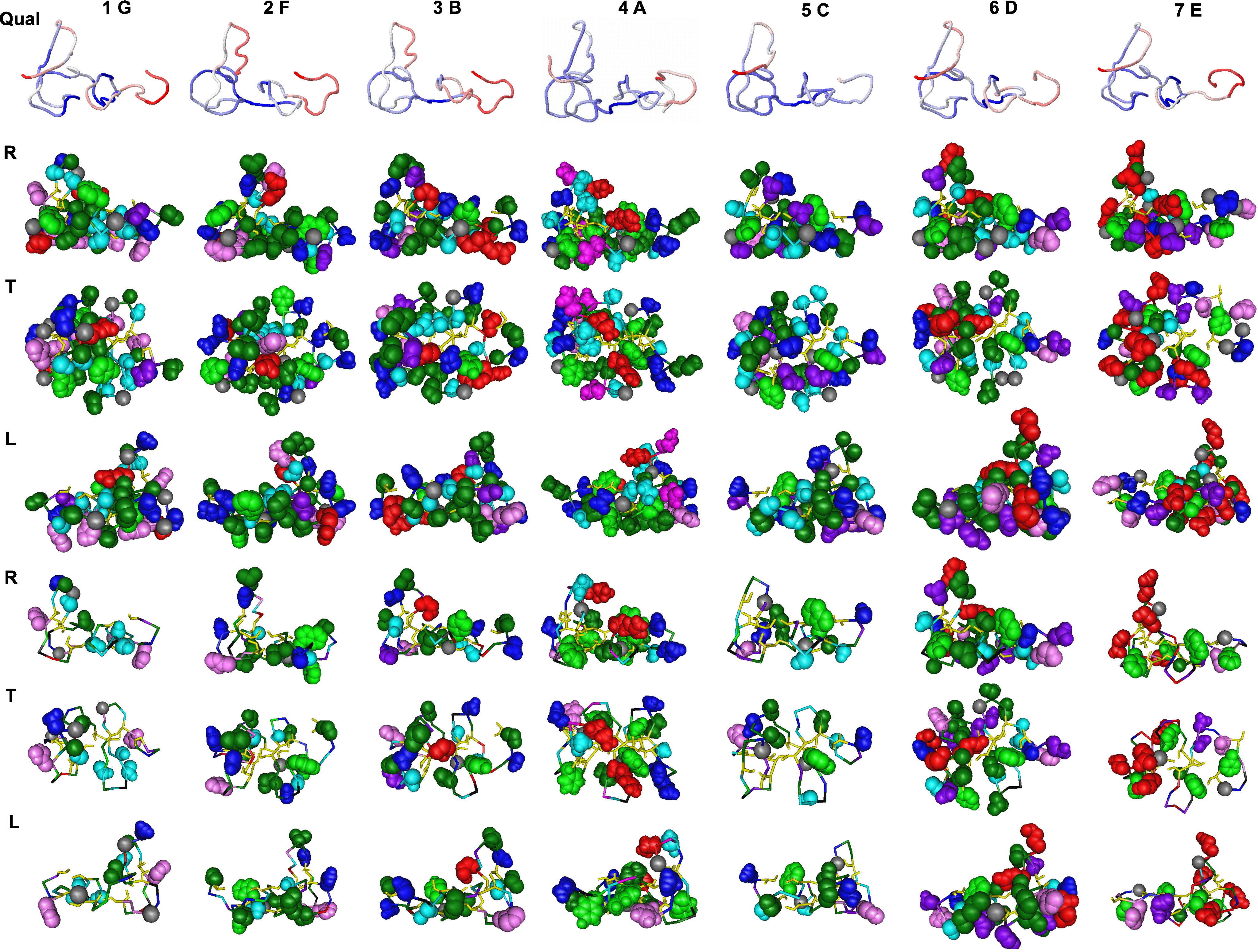

Supplement: S6 Fig — Based upon the 3D structure of granulin A (module 4) provided in 2JYE.pdb, 3D models for the other 6 modules were developed using Swiss Model. The fold quality (by ProQ2) is illustrated in the top row with blue as high to red as low. Structures were prepared for presentation using Cn3D. Since the modeling failed to include flanking residues for some modules, only the fold from first to last cysteine is shown. In rows 2 to 4 all side chains are rendered as space fill except those most conserved in all modules. In rows 5 to 7 the space fill is used to display only those residues most conserved within the module based upon the data of Fig 8. Since glycine has no side chain, space fill style for glycine was applied to the backbone (dark grey sphere). Colouring of residues is consistent with the colouring in Fig 8. The 2D projections are as follows: R and L have the N-terminal at the right or left respectively, being rotated approximately 180 degrees about the vertical axis from each other. T is a "top" view, being a rotation relative to R of approximately 90 degrees forward about the horizontal axis. (TIFF) [file pone.0133749.s006.tiff]
