# Supplementary material for: The Evolution of the Secreted Regulatory Protein Progranulin
Source: PLoS One. 2015 Aug 6;10(8):e0133749. doi: 10.1371/journal.pone.0133749 (PMC4527844; doi:10.1371/journal.pone.0133749)
Supplement: S1 Table — Grn gene sequences from public databases were evaluated, and where necessary corrected. (PDF) [file pone.0133749.s007.pdf]

The evolution of the secreted regulatory protein progranulin: from premetazoan protozoa to humans.

Sequences used in the phylogenetic analysis for manuscript.

Updated: 2015-04-14 (y-m-d)

- Note 1: A predicted transcript sequence is given only when cDNA data provides less of the coding sequence than predictable from genomic data.
- Note 2: A genomic sequence is provided only in special cases. In most cases the exons and the appropriate database accession code are sufficient.
- Note 3: Predicted sequence (if any) is followed by cDNA data (if any), then exons from genomic data, finally a full genomic sequence if necessary.
- Note 4: The names and abbreviations for the proteins containing Grn modules and Grn-related modules were chosen for illustrations and discussion in the manuscript.
- Note 5: Shorthand to indicate that an exon encodes an N- or C-half uses n or c. Variant forms are indicated by n' or n", for example. There can be no consistency with respect to what kind of variant it is. One must read the annotation with the exon for that information.

List of the 48 progranulins described here:

A. queenslandica L   A. queenslandica S1   A. queenslandica S2   A. queenslandica S3   Bovine   Branchiostoma floridae (I)   C. intestinalis (long)   C. milii 1   C. milii 2   Chicken   Danio rerio 1   Danio rerio 2   Danio rerio A   Danio rerio B   G. aculeatus A   G. aculeatus B   G. aculeatus C   G. morhua C   Green Anole   Human   L. chalumnae A   L. chalumnae C   N. vectensis   O. lobularis   O. niloticus A   O. niloticus B   O. niloticus C1   O. niloticus C2   O. niloticus C3   O. niloticus C4   P. lividus   Paralabidochromis chilotes C   Petromyzon marinus Grn-related S1   Petromyzon marinus Grn-related S2   Petromyzon marinus Grn-related S3   Petromyzon marinus Grn-related S4   Petromyzon marinus L (Large or Long form)   R. esox C   S. kowalevskii   S. purpuratus   S. salar 1   S. salar A   S. salar B   S. salar C   T. rubripes B   T. rubripes C   T. rubripes D   X. tropicalis

00040 **A. queenslandica progranulin L**   Short name: **A_queL**   Date entered: 2014-03

Species: ***Amphimedon queenslandica (sponge)***
Taxonomy (via NCBI): *Eukaryota; Metazoa; Porifera; Demospongiae; Ceractinomorpha; Haplosclerida; Niphatidae; Amphimedon.*
General Comment: Four apparent progranulin genes are close together in the genome. This is the first and largest, encoding 15 granulin modules in just 2 exons. At present it seems to be the only one represented by cDNA sequences in the EST database. The other small forms encode 2 or 3 modules, and have just one coding exon. While splicing of the large exon into a downstream granulin sequence is possible (a la ensembl Aqu1.229537), current cDNA evidence only supports termination of the large form in the A_queL coding exon 2.

Protein Sequence

```
MAVKFVLLLLFASVYCRPSLPQSNLYPQLDVVHCPNSEDLCPANNTCCRLTPELWGCCPHIDAYCCSDKR
HCCPRGHPCSADSLNCSDSTTTFPSVLMSFKRQRNRNKPFFESKISDTLSQVAVNVNCTPPGSKESWQCP
ADHTCCYISTTRRYGCCPIVPATCCPDGRGCCPSGYTCDLTDNTCTPPKDSLATLAPTLIDVRPRPTNVT
DIICPNGINQCPDGETCCRVSSDDFGCCHFSNAVCCADMKHCCPSGYMCSPNDGNCIRSQVAILKKKPAI
SISFTNVTEVHCPDGKVCLDGNTCCQTLSGSYGCCPREDAVCCADRVHCCPHGTECKSDGTCTRTNEVKK
TLKLDSVKCPDGGVCLDGETCCRESGYKYGCCSYPDATCCSDGIHCCPSGEICDVARGTCFYGGSAKAMM
KKLSFLRSPIKVKNVVCPDGGSCPDDNTCCLTGDNKYGCCPQPNANCCADKVHCCPSGYSCNVADGTCTR
GVSVVPMMKKLSSLRSPSDVKNVVCPGGRQKCPDDNTCCELESGSYGCCPRPDAVCCADKTHCCPNGYTC
QNDGTCKKDTKVYEALAVVKLDSVKCPDGGVCLDGQTCCRESGYKYGCCPYLDATCCSDGIHCCPSGEIC
DVARGTCFYGDSAKAMIKKLSFLKSPTKVKNVVCPNGRQECPDDNTCCELKSGSYGCCPRADAVCCADKI
HCCPNGYTCENDGTCKRDSKVYEALAVLKLNTVDCPDGGSCPDNNTCCLTGVDKYGCCPQPNAVCCADKV
HCCPKDFSCNTADHTCTKGVSVIPMMKKLPTLGSPTKLKGVVCPNGKNECPDGSTCCSKGDAGYGCCPLA
NAVCCADQTHCCPNGFSCDASNGTCVKEAPVFSALHKVFQPLYNSKVKENVCPDGQSECSDINTCCKTDT
GKYGCCELPQATCCSDMKHCCPSGYICNSDGSCNRQDSFIQIEAVLIKNKVKDVTCSDGNTSCKNDQTCC
LLKDGRYGCCNLTDANCCGDGIHCCPNGYSCDVAKGTCVKEASVIPILKKMMSVSTQVKDDIKCPDDTVC
SDNNTCCKAKSNSFGCCPSPKATCCADEIHCCPSGTTCDLEGHTCRVSEAKHQPLLHVGSMKKPVVVNVV
```

CPSVPGHEEKECSSNSTCCLMSEGNYGCCPFPNAVCCDDKKHCCPFDYACNPDDGTCTDKSKGFLYTIPP
TRMSYKRPLL*

Transcript:

Note: The transcript sequence predicted from ACUQ01000195.1 is mostly confirmed by EST data. In particular, the single splice is confirmed by one EST sequence, and 18 EST sequences confirm the transcription stop. At present, none support a proposed splice into what we are calling S1 or either of the other apparent small form single coding exon genes.

Predicted sequence:

Note: Predicted from ACUQ01000195.1 from translation start codon to stop codon. The first coding exon probably starts at base 7164, 16 bases before the start codon.
Derivation: NCBI wgs ACUQ01000195.1 GI:296313059

ATGGCCGTGAAATTCGTTCTCTTGCTGCTGTTTGCTTCAGTTTACTGTCGTCCTTCTCTTCCACAAAGCA
ACCTTTATCCTCAACTGGATGTAGTTCACTGCCCCAATTCTGAGGACCTCTGCCCGGCTAATAATACGTG
TTGCCGTCTTACCCCGGAGCTCTGGGGATGCTGTCCACACATAGACGCTTACTGCTGCTCTGATAAGCGC
CACTGCTGTCCTCGTGGCCATCCTTGCTCCGCGGACTCCTTGAACTGCTCGGACTCTACTACAACCTTCC
CTTCCGTTTTGATGAGTTTTAAAAGGCAAAGAAACAGAAATAAACCCTTCTTTGAAAGTAAAATTTCAGA
TACATTATCACAAGTTGCCGTTAATGTGAATTGCACTCCTCCTGGTTCAAAAGAGAGCTGGCAGTGTCCT
GCCGATCATACGTGCTGCTATATTTCAACCACTAGGAGATATGGTTGCTGTCCAATAGTACCTGCAACTT
GCTGCCCTGATGGAAGAGGATGCTGTCCATCAGGATACACTTGTGACCTAACCGATAATACATGCACTCC
ACCAAAAGACTCACTTGCAACACTGGCACCGACTCTAATTGACGTGAGACCTAGACCCACCAACGTTACT
GATATAATTTGTCCCAATGGGATAAATCAATGTCCTGATGGTGAAACCTGCTGTCGGGTGAGCAGTGATG
ATTTCGGGTGCTGTCATTTTTCCAATGCTGTCTGCTGTGCTGATATGAAGCACTGCTGTCCTAGCGGATA
TATGTGTAGTCCGAATGACGGTAACTGTATAAGGTCTCAAGTGGCTATACTTAAGAAGAAACCTGCAATT
AGCATATCCTTCACTAACGTGACTGAAGTCCATTGTCCTGATGGTAAAGTATGTCTCGATGGCAATACTT
GCTGTCAGACCCTCTCTGGTAGCTATGGTTGCTGTCCTAGGGAAGATGCTGTTTGTTGTGCTGATAGAGT
TCACTGCTGTCCTCATGGCACTGAATGCAAAAGTGATGGCACTTGCACTAGGACCAATGAGGTAAAGAAG
ACCCTGAAACTTGATTCAGTTAAGTGTCCTGATGGTGGAGTTTGTCTTGATGGTGAAACTTGTTGTCGAG
AAAGTGGTTATAAATATGGTTGTTGTTCTTATCCTGATGCTACCTGTTGCTCTGATGGAATCCATTGCTG
TCCCTCAGGAGAGATATGTGACGTAGCTCGTGGGACTTGTTTTTATGGAGGCTCTGCTAAAGCAATGATG
AAGAAACTGTCATTCTTGAGGTCACCAATTAAAGTTAAAAATGTGGTTTGTCCTGATGGAGGAAGCTGTC
CTGATGATAATACATGTTGTCTAACTGGTGACAATAAGTATGGGTGCTGTCCTCAACCTAATGCTAACTG
TTGCGCTGATAAAGTCCATTGCTGTCCTTCAGGCTATTCTTGTAATGTAGCTGATGGTACTTGTACTAGA
GGGGTCTCTGTTGTACCAATGATGAAGAAATTGTCATCCTTGAGGTCACCATCTGATGTAAAAAATGTGG
TTTGTCCTGGTGGGAGGCAGAAGTGTCCTGATGACAATACTTGCTGTGAATTGGAATCTGGTAGCTATGG
TTGCTGTCCTAGACCAGATGCTGTTTGCTGTGCTGATAAAACTCACTGCTGTCCTAATGGCTATACGTGC
CAGAACGATGGTACCTGCAAGAAAGACACGAAAGTCTATGAAGCACTGGCAGTTGTGAAACTTGATTCAG
TTAAGTGTCCTGATGGTGGAGTTTGTCTTGATGGTCAAACTTGTTGTCGAGAAAGTGGTTATAAATATGG
TTGTTGTCCTTATCTTGATGCTACCTGTTGCTCTGATGGAATCCATTGCTGTCCCTCAGGAGAGATATGT
GACGTAGCTCGTGGGACTTGTTTTTACGGAGACTCCGCTAAAGCAATGATCAAGAAACTGTCATTCTTGA
AGTCACCAACTAAAGTTAAAAATGTGGTTTGTCCTAATGGGAGGCAGGAGTGTCCTGATGACAATACTTG
TTGTGAATTGAAATCTGGTAGCTATGGTTGCTGTCCTAGAGCAGATGCTGTTTGCTGTGCTGATAAAATT
CACTGCTGTCCTAATGGCTACACGTGCGAGAACGATGGTACTTGCAAGAGAGACTCGAAAGTCTATGAAG
CTCTGGCAGTTTTGAAACTCAATACAGTTGACTGTCCTGATGGGGGAAGCTGTCCTGATAATAATACATG
TTGTCTAACTGGTGTAGATAAGTACGGGTGCTGTCCTCAACCTAATGCTGTCTGTTGCGCTGATAAAGTC
CATTGCTGCCCTAAAGACTTTTCTTGTAATACAGCTGATCATACTTGTACTAAGGGGGTCTCTGTTATAC
CAATGATGAAGAAGCTTCCAACGTTGGGCTCACCTACTAAATTAAAAGGGGTTGTATGCCCTAATGGAAA
GAATGAGTGTCCTGATGGTAGCACTTGCTGCAGTAAAGGTGATGCAGGATATGGCTGCTGCCCTTTGGCT
AATGCTGTTTGCTGTGCTGATCAAACCCATTGTTGTCCTAATGGTTTCTCATGTGATGCAAGTAATGGTA
CCTGTGTCAAGGAAGCCCCTGTATTCTCAGCCTTACACAAAGTCTTTCAGCCTCTGTATAATAGTAAAGT
CAAAGAAAATGTCTGCCCTGATGGTCAAAGTGAATGCTCTGATATTAATACATGCTGTAAGACCGATACA
GGAAAGTATGGCTGCTGTGAGTTACCTCAAGCTACCTGCTGCTCTGATATGAAGCATTGCTGTCCAAGTG
GTTACATTTGCAATTCTGATGGCTCCTGTAATAGGCAGGATTCCTTTATACAAATTGAAGCAGTTCTCAT
TAAGAATAAGGTAAAGGATGTCACTTGTAGCGATGGCAATACATCGTGTAAAAATGATCAGACTTGCTGT
CTTCTTAAAGATGGACGCTATGGTTGCTGTAACCTCACTGATGCTAATTGCTGTGGCGATGGGATACATT
GCTGTCCAAATGGTTACTCGTGTGACGTGGCAAAAGGCACGTGTGTAAAAGAAGCATCTGTCATACCAAT
ACTAAAGAAAATGATGTCAGTGTCTACTCAAGTAAAGGATGATATAAAATGTCCTGATGATACGGTTTGC
TCTGACAATAACACTTGCTGTAAAGCAAAGTCTAATTCGTTTGGTTGTTGTCCTTCACCTAAAGCTACCT

GTTGTGCTGATGAGATCCACTGCTGTCCTAGTGGCACAACTTGCGATCTTGAAGGCCATACCTGCAGGGT
CTCAGAAGCAAAGCATCAACCATTACTTCATGTTGGGAGTATGAAGAAACCAGTCGTCGTTAATGTTGTG
TGTCCTTCTGTTCCAGGCCACGAGGAGAAGGAGTGCTCTAGTAACAGCACGTGCTGCTTGATGTCTGAAG
GCAATTATGGTTGCTGCCCTTTTCCGAATGCTGTCTGCTGTGATGACAAGAAGCATTGCTGTCCTTTTGA
TTATGCATGTAACCCTGATGATGGAACATGTACAGACAAATCCAAAGGATTCCTTTACACCATCCCTCCT
ACAAGGATGAGCTACAAGAGGCCTCTCCTGTAA

cDNA data:

From: NCBI est: GI:282424200 and GI:282424199

Note: This cDNA data confirms the splice between coding exons 1 and 2. gi:282424199 is in gi:282424200

TCCTCGTGGCCATCCTTGCTCCGCGGACTCCTTGAACTGCTCGGACTCTACTACAACCTTCCCTTCCGTT
TTGATGAGTTTTAAAAGGCAAAGAAACAGAAATAAACCCTTCTTTGAAAGTAAAATTTCAGATACATTAT
CACAAGTTGCCGTTAATGTGAATTGCACTCCTCCTGGTTCAAAAGAGAGCTGGCAGTGTCCTGCCGATCA
TACGTGCTGCTATATTTCAACCACTAGGAGATATGGTTGCTGTCCAATAGTACCTGCAACTTGCTGCCCT
GATGGAAGAGGATGCTGTCCATCAGGATACACTTGTGACCTAACCGATAATACATGCACTCCCC

From: NCBI est: Contig of GI:(282445370, 282447771, 282447773, 282448561, 282448360, 282452022)

Note: This cDNA contig encodes granulin modules most of 6 to the start of 12.

ATAATACATGTTGTCTAACTGGTGACAATAAGTATGGGTGCTGTCCTCAACCTAATGCTAACTGTTGCGC
TGATAAAGTCCATTGCTGTCCTTCAGGCTATTCTTGTAATGTAGCTGATGGTACTTGTACTAGAGGGGTC
TCTGTTGTACCAATGATGAAGAAATTGTCATCCTTGAGGTCACCATCTGATGTAAAAAATGTGGTTTGTC
CTGGTGGGAGGCAGAAGTGTCCTGATGACAATACTTGCTGTGAATTGGAATCTGGTAGCTATGGTTGCTG
TCCTAGACCAGATGCTGTTTGCTGTGCTGATAAAACTCACTGCTGTCCTAATAGCTATACGTGCCAGAAC
GATGGTACCTGCAAGAAAGACACGAAAGTCTATGAAGCACTGGCAGTTGTGAAACTTGATTCAGTTAAGT
GCCCTGATGGTGGAGTTTGTCTTGATGGTCAAACTTGTTGTCGAGAAAGTGGTTATAAATATGGTTGTTG
TCCTTATCTTGATGCTACCTGTTGCTCTGATGGAATCCATTGCTGTCCCTCAGGAGAGATATGTGACGTA
GCTCGTGGGACTTGTTTTTACGGAGACTCCGCTAAAGCAATGATCAAGAAACTGTCATTCTTGAAGTCAC
CAACTAAAGTTAAAAATGTGGTTTGTCCTAATGGGAGGCAGGAGTGTCCTGATGACAATACTTGTTGTGA
ATTGAAATCTGGTAGCTATGGTTGCTGTCCTAGAGCAGATGCTGTTTGCTGTGCTGATAAAATTCACTGC
TGTCCTAATGGCTACACGTGCGAGAACGATGGTACTTGCAAGAGAGACTCGAAAGTCTATGAAGCTCTGG
CAGTTTTGAAACTCAATACAGTTGACTGTCCTGATGGGGGAAGCTGTCCTGATAATAATACATGTTGTCT
AACTGGTGTAGATAAGTACGGGTGCTGTCCTCAACCTAATGCTGTCTGTTGCGCTGATAAAGTCCATTGC
TGCCCTAAAGACTTTTCTTGTAATACAGCTGATCATACTTGTACTAAGGGGGTCTCTGTTATACCAATGA
TGAAGAAGCTTCCAACGTTGGGCTCACCTACTAAATTAAAAGGGGTTGTATGCCCTAATGGAAAGAATGA
GTGTCCTGATGGTAGCACTTGCTGCAGTAAAGGTGATGCAGGATATGGCTGCTGCCCTTTGGCTAATGCT
GTTTGCTGTGCTGATCAAACCCATTGTTGTCCTAATGGTTTCTCATGTGATGCAAGTAATGGTACCTGTG
TCAAGGAAGCCCCTGTATTCTCAGCCTTACACAAAGTCTTTCAGCCTCTGTATAATAGTAAAGTCAAAGA
AAATGTCTGCCCTGATGGTCAAAGTGAATGCTCTGATAT

From: NCBI est: Contig of GI:(282431968, 282442850, 282431967, 282461281, 282448361, 282448074, 282447774, 282436548, 282446597, 282447772, 282445371, 282428021, 282428020, 282452023, 282465071, 282448562, 282461280, 282436547, 282465070, 282446596, 282442849, 282448073)

Note: This cDNA contig encodes a little more than the last 7 modules, confirming the termination, and including the 3'ut. There are some differences with the genomic prediction which may be due to allelic differences.

TTGTCCTCAACCTAATGCTGTCTGTTGTGCTGATAAAGTCCACTGCTGTCCTTCAGGCTATTCTTGTAAT
ACAGCTGATGGTACTTGTACTAGAGGGGTCTCTGTTGTACCGATGATGAAGAAACTTACATCCTTGAGGT
CACTGTCTGATATAAAAAATGTGGTTTGTCCTGATGGGAGGCAGAAGTGTCCTGATGACAATACTTGCTG
TGAATTGGAATCTGGTAGCTATGGTTGCTGTCCTAGAGCAGATGCTGTTTGCTGTGCTGATAAAACTCAC
TGCTGTCCTAATGGCTACATGTGCGAGAATAATGGTACCTGCAAGAGAGACTCTAAAGTCTATGAAGCAC
TGGCAGTTGTGAGACTTGATGAAGTTAAGTGTCCTGATGGGGGAAGCTGTCCTGATAATAATACATGTTG
TCCAACTGGTGAAGATAAGTATGGGTGTTGTCCTCAACCTAATACTGTCTGTTGTGCTGATAAAGTCCAT
TGCTGCCCTAAAGACTTTTCTTGTAACACAGCTGATCATACTTGTACTAAGGGGGTCTCTGTTATACCAA
TGATGAAGAAGCTTCCAACGTTGGGCTCACCTACTAATTTTAAAGGGGTTGTATGCCCTAATGGAAAGGA
TGAGTGTCCTGATGGTAGCACTTGCTGCATTAGAGGTGATGCAAAAGATGGCTGCTGTCCTTTGGCTGGT
GCTGTTTGTTGTGCTGATCAAACCCATTGTTGTCCTAATGGTTTCTCATGTGATGCAAGTAATGGTACCT
GTGTCAAAGAAGCCTCTGTATTCTCAGCCTTACACAAAGTCTTTCAGCCTCTGTATAATAGTAAAGTCAA
AGAAAATGTCTGCCCTGATGGTCAAAGTGAATGCTCTGATATTAATACATGCTGTAAGACCGATACAGGA
AAGTATGGCTGCTGTGAGTTACCTCAAGCTACCTGCTGCTCTGATATGAAGCATTGCTGTCCAAGTGGTT
ACATTTGCAATTCTAATGGCACCTGTAATAGGCAGGATTCATTTATACCAATTGAAGCAGTTCTCATTAA
GAATAAGGTAAAGGATGTCACTTGTAGCGATGGCAGTACATCGTGTAAAAATGATCAGACTTGCTGTCTT

CTTAAAGATGGACGCTACGGTTGCTGTAACCTCACTGATGCCAATTGCTGTGGTGATGGGATACATTGCT
GTCCAAATGGTTACTTGTGTGACGTGGCACAAGGCACGTGTGTAAAAGAAGCATCAGTCATACCAATACT
GAAGAAAATGATGTCAGTGTCTACTCAAGTAAAGGGTGATATAAAATGTCCTGATGATACGGTTTGCTCT
GACAATAACACTTGCTGTAAAACGAAGTCTAATTCGTTTGGTTGTTGTCCTTCACCTAAAGCTACCTGTT
GTGCTGATGAGGTCCACTGCTGTCCTAGTGGCACAACTTGTGATCTTGAAGGTCATACATGCAGGGTCTC
AGAAGCAAAGCATCAACCATTACTTCGTGTTGGGAGTATGAAGAAACCGGTCGTCGTTAATGTTGTGTGC
CCTTCTGTTCCAGGCCACGAGGAGAAGGAGTGTTCTAGTAACAGCACATGCTGCTTGATGTCTGAAGGCA
ATTATGGTTGCTGCCCTTTTCCGAATGCTGTCTGCTGTGATGACAAGAAGCATTGCTGTCCTTTTGATTA
TGCATGTAACCCTGATGATGGAACATGTACAGACAAATCCAAAGGATTCCTTTACACCATCCCTCCTACA
ACGATGAGCTACAAGAGGCCTCTCCTGTAAGTATCATGACAATAGCTTGCTGTCATTATACGTTGCTATT
GCTGTATAAATTTGTTTTCTCCCCATATTGCTACATGCATTATACTCCTTATTAATAATTTATTGTGATT
CAGTCTCAATTCATTGCTAGTATAATTTGTTGCTTTTTTCTTAAGGATACAATTTTGTTGTAAACACTTC
AAAAAAAAAAAAAAAAAAAA

Genomic data:

Note: Genomic data (exons).
From NCBI wgs: ACUQ01000195.1 GI:296313059
Coding exon 1 **Exon type:** **snc**
Note: From the start codon
atggccgtgaaattcgttctcttgctgctgtttgcttcagtttactgtcgtccttctcttccacaaagca
acctttatcctcaactggatgtagttcactgccccaattctgaggacctctgcccggctaataatacgtg
ttgccgtcttaccccggagctctggggatgctgtccacacatagacgcttactgctgctctgataagcgc
cactgctgtcctcgtggccatccttgctccgcggactccttgaactgctcggactctactacaaccttcc
cttccgttttgatgagttttaaaaggcaaagaaacagaaataaacccttctttgaaagtaaaa

Coding exon 2 **Exon type:** **ncncncncncncncncncncncncnc**
Note: Showing to the stop codon. This exon continues through the 3'ut as confirmed by an EST contig.
tttcagatacattatcacaagttgccgttaatgtgaattgcactcctcctggttcaaaagagagctggca
gtgtcctgccgatcatacgtgctgctatatttcaaccactaggagatatggttgctgtccaatagtacct
gcaacttgctgccctgatggaagaggatgctgtccatcaggatacacttgtgacctaaccgataatacat
gcactccaccaaaagactcacttgcaacactggcaccgactctaattgacgtgagacctagacccaccaa
cgttactgatataatttgtcccaatgggataaatcaatgtcctgatggtgaaacctgctgtcgggtgagc
agtgatgatttcgggtgctgtcatttttccaatgctgtctgctgtgctgatatgaagcactgctgtccta
gcggatatatgtgtagtccgaatgacggtaactgtataaggtctcaagtggctatacttaagaagaaacc
tgcaattagcatatccttcactaacgtgactgaagtccattgtcctgatggtaaagtatgtctcgatggc
aatacttgctgtcagaccctctctggtagctatggttgctgtcctagggaagatgctgtttgttgtgctg
atagagttcactgctgtcctcatggcactgaatgcaaaagtgatggcacttgcactaggaccaatgaggt
aaagaagaccctgaaacttgattcagttaagtgtcctgatggtggagtttgtcttgatggtgaaacttgt
tgtcgagaaagtggttataaatatggttgttgttcttatcctgatgctacctgttgctctgatggaatcc
attgctgtccctcaggagagatatgtgacgtagctcgtgggacttgtttttatggaggctctgctaaagc
aatgatgaagaaactgtcattcttgaggtcaccaattaaagttaaaaatgtggtttgtcctgatggagga
agctgtcctgatgataatacatgttgtctaactggtgacaataagtatgggtgctgtcctcaacctaatg
ctaactgttgcgctgataaagtccattgctgtccttcaggctattcttgtaatgtagctgatggtacttg
tactagaggggtctctgttgtaccaatgatgaagaaattgtcatccttgaggtcaccatctgatgtaaaa
aatgtggtttgtcctggtgggaggcagaagtgtcctgatgacaatacttgctgtgaattggaatctggta
gctatggttgctgtcctagaccagatgctgtttgctgtgctgataaaactcactgctgtcctaatggcta
tacgtgccagaacgatggtacctgcaagaaagacacgaaagtctatgaagcactggcagttgtgaaactt
gattcagttaagtgtcctgatggtggagtttgtcttgatggtcaaacttgttgtcgagaaagtggttata
aatatggttgttgtccttatcttgatgctacctgttgctctgatggaatccattgctgtccctcaggaga
gatatgtgacgtagctcgtgggacttgtttttacggagactccgctaaagcaatgatcaagaaactgtca
ttcttgaagtcaccaactaaagttaaaaatgtggtttgtcctaatgggaggcaggagtgtcctgatgaca
atacttgttgtgaattgaaatctggtagctatggttgctgtcctagagcagatgctgtttgctgtgctga
taaaattcactgctgtcctaatggctacacgtgcgagaacgatggtacttgcaagagagactcgaaagtc
tatgaagctctggcagttttgaaactcaatacagttgactgtcctgatggggggaagctgtcctgataata
atacatgttgtctaactggtgtagataagtacgggtgctgtcctcaacctaatgctgtctgttgcgctga
taaagtccattgctgccctaaagacttttcttgtaatacagctgatcatacttgtactaaggggggtctct
gttataccaatgatgaagaagcttccaacgttgggctcacctactaaattaaaagggggttgtatgcccta
atggaaagaatgagtgtcctgatggtagcacttgctgcagtaaaggtgatgcaggatatggctgctgccc
tttggctaatgctgtttgctgtgctgatcaaacccattgttgtcctaatggtttctcatgtgatgcaagt

aatggtacctgtgtcaaggaagcccctgtattctcagccttacacaaagtctttcagcctctgtataata
gtaaagtcaaagaaaatgtctgccctgatggtcaaagtgaatgctctgatattaatacatgctgtaagac
cgatacaggaaagtatggctgctgtgagttacctcaagctacctgctgctctgatatgaagcattgctgt
ccaagtggttacatttgcaattctgatggctcctgtaataggcaggattcctttatacaaattgaagcag
ttctcattaagaataaggtaaaggatgtcacttgtagcgatggcaatacatcgtgtaaaaatgatcagac
ttgctgtcttcttaaagatggacgctatggttgctgtaacctcactgatgctaattgctgtggcgatggg
atacattgctgtccaaatggttactcgtgtgacgtggcaaaaggcacgtgtgtaaaagaagcatctgtca
taccaatactaaagaaaatgatgtcagtgtctactcaagtaaaggatgatataaaatgtcctgatgatac
ggtttgctctgacaataacacttgctgtaaagcaaagtctaattcgtttggttgttgtccttcacctaaa
gctacctgttgtgctgatgagatccactgctgtcctagtggcacaacttgcgatcttgaaggccatacct
gcagggtctcagaagcaaagcatcaaccattacttcatgttgggagtatgaagaaaccagtcgtcgttaa
tgttgtgtgtccttctgttccaggccacgaggagaaggagtgctctagtaacagcacgtgctgcttgatg
tctgaaggcaattatggttgctgccctttttccgaatgctgtctgctgtgatgacaagaagcattgctgtc
cttttgattatgcatgtaaccctgatgatggaacatgtacagacaaatccaaaggattcctttacaccat
ccctcctacaaggatgagctacaagaggcctctcctgtaa

Genomic sequence note: A genomic sequence is not presented (see note 2 in header).

00041 **A. queenslandica progranulin S1** Short name: **A_queS1** Date entered: 2014-03

Species: ***Amphimedon queenslandica (sponge)***
Taxonomy (via NCBI): *Eukaryota; Metazoa; Porifera; Demospongiae; Ceractinomorpha; Haplosclerida; Niphatidae; Amphimedon.*
General Comment: Four apparent progranulin genes are close together in the genome. This is the second, which encodes a progranulin with 2 modules in a single exon. Its coding starts about 810 bases after the stop codon, and 600 bases after the cDNA-supported 3'ut end of the preceding large progranulin gene (A_queL). The translation begins with a signal-peptide-like sequence, but it does not score well as a signal sequence. There is a possible splice from just before the A_queL stop codon into the beginning of A_queS1 (see ensembl Aqu1.229537), but so far all cDNA data support the termination of the large form and its 3'ut in A_queL exon 2.

Protein Sequence

MKVTILSFFFAAICLTNARMVSYVKDIPCDNGSCPLGDTCCSGDAKPCCPLPNAVCCGDSKCCPSGYTCS
PPHCVREEDNHITLMLSSSDIPQKSNNVTIIPCNTGYCPDGDTCCPDGCCPDYNGVCCYNGFCCPTGYVC
SRVDCVRSAKEGLNVVPQRKILLPHTKKNKD*

Transcript:

Note: The transcript sequence is predicted from ACUQ01000195.1. There is as yet no cDNA data for this gene.

Predicted sequence:

Note: Predicted from ACUQ01000195.1 from translation start codon to stop codon. It is the same as the single coding exon sequence.
Derivation: NCBI wgs ACUQ01000195.1 GI:296313059
ATGAAGGTCACTATTCTTTCTTTTTTCTTTGCTGCAATTTGTCTGACCAATGCAAGAATGGTCAGTTATG
TAAAAGATATACCTTGTGATAATGGTTCATGTCCTCTTGGTGACACATGCTGCTCTGGTGATGCAAAGCC
ATGCTGTCCTTTACCTAATGCTGTCTGCTGTGGTGATAGCAAATGCTGTCCCTCTGGCTACACTTGCAGT
CCACCTCACTGTGTCAGGGAAGAAGATAATCACATTACTCTAATGCTGTCTTCATCAGACATTCCACAAA
AGAGCAACAATGTCACAATCATTCCTTGCAACACTGGATACTGCCCTGATGGTGATACATGCTGCCCTGA
TGGTTGTTGTCCTGACTATAATGGCGTCTGCTGCTATAACGGATTTTGCTGTCCTACTGGTTACGTCTGC
AGCAGGGTTGATTGTGTGCGAAGTGCAAAGGAAGGTTTAAATGTTGTGCCTCAAAGAAAAATCCTTCTCC
CACATACCAAGAAGAATAAAGATTAA

cDNA data:

cDNA sequence data: No cDNA data (or empty cDNA entry).

Genomic data:

Note: Genomic data (exons).
From NCBI wgs: ACUQ01000195.1 GI:296313059
Coding exon 1 **Exon type:** sncnc

Note: From start codon to stop codon.
atgaaggtcactattctttcttttttctttgctgcaatttgtctgaccaatgcaagaatggtcagttatg
taaaagatataccttgtgataatggttcatgtcctcttggtgacacatgctgctctggtgatgcaaagcc
atgctgtcctttacctaatgctgtctgctgtggtgatagcaaatgctgtccctctggctacacttgcagt
ccacctcactgtgtcagggaagaagataatcacattactctaatgctgtcttcatcagacattccacaaa
agagcaacaatgtcacaatcattccttgcaacactggatactgccctgatggtgatacatgctgccctga
tggttgttgtcctgactataatggcgtctgctgctataacggatttgctgtcctactggttacgtctgc
agcagggttgattgtgtgcgaagtgcaaaggaaggtttaaatgttgtgcctcaaagaaaaatccttctcc
cacataccaagaagaataaagattaa

Genomic sequence note: A genomic sequence is not presented (see note 2 in header).

00042 **A. queenslandica progranulin S2** Short name: **A_queS2** Date entered: 2014-03

Species: ***Amphimedon queenslandica (sponge)***
Taxonomy (via NCBI): *Eukaryota; Metazoa; Porifera; Demospongiae; Ceractinomorpha; Haplosclerida; Niphatidae; Amphimedon.*
General Comment: Four apparent progranulin genes are close together in the genome. This is the third, which encodes a progranulin with 2 modules in a single exon. Its coding starts about 886 bases after the stop codon of the preceding small progranulin gene (A_queS1).

Protein Sequence
MKVAILSFVFAAICLTNARMVSYVKDIPCDGGTCPDGDTCCSGDAKPCCPTPNGVCCGGGKCCRSGYTCR
PPYCIKDDAHYESVILSKSRTVEKMKHYQKSSLTGIPCNGGECPDGYNTCCPNEPKPCCSYSNGVCCGDG
TCCPSGYTCSGDSCLREDAHEKGVTRMLSTSGLPRKGNNVTDIPCNGDSCPRKKDKNY*

Transcript:
Note: The transcript sequence is predicted from ACUQ01000195.1. There is as yet no cDNA data for this gene. It begins with a signal peptide which is skipped by ensembl Aqu1.229538, which begins 20 codons downstream.

Predicted sequence:
Note: Predicted from ACUQ01000195.1 from translation start codon to stop codon. It is the same as the single coding exon sequence.
Derivation: NCBI wgs ACUQ01000195.1 GI:296313059
ATGAAAGTTGCTATTCTTTCTTTTGTCTTTGCTGCAATTTGTTTGACCAATGCAAGAATGGTCAGTTATG
TAAAAGATATACCTTGCGATGGAGGTACATGCCCTGATGGGGACACGTGCTGCTCTGGTGATGCAAAGCC
ATGCTGTCCTACACCTAATGGTGTTTGCTGTGGTGGCGGCAAATGCTGTCGCTCTGGCTACACTTGCCGT
CCACCTTACTGTATCAAGGACGACGCTCATTATGAATCAGTGATTCTATCCAAGTCTCGTACAGTTGAAA
AGATGAAGCATTACCAAAAATCTTCCCTGACTGGCATTCCATGTAATGGAGGAGAATGCCCTGATGGTTA
CAACACATGCTGTCCCAATGAACCTAAACCATGTTGCTCATATTCTAATGGTGTCTGCTGTGGTGATGGT
ACTTGCTGCCCCTCTGGGTACACCTGCAGTGGAGACTCCTGTCTCAGGGAAGATGCACATGAGAAAGGTG
TTACTCGAATGCTATCTACATCAGGCCTTCCAAGAAAGGGCAACAATGTCACAGACATTCCTTGCAATGG
AGATTCCTGTCCACGCAAGAAGGATAAAAATTACTGA

cDNA data:
cDNA sequence data: No cDNA data (or empty cDNA entry).

Genomic data:
Note: Genomic data (exons).
From NCBI wgs: ACUQ01000195.1 GI:296313059
Coding exon 1 **Exon type:** sncnc
atgaaagttgctattctttcttttgtctttgctgcaatttgtttgaccaatgcaagaatggtcagttatg
taaaagatataccttgcgatggaggtacatgccctgatggggacacgtgctgctctggtgatgcaaagcc
atgctgtcctacacctaatggtgtttgctgtggtggcggcaaatgctgtcgctctggctacacttgccgt
ccaccttactgtatcaaggacgacgctcattatgaatcagtgattctatccaagtctcgtacagttgaaa
agatgaagcattaccaaaaatcttccctgactggcattccatgtaatggaggagaatgccctgatggtta
caacacatgctgtcccaatgaacctaaaccatgttgctcatattctaatggtgtctgctgtggtgatggt
acttgctgcccctctgggtacacctgcagtggagactcctgtctcagggaagatgcacatgagaaaggtg

```
ttactcgaatgctatctacatcaggccttccaagaaagggcaacaatgtcacagacattccttgcaatgg
agattcctgtccacgcaagaaggataaaaattactga
```

Genomic sequence note: A genomic sequence is not presented (see note 2 in header).

00043 **A. queenslandica progranulin S3** Short name: **A_queS3** Date entered: 2014-03

Species: ***Amphimedon queenslandica (sponge)***
Taxonomy (via NCBI): *Eukaryota; Metazoa; Porifera; Demospongiae; Ceractinomorpha; Haplosclerida; Niphatidae; Amphimedon.*
General Comment: Four apparent progranulin genes are close together in the genome. This is the fourth, which encodes a progranulin with 3 modules in a single exon. Its coding starts about 620 bases after the stop codon of the preceding small progranulin gene (A_queS2).

Protein Sequence

```
MKVAILSLVLAAICLTNARMVSYVKDIPCNGGTCPDGDTCCSGDSLPCCPTSNGVCCGDNHCCPSGTTCS
PPFCIGKDAELSSFVLPKIHGAEKMKLNSQPTLTDIPCEGGYCPDGDTCCSNEPKPCCPTSNGVCCGDGT
CCPSGYTCTPTSCIREDAHKKGVTLMLSSSGLPRKDNNVTDIPCGGGKSCRDGETCCPTGCCPESNGVCC
SNDACCPSGYSCDSDGVHCDPDAKDGFAVLQTKIPLGRTKKTKN*
```

Transcript:

Note: The transcript sequence is predicted from ACUQ01000195.1. There is as yet no cDNA data for this gene. It begins with a signal peptide which is skipped by ensembl Aqu1.229539, which begins 20 codons downstream.

Predicted sequence:

Note: Predicted from ACUQ01000195.1 from translation start codon to stop codon. It is the same as the single coding exon sequence.
Derivation: NCBI wgs ACUQ01000195.1 GI:296313059

```
ATGAAGGTTGCTATTCTTTCTCTTGTCTTAGCTGCAATTTGTTTGACCAATGCAAGAATGGTCAGTTATG
TAAAAGATATACCTTGCAATGGAGGCACATGTCCTGATGGTGACACATGCTGCTCTGGTGATTCATTGCC
CTGCTGTCCTACATCTAACGGTGTCTGCTGTGGTGATAACCATTGCTGTCCTTCTGGAACCACTTGCAGT
CCACCTTTCTGTATTGGGAAAGATGCCGAGTTGTCATCTTTTGTTCTACCAAAAATTCATGGTGCTGAAA
AGATGAAACTAAACTCACAACCCACACTGACAGATATACCTTGCGAAGGAGGTTACTGTCCTGATGGTGA
CACATGCTGCTCTAATGAACCAAAGCCATGCTGTCCTACATCTAATGGTGTCTGCTGTGGTGATGGTACT
TGCTGCCCCTCTGGGTACACCTGCACTCCAACCTCCTGTATCAGGGAAGATGCACATAAGAAAGGTGTCA
CTCTAATGCTATCTTCATCAGGCCTTCCAAGAAAGGACAACAATGTCACAGACATTCCTTGTGGCGGTGG
TAAGTCTTGTCGTGATGGTGAAACATGCTGTCCTACTGGTTGCTGTCCTGAATCTAACGGTGTCTGCTGT
AGTAATGATGCATGCTGTCCTTCTGGTTACTCCTGTGACAGTGATGGGGTCCATTGTGATCCAGATGCAA
AGGATGGTTTTGCAGTACTTCAAACAAAAATCCCTCTTGGACGTACTAAGAAGACTAAAAATTAA
```

cDNA data:

cDNA sequence data: No cDNA data (or empty cDNA entry).

Genomic data:

Note: Genomic data (exons).
From NCBI wgs: ACUQ01000195.1 GI:296313059
Coding exon 1 **Exon type:** sncncnc
Note: From the start codon to the stop codon.

```
atgaaggttgctattctttctcttgtcttagctgcaatttgtttgaccaatgcaagaatggtcagttatg
taaaagatataccttgcaatggaggcacatgtcctgatggtgacacatgctgctctggtgattcattgcc
ctgctgtcctacatctaacggtgtctgctgtggtgataaccattgctgtccttctggaaccacttgcagt
ccacctttctgtattgggaaagatgccgagttgtcatcttttgttctaccaaaaattcatggtgctgaaa
agatgaaactaaactcacaacccacactgacagatataccttgcgaaggaggttactgtcctgatggtga
cacatgctgctctaatgaaccaaagccatgctgtcctacatctaatggtgtctgctgtggtgatggtact
tgctgcccctctgggtacacctgcactccaacctcctgtatcagggaagatgcacataagaaaggtgtca
ctctaatgctatcttcatcaggccttccaagaaaggacaacaatgtcacagacattccttgtggcggtgg
taagtcttgtcgtgatggtgaaacatgctgtcctactggttgctgtcctgaatctaacggtgtctgctgt
agtaatgatgcatgctgtccttctggttactcctgtgacagtgatggggtccattgtgatccagatgcaa
```

aggatggttttgcagtacttcaaacaaaaatccctcttggacgtactaagaagactaaaaattaa

Genomic sequence note: A genomic sequence is not presented (see note 2 in header).

00002 **Bovine progranulin** Short name: **B_tau or bovine** Date entered: 2014-03

Species: ***Bos taurus (cattle)***
Taxonomy (via NCBI): *Eukaryota; Metazoa; Chordata; Craniata; Vertebrata; Euteleostomi; Mammalia; Eutheria; Laurasiatheria; Cetartiodactyla; Ruminantia; Pecora; Bovidae; Bovinae; Bos.*
General Comment: Well documented transcript and genomic sequences

Protein Sequence

```
MWTLVSWVALVTGLVVGTQCPDGQLCPVACCLDPNGATYSCCNPVQDQRPSVLSQRLGRPCQADGHCAPG
YSCILTVSGTSSCCPFPEAVSCGDGRHCCPRGFHCNADGRSCFQRSDTKPLDAVQCPDKQFQCPNSSTCC
TMPDGSWGCCPMPQASCCEDKIHCCPHGTSCDLARGRCLSATGTHPLAKKMPAHKTKSSVILCPDGQSQC
PDGSTCCKLPTGKYGCCPMPNAICCSDHLHCCPQNTVCDLTQSKCLSKENATDLLTKLPAHTVQDVKCDM
EVSCPDDYTCCRLQSGAWGCCPFVQAVCCEDHVHCCPSGFRCDTEKGVCEQGTRQVPWMKKAPAHLSLLD
LGAVEGDVPCDNVTSCPSSTTCCRLKSGEWACCPAPEAVCCSDHQHCCPKGYTCVARRHCKRGKQVVTGL
DKVPAHRASPSHPRDMGCDQHTSCPVGQTCCPSLRGAWACCKLPHAVCCEDRQHCCPAGYTCNVKARSCE
KEVDPVHPAVRLASGPPVGMGNVECGARHFCHDNQTCCPDSQGGWACCPYRKGTCCGDKHHCCPSGFRCA
ARGTKCLRREALRWDAPWRDPTPRQLL*
```

Transcript:

The cDNA data define the transcript well (below), so no predicted sequence is required.

cDNA data:

From: NCBI: BC105334
Note: cDNA sequence data:

```
GAGAGATCGCGGACCCCGACGCAGGTGGACCATGTGGACTTTGGTGAGCTGGGTGGCCTTAGTGACAGGG
CTGGTGGTTGGAACACAGTGCCCAGACGGTCAGCTCTGCCCTGTGGCCTGCTGCCTGGACCCGAATGGAG
CCACCTACAGCTGCTGCAATCCCGTTCAGGACCAGCGGCCGTCTGTGCTGAGCCAGCGTCTGGGCCGACC
CTGCCAGGCCGATGGCCACTGCGCTCCTGGCTACTCCTGCATCCTCACTGTCTCAGGGACCTCCAGCTGC
TGCCCGTTCCCAGAGGCTGTGTCATGTGGGGATGGCCGCCACTGTTGCCCCCGGGGCTTCCACTGCAACG
CTGACGGGCGGTCCTGCTTCCAAAGATCAGATACCAAGCCCCTGGATGCCGTCCAGTGCCCCGACAAACA
GTTCCAGTGCCCCAACTCCTCCACGTGCTGCACCATGCCTGATGGCTCCTGGGGATGCTGCCCCATGCCC
CAGGCTTCTTGCTGTGAAGACAAGATACACTGCTGCCCCCATGGCACGTCCTGTGACCTGGCTCGTGGCC
GCTGTCTCTCGGCCACAGGCACCCACCCCCTGGCTAAGAAGATGCCTGCACACAAGACTAAAAGTTCAGT
GATCTTATGCCCAGATGGACAGTCCCAGTGCCCCGATGGTTCTACCTGCTGCAAGCTGCCCACTGGAAAG
TATGGCTGCTGCCCGATGCCCAATGCCATTTGCTGCTCCGACCACCTGCACTGCTGCCCCCAGAACACTG
TGTGTGACCTGACCCAGAGTAAGTGCCTCTCCAAGGAGAACGCTACGGACCTCCTCACCAAGCTGCCCGC
ACACACAGTGCAGGATGTCAAGTGCGACATGGAGGTGAGCTGCCCAGACGACTACACCTGCTGCCGCCTA
CAGTCCGGGGCCTGGGGCTGCTGCCCTTTTGTGCAGGCCGTGTGCTGTGAGGACCATGTGCACTGCTGCC
CGTCCGGGTTTAGGTGTGACACAGAGAAGGGTGTGTGTGAGCAGGGGACCCGCCAGGTGCCGTGGATGAA
GAAAGCCCCAGCCCACCTCAGCCTGCTGGACCTCGGAGCCGTGGAGGGGGACGTCCCCTGTGATAACGTC
ACCAGCTGTCCTTCTTCCACTACCTGCTGTCGACTCAAGTCTGGGGAGTGGGCCTGCTGTCCTGCTCCAG
AGGCTGTCTGCTGCTCGGACCACCAGCACTGCTGCCCGAAGGGCTACACGTGTGTGGCCAGAAGGCACTG
TAAGAGGGGGAAACAGGTGGTGACCGGACTGGACAAAGTGCCTGCCCACAGGGCCTCCCCGTCCCACCCC
AGAGACATGGGCTGTGACCAGCACACCAGCTGCCCGGTGGGGCAGACCTGCTGCCCGAGCCTGAGGGGGG
CCTGGGCCTGCTGCAAGTTGCCGCACGCCGTGTGCTGTGAGGACCGCCAGCACTGCTGCCCGGCTGGCTA
CACCTGCAACGTGAAGGCCCGATCCTGTGAGAAGGAGGTGGACCCTGTGCACCCTGCCGTCCGCCTGGCC
AGCGGCCCTCCTGTGGGCATGGGGAACGTGGAGTGTGGGGCGAGGCACTTCTGCCACGATAACCAGACCT
GCTGCCCAGACAGCCAGGGGGGCTGGGCCTGCTGCCCCTACCGCAAGGGCACCTGTTGTGGGGACAAGCA
TCACTGCTGTCCCAGTGGCTTCCGCTGTGCAGCCAGGGGGACCAAGTGCTTACGTCGGGAGGCCCTGCGC
TGGGACGCTCCTTGGAGGGACCCGACACCGAGACAGCTGCTGTGAGGAGGGCCTGAGGACTGAAGACACT
TGGCAGCCCTCAGGACCCTGCTCAGAGGGTCCCCACTGCTCAGGCCTCCCCAGCACCTCTCCCCCACCAC
ATTCTCCCAGACCCCCTTCTGAGTCCCCCATCACCCTGGGAGGTGGGGCCTCAATCTAAGGCCTCCCTTA
TGCCAAGGTGGTGGTGGGGGGGGGGCTGTGGCAAAAGCCACGTTTCAAGCTGCCATCCCCTCCCCCAATT
TCTGTCAACTCTGTGGCCAGGTGCTCTTCCTATCCACGGGTGTGCGTGCGTGTGTATGTGTGTGTGTGTG
CGTGCTGCAATAAAGTTTGTACACTTTCAAAAAAAAAAAAAAAAAAAAAAAAAAAAAAAAAAAAAAAAAA
```

Genomic data:

Note: Genomic data (exons).

From NCBI: NC_007317

Coding exon 1 **Exon type:** sn

Note: from start codon

ATGTGGACTTTGGTGAGCTGGGTGGCCTTAGTGACAGGGCTGGTGGTTGGAACACAGTGCCCAGACGGTC
AGCTCTGCCCTGTGGCCTGCTGCCTGGACCCGAATGGAGCCACCTACAGCTGCTGCAATCCCGTTCAG

Coding exon 2 **Exon type:** n*

Note: * Encodes the N-half of a 10 cys module

GACCAGCGGCCGTCTGTGCTGAGCCAGCGTCTGGGCCGACCCTGCCAGGCCGATGGCCACTGCGCTCCTG
GCTACTCCTGCATCCTCACTGTCTCAGGGACCTCCAGCTGCTGCCCGTTCCCAGAG

Coding exon 3 **Exon type:** c*

Note: * Encodes the C-half of a 10 cys module

GCTGTGTCATGTGGGGATGGCCGCCACTGTTGCCCCCGGGGCTTCCACTGCAACGCTGACGGGCGGTCCT
GCTTCCAAAGATCAG

Coding exon 4 **Exon type:** n

ATACCAAGCCCCTGGATGCCGTCCAGTGCCCCGACAAACAGTTCCAGTGCCCCAACTCCTCCACGTGCTG
CACCATGCCTGATGGCTCCTGGGGATGCTGCCCCATGCCCCAG

Coding exon 5 **Exon type:** c

GCTTCTTGCTGTGAAGACAAGATACACTGCTGCCCCCATGGCACGTCCTGTGACCTGGCTCGTGGCCGCT
GTCTCTCGGCCACAGGCACCCACCCCCTGGCTAAGAAGATGCCTGCACACAAGACTAAAAGTTCAG

Coding exon 6 **Exon type:** n

TCTTTCCCCTTCCAGTGATCTTATGCCCAGATGGACAGTCCCAGTGCCCCGATGGTTCTACCTGCTGCAA
GCTGCCCACTGGAAAGTATGGCTGCTGCCCGATGCCCAAT

Coding exon 7 **Exon type:** c

GCCATTTGCTGCTCCGACCACCTGCACTGCTGCCCCCAGAACACTGTGTGTGACCTGACCCAGAGTAAGT
GCCTCTCCAAGGAGAACGCTACGGACCTCCTCACCAAGCTGCCCGCACACACAG

Coding exon 8 **Exon type:** n

TGCAGGATGTCAAGTGCGACATGGAGGTGAGCTGCCCAGACGACTACACCTGCTGCCGCCTACAGTCCGG
GGCCTGGGGCTGCTGCCCTTTTGTGCAG

Coding exon 9 **Exon type:** cn

GCCGTGTGCTGTGAGGACCATGTGCACTGCTGCCCGTCCGGGTTTAGGTGTGACACAGAGAAGGGTGTGT
GTGAGCAGGGGACCCGCCAGGTGCCGTGGATGAAGAAAGCCCCAGCCCACCTCAGCCTGCTGGACCTCGG
AGCCGTGGAGGGGGACGTCCCCTGTGATAACGTCACCAGCTGTCCTTCTTCCACTACCTGCTGTCGACTC
AAGTCTGGGGAGTGGGCCTGCTGTCCTGCTCCAGAG

Coding exon 10 **Exon type:** cn

GCTGTCTGCTGCTCGGACCACCAGCACTGCTGCCCGAAGGGCTACACGTGTGTGGCCAGAAGGCACTGTA
AGAGGGGGAAACAGGTGGTGACCGGACTGGACAAAGTGCCTGCCCACAGGGCCTCCCCGTCCCACCCCAG
AGACATGGGCTGTGACCAGCACACCAGCTGCCCGGTGGGGCAGACCTGCTGCCCGAGCCTGAGGGGGGCC
TGGGCCTGCTGCAAGTTGCCGCAC

Coding exon 11 **Exon type:** cn

GCCGTGTGCTGTGAGGACCGCCAGCACTGCTGCCCGGCTGGCTACACCTGCAACGTGAAGGCCCGATCCT
GTGAGAAGGAGGTGGACCCTGTGCACCCTGCCGTCCGCCTGGCCAGCGGCCCTCCTGTGGGCATGGGGAA
CGTGGAGTGTGGGGCGAGGCACTTCTGCCACGATAACCAGACCTGCTGCCCAGACAGCCAGGGGGGCTGG
GCCTGCTGCCCCTACCGCAAG

Coding exon 12 **Exon type:** c

Note: to stop codon

GGCACCTGTTGTGGGGACAAGCATCACTGCTGTCCCAGTGGCTTCCGCTGTGCAGCCAGGGGGACCAAGT
GCTTACGTCGGGAGGCCCTGCGCTGGGACGCTCCTTGGAGGGACCCGACACCGAGACAGCTGCTGTGA

Genomic sequence note: A genomic sequence is not presented (see note 2 in header).

00004 **Branchiostoma floridae progranulin (I)** Short name: **B_flo or Brafl** Date entered: 2014-03

Species: ***Branchiostoma floridae (Florida lancelet)***
Taxonomy (via NCBI): *Eukaryota; Metazoa; Chordata; Cephalochordata; Branchiostomidae; Branchiostoma.*
General Comment: There are no cDNA data for this progranulin gene. In the middle of the gene there is a long repeat sequence (about 27 repeats) encoding nearly identical granulin modules except that the data indicate frame-shifts within the repeat. It is likely assembly errors were introduced here, but also splice donors and acceptors can be found within the sequence, so it remains to be clarified how the repeat sequence is used in the transcripts. The prediction here for the transcript before and after the repetitive sequence is from the now "obsolete" wgs sequence NW_002235344 and the current ABEP02018454. A second, distinct transcript with a high level of identity was predicted from the now "obsolete" NW_002234951. These differences are most likely allelic. In the original analysis we kept track of these two sequences by labelling them A and B, but when this might be confused with the distinct A and B forms found in fish, we used I and II respectively. The latest sequence for this gene at the JGI B. floridae genome project site is estExt_fgenesh2_pg.C_160005. There is only one, and it appears to be a hybrid between our two predictions. For our analysis these differences are not important, except for coding exon 7, which the jgi prediction omits. Except for the middle repetitive sequence, the sequence provided here is from our prediction I. For the splicing within the repeat, we include here the exons predicted in estExt_fgenesh2_pg.C_160005. It encodes repeat modules typical of those encoded by the sequences we used in our analysis.

Protein Sequence

```
MRLLIVLLCSLALCSAVPFPKGAHQCPDVCQPEETCCKSNSTLTHGCCPISQATCCSDGLHCCPSGFKCD
LSQNACLQGDLTLNWSPKMEVKFRATITCPGGTHDCPENTTCCLTAKGDYGCCPLAKAACCKDHTHCCPS
GFKCDESAGKCRQGNRTLPFLEKFAARPRFVANQEQVCPGGKSTCPTTSTCCMLKNGKYGCCPMEKAVCC
EDKVHCCPSGFTCAATKCKKGEYSIPLFSKTEAKIRQMHPAKDAKVVTCPDGLSACKDNTTCCKDDTGEY
GCCPIPQAECCNDQIHCCPAGFTCQTEKGKCSKGGVVVPLLTKTRADVLSEEVESVHCDDGSACPNHHTC
CKTQTGKYGCCPLPKAVCCQDEIHCCPDGYTCDTAKSRCIKGDMTLPWLEKRPSKSSRKDPGTKPPPGAV
VCPDHVSMCPDNNTCCLLQDGSYGCCPEPHAVCCSDRKHCCPQGYTCDTSQGKCLQGYRTIPWIENTVAP
PNKVVTPYKVEPPVNPIDCDEEGKVKCPDKNTCCKKTTGEWGCCPLEKALCCEDKVHCCPHGTVCNKTSG
ECHYPDGTSQAWVQKTPPLLDVRDDVKDVPCPDGKSACEEDYTCCKNSTGYGCCPFSRAVCCNDTVHCCP
QHYTCNSGAPGTCIHNFLPTVPMAKKTTPKMVQLHNEEIETDNEILNNVRCDTFHECPANNTCCKLSSGQ
WGCCPLPSAVCCDDHVHCCPEDTTCSVSTGKCHKGDASITWFEKLPAKTVQNDPNIMCDATHECPSKHTC
CKLASGQWGCCPLPSAVCCNDHVHCCPEGTTCKVSTGKCEKGDITLAWFEKTSAITVEDAPNVMCDATHE
CKAKETCCKLKSGQWGCCPLPKAVCCDDKVHCCPEGTTCSVSTGKCNKGDVSLAWFEKSPAKNVQNDQNI
MCDATHECPSKHTCCKLASGQWGCCPLPSAVCCNDHVHCCPEGTTCSVSTGKCEKGEITLAWFEKTSAIT
VEDAPNVMCDATHECKAKETCCKLASGQWGCCPLPKAVCCDDKVHCCPEGTTCSVSTGKCNKGDISFPWF
EKVPANNVQNVQNMKCDATHECPSKHTCCKLASGQWGCCPLPSAVCCNDHVHCCPEDTTCEVSSGKCKRK
DISFPWFEKVPAITTAQEVSNVKCDATHECPSKHTCLLSAVTTTSTAVPKILHVKCLLESEKGENSLPWF
EKTSAITVEDAPNVMCDATHECKAKETCCKLASGQWGCCPLPKAVCCDDHVHCCPEGTTYTTCEVSSGKC
KRKDSSIPWFEKVPAITTEEVSNVKCDATHECPSKHTCCKLASGQWGCCPLPSAVCCNDHVHCCPEDTTC
SVSTGKCEKGEISLPWFEKTSAITVEDAPNVMCDATHECKARETCCKLESGQWGCCPLPKAVCCDDKVHC
CPEGTTCSVSTGKCHKGDVSITWFEKVPAITAEEEVSNVKCDATHECPSKHTCCKLASGQWGCCPLPSAV
CCNDHVHCCPEDTTCEVSSGKCKRKDASIPWFDKVPAITTEEVSNVKCDASHECPSKHTCCKLASGQWGC
CPLPSAVCCNDHVHCCPEDTTCEAVCCDDKVHCCPEGTTCSVSTGKCHKGDVSIPWFEKVPAITAEEEVS
NVKCDATHECPSKHTCCKLASGQWGCCPLPSAVCCNDHVHCCPEDTTCEVSSGKCKRKDISFPWFEKKAA
INVEESPNIKCDSTHECKATDTCCKLKSGQWGCCPLPKAVCCDDKVHCCPEGTTCSVSTGKCHKGDVSIP
WFEKVPAITADKEVSNVKCDATHECPSKHTCCKLASGQWGCCPLPSAVCCNDHVHCCPEDTTFEDAPNVM
CDATHECKAKETCCKLASGQWGCCPLPSAVCCNDHVHCCPEGTTCSVSTGKCKHGDISLPWFEKKAALTV
DVEKTPCPGGKQECATNYTCCQRGATPYTWGCCDFPNAVCCPDRKTCCPKGYHCDPSRATCFKSESDDIV
VAKQLAIPDTEQAEVGNVICPGGEYQCPEKTTCCVSPSGTFGCCPVPEAVCCVDGVHCCPKDHTCDPSSQ
TCKSASGQLPWQDKLLAKQVQVKDIKCGSSTSTCGDTETCCKMATGQWGCCPMPKAVCCEDHLHCCPRGY
KCDMPTSTCIRGNDILPMAKKRPADIFNKS*
```

Transcript:
Note: No cDNA data available.

Predicted sequence:

Note: The prediction here is from the exons presented and explained in this entry. The parts predicted from the sequence in the current ABEP02018454 (originally from the now "obsolete" wgs sequence NW_002235344) were based upon manual splice prediction assisted by programs Augustus and FGENESH. The splicing within the repeat is from the JGI estExt_fgenesh2_pg.C_160005 entry and remains to be clarified by additional data.

Derivation: As described in this entry.

atgaggttactgatagtgctcctgtgcagcctggccctctgcagtgctgtgccattccccaaggggcgc
accaatgccctgatgtctgccaacctgaagaaacctgctgcaagtccaacagtacactgacacatggctg
ctgtcctatttctcaggctacctgttgcagtgacggcctccactgttgccccagcgggttcaaatgtgac
ctttcccagaatgcctgcctgcagggagacctgaccttgaattggtcccccaaaatggaggtcaaattcc
gcgccacgatcacatgtccgggcggaactcatgactgtccggagaataccacttgttgtttgaccgccaa
gggcgactatgggtgctgccctcttgcgaaggctgcctgctgtaaggatcacacccactgctgcccctct
gggttcaaatgcgacgagtcggcgggaaagtgtcgccaaggcaacaggacactccccttcctggagaagt
ttgcagccagacccaggtttgttgcgaaccaagaacaggtgtgccctggcgggaagtccacctgtcccac
caccagcacctgctgtatgctgaagaatgggaagtatggctgctgtcccatggaaaaggctgtgtgttgt
gaagacaaggtccactgctgtcccagcggcttcacctgtgcagctaccaagtgtaagaagggagagtaca
gcattcctctgttcagcaaaacagaagctaagatcaggcaaatgcacccagcgaaagacgccaaagtagt
gacgtgccccgacggtctgtctgcctgtaaggacaacaccacctgctgcaaggatgacactggagagtac
ggctgctgtccaataccacaggctgagtgctgtaatgatcagatccactgctgtcctgctggcttcacct
gccagacagagaagggaagtgttccaagggggcgtggtcgtccctctcctgactaaaacacgggctga
tgttttgtcagaggaggttgagtccgtccattgcgatgatggaagtgcctgtccaaaccatcacacctgc
tgcaagacgcagactgggaaatacggctgctgtccattgccaaaggccgtgtgctgtcaggatgagatcc
actgctgtcctgacggctacacctgtgacactgccaagagcaggtgtatcaagggcgacatgaccctccc
ctggctggagaaacgaccttcaaagtcctccaggaaggaccctggaaccaagcccctcccggtgcagtg
gtctgtcccgaccacgtgtcaatgtgtccagacaacaacacctgctgtctgctgcaggatgggagctatg
gctgctgccccgagccacatgccgtgtgctgcagcgaccgtaagcactgctgcccacaaggctacacctg
cgacacctcacagggaaagtgcctgcagggctacagaaccatcccgtggatagagaacacggtcgcgccc
cccaacaaggtcgtgactccctacaaggtcgagcccccagtcaaccccatcgactgtgatgaggaaggca
aggtcaagtgcccagataagaacacctgctgcaagaagacgaccggagaatgggggtgctgtcctcttga
gaaggccctgtgctgtgaggacaaggtccactgctgtccccacggtaccgtgtgtaacaaaacctccggg
gagtgtcactatcctgatggcacctcccaggcctgggtgcagaagacaccacccctcctggacgtcaggg
atgatgtgaaagatgtgccgtgtcccgatggaaagtccgcttgtgaggaagactacacctgctgtaagaa
cagcaccggctatggctgctgtcccttctccagggctgtctgctgtaacgacacggtccactgctgtccc
cagcactacacatgcaactctggggctccaggaacctgtatccacaacttcctgcccactgtcccaatgg
ccaagaagactactccaaagatggtacaactccacaacgaagagattgaaactgacaatgagatactgaa
caatgtaaggtgcgacacattccatgagtgcccagctaacaatacgtgctgtaagctttcctctggacag
tggggctgctgtcccttgccttcggctgtctgctgtgacgaccatgtccactgctgtcctgaagatacaa
catgcagtgtgtccactggaaaatgtcacaaaggtgatgccagcattacttggtttgagaagttgcctgc
taagactgttcagaatgacccaaacatcatgtgtgatgccacccacgaatgcccttcaaaacacacatgc
tgtaagctggcctctggacagtggggctgctgtcccttacccagtgctgtctgctgtaacgaccatgttc
actgctgtcctgaaggaacaacatgtaaagtctcaacaggaaagtgtgaaaagggagacatcaccttggc
ttggtttgagaaaacaagtgccataacagttgaagatgcaccaaatgtcatgtgtgatgccacccatgaa
tgcaaagctaaagaaacgtgctgtaaactaaagtctggacagtggggctgctgtcctttgccaaaggctg
tctgctgtgatgacaaagttcactgctgtcctgaaggaacaacctgtagtgtgtccactggaaaatgcaa
caaaggtgatgttagcttagcctggtttgaaaagtcgcctgctaagaatgtccaaaatgaccaaaacatc
atgtgtgatgccacccacgaatgcccttcaaaacacacgtgctgtaagctggcctctggacagtggggct
gctgtcccttacccagtgctgtctgctgtaacgaccatgtccactgctgtcctgagggaacaacatgcag
tgtttcaacaggaaagtgtgaaaagggagaaatcaccttggcttggtttgagaaaacaagtgccataaca
gttgaagatgcaccaaatgtcatgtgtgatgccacccatgaatgcaaagctaaagaaacgtgctgtaaac
tggcgtctggacagtggggctgctgtcctcttccaaaggctgtctgctgtgatgacaaagttcactgctg
tcctgaaggaacaacctgtagtgtgtccactggaaaatgcaacaaaggtgacattagctttccctggttt
gagaaagttcctgctaacaatgtccaaaatgtccaaaacatgaagtgtgacgccacccacgagtgccctt
caaaacacacgtgctgtaagctggcctctggacagtggggctgctgtcccttacccagtgctgtctgctg
taacgaccacgtccactgctgtcccgaagatactacatgcgaagtttcatctggaaagtgcaagagaaaa
gacatcagctttccctggtttgagaaagttccagccataacaacagcacaagaagtttcaaatgtgaaat
gcgacgccacccatgaatgcccttcaaaacacacgtgcttgctgtctgctgtaacgaccacgtccactgc
tgtcccgaagatactacatgtgaagtgtcttctggaaagtgaaaagggagaaaacagtctaccatggttt
gagaaaacaagtgccataacagttgaagatgcaccaaatgtcatgtgtgatgccacccatgaatgcaaag
ctaaagaaacttgctgtaagctggcctctggacagtggggctgctgtcctcttccaaaggctgtctgctg
tgacgaccatgttcactgctgtccagaaggaacaacctatactacatgtgaggtgtcatcaggaaagtgc
aagagaaaggattctagcattccctggtttgaaaaagttccagctataacaactgaagaagtttcaaatg

taaaatgcgatgccacccacgaatgcccttcaaaacacacatgctgtaagctggcctctggacagtgggg
ctgctgtcccttacccagtgctgtctgctgtaacgaccatgttcactgctgtcccgaagatactacatgc
agtgtgtcgacaggaaagtgtgaaaagggagaaatcagccttccttggtttgagaaaacaagtgccataa
cagttgaagatgcaccaaatgtcatgtgtgatgccacccatgaatgcaaggcaagagaaacttgctgtaa
gctggagtctggacagtggggctgctgtcctcttccaaaggctgtctgctgtgatgacaaagttcattgc
tgtcctgaaggaactacctgtagtgtgtcaactggaaaatgtcacaaaggtgacgttagcattacttggt
tcgagaaagttccagctataacagctgaggaagaagtctccaatgtaaaatgcgatgccacccacgagtg
tccttcaaaacacacatgctgtaagctggcctctggacagtggggctgctgtcccctacccagtgctgtc
tgctgtaacgaccacgttcactgctgtcctgaagatacaacgtgcgaggtttcatctggaaagtgcaaga
gaaaggatgctagcattccttggtttgacaaagttccagctataacaactgaagaagtttcaaatgtaaa
atgcgatgcctcccacgaatgcccttcaaaacacacatgctgtaagctggcctctggacagtggggctgc
tgtcccttacccagtgctgtctgctgtaacgaccatgttcactgctgtcccgaagatactacatgcgagg
ctgtctgctgtgatgacaaagttcactgctgtcctgaaggaactacctgtagtgtgtcaactggaaaatg
tcacaaaggtgatgttagcattccctggtttgagaaagtgccagctataacagctgaggaagaagtttcc
aatgtcaaatgcgatgccacccacgagtgtccttcaaaacacacgtgctgtaagctggcctctggacagt
ggggctgctgtcccctacccagtgctgtctgctgtaacgaccatgttcactgctgtcccgaagatactac
atgcgaggtgtcttctggaaagtgtaagagaaaagacatcagctttccttggtttgagaagaaagcagcc
attaatgttgaagaaagcccaaacattaaatgtgactccacccatgagtgcaaggccactgacacttgct
gtaaactaaagtctggacagtggggctgctgtcctcttccaaaggctgtctgctgtgatgacaaagttca
ttgctgtcctgaaggaactacctgtagtgtgtcaactggtaaatgtcacaaaggtgatgttagcattccc
tggtttgagaaagttccagctataacagctgacaaagaagtctccaatgtcaaatgcgatgccacccacg
agtgtccttcaaaacacacgtgctgtaagctggcctctggacagtggggctgctgtcccttacccagtgc
tgtctgctgtaacgaccatgttcactgctgtccagaagatactacatttgaagatgcaccaaatgtcatg
tgtgacgccacccacgaatgcaaagctaaagaaacgtgctgcaagctggcctctggacagtggggctgct
gtcccttacccagtgctgtctgctgtaacgaccacgtccactgctgtccagaaggaactacttgtagtgt
gtcaacgggaaaatgcaagcatggtgacatcagcttaccctggtttgagaagaaggctgctttaacagtg
gatgttgagaagacccccctgtccaggaggtaaacaggaatgtgcaacaaactacacctgctgccagagag
gagccacaccttacacctggggggtgctgcgacttccctaacgctgtatgttgtccggatcgtaagacctg
ctgtcctaaaggataccactgtgatcctagcagagcaacgtgcttcaagagcgaaagtgatgacattgtt
gtagccaagcaacttgccataccggatactgaacaggctgaggtgggaaatgtgatatgtcctggtggtg
agtaccagtgcccagagaagaccacctgttgtgtgagtccctctggtacatttggatgctgtcctgtccc
cgaggctgtatgctgtgtggatggggtacactgttgcccaaaggaccacacgtgtgatccgtcatcacaa
acttgtaagagtgccagtggtcaactgccctggcaggacaagctgttagccaaacaagtacaggtgaaag
atattaagtgtggcagtagcacctccacttgcggagatacagagacctgctgtaagatggctactggaca
gtggggatgctgtcctatgcctaaggctgtctgctgtgaggaccacctacactgctgccctagaggctac
aagtgcgacatgccgaccagtacgtgtatccgtggtaacgacatccttcccatggccaagaaaagacccg
cagacatcttcaacaaatcttaa

cDNA data:

cDNA sequence data: No cDNA data (or empty cDNA entry).

Genomic data:

Note: Genomic data (exons). The exons before and after the granulin repeat sequence were predicted from ABEP02018454.1 (GI:227042074), because these were used in our analysis of releationships between modules in the various species. The 5 exons from the repeat were taken from the estExt_fgenesh2_pg.C_160005 model. They come from splicing within a highly repetitive sequence of about 27 granulin-module-coding sequences. There is not a clean read through this repeat because of frame shifts, but there may be sequence errors in this region, so the prediction within the repeat is quite tentative. Whatever the number in the transcript, however, the repeat modules are highly similar.

From NCBI and JGI: ABEP02018454.1 GI:227042074, and estExt_fgenesh2_pg.C_160005

Coding exon 1 **Exon type: sn**

Note: From start codon

ATGAGGTTACTGATAGTGCTCCTGTGCAGCCTGGCCCTCTGCAGTGCTGTGCCATTCCCCAAGGGGGCGC
ACCAATGCCCTGATGTCTGCCAACCTGAAGAAACCTGCTGCAAGTCCAACAGTACACTGACACATGGCTG
CTGTCCTATTTCTCAG

Coding exon 2 **Exon type: cn**

GCTACCTGTTGCAGTGACGGCCTCCACTGTTGCCCCAGCGGGTTCAAATGTGACCTTTCCCAGAATGCCT
GCCTGCAGGGAGACCTGACCTTGAATTGGTCCCCCAAAATGGAGGTCAAATTCCGCGCCACGATCACATG

TCCGGGCGGAACTCATGACTGTCCGGAGAATACCACTTGTTGTTTGACCGCCAAGGGCGACTATGGGTGC
TGCCCTCTTGCGAAG

Coding exon 3 Exon type: cn
GCTGCCTGCTGTAAGGATCACACCCACTGCTGCCCCTCTGGGTTCAAATGCGACGAGTCGGCGGGAAAGT
GTCGCCAAGGCAACAGGACACTCCCCTTCCTGGAGAAGTTTGCAGCCAGACCCAGGTTTGTTGCGAACCA
AGAACAGGTGTGCCCTGGCGGGAAGTCCACCTGTCCCACCACCAGCACCTGCTGTATGCTGAAGAATGGG
AAGTATGGCTGCTGTCCCATGGAAAAG

Coding exon 4 Exon type: c
GCTGTGTGTTGTGAAGACAAGGTCCACTGCTGTCCCAGCGGCTTCACCTGTGCAGCTACCAAGTGTAAGA
AGGGAGAGTACAGCATTCCTCTGTTCAGCAAAACAGAAGCTAAGATCAG

Coding exon 5 Exon type: n
GCAAATGCACCCAGCGAAAGACGCCAAAGTAGTGACGTGCCCCGACGGTCTGTCTGCCTGTAAGGACAAC
ACCACCTGCTGCAAGGATGACACTGGAGAGTACGGCTGCTGTCCAATACCACAG

Coding exon 6 Exon type: cn
GCTGAGTGCTGTAATGATCAGATCCACTGCTGTCCTGCTGGCTTCACCTGCCAGACAGAGAAGGGGAAGT
GTTCCAAGGGGGGCGTGGTCGTCCCTCTCCTGACTAAAACACGGGCTGATGTTTTGTCAGAGGAGGTTGA
GTCCGTCCATTGCGATGATGGAAGTGCCTGTCCAAACCATCACACCTGCTGCAAGACGCAGACTGGGAAA
TACGGCTGCTGTCCATTGCCAAAG

Coding exon 7 Exon type: c
GCCGTGTGCTGTCAGGATGAGATCCACTGCTGTCCTGACGGCTACACCTGTGACACTGCCAAGAGCAGGT
GTATCAAGGGCGACATGACCCTCCCCTGGCTGGAGAAACGACCTTCAAAGTCCTCCAGGAAG

Coding exon 8 Exon type: n
GACCCTGGAACCAAGCCCCCTCCCGGTGCAGTGGTCTGTCCCGACCACGTGTCAATGTGTCCAGACAACA
ACACCTGCTGTCTGCTGCAGGATGGGAGCTATGGCTGCTGCCCCGAGCCACAT

Coding exon 9 Exon type: cn
GCCGTGTGCTGCAGCGACCGTAAGCACTGCTGCCCACAAGGCTACACCTGCGACACCTCACAGGGAAAGT
GCCTGCAGGGCTACAGAACCATCCCGTGGATAGAGAACACGGTCGCGCCCCCCAACAAGGTCGTGACTCC
CTACAAGGTCGAGCCCCCAGTCAACCCCATCGACTGTGATGAGGAAGGCAAGGTCAAGTGCCCAGATAAG
AACACCTGCTGCAAGAAGACGACCGGAGAATGGGGGTGCTGTCCTCTTGAGAAG

Coding exon 10 Exon type: c'
Note: The notation c' for this unusual exon denotes encoding also the first Cys of the next granulin module
GCCCTGTGCTGTGAGGACAAGGTCCACTGCTGTCCCCACGGTACCGTGTGTAACAAAACCTCCGGGGAGT
GTCACTATCCTGATGGCACCTCCCAGGCCTGGGTGCAGAAGACACCACCCCTCCTGGACGTCAGGGATGA
TGTGAAAGATGTGCCGTGTCCCGATGGAAAG

Coding exon 11 Exon type: |nc|
Note: The notation |nc| denotes an exon encoding all but the first and last Cys of a granulin module
TCCGCTTGTGAGGAAGACTACACCTGCTGTAAGAACAGCACCGGCTATGGCTGCTGTCCCTTCTCCAGGG
CTGTCTGCTGTAACGACACGGTCCACTGCTGTCCCCAGCACTACACATGCAACTCTGGGGCTCCAG

Coding exon r1 Exon type: n'cncncncncnc+
Note: From estExt_fgenesh2_pg.C_160005. The notation n' indicates the exon encodes also the last Cys of the previous module, and + indicates the beginning of an N-half module truncated after the third Cys codon.
GAACCTGTATCCACAACTTCCTGCCCACTGTCCCAATGGCCAAGAAGACTACTCCAAAGATGGTACAACT
CCACAACGAAGAGATTGAAACTGACAATGAGATACTGAACAATGTAAGGTGCGACACATTCCATGAGTGC
CCAGCTAACAATACGTGCTGTAAGCTTTCCTCTGGACAGTGGGGCTGCTGTCCCTTGCCTTCGGCTGTCT
GCTGTGACGACCATGTCCACTGCTGTCCTGAAGATACAACATGCAGTGTGTCCACTGGAAAATGTCACAA
AGGTGATGCCAGCATTACTTGGTTTGAGAAGTTGCCTGCTAAGACTGTTCAGAATGACCCAAACATCATG
TGTGATGCCACCCACGAATGCCCTTCAAAACACACATGCTGTAAGCTGGCCTCTGGACAGTGGGGCTGCT
GTCCCTTACCCAGTGCTGTCTGCTGTAACGACCATGTTCACTGCTGTCCTGAAGGAACAACATGTAAAGT
CTCAACAGGAAAGTGTGAAAAGGGAGACATCACCTTGGCTTGGTTTGAGAAAACAAGTGCCATAACAGTT
GAAGATGCACCAAATGTCATGTGTGATGCCACCCATGAATGCAAAGCTAAAGAAACGTGCTGTAAACTAA
AGTCTGGACAGTGGGGCTGCTGTCCTTTGCCAAAGGCTGTCTGCTGTGATGACAAAGTTCACTGCTGTCC

TGAAGGAACAACCTGTAGTGTGTCCACTGGAAAATGCAACAAAGGTGATGTTAGCTTAGCCTGGTTTGAA
AAGTCGCCTGCTAAGAATGTCCAAAATGACCAAAACATCATGTGTGATGCCACCCACGAATGCCCTTCAA
AACACACGTGCTGTAAGCTGGCCTCTGGACAGTGGGGCTGCTGTCCCTTACCCAGTGCTGTCTGCTGTAA
CGACCATGTCCACTGCTGTCCTGAGGGAACAACATGCAGTGTTTCAACAGGAAAGTGTGAAAAGGGAGAA
ATCACCTTGGCTTGGTTTGAGAAAACAAGTGCCATAACAGTTGAAGATGCACCAAATGTCATGTGTGATG
CCACCCATGAATGCAAAGCTAAAGAAACGTGCTGTAAACTGGCGTCTGGACAGTGGGGCTGCTGTCCTCT
TCCAAAGGCTGTCTGCTGTGATGACAAAGTTCACTGCTGTCCTGAAGGAACAACCTGTAGTGTGTCCACT
GGAAAATGCAACAAAGGTGACATTAGCTTTCCCTGGTTTGAGAAAGTTCCTGCTAACAATGTCCAAAATG
TCCAAAACATGAAGTGTGACGCCACCCACGAGTGCCCTTCAAAACACACGTGCTGTAAGCTGGCCTCTGG
ACAGTGGGGCTGCTGTCCCTTACCCAGTGCTGTCTGCTGTAACGACCACGTCCACTGCTGTCCCGAAGAT
ACTACATGCGAAGTTTCATCTGGAAAGTGCAAGAGAAAAGACATCAGCTTTCCCTGGTTTGAGAAAGTTC
CAGCCATAACAACAGCACAAGAAGTTTCAAATGTGAAATGCGACGCCACCCATGAATGCCCTTCAAAACA
CACGTGCT

Coding exon r2 Exon type: jnc''

Note: From estExt_fgenesh2_pg.C_160005. Here j is a module joining sequence produced by a mostly out of frame C-half sequence, and c'' denotes encoding of a C-half truncated before the last two single Cys.

TGCTGTCTGCTGTAACGACCACGTCCACTGCTGTCCCGAAGATACTACATGTGAAGTGTCTTCTGGAAAG
TGAAAAGGGAGAAAACAGTCTACCATGGTTTGAGAAAACAAGTGCCATAACAGTTGAAGATGCACCAAAT
GTCATGTGTGATGCCACCCATGAATGCAAAGCTAAAGAAACTTGCTGTAAGCTGGCCTCTGGACAGTGGG
GCTGCTGTCCTCTTCCAAAGGCTGTCTGCTGTGACGACCATGTTCACTGCTGTCCAGAAGGAACAACCT

Coding exon r3 Exon type: ncncncnc''

Note: From estExt_fgenesh2_pg.C_160005. In this case the C-half truncation (c'') is after the penultimate single Cys.

ATACTACATGTGAGGTGTCATCAGGAAAGTGCAAGAGAAAGGATTCTAGCATTCCCTGGTTTGAAAAAGT
TCCAGCTATAACAACTGAAGAAGTTTCAAATGTAAAATGCGATGCCACCCACGAATGCCCTTCAAAACAC
ACATGCTGTAAGCTGGCCTCTGGACAGTGGGGCTGCTGTCCCTTACCCAGTGCTGTCTGCTGTAACGACC
ATGTTCACTGCTGTCCCGAAGATACTACATGCAGTGTGTCGACAGGAAAGTGTGAAAAGGGAGAAATCAG
CCTTCCTTGGTTTGAGAAAACAAGTGCCATAACAGTTGAAGATGCACCAAATGTCATGTGTGATGCCACC
CATGAATGCAAGGCAAGAGAAACTTGCTGTAAGCTGGAGTCTGGACAGTGGGGCTGCTGTCCTCTTCCAA
AGGCTGTCTGCTGTGATGACAAAGTTCATTGCTGTCCTGAAGGAACTACCTGTAGTGTGTCAACTGGAAA
ATGTCACAAAGGTGACGTTAGCATTACTTGGTTCGAGAAAGTTCCAGCTATAACAGCTGAGGAAGAAGTC
TCCAATGTAAAATGCGATGCCACCCACGAGTGTCCTTCAAAACACACATGCTGTAAGCTGGCCTCTGGAC
AGTGGGGCTGCTGTCCCCTACCCAGTGCTGTCTGCTGTAACGACCACGTTCACTGCTGTCCTGAAGATAC
AACGTGCGAGGTTTCATCTGGAAAGTGCAAGAGAAAGGATGCTAGCATTCCTTGGTTTGACAAAGTTCCA
GCTATAACAACTGAAGAAGTTTCAAATGTAAAATGCGATGCCTCCCACGAATGCCCTTCAAAACACACAT
GCTGTAAGCTGGCCTCTGGACAGTGGGGCTGCTGTCCCTTACCCAGTGCTGTCTGCTGTAACGACCATGT
TCACTGCTGTCCCGAAGATACTACATGCGAG

Coding exon r4 Exon type: cncncnc''

Note: From estExt_fgenesh2_pg.C_160005. Here c'' denotes encoding of a C-half truncated before the last two single Cys.

GCTGTCTGCTGTGATGACAAAGTTCACTGCTGTCCTGAAGGAACTACCTGTAGTGTGTCAACTGGAAAAT
GTCACAAAGGTGATGTTAGCATTCCCTGGTTTGAGAAAGTGCCAGCTATAACAGCTGAGGAAGAAGTTTC
CAATGTCAAATGCGATGCCACCCACGAGTGTCCTTCAAAACACACGTGCTGTAAGCTGGCCTCTGGACAG
TGGGGCTGCTGTCCCCTACCCAGTGCTGTCTGCTGTAACGACCATGTTCACTGCTGTCCCGAAGATACTA
CATGCGAGGTGTCTTCTGGAAAGTGTAAGAGAAAAGACATCAGCTTTCCTTGGTTTGAGAAGAAAGCAGC
CATTAATGTTGAAGAAAGCCCAAACATTAAATGTGACTCCACCCATGAGTGCAAGGCCACTGACACTTGC
TGTAAACTAAAGTCTGGACAGTGGGGCTGCTGTCCTCTTCCAAAGGCTGTCTGCTGTGATGACAAAGTTC
ATTGCTGTCCTGAAGGAACTACCTGTAGTGTGTCAACTGGTAAATGTCACAAAGGTGATGTTAGCATTCC
CTGGTTTGAGAAAGTTCCAGCTATAACAGCTGACAAAGAAGTCTCCAATGTCAAATGCGATGCCACCCAC
GAGTGTCCTTCAAAACACACGTGCTGTAAGCTGGCCTCTGGACAGTGGGGCTGCTGTCCCTTACCCAGTG
CTGTCTGCTGTAACGACCATGTTCACTGCTGTCCAGAAGATACTACAT

Coding exon r5 Exon type: nc

Note: From estExt_fgenesh2_pg.C_160005.

TTGAAGATGCACCAAATGTCATGTGTGACGCCACCCACGAATGCAAAGCTAAAGAAACGTGCTGCAAGCT
GGCCTCTGGACAGTGGGGCTGCTGTCCCTTACCCAGTGCTGTCTGCTGTAACGACCACGTCCACTGCTGT
CCAGAAGGAACTACTTGTAGTGTGTCAACGGGAAAATGCAAGCATGGTGACATCAGCTTACCCTGGTTTG
AGAAGAAGGCTGCTTTAACA

Coding exon u **Exon type: n**
GTGGATGTTGAGAAGACCCCCTGTCCAGGAGGTAAACAGGAATGTGCAACAAACTACACCTGCTGCCAGA
GAGGAGCCACACCTTACACCTGGGGGTGCTGCGACTTCCCTAAC

Coding exon v **Exon type: c**
GCTGTATGTTGTCCGGATCGTAAGACCTGCTGTCCTAAAGGATACCACTGTGATCCTAGCAGAGCAACGT
GCTTCAAGAGCGAAAGTGATGACATTGTTGTAGCCAAGCAACTTGCCATACCGGATACTGAACAGGCTGA
G

Coding exon w **Exon type: n**
GTGGGAAATGTGATATGTCCTGGTGGTGAGTACCAGTGCCCAGAGAAGACCACCTGTTGTGTGAGTCCCT
CTGGTACATTTGGATGCTGTCCTGTCCCCGAG

Coding exon x **Exon type: c**
GCTGTATGCTGTGTGGATGGGGTACACTGTTGCCCAAAGGACCACACGTGTGATCCGTCATCACAAACTT
GTAAGAGTGCCAGTGGTCAACTGCCCTGGCAGGACAAGCTGTTAGCCAAACAAGTACAG

Coding exon y **Exon type: n**
GTGAAAGATATTAAGTGTGGCAGTAGCACCTCCACTTGCGGAGATACAGAGACCTGCTGTAAGATGGCTA
CTGGACAGTGGGGATGCTGTCCTATGCCTAAG

Coding exon z **Exon type: c**
Note: Up to stop codon
GCTGTCTGCTGTGAGGACCACCTACACTGCTGCCCTAGAGGCTACAAGTGCGACATGCCGACCAGTACGT
GTATCCGTGGTAACGACATCCTTCCCATGGCCAAGAAAAGACCCGCAGACATCTTCAACAAATCTTAA

Genomic sequence note: A genomic sequence is not presented (see note 2 in header).

00036 **C. intestinalis progranulin (long)** Short name: **C_intL** Date entered: 2014-03

Species: ***Ciona intestinalis (yellow sea squirt)***
Taxonomy (via NCBI): *Eukaryota; Metazoa; Chordata; Tunicata; Ascidiacea; Enterogona; Phlebobranchia; Cionidae; Ciona.*
General Comment: We include the L (long) sufffix in case a short form is found, as we have found a short form in C. savignyi. The C. intestinalis data are currently better for the long form progranulin. Genomic data allow prediction of a 7 module progranulin (as in the protein sequence presented). The EST cDNA data allow assembly of 2 contigs representing the same gene. One encodes a complete 4 module progranulin having the first 2 and last 2 predicted modules. The other is incomplete, beginning in the middle repetitive sequence and encoding the last 4 modules of a longer product. We are assuming there are 7 modules in the maximum progranulin from this gene, bacause the gap in AABS01000126.1 is given as only 50 bases, but perhaps the number of nearly identical modules encoded in the middle of this gene is not definitely settled by the data at this time.

Protein Sequence
MNLYFILTCIVLSSFSTSSNGQVSNFGLKRILRPEYDTATVLCPDGRSECFSGSTCCKLSTGQYGCCPHP
NAVCCKDDKHCCPHNYKCNVQNSTCDMALDVQVQPIMLTDSVILNVGMVQCPDGRSACPDGNTCCKLASG
AYGCCPQPKAVCCSDHVHCCPQGYSCNVGSGTCLKQDSLSVVPWMEKQEAVAYNVGMVQCPDGRSACPDG
NTCCKLASGAYGCCPQPKAVCCSDHVHCCPQGYSCNVGSGTCLKQDSLSVVPWMEKQEAVTLNVGMVQCP
DGHSACPDGNTCCXXXXXXXXXXXXXXAVCCSDHVHCCPQGYSCNVGSGTCLKQDSLSVVPWMEKQDSVA
FNVGMVQCPDGRSACPDGNTCCKLASGAYGCCPQPKAVCCSDHVHCCPQGYSCNVGSGTCLKQDSLSVVP
WMEKQEAVAYNVGMVQCPDGRSACPDGNTCCKLASGAYGCCPQPKAVCCSDHVHCCPQGYSCNVGSGTCL
KQDSLSVVPWMEKQMSSLRMTLNQNKKLKLTKEKCTMCPDGSECPDYYTCCTLTSGRYGCCKFNSAVCCA
DHLHCCPYNTVCDTQRKVCLSRFGEIGFEPMVASTWSENEVTDNTDDESPIIL*

Transcript:
Note: So far the EST cDNA data indicate at least 2 alternatively spliced transcripts.

Predicted sequence:
Note: This hypothetical sequence comes from the maximum exon use.
Derivation: Based upon the analysis of genomic data (see below).
ATGAATTTGTACTTCATCTTAACTTGTATAGTTCTGTCTAGCTTCAGCACTTCATCAAATGGCCAAGTTT
CAAACTTCGGTTTAAAAAGAATTTTAAGACCAGAGTATGACACAGCCACAGTTCTTTGTCCAGATGGAAG

GTCCGAGTGTTTTTCTGGAAGCACTTGCTGCAAGTTGTCAACTGGTCAGTATGGTTGCTGTCCTCATCCA
AATGCTGTTTGTTGCAAGGATGATAAGCATTGCTGTCCACATAATTACAAATGCAATGTTCAAAATTCCA
CATGTGACATGGCCTTGGATGTACAAGTACAGCCAATCATGTTAACAGACTCCGTTATATTAAATGTTGG
TATGGTTCAATGTCCTGATGGTCGTTCTGCTTGTCCAGATGGTAACACTTGTTGTAAGCTAGCATCTGGT
GCATATGGTTGTTGCCCACAACCTAAAGCAGTATGCTGCTCAGATCATGTACATTGTTGTCCACAAGGTT
ATTCTTGTAATGTTGGCAGTGGAACTTGCCTTAAACAAGATTCTTTATCAGTTGTACCATGGATGGAGAA
GCAAGAAGCGGTTGCATATAATGTTGGTATGGTTCAATGTCCTGATGGTCGTTCTGCTTGTCCAGATGGT
AACACTTGTTGTAAGCTAGCATCTGGTGCATATGGTTGTTGCCCACAACCTAAAGCAGTTTGTTGCTCAG
ATCATGTACATTGTTGTCCACAAGGTTATTCTTGTAATGTTGGCAGTGGAACTTGCCTTAAACAAGATTC
TTTATCAGTTGTACCATGGATGGAGAAGCAAGAAGCGGTTACATTAAATGTTGGTATGGTTCAATGTCCC
GATGGTCATTCTGCTTGTCCAGATGGTAACACTTGTTGTAANNNNNNNNNNNNNNNNNNNNNNNNNNNNN
NNNNNNNNNNNGCAGTTTGTTGCTCAGATCATGTACATTGTTGTCCACAAGGTTATTCTTGTAATGTTGG
CAGTGGAACTTGCCTTAAACAAGATTCTTTATCAGTAGTACCATGGATGGAGAAGCAAGATTCTGTTGCA
TTTAATGTTGGTATGGTTCAATGTCCCGATGGTCGTTCTGCTTGTCCAGATGGTAACACTTGTTGTAAGC
TAGCATCTGGTGCATATGGTTGTTGCCCACAACCTAAAGCAGTATGCTGCTCAGATCATGTACATTGTTG
TCCACAAGGTTATTCTTGTAATGTTGGCAGTGGAACTTGCCTTAAACAAGATTCTTTATCAGTAGTACCA
TGGATGGAGAAGCAAGAAGCGGTTGCATATAATGTTGGTATGGTTCAATGTCCTGATGGTCGTTCTGCTT
GTCCAGATGGTAACACTTGTTGTAAGCTAGCATCTGGTGCATATGGTTGTTGCCCACAACCTAAAGCAGT
TTGTTGCTCAGATCATGTACATTGTTGTCCACAAGGTTATTCTTGTAATGTTGGCAGTGGCACTTGCCTT
AAACAAGATTCTTTATCAGTTGTACCATGGATGGAGAAGCAAATGTCATCCCTGAGAATGACTTTGAACC
AAAATAAAAAGCTTAAACTTACCAAAGAAAAATGCACAATGTGCCCCGATGGTTCGGAATGTCCGGATTA
TTACACTTGTTGCACATTAACCTCTGGAAGATATGGCTGTTGCAAGTTTAACTCGGCTGTATGTTGTGCT
GATCATTTGCACTGCTGCCCCTACAACACTGTGTGTGACACACAAAGAAAAGTTTGCCTTTCGAGATTTG
GTGAGATTGGTTTTGAACCAATGGTTGCTTCAACTTGGAGTGAGAATGAAGTAACAGACAACACAGATGA
TGAATCTCCAATTATACTTTAA

cDNA data:

From: NCBI est: Contig of GI:(184067240, 192945343, 47769051, 184134408, 184145879, 192907757, 183991537, 47779693, 183989778, 184101113, 184080464, 184078806, 23952661, 23950672, 47741963, 184015413, 192978372, 183262339, 183323666, 47735750, 184126120, 47784603, 24149927, 23923469, 184110755, 24162279)
Note: This cDNA contig represents a complete short spliced transcript encoding just 4 granulin modules.
ATAAGGTATTGCCGTTAAAACTAAGTATATAGGCTACATCATTTGTTGTTTAAACAGAAGTAAATATGAA
TTTGTACTTCATCTTAACTTGTATAGTTCTGTCTAGCTTCAGCACTTCATCAAATGGCCAAGTTTCAAAC
TTCGGTTTAAAAAGAATTTTAAGACCAGAGTATGACACAGCCACAGTTCTTTGTCCAGATGGAAGGTCCG
AGTGTTTTTCTGGAAGCACTTGCTGCAAGTTGTCAACTGGTCAGTATGGTTGCTGTCCTCATCCAAATGC
TGTTTGTTGCAAGGATGATAAGCATTGCTGTCCACATAATTACAAATGCAATGTTCAAAATTCCACATGT
GACATGGCCTTGGATGTACAAGTACAGCCAATCATGTTAACAGACTCCGTTATATTAAATGTTGGTATGG
TTCAATGTCCTGATGGTCATTCTGCTTGTCCAGATGGTAACACTTGTTGTAAGCTAGCATCTGGTGCATA
TGGTTGTTGCCCACAACCTAAAGCAGTATGCTGCTCGGATCATGTACATTGTTGTCCACAAGGTTATTCT
TGTAATGTTGGCAGTGGAACTTGCCTTAAACAAGATTCTTTATCAGTTGTACCATGGATGGAGAAGCAAG
AAGCTGTTGCATATAATGTTGGTATGGTTCAATGTCCCGATGGTCGTTCTGCTTGTCCAGATGGTAACAC
TTGTTGTAAGCTAGCATCTGGTGCATATGGTTGTTGCCCACAACCTAAAGCAGTTTGTTGCTCAGATCAT
GTACATTGTTGTCCACAAGGTTATTCTTGTAATGTTGGCAGTGGCACTTGCCTTAAACAAGATTCTTTAT
CAGTTGTACCATGGATGGAGAAGCAAATGTCATCCCTGAGAATGACTTTGAACCAAAATAAAAAGCTTAA
ACTTACCAAAGAAAAATGCACAATGTGCCCCGATGGTTCGGAATGTCCGGATTATTACACTTGTTGCACA
TTAACCTCTGGAAGATATGGCTGTTGCAAGTTTAACTCGGCTGTATGTTGTGCTGATCATTTGCACTGCT
GCCCCTACAACACTGTGTGTGACACACAAAGAAAAGTTTGCCTTTCGAGATTTGGTGAGATTGGTTTTGA
ACCAATGGTTGCTTCAACTTGGAGTGAGAATGAAGTAACAGACAACACAGATGATGAATCTCCAATTATA
CTTTAACTCACCGAAGCTAATTGAATATTCCCTTAATAATCAAATAAATGTGTATCTATTATCTAATGAA
TGTATATCTATCATCTATGCATATAGAACAGTTCTATTTGTTAATTTTGGATTGTGTATTTTTCTCTGAA
TGCGAATAAATTGATTGTATGCAAA

From: NCBI est: Contig of GI:(24156788, 184067241, 184123034, 183991538, 193031297, 184078807, 183347246, 192978373, 184086795, 192907758, 183262338, 183989779, 184126121, 23935852, 24165809, 24568171, 47955823, 47841386, 24570348, 47960792, 47800072, 47793741, 47958869, 23942826, 24175060, 183275187, 192945344, 23921482, 184123033, 183347247, 183275188, 183249457, 47765322)
Note: This cDNA contig represents an incomplete transcript missing sequence from the 5' end, but complete to polyA at 3' end.
AAGGTTATTCTTGTAATGTTGGCAGTGGCACTTGCCTTAAACAAGATTCTTTATCAGTTGTACCATGGAT

GGAGAAGCAAGAAGCGGTTACATTAAATGTTGGTATGGTTCAATGTCCTGATGGTCGTTCTGCTTGTCCA
GATGGTAACACTTGTTGTAAGCTAGCATCTGGTGCATATGGTTGTTGCCCACAACCTAAAGCAGTATGCT
GCTCGGATCATGTACATTGTTGTCCACAAGGTTATTCTTGTAATGTTGGCAGTGGAACTTGCCTTAAACA
AGATTCTTTATCAGTTGTACCATGGATGGAGAAGCAAGAAGCGGTTACATTTAATGTTGGTATGGTTCAA
TGTCCTGATGGTCGTTCTGCTTGTCCAGATGGTAACACTTGTTGTAAGCTAGCATCTGGTGCATATGGTT
GTTGCCCACAACCTAAAGCAGTTTGCTGCTCAGATCATGTACATTGTTGTCCACAAGGTTATTCTTGTAA
TGTTGGCAGTGGCACTTGCCTTAAACAAGATTCTTTATCAGTTGTACCATGGATGGAGAAGCAAGATGCT
GTTGCATTTAATGTTGGTATGGTTCAATGTCCCGATGGTCGTTCTGCTTGTCCAGATGGTAACACTTGTT
GTAAGCTAGCATCTGGTGCATATGGTTGTTGCCCACAACCTAAAGCAGTTTGTTGCTCAGATCATGTACA
TTGTTGTCCACAAGGTTATTCTTGTAATGTTGGCAGTGGCACTTGCCTTAAACAAGATTCTTTATCAGTT
GTACCATGGATGGAGAAGCAAATGTCATCCCTGAGAATGACTTTGAACCAAAATAAAAAGCTTAAACTTA
CCAAAGAAAAATGCACAATGTGCCCCGATGGTTCGGAATGTCCGGATTATTACACTTGTTGCACATTAAC
CTCTGGAAGATATGGCTGTTGCAAGTTTAACTCGGCTGTATGTTGTGCTGATCATTTGCACTGCTGCCCC
TACAACACTGTGTGTGACACACAAAGAAAAGTTTGCCTTTCGAGATTTGGTGAGATTGGTTTTGAACCAA
TGGTTGCTTCAACTTGGAGTGAGAATGAAGTAACATACAACACAGATGATGAATCTCCAATTATACTTTA
ACTCACCGAAGCTAATTGAATATTCCCTTAATAATCAAATAAATGTGTATCTATTATCTAATGAATGTAT
ATCTATCATCTATGCATATAGAACAGTTCTATTTGTTAATTTTGGATTGTGTATTTTTCTCTGAATGCGA
ATAAATTGATTGTATGCAAAAAAAAAA

Genomic data:

Note: Genomic data (exons). This gene contains only n and c exons. EAAA01000240.1 GI:330857085 contains 7 exons including the first coding exon with signal peptide. The reverse complement of AABS01000126.1 GI:26554616 contains the same, a short gap (given as about 50 bases), which coincides with the end of EAAA01000240.1, and the last 7 exons.

From NCBI wgs: AABS01000126.1 GI:26554616 (122052-130654) in reverse

Note: The relevant sequence has a short (50 base) gap in it and ends within the 3'ut of the last coding exon at a much larger gap. EST cDNA data shows that this last exon has errors which do not affect the C-half granulin module encoded in it, but shift frame to predict a different stop.

Coding exon 1 **Exon type: s**

Note: From the start codon. (The exon has 60 bases of upstream 5'ut).

ATGAATTTGTACTTCATCTTAACTTGTATAGTTCTGTCTAGCTTCAGCACTTCATCAAATGGCCAAGTTT
CAAACTTCGGTTTAAAAAGAATTTTAAGACCAGAGTATGACACAGCCACAG

Coding exon 2 **Exon type: n**

TTCTTTGTCCAGATGGAAGGTCCGAGTGTTTTTCTGGAAGCACTTGCTGCAAGTTGTCAACTGGTCAGTA
TGGTTGCTGTCCTCATCCAAAT

Coding exon 3 **Exon type: c**

GCTGTTTGTTGCAAGGATGATAAGCATTGCTGTCCACATAATTACAAATGCAATGTTCAAAATTCCACAT
GTGACATGGCCTTGGATGTACAAGTACAGCCAATCATGTTAACAG

Coding exon 4 **Exon type: n**

ACTCCGTTATATTAAATGTTGGTATGGTTCAATGTCCTGATGGTCGTTCTGCTTGTCCAGATGGTAACAC
TTGTTGTAAGCTAGCATCTGGTGCATATGGTTGTTGCCCACAACCTAAA

Coding exon 5 **Exon type: c**

GCAGTATGCTGCTCAGATCATGTACATTGTTGTCCACAAGGTTATTCTTGTAATGTTGGCAGTGGAACTT
GCCTTAAACAAGATTCTTTATCAGTTGTACCATGGATGGAGAAGCAAG

Coding exon 6 **Exon type: n**

AAGCGGTTGCATATAATGTTGGTATGGTTCAATGTCCTGATGGTCGTTCTGCTTGTCCAGATGGTAACAC
TTGTTGTAAGCTAGCATCTGGTGCATATGGTTGTTGCCCACAACCTAAA

Coding exon 7 **Exon type: c**

GCAGTTTGTTGCTCAGATCATGTACATTGTTGTCCACAAGGTTATTCTTGTAATGTTGGCAGTGGAACTT
GCCTTAAACAAGATTCTTTATCAGTTGTACCATGGATGGAGAAGCAAG

Coding exon 8 **Exon type: n**

Note: The gap in sequence here would have prevented automatic includion of this exon. It is one of the repetitive exons, and is expected to be much like exons 6 and 10.

AAGCGGTTACATTAAATGTTGGTATGGTTCAATGTCCCGATGGTCATTCTGCTTGTCCAGATGGTAACAC
TTGTTGTAANNNNNNNNNNNNNNNNNNNNNNNNNNNNNNNNNNNNNNNNNN

Coding exon 9 Exon type: c
GCAGTTTGTTGCTCAGATCATGTACATTGTTGTCCACAAGGTTATTCTTGTAATGTTGGCAGTGGAACTT
GCCTTAAACAAGATTCTTTATCAGTAGTACCATGGATGGAGAAGCAAG

Coding exon 10 Exon type: n
ATTCTGTTGCATTTAATGTTGGTATGGTTCAATGTCCCGATGGTCGTTCTGCTTGTCCAGATGGTAACAC
TTGTTGTAAGCTAGCATCTGGTGCATATGGTTGTTGCCCACAACCTAAA

Coding exon 11 Exon type: c
GCAGTATGCTGCTCAGATCATGTACATTGTTGTCCACAAGGTTATTCTTGTAATGTTGGCAGTGGAACTT
GCCTTAAACAAGATTCTTTATCAGTAGTACCATGGATGGAGAAGCAAG

Coding exon 12 Exon type: n
AAGCGGTTGCATATAATGTTGGTATGGTTCAATGTCCTGATGGTCGTTCTGCTTGTCCAGATGGTAACAC
TTGTTGTAAGCTAGCATCTGGTGCATATGGTTGTTGCCCACAACCTAAA

Coding exon 13 Exon type: c
GCAGTTTGTTGCTCAGATCATGTACATTGTTGTCCACAAGGTTATTCTTGTAATGTTGGCAGTGGCACTT
GCCTTAAACAAGATTCTTTATCAGTTGTACCATGGATGGAGAAGCAAATGTCATCCCTGAGAATGACTTT
GAACCAAAATAAAAAGCTTAA

Coding exon 14 Exon type: n
ACTTACCAAAGAAAAATGCACAATGTGCCCCGATGGTTCGGAATGTCCGGATTATTACACTTGTTGCACA
TTAACCTCTGGAAGATATGGCTGTTGCAAGTTTAACTCG

Coding exon 15 Exon type: c
Note: Up to the stop codon. A base (a) was added to correct the and of this exon's coding sequence based upon about 28 EST sequences confirming the read.
GCTGTATGTTGTGCTGATCATTTGCACTGCTGCCCCTACAACACTGTGTGTGACACACAAAGAAAAGTTT
GCCTTTCGAGATTTGGTGAGATTGGTTTTGAACCAATGGTTGCTTCAACTTGGAGTGAGAATGAAGTAAC
AGACAACACAGATGATGAaTCTCCAATTATACTTTAA

Genomic sequence note: A genomic sequence is not presented (see note 2 in header).

00032 **C. milii progranulin 1** Short name: **C_mil1 or Cm1** Date entered: 2014-03

Species: ***Callorhinchus milii (elephantfish)***
Taxonomy (via NCBI): *Eukaryota; Metazoa; Chordata; Craniata; Vertebrata; Chondrichthyes; Holocephali; Chimaeriformes; Callorhinchidae; Callorhinchus.*
General Comment: This has the character of a large form of fish progranulin (A/B). Genomic prediction almost completely confirmed by cDNA data. There is evidence of alternative splicing or allelic variation in EST data.

Protein Sequence
MFQAVVILWLATGWAAAHPCPDGSECLDEETCCKSGVETYACCPMPDAVCCSDELHCCPANTTCDLVHSA
CIQGFNLVSWVKKMPANYPATTKAVCCGDEQHCCPEGFRCETGGNSCIKISENEKAVICPDGASECPDGS
TCCLLPNDQWGCCPLEKAVCCNDRLHCCPFQTKCDLKMSKCLFPYGLVDMWEKVPARRRFSLTNVGDQDV
RCSSTASCSAGHTCCQLPSGRFGCCPSPEAVCCADHRHCCPTGSTCDTQAETCQMKGSSIPWLTKVQATV
RGNVELDVQCDGTVSCPTGATCCRASSGGWSCCPLPQAVCCEDHIHCCPTGHTCNLTKGTCDVPGFPIPW
FTKNPATVKDNEALRVMCDETASCPSGTTCCRKPSGYWGCCPMPEAVCCEDHIHCCPNGYTCETRTETCQ
QQIPPIPWVSKFPATVRRIEDEMADVQCDGTVSCPTGATCCRASSGGWSCCPLPQAVCCEDHLHCCPTGH
TCNLKKGTCDVPGFPIPWFTKNPATLKDNKALNVMCDKTASCPSGTTCCRKPSGYWGCCPVPEAVCCVDH
VHCCPRGLTCSDMKCEGNGETLPWLTKIPATEKLSAPGIKCDEKTICPPENTCCKQSSAEWSCCPLPQAV
CCEDREHCCPKGYTCNVGAGTCEKQSLSLLWEVQRASLSASDRNLPIVMCDDTHHCSSPATCCRSASGWA
CCPYEQAVCCGDRFCCPQGYVCDGALRECVKKPRLNWNLLISKHKKTFGTL*

Transcript:
Note: Support for prediction from EST cDNA data confirms all granulin modules. Just 48 codons in a module joining

region remain to be confirmed. Transcript beginning defined by JK876886.1. Last 7 modules well defined by a 20 EST contig.

Predicted sequence:

Note: Predicted from AAVX02031853.1 GI:564441399 from start codon to stop codon.
Derivation: Prediction from NCBI wgs AAVX02031853.1 GI:564441399
ATGTTCCAAGCTGTTGTGATCTTGTGGTTGGCCACTGGTTGGGCTGCAGCTCATCCATGTCCTGATGGCT
CCGAGTGCTTGGATGAAGAAACCTGCTGCAAGTCCGGTGTTGAGACATATGCTTGCTGTCCTATGCCAGA
TGCGGTGTGCTGCTCAGATGAATTGCATTGTTGCCCCGCTAATACCACCTGTGATCTGGTCCATTCAGCC
TGCATTCAAGGTTTCAATCTTGTGTCTTGGGTAAAGAAAATGCCAGCAAACTATCCAGCAACTACCAAGG
CTGTTTGCTGTGGTGATGAGCAGCACTGCTGTCCTGAAGGCTTCAGGTGTGAAACTGGGGGGAATTCCTG
CATTAAAATCAGTGAAAATGAAAAAGCAGTGATTTGCCCAGACGGGGCATCTGAATGCCCGGACGGCTCA
ACGTGCTGTTTGTTGCCAAATGATCAATGGGGTTGTTGTCCATTGGAAAAGGCCGTGTGTTGTAATGACC
GACTGCACTGTTGTCCTTTTCAAACAAAATGCGATTTGAAAATGTCCAAGTGTTTGTTTCCGTACGGTCT
GGTGGACATGTGGGAAAAAGTTCCAGCTCGCAGGAGATTTTCTTTAACAAATGTTGGAGATCAGGATGTG
CGGTGCAGCTCCACAGCGAGTTGCTCTGCTGGCCACACCTGCTGCCAGCTCCCCTCGGGCCGGTTCGGGT
GCTGTCCCTCACCAGAAGCAGTTTGCTGTGCTGACCACAGACATTGCTGTCCCACTGGCTCCACATGTGA
CACTCAGGCAGAAACCTGTCAGATGAAAGGTTCTTCCATCCCATGGCTCACCAAGGTCCAGGCCACTGTG
AGAGGCAATGTGGAGTTAGATGTCCAGTGTGATGGCACTGTGTCCTGTCCCACGGGTGCCACCTGCTGCA
GGGCCAGTTCTGGAGGTTGGAGCTGTTGCCCTCTGCCTCAGGCAGTTTGCTGTGAGGATCATATACACTG
TTGTCCTACTGGCCATACGTGTAACCTTACGAAAGGAACCTGTGATGTGCCCGGTTTTCCCATCCCCTGG
TTCACCAAGAATCCAGCGACTGTGAAAGACAACGAGGCGTTAAGAGTCATGTGTGATGAAACCGCTAGTT
GTCCTTCAGGGACCACCTGCTGCAGAAAACCCTCGGGATATTGGGGCTGCTGCCCTATGCCTGAGGCGGT
TTGCTGTGAGGACCACATTCACTGCTGTCCTAATGGCTACACGTGTGAAACTCGGACAGAAACCTGTCAG
CAGCAAATCCCTCCTATCCCCTGGGTGTCAAAGTTCCCAGCAACCGTGCGAAGGATTGAAGACGAGATGG
CTGATGTGCAGTGTGATGGCACTGTGTCCTGTCCCACGGGTGCCACCTGCTGCAGGGCCAGTTCTGGAGG
TTGGAGCTGTTGCCCTCTGCCTCAGGCAGTTTGCTGTGAGGATCATTTACACTGTTGTCCTACTGGCCAT
ACCTGTAATCTTAAGAAAGGAACCTGTGATGTGCCCGGTTTTCCCATCCCCTGGTTCACCAAGAATCCAG
CGACTCTGAAAGACAACAAGGCATTAAATGTCATGTGTGATAAAACCGCTAGTTGTCCTTCAGGGACCAC
CTGCTGCAGAAAGCCCTCGGGGTATTGGGGCTGCTGCCCTGTGCCTGAGGCAGTCTGTTGTGTGGACCAT
GTGCACTGCTGTCCTCGAGGGTTAACGTGCAGTGATATGAAGTGTGAAGGTAATGGAGAAACACTTCCTT
GGCTTACTAAGATACCAGCCACAGAGAAACTTTCTGCTCCAGGTATCAAATGTGATGAGAAGACAATATG
TCCACCAGAGAATACCTGCTGCAAACAGAGCTCAGCAGAGTGGAGCTGCTGTCCTTTGCCTCAAGCTGTG
TGTTGTGAGGATCGTGAGCATTGTTGTCCGAAGGGTTACACTTGTAATGTTGGTGCTGGTACCTGTGAGA
AGCAAAGCCTCTCTCTACTCTGGGAAGTCCAGAGAGCGTCTCTCTCTGCCAGTGACCGCAATCTCCCTAT
AGTGATGTGTGATGACACCCACCATTGCTCTTCTCCTGCCACTTGCTGTCGGTCTGCGTCAGGCTGGGCT
TGCTGCCCCTACGAACAAGCTGTGTGCTGTGGGGACAGGTTCTGCTGCCCACAGGGTTATGTCTGTGATG
GGGCACTGAGAGAATGTGTGAAGAAACCTCGCCTGAATTGGAATTTGTTAATTTCCAAACATAAAAAGAC
CTTTGGTACTCTGTGA

cDNA data:

From: NCBI est: JK876886.1 GI:408952554
Note: This is the only cDNA sequence data at present which confirms the first exons and encoding of the C-half module unpaired with an N-half.
ATTTAGGAGCTGTGCCTCAGAGACAGCAGTGTTGAATTTGTTCAGAGAGTTGCTGAACTGCATCCTTCTG
CAATTGAGATTACTCCATTCGGAGAAGCAGGCATGTTCCAAGCTGTTGTGATCTTGTGGTTGGCCACTGG
CTGGGCTGCAGCTCATCCATGTCCTGATGGCTCCGAGTGCTTGGATGAAGAAACCTGCTGCAAGTCCGGT
GTTGAGACATATGCTTGCCGTCCTATGCCAGATGCGGTGTGCTGCTCAGATGAATTGCATTGTTGCCCCG
CTAATACCACCTGTGATCTGGTCCATTCAGCCTGCATTCAAGGTTTCAATCTTGTGTCTTGGGTAAAGAA
AATGCCAGCAAACTATCCAGCAACTACCAAGGCTGTTTGCTGTGGTGATGAGCAGCACTGCTGTCCTGAA
GGCTTCAGGTGTGAAACTGGGGGGAATTCCTGCATTAAAATCAGTGGTGAAAATGAAAAAGCAGTGATTT
GCCCAGACGGGGCATCTGAATGCCCGGACGGCTCAACGTGCTGTTTGTTGCCAAATGATCAATGGGGTTG
TTGTCCATTGGAAAAGGCCGTGTGTTGTAATGACCGACTGCACTGTTGTCCTTTTCAAACAAAATGCGAT
TTGAAAATGTCCAAGTGTTTGTTTCCGTACGGTCTGGTGGACATGTGGGAAAAAGTTCCAGCTC

From: NCBI est: Contig of GI:(408959291, 408971809, 408984391, 408909623, 408993559, 408945979, 408972633, 408960618, 409007115, 408911307, 408954242, 408984430, 408929023, 408979615, 409017901, 408947103, 408945943, 408912119, 408980485, 408983476)
Note: This cDNA contig confirms exons encoding the last 7 granulin modules.

CAGGATGTGCGGTGCAGCTCCACAGCGAGTTGCTCTGCTGGCCACACCTGCTGCCAGCTCCCCTCGGGCC
GGTTCGGGTGCTGTCCCTCACCAGAAGCAGTTTGCTGTGCTGACCACAGACATTGCTGTCCCACTGGCTC
CACATGTGACACTCAGGCAGAAACCTGTCAGATGAAAGGTTCTTCCATCCCATGGCTCACCAAGGTCCAG
GCCACTGTGAGAGGCAATGTGGAGTTAGATGTCCAGTGTGATGGCACTGTGTCCTGTCCCACGGGTGCCA
CCTGCTGCAGGGCCAGTTCTGGAGGTTGGAGCTGTTGCCCTCTGCCTCAGGCAGTTTGCTGTGAGGATCA
TATACACTGTTGTCCTACTGGCCATACGTGTAACCTTACGAAAGGAACCTGTGATGTGCCCGGTTTTCCC
ATCCCCTGGTTCACCAAGAATCCAGCGACTGTGAAAGACAACGAGGCGTTAAGAGTCATGTGTGATGAAA
CCGCTAGTTGTCCTTCAGGGACCACCTGCTGCAGAAAACCCTCGGGATATTGGGGCTGCTGCCCTATGCC
TGAGGCGGTTTGCTGTGAGGACCACATTCACTGCTGTCCTAATGGCTACACGTGTGAAACTCGGACAGAA
ACCTGTCAGCAGCAAATCCCTCCTATCCCCTGGGTGTCAAAGTTCCCAGCAACCGTGCGAAGGATTGAAG
ACGAGATGGCTGATGTGCAGTGTGATGGCACTGTGTCCTGTCCCACGGGTGCCACCTGCTGCAGGGCCAG
TTCTGGAGGTTGGAGCTGTTGCCCTCTGCCTCAGGCAGTTTGCTGTGAGGATCATTTACACTGTTGTCCT
ACTGGCCATACCTGTAATCTTAAGAAAGGAACCTGTGATGTGCCCGGTTTTCCCATCCCCTGGTTCACCA
AGAATCCAGCGACTCTGAAAGACAACAAGGCATTAAATGTCATGTGTGATAAAACCGCTAGTTGTCCTTC
AGGGACCACCTGCTGCAGAAAGCCCTCGGGGTATTGGGGCTGCTGCCCTGTGCCTGAGGCAGTCTGTTGT
GTGGACCATGTGCACTGCTGTCCTCGAGGGTTAACGTGCAGTGATATGAAGTGTGAAGGTAATGGAGAAA
CACTTCCTTGGCTTACTAAGATACCAGCCACAGAGAAACTTTCTGCTCCAGGTATCAAATGTGATGAGAA
GACAATATGTCCACCAGAGAATACCTGCTGCAAACAGAGCTCAGCAGAGTGGAGCTGCTGTCCTTTGCCT
CAAGCTGTGTGTTGTGAGGATCGTGAGCATTGTTGTCCGAAGGGTTACACTTGTAATGTTGGTGCTGGTA
CCTGTGAGAAGCAAAGCCTCTCTCTACTCTGGGAAGTCCAGAGAGCGTCTCTCTCTGCCAGTGACCGCAA
TCTCCCTATAGTGATGTGTGATGACACCCACCATTGCTCTTCTCCTGCCACTTGCTGTCGGTCTGCGTCA
GGCTGGGCTTGCTGCCCCTACGAACAAGCTGTGTGCTGTGGGGACAGGTTCTGCTGCCCACAGGGTTATG
TCTGTGATGGGGCACTGAGAGAATGTGTGAAGAAACCTCGCCTGAATTGGAATTTGTTAATTTCCAAACA
TAAAAAGACCTTTGGTACTCTGTGATGCTCGGGTACCTTCTCTCCCATGTTATGCAGCCAATGAAATGCA
CTTGTTCTTCTGGAGCCTTTTGAACTCATAATTGCTTCTTGATTTTTTTTC

Genomic data:

Note: Genomic sequence is in AAVX02031853.1

From NCBI wgs: AAVX02031853.1 GI:564441399

Coding exon 1 **Exon type: sn**

Note: From the start codon. There is 5'ut sequence in this exon.

ATGTTCCAAGCTGTTGTGATCTTGTGGTTGGCCACTGGTTGGGCTGCAGCTCATCCATGTCCTGATGGCT
CCGAGTGCTTGGATGAAGAAACCTGCTGCAAGTCCGGTGTTGAGACATATGCTTGCTGTCCTATGCCAGA
T

Coding exon 2 **Exon type: c**

GCGGTGTGCTGCTCAGATGAATTGCATTGTTGCCCCGCTAATACCACCTGTGATCTGGTCCATTCAGCCT
GCATTCAAGGTTTCAATCTTGTGTCTTGGGTAAAGAAAATGCCAGCAAACTATCCAGCAACTACCAAG

Coding exon 3 **Exon type: c**

GCTGTTTGCTGTGGTGATGAGCAGCACTGCTGTCCTGAAGGCTTCAGGTGTGAAACTGGGGGGAATTCCT
GCATTAAAATCA

Coding exon 4 **Exon type: n**

GTGAAAATGAAAAAGCAGTGATTTGCCCAGACGGGGCATCTGAATGCCCGGACGGCTCAACGTGCTGTTT
GTTGCCAAATGATCAATGGGGTTGTTGTCCATTGGAAAAG

Coding exon 5 **Exon type: c**

GCCGTGTGTTGTAATGACCGACTGCACTGTTGTCCTTTTCAAACAAAATGCGATTTGAAAATGTCCAAGT
GTTTGTTTCCGTACGGTCTGGTGGACATGTGGGAAAAAGTTCCAGCTCGCAGGAGATTTTCTTTAACAAA
TGTTGGAG

Coding exon 6 **Exon type: n**

ATCAGGATGTGCGGTGCAGCTCCACAGCGAGTTGCTCTGCTGGCCACACCTGCTGCCAGCTCCCCTCGGG
CCGGTTCGGGTGCTGTCCCTCACCAGAA

Coding exon 7 **Exon type: cn**

GCAGTTTGCTGTGCTGACCACAGACATTGCTGTCCCACTGGCTCCACATGTGACACTCAGGCAGAAACCT
GTCAGATGAAAGGTTCTTCCATCCCATGGCTCACCAAGGTCCAGGCCACTGTGAGAGGCAATGTGGAGTT
AGATGTCCAGTGTGATGGCACTGTGTCCTGTCCCACGGGTGCCACCTGCTGCAGGGCCAGTTCTGGAGGT

TGGAGCTGTTGCCCTCTGCCTCAG

Coding exon 8 Exon type: cn
GCAGTTTGCTGTGAGGATCATATACACTGTTGTCCTACTGGCCATACGTGTAACCTTACGAAAGGAACCT
GTGATGTGCCCGGTTTTCCCATCCCCTGGTTCACCAAGAATCCAGCGACTGTGAAAGACAACGAGGCGTT
AAGAGTCATGTGTGATGAAACCGCTAGTTGTCCTTCAGGGACCACCTGCTGCAGAAAACCCTCGGGATAT
TGGGGCTGCTGCCCTATGCCTGAG

Coding exon 9 Exon type: cn
GCGGTTTGCTGTGAGGACCACATTCACTGCTGTCCTAATGGCTACACGTGTGAAACTCGGACAGAAACCT
GTCAGCAGCAAATCCCTCCTATCCCCTGGGTGTCAAAGTTCCCAGCAACCGTGCGAAGGATTGAAGACGA
GATGGCTGATGTGCAGTGTGATGGCACTGTGTCCTGTCCCACGGGTGCCACCTGCTGCAGGGCCAGTTCT
GGAGGTTGGAGCTGTTGCCCTCTGCCTCAG

Coding exon 10 Exon type: cn
GCAGTTTGCTGTGAGGATCATTTACACTGTTGTCCTACTGGCCATACCTGTAATCTTAAGAAAGGAACCT
GTGATGTGCCCGGTTTTCCCATCCCCTGGTTCACCAAGAATCCAGCGACTCTGAAAGACAACAAGGCATT
AAATGTCATGTGTGATAAAACCGCTAGTTGTCCTTCAGGGACCACCTGCTGCAGAAAGCCCTCGGGGTAT
TGGGGCTGCTGCCCTGTGCCTGAG

Coding exon 11 Exon type: cn
GCAGTCTGTTGTGTGGACCATGTGCACTGCTGTCCTCGAGGGTTAACGTGCAGTGATATGAAGTGTGAAG
GTAATGGAGAAACACTTCCTTGGCTTACTAAGATACCAGCCACAGAGAAACTTTCTGCTCCAGGTATCAA
ATGTGATGAGAAGACAATATGTCCACCAGAGAATACCTGCTGCAAACAGAGCTCAGCAGAGTGGAGCTGC
TGTCCTTTGCCTCAA

Coding exon 12 Exon type: cn
GCTGTGTGTTGTGAGGATCGTGAGCATTGTTGTCCGAAGGGTTACACTTGTAATGTTGGTGCTGGTACCT
GTGAGAAGCAAAGCCTCTCTCTACTCTGGGAAGTCCAGAGAGCGTCTCTCTCTGCCAGTGACCGCAATCT
CCCTATAGTGATGTGTGATGACACCCACCATTGCTCTTCTCCTGCCACTTGCTGTCGGTCTGCGTCAGGC
TGGGCTTGCTGCCCCCTACGAACAA

Coding exon 13 Exon type: c
Note: up to the stop codon.
GCTGTGTGCTGTGGGGACAGGTTCTGCTGCCCACAGGGTTATGTCTGTGATGGGGCACTGAGAGAATGTG
TGAAGAAACCTCGCCTGAATTGGAATTTGTTAATTTCCAAACATAAAAAGACCTTTGGTACTCTGTGA

Genomic sequence note: A genomic sequence is not presented (see note 2 in header).

00033 **C. milii progranulin 2** Short name: **C_mil2 or Cm2** Date entered: 2014-03

Species: ***Callorhinchus milii (elephantfish)***
Taxonomy (via NCBI): *Eukaryota; Metazoa; Chordata; Craniata; Vertebrata; Chondrichthyes; Holocephali; Chimaeriformes; Callorhinchidae; Callorhinchus.*
General Comment: This gene encodes a progranulin somewhat more related to the fish small forms. But the predicted progranulin has 7 modules, more in line with the long forms (A/B). Three of these modules, however, lack the last two single Cys of the defining Cys motif, and arise from a 3 fold repeat of a variant cn exon.

Protein Sequence
MFLLVTILLLREVVSGAVLCPDQSQCPEGTTCCLMQSSQYGCCPVPKGVCCSDHQHCCPQGYNCNNETCV
KGGLSIPYINKIPVLERLIARNSIESHSTKVFCDAIHYCQGGNTCCRNRTGEWACCGYPQATCCANGLNC
CPNEFTCEISASICTQNGLKIPMETKKPSQKIISLSSSVSSDSSAVKCDSVHSCPDGNTCCRQNTGHWSC
CPHPQTVCCFAGTMCCPKDYTSGVSTSSRAMNGLSIPLVSMISAVESALSTSVSSDSSAVKCDSLYSCPD
GNTCCRQNTGHWSCCPHPQTVCCFAGTRCCPKDYTSGVSTSSRAMNGLSIPLVSMISAVESALSTSVSSD
SSAVKCDSVYSCPDGNTCCRQNTGHWSCCPHPQTVCCFGGTRCCPKDFSTLFKTSSSLSVPVNRMKPVME
INALSIQFNSGRSVTYCDNLHYCSAENTCCRLPSGSWGCCPIPSATCCNDGVHCCPHGMNCDAENSFCTT
DSIRIPWFNKKPALIATDVVNSVGRDSSVVNCDKTYFCKDGLTCCPTAMGRWGCCPFPNGQCCKDKQTCC
PRHYKCSGGPKTMTCKWKIWN*

Transcript:

Note: Incomplete transcript cDNA data. Only 2 EST sequences. The C-terminal end confirmed in JK944366.1.

Predicted sequence:

Note: Predicted and partially supported by sequence cDNA data.

Derivation: Combined exons (start to stop) predicted from AAVX02002193.1 and AAVX02002194.1.

```
ATGTTTCTACTGGTAACAATTCTGTTGTTGAGGGAAGTGGTTTCTGGAGCAGTGCTATGCCCAGATCAAA
GTCAATGCCCGGAAGGAACAACATGCTGTCTGATGCAAAGTAGTCAATATGGCTGCTGTCCGGTTCCAAA
GGGTGTTTGTTGTTCAGACCATCAGCATTGTTGCCCACAAGGGTATAACTGTAATAATGAAACGTGTGTG
AAAGGAGGTCTTAGCATTCCTTACATCAACAAGATCCCTGTCTTGGAGAGATTGATTGCTCGCAACAGCA
TTGAGAGCCACAGCACAAAAGTATTCTGTGATGCCATTCATTACTGTCAAGGAGGAAATACATGCTGCAG
GAATCGAACTGGAGAATGGGCTTGTTGCGGTTATCCTCAAGCTACTTGCTGTGCTAATGGCCTAAACTGC
TGTCCTAATGAATTCACCTGTGAAATCTCTGCATCAATTTGTACACAAAATGGTCTCAAAATCCCAATGG
AAACAAAGAAACCATCACAAAAGATAATAAGTCTGAGCAGCAGTGTGAGCAGTGACAGCTCAGCTGTTAA
GTGTGATAGTGTGCATTCCTGTCCAGATGGAAACACTTGCTGTCGACAAAACACTGGACATTGGAGCTGC
TGCCCCCATCCTCAGACTGTCTGTTGTTTTGCTGGAACAATGTGCTGTCCCAAAGATTACACAAGTGGTG
TTTCCACTTCATCCAGAGCAATGAATGGCCTGAGCATTCCACTGGTGAGCATGATATCTGCAGTGGAGTC
AGCTCTGAGCACCAGTGTGAGCAGTGACAGCTCAGCTGTTAAGTGTGATAGTCTGTATTCCTGTCCAGAT
GGAAACACTTGCTGTCGACAAAACACTGGACATTGGAGCTGCTGCCCCCATCCTCAGACTGTCTGTTGTT
TTGCTGGAACAAGGTGCTGTCCCAAAGATTACACAAGTGGTGTTTCCACTTCATCCAGAGCAATGAATGG
CCTGAGCATTCCACTGGTGAGCATGATATCTGCAGTGGAGTCAGCTCTGAGCACCAGTGTGAGCAGTGAC
AGCTCAGCTGTTAAGTGTGATAGTGTGTATTCCTGTCCAGATGGAAACACTTGCTGTCGACAAAACACTG
GACATTGGAGCTGCTGCCCCCATCCTCAGACTGTGTGTTGTTTTGGTGGCACACGATGCTGTCCCAAGGA
TTTTTCTACTTTGTTCAAGACATCAAGTAGTCTGAGCGTTCCAGTTAACAGGATGAAACCTGTAATGGAG
ATAAACGCTTTGAGCATTCAGTTTAACAGTGGCAGATCAGTCACATACTGTGATAATCTGCATTACTGTT
CTGCTGAAAATACATGTTGCAGACTGCCTAGTGGATCTTGGGGATGTTGCCCCATTCCTTCTGCTACATG
TTGTAATGATGGTGTGCACTGCTGTCCTCATGGCATGAATTGTGATGCAGAAAATTCCTTCTGTACAACG
GATAGTATCCGTATCCCATGGTTCAACAAAAAACCTGCACTCATCGCAACAGACGTTGTCAATAGTGTTG
GTCGTGATAGTTCGGTGGTCAACTGTGATAAGACATATTTTTGTAAAGATGGACTGACCTGTTGTCCAAC
GGCTATGGGAAGATGGGGATGTTGCCCATTTCCTAATGGTCAATGTTGTAAAGATAAACAAACCTGCTGC
CCTCGGCACTACAAATGTAGTGGGGGTCCCAAAACAATGACCTGCAAATGGAAAATCTGGAACTAG
```

cDNA data:

From: NCBI est: JK928855.1 GI:408995594

Note: This cDNA encodes the last 2 variant modules and the beginning of the next (penultimate) normal module.

```
AGTGGTGTTTCCACTTCATCCAGAGCAATGAATGGCCTGAGCATTCCACTGGTGAGCATGATATCTGCAG
TGGAGTCAGCTCTGAGCACCAGTGTGAGCAGTGACAGCTCAGCTGTTCAGTGTGATAGTCTGTATTCCTG
TCCAGATGGAAACACTTGCTGTCGACAAATCACTGGACATTGGAGCTGCTGCCCTCATCCTCAGACTGTC
TGTTGTTTTGCTGGAACAATGTGCTGTCCCAAAGATTACACAAGTGGTGTTTCCACTTCATCCAGAGCAA
TGAATGGCCTGAGCATTCCACTGGTGAGCATGATATCTGCAGTGGAGCCAGCTCTGAGCACCAGTGTGAG
CAGTGACAGCTCAGCTGTTATGTGTGATAGTGTGTATTCCTGTCCAGATGGAAACACTTGCTGTCGACAA
AACACTGGACATTGGAGCTGCTGCCCCCATCCTCAGACTGTGTGTTGTTTTGGTGGCACACGATGCTGTC
CCAAGGATTTTTCTACTTTGTTCAAGACATCAAGTAGTCTGAGCGTTCTAGTTAACAGGATGAAACCTGC
AATGGAGATAAACGCTTTGAGCATTCAGTTTAACAGTGGCAGATCAGTCACATACTGTGATAATCTGCAT
TACTGTTCTGCTGAAAATACATGTTGCAGACTG
```

From: NCBI est: JK944366.1 GI:408946050

Note: This cDNA encodes DVVNSVGRDS SVVNCDKTYF CKDGLTCCPT AMGRWGCCPF XNGQCCKDKQ TCCPRHYKCS GGPKTMTCKW KIWN* and continues into 3'ut.

```
GACGTNGTCAATAGTGTTGGTCGTGATAGTTCGGTGGTCAACTGTGATAAGACATATTTTTGTAAAGATG
GACTGACCTGTTGTCCAACGGCTATGGGAAGATGGGGATGTTGCCCATTTCNTAATGGTCAATGTTGTAA
AGATAAACAAACCTGCTGCCCTCGGCACTACAAATGTAGTGGGGGTCCCAAAACAATGACCTGCAAATGG
AAAATCTGGAACTAGATCTCATCGGTTGGAAATACTGAAGAGTTTGAAAACTTTTGATGGATTTTTCATT
AACTTTGCTTTACTTCACGGTCATGTTTGCGTTAAATGTTGGAATAAATTATCCTTACATTTACATTTTA
CATTTACAGTTTTAACTTACATTTGATTTAATAATAATAGTGATCCTTGCATTTGATATAGCGCCTTATC
AAGTCTTCGAAACGTCTCAAAGCGCTTCACAGAATATACTGTGAAGTGAATTGACTGTATATTTTGTGGG
CATTTATATCTAGAGGATGTCTCAAAGTGCTTCACGGGATATACTTTAAAGTGGAGTAACTGCTGTATTT
GTAGGCTTGGTGCAGATTCAAATAATTGCTTATATCTGACTTTATTTGACTTTTTATAAACCTGGCATTT
CAGAAATCAGAAAATTAGTCATTTAAAAATGTTGCTTTGCTAGAATTGTCTTTGAAATACACAATCAGAT
TTTCTAACTTTAACAGTGCTACAATAATTTTCTCTTTTGAATTAAGTGATTGATTACAGTTGAAAACGTC
```

TATCTCTAAATTGTCTATTAAATCTGTGTTACTGATGATGT

Genomic data:

Note: Genomic sequence is in AAVX02002193.1 GI:564471076 and AAVX02002194.1 GI:564471075, which overlap by about 1600 bases very well,

From NCBI wgs: AAVX02002193.1 GI:564471076

Coding exon 1 **Exon type: sn**

Note: from the start codon (which appears to be the beginning of this exon)

ATGTTTCTACTGGTAACAATTCTGTTGTTGAGGGAAGTGGTTTCTGGAGCAGTGCTATGCCCAGATCAAA
GTCAATGCCCGGAAGGAACAACATGCTGTCTGATGCAAAGTAGTCAATATGGCTGCTGTCCGGTTCCAAA
G

Coding exon 2 **Exon type: cn**

GGTGTTTGTTGTTCAGACCATCAGCATTGTTGCCCACAAGGGTATAACTGTAATAATGAAACGTGTGTGA
AAGGAGGTCTTAGCATTCCTTACATCAACAAGATCCCTGTCTTGGAGAGATTGATTGCTCGCAACAGCAT
TGAGAGCCACAGCACAAAAGTATTCTGTGATGCCATTCATTACTGTCAAGGAGGAAATACATGCTGCAGG
AATCGAACTGGAGAATGGGCTTGTTGCGGTTATCCTCAA

Coding exon 3 **Exon type: cn**

GCTACTTGCTGTGCTAATGGCCTAAACTGCTGTCCTAATGAATTCACCTGTGAAATCTCTGCATCAATTT
GTACACAAAATGGTCTCAAAATCCCAATGGAAACAAAGAAACCATCACAAAAGATAATAAGTCTGAGCAG
CAGTGTGAGCAGTGACAGCTCAGCTGTTAAGTGTGATAGTGTGCATTCCTGTCCAGATGGAAACACTTGC
TGTCGACAAAACACTGGACATTGGAGCTGCTGCCCCCATCCTCAG

Coding exon 4 **Exon type: c'n**

Note: This is a cn variant in whivh the encoded C-half module lacks the lasst 2 single Cys of the normal motif.

ACTGTCTGTTGTTTTGCTGGAACAATGTGCTGTCCCAAAGATTACACAAGTGGTGTTTCCACTTCATCCA
GAGCAATGAATGGCCTGAGCATTCCACTGGTGAGCATGATATCTGCAGTGGAGTCAGCTCTGAGCACCAG
TGTGAGCAGTGACAGCTCAGCTGTTAAGTGTGATAGTCTGTATTCCTGTCCAGATGGAAACACTTGCTGT
CGACAAAACACTGGACATTGGAGCTGCTGCCCCCATCCTCAG

From NCBI wgs: AAVX02002194.1 GI:564471075

Note: Because of the very high level sequence identity with exon 4 and the last part of AAVX02002193.1, the first exon in this wgs sequence is assumed to be the same exon 4 and is not given here.

Coding exon 5 **Exon type: c'n**

Note: See exon 4 comment.

ACTGTCTGTTGTTTTGCTGGAACAAGGTGCTGTCCCAAAGATTACACAAGTGGTGTTTCCACTTCATCCA
GAGCAATGAATGGCCTGAGCATTCCACTGGTGAGCATGATATCTGCAGTGGAGTCAGCTCTGAGCACCAG
TGTGAGCAGTGACAGCTCAGCTGTTAAGTGTGATAGTGTGTATTCCTGTCCAGATGGAAACACTTGCTGT
CGACAAAACACTGGACATTGGAGCTGCTGCCCCCATCCTCAG

Coding exon 6 **Exon type: c'n**

Note: See exon 4 comment.

ACTGTGTGTTGTTTTGGTGGCACACGATGCTGTCCCAAGGATTTTTCTACTTTGTTCAAGACATCAAGTA
GTCTGAGCGTTCCAGTTAACAGGATGAAACCTGTAATGGAGATAAACGCTTTGAGCATTCAGTTTAACAG
TGGCAGATCAGTCACATACTGTGATAATCTGCATTACTGTTCTGCTGAAAATACATGTTGCAGACTGCCT
AGTGGATCTTGGGGATGTTGCCCCATTCCTTCT

Coding exon 7 **Exon type: cn**

GCTACATGTTGTAATGATGGTGTGCACTGCTGTCCTCATGGCATGAATTGTGATGCAGAAAATTCCTTCT
GTACAACGGATAGTATCCGTATCCCATGGTTCAACAAAAAACCTGCACTCATCGCAACAGACGTTGTCAA
TAGTGTTGGTCGTGATAGTTCGGTGGTCAACTGTGATAAGACATATTTTTGTAAAGATGGACTGACCTGT
TGTCCAACGGCTATGGGAAGATGGGGATGTTGCCCATTTCCTAAT

Coding exon 8 **Exon type: c**

Note: up to the stop codon

GGTCAATGTTGTAAAGATAAACAAACCTGCTGCCCTCGGCACTACAAATGTAGTGGGGGTCCCAAAACAA
TGACCTGCAAATGGAAAATCTGGAACTAG

Genomic sequence note: A genomic sequence is not presented (see note 2 in header).

00005 **Chicken progranulin** Short name: **G_gal or chick** Date entered: 2014-03

Species: ***Gallus gallus (chicken)***
Taxonomy (via NCBI): *Eukaryota; Metazoa; Chordata; Craniata; Vertebrata; Euteleostomi; Archosauria; Aves; Neognathae; Galliformes; Phasianidae; Phasianinae; Gallus.*
General Comment: Transcript well defined by EST data. Partial genomic data in 2 unconnected wgs sequences.

Protein Sequence

```
MRTALTLWLALIGGAAVTALRCPDGASECPQNTTCCGTASGAWGCCPMPEAVCCRDEEHCCPHSTSCDLE
RGRCVSPTGDVPMATKFPAWKRPRGAAAQPRLRVPAVALLRVQCPDNSSACPDGATCCQLPSGRYGCCPL
QNAVCCGDGWHCCPQGTACDLQRSMCTSVRDVARVGDVKCDDEMSCPDGNTCCRLSSGQWGCCPLEQAVC
CPDHIHCCPQGYTCDPQGGTCLQGGVRLPWLRKTPALWGRGGDVRCDDRTSCPDGSTCCRLSGGAWGCCP
LEQAVCCKDHQHCCPQGYTCDIATQSCEKL*
```

Transcript:

The cDNA data define the transcript well (below), so no predicted sequence is required.

cDNA data:

From: NCBI: Chick_pgn_EST_contig of GI: 20209372, 5884962, 46297579, 6477511, 53546304, 15085465, 14004522, 68327488, 18611534, 18471080, 32372599, 14005635, 18608360, 15083859, 25365290, 75477822, 30954406, 18611004, 46302282, 18612424, 18431575, 14475114, 25340793, 25746988, 90217465, 41336278, 14006743
Note: cDNA sequence data:

```
GAGGGGGCGTGGCTCGCGGAGGGGAGGGGCGGGGCGTGAAGGGAGGAGAGAGTCCGGAGCGTGCAGGGTG
TCCCGGCCATGAGGACGGCGCTGACGCTGTGGTTGGCCCTCATTGGGGGCGCTGCGGTGACGGCGCTGCG
GTGCCCCGACGGCGCCTCCGAGTGCCCCCAAAACACCACATGCTGCGGCACCGCGAGCGGAGCCTGGGGG
TGCTGCCCCATGCCGGAGGCCGTGTGCTGCCGGGATGAGGAGCACTGCTGTCCCCACTCCACCAGCTGTG
ATTTGGAGCGCGGGCGCTGTGTGTCCCCTACGGGGGACGTCCCCATGGCCACCAAATTCCCGGCCTGGAA
GAGACCGCGCGGTGCTGCGGCACAGCCCCGGCTCCGCGTCCCAGCAGTGGCGCTGCTCCGGGTGCAGTGT
CCCGACAACAGCTCCGCGTGTCCCGACGGCGCCACGTGCTGCCAGCTGCCCTCGGGGCGCTACGGCTGCT
GTCCCCTGCAGAACGCCGTGTGCTGCGGCGACGGGTGGCACTGCTGTCCCCAGGGCACCGCGTGTGACCT
GCAGCGCTCCATGTGCACCTCGGTGCGGGACGTGGCGCGCGTTGGCGACGTGAAGTGTGACGATGAGATG
AGCTGTCCCGACGGGAACACGTGCTGCAGGCTGAGCTCCGGGCAGTGGGGGTGCTGCCCGCTGGAGCAGG
CCGTGTGCTGCCCCGACCACATCCACTGCTGCCCCCAGGGCTACACCTGCGACCCCCAGGGGGGGACGTG
CCTGCAGGGGGGGGTCCGCCTGCCCTGGCTGAGGAAGACCCCGGCACTGTGGGGTCGGGGTGGGGACGTG
CGGTGCGACGACAGGACGAGCTGTCCCGATGGGAGCACGTGCTGCAGGCTGAGCGGGGGCGCGTGGGGGT
GCTGCCCTCTGGAGCAGGCTGTGTGCTGTAAGGACCACCAGCACTGCTGCCCCCAGGGCTACACCTGCGA
CATCGCCACGCAGAGCTGCGAGAAGCTGTGACCCCCCCGGGGCTGTGACCCCCCCACCCCAATAAAGGGT
TTGTAGGAAAAAAAACCCAAAGGATTTTGGGGGAGGAAGAGGCGGAGGC
```

Genomic data:

Note: Genomic sequence remains very limited. There are two whole genome shotgun contigs which do not overlap. Numbering of the coding exons is based upon comparison with data from the Zebrafinch, which, while also incomplete, gets closer to the beginning of the gene.
From NCBI: AADN03008716.1 GI:354533746
Note: The exon sequences are from the reverse complement of the contig.

Coding exon 4 **Exon type: n**

```
CGCTGCTCCGGGTGCAGTGTCCCGACAACAGCTCCGCGTGTCCCGACGGCGCCACGTGCTGCCAGCTGCC
CTCGGGGCGCTACGGCTGCTGTCCCCTGCAGAAC
```

Coding exon 5 **Exon type: c**

```
GCCGTGTGCTGCGGCGACGGGTGGCACTGCTGTCCCCAGGGCACCGCGTGTGACCTGCAGCGCTCCATGT
GCACCTCGGTGCGGGACGTGGCGCGCG
```

Coding exon 6 **Exon type: n**

```
TTGGCGACGTGAAGTGTGACGATGAGATGAGCTGTCCCGACGGGAACACGTGCTGCAGGCTGAGCTCCGG
GCAGTGGGGGTGCTGCCCGCTGGAGCAG
```

Coding exon 7 **Exon type: cn**

Note: Incomplete at the end of the sequence data. The expected completion is added in lower case based upon the cDNA sequece.
GCCGTGTGCTGCCCCGACCACATCCACTGCTGCCCCCAGGGCTACACCTGCGACCCCCAGGGGGGGACGT
GCCTGCAGGGGGGGGTCCGCCTGCCCTGGCTGAGGAAGACCCCGGCGCTGTGGGGTCGGGGTGGGGACGT
GCGGTGCGACGACAGGacgagctgtcccgatgggagcacgtgctgcaggctgagcgggggcgcgtggggg
tgctgccctctggagcag

From NCBI: AADN03022721.1 GI:354519740
Coding exon 8 **Exon type: c**
Note: Up to the stop codon.
GCTGTGTGCTGTAAGGACCACCAGCACTGCTGCCCCCAGGGCTACACCTGCGACATCGCCACGCAGAGCT
GCGAGAAGCTGTGA

Genomic sequence note: A genomic sequence is not presented (see note 2 in header).

00018 **Danio rerio progranulin 1** Short name: **D_rer1 or Dr1** Date entered: 2014-03

Species: ***Danio rerio (zebrafish)***
Taxonomy (via NCBI): *Eukaryota; Metazoa; Chordata; Craniata; Vertebrata; Euteleostomi; Actinopterygii; Neopterygii; Teleostei; Ostariophysi; Cypriniformes; Cyprinidae; Danio.*
General Comment: Good cDNA and genomic data.

Protein Sequence
MFPVLMLLMAALVAADEPLLDLSIPVETVDTSASVIHCDAQTVCPDGTTCCLSPYGIWSCCPYSMGQCCR
DGIHCCQHGYRCDSTSTRCLRGWLTLPSSFQKATRTFQKDQTHAETVQCEGNFYCPAEKFCCKTRTGQWG
CCSGLEL*

Transcript:
The cDNA data define the transcript well (below), so no predicted sequence is required.

cDNA data:
From: NCBI: AF273479.1 GI:14279723
Note: cDNA sequence data:
ATGTTCCCAGTGTTGATGTTACTCATGGCGGCTCTGGTGGCTGCAGATGAGCCTCTGCTTGATCTGTCTA
TCCCGGTGGAGACTGTAGACACCTCTGCCTCTGTGATTCACTGCGATGCTCAAACTGTGTGTCCAGACGG
AACAACATGCTGTCTGAGTCCATATGGCATATGGTCATGTTGTCCTTACTCAATGGGTCAGTGTTGCAGG
GATGGGATTCACTGTTGTCAACATGGATATCGCTGCGATTCCACCTCCACCCGATGCCTGCGGGGCTGGC
TGACGCTGCCATCGTCTTTCCAGAAGGCCACCAGAACCTTCCAGAAAGATCAGACCCACGCTGAGACTGT
CCAATGTGAAGGAAATTTCTACTGCCCGGCTGAGAAGTTTTGCTGCAAGACTAGAACTGGCCAGTGGGGC
TGCTGCAGCGGGTTGGAGTTGTAAGCAAACAACCACTGTTACACTGGTTCACCTCTTACTGAAGACTGTC
AAAATGCAAGAGGCTCCAAACTTCATTCATGTTTCAGGCTATATTCTTACATCCAGGCTGAATTAGTGTG
AAATAAAAACTAATAGAATTACTGTCAAAATGCACCGTCAAGG

Genomic data:
Note: Genomic data (exons).
From NCBI: NC_007130.5 (41147019..41152510, complement)
Note: Also in Ensembl grn1 ENSDARG00000089362 Chromosome 19: 41, 147, 019-41, 152, 591 reverse strand.
Coding exon 1 **Exon type: sn**
Note: from the start codon
ATGTTCCCAGTGTTGATGTTACTCATGGCGGCTCTGGTGGCTGCAGATGAGCCTCTGCTTGATCTGTCTA
TCCCGGTGGAGACTGTAGACACCTCTGCCTCTGTGATTCACTGCGATGCTCAAACTGTGTGTCCAGACGG
AACAACATGCTGTCTGAGTCCATATGGCATATGGTCATGTTGTCCTTACTCAATG

Coding exon 2 **Exon type: c**
GGTCAGTGTTGCAGGGATGGGATTCACTGTTGTCAACATGGATATCGCTGCGATTCCACCTCCACCCGAT
GCCTGCGGGGCTGGCTGACGCTGCCATCGTCTTTCCAGAAGGCCACCAGAACCTTCCAGAAAGATCAG

Coding exon 3 **Exon type: n**
Note: unpaired with a c-exon encodes a paragranulin.

ACCCACGCTGAGACTGTCCAATGTGAAGGAAATTTCTACTGCCCGGCTGAGAAGTTTTGCTGCAAGACTA
GAACTGGCCAGTGGGGCTGCTGCAGCG

Coding exon 4 **Exon type:** **termination and (not shown) 3'ut**
Note: up to the stop codon
GGTTGGAGTTGTAA

Genomic sequence note: A genomic sequence is not presented (see note 2 in header).

00019 **Danio rerio progranulin 2** Short name: **D_rer2 or Dr2** Date entered: 2014-03

Species: ***Danio rerio (zebrafish)***
Taxonomy (via NCBI): *Eukaryota; Metazoa; Chordata; Craniata; Vertebrata; Euteleostomi; Actinopterygii; Neopterygii; Teleostei; Ostariophysi; Cypriniformes; Cyprinidae; Danio.*
General Comment: Good cDNA and genomic data.

Protein Sequence

MFPVLMLLMAALVAADEPLLDLSIPMETEDVSASVIHCDARTVCPDRTTCCRTPYGKWTCCPFPMGQCCR
DGIHCCRHGYRCNFASTRCLRGWLSLPSSFQEATRTFEKDQTQAETVQCEGNFYCPAEKFCCKTGTGQWG
CCSELEL*

Transcript:

The cDNA data define the transcript well (below), so no predicted sequence is required.

cDNA data:

From: NCBI: NM_212756.1 GI:47086536
Note: cDNA sequence data:
ATGTTCCCAGTGTTGATGTTACTCATGGCGGCTCTGGTGGCTGCAGATGAGCCTCTGCTGGATCTGTCCA
TTCCGATGGAGACTGAAGACGTCTCTGCCTCTGTGATTCACTGCGATGCTCGAACTGTGTGTCCAGATAG
AACAACATGCTGTCGGACTCCATATGGCAAATGGACATGTTGTCCCTTCCCAATGGGTCAGTGTTGCAGG
GATGGGATTCACTGCTGTCGACACGGTTATCGCTGCAATTTCGCCTCCACCAGATGCCTGCGAGGCTGGC
TGTCACTGCCATCGTCTTTCCAGGAGGCCACCAGAACCTTCGAGAAAGATCAGACCCAGGCTGAGACTGT
CCAATGTGAAGGAAATTTCTACTGCCCGGCTGAGAAGTTTTGCTGCAAGACTGGAACTGGCCAGTGGGGC
TGCTGCAGTGAGTTGGAGTTGTAAGCAAACAACCACTGTTACACTGGTTCACCTCTTACTGAAGACTGTC
AAAATGCAAGAGGC

Genomic data:

Note: Genomic data (exons).
From NCBI: NC_007130.5 (41156407..41159839, complement)
Note: Also in Ensembl grn2 ENSDARG00000088641 Chromosome 19: 41, 156, 804-41, 159, 440 reverse strand.
Coding exon 1 **Exon type:** **sn**
Note: from the start codon.
ATGTTCCCAGTGTTGATGTTACTCATGGCGGCTCTGGTGGCTGCAGATGAGCCTCTGCTGGATCTGTCCA
TTCCGATGGAGACTGAAGACGTCTCTGCCTCTGTGATTCACTGCGATGCTCGAACTGTGTGTCCAGATAG
AACAACATGCTGTCGGACTCCATATGGCAAATGGACATGTTGTCCCTTCCCAATG

Coding exon 2 **Exon type:** **c**
GGTCAGTGTTGCAGGGATGGGATTCACTGCTGTCGACACGGTTATCGCTGCAATTTCGCCTCCACCAGAT
GCCTGCGAGGCTGGCTGTCACTGCCATCGTCTTTCCAGGAGGCCACCAGAACCTTCGAGAAAGATCAG

Coding exon 3 **Exon type:** **n**
Note: unpaired with a c-exon encodes a paragranulin.
ACCCAGGCTGAGACTGTCCAATGTGAAGGAAATTTCTACTGCCCGGCTGAGAAGTTTTGCTGCAAGACTG
GAACTGGCCAGTGGGGCTGCTGCAGTG

Coding exon 4 **Exon type:** **termination and (not shown) 3'ut.**
Note: up to the stop codon.
AGTTGGAGTTGTAA

Genomic sequence note: A genomic sequence is not presented (see note 2 in header).

00016 **Danio rerio progranulin A** Short name: **D_rerA or DrA** Date entered: 2014-03

Species: ***Danio rerio (zebrafish)***
Taxonomy (via NCBI): *Eukaryota; Metazoa; Chordata; Craniata; Vertebrata; Euteleostomi; Actinopterygii; Neopterygii; Teleostei; Ostariophysi; Cypriniformes; Cyprinidae; Danio.*
General Comment: Good genomic and transcript data. in the middle repeat of very similar module sequences, EST data support a possible 1 or 2 additional modules encoded in the tanscript from a -c-n- exon repeat, but not an additional cn exon.

Protein Sequence

MLRLTVCLAVVTLVICSQCPDNEVCEAGQSCCQDPTGGFSCCPFHHGECCEDHLHCCPEGMLCSVKDLTC
TNATHTEPLADRTQAKKPDLPKSFRMIFSMPASESDISCPDGSSCPAEFSCLLMSTSYGCCPVAQGLACS
DGKHCCPNDHECSSDSSLCVKRKVKVETVLCGNGTSECPADTTCCQAEDGLWGCCPMPKAVCCDDKIHCC
PEDTVCDVKALKCISSTNQELPMWDKFPARLRAEWEDHKQKKPETQRTTTRPTGTTSTNTAANQMTTLPA
EHQAVSSDVPCNDTAACADGTTCCKTKDGGWACCPLPEAVCCEDFIHCCPHGKKCDVAAGSCDDPSGSVP
WVEKVPVRPIKKQKVAVTQVSSLSSDVPCNDTAACADGTTCCKTKDGDWACCPLPEAVCCEDFVHCCPKG
KKCNIAAMKCEDPSCTGEPLVKQTPVQSTTTPNVIGKQKSNVPCNDTAACADGTTCCKTKDGDWACCPLP
EAVCCEDFIHCCPHGKKCDLAAGSCDDPSGSVPWVEKVPVRPIKKQKVAVTKVSSVSSDVPCNDTAACAD
GTTCCKTKEGGWACCPLPEAVCCEDFIHCCPHGKKCNVAAGSCDDPSGSVPWVEKVPVHLRAGQRSSGKV
KCNATHGCPESSTCCKNIAGEWGCCPFSQAVCCTDGEHCCPAHYKCNLSSVSCIKGDVVIPWYNKIAAES
TPAPKLDLGVVKCDEQSSCSADSTCCLLSKEETGCCPFPEAVCCPDQKHCCPEGYRCDLRRRSCVKTTRL
YVEITQLTHIRSNKPQPSVIVKDVQCGGGFSCHDGETCCPTSQTTWGCCPSPKAVCCDDMQHCCPAGYKC
GPGGTCISAGDLDWSNWVNWKLFFSKKKRALTL*

Transcript:

The cDNA data define the transcript well (below), so no predicted sequence is required.

cDNA data:

From: NCBI: AF375477.3 GI:77744586
Note: This cDNA sequence is the one used in our analyses.

CAAAGGCAGACCTCTAATAAAGGCGGAGCTTTAAAGAATTTTACATTTAAGAGCAATCTTTAATTAATGC
AAGCACAGTGTGGGGAGGCAGCGAGGTGACAGGATCGGTAACTTGGACCCTAAGGATCCACGGACTGATC
ATTTACTTTCCGCTGTTCACAAATCCACCTGCTCAAAAAATGTTGAGACTGACAGTCTGCCTCGCTGTGG
TGACCCTGGTTATTTGCTCGCAGTGCCCCGATAATGAAGTCTGTGAAGCAGGCCAGTCCTGCTGCCAGGA
TCCCACTGGTGGCTTCAGCTGCTGCCCTTTCCATCATGGAGAGTGTTGTGAAGACCATCTGCACTGCTGT
CCCGAAGGCATGTTGTGCAGTGTGAAGGACTTAACATGTACAAACGCAACACATACAGAGCCATTGGCGG
ACAGGACACAAGCTAAAAAGCCAGACCTTCCCAAATCATTCAGAATGATCTTCTCCATGCCTGCAAGTGA
AAGCGACATCAGCTGCCCTGACGGCTCTTCCTGTCCTGCTGAGTTCTCCTGTCTGCTGATGTCTACATCA
TATGGCTGCTGTCCAGTAGCACAGGGCCTTGCATGTTCTGATGGGAAACACTGCTGCCCAAATGACCATG
AATGCAGTTCTGACAGCAGCTTGTGTGTCAAACGGAAAGTAAAAGTTGAGACTGTTCTTTGTGGAAATGG
GACGTCGGAGTGTCCTGCAGACACTACATGTTGTCAGGCTGAAGATGGCCTATGGGGGTGCTGCCCCATG
CCAAAGGCTGTATGCTGTGATGACAAAATCCATTGCTGCCCAGAGGACACTGTTTGTGACGTCAAAGCCT
TGAAATGCATATCCTCAACCAACCAGGAGCTGCCCATGTGGGACAAATTCCCTGCTCGCCTTAGGGCTGA
ATGGGAAGATCACAAACAAAAAAAGCCTGAAACTCAACGCACTACAACTAGACCTACAGGCACTACAAGC
ACTAATACAGCTGCCAACCAAATGACTACGCTGCCTGCCGAACACCAAGCGGTGTCTTCAGATGTTCCCT
GTAACGACACTGCGGCCTGTGCTGATGGAACCACATGCTGTAAGACTAAAGATGGAGGATGGGCCTGCTG
TCCTCTGCCTGAGGCCGTGTGTTGTGAAGACTTCATCCACTGCTGTCCTCATGGTAAGAAATGTGATGTA
GCTGCAGGATCCTGTGATGACCCTTCAGGCTCTGTGCCCTGGGTGGAGAAGGTGCCTGTCCGTCCAATCA
AAAAACAGAAAGTGGCGGTTACACAAGTTTCTTCACTGTCTTCAGATGTTCCCTGTAACGACACTGCGGC
CTGTGCTGATGGAACCACATGCTGTAAGACTAAAGATGGAGATTGGGCCTGCTGTCCTCTGCCTGAGGCC
GTGTGTTGTGAAGACTTTGTCCATTGCTGTCCTAAGGGCAAGAAATGTAACATTGCTGCTATGAAATGTG
AAGACCCTTCATGTACCGGAGAGCCCTTGGTAAAACAGACGCCTGTGCAATCAACCACGACTCCCAATGT
GATTGGCAAACAGAAGTCTAATGTTCCCTGTAACGACACTGCGGCCTGTGCTGATGGAACCACATGCTGT
AAGACTAAAGATGGAGATTGGGCCTGCTGTCCTCTGCCTGAGGCTGTGTGTTGTGAAGACTTCATCCACT
GCTGTCCTCATGGTAAGAAATGTGATTTAGCTGCGGGGTCCTGTGATGACCCTTCAGGCTCTGTGCCCTG
GGTGGAGAAGGTGCCCGTCCGTCCAATCAAAAAACAGAAAGTGGCTGTTACAAAAGTTTCTTCAGTGTCT
TCAGATGTTCCCTGTAACGACACTGCGGCCTGTGCTGATGGAACCACATGCTGTAAGACTAAAGAAGGAG
GATGGGCCTGCTGTCCTCTGCCTGAGGCCGTGTGTTGTGAAGACTTCATCCACTGCTGTCCTCATGGTAA
GAAATGTAACGTAGCTGCGGGGTCCTGTGATGACCCTTCAGGCTCTGTGCCCTGGGTGGAGAAGGTGCCC

GTCCATCTTAGAGCAGGTCAGAGGTCATCTGGGAAAGTGAAATGCAACGCTACTCATGGTTGTCCTGAAT
CTAGTACATGCTGTAAGAACATTGCTGGTGAATGGGGCTGCTGTCCTTTCTCTCAGGCTGTATGTTGCAC
AGATGGAGAGCACTGCTGTCCGGCCCACTATAAGTGTAACCTGAGCAGTGTGTCTTGTATTAAGGGAGAC
GTGGTGATCCCCTGGTACAATAAAATCGCTGCTGAAAGCACACCAGCTCCAAAGTTGGATCTCGGCGTTG
TTAAATGCGATGAACAGTCGAGTTGCTCTGCAGATTCGACCTGCTGCCTCTTGTCTAAAGAAGAAACGGG
CTGCTGCCCTTTTCCTGAGGCTGTTTGCTGCCCAGACCAGAAGCACTGCTGTCCTGAGGGCTACAGATGT
GACCTGCGCAGACGCTCCTGTGTAAAGACCACTCGGCTGTACGTGGAAATTACTCAACTCACTCACATCC
GCAGCAACAAGCCCCAGCCAAGTGTTATAGTGAAGGACGTTCAGTGTGGTGGTGGATTCAGCTGTCATGA
TGGTGAGACCTGCTGTCCAACCTCACAAACCACATGGGGATGTTGCCCGTCTCCAAAGGCGGTGTGCTGT
GATGATATGCAACACTGCTGTCCCGCGGGGTATAAGTGTGGGCCGGGTGGCACCTGTATTTCAGCCGGAG
ACTTGGACTGGAGCAACTGGGTCAACTGGAAGTTGTTCTTCTCCAAAAAGAAACGAGCCCTAACTCTATA
AACATCACAGCAGCACATTGATTTTTACCAAAGACACTTACATGCTCAAGTGTCTGATATTTTTTTCACA
TTTTACATTCCTCAATGTACTAATCAGTGATAATCAGATGATTAATTTCACATGGTTGATTTTATTTGCA
GAAGAAAATGTTTAATAAGGACTCATTTTGGTAAGTTTCCATTGTCTAAAAGGTAATACCAACTACGCTT
TAGTCAGAAAGTATGTGTCATTTTTGTAAAGTGTGTTCATAAAATGACCATCGTTACTGCTTAGTGCTGT
CTGAGGATTTATTTATTTCCTAAATGGTGTAAAGATGTAACATATTAAAGCGTAGATGACCTCTAGTGGC
ATATCTTTCATTATAACTCGCATGTGGAAATGTCAGGTTTATAGGTTACATTATTTCAGACTTGACATCA
TGCAGTCTTGTGATTATGACTAGATCACCCTTGACAAGGAAATGTTTAAATGAAGATCTACCAGATATC
CTCTTTTTAATTTCCCTTGGTCTTTACTCTACTTTTGTGTGTAACTTTTTTTCTTCATTAATTTAAAGGG
TTTTAATACTATATTGATTGAGCTTTGTGAAAGATATCTTAAAGATTTCTCAGCATTTCTGTATTGAATA
TTTCTCACAGCGCATCTGAAACCTTGTCAGCTGTAAAAGAGAAATAAAAAAAGAAATGCTTGTGTGTTTT
CTTTTGTATCTCCTGTTCACTTTTTCCAATTTCATTTGTATTTGAGCATTTAGCAGACTAAAATCAGTGG
AATCCAGTATTGTTTTGAACACAAATGACCTTTCATTAAACTGAAATAAACATTCTTCTGAATTTCTAAA
AAAAAAAAA

Genomic data:

Note: Genomic data (exons). Exons from the genomic reference assembly sequence are presented here. They are for the maximum transcript.

From NCBI: NC_007114.3 GI:189908168

Note: Genomic reference assembly sequence. Also found in grna ENSDARG00000004954 Chromosome 3: 30, 065, 546-30, 092, 476 reverse strand.

Coding exon 1 **Exon type: sn**

Note: from start codon

ATGTTGAGACTGACAGTCTGCCTCGCTGTGGTGACCCTGGTTATTTGCTCGCAGTGCCCCAATAATGAAG
TCTGTGAAGCAGGCCAGTCCTGCTGCCAGGATCCCACTGGTGGCTTCAGCTGCTGCCCTTTCCATCAT

Coding exon 2 **Exon type: c**

GGAGAATGTTGTGAGGACCATCTGCACTGCTGTCCTGAAGGCATGTTGTGCAGTGTTAAGGACTTAACAT
GTACAAACGCAACACATACACAGCCATTGGCAGACAGGACACTAGCTAAAAAGCCAGACCTTCCCAAA

Coding exon 3 **Exon type: n***

Note: n* = encodes N-half of a 10 cys module

TCATTCAGAATGATCTTCTCAATGCCTGCAAGTGAAAGCGACATCACCTGTCCTGACGGCTCCTCCTGTC
CTGCTGAGTTCTCTTGTCTGCTGATGTCTACATCATACGGCTGCTGTCCAGTAGCACAG

Coding exon 4 **Exon type: c***

Note: c* = encodes C-half of a 10 cys module

GGCCTTGCATGTTCGGATGGGAAACACTGCTGCCCAAATGACCATGAATGCAGTACTGACAGCAGCTTGT
GTGTCAAGCGAAAAG

Coding exon 5 **Exon type: n**

TAAAAGTTCAGACTGTTCTTTGTGGAAACGGGACATCGGAGTGTCCTGCAGATACTACATGTTGTCAGGC
TGATGATGGCCAATGGGGTTGCTGCCCCATGCCAAAG

Coding exon 6 **Exon type: c**

GCTGTATGCTGTGATGACAAAATCCACTGCTGCCCAGAGGACACTGTTTGTGACGTCAAAGCCTTGAAAT
GCGTATCCTCAACCAACCAGGAGCTGCCCATGTGGGACAAATTCCCAGCTCGCCTTAGGGCTGAATGGGA
AGATCACAAAC

Coding exon 7 **Exon type: j**

Note: j = encoding only more inter-module joining sequence.
AAAAAAGGCCTGAAACTCAACGCACTACAGCTAGACCTACAGGCACTACAAGCACTACTACTGCAGCTAA
CCAAATGACTACGCTGCCTGCCGAACACCAAGGGG

Coding exon 8 **Exon type: n**
TGTCTTCAGATGTTGCTTGTAACGACACTGCGGCCTGTCCTGATGGAAGCACTTGCTGTAAGACTAAAGA
TGGAGGATGGGCCTGCTGTCCTCTGCCTGAG

Coding exon 9 **Exon type: c**
GCCGTGTGTTGTGAAGACTTCATCCACTGCTGTCCTCATGGTAAGAAATGTGACGTAGCTGCGGGGTCCT
GTGAAGACCCTTCAGGCTCTGTGTCCTGGGTGGAGAAGGTTCCTGTCCGTCCTATTAGAAAACAGAAAGT
GGCTGTTACACAAG

Coding exon 10 **Exon type: n**
TCTCTTCAGTGTCTTCAGATGTTCCCTGTAACGACACTGCTGCCTGTGCTGATGGAACCACTTGCTGTAA
GACTAAAGAAGGAGATTGGGCCTGCTGTCCTCTGCCTGAG

Coding exon 11 **Exon type: c**
GCCGTGTGCTGTGAAGACTTTGTCCATTGCTGTCCTAAGGGCAAGAAATGTAACATTGCTGCTATGAAAT
GTGAAGACCCTTTATGTACCGAAGAGCCCTTGGTAAAACAGACACCTGTGCAATCAACTACTACTCCCAA
TGTGATTGGCACACAGA

Coding exon 12 **Exon type: n**
AGTCTGACGTTCCCTGTAACGACACTGCAGCCTGTCCTGATGGAAGCACATGCTGTAAGACTAAAGATGG
AGGATGGGCCTGCTGTCCTCTGCCTGAG

Coding exon 13 **Exon type: c**
GCCGTGTGTTGTGAAGACTTCATCCACTGCTGTCCTCATGGCAAGAAATGTGACGTAGCTGCGGGGTCCT
GTGAAGACCCTTCAGGCTCTGTGCCCTGGGTGGAGAAGGTGCCCGTCCGTCCAATCAGGAAACAGAAAGT
GGCTGTTACAACAG

Coding exon 14 **Exon type: n**
TCTCTCCAGTGTCTTCAGATATTGCCTGTAACGACACTGTGGCCTGTCCTGATGGAAGCACCTGCTGTAA
GGATAAAGATGGAGAATGGGGTTGCTGTCCTCTGCCTGAG

Coding exon 15 **Exon type: c**
GCCGTGTGTTGTGAAGACTTCATCCACTGCTGTCCTCATGGTAAGAAATGTAATGTTGCTGCGGGGTCCT
GTGATGACCCTTCAGGCTCTGTGCCCTGGATGGACAAGGTGCCCGTCCGTCCAATCAAAAAACAGAAAGT
GGCTGTTACACAAG

Coding exon 16 **Exon type: n**
CCTCATCAGCATCTTCAGATGTCCCCTGTAACGACACTGCGGCCTGTCCTGATGGAAGCACATGCTGTAA
AACTAAAGATGGAGGATGGGCCTGCTGTCCTCTGCCTGAG

Coding exon 17 **Exon type: c**
GCCGTGTGTTGTGAAGACTTCATCCACTGCTGTCCTCATGGCAAGAAATGTGACGTAGCTGCGGGGTCCT
GTGAAGACTCTTCAGGCTCTGTGCCATGGGTGGAGAAGGTGCCCGTCCGTCCAATCAGAAAACAGAAAGT
GGCTAATACAAAAG

Coding exon 18 **Exon type: n**
TCTCTCCAGTGTCTTCAGATGTTGCCTGTAACGACACTGTGGCCTGTCCTGATGGAAGCACCTGCTGTAA
GGATAAAGATGGAGATTGGGGCTGCTGTCCTCTGCCTGAA

Coding exon 19 **Exon type: cn**
GCCGTGTGTTGTGAAGACTTCATCCACTGCTGTCCTCATGGTAAGAAATGTAACGTTGCTGCGGGGTCCT
GTGATGACCCTTCAGGCTCTGTGCCCTGGGTGGAGAAGGTCCCCGTCCATCTTAGAGCAGGTCAGAGGCC
ATCTGGCAAAGTGAAATGCAACGCTACTCATGGTTGCCCTGAATCCAGCACTTGCTGTAAGAACATTGCT
GGAGAATGGGGCTGCTGTCCTTTCTCTCAG

Coding exon 20 **Exon type: cn**
GCTGTATGTTGCACAGATGGAGAGCACTGCTGTCCGGCCCACTATAAGTGTAACCTGAGCAGTGTGTCTT
GTATTAAGGGAGACGTGGTGATCCCCTGGTACAATAAAATCGCTGCTGAAAGCACACCAGCTCCAAAGTT

GGATCTCGGCGTTGTTAAATGCGATGAACAGTCGAGTTGCTCTGCAGATTCGACCTGCTGCCTCTTGTCT
AAAGACGAAACGGGCTGCTGCCCTTTTCCTGAG

Coding exon 21 **Exon type: cn**
GCTGTTTGCTGCCCAGACCAGAAGCACTGCTGTCCTGAGGGCTACAGATGTGACCTGCGCAGACGCTCCT
GTGTAAAGACCACTAGGCTGTACGTGGAAATTACTCAACTCACTCACATCCGCAGCAACAAGCCCCAGCC
AAGTGTTGTAGTGAAGGACGTTCAGTGTGGTGGTGGATTCAGCTGTCATGATGGTGAGACCTGCTGTCTA
ACCTCACAAACCACATGGGGATGTTGCCCTTCTCCAAAG

Coding exon 22 **Exon type: c**
Note: up to the stop codon
GCGGTGTGCTGTGATGATATGCAACACTGCTGTCCCGCGGGGTATAAGTGTGGGCCGGGTGGCACCTGTA
TTTCAGCCGGAGACTTGGACTGGAGCAACTGGGTCAACTGGAAGTTGTTCTTCTCCAAAAAGAAACGAGC
CCTAACTCTATAA

Genomic sequence note: A genomic sequence is not presented (see note 2 in header).

00017 **Danio rerio progranulin B** Short name: **D_rerB or DrB** Date entered: 2014-03

Species: ***Danio rerio (zebrafish)***
Taxonomy (via NCBI): *Eukaryota; Metazoa; Chordata; Craniata; Vertebrata; Euteleostomi; Actinopterygii; Neopterygii; Teleostei; Ostariophysi; Cypriniformes; Cyprinidae; Danio.*
General Comment: Good cDNA and genomic data.

Protein Sequence
MVRAAFIALLCVCVNACTALICPDGGMCEDENTCCLTPSGGYGCCPLPHAECCSDHLHCCYQGTLCDLEH
SKCVNKTHVLDWVEKVEAKLQAVVCPDGESECPDDTTCCQMPDGGWGCCPMKNAVCCDDRKHCCPQGTTC
DLVHSMCVSATYGSSPFLRKFAARRRKPLEKNAVDLPAEVNNIREVICPDKISKCPEDTTCCLLETGSYG
CCPMPKAVCCSDQKHCCPEGTTCDLIHSTCLSANGVSEMAIKIPAVTVLKPKEEVVPCNETVACSSGTTC
CKTPEGSWACCPLPKAVCCEDHIHCCPEGTLCNVAASSCDDPTELSVSVPWMEKVSTKPIAPPPNKKCDE
SSSCPGESTCCKLSSGDWGCCPLPEAVCCEDHVHCCPHGSVCNVAAETCETVSDSALRISVPMVKKIPAV
SVPSQKQNCDETSSCPTGTTCCKLTSGSWACCPVPQAVCCADQEHCCPQGYTCDLAQSSCVRSGLPSMAW
FRKEPALRETQRVEDRHMCDAHTSCPRDDTCCFINRIGKWGCCPLPKAVCCKDGDHCCPSGYTCNEEKTS
CTKGLHQIPWFTKKTARVWKSSDELLGHEDVKCDSSTSCPSGSTCCILPTGQWGCCPLVKAVCCEDHEHC
CPQGYICKLELGTCEKASADLSVSLTAVQMPEIQCDTFTRCAHTQSCCRLADSTWACCPYTQAVCCKDMK
HCCPMGYKCDPKVQGCTKSSSSTWWNNSL*

Transcript:
The cDNA data define the transcript well (below), so no predicted sequence is required.

cDNA data:
From: NCBI: NM_212738.1 GI:47086568
Note: This is the cDNA sequence used in our analyses. Two splice variants are reported in Ensembl. We found two EST contigs differing only in 4 codons.
CACTTTACAGCACACCGTTTTACACGGATTTCTTTACCGACTTCGTTAAATACGCACAAGAAAGTCGTTC
ATGGCTTTTCATCTGTAAATTTGAAGCACATTATTTTTGTTGGCCAACATTCACACAATGGTGCGTGCAG
CTTTCATAGCCTTATTATGCGTGTGTGTGAACGCATGCACTGCTCTGATCTGTCCGGATGGAGGGATGTG
TGAAGATGAAAACACCTGCTGTCTAACGCCGTCAGGAGGATACGGCTGCTGCCCACTTCCCCATGCTGAA
TGCTGCTCCGATCACTTGCACTGCTGTTATCAGGGAACTTTGTGCGACCTGGAACATTCCAAGTGTGTGA
ATAAGACGCATGTTTTAGACTGGGTGGAAAAAGTCGAGGCCAAACTGCAAGCGGTAGTTTGTCCGGATGG
AGAGTCTGAATGCCCTGATGATACCACCTGCTGCCAGATGCCTGATGGGGGCTGGGGCTGCTGTCCTATG
AAAAATGCCGTGTGCTGTGATGATAGGAAACACTGCTGTCCTCAAGGAACCACATGTGACCTTGTGCATT
CAATGTGTGTGTCGGCCACGTATGGCTCGTCCCCTTTCTTAAGGAAATTTGCTGCTCGTCGCAGGAAGCC
ATTAGAGAAAAACGCAGTCGATCTGCCGGCAGAGGTCAACAATATTAGAGAAGTGATCTGTCCAGATAAG
ATTTCCAAATGTCCAGAAGATACAACATGCTGTTTACTAGAGACCGGTAGTTACGGATGCTGTCCAATGC
CAAAGGCTGTGTGTTGTTCTGATCAAAAGCACTGCTGTCCAGAGGGCACGACCTGTGACCTCATCCATAG
TACGTGTTTGTCAGCAAATGGAGTCTCAGAAATGGCCATTAAGATCCCAGCTGTCACTGTTCTGAAACCC
AAAGAAGAAGTAGTTCCCTGTAATGAAACCGTGGCGTGTTCTAGTGGCACCACCTGCTGTAAAACGCCAG
AAGGCTCCTGGGCTTGCTGTCCTTTACCAAAGGCAGTGTGTTGTGAGGATCACATTCACTGTTGTCCAGA

AGGAACGTTGTGTAATGTTGCGGCCAGCTCCTGTGATGACCCCACAGAGCTTTCTGTGTCTGTGCCCTGG
ATGGAGAAGGTGTCCACCAAACCCATCGCTCCTCCTCCTAATAAGAAATGTGACGAAAGCTCATCGTGTC
CTGGAGAATCCACCTGCTGCAAACTCTCATCTGGAGACTGGGGCTGCTGCCCACTGCCTGAGGCAGTGTG
TTGTGAGGACCATGTCCACTGTTGTCCCCATGGCTCAGTGTGTAATGTGGCTGCTGAAACCTGTGAAACT
GTCAGTGATTCGGCTCTTCGTATTTCTGTGCCAATGGTGAAGAAGATCCCTGCAGTGTCTGTGCCATCAC
AGAAACAAAACTGTGATGAAACGTCTTCGTGTCCAACCGGGACCACCTGCTGTAAACTGACCTCCGGCTC
CTGGGCCTGCTGTCCAGTGCCACAGGCCGTGTGCTGTGCGGATCAGGAGCACTGCTGTCCACAGGGATAC
ACGTGTGATTTGGCTCAGAGTAGCTGTGTGCGTTCTGGCCTTCCTAGCATGGCATGGTTCAGGAAGGAGC
CTGCGCTGAGAGAAACCCAGCGGGTAGAGGACAGACACATGTGTGACGCTCACACCAGCTGCCCCAGAGA
CGACACCTGCTGCTTCATCAACCGCATCGGCAAGTGGGGCTGCTGTCCACTGCCAAAGGCGGTGTGCTGT
AAGGATGGAGATCACTGCTGTCCAAGCGGCTACACCTGTAACGAGGAGAAAACCTCCTGCACCAAAGGCC
TACATCAGATCCCTTGGTTCACGAAAAAAACGGCAAGAGTCTGGAAGAGTTCAGACGAGCTGCTCGGACA
TGAAGATGTGAAGTGTGACTCCAGCACCAGCTGCCCGTCTGGCTCCACCTGCTGTATTTTACCCACAGGA
CAGTGGGGATGCTGCCCTCTAGTCAAGGCCGTGTGCTGTGAGGATCATGAGCACTGCTGTCCACAGGGCT
ACATCTGTAAGCTGGAGTTAGGGACGTGTGAGAAAGCGTCTGCGGATCTCAGTGTTTCTCTGACTGCAGT
TCAGATGCCAGAGATTCAGTGCGACACCTTCACTCGCTGCGCACACACGCAGAGCTGCTGCAGATTAGCA
GACTCCACGTGGGCCTGCTGTCCTTATACACAGGCTGTGTGCTGTAAAGACATGAAGCACTGCTGCCCAA
TGGGATACAAATGTGACCCAAAAGTCCAAGGTTGCACTAAATCATCCTCATCCACGTGGTGGAATAATTC
TCTCTAACTGAAAAAAATAGACTGACACTGAACCACAAAATATCACACACACACACACACACACACACTC
AAGCATAAAGCATAGTTATTTTTCAGGTACCTGCTTTGGAAAAGGGACTATGCCTCATCTTTCTCTTTGT
AATATTTAGTTTTTATTGAAATACAACATATAGCAGGCTTTATGATTAAGCAGGACACACACTGAGAGAC
TTTTATAGTCCATGAAACTGACTACAAAGCAAGAGCATTGTTCAGTGTCGATGCACACATTAAAAGCTGC
AAGTTTTCTAGTTATCCAGAGTGAATGTGAATGTGATTTTTTTTATTTTTTTTTATAGCAAAATTGTAGT
TCCCGGTCCCCTGAAACATAAAAAATTATCATTAAAGGGTAAATGACTAGGGTAATGACATCCTGGAGTC
CATCGTGTGTTCGTAAGATGATGATTTTCTTGTATTTGTATTTATGATGCTGATTAAAACCCAGTGAACC
CATGAAAAAAAAAAAAAAAA

Genomic data:

Note: Genomic data (exons).
From NCBI: NC_007135.3 GI:189908166 complement(32279693..32306979)
Note: Also Ensemble grnb ENSDARG00000025081 Chromosome 24: 37, 836, 254-37, 861, 603 forward strand.
No exon sequence to show (or no genomic sequence entry).

Coding exon 1 **Exon type: sn**
Note: All but the first codon for the signal peptide and an N-half module.
GTGCGTGCAGCTTTCATAGCCTTATTATGCGTGTGTGTGAACGCATGCACTGCTCTGATCTGTCCGGATG
GAGGGATGTGTGAAGATGAAAACACCTGCTGTCTAACGCCGTCAGGAGGATACGGCTGCTGCCCACTTCC
CCAT

Coding exon 2 **Exon type: c**
GCTGAATGCTGCTCCGATCACTTGCACTGCTGTTATCAGGGAACTTTGTGCGACCTGGAACATTCCAAGT
GTGTGAATAAGACGCATGTTTTAGACTGGGTGGAAAAAGTCGAGGCCAAACTG

Coding exon 3 **Exon type: n**
CAAGCGGTAGTTTGTCCGGATGGAGAGTCTGAATGCCCTGATGATACCACCTGCTGCCAGATGCCTGATG
GGGGCTGGGGCTGCTGTCCTATGAAAAAT

Coding exon 4 **Exon type: c**
GCCGTGTGCTGTGATGATAGGAAACACTGCTGTCCTCAAGGAACCACATGTGACCTTGTGCATTCAATGT
GTGTGTCGGCCACGTATGGCTCGTCCCCTTTCTTAAGGAAATTTGCTGCTCGTCGCAGGAAGCCATTAGA
GAAAAACGCAG

Coding exon 5 **Exon type: j**
Note: j = encoding only more inter-module joining sequence.
TCGATCTGCCGGCAGAGGTCAACAATATTAGAGAAG

Coding exon 6 **Exon type: n**
TGATCTGTCCAGATAAGATTTCCAAATGTCCAGAAGATACAACATGCTGTTTACTAGAGACCGGTAGTTA
CGGATGCTGTCCAATGCCAAAG

Coding exon 7 **Exon type: c**

GCTGTGTGTTGTTCTGATCAAAAGCACTGCTGTCCAGAGGGCACGACCTGTGACCTCATCCATAGTACGT
GTTTGTCAGCAAATGGAGTCTCAGAAATGGCCATTAAGATCCCAGCTGTCACTGTTCTGAAACCCAAAG

Coding exon 8 **Exon type: n**
AAGAAGTAGTTCCCTGTAATGAAACCGTGGCGTGTTCTAGTGGCACCACCTGCTGTAAAACGCCAGAAGG
CTCCTGGGCTTGCTGTCCTTTACCAAAG

Coding exon 9 **Exon type: cn**
GCAGTGTGTTGTGAGGATCACATTCACTGTTGTCCAGAAGGAACGTTGTGTAATGTTGCGGCCAGCTCCT
GTGATGACCCCACAGAGCTTTCTGTGTCTGTGCCCTGGATGGAGAAGGTGTCCACCAAACCCATCGCTCC
TCCTCCTAATAAGAAATGTGACGAAAGCTCATCGTGTCCTGGAGAATCCACCTGCTGCAAACTCTCATCT
GGAGACTGGGGCTGCTGCCCACTGCCTGAG

Coding exon 10 **Exon type: cn**
GCAGTGTGTTGTGAGGACCATGTCCACTGTTGTCCCCATGGCTCAGTGTGTAATGTGGCTGCTGAAACCT
GTGAAACTGTCAGTGATTCGGCTCTTCGTATTTCTGTGCCAATGGTGAAGAAGATCCCTGCAGTGTCTGT
GCCATCACAGAAACAAAACTGTGATGAAACGTCTTCGTGTCCAACCGGGACCACCTGCTGTAAACTGACC
TCCGGCTCCTGGGCCTGCTGTCCAGTGCCACAG

Coding exon 11 **Exon type: cn**
GCCGTGTGCTGTGCGGATCAGGAGCACTGCTGTCCACAGGGATACACGTGTGATTTGGCTCAGAGTAGCT
GTGTGCGTTCTGGCCTTCCTAGCATGGCATGGTTCAGGAAGGAGCCTGCGCTGAGAGAAACCCAGCGGGT
AGAGGACAGACACATGTGTGACGCTCACACCAGCTGCCCCAGAGACGACACCTGCTGCTTCATCAACCGC
ATCGGCAAGTGGGGCTGCTGTCCACTGCCAAAG

Coding exon 12 **Exon type: cn**
GCGGTGTGCTGTAAGGATGGAGATCACTGCTGTCCAAGCGGCTACACCTGTAACGAGGAGAAAACCTCCT
GCACCAAAGGCCTACATCAGATCCCTTGGTTCACGAAAAAAACGGCAAGAGTCTGGAAGAGTTCAGACGA
GCTGCTCGGACATGAAGATGTGAAGTGTGACTCCAGCACCAGCTGCCCGTCTGGCTCCACCTGCTGTATT
TTACCCACAGGACAGTGGGGATGCTGCCCTCTAGTCAAG

Coding exon 13 **Exon type: cn**
GCCGTGTGCTGTGAGGATCATGAGCACTGCTGTCCACAGGGCTACATCTGTAAGCTGGAGTTAGGGACGT
GTGAGAAAGCGTCTGCGGATCTCAGTGTTTCTCTGACTGCAGTTCAGATGCCAGAGATTCAGTGCGACAC
CTTCACTCGCTGCGCACACACGCAGAGCTGCTGCAGATTAGCAGACTCCACGTGGGCCTGCTGTCCTTAT
ACACAG

Coding exon 14 **Exon type: c**
Note: up to the stop codon
GCTGTGTGCTGTAAAGACATGAAGCACTGCTGCCCAATGGGATACAAATGTGACCCAAAAGTCCAAGGTT
GCACTAAATCATCCTCATCCACGTGGTGGAATAATTCTCTCTAA

Genomic sequence note: A genomic sequence is not presented (see note 2 in header).

00020 **G. aculeatus progranulin A** Short name: **G_acuA or GaA** Date entered: 2014-03

Species: ***Gasterosteus aculeatus (stickelback)***
Taxonomy (via NCBI): *Eukaryota; Metazoa; Chordata; Craniata; Vertebrata; Euteleostomi; Actinopterygii; Neopterygii; Teleostei; Euteleostei; Neoteleostei; Acanthomorpha; Acanthopterygii; Percomorpha; Gasterosteiformes; Gasterosteidae; Gasterosteus.*
General Comment:

Protein Sequence
MMQMQVVICLALVALVGADECPDGGRCEDGHTCCNDPANGYECCPLDQAECCGDHAHCCPAGTLCHAATS
SCLNTTGSVPWVERSPADQPGLSKSFRMIKSSAGEEDDNICPDLSRCPAEFSCLKALTKFGCCPLAQGVP
CSDGKHCCPEGHQCSADCRSCIKQELVTTVLCKDGVSECPDGTTCCENPDGKWACCPLPKAVCCEDKTHC
CPEGTTCDVEHSKCISPFTKQELPMWAKSPARLRADWENPKEQVTSKTAVEVTTVTAVSPTEEEPSLTTP
AADAMFPMHLGVSVACDATEACAGNSTCCMTPEGGWSCCPLPEAVDCEDSVHCCPKGRKCNPATQACDSE
GRSVPWLQKVPTIPRLQVGNVSCDATHQCPDGTTCCKTATQEWACCPLPEAVCCDDHEHCCPTGTTCDLA
SLSCTGASGSTPMMPKTPAFVTAGPFPQSPATITKGEEATKAPEEDEVVQCDSRTSCPQSNTCCFMAESQ
KWGCCPLPKAVCCSDGNHCCPTDYTCDVEKTTCTKGEVVIPWYTKLPASASVEDDPASVQCDGQEQCPDD

NTCCQLQSGEWGCCPMPDAVCCPDKVHCCPQGYTCNMASNSCQKLLMLQLQAVPLTRVYLPEAEPTSSPS
EQGDVVCDDQTRCPDGQTCCRTSATTWGCCPAPNAVCCSDMQHCCPEGHTCTETGGCTGNNVPHWHKWQV
FFSNKKRSLIK*

Transcript:

Note: Good EST data.

The cDNA data define the transcript well (below), so no predicted sequence is required.

cDNA data:

From: NCBI est: cDNA EST contig from GI:(83710864, 83710064, 76124045, 83707146, 85458543, 85438873, 76037415, 85423576, 83710293, 76124044, 83710863, 83707145, 83761577, 85423573, 83711476, 31437663, 31421034)

Note: A cDNA contig assembled with Cap3. The EST list for the contig gives a sufficient subset of sequences which define the transcript.

cagctcctcagccttctactatGATGCAGATGCAGGTGGTGATCTGTTTGGCTCTCGTGGCTCTCGTTGG
GGCTGATGAGTGTCCGGATGGAGGGAGATGTGAAGACGGTCACACCTGCTGCAACGACCCGGCCAACGGC
TACGAATGCTGCCCTCTGGATCAGGCCGAGTGCTGCGGGGATCACGCGCACTGCTGTCCTGCCGGAACAC
TCTGCCACGCAGCCACGTCCAGCTGTTTGAACACCACCGGCTCCGTCCCCTGGGTGGAAAGATCCCCCGC
GGATCAGCCCGGACTCTCCAAGTCCTTCAGGATGATCAAGTCGTCCGCGGGGGAGGAGGACGACAACATC
TGTCCCGATCTGTCCCGATGCCCGGCCGAGTTTTCCTGCCTGAAGGCTTTGACAAAGTTTGGCTGCTGTC
CGTTAGCTCAGGGAGTCCCCTGCTCCGATGGGAAACACTGCTGTCCTGAGGGCCACCAGTGCAGCGCAGA
CTGCCGCTCCTGCATCAAACAAGAGCTTGTGACTACAGTTCTGTGCAAGGATGGAGTGTCAGAGTGTCCG
GATGGAACCACCTGCTGTGAAAACCCAGATGGAAAGTGGGCCTGCTGTCCCCTGCCGAAGGCCGTGTGCT
GCGAGGACAAGACGCACTGCTGCCCTGAAGGGACCACGTGTGATGTCGAACACAGCAAATGTATTTCCCC
GTTTACCAAACAGGAGTTGCCTATGTGGGCCAAATCGCCTGCCAGGTTGAGAGCGGACTGGGAGAACCCG
AAAGAACAAGTCACTTCAAAAACGGCCGTTGAAGTCACCACAGTGACTGCAGTCAGTCCCACCGAGGAAG
AACCGTCGCTCACCACACCCGCTGCAGACGCGATGTTTCCCATGCATTTAGGCGTAAGTGTGGCCTGTGA
TGCCACAGAAGCCTGCGCAGGTAACAGCACATGCTGTATGACCCCGGAGGGCGGCTGGAGCTGCTGCCCA
CTTCCAGAGGCTGTTGACTGTGAGGATTCAGTTCACTGCTGCCCAAAAGGGCGAAAATGCAACCCGGCCA
CCCAGGCCTGCGATAGCGAGGGACGCTCTGTGCCGTGGCTCCAGAAGGTGCCCACAATCCCCAGACTACA
GGTGGGGAATGTGTCATGTGACGCCACCCACCAGTGTCCCGACGGCACCACCTGCTGCAAGACTGCAACA
CAGGAGTGGGCCTGCTGTCCTCTGCCCGAGGCCGTCTGCTGCGACGACCACGAGCACTGCTGCCCGACCG
GCACCACCTGCGACCTGGCCTCGCTCAGCTGTACCGGCGCCTCGGGCTCCACGCCCATGATGCCCAAGAC
GCCCGCGTTCGTCACGGCGGGCCCTTTCCCCCAAAGTCCGGCAACGATCACCAAGGGAGAGGAGGCGACG
AAAGCGCCGGAGGAGGACGAGGTGGTTCAGTGCGACTCCCGCACCAGCTGCCCTCAGTCCAACACCTGCT
GCTTCATGGCCGAGTCTCAGAAGTGGGGTTGTTGCCCGCTGCCGAAGGCCGTTTGCTGCTCCGACGGGAA
CCACTGCTGTCCCACGGACTACACGTGTGACGTGGAGAAGACCACGTGCACCAAAGGGGAAGTGGTGATC
CCCTGGTACACCAAGCTCCCGGCCAGCGCCAGCGTGGAGGACGATCCCGCCTCTGTGCAGTGCGACGGGC
AGGAGCAATGCCCCGACGACAACACCTGCTGCCAGCTGCAGTCGGGCGAGTGGGGCTGCTGCCCCATGCC
AGACGCCGTGTGCTGCCCGGATAAGGTGCACTGCTGCCCGCAGGGCTACACCTGCAACATGGCCTCCAAC
TCCTGCCAGAAGCTCCTCATGCTGCAGCTGCAAGCCGTCCCGCTGACACGGGTGTATCTGCCCGAGGCGG
AGCCCACGTCGAGTCCCTCGGAGCAGGGCGACGTGGTGTGTGATGATCAAACCAGATGTCCCGATGGACA
GACCTGCTGCAGGACCTCGGCCACGACGTGGGGCTGCTGTCCAGCTCCCAATGCGGTGTGCTGCAGCGAC
ATGCAGCACTGCTGCCCCGAGGGCCACACCTGCACCGAGACCGGAGGCTGCACCGGGAACAACGTCCCCC
ACTGGCACAAGTGGCAGGTGTTCTTCAGCAACAAAAAGAGATCCCTAATTAAGTGAAGACCCTCCACCCC
CCCCGCACTGCCATTTGATTCTGCAACGTGCACTCAATGGAAATGATGTAGAAGGGATTTTATTTGTGGT
GAAGGGGATGCTGAGTGCATTGCACGATGGGTTATGATGTTCTGCAACTACCACTACACGCCACAACATC
AGGGCATTTACCTTTTAACGACTCTTTTTAAAATTCAGCCTTGGTATTCCTTGTTATTGAATATAACTTC
CATCCATTAAATACAATCCACTGGCAATGTGATTCTCCTTTTGTGTGTTCTGCTTCAGCTGCTTTGTACT
GCCGGGCACTTCATCAATAATTAGTCACAATTCAGGGCATGATTTTATCATTTTAATTATATGTGATTCC
ACCTCATGATGGGCGAGTCCTGTTTAATGCTGTTTTCTACTACTTCAGATTTGGAAGATAATGGAATAAT
CTGTAATGAGTTATTGTTTTGATTTTGTGCATGTAGTTGTCATCCATGTTAATTTGGGATTTCAAACACA
CAATGACGAAAAAACGCAAGTACACTTGAGGCTTTATCTGCATG

Genomic data:

Note: Genomic data (exons).

From NCBI: AANH01011296.1 GI:86291392 (reverse complement 39340-46200)

Coding exon 1 **Exon type:** **sn**

Note: from start codon.

ATGCAGATGCAGGTGGTGATCTGTTTGGCTCTCGTGGCTCTCGTTGGGGCTGATGAGTGTCCGGATGGAG
GGAGATGTGAAGACGGTCACACCTGCTGCAACGACCCGGCCAACGGCTACGAATGCTGCCCTCTGGATCA
G

Coding exon 2 **Exon type: c**
GCCGAGTGCTGCGGGGATCACGCGCACTGCTGTCCTGCCGGAACACTCTGCCACGCAGCCACGTCCAGCT
GTTTGAACACCACCGGCTCCGTCCCCTGGGTGGAAAGATCCCCCGCGGATCAGCCCGGACTCTCCAAG

Coding exon 3 **Exon type: n***
Note: encodes the N-half of a 10 Cys module.
TCCTTCAGGATGATCAAGTCGTCCGCGGGGGAGGAGGACGACAACATCTGTCCCGATCTGTCCCGATGCC
CGGCCGAGTTTTCCTGCCTGAAGGCTTTGACAAAGTTTGGCTGCTGTCCGTTAGCTCAG

Coding exon 4 **Exon type: c***
Note: encodes the C-half of a 10 Cys module.
GGAGTCCCCTGTTCCGATGGGAAACACTGCTGTCCTGAGGGCCACCAGTGCAGCGCAGACTGCCGCTCCT
GCATCAAACAAG

Coding exon 5 **Exon type: n**
AGCTTGTGACTACAGTTCTGTGCAAGGATGGAGTGTCAGAGTGTCCGGATGGAACCACCTGCTGTGAAAA
CCCAGATGGAAAGTGGGCCTGCTGTCCCCTGCCGAAG

Coding exon 6 **Exon type: c**
GCCGTGTGCTGCGAGGACAAGACGCACTGCTGCCCTGAAGGGACCACGTGTGATGTCGAACACAGCAAAT
GTATTTCCCTGTTTACCAAACAGGAGTTGCCTATGTGGGCCAAATCGCCTGCCAGGTTGAGAGCGGACTG
GGAGAACCCGAAAG

Coding exon 7 **Exon type: j**
Note: j = encodes additional joining sequence between modules.
AACAAGTCACTTCAAAAACGGCCGTTGAAGTCACCACAGTGACTGCAGTCAGTCCCACCGAGGAAGAACC
GTCGCTCACCAAGCCCGCTGCAGACGCGATGTTTCCCATGCATTTAG

Coding exon 8 **Exon type: n**
GCGTTAGTGTGGCCTGTGATGCCACAGAAGCCTGCGCAGGTAACAGCACATGCTGTATGACCCCGGAGGG
CGGCTGGAGCTGCTGCCCACTTCCAGAG

Coding exon 9 **Exon type: c*n**
Note: c* = encodes a C-half missing the first Cys as in a 10 Cys module.
GCTGTTGACTGTGAGGATTCAGTTCACTGCTGCCCAAAAGGGCGAAAATGCAACCCGGCCACCCAGGCCT
GCGATAGCGAGGGATGCTCTGTGCCGTGGCTCCAGAAGGTGCCCACAATCCCCAGACTACAGGTGGGGAA
TGTGTCATGTGACGCCACCCACCAGTGTTCCGACGGCACCACCTGCTGCAAGACTGCAACACAGGAGTGG
GCCTGCTGTCCTCTTCCCGAG

Coding exon 10 **Exon type: cn**
GCCGTCTGCTGCGACGACCACGAGCACTGCTGCCCGACCGGCACCACCTGCGACCTGGCCTCGCTCAGCT
GTACCGGCGCCTCGGGCTCCACGCCCATGATGCCCAAGACGCCCGCGTTCGTCACGGCGGGCCCTTTCCC
CCAAAGCCGGGCAACGATCACCAAGGGAGAGGAGGCGACGAAAGCGCCGGAGGAGGACGAGGTGGTTCAG
TGCGACTCCCGCACCAGCTGCCCTCAGTCCAACACCTGCTGCTTCATGGCCGAGTCTCAGAAGTGGGGTT
GTTGCCCGCTGCCGAAG

Coding exon 11 **Exon type: cn**
GCCGTTTGCTGCTCCGACGGGAACCACTGCTGTCCCACGGACTACACGTGTGACGTGGAGAAGACCACGT
GCACCAAAGGGGAAGTGGTGATCCCCTGGTACACCAAGCTCCCGGCCAGCGCCAGCGTGGAGGACGATCC
CGCCTCTGTGCAGTGCGACGGGCAGGAGCAATGCCCCGACGACAACACCTGCTGCCAGCTGCAGTCGGGC
GAGTGGGGCTGCTGCCCCATGCCAGAC

Coding exon 12 **Exon type: cn**
GCCGTGTGCTGCCCGGATAAGGTGCACTGCTGCCCGCAGGGCTACACCTGCAACATGGCCTCCAACTCCT
GCCAGAAGCTCCTCATGCTGCAGCTGCAAGCCGTCCCGCTGACACGGGTGTATCTGCCCGAGGCGGAGCC
CACGTCGAGTCCCTCGGAGCAGGGCGACGTGGTGTGTGATGATCAAACCAGCTGTCCCGATGGACAGACC
TGCTGCAGGACCTCGGCCACGACGTGGGGCTGCTGTCCAGCTCCCAAT

Coding exon 13 **Exon type: c**
GCGGTGTGCTGCAGCGACATGCAGCACTGCTGCCCCGAGGGCCACACCTGCACCGAGACCGGAGGCTGCA
CCGGGAACAACGTCCCCCACTGGCACAAGTGGCAGGTGTTCTTCAGCAACAAAAAGAGATCCCTAATTAA
GTGA

Genomic sequence note: A genomic sequence is not presented (see note 2 in header).

00021 **G. aculeatus progranulin B** Short name: **G_acuB or GaB** Date entered: 2014-03

Species: ***Gasterosteus aculeatus (stickelback)***
Taxonomy (via NCBI): *Eukaryota; Metazoa; Chordata; Craniata; Vertebrata; Euteleostomi; Actinopterygii; Neopterygii; Teleostei; Euteleostei; Neoteleostei; Acanthomorpha; Acanthopterygii; Percomorpha; Gasterosteiformes; Gasterosteidae; Gasterosteus.*
General Comment:

Protein Sequence

MTLKMLPMGVMFLALLGLSCTRVCPDGGICRHQNTCCKNAVGGYGCCPLPYAECCSDHLHCCYERTVCDL
VHSRCVNKTVSLPMMRRVPQLQETLRAIICPDQESECPNDATCCQLLDGSWGCCPLTKAVCCEDKLHCCP
EATKCDLAHSKCVSSSLESFPMLEKLPAKRRDNHSVGSVSCPGGKSSCPDSYTCCLLASGDYGCCPYSQA
MCCSDHLHCCPSNTICDLAHGVCKDGEAIFPLLKKIAAVPNDVTCPDETSSCPDETTCCKMVDGSYGCCP
MPDAVCCSDHIHCCPEGTQCDVAHSACLSARGAAALTAVAVAMPTEVQKKVSVLPCNDSVACAEGSTCCG
LAEGGWACCPLPKAVCCEDHQHCCPHGTVCDLEASTCVDSSAGTSTPWFDKSPAFSLPTGNSQCDKTTSC
PGTSTCCRAVGGGWACCPLPQAVCCEDHLHCCPHDTVCNLQAETCDDPSGVLPSLRWVEKASATRAVAMP
TLPARNMCDAQTSCPRDTTCCFMDQTRKWGCCPLPKAVCCHDGDHCCPSGHTCEPHRSSCSKGPLVLVPW
FSKLSARSEAAGNASATDVKCDNKSSCASGTTCCKLKTEGWGCCPLVKAVCCADREHCCPQGYTCNMQTS
TCEKTANALLPPLPQVRVVRPEPVDHEDVACDVGGEFRCPGRATCCRVSASEWGCCPSPQAVCCPDSKHC
CPAGYSCDPKAGGCSRPQLTWDGKSDFVPHGL*

Transcript:
Note: A full cDNA transcript sequence is available.
The cDNA data define the transcript well (below), so no predicted sequence is required.

cDNA data:
From: NCBI: BT028434.1 GI:112421697
Note: cDNA sequence data:
CGCGTCGAATTGGACGGTTTTCCCATTTGCGGCGAACCCATCAGGTTGCAAGAGGAGAATGCAGCATAGA
GCTGTTCATTAATAACACCTGAACTGAAGCTGACTGCAAAGCCTCTGCTATGACGTTAAAGATGTTGCCG
ATGGGGGTCATGTTCCTGGCTCTGCTGGGCCTGAGCTGCACCAGGGTCTGTCCGGATGGAGGCATCTGCC
GCCACCAGAACACCTGCTGCAAGAACGCCGTTGGAGGCTACGGATGCTGTCCGCTACCATATGCGGAGTG
TTGCTCAGACCACCTGCACTGCTGCTACGAGAGGACGGTGTGTGATCTGGTCCACAGCCGATGTGTGAAT
AAGACCGTTTCCCTTCCCATGATGAGACGCGTCCCTCAGCTTCAAGAGACGCTGAGAGCCATCATCTGTC
CAGACCAAGAGTCCGAGTGTCCGAACGATGCCACCTGCTGCCAGCTTCTGGACGGCTCGTGGGGCTGCTG
CCCATTAACTAAGGCGGTGTGTTGTGAGGATAAACTCCACTGCTGCCCCGAGGCGACAAAGTGCGACCTC
GCTCATTCAAAGTGTGTTTCCTCCTCGCTGGAGTCCTTCCCCATGCTGGAGAAGCTGCCTGCAAAGAGGA
GGGACAATCACTCAGTCGGCTCGGTGTCGTGTCCCGGGGGCAAAAGCAGCTGCCCAGACAGTTATACGTG
TTGCCTTCTGGCCAGTGGAGATTACGGCTGCTGTCCTTACTCACAGGCCATGTGCTGCAGCGACCATCTC
CACTGTTGCCCCAGCAACACGATATGTGACCTGGCCCACGGGGTCTGCAAAGACGGGGAGGCGATATTTC
CGCTGCTGAAGAAGATCGCCGCTGTGCCCAATGACGTTACGTGTCCAGACGAGACGTCGTCGTGTCCAGA
CGAGACGACGTGCTGCAAGATGGTGGACGGCTCGTACGGCTGCTGTCCGATGCCCGACGCCGTCTGTTGC
TCAGATCACATCCACTGTTGTCCTGAAGGAACCCAGTGTGACGTCGCCCACAGCGCATGTTTATCCGCCC
GTGGAGCGGCTGCCTTAACGGCCGTCGCCGTGGCAATGCCGACGGAAGTACAGAAAAAAGTCAGCGTCCT
TCCCTGCAACGACTCTGTTGCGTGTGCTGAGGGGAGCACCTGCTGCGGGTTGGCGGAGGGAGGATGGGCC
TGTTGTCCACTACCCAAGGCCGTATGCTGCGAGGACCACCAGCACTGCTGTCCACACGGCACTGTTTGTG
ACCTCGAGGCCTCCACCTGCGTCGACTCCTCTGCCGGCACGTCCACGCCGTGGTTCGACAAATCGCCCGC
CTTCTCTCTACCGACCGGCAACAGCCAGTGTGACAAGACCACCTCGTGTCCCGGGACGTCCACCTGCTGC
AGGGCGGTGGGCGGCGGCTGGGCCTGCTGTCCTCTGCCTCAGGCCGTGTGCTGCGAGGACCACCTCCACT
GCTGCCCTCACGACACCGTCTGCAACCTGCAGGCCGAGACCTGCGACGACCCCTCTGGCGTCCTGCCGTC
CCTCCGCTGGGTGGAGAAAGCGAGCGCCACTCGGGCCGTTGCCATGCCGACTCTTCCGGCCAGGAACATG
TGCGACGCCCAGACCAGCTGCCCCAGAGACACAACCTGCTGCTTCATGGACCAGACACGCAAATGGGGGT

GTTGCCCTTTGCCGAAGGCTGTGTGCTGCCACGACGGAGACCACTGCTGCCCCAGCGGCCACACCTGTGA
GCCCCACCGCTCCTCCTGCTCGAAGGGACCCCTTGTCCTTGTGCCCTGGTTCTCCAAGCTGAGCGCCCGC
AGCGAGGCGGCGGGGAACGCCTCTGCGACGGACGTCAAGTGCGACAACAAGAGCAGCTGCGCCTCGGGGA
CGACGTGCTGCAAGCTGAAGACGGAGGGGTGGGGCTGCTGCCCGCTGGTGAAGGCCGTTTGCTGTGCCGA
CCGCGAGCACTGCTGTCCTCAGGGTTACACCTGCAACATGCAGACGAGCACCTGCGAGAAGACCGCCAAC
GCCCTCCTCCCCCCCCTCCCTCAGGTCAGAGTGGTACGGCCCGAGCCCGTAGACCACGAGGATGTGGCGT
GTGACGTCGGGGGGGAGTTCCGCTGCCCCGGACGGGCCACGTGCTGCAGGGTCTCTGCATCGGAGTGGGG
TTGCTGCCCCTCCCCGCAGGCCGTCTGCTGCCCTGACTCAAAGCACTGCTGCCCCGCCGGCTACTCATGT
GACCCGAAGGCCGGCGGTTGCTCCCGCCCCCAGCTGACCTGGGACGGAAAGAGTGACTTTGTCCCGCATG
GACTTTGATCCCACCTAAAGGTGCACCAGTCCTTTTCATCCATAACACCCACTTTAGTCTGTGTCCTCTT
TAGTCACTTCCTCTTTTTGTCCTTCATATGAGGGGGGGGAGCTGATGAAACGTGGTGGCAATGAAGACAT
GATAATGAAGTAGAAGCACTTTGACCAGGTGACATCGCTGTTTATTCTTTATTATCATGATCTCAGTTTG
CTGCAGATGATGAGTGACTATGTGGGTGTTGCGTCATGGCTTTCTTCATTTTAACAAATGTAGAGACAAA
AACATCAATGCGTTCCTTTGTCATGTTGGTTTCATGTATTTTTTGTAACTGTTTCAAACCGCTGGAGGAG
CAAGTTGGACAATGAGCAAAGGAATTACTTTTTAGTGTGTTTCATTGGGTTTTATGAACAGATGAATAAG
TTGTTAAACTGGTTACCTTCATGGTCTTAATGGACACGATGTTAAATAAAGTTGTATAATTAAAAAAAAA
AAAAAAAAAAAAAAAAAA

Genomic data:
Note: Genomic data (exons).
From NCBI: AANH01006432.1 GI:86296256 (94690-102950)
Note: The gene is within the sequence gb|AANH01006432.1|:94690-102950 Gasterosteus aculeatus cont1.006431, whole genome shotgun sequence
Coding exon 1 **Exon type: sn**
Note: from the start codon (which begins this exon)
ATGTTGCCGATGGGGGTCGTGTTCCTGGCTCTGCTGGGCCTGAGCTGCACCAGGGTCTGTCCGGATGGAG
GCATCTGCCGGCACCAGAACACCTGCTGCAAGAACGCCGTTGGAGGCTACGGGTGCTGTCCGCTACCATA
T

Coding exon 2 **Exon type: c**
GCGGAGTGTTGCTCAGACCACCTGCACTGCTGCTACGAGAGGACGGTGTGTGATCTGGTCCACAGCCGAT
GTGTGAATAAGACCGTTTCCCTTCCCATGATGAGACGCGTCCCTCAG

Coding exon 3 **Exon type: n**
CTTCAAGAGACGCTGAGAGCCATCATCTGTCCAGACCAAGAGTCCGAGTGTCCGAACGATGCCACCTGCT
GCCAGCTTCTGGACGGCTCGTGGGGCTGCTGCCCATTAACTAAG

Coding exon 4 **Exon type: c**
GCGGTGTGTTGTGAGGATAAACTCCACTGCTGCCCCGAGGCGACAAAGTGCGACCTCGCTCATTCAAAGT
GTGTTTCCTCCTCGCTGGAGTCCTTCCCCATGCTGGAGAAGCTGCCTGCAAAGAGGAGGGACAATCACTC
AG

Coding exon 5 **Exon type: n**
TCGGCTCGGTGTCGTGTCCCGGGGGCAAAAGCAGCTGCCCAGACAGTTATACGTGTTGCCTTCTGGCCAG
TGGAGATTACGGCTGCTGTCCTTACTCACAG

Coding exon 6 **Exon type: c**
GCCATGTGCTGCAGCGACCATCTCCACTGTTGCCCCAGCAACACGATATGTGACCTGGCCCACGGGGTCT
GCAAAGACGGGGAGGCGATATTTCCGCTGCTGAAGAAGATCGCCGCTGTGCCCAATGACG

Coding exon 7 **Exon type: n**
TTACGTGTCCAGACGAGACGTCGTCGTGTCCAGACGAGACGACGTGCTGCAAGATGGTGGACGGCTCGTA
CGGCTGCTGTCCGATGCCCGAC

Coding exon 8 **Exon type: c**
GCCGTCTGTTGCTCAGATCACATCCACTGTTGTCCTGAAGGAACCCAGTGTGACGTCGCCCACAGCGCAT
GTTTATCCGCCCGTGGAGCGGCTGCCTTAACGGCCGTCGCCGTGGCAATGCCGACGGAAGTACAGAAAAA
AG

Coding exon 9 **Exon type: n**
TCAGCGTCCTTCCCTGCAACGACTCTGTTGCGTGTGCTGAGGGGAGCACCTGCTGCGGGTTGGTGGAGGG

AGGATGGGCCTGTTGTCCACTACCCAAG

Coding exon 10 Exon type: cn
GCCGTATGCTGCGAGGACCACCAGCACTGCTGTCCACACGGCACTGTTTGTGACCTCGAGGCCTCCACCT
GCGTCGACTCCTCTGCCGGCACGTCCACGCCGTGGTTCGACAAATCGCCCGCCTTCTCTCTACCGACCGG
CAACAGCCAGTGTGACAAGACCACCTCATGTCCCGGGACGTCCACCTGCTGCAGGGCGGTGGGCGGCGGC
TGGGCCTGCTGTCCTCTGCCTCAG

Coding exon 11 Exon type: cn
GCCGTGTGCTGCGAGGACCACCTCCACTGCTGCCCTCACGACACCGTCTGCAACCTGCAGGCCGAGACCT
GCGACGACCCCTCGGGTGTCCTGCCGTCCCTCCGCTGGGTGGAGAAAGCGAGCGCCACTCGGGCCGTTGC
CATGCCGACTCTTCCGGCCAGGAACATGTGCGACGCCCAGACCAGCTGCCCCAGAGACACAACCTGCTGC
TTCATGGACCAGACACGCAAATGGGGGTGTTGCCCTTTGCCGAAG

Coding exon 12 Exon type: cn
GCTGTGTGCTGCCACGACGGAGACCACTGCTGCCCCAGCGGCCACACCTGTGAGCCCCACCGCTCCTCCT
GCTCGAAGGGACCCCTTGTCCTTGTGCCCTGGTTCTCCAAGCTGAGCGCCCGCAGCGAGGCGGGGGGGAA
CGCCTCCGCGACGGACGTCAAGTGCGACAACAAGAGCAGCTGCGCCTCGGGGACGACGTGCTGCAAGCTG
AAGACGGAGGGGTGGGGCTGCTGCCCGCTGGTGAAG

Coding exon 13 Exon type: cn
GCCGTTTGCTGTGCCGACCGCGAGCACTGCTGTCCTCAGGGTTACACCTGCAACATGCAGACGAGCACCT
GCGAGAAGACCGCCGACGCCCTCCTCCCCCCCCTCCCTCAGGTCAGAGTGGTACGGCCCGAGCCCGTAGA
CCACGAGGACGTGGCGTGTGACGTCGGGGGGGAGTTCCGCTGCCCCGGACGGGCCACGTGCTGCAGGGTC
TCTGCATCGGAGTGGGGCTGCTGCCCCTCCCCGCAG

Coding exon 14 Exon type: c
Note: up to stop codon
GCCGTCTGCTGCCCTGACTCAAAGCACTGCTGCCCCGCCGGCTACTCATGTGACCCGAAGGCCGGCGGTT
GCTCCCGCCCCCAGCTGACCTGGGACGGAAAGAGTGACTTTGTCCCGCATGGACTTTGA

Genomic sequence note: A genomic sequence is not presented (see note 2 in header).

00022 **G. aculeatus progranulin C** Short name: **G_acuC or GaC** Date entered: 2014-03

Species: ***Gasterosteus aculeatus (stickelback)***
Taxonomy (via NCBI): *Eukaryota; Metazoa; Chordata; Craniata; Vertebrata; Euteleostomi; Actinopterygii; Neopterygii; Teleostei; Euteleostei; Neoteleostei; Acanthomorpha; Acanthopterygii; Percomorpha; Gasterosteiformes; Gasterosteidae; Gasterosteus.*
General Comment:

Protein Sequence
MLGVTLWLLGVFVCASCAVTCPDGTTCSNLNTCCMTKRGYSCCHYPNAVCCSDLAHCCPSGFRCNTSTQM
CERKPWTMITPMVKKQAAEKSDTLVLPVSPLREPVKNQVADPKTSSVVHCDNYYTCPDATTCCRHPAGTW
FCCPYSPGRCCLDGFHCCPYGYDCDLTYTHCVRRGLRYPFSPNKSVPSVPASLILPSEEKSRLQETPMTA
LTEANGVVAVDGVIRCDSSFYCAAGTTCCKAPTGRWNCCPYPLGQCCADGRHCCQYGYACDSTSLSCKSG
YSQIPSGAQEAAKTD*

Transcript:
Note: Transcript cDNA contig from meagre EST data
The cDNA data define the transcript well (below), so no predicted sequence is required.

cDNA data:
From: NCBI est: EST contig from gi: (62076839, 85405602, 62076838, 85405603)
Note: The cDNA EST contig required some manual assembly.
GCGGTGAGCTGTTGAATCACTTGACCTGCAAGATGTTGGGAGTGACTCTGTGGTTGTTGGGAGTATTTGT
GTGTGCCTCCTGTGCCGTCACATGCCCGGATGGGACCACGTGTTCTAATCTCAACACCTGCTGTATGACT
AAACGCGGCTACAGCTGCTGTCATTACCCAAACGCCGTGTGTTGCTCTGACTTGGCCCACTGCTGCCCTT
CAGGGTTCCGCTGTAACACGTCTACCCAGATGTGTGAGCGAAAGCCGTGGACGATGATTACACCCATGGT
GAAGAAGCAGGCTGCAGAGAAATCTGACACTCTTGTTCTACCGGTTTCTCCCCTTCGGGAGCCCGTGAAG

AACCAGGTGGCAGACCCGAAAACGAGCTCAGTTGTTCACTGTGACAACTATTACACGTGTCCTGATGCCA
CTACTTGCTGCAGACACCCAGCGGGTACTTGGTTCTGTTGCCCGTACTCTCCTGGAAGGTGTTGTCTGGA
TGGCTTCCACTGTTGCCCTTATGGCTATGACTGTGACCTCACGTACACGCACTGTGTGAGACGAGGCCTG
CGGTATCCTTTCAGCCCCAACAAAAGTGTGCCATCAGTGCCGGCCTCTCTCATTTTGCCTTCAGAGGAAA
AAAGCCGCTTGCAAGAGACACCCATGACTGCTCTGACAGAAGCAAATGGTGTCGTCGCCGTGGATGGCGT
CATTCGCTGCGATTCATCGTTCTACTGCGCAGCAGGAACAACTTGCTGCAAAGCACCTACAGGCCGATGG
AACTGCTGTCCCTACCCACTGGGCCAGTGTTGTGCAGATGGGCGACACTGCTGTCAGTATGGTTATGCAT
GTGACTCCACCTCCCTGTCATGTAAAAGCGGCTACTCTCAGATCCCATCAGGAGCCCAAGAAGCCGCCAA
AACCGACTGAGGAAGTTATTTTATTTTTTTCTGCACCGGGACAGCAGTCTAATGAATGATGTACTTAATA
ACATAGATCTCATGGG

Genomic data:

Note: Genomic data (exons).
From NCBI: AANH01002322.1 GI:86300366 (81340-83840)
Note: gb|AANH01002322.1|:81340-83840 Gasterosteus aculeatus cont1.002321, whole genome shotgun sequence
Coding exon 1 **Exon type: sn**
Note: from start codon (which is at the beginning of this exon)
ATGTTGGGAGTGACTCTGTGGTTGTTGGGAGTATTTGTGTGTGCCTCCTGTGCCGTCACATGCCCGGATG
GGACCACGTGTTCTAATCTCAACACCTGCTGTATGACTAAACGCGGCTACAGCTGCTGTCATTACCCAAA
C

Coding exon 2 **Exon type: cn**
GCCGTGTGTTGCTCTGACTTGGCCCACTGCTGCCCTTCAGGGTTCCGCTGTAACACGTCTACCCAGATGT
GTGAGCGAAAGCCGTGGACGATGATTACACCCATGGTGAAGAAGCAGGCTGCAGAGAAATCTGACACTCT
TGTTCTACCGGTTTCTCCCCTTCGGGAGCCCGTGAAGAACCAGGTGGCAGACCCGAAAACGAGCTCAGTT
GTTCACTGTGACAACTATTACACGTGTCCTGATGCCACTACTTGCTGCAGACACCCAGCGGGTACTTGGT
TCTGTTGCCCGTACTCTCCT

Coding exon 3 **Exon type: c**
GGAAGGTGTTGTCTGGATGGCTTCCACTGTTGCCCTTATGGCTATGACTGTGACCTCACGTACACGCACT
GTGTGAGACGAGGCCTGCGGTATCCTTTCAGCCCCAACAAAAGTGTGCCATCAGTGCCGGCCTCTCTCAT
TTTGCCTTCAGAGGAAAAAAGCCGCTTGCAAGAG

Coding exon 4 **Exon type: n**
ACACCCATGACTGCTCTGACAGAAGCAAATGGTGTCGTCGCCGTGGATGGCGTCATTCGCTGCGATTCAT
CGTTCTACTGCGCAGCAGGAACAACTTGCTGCAAAGCACCTACAGGCCGATGGAACTGCTGTCCCTACCC
ACTG

Coding exon 5 **Exon type: c**
Note: up to the stop codon.
GGCCAGTGTTGTGCAGATGGGCGACACTGCTGTCAGTATGGTTATGCATGTGACTCCACCTCCCTGTCAT
GTAAAAGCGGCTACTCTCAGATCCCATCAGGAGCCCAAGAAGCCGCCAAAACCGACTGA

Genomic sequence note: A genomic sequence is not presented (see note 2 in header).

00029 **G. morhua progranulin C** Short name: **G_morC or codC** Date entered: 2014-03

Species: ***Gadus morhua (Atlantic cod)***
Taxonomy (via NCBI): *Eukaryota; Metazoa; Chordata; Craniata; Vertebrata; Euteleostomi; Actinopterygii; Neopterygii; Teleostei; Euteleostei; Neoteleostei; Acanthomorpha; Paracanthopterygii; Gadiformes; Gadidae; Gadus.*
General Comment: This cod progranulin small form sequence was included in the analysis because it it displays another variety branching off from the 3 module form by loss of the third (c) coding exon and replacement with a short unrelated exon, and more recently via a mutation of the second codon of the last (c) coding exon to produce a stop codon. If this stop were suppressed, translation would continue through a nearly typical (except for an extra Cys) C-half module. This fossil C-half last exon was included in our analysis, and the translation is included in lower case below.

Protein Sequence

MWCYSVLVLVGVAVGWSVQVSCYVVCPDGSLCANDNTCCKNIFGGYGCCPFPQAVCCSDMMQCCPNGWDC
HVSTQTCTKEGHPLLRLPMRQKVIAGGPGGSLSTLPLSRLQGQRADLGQGGAEGPGGRQVDCGEEDDGSC

PSGTECCRGADGGWLCCPVSAQDLGEALSPRGSAVHPATKAWNRILGLRENEMEGSDGMGGVYCDAVSYC
PSGTSCCQGTAHGWSCCPDHMG*ccsdgvhccdcgyacdpsnlrcrkgfadvpsgakkdarpy*

Transcript:

Note: Well defined by 46 ESTs (e.g. gi: 295336340 gb: GW859673.1)
The cDNA data define the transcript well (below), so no predicted sequence is required.

cDNA data:

From: NCBI: Contig of GW859673.1 GI:295336340 and 45 other EST sequences.
Note: cDNA sequence data:

```
TCGGGGGAATAAATGTGATGCACTGCATCTGATAGGAAGAGATCCAACCACTGCAGGCTGAAAAGATGTG
GTGTTACAGTGTGTTGGTCCTAGTGGGCGTGGCCGTGGGGTGGAGTGTCCAGGTCTCCTGCTACGTCGTC
TGTCCGGATGGATCGCTTTGCGCAAATGACAACACCTGCTGCAAGAACATTTTCGGTGGATACGGCTGCT
GCCCTTTCCCACAGGCGGTGTGCTGCTCCGACATGATGCAATGCTGTCCAAACGGCTGGGATTGTCACGT
CAGCACCCAGACGTGTACGAAGGAGGGCCATCCACTGCTCCGCTTACCCATGAGGCAGAAGGTGATCGCT
GGGGGACCGGGCGGCTCCCTCTCTACCCTTCCCCTCTCACGCCTCCAGGGGCAGCGGGCTGATCTGGGTC
AGGGAGGAGCTGAAGGCCCGGGGGGTCGGCAGGTCGACTGCGGAGAGGAAGACGACGGCTCCTGCCCCTC
GGGCACGGAGTGCTGCAGAGGGGCCGACGGTGGCTGGCTCTGCTGCCCCGTCTCCGCTCAGGACCTCGGG
GAGGCTCTCAGCCCCAGAGGGTCCGCCGTACACCCGGCCACTAAGGCATGGAACAGGATCCTCGGGCTCA
GAGAGAACGAGATGGAAGGGTCCGATGGCATGGGAGGGGTGTACTGTGATGCGGTATCCTACTGCCCATC
GGGGACCTCCTGCTGTCAGGGCACTGCACACGGCTGGAGCTGCTGCCCTGACCACATGGGCTAGTGTTGT
TCTGACGGGGTTCACTGCTGTGATTGTGGCTATGCCTGTGACCCCTCGAACCTGAGATGCAGGAAGGGAT
TCGCTGACGTCCCTTCAGGAGCAAAGAAGGATGCTAGACCATACTGAGACACAAAAGCACATATTCTCAG
CTTCATGTGTGCAGTCTTAAATTCAAATACCAAACCATATAGGCAAAGCCTTATTCTTACATGTTCTGTG
AGATTAATTGTTCCTTATTTGATAGACAATTGTTCAATAAACTGGCATTCTAAAATCAAAAAAAAAAAAA
AAAAAAAAAAAAAAAAAAAAAAAAAAAAAA
```

Genomic data:

Note: Genomic data (exons).
From NCBI: CAEA01374489.1 GI:344526866
Coding exon 1 **Exon type: sn**
Note: from the start codon (which is also the beginning of this exon)

```
ATGTGGTGTTACAGTGTGTTGGTCCTAGTGGGCGTGGCCGTGGGGTGGAGTGTCCAGGTCTCCTGCTACG
TCGTCTGTCCGGATGGATCGCTTTGCGCAAATGACAACACCTGCTGCAAGAACATTTTCGGTGGATACGG
CTGCTGCCCTTTCCCACAG
```

Coding exon 2 **Exon type: cn**

```
GCGGTGTGCTGCTCCGACATGATGCAATGCTGTCCAAACGGCTGGGATTGTCACGTCAGCACCCAGACGT
GTACGAAGGAGGGCCATCCACTGCTCCGCTTACCCATGAGGCAGAAGGTGATCGCTGGGGGACCGGGCGG
CTCCCTCTCTACCCTTCCCCTCTCACGCCTCCAGGGGCAGCGGGCTGATCTGGGTCAGGGAGGAGCTGAA
GGCCCGGGGGGTCGGCAGGTCGACTGCGGAGAGGAAGACGACGGCTCCTGCCCCTCGGGCACGGAGTGCT
GCAGAGGGGCCGACGGTGGCTGGCTCTGCTGCCCCGTCTCCGCT
```

Coding exon 3 **Exon type: x**
Note: Normally, one would expect a c type exon here. This is a short unrelated sequence.

```
CAGGACCTCGGGGAGGCTCTCAGCCCCAGAGGGTCCGCCGTACACCCGGCCACTAAGGCATGGAACAGGA
TCCTCGGGCTCAGAGAG
```

Coding exon 4 **Exon type: n**

```
AACGAGATGGAAGGGTCCGATGGCATGGGAGGGGTGTACTGTGATGCGGTATCCTACTGCCCATCGGGGA
CCTCCTGCTGTCAGGGCACTGCACACGGCTGGAGCTGCTGCCCTGACCACATG
```

Coding exon 5 **Exon type: sup c**
Note: Where sup c means that there is c like coding following a second codon stop mutation. The sequence is given up to the second stop codon (the normal termination).

```
GGCTAGTGTTGTTCTGACGGGGTTCACTGCTGTGATTGTGGCTATGCCTGTGACCCCTCGAACCTGAGAT
GCAGGAAGGGATTCGCTGACGTCCCTTCAGGAGCAAAGAAGGATGCTAGACCATACTGA
```

Genomic sequence note: A genomic sequence is not presented (see note 2 in header).

00003 **Green Anole progranulin** Short name: **A_car or Anole** Date entered: 2014-03

Species: ***Anolis carolinensis (green anole)***
Taxonomy (via NCBI): *Eukaryota; Metazoa; Chordata; Craniata; Vertebrata; Euteleostomi; Lepidosauria; Squamata; Iguania; Iguanidae; Polychrotinae; Anolis.*
General Comment: Well documented genomic sequence. Little cDNA data.

Protein Sequence

```
MWAPLIVYIVFCGTAFSLRCPDGSHCDGSSTCCQRHGEDGYTCCDKPQFVGTSLRMLPPDSVNELSNVVC
PDGSMCPVDYSCLRTVDAAFACCPWKEAISCADGHHCCPLGSHCSAKGDACIQSEVAAPLATFSAVQCPD
HESECPNDSTCCLMPDGSWGCCPMPEASCCSDKIHCCPHGTSCDLAHARCFSSTQEQPLLKKFPAKKRAL
VLSLAQKNICPGKQSSCPDVATCCLLSTSQYGCCPLQNAVCCSDRLHCCPQGTTCDLLHSKCTMTSRWSW
PITRLSVDLQKAHDIQCDLKYSCPSGNTCCKKASGEWACCPLEEAVCCTDNIHCCPKGYQCNLAEGTCEK
GSRSIPWVAKTSTISTAASTDMIVQCDFQTACPDGQTCCRLVSGAWGCCPIPQAVCCQDHIHCCPNGYSC
NPSTGSCFKSGHHLPWLEKKAAISTITSEIQNVRCDDVSSCADGQTCCHTATGGWSCCPLPEAVCCADSL
HCCPSGYLCDVAHGSCVKSQVPSQRLSMTPVLRRQVGEVKCDDQTSCPDGQTCCRLSSGQWGCCPFPRAV
CCADHVHCCPSGFMCDPSSGVCSKSQHTLPWAPKLPAQTQQSQDIRCNDTASCEEGQTCCKGVSGAWSCC
QLPNAVCCEDHQHCCPSGYTCNVAAQSCEKQHKPTVLPSGDLLMSSQVAITTLDVSCGDQHYCHYGQTCC
KAKSGGWACCPYDKGTCCRDELHCCPPGFYCSISGFECYKKWPLRWDAGAFSPRSSQARPLL*
```

Transcript:

Note: Only 2 EST sequences. One confirms the 5' end, but is anomalous, as it also has 3' end sequence and most coding region deleted. The other confirms the 3' end.

Predicted sequence:

Note: Our original manual prediction from genomic data differs from XM_003222517.1, but is supported now by Transcriptome Shotgun Assembly data: e.g. GAFZ01049906.1.
Derivation: Prediction from sequence found in NC_014781 GI:315422580 bases 64130898-64155855

```
atgtgggcaccattgatagtgtatatagtgttctgtgggacggcgttttctctacggtgcccagatggaa
gtcactgtgatggatcatccacttgttgccaacgccacggagaggatgggtacacctgctgtgacaaacc
tcagtttgtggggacctccctgcgcatgttgcccccagacagtgtgaatgagctctccaacgttgtgtgt
cccgatggcagcatgtgtcctgtggactactcctgtttgcgtacggtagatgctgcctttgcctgctgtc
cctggaaagaggccatttcctgtgctgatggacaccattgttgcccctgggctcccactgcagtgcgaa
gggagatgcctgtatacagagtgaagtggcagctcctctggccacgttcagtgctgtccagtgcccagac
catgagtcggagtgcccaaatgattccacgtgttgcctgatgcctgacggatcgtggggctgctgcccaa
tgccagaggcttcctgctgttctgacaagatccactgctgtcctcatggcacaagttgtgatctggctca
tgcgcggtgcttctcatccactcaggagcagcctcttttgaagaagttcccagccaagaaacgagctctg
gtgttaagtctggctcagaaaaatatctgtccagggaaacagtctagctgtcctgacgttgccacctgtt
gccttctctcgaccagccaatatggctgttgccctttacagaacgctgtgtgctgcagcgaccgtcttca
ctgctgtccacaaggcaccacctgtgacctgctgcattcgaaatgcacaatgacctctcgctggtcatgg
cccatcacccgtctctcggtggacctccaaaaagctcatgatattcagtgtgatttaaagtacagttgcc
catctgggaacacttgctgcaagaaagcctcaggggaatgggcgtgctgcccccctggaagaggctgtatg
ctgcacagacaatatccactgctgcccaaagggctaccaatgcaatctggcagaaggcacctgtgagaaa
ggaagcagaagtatcccatgggtggcaaagacaagcacaatatctacagcagcctctactgatatgatcg
tgcaatgtgatttccagacagcctgcccagatggccaaacttgttgccgcctagtatcaggcgcctgggg
ctgctgtccaataccacaggctgtgtgttgtcaagatcacatccattgctgtcccaatggctactcatgt
aatccatcgactggctcttgcttcaagagtggacaccatctcccttggctggagaagaaagctgctatct
caaccatcacctctgagatccagaatgtccggtgtgatgatgtgtcaagttgtgcggatgggcagacatg
ctgccacactgcaacaggtggctggagctgttgccctctgccagaggcagtctgctgcgctgactctttg
cattgctgccccagtgggtacttgtgtgatgttgcacatggctcctgcgtcaaaagccaggtcccctccc
agcgcctctccatgactcctgttctgagaaggcaggttggagaagtgaaatgtgacgaccaaaccagctg
cccagatggacagacatgttgccggctttcctcgggccaatggggttgctgtcctttcccacgggctgtc
tgttgtgcagaccatgtgcattgctgccccagtggcttcatgtgtgatccatcctcaggcgtctgctcga
agtctcagcacactctgccctgggcacccaagcttccagcccagacgcagcaaagccaagacatccgttg
caacgatactgccagctgcgaagaagggcagacctgctgcaagggtgtgtcaggggcttggagctgctgc
caactacccaatgctgtttgctgtgaggatcaccaacactgttgcccctctggctacacctgcaatgttg
cagctcagagttgtgagaaacagcacaagcccacagttctgccttcaggggacctgctgatgtcttccca
agttgccatcaccactttggatgtgtcttgtggtgaccaacactactgtcattatggacagacctgctgc
aaggccaagtcaggcgggtgggcttgttgcccctacgacaagggaacctgctgccgtgacgaactccatt
```

gctgtccacctggtttctattgctccatttcaggatttgaatgttacaagaaatggcccctccgttggga
cgctggtgccttctcccctcgctcttcccaagcgcgcccattgctttga

cDNA data:

From: NCBI: FG711007
Note: Anomalous cDNA sequence. Confirms start and first splice in coding region, but then skips out of frame to the last 5 codons of the last coding exon.

GGCACAGCTACAGTGGGAAAGCCTGGTTTTATATCCAAGATGTGGGCACCATTGATAGTGTATATAGTGT
TCTGTGGGACGGCGTTTTCTCTACGGTGCCCAGATGGAAGTCACTGTGATGGATCATCCACTTGTTGCCA
ACGCCACGGAGAGGATGGGTACACCTGCTGTGACAAACCTCAGTTTGTGGGGACCTCCCTGCGCATGTTG
CCCCCAGACAGTGTGAATGAGCTCTCCATGACTCCCAAGCGCGCCCATTGCTTTGAAGTGCCTTTTTGGG
TGGACTTGCCCCATGTTTGGGGGCTAGAGTGCTTCTTTTCAGGGAGGGGTTTTTCTGGCCCAGAGGGAGT
CCTGGTTTTTTTATACTTGTTGCCTCAGGGGTTGGAAGAGGCTTCTCCTTTCTCCTGCTTTCCGGTATTG
CCTGAATAACAAGCCCACACTCCTGTGGACAGTTCCTCCTTTTCTTGCCTTCTGGCATTTCTGGTTCACT
TCCAAGCCCAGAATCATCCTGGCCGCCTCCTCCAATCTTGAAAGAGTTGTCATGTTTCCTTTCTCTTTGG
AAGATTTATGGGGTTTAGTGTCTTTTAAAAAAAATTGCCATTGCTCTTCCAATATTTTTTAAAGTTTTGT
TTTACTTTCATGCACTTTCTGAAAATAATAATCTGCACACTGGATTAGCAGCTGCCATTTTTCCTGCCAT
GTCTGAGCGTGTAAGGTTCCACATATCGTGTTGCAAGGCTTTTGCTGGAGAGAAAGCCAACCTTTGTCTT
TTTTTGTTATAACACAGAGTTTTTGGGAAAGAACAGGGGTCCATTGC

From: NCBI: FG675846
Note: cDNA sequence has some errors, but confirms last 3 coding exons.

GTGCAGACCATGTGCATTGCTGCCCCAGTGGCTTCATGTGTGATCCATCCTCAGGCGTCTGCTCGAAGTC
TCAGCATACTCTGCCCTGGGCACCCAAGCTTCCAGCCCAGACGCAGCAAAGCCAAGATATCCGTTGCGAC
GATACTGCCGGCTGCGAAGAAGGGCAGACCTGCTGCAAGGGTGTGTCAGGGGCTTGGAGCTGCTGCCAAC
TACCCAATGCTGTTTGCTGTGAGGATCACCAACACAGTTGCCCCTCTGGCTACACCTGCAATGTTGCAGC
TCAGAGTTGTGAGAAATAGCACAAGCCCACAGTTCTGCCTTCAGGGGACCTGCTGATGTCTTCCCAAGTT
GCCATCACCACTTTGGATGTGTCTTGTGGTGACCAACACTACTGTCATTATGGACAGACCTGCTGCAAGG
CCAAGTCAGGCGGGTGGGCTTGTTGCCCCTACGACAAGGGAACCTGCTGCCGTGACGAACTCCATTGCTG
TCCACCTGGTTTCTATTGCTCCATTTCGGGATTTGAATGTTACAAGAAATGGCCCCTCCGTTGGGACGCT
GGTGCCTTCTCCTCTCGCTCTTCCCAAGCGCGCCCATTGCTTTGAAGTGCCTTTTTGGGTGGACTTGCCC
CATGTTTGGGGGCTAGAGTGCTTCTTTCAGGGAGGGGCTTTTCTGGCCCAGAGGGAGTCCTGGTTTTTTT
ATACTTGTTGCCTTAGGGGTTGGAAGAGGCTTCTCCTTTCTCCTGCTTTCAGGTATTGCCTGAATACAGC
CCACACTCCTGT

Genomic data:

Note: Genomic data (exons). Originally deduced from the now obsolete AAWZ01020356. Currently (Feb. 2014) represented in NC_014781 GI:315422580: 64130898-64155855, and as TSA GAFZ01049906.1 GI:463412945
From NCBI: NC_014781 GI:315422580: 64130898-64155855

Coding exon 1 **Exon type: sn**
Note: from start codon

ATGTGGGCACCATTGATAGTGTATATAGTGTTCTGTGGGACGGCGTTTTCTCTACGGTGCCCAGATGGAA
GTCACTGTGATGGATCATCCACTTGTTGCCAACGCCACGGAGAGGATGGGTACACCTGCTGTGACAAACC
TCAG

Coding exon 2 **Exon type: n***
Note: * Encodes the N-half of a 10 cys module

TTTGTGGGGACCTCCCTGCGCATGTTGCCCCCAGACAGTGTGAATGAGCTCTCCAACGTTGTGTGTCCCG
ATGGCAGCATGTGTCCTGTGGACTACTCCTGTTTGCGTACGGTAGATGCTGCCTTTGCCTGCTGTCCCTG
GAAAGAG

Coding exon 3 **Exon type: c***
Note: * Encodes the C-half of a 10 cys module

GCCATTTCCTGTGCTGATGGACACCATTGTTGCCCCCTGGGCTCCCACTGCAGTGCGAAGGGAGATGCCT
GTATACAGAGTGAAG

Coding exon 4 **Exon type: n**

TGGCAGCTCCTCTGGCCACGTTCAGTGCTGTCCAGTGCCCAGACCATGAGTCGGAGTGCCCAAATGATTC
CACGTGTTGCCTGATGCCTGACGGATCGTGGGGCTGCTGCCCAATGCCAGAG

Coding exon 5 Exon type: c
GCTTCCTGCTGTTCTGACAAGATCCACTGCTGTCCTCATGGCACAAGTTGTGATCTGGCTCATGCGCGGT
GCTTCTCATCCACTCAGGAGCAGCCTCTTTTGAAGAAGTTCCCAGCCAAGAAACGAGCTCTGGTGTTAA

Coding exon 6 Exon type: n
GTCTGGCTCAGAAAAATATCTGTCCAGGGAAACAGTCTAGCTGTCCTGACGTTGCCACCTGTTGCCTTCT
CTCGACCAGCCAATATGGCTGTTGCCCTTTACAGAAC

Coding exon 6 Exon type: c
GCTGTGTGCTGCAGCGACCGTCTTCACTGCTGTCCACAAGGCACCACCTGTGACCTGCTGCATTCGAAAT
GCACAATGACCTCTCGCTGGTCATGGCCCATCACCCGTCTCTCGGTGGACCTCCAAAAAG

Coding exon 7 Exon type: n
CTCATGATATTCAGTGTGATTTAAAGTACAGTTGCCCATCTGGGAACACTTGCTGCAAGAAAGCCTCAGG
GGAATGGGCGTGCTGCCCCCTGGAAGAG

Coding exon 8 Exon type: cn
GCTGTATGCTGCACAGACAATATCCACTGCTGCCCAAAGGGCTACCAATGCAATCTGGCAGAAGGCACCT
GTGAGAAAGGAAGCAGAAGTATCCCATGGGTGGCAAAGACAAGCACAATATCTACAGCAGCCTCTACTGA
TATGATCGTGCAATGTGATTTCCAGACAGCCTGCCCAGATGGCCAAACTTGTTGCCGCCTAGTATCAGGC
GCCTGGGGCTGCTGTCCAATACCACAG

Coding exon 9 Exon type: cn
GCTGTGTGTTGTCAAGATCACATCCATTGCTGTCCCAATGGCTACTCATGTAATCCATCGACTGGCTCTT
GCTTCAAGAGTGGACACCATCTCCCTTGGCTGGAGAAGAAAGCTGCTATCTCAACCATCACCTCTGAGAT
CCAGAATGTCCGGTGTGATGATGTGTCAAGTTGTGCGGATGGGCAGACATGCTGCCACACTGCAACAGGT
GGCTGGAGCTGTTGCCCTCTGCCAGAG

Coding exon 10 Exon type: cn
GCAGTCTGCTGCGCTGACTCTTTGCATTGCTGCCCCAGTGGGTACTTGTGTGATGTTGCACATGGCTCCT
GCGTCAAAAGCCAGGTCCCCTCCCAGCGCCTCTCCATGACTCCTGTTCTGAGAAGGCAGGTTGGAGAAGT
GAAATGTGACGACCAAACCAGCTGCCCAGATGGACAGACATGTTGCCGGCTTTCCTCGGGCCAATGGGGT
TGCTGTCCTTTCCCACGG

Coding exon 11 Exon type: cn
GCTGTCTGTTGTGCAGACCATGTGCATTGCTGCCCCAGTGGCTTCATGTGTGATCCATCCTCAGGCGTCT
GCTCGAAGTCTCAGCACACTCTGCCCTGGGCACCCAAGCTTCCAGCCCAGACGCAGCAAAGCCAAGACAT
CCGTTGCAACGATACTGCCAGCTGCGAAGAAGGGCAGACCTGCTGCAAGGGTGTGTCAGGGGCTTGGAGC
TGCTGCCAACTACCCAAT

Coding exon 12 Exon type: cn
GCTGTTTGCTGTGAGGATCACCAACACTGTTGCCCCTCTGGCTACACCTGCAATGTTGCAGCTCAGAGTT
GTGAGAAACAGCACAAGCCCACAGTTCTGCCTTCAGGGGACCTGCTGATGTCTTCCCAAGTTGCCATCAC
CACTTTGGATGTGTCTTGTGGTGACCAACACTACTGTCATTATGGACAGACCTGCTGCAAGGCCAAGTCA
GGCGGGTGGGCTTGTTGCCCCTACGACAAG

Coding exon 13 Exon type: c
Note: up to stop codon
GGAACCTGCTGCCGTGACGAACTCCATTGCTGTCCACCTGGTTTCTATTGCTCCATTTCAGGATTTGAAT
GTTACAAGAAATGGCCCCTCCGTTGGGACGCTGGTGCCTTCTCCCCTCGCTCTTCCCAAGCGCGCCCATT
GCTTTGA

Genomic sequence note: A genomic sequence is not presented (see note 2 in header).

00001 **Human progranulin** Short name: **H_sap or human** Date entered: 2014-03

Species: ***Homo sapiens (human)***
Taxonomy (via NCBI): *Eukaryota; Metazoa; Chordata; Craniata; Vertebrata; Euteleostomi; Mammalia; Eutheria; Euarchontoglires; Primates; Haplorrhini; Catarrhini; Hominidae; Homo*
General Comment: Well documented transcript and genomic sequences

Protein Sequence

MWTLVSWVALTAGLVAGTRCPDGQFCPVACCLDPGGASYSCCRPLLDKWPTTLSRHLGGPCQVDAHCSAG
HSCIFTVSGTSSCCPFPEAVACGDGHHCCPRGFHCSADGRSCFQRSGNNSVGAIQCPDSQFECPDFSTCC
VMVDGSWGCCPMPQASCCEDRVHCCPHGAFCDLVHTRCITPTGTHPLAKKLPAQRTNRAVALSSSVMCPD
ARSRCPDGSTCCELPSGKYGCCPMPNATCCSDHLHCCPQDTVCDLIQSKCLSKENATTDLLTKLPAHTVG
DVKCDMEVSCPDGYTCCRLQSGAWGCCPFTQAVCCEDHIHCCPAGFTCDTQKGTCEQGPHQVPWMEKAPA
HLSLPDPQALKRDVPCDNVSSCPSSDTCCQLTSGEWGCCPIPEAVCCSDHQHCCPQGYTCVAEGQCQRGS
EIVAGLEKMPARRASLSHPRDIGCDQHTSCPVGQTCCPSLGGSWACCQLPHAVCCEDRQHCCPAGYTCNV
KARSCEKEVVSAQPATFLARSPHVGVKDVECGEGHFCHDNQTCCRDNRQGWACCPYRQGVCCADRRHCCP
AGFRCAARGTKCLRREAPRWDAPLRDPALRQLL*

Transcript:

The cDNA data define the transcript well (below), so no predicted sequence is required.

cDNA data:

From: NCBI: NM_002087

Note: cDNA sequence data:

GGCGAGAGGAAGCAGGGAGGAGAGTGATTTGAGTAGAAAAGAAACACAGCATTCCAGGCTGGCCCCACCT
CTATATTGATAAGTAGCCAATGGGAGCGGGTAGCCCTGATCCCTGGCCAATGGAAACTGAGGTAGGCGGG
TCATCGCGCTGGGGTCTGTAGTCTGAGCGCTACCCGGTTGCTGCTGCCCAAGGACCGCGGAGTCGGACGC
AGGCAGACCATGTGGACCCTGGTGAGCTGGGTGGCCTTAACAGCAGGGCTGGTGGCTGGAACGCGGTGCC
CAGATGGTCAGTTCTGCCCTGTGGCCTGCTGCCTGGACCCCGGAGGAGCCAGCTACAGCTGCTGCCGTCC
CCTTCTGGACAAATGGCCCACAACACTGAGCAGGCATCTGGGTGGCCCCTGCCAGGTTGATGCCCACTGC
TCTGCCGGCCACTCCTGCATCTTTACCGTCTCAGGGACTTCCAGTTGCTGCCCCTTCCCAGAGGCCGTGG
CATGCGGGGATGGCCATCACTGCTGCCCACGGGGCTTCCACTGCAGTGCAGACGGGCGATCCTGCTTCCA
AAGATCAGGTAACAACTCCGTGGGTGCCATCCAGTGCCCTGATAGTCAGTTCGAATGCCCGGACTTCTCC
ACGTGCTGTGTTATGGTCGATGGCTCCTGGGGGTGCTGCCCCATGCCCCAGGCTTCCTGCTGTGAAGACA
GGGTGCACTGCTGTCCGCACGGTGCCTTCTGCGACCTGGTTCACACCCGCTGCATCACACCCACGGGCAC
CCACCCCCTGGCAAAGAAGCTCCCTGCCCAGAGGACTAACAGGGCAGTGGCCTTGTCCAGCTCGGTCATG
TGTCCGGACGCACGGTCCCGGTGCCCTGATGGTTCTACCTGCTGTGAGCTGCCCAGTGGGAAGTATGGCT
GCTGCCCAATGCCCAACGCCACCTGCTGCTCCGATCACCTGCACTGCTGCCCCCAAGACACTGTGTGTGA
CCTGATCCAGAGTAAGTGCCTCTCCAAGGAGAACGCTACCACGGACCTCCTCACTAAGCTGCCTGCGCAC
ACAGTGGGGGATGTGAAATGTGACATGGAGGTGAGCTGCCCAGATGGCTATACCTGCTGCCGTCTACAGT
CGGGGGCCTGGGGCTGCTGCCCTTTTACCCAGGCTGTGTGCTGTGAGGACCACATACACTGCTGTCCCGC
GGGGTTTACGTGTGACACGCAGAAGGGTACCTGTGAACAGGGGCCCCACCAGGTGCCCTGGATGGAGAAG
GCCCCAGCTCACCTCAGCCTGCCAGACCCACAAGCCTTGAAGAGAGATGTCCCCTGTGATAATGTCAGCA
GCTGTCCCTCCTCCGATACCTGCTGCCAACTCACGTCTGGGGAGTGGGGCTGCTGTCCAATCCCAGAGGC
TGTCTGCTGCTCGGACCACCAGCACTGCTGCCCCCAGGGCTACACGTGTGTAGCTGAGGGGCAGTGTCAG
CGAGGAAGCGAGATCGTGGCTGGACTGGAGAAGATGCCTGCCCGCCGGGCTTCCTTATCCCACCCCAGAG
ACATCGGCTGTGACCAGCACACCAGCTGCCCGGTGGGGCAGACCTGCTGCCCGAGCCTGGGTGGGAGCTG
GGCCTGCTGCCAGTTGCCCCATGCTGTGTGCTGCGAGGATCGCCAGCACTGCTGCCCGGCTGGCTACACC
TGCAACGTGAAGGCTCGATCCTGCGAGAAGGAAGTGGTCTCTGCCCAGCCTGCCACCTTCCTGGCCCGTA
GCCCTCACGTGGGTGTGAAGGACGTGGAGTGTGGGGAAGGACACTTCTGCCATGATAACCAGACCTGCTG
CCGAGACAACCGACAGGGCTGGGCCTGCTGTCCCTACCGCCAGGGCGTCTGTTGTGCTGATCGGCGCCAC
TGCTGTCCTGCTGGCTTCCGCTGCGCAGCCAGGGGTACCAAGTGTTTGCGCAGGGAGGCCCCGCGCTGGG
ACGCCCCTTTGAGGGACCCAGCCTTGAGACAGCTGCTGTGAGGGACAGTACTGAAGACTCTGCAGCCCTC
GGGACCCCACTCGGAGGGTGCCCTCTGCTCAGGCCTCCCTAGCACCTCCCCCTAACCAAATTCTCCCTGG
ACCCCATTCTGAGCTCCCCATCACCATGGGAGGTGGGGCCTCAATCTAAGGCCTTCCCTGTCAGAAGGGG
GTTGTGGCAAAAGCCACATTACAAGCTGCCATCCCCTCCCCGTTTCAGTGGACCCTGTGGCCAGGTGCTT
TTCCCTATCCACAGGGGTGTTTGTGTGTGTGCGCGTGTGCGTTTCAATAAAGTTTGTACACTTTCAAAAA
AAAAAAAAAAAAA

Genomic data:

Note: Genomic data (exons).

From NCBI: NG_007886

Coding exon 1 **Exon type:** sn

Note: from start codon

ATGTGGACCCTGGTGAGCTGGGTGGCCTTAACAGCAGGGCTGGTGGCTGGAACGCGGTGCCCAGATGGTC
AGTTCTGCCCTGTGGCCTGCTGCCTGGACCCCGGAGGAGCCAGCTACAGCTGCTGCCGTCCCCTTCTG

Coding exon 2 **Exon type: n***
Note: * Encodes the N-half of a 10 cys module
GACAAATGGCCCACAACACTGAGCAGGCATCTGGGTGGCCCCTGCCAGGTTGATGCCCACTGCTCTGCCG
GCCACTCCTGCATCTTTACCGTCTCAGGGACTTCCAGTTGCTGCCCCTTCCCAGAG

Coding exon 3 **Exon type: c***
Note: * Encodes the C-half of a 10 cys module
GCCGTGGCATGCGGGGATGGCCATCACTGCTGCCCACGGGGCTTCCACTGCAGTGCAGACGGGCGATCCT
GCTTCCAAAGATCAG

Coding exon 4 **Exon type: n**
GTAACAACTCCGTGGGTGCCATCCAGTGCCCTGATAGTCAGTTCGAATGCCCGGACTTCTCCACGTGCTG
TGTTATGGTCGATGGCTCCTGGGGGTGCTGCCCCATGCCCCAG

Coding exon 5 **Exon type: c**
GCTTCCTGCTGTGAAGACAGGGTGCACTGCTGTCCGCACGGTGCCTTCTGCGACCTGGTTCACACCCGCT
GCATCACACCCACGGGCACCCACCCCCTGGCAAAGAAGCTCCCTGCCCAGAGGACTAACAGGGCAG

Coding exon 6 **Exon type: n**
TGGCCTTGTCCAGCTCGGTCATGTGTCCGGACGCACGGTCCCGGTGCCCTGATGGTTCTACCTGCTGTGA
GCTGCCCAGTGGGAAGTATGGCTGCTGCCCAATGCCCAAC

Coding exon 7 **Exon type: c**
GCCACCTGCTGCTCCGATCACCTGCACTGCTGCCCCCAAGACACTGTGTGTGACCTGATCCAGAGTAAGT
GCCTCTCCAAGGAGAACGCTACCACGGACCTCCTCACTAAGCTGCCTGCGCACACAG

Coding exon 8 **Exon type: n**
TGGGGGATGTGAAATGTGACATGGAGGTGAGCTGCCCAGATGGCTATACCTGCTGCCGTCTACAGTCGGG
GGCCTGGGGCTGCTGCCCTTTTACCCAG

Coding exon 9 **Exon type: cn**
GCTGTGTGCTGTGAGGACCACATACACTGCTGTCCCGCGGGGTTTACGTGTGACACGCAGAAGGGTACCT
GTGAACAGGGGCCCCACCAGGTGCCCTGGATGGAGAAGGCCCCAGCTCACCTCAGCCTGCCAGACCCACA
AGCCTTGAAGAGAGATGTCCCCTGTGATAATGTCAGCAGCTGTCCCTCCTCCGATACCTGCTGCCAACTC
ACGTCTGGGGAGTGGGGCTGCTGTCCAATCCCAGAG

Coding exon 10 **Exon type: cn**
GCTGTCTGCTGCTCGGACCACCAGCACTGCTGCCCCCAGGGCTACACGTGTGTAGCTGAGGGGCAGTGTC
AGCGAGGAAGCGAGATCGTGGCTGGACTGGAGAAGATGCCTGCCCGCCGGGCTTCCTTATCCCACCCCAG
AGACATCGGCTGTGACCAGCACACCAGCTGCCCGGTGGGGCAGACCTGCTGCCCGAGCCTGGGTGGGAGC
TGGGCCTGCTGCCAGTTGCCCCAT

Coding exon 11 **Exon type: cn**
GCTGTGTGCTGCGAGGATCGCCAGCACTGCTGCCCGGCTGGCTACACCTGCAACGTGAAGGCTCGATCCT
GCGAGAAGGAAGTGGTCTCTGCCCAGCCTGCCACCTTCCTGGCCCGTAGCCCTCACGTGGGTGTGAAGGA
CGTGGAGTGTGGGGAAGGACACTTCTGCCATGATAACCAGACCTGCTGCCGAGACAACCGACAGGGCTGG
GCCTGCTGTCCCTACCGCCAG

Coding exon 12 **Exon type: c**
Note: to stop codon
GGCGTCTGTTGTGCTGATCGGCGCCACTGCTGTCCTGCTGGCTTCCGCTGCGCAGCCAGGGGTACCAAGT
GTTTGCGCAGGGAGGCCCCGCGCTGGGACGCCCCTTTGAGGGACCCAGCCTTGAGACAGCTGCTGTGA

Genomic sequence note: A genomic sequence is not presented (see note 2 in header).

00027 **L. chalumnae progranulin A** Short name: **L_chaA** Date entered: 2014-03

Species: ***Latimeria chalumnae (Coelacanth)***
Taxonomy (via NCBI): *Eukaryota; Metazoa; Chordata; Craniata; Vertebrata; Euteleostomi; Coelacanthiformes; Coelacanthidae; Latimeria.*
General Comment: No cDNA sequences (as of 2014/02). Our prediction differs from XM_005992229.1

GI:556965436. The prediction comes from 4 genomic contigs.

Protein Sequence

MAVCGTLYKFSVLCLWLGLVTALKCPGGLVCDNEDHCCELPGGGYECCPQSKEHMRSFPMVQSGMLNEVT
ENLCLDHTSCPLEYSCMRTPAGSYGCCPVEEAVSCRDGNHCCPKGFLCSADGKSCIKRLDDLNLGAIICP
DETSECPDESTCCQLPSGQWGCCPFSQAVCCSDKLHCCPHGTSCDVAHSQCLKGLSKTPMWSSFPATKRA
HDVKYNATSSCPHLFTSCKGESGEETCCPLSKAVCCEDHTHCCPEGTTCDVKEGKCQHKVLAIPWFTKRP
AGPVKKSLTTEEKSGERDVKCNDTVACPDGSTCCKVQSGDWACCPLAKAVCCEDHIHCCPEGTTCDVKEG
KCQHKVLAIPWFTKRPAGPVKKSLTTEEKSGERDVKCNDTVACPDGSTCCKVQSGDWACCPLAKAVCCED
HTHCCPEGTTCDVKEGKCQHKVLAIPWFTKRPAGPVKKSLTTEEKSGERDVKCNDTVACPDGNTCCKMQS
GDWGCCPAPNAVCCEDHIHCCPEGTTCDAKGEACNQNILVVPWLTKRPATPVKKSRTTEEKSMERDVKCN
DTVACPDGNTCCKMQSGNWGCCPIAEAVCCADHIHCCPQGYTCETETCEKGKLSFPWMQKEPAITNKLPE
EESAPSVMCDDQTECPVGNTCCKYSTGEWGCCPLSQAVCCSDHKHCCPTDYTCDLLHSTCSKGKIAIPWV
SKTVARKMIRTSTDPLTVVTCDSHTRCPDRNTCCKLLTGKWACCPLPNAVCCEDHTHCCPQGYQCNVQAQ
TCESQDGVLFPWNIQKAPVHTFSTSEKNAGNDVKCDSKKSCAPHNTCCKKASGGWGCCLYEEAVCCEDQS
HCCPKGYTCEVSAGLCLKKDGFYWDGLLSNRKRSFSTL*

Transcript:

Note: No cDNA data at this time

Predicted sequence:

Note: Our prediction differs somewhat from XM_005992229.1.

Derivation: Predicted from BAHO01286349.1 GI:405617871, BAHO01286353.1 GI: 405617867, AFYH01038604.1 GI: 346176232, and BAHO01286354.1 GI: 405617866.

ATGGCAGTGTGTGGGACTTTGTACAAGTTTTCAGTGCTATGTCTTTGGCTGGGTCTGGTAACAGCACTGA
AATGTCCTGGTGGATTGGTTTGTGACAATGAAGATCACTGCTGTGAGCTTCCAGGAGGAGGGTATGAGTG
TTGCCCTCAGTCAAAGGAGCATATGAGGTCTTTCCCGATGGTCCAGTCTGGAATGTTGAATGAAGTTACA
GAGAACTTGTGTCTAGATCACACTTCATGCCCTCTGGAGTACTCCTGTATGCGTACACCTGCAGGGTCCT
ATGGCTGCTGTCCTGTAGAGGAGGCTGTATCCTGTCGTGATGGAAATCACTGTTGTCCTAAAGGTTTTCT
TTGCAGTGCAGATGGGAAATCCTGTATAAAACGCTTAGATGACTTGAACTTGGGTGCAATTATTTGTCCA
GATGAAACTTCTGAGTGTCCTGATGAGTCTACCTGCTGCCAGCTACCAAGTGGACAGTGGGGGTGTTGCC
CCTTCTCGCAGGCTGTGTGTTGTTCAGATAAATTGCATTGCTGCCCTCATGGAACATCTTGTGATGTAGC
TCACTCTCAGTGTTTAAAAGGACTCTCAAAGACACCTATGTGGAGCAGTTTTCCAGCAACCAAGAGAGCC
CATGATGTGAAATACAATGCCACGAGCAGCTGTCCCCATCTCTTTACAAGCTGTAAAGGGGAGTCTGGAG
AAGAGACTTGCTGTCCTCTATCAAAGGCTGTGTGCTGTGAAGATCATACCCACTGCTGCCCAGAGGGGAC
CACTTGTGATGTAAAGGAAGGGAAGTGTCAGCACAAAGTCCTGGCTATCCCCTGGTTCACTAAGAGACCA
GCTGGACCAGTGAAGAAGTCTCTGACTACAGAAGAGAAGAGCGGGGAAAGAGACGTGAAATGTAATGATA
CAGTTGCTTGTCCTGATGGGAGCACCTGTTGTAAGGTGCAGTCTGGAGACTGGGCCTGCTGCCCCCTGGC
TAAAGCTGTGTGCTGTGAAGATCATATCCACTGCTGTCCAGAGGGGACCACTTGTGATGTAAAGGAAGGA
AAGTGTCAGCACAAAGTCCTGGCTATCCCCTGGTTCACTAAGAGACCAGCTGGACCAGTGAAGAAGTCTC
TGACTACAGAAGAGAAGAGCGGGGAAAGAGACGTGAAATGTAATGATACAGTTGCTTGTCCTGATGGGAG
CACCTGTTGTAAGGTGCAGTCTGGAGACTGGGCCTGCTGCCCCCTGGCTAAAGCTGTGTGCTGTGAAGAT
CATACCCACTGCTGTCCAGAGGGGACCACTTGTGATGTAAAGGAAGGAAAGTGTCAGCACAAAGTCCTGG
CTATCCCCTGGTTCACTAAGAGACCAGCTGGACCAGTGAAGAAGTCTCTGACTACAGAAGAGAAGAGCGG
GGAAAGAGACGTGAAATGTAATGATACAGTTGCTTGTCCTGATGGGAACACTTGTTGTAAGATGCAGTCT
GGGGACTGGGGCTGCTGTCCTGCGCCTAATGCTGTGTGCTGTGAAGATCATATCCACTGCTGTCCAGAGG
GGACCACTTGTGATGCAAAGGGAGAAGCATGTAACCAGAATATCTTGGTTGTCCCCTGGCTCACTAAGAG
ACCAGCCACACCTGTGAAGAAGTCTCGGACTACAGAAGAGAAGAGCATGGAAAGAGATGTGAAATGTAAT
GATACAGTTGCTTGTCCTGATGGGAACACCTGTTGTAAGATGCAGTCTGGGAACTGGGGCTGCTGCCCCA
TCGCTGAAGCAGTGTGCTGTGCTGATCATATCCACTGCTGTCCACAAGGTTATACATGTGAAACCGAAAC
ATGTGAGAAGGGGAAGCTATCTTTCCCTTGGATGCAGAAGGAGCCAGCTATTACTAACAAGCTACCTGAA
GAAGAAAGTGCTCCCAGTGTAATGTGTGATGATCAAACAGAATGCCCTGTTGGAAATACTTGCTGTAAAT
ATAGCACTGGAGAGTGGGGATGCTGCCCTCTATCTCAGGCTGTTTGCTGCAGTGACCACAAGCATTGTTG
CCCAACTGATTATACTTGTGACCTTTTGCATAGCACATGCTCAAAGGGAAAGATAGCCATTCCTTGGGTC
AGTAAGACCGTGGCGAGAAAAATGATCAGGACTTCTACAGATCCTCTCACTGTTGTGACGTGTGATTCTC
ATACTAGGTGCCCTGATAGAAACACATGCTGTAAGCTTCTCACAGGAAAATGGGCTTGCTGTCCTCTTCC
AAATGCTGTATGTTGTGAAGATCACACACATTGTTGTCCTCAGGGATACCAGTGTAATGTTCAGGCTCAG
ACTTGTGAGTCGCAGGATGGGGTACTCTTTCCCTGGAACATACAGAAAGCTCCTGTTCACACTTTCTCCA
CCTCAGAAAAGAATGCTGGAAATGATGTGAAGTGTGACTCCAAAAAGTCTTGTGCTCCCCATAACACCTG

TTGTAAAAAGGCTTCGGGAGGTTGGGGCTGTTGTCTATATGAAGAGGCTGTTTGCTGTGAAGATCAATCC
CATTGCTGCCCTAAGGGCTACACCTGTGAGGTTTCGGCAGGACTGTGTCTGAAAAAGGATGGCTTCTACT
GGGATGGTCTGCTCTCTAATAGGAAAAGATCTTTCAGCACCTTGTAA

cDNA data:

cDNA sequence data: No cDNA data (or empty cDNA entry).

Genomic data:

Note: Genomic data (exons).
From NCBI: BAHO01286349.1 GI:405617871
No exon sequence to show (or no genomic sequence entry).

Coding exon 1 Exon type: sn
Note: Encodes a potential signal peptide (along with the 5 codons from exon 0), and also an N-half module which, with no C-half to complete it, is apparently a paragranulin.
ACTTTGTACAAGTTTTCAGTGCTATGTCTTTGGCTGGGTCTGGTAACAGCACTGAAATGTCCTGGTGGAT
TGGTTTGTGACAATGAAGATCACTGCTGTGAGCTTCCAGGAGGAGGGTATGAGTGTTGCCCTCAGTCAAA
G

Coding exon 2 Exon type: n*
GAGCATATGAGGTCTTTCCCGATGGTCCAGTCTGGAATGTTGAATGAAGTTACAGAGAACTTGTGTCTAG
ATCACACTTCATGCCCTCTGGAGTACTCCTGTATGCGTACACCTGCAGGGTCCTATGGCTGCTGTCCTGT
AGAGGAG

From NCBI: BAHO01286353.1 GI:405617867
Note: Does not overlap with BAHO01286349.1, but the first progranulin exon in it is a c*, which is expected to come next after the n* exon.

Coding exon 3 Exon type: c*
GCTGTATCCTGTCGTGATGGAAATCACTGTTGTCCTAAAGGTTTTCTTTGCAGTGCAGATGGGAAATCCT
GTATAAAACGCTTAG

Coding exon 4 Exon type: n
ATGACTTGAACTTGGGTGCAATTATTTGTCCAGATGAAACTTCTGAGTGTCCTGATGAGTCTACCTGCTG
CCAGCTACCAAGTGGACAGTGGGGGTGTTGCCCCTTCTCGCAG

Coding exon 5 Exon type: c
GCTGTGTGTTGTTCAGATAAATTGCATTGCTGCCCTCATGGAACATCTTGTGATGTAGCTCACTCTCAGT
GTTTAAAAGGACTCTCAAAGACACCTATGTGGAGCAGTTTTCCAGCAACCAAGAGAG

Coding exon 6 Exon type: n'
Note: The n' indicates mutations affecting the first and third Cys codons for the N-half module.
CCCATGATGTGAAATACAATGCCACGAGCAGCTGTCCCCATCTCTTTACAAGCTGTAAAGGGGAGTCTGG
AGAAGAGACTTGCTGTCCTCTATCAAAG

Coding exon 7 Exon type: cn
GCTGTGTGCTGTGAAGATCATACCCACTGCTGCCCAGAGGGGACCACTTGTGATGTAAAGGAAGGGAAGT
GTCAGCACAAAGTCCTGGCTATCCCCTGGTTCACTAAGAGACCAGCTGGACCAGTGAAGAAGTCTCTGAC
TACAGAAGAGAAGAGCGGGGAAAGAGACGTGAAATGTAATGATACAGTTGCTTGTCCTGATGGGAGCACC
TGTTGTAAGGTGCAGTCTGGAGACTGGGCCTGCTGCCCCCTGGCTAAA

Coding exon 8 Exon type: cn
GCTGTGTGCTGTGAAGATCATATCCACTGCTGTCCAGAGGGGACCACTTGTGATGTAAAGGAAGGAAAGT
GTCAGCACAAAGTCCTGGCTATCCCCTGGTTCACTAAGAGACCAGCTGGACCAGTGAAGAAGTCTCTGAC
TACAGAAGAGAAGAGCGGGGAAAGAGACGTGAAATGTAATGATACAGTTGCTTGTCCTGATGGGAGCACC
TGTTGTAAGGTGCAGTCTGGAGACTGGGCCTGCTGCCCCCTGGCTAAA

Coding exon 9 Exon type: cn
GCTGTGTGCTGTGAAGATCATACCCACTGCTGTCCAGAGGGGACCACTTGTGATGTAAAGGAAGGAAAGT
GTCAGCACAAAGTCCTGGCTATCCCCTGGTTCACTAAGAGACCAGCTGGACCAGTGAAGAAGTCTCTGAC
TACAGAAGAGAAGAGCGGGGAAAGAGACGTGAAATGTAATGATACAGTTGCTTGTCCTGATGGGAACACT
TGTTGTAAGATGCAGTCTGGGGACTGGGGCTGCTGTCCTGCGCCTAAT

Coding exon 10 **Exon type: cn**
GCTGTGTGCTGTGAAGATCATATCCACTGCTGTCCAGAGGGGACCACTTGTGATGCAAAGGGAGAAGCAT
GTAACCAGAATATCTTGGTTGTCCCCTGGCTCACTAAGAGACCAGCCACACCTGTGAAGAAGTCTCGGAC
TACAGAAGAGAAGAGCATGGAAAGAGATGTGAAATGTAATGATACAGTTGCTTGTCCTGATGGGAACACC
TGTTGTAAGATGCAGTCTGGGAACTGGGGCTGCTGCCCCATCGCTGAA

From NCBI: AFYH01038604.1 GI:346176232
Note: Overlaps BAHO01286353.1 including exons 9 and 10, and has two more cn exons given in this entry.
Coding exon 11 **Exon type: cn**
GCAGTGTGCTGTGCTGATCATATCCACTGCTGTCCACAAGGTTATACATGTGAAACCGAAACATGTGAGA
AGGGGAAGCTATCTTTCCCTTGGATGCAGAAGGAGCCAGCTATTACTAACAAGCTACCTGAAGAAGAAAG
TGCTCCCAGTGTAATGTGTGATGATCAAACAGAATGCCCTGTTGGAAATACTTGCTGTAAATATAGCACT
GGAGAGTGGGGATGCTGCCCTCTATCTCAG

Coding exon 12 **Exon type: cn**
GCTGTTTGCTGCAGTGACCACAAGCATTGTTGCCCAACTGATTATACTTGTGACCTTTTGCATAGCACAT
GCTCAAAGGGAAAGATAGCCATTCCTTGGGTCAGTAAGACCGTGGCGAGAAAAATGATCAGGACTTCTAC
AGATCCTCTCACTGTTGTGACGTGTGATTCTCATACTAGGTGCCCTGATAGAAACACATGCTGTAAGCTT
CTCACAGGAAAATGGGCTTGCTGTCCTCTTCCAAAT

From NCBI: BAHO01286354.1 GI:405617866
Note: Overlaps AFYH01038604.1 including exons 11 and 12, and provides the final 2 coding exons for the gene, which are given in this entry.
Coding exon 13 **Exon type: cn**
GCTGTATGTTGTGAAGATCACACACATTGTTGTCCTCAGGGATACCAGTGTAATGTTCAGGCTCAGACTT
GTGAGTCGCAGGATGGGGTACTCTTTCCCTGGAACATACAGAAAGCTCCTGTTCACACTTTCTCCACCTC
AGAAAAGAATGCTGGAAATGATGTGAAGTGTGACTCCAAAAAGTCTTGTGCTCCCCATAACACCTGTTGT
AAAAAGGCTTCGGGAGGTTGGGGCTGTTGTCTATATGAAGAG

Coding exon 14 **Exon type: c**
Note: up to the stop codon.
GCTGTTTGCTGTGAAGATCAATCCCATTGCTGCCCTAAGGGCTACACCTGTGAGGTTTCGGCAGGACTGT
GTCTGAAAAAGGATGGCTTCTACTGGGATGGTCTGCTCTCTAATAGGAAAAGATCTTTCAGCACCTTGTA
A

Genomic sequence note: A genomic sequence is not presented (see note 2 in header).

00028 **L. chalumnae progranulin C** Short name: **L_chaC** Date entered: 2014-03

Species: ***Latimeria chalumnae (Coelacanth)***
Taxonomy (via NCBI): *Eukaryota; Metazoa; Chordata; Craniata; Vertebrata; Euteleostomi; Coelacanthiformes; Coelacanthidae; Latimeria.*
General Comment: Incomplete sequence. The protein sequence is tentative but the best current prediction for a complete product. Although the progranulin form labelled C was originally chosen for a fish small form, the common occurrence of repeated sequence in the middle of progranulin genes has resulted in a long progranulin encoded in this Coelacanth gene, despite its closer sequence relationship with other fish small forms.

Protein Sequence
MKPLAVILLWVGLASSALVCQDGTQCPDQSTCCQTETGFGCCPLLKAVCCSTAYCCPEGYTCNVQEGVCE
RNGLRIPLVSKTPPLENPELSLLRVQSNASVVFCDNFHHCPDGNTCCRGPTGTWFCCPHPSAYCCRDGYH
CCPYGYFCDVTSTRCVKWGISIPSEDKMAALLATDALSIVSEPPSSAVYCGNQYYCPDGNTCCTLPSGSW
ACCPHPHAICCRDGYHCCPYGYFCDFTSTKCTKGGVSIPSEEKMPALVQTNALSITSESQSSKVYCGNQY
YCPDGNTCCKLPSGSWGCCPHPHAICCRDGYHCCPYGYFCDFTSTKCTKGGVSIPSEEKMPALVQTNALS
ITSESPSSAVYCGNQYYCPDGNTCCKLPSGSWGCCPHPHAICCRDGYHCCPYGYICDFTSTRCKRRGVSI
PSEAKIPAFVQTSALSITSESPSSAVYCGNKQYCPDGNTCCKLPSGSWGCCPHPHAICCRDGYHCCPYGY
FCDFTSTKCTKGGVSIPSEEKMPALVQTNALSITSESPSSAVYCGNQYYCPDGNTCCKLPSGSWGCCPYR
QSVCCRDGYHCCPAGYFCDLTSTQCIPSNPFNKKLPPKEINAVSVTSAFDSSNTFCELDHCCLAGNKCCR
LQsgswgllsists*

Transcript:

Predicted sequence:
Note: Predicted sequence is tentative. It may omit exons at the beginning and end. See notes with the exon data.
Derivation: Tentative prediction from exon data (see genomic data below).
ATGAAACCATTAGCCGTGATCTTATTGTGGGTGGGCTTGGCTTCCAGTGCACTGGTGTGCCAGGATGGTA
CTCAATGTCCTGATCAGTCGACCTGCTGTCAAACAGAAACAGGATTTGGTTGCTGTCCACTCTTGAAGGC
TGTTTGTTGTTCCACTGCATACTGCTGCCCGGAAGGCTACACCTGTAATGTCCAAGAAGGAGTCTGTGAG
AGGAATGGACTTCGGATTCCTTTGGTCAGCAAGACTCCACCTCTGGAAAACCCCGAGCTATCTCTACTCC
GTGTTCAGAGCAATGCGTCTGTCGTTTTTTGCGATAACTTCCATCACTGTCCTGATGGCAACACTTGTTG
TAGAGGCCCAACAGGAACATGGTTCTGCTGTCCACATCCTTCGGCCTATTGTTGCAGGGATGGTTATCAC
TGCTGTCCTTATGGCTACTTCTGTGATGTTACTTCAACAAGATGTGTAAAATGGGGTATCAGTATCCCAT
CAGAAGACAAGATGGCAGCTCTTCTAGCAACAGATGCTTTGAGCATTGTCTCAGAGCCTCCAAGCTCAGC
AGTGTACTGTGGTAACCAGTATTACTGCCCTGATGGCAATACGTGCTGCACGTTACCAAGTGGATCATGG
GCCTGCTGTCCTCATCCTCACGCCATCTGTTGTAGGGATGGTTATCATTGCTGTCCTTATGGCTACTTCT
GTGATTTTACTTCAACAAAATGTACAAAAGGGGGTGTCAGTATCCCATCAGAAGAGAAGATGCCAGCCCT
TGTACAGACGAATGCTTTGAGCATCACCTCAGAATCTCAGAGCTCAAAAGTGTACTGTGGTAACCAGTAT
TACTGCCCTGATGGCAATACATGCTGCAAGTTACCAAGTGGATCATGGGGCTGCTGTCCTCATCCTCACG
CCATCTGTTGTAGGGATGGTTATCACTGCTGTCCTTATGGCTACTTCTGTGATTTTACTTCAACAAAATG
TACAAAAGGGGGTGTCAGTATCCCATCAGAAGAGAAGATGCCAGCCCTTGTACAGACGAATGCTTTGAGC
ATCACCTCAGAGTCTCCGAGCTCAGCAGTGTACTGTGGTAACCAGTATTACTGCCCTGATGGCAATACAT
GCTGCAAGTTACCAAGTGGATCATGGGGCTGCTGTCCTCATCCTCACGCCATCTGTTGTAGGGATGGTTA
TCACTGCTGTCCTTATGGCTACATCTGTGATTTTACTTCAACAAGATGTAAAAGAAGGGGTGTCAGTATC
CCATCAGAAGCCAAGATACCAGCTTTTGTACAGACAAGTGCTTTGAGCATCACCTCAGAGTCTCCGAGCT
CAGCAGTGTACTGTGGTAACAAGCAGTACTGCCCTGATGGCAATACATGCTGCAAGTTACCAAGTGGATC
ATGGGGCTGCTGTCCACATCCTCATGCCATCTGTTGTAGGGATGGTTATCACTGCTGTCCTTATGGCTAC
TTCTGTGATTTTACTTCAACAAAATGTACAAAAGGGGGTGTCAGTATTCCATCAGAAGAGAAGATGCCAG
CCCTTGTACAGACGAATGCTTTGAGCATCACCTCAGAGTCTCCGAGCTCAGCAGTGTACTGTGGTAACCA
GTATTACTGCCCTGATGGCAATACATGCTGCAAGTTACCAAGTGGATCTTGGGGCTGCTGTCCTTATCGT
CAATCGGTCTGTTGCAGGGACGGTTATCACTGCTGTCCTGCTGGCTACTTCTGTGATTTAACTTCAACCC
AATGCATACCAAGCAATCCATTTAACAAGAAATTACCTCCAAAAGAGATTAATGCTGTAAGTGTCACCTC
AGCGTTTGATAGCTCAAATACATTTTGTGAGTTGGACCACTGTTGTCTTGCTGGTAATAAATGCTGCAGG
TTGCAAAgtggcagctgggggctgctgtccatatccaccagttag

cDNA data:
cDNA sequence data: No cDNA data (or empty cDNA entry).

Genomic data:
Note: Genomic data (exons).
From NCBI: BAHO01048624.1 GI:405887596
Note: First coding exon is from 13399-13536 close to the end of this wgs sequence.
Coding exon 1 **Exon type:** **sn**
Note: from the start codon (which is at the beginning of this exon)
ATGAAACCATTAGCCGTGATCTTATTGTGGGTGGGCTTGGCTTCCAGTGCACTGGTGTGCCAGGATGGTA
CTCAATGTCCTGATCAGTCGACCTGCTGTCAAACAGAAACAGGATTTGGTTGCTGTCCACTCTTGAAG

From NCBI: AFYH01132928.1 GI:346075846
Note: This wgs sequence contains 3 cn exons. It does not overlap with BAHO01048624.1, so it is not known if the gap has exons. But the fish small C type progranulins have a cn exon as the second coding exon, and our analysis places the encoded C-half module only of the first of these 3 cn exons in close relationship with the first C-half module in those other fish small forms. Therefore, we tentatively label these exons 2 to 4.
Coding exon 2 **Exon type:** **cn**
Note: Most likely the second coding exon in this gene. But a gap in sequence needs to be bridged.
GCTGTTTGTTGTTCCACTGCATACTGCTGCCCGGAAGGCTACACCTGTAATGTCCAAGAAGGAGTCTGTG
AGAGGAATGGACTTCGGATTCCTTTGGTCAGCAAGACTCCACCTCTGGAAAACCCCGAGCTATCTCTACT
CCGTGTTCAGAGCAATGCGTCTGTCGTTTTTTGCGATAACTTCCATCACTGTCCTGATGGCAACACTTGT
TGTAGAGGCCCAACAGGAACATGGTTCTGCTGTCCACATCCTTCG

Coding exon 3 **Exon type:** **cn**
Note: See exon 2 note
GCCTATTGTTGCAGGGATGGTTATCACTGCTGTCCTTATGGCTACTTCTGTGATGTTACTTCAACAAGAT

GTGTAAAATGGGGTATCAGTATCCCATCAGAAGACAAGATGGCAGCTCTTCTAGCAACAGATGCTTTGAG
CATTGTCTCAGAGCCTCCAAGCTCAGCAGTGTACTGTGGTAACCAGTATTACTGCCCTGATGGCAATACG
TGCTGCACGTTACCAAGTGGATCATGGGCCTGCTGTCCTCATCCTCAC

Coding exon 4 **Exon type: cn**
Note: see exon 2 note
GCCATCTGTTGTAGGGATGGTTATCATTGCTGTCCTTATGGCTACTTCTGTGATTTTACTTCAACAAAAT
GTACAAAAGGGGGTGTCAGTATCCCATCAGAAGAGAAGATGCCAGCCCTTGTACAGACGAATGCTTTGAG
CATCACCTCAGAATCTCAGAGCTCAAAAGTGTACTGTGGTAACCAGTATTACTGCCCTGATGGCAATACA
TGCTGCAAGTTACCAAGTGGATCATGGGGCTGCTGTCCTCATCCTCAC

From NCBI: BAHO01048630.1 GI:405887590
Note: This wgs contig contributes another 3 cn exons and a c-half in another exon. This 4th exon is like a cn exon with mutations in the n sequence and a likely splice donor before the mutant n. This wgs sequence does not overlap with AFYH01132928.1, but continues a repeat of cn exons closely related to the tentative exon 4 in AFYH01132928.1, so for now it is reasonable to continue the numbering in labelling these exons.
Coding exon 5 **Exon type: cn**
Note: The labelling as exon 5 is tentative, but if there is another exon in the data gap, it will likely be one of these repeat exons.
GCCATCTGTTGTAGGGATGGTTATCACTGCTGTCCTTATGGCTACTTCTGTGATTTTACTTCAACAAAAT
GTACAAAAGGGGGTGTCAGTATCCCATCAGAAGAGAAGATGCCAGCCCTTGTACAGACGAATGCTTTGAG
CATCACCTCAGAGTCTCCGAGCTCAGCAGTGTACTGTGGTAACCAGTATTACTGCCCTGATGGCAATACA
TGCTGCAAGTTACCAAGTGGATCATGGGGCTGCTGTCCTCATCCTCAC

Coding exon 6 **Exon type: cn**
GCCATCTGTTGTAGGGATGGTTATCACTGCTGTCCTTATGGCTACATCTGTGATTTTACTTCAACAAGAT
GTAAAAGAAGGGGTGTCAGTATCCCATCAGAAGCCAAGATACCAGCTTTTGTACAGACAAGTGCTTTGAG
CATCACCTCAGAGTCTCCGAGCTCAGCAGTGTACTGTGGTAACAAGCAGTACTGCCCTGATGGCAATACA
TGCTGCAAGTTACCAAGTGGATCATGGGGCTGCTGTCCACATCCTCAT

Coding exon 7 **Exon type: cn**
GCCATCTGTTGTAGGGATGGTTATCACTGCTGTCCTTATGGCTACTTCTGTGATTTTACTTCAACAAAAT
GTACAAAAGGGGGTGTCAGTATTCCATCAGAAGAGAAGATGCCAGCCCTTGTACAGACGAATGCTTTGAG
CATCACCTCAGAGTCTCCGAGCTCAGCAGTGTACTGTGGTAACCAGTATTACTGCCCTGATGGCAATACA
TGCTGCAAGTTACCAAGTGGATCTTGGGGCTGCTGTCCTTATCGTCAA

Coding exon 8 **Exon type: c**
Note: It has not been possible to find downstream exons in the wgs data to complete this gene as one would expect the coding sequence to end. Just in case this is an unusual last coding exon, the sequence through to a stop codon has been included here (in loower case after a possible splice donor).
TCGGTCTGTTGCAGGGACGGTTATCACTGCTGTCCTGCTGGCTACTTCTGTGATTTAACTTCAACCCAAT
GCATACCAAGCAATCCATTTAACAAGAAATTACCTCCAAAAGAGATTAATGCTGTAAGTGTCACCTCAGC
GTTTGATAGCTCAAATACATTTTGTGAGTTGGACCACTGTTGTCTTGCTGGTAATAAATGCTGCAGGTTG
CAAAgtggcagctgggggctgctgtccatatccaccagttag

Genomic sequence note: A genomic sequence is not presented (see note 2 in header).

00035 **N. vectensis progranulin** Short name: **N_vec** Date entered: 2014-03

Species: ***Nematostella vectensis (starlet sea anemone)***
Taxonomy (via NCBI): *Eukaryota; Metazoa; Cnidaria; Anthozoa; Hexacorallia; Actiniaria; Edwardsiidae; Nematostella.*
General Comment:

Protein Sequence

MLSLVVGLVILGATPVISVQCPGGEVECPDDYTCCLMPNRMYGCCPIPNAVCCSDLRHCCPYGYKCDTSG
GACIRGKHVLQLLKKIPSLSVKVASPIKNQKDVLCPDESVCPDGNTCCLTEFGLYGCCPTPKAVCCLDRL
HCCPSGYKCDLARHECVQENVQTNSQVKPLIHLRKQNKNILGTTVCPDKASKCPDGNTCCKLSSDQYGCC
PLPNAMCCDDGVHCCPNGYTCDTSSGRCSKGDSVLPLFKKTPAKQLKNVVCPDGASECPDGNTCCKLSSG
QYGCCPLPNAVCCDDGVHCCPNGYTCDTASGTCNKGDSVLPLFKKTPAKQLKNVVCPDGTSQCPDGNTCC
KLSSGQYGCCPLPNAVCCDDGVHCCPDGYTCNSSSGTCSEGDSVLPLFKKTPAKQLKNVVCPDGASHCPD

GNTCCKLSSGQYGCCPLPNAVCCDDGVHCCPNGYTCNSSGGTCNKGDSVLPLFKKTPAKQLKHVVCPDGT
SQCPDGNTCCKLSSGQYGCCPLPNAVCCDDGVHCCPNGYTCNSSSGTCSEGDSVLPLFKKTPAKQLKNVV
CPDETSQCPDGNTCCKLSSGQYGCCPLPNAVCCDDGVHCCPNGYTCDTSSGRCNKGDSVLPLFKKTPAKQ
LKNVVCPGGEAECPDGNTCCKLSSGQYGCCPLPNAVCCDDGSVLPLVKKTPAQKFKHVICEGSHLACPDG
TTCCKKRSGGYACCPIPKAVCCSDEVHCCPNGYSCDLSSKKCVKGASRVPILQNSLALCIDIQTTSELST
LHSVSLISAANNRPGASGANYITCNDGSHCYDYETCCELSTGSYGCCPLPNAVCCSDGKHCCPCGYHCDI
SSSTCDMAKDSTPMVSYHATVQRAGVGEIKCKDGSHCNTGETCCLVGPTKYGCCPLPSAVCCADMQHCCP
SGYTCNDKSEHCQRGRHVLPMFQKHPSVHPRPAVAKTPVIIDHTIRFVPRGGRGAGCTCEPYHTCCGSSS
AQQHCCPLPNASCCSDGLHCCPQGFICDDQKGCVKQP*

Transcript:

Predicted sequence:

Note: The last approximately 60% is confirmed by cDNA data.
Derivation: predicted from stellabase c441202720.Contig1

ATGTTGAGTCTAGTCGTAGGGCTGGTGATACTTGGTGCAACTCCAGTGATCTCTGTCCAATGTCCAGGTG
GAGAGGTGGAGTGTCCTGATGACTATACCTGTTGTCTAATGCCCAATAGAATGTATGGCTGTTGCCCTAT
CCCCAATGCTGTCTGTTGTTCAGATCTAAGGCACTGCTGCCCTTATGGTTACAAATGTGATACATCAGGA
GGGGCCTGCATAAGAGGAAAGCATGTGCTACAGCTGCTCAAGAAAATCCCATCTCTTTCAGTTAAGGTTG
CCAGCCCCATCAAAAATCAGAAGGATGTTTTGTGCCCAGATGAATCTGTTTGTCCAGATGGTAACACCTG
CTGTTTAACTGAATTTGGACTGTATGGCTGCTGTCCTACACCAAAAGCAGTGTGTTGCCTTGATCGCCTA
CACTGCTGTCCCAGTGGATACAAGTGTGACCTTGCCAGGCATGAATGTGTGCAGGAAAATGTGCAAACGA
ATTCACAGGTCAAACCTCTTATCCATTTAAGGAAACAAAACAAGAATATATTAGGTACTACTGTCTGTCC
AGACAAGGCGTCAAAGTGTCCCGATGGTAATACTTGCTGCAAACTATCCTCTGATCAATACGGCTGCTGT
CCTCTTCCCAACGCTATGTGCTGTGATGATGGCGTTCACTGCTGCCCTAATGGATACACGTGCGACACCT
CGAGTGGCAGATGCAGCAAAGGAGACTCCGTGCTTCCACTTTTCAAGAAAACACCAGCCAAGCAGCTGAA
GAATGTTGTTTGTCCAGACGGGGCGTCAGAGTGTCCCGATGGTAATACTTGCTGCAAACTATCCTCTGGT
CAATACGGCTGCTGTCCTCTTCCCAATGCTGTGTGCTGTGATGATGGCGTTCACTGCTGCCCTAATGGAT
ACACGTGCGACACTGCGAGTGGCACATGCAACAAAGGAGACTCTGTGCTTCCACTTTTCAAGAAAACACC
AGCCAAGCAGCTAAAGAATGTTGTCTGTCCAGACGGGACGTCACAGTGTCCCGATGGTAATACTTGCTGC
AAACTATCCTCTGGTCAATACGGCTGCTGTCCTCTTCCCAACGCTGTGTGCTGTGATGATGGCGTTCACT
GCTGCCCTGATGGATACACGTGCAACTCCTCGAGTGGCACATGCAGCGAAGGAGACTCCGTGCTTCCACT
TTTCAAGAAAACACCAGCCAAGCAGCTGAAGAATGTTGTTTGTCCAGACGGGGCGTCACACTGTCCCGAT
GGTAATACTTGCTGCAAACTATCCTCTGGTCAATACGGCTGCTGTCCTCTTCCCAACGCTGTGTGCTGTG
ATGATGGCGTTCACTGCTGCCCTAATGGATACACGTGCAACTCCTCGGGAGGCACATGCAACAAAGGAGA
CTCTGTGCTTCCACTTTTCAAGAAAACACCAGCCAAGCAGCTGAAGCATGTTGTTTGTCCAGACGGGACG
TCACAGTGTCCCGATGGTAATACTTGCTGCAAACTATCCTCTGGTCAATACGGCTGCTGTCCTCTTCCCA
ACGCTGTGTGCTGTGATGATGGCGTTCACTGCTGCCCTAATGGATACACGTGCAACTCCTCGAGTGGCAC
ATGCAGCGAAGGAGACTCCGTGCTTCCACTTTTCAAGAAAACACCAGCCAAGCAGCTGAAGAATGTTGTT
TGTCCAGACGAGACGTCACAGTGTCCCGATGGTAATACTTGCTGCAAACTATCCTCTGGTCAATACGGCT
GCTGTCCTCTTCCCAATGCTGTGTGCTGTGATGATGGCGTTCACTGCTGCCCTAATGGATACACGTGCGA
CACCTCGAGTGGCAGATGCAACAAAGGAGACTCTGTGCTTCCACTTTTCAAGAAAACACCAGCCAAGCAG
CTGAAGAATGTTGTGTGTCCAGGTGGGGAAGCAGAATGTCCCGATGGTAATACTTGCTGCAAACTATCCT
CTGGTCAATACGGCTGCTGTCCTCTTCCCAATGCTGTGTGCTGTGATGATGGCTCTGTGCTTCCTCTCGT
CAAGAAAACACCAGCTCAGAAGTTCAAGCATGTTATCTGCGAGGGTAGTCACCTTGCATGCCCAGATGGT
ACCACTTGTTGTAAGAAGAGGTCAGGGGGTTACGCTTGCTGTCCAATCCCCAAGGCCGTTTGTTGCTCGG
ATGAAGTCCACTGCTGCCCTAATGGGTATTCTTGTGACCTTTCCAGTAAAAAATGTGTCAAGGGTGCTTC
TCGCGTGCCGATTTTACAGAACTCTCTTGCCCTTTGCATTGATATACAGACGACATCAGAATTATCCACA
CTTCACAGTGTTAGTTTGATATCGGCTGCGAATAACCGACCAGGGGCTTCAGGAGCGAATTACATCACTT
GCAACGACGGTTCTCACTGCTACGATTATGAAACTTGCTGCGAACTTTCTACCGGCTCCTACGGCTGCTG
TCCCCTACCTAACGCTGTCTGCTGTAGCGATGGCAAGCACTGCTGCCCGTGTGGTTATCACTGCGACATA
AGCTCGTCGACGTGCGACATGGCCAAGGACTCTACACCTATGGTCTCATACCACGCAACTGTTCAGCGTG
CTGGCGTTGGCGAAATCAAATGCAAAGACGGTTCGCACTGTAACACCGGTGAAACGTGCTGCCTTGTGGG
GCCAACCAAATACGGCTGTTGTCCCCTTCCCTCAGCTGTTTGCTGTGCTGACATGCAGCACTGCTGTCCT
TCGGGCTACACTTGCAACGACAAGAGCGAACATTGTCAGCGAGGACGCCATGTTTTACCGATGTTCCAAA
AGCACCCCTCGGTGCATCCACGACCTGCTGTTGCAAAGACGCCTGTGATTATCGATCATACGATTCGTTT
CGTGCCGAGAGGTGGCCGAGGTGCTGGATGCACCTGCGAGCCGTACCACACATGCTGTGGCTCCTCGTCC
GCTCAGCAGCACTGCTGTCCTCTTCCGAATGCTTCGTGCTGCAGCGATGGTCTGCATTGTTGTCCACAAG
GGTTCATTTGCGATGACCAAAAGGGCTGTGTCAAACAACCATAG

cDNA data:

From: NCBI est: Contig of GI:(162034518, 161990857, 162099514, 82863220, 162101879, 162095075, 162034168, 162046635, 161990856, 162090445, 162098231, 162101878, 162090446, 162098232)

Note: In assembling the cDNA data, Cap3 produced 2 contigs, which were found to overlap by 15 bases to produce this sequence. It is consistent with the prediction from genomic data, and confirms the last 60% of the prediction.

```
GTGTCCGATGGTATACTTGCTGCAACTATCCTCTGGTCAATACGGCTGCTGTCCTCTTCCCAACGCTGTG
TGCTGTGATGATGGCGTTCACTGCTGCCCTAATGGATACACGTGCAACTCCTCGGGAGGCACATGCAACA
AAGGAGACTCTGTGCTTCCACTTTTCAAGAAAACACCAGCCAAGCAGCTGAAGCATGTTGTTTGTCCAGA
CGGGACGTCACAGTGTCCCGATGGTAATACTTGCTGCAAACTATCCTCTGGTCAATACGGCTGCTGTCCT
CTTCCCAACGCTGTGTGCTGTGATGATGGCGTTCACTGCTGCCCTAATGGATACACGTGCAACTCCTCGA
GTGGCACATGCAGCGAAGGAGACTCCGTGCTTCCACTTTTCAAGAAAACACCAGCCAAGCAGCTGAAGAA
TGTTGTTTGTCCAGACGAGACGTCACAGTGTCCCGATGGTAATACTTGCTGCAAACTATCCTCTGGTCAA
TACGGCTGCTGTCCTCTTCCCAATGCTGTGTGCTGTGATGATGGCGTTCACTGCTGCCCTAATGGATACA
CGTGCGACACCTCGAGTGGCAGATGCAACAAAGGAGACTCTGTGCTTCCACTTTTCAAGAAAACACCAGC
CAAGCAGCTGAAGAATGTTGTGTGTCCAGGTGGGGAAGCAGAATGTCCCGATGGTAATACTTGCTGCAAA
CTATCCTCTGGTCAATACGGCTGCTGTCCTCTTCCCAATGCTGTGTGCTGTGATGATGGCTCTGTGCTTC
CTCTCGTCAAGAAAACACCAGCTCAGAAGTTCAAGCATGTTATCTGCGAGGGTAGTCACCTTGCATGCCC
AGATGGTACCACTTGTTGTAAGAAGAGGTCAGGGGGTTACGCTTGCTGTCCAATCCCCAAGGCCGTTTGT
TGCTCGGATGAAGTCCACTGCTGCCCTAATGGGTATTCTTGTGACCTTTCCAGTAAAAAATGTGTCAAGG
gtgcttctcgcgtgccgattttacagaactctcttgccctttgcattgatatacagacgacatcagaatt
atccacacttcacagtgttagtttgatatcggctgcgaataaccgaccaggggcttcaggagcgaattac
atcacttgcaacgacggttctcactgctacgattatgaaacttgctgcgaactttctaccggctcctacg
gctgctgtcccctacctaacgctgtctgctgtagcgatggcaagcactgctgcccgtgtggttatcactg
cgacataagctcgtcgacgtgcgacatggccaaggactctacacctatggtctcataccacgcaactgtt
cagcgtgctggcgttggcgaaatcaaatgcaaagacggttcgcactgtaacaccggtgaaacgtgctgcc
ttgtggggccaaccaaatacggctgttgtccccttccctcagctgtttgctgtgctgacatgcagcactg
ctgtccttcgggctacacttgcaacgacaagagcgaacattgtcagcgaggacgccatgttttaccgatg
ttccaaaagcacccctcggtgcatccacgacctgctgttgcaaagacgcctgtgattatcgatcatacga
ttcgtttcgtgccgagaggtggccgaggtgctggatgcacctgcgagccgtaccacacatgctgtggctc
ctcgtccgctcagcagcactgctgtcctcttccgaatgcttcgtgctgcagcgatggtctgcattgttgt
ccacaagggttcatttgcgatgaccaaaagggctgtgtcaaacaaccatagactcatacagtcacagtca
gaatcgtgacaagcgttccttgatctttccattgtatgtaaaaaaatacccttttgtgtcacagacgaac
ctccgagctacgatggtagatagctattcaatactaatttgtctagaatattatttttatttttggtac
caaacgatctttttgtaactatacgattcggaataaaaaaaatacatcatcaaaaaa
```

Genomic data:

Note: Genomic data (exons). The gene was predicted from stellabase c441202720.Contig1. The NCBI entry gi: 149353570 gb: ABAV01000652.1 has errors. With only 3 exons encoding just over 12 modules, this gene is different from vertebrate progranulin genes. The second exon encodes nearly 9 modules, the eighth of which is truncated in the C-half. Six and the truncated module constitute a repeat displaying a high level of identity. Among the chordates, Branchiostoma presents a similar example of repetitive multiple module exons.

From stellabase: c441202720.Contig1

Coding exon 1 **Exon type: snc**

Note: from the start codon (which is at the beginning of this exon)

```
ATGTTGAGTCTAGTCGTAGGGCTGGTGATACTTGGTGCAACTCCAGTGATCTCTGTCCAATGTCCAGGTG
GAGAGGTGGAGTGTCCTGATGACTATACCTGTTGTCTAATGCCCAATAGAATGTATGGCTGTTGCCCTAT
CCCCAATGCTGTCTGTTGTTCAGATCTAAGGCACTGCTGCCCTTATGGTTACAAATGTGATACATCAGGA
GGGGCCTGCATAAGAGGAAAGCATGTGCTACAGCTGCTCAAGAAAATCCCATCTCTTTCAGTTAAG
```

Coding exon 2 **Exon type: ncncncncncncncnc”nc**

Note: Here c” encodes the beginning of a C-half module lacking the last 4 Cys of the motif.

```
GTTGCCAGCCCCATCAAAAATCAGAAGGATGTTTTGTGCCCAGATGAATCTGTTTGTCCAGATGGTAACA
CCTGCTGTTTAACTGAATTTGGACTGTATGGCTGCTGTCCTACACCAAAAGCAGTGTGTTGCCTTGATCG
CCTACACTGCTGTCCCAGTGGATACAAGTGTGACCTTGCCAGGCATGAATGTGTGCAGGAAAATGTGCAA
ACGAATTCACAGGTCAAACCTCTTATCCATTTAAGGAAACAAAACAAGAATATATTAGGTACTACTGTCT
GTCCAGACAAGGCGTCAAAGTGTCCCGATGGTAATACTTGCTGCAAACTATCCTCTGATCAATACGGCTG
CTGTCCTCTTCCCAACGCTATGTGCTGTGATGATGGCGTTCACTGCTGCCCTAATGGATACACGTGCGAC
ACCTCGAGTGGCAGATGCAGCAAAGGAGACTCCGTGCTTCCACTTTTCAAGAAAACACCAGCCAAGCAGC
TGAAGAATGTTGTTTGTCCAGACGGGGCGTCAGAGTGTCCCGATGGTAATACTTGCTGCAAACTATCCTC
```

TGGTCAATACGGCTGCTGTCCTCTTCCCAATGCTGTGTGCTGTGATGATGGCGTTCACTGCTGCCCTAAT
GGATACACGTGCGACACTGCGAGTGGCACATGCAACAAAGGAGACTCTGTGCTTCCACTTTTCAAGAAAA
CACCAGCCAAGCAGCTAAAGAATGTTGTCTGTCCAGACGGGACGTCACAGTGTCCCGATGGTAATACTTG
CTGCAAACTATCCTCTGGTCAATACGGCTGCTGTCCTCTTCCCAACGCTGTGTGCTGTGATGATGGCGTT
CACTGCTGCCCTGATGGATACACGTGCAACTCCTCGAGTGGCACATGCAGCGAAGGAGACTCCGTGCTTC
CACTTTTCAAGAAAACACCAGCCAAGCAGCTGAAGAATGTTGTTTGTCCAGACGGGGCGTCACACTGTCC
CGATGGTAATACTTGCTGCAAACTATCCTCTGGTCAATACGGCTGCTGTCCTCTTCCCAACGCTGTGTGC
TGTGATGATGGCGTTCACTGCTGCCCTAATGGATACACGTGCAACTCCTCGGGAGGCACATGCAACAAAG
GAGACTCTGTGCTTCCACTTTTCAAGAAAACACCAGCCAAGCAGCTGAAGCATGTTGTTTGTCCAGACGG
GACGTCACAGTGTCCCGATGGTAATACTTGCTGCAAACTATCCTCTGGTCAATACGGCTGCTGTCCTCTT
CCCAACGCTGTGTGCTGTGATGATGGCGTTCACTGCTGCCCTAATGGATACACGTGCAACTCCTCGAGTG
GCACATGCAGCGAAGGAGACTCCGTGCTTCCACTTTTCAAGAAAACACCAGCCAAGCAGCTGAAGAATGT
TGTTTGTCCAGACGAGACGTCACAGTGTCCCGATGGTAATACTTGCTGCAAACTATCCTCTGGTCAATAC
GGCTGCTGTCCTCTTCCCAATGCTGTGTGCTGTGATGATGGCGTTCACTGCTGCCCTAATGGATACACGT
GCGACACCTCGAGTGGCAGATGCAACAAAGGAGACTCTGTGCTTCCACTTTTCAAGAAAACACCAGCCAA
GCAGCTGAAGAATGTTGTGTGTCCAGGTGGGGAAGCAGAATGTCCCGATGGTAATACTTGCTGCAAACTA
TCCTCTGGTCAATACGGCTGCTGTCCTCTTCCCAATGCTGTGTGCTGTGATGATGGCTCTGTGCTTCCTC
TCGTCAAGAAAACACCAGCTCAGAAGTTCAAGCATGTTATCTGCGAGGGTAGTCACCTTGCATGCCCAGA
TGGTACCACTTGTTGTAAGAAGAGGTCAGGGGGTTACGCTTGCTGTCCAATCCCCAAGGCCGTTTGTTGC
TCGGATGAAGTCCACTGCTGCCCTAATGGGTATTCTTGTGACCTTTCCAGTAAAAAATGTGTCAAGGGTG
CTTCTCGCGTGCCGATTTTACAGAACTCTCTTGCCCTTTGCATTGATATACAGACGACATCAGAATTATC
CACACTTCACAGTGTTAGTTTGATATCGGCTGCGAATAACCGACCAG

Coding exon 3 **Exon type:** ncncnc
Note: up to the stop codon.
GGGCTTCAGGAGCGAATTACATCACTTGCAACGACGGTTCTCACTGCTACGATTATGAAACTTGCTGCGA
ACTTTCTACCGGCTCCTACGGCTGCTGTCCCCTACCTAACGCTGTCTGCTGTAGCGATGGCAAGCACTGC
TGCCCGTGTGGTTATCACTGCGACATAAGCTCGTCGACGTGCGACATGGCCAAGGACTCTACACCTATGG
TCTCATACCACGCAACTGTTCAGCGTGCTGGCGTTGGCGAAATCAAATGCAAAGACGGTTCGCACTGTAA
CACCGGTGAAACGTGCTGCCTTGTGGGGCCAACCAAATACGGCTGTTGTCCCCTTCCCTCAGCTGTTTGC
TGTGCTGACATGCAGCACTGCTGTCCTTCGGGCTACACTTGCAACGACAAGAGCGAACATTGTCAGCGAG
GACGCCATGTTTTACCGATGTTCCAAAAGCACCCCTCGGTGCATCCACGACCTGCTGTTGCAAAGACGCC
TGTGATTATCGATCATACGATTCGTTTCGTGCCGAGAGGTGGCCGAGGTGCTGGATGCACCTGCGAGCCG
TACCACACATGCTGTGGCTCCTCGTCCGCTCAGCAGCACTGCTGTCCTCTTCCGAATGCTTCGTGCTGCA
GCGATGGTCTGCATTGTTGTCCACAAGGGTTCATTTGCGATGACCAAAAGGGCTGTGTCAAACAACCATA
G

Genomic sequence note: A genomic sequence is not presented (see note 2 in header).

00039 **O. lobularis progranulin** Short name: **O_lob** Date entered: 2014-03

Species: ***Oscarella lobularis (Sponge)***
Taxonomy (via NCBI): *Eukaryota; Metazoa; Porifera; Demospongiae; Homoscleromorpha; Homosclerophorida; Plakinidae; Oscarella.*
General Comment: Only cDNA data and only a partial transcript sequence. O. lobularis provided the first evidence of a progranulin in sponge, and its granulin sequences were used for an outgroup in analyses of chordate granulin modules. Unfortunately, no new sequences for this species have arrived in the database, but there has been more recent data accumulating from other sponges.

Protein Sequence
Protein sequence not yet prepared. May be too incomplete to show.

Transcript:
Note: Only 2 EST sequences, both providing data for the beginning of a reading frame for a signal peptide, a demi-module which has a Cys motif C-C-CC-C-C, and almost 3 granulin modules. The encoded polypeptides differ by 6% and may be alleles. We refer to them as O_lob1 and O_lob2.
The cDNA data define the transcript well (below), so no predicted sequence is required.

cDNA data:

From: NCBI: AM762841.1 GI:193857770
Note: This cDNA fragment provides incomplete 5'end data for the transcript.
GTTTTGCTTTTCCAAGCTCGCCTCGTGCTTTCCACAGCCCAAGAAGTGCTTCAAACCATGGCTCCTCTTA
CAGCGGCTTGCATCGTGTTCGCCGCAATCTGCGCGATTAGCGAGTGCAAATCGGTGTGTCCAGAAGGCAA
CGTGCCCTGTCCCTTGAATGACGGGACAATCAGCTGCTGCAACGAGGGATACTTCTGTGGAAGCAACGGT
ACTTGTCTTCAGTGGAACGACGCGAGCAATGTTATATGTCCTGATGGACAGTCTCAATGTCCGGCTGGAA
ATACTTGTTGCAAATTGTCTAGTGGACAATGGGGATGCTGTCCTCTTCCAAATGCAGTCTGTTGCTCTAA
CGGCGAAAATTGCTGTCCATCGGGATACACTTGTGATGTATCTGCGGGCACTTGCACGAAAGGAGACATT
GCGATAGCTATGATTGAAAAAAGGCCTGCTGCTTTGAGAGAAGGAGACGTCACATGTCCTGATGGACAGT
CTGAATGTCCGACTGGAAATACATGTTGCAAACTGTCTAGTGGACAATGGGGATGCTGTCCTATTCCAAA
TGCAGTCTGTTGCTCTGACGGAAAACATTGCTGTCCATCAGGATACACTTGTGATCTTTCAGCGGGCACT
TGCACGAAAGGAGACATTGCCATAGCTATGATTGAAAAAACGCCTGCTGCTTTGAGAGAAGAAAACGTTG
TATGTCCTGATGGACAGTCTCAATGTCCGGCTGGAAATACTTGTTGCAAATTGTCTAGTGGACAATGGGG
ATGCTGTCCTCTTCCAAATGCAGTCTGTTGCTCTAACGGAGAAAATTGCTGTCCATCGGGATACACTTGT
GATGTAT

From: NCBI: AM763289.1 GI:193860561
Note: This cDNA fragment provides incomplete 5'end data for the transcript.
CGCCTCGTGCCTTCCACAGCCCAAGAAATGCTTCAAACCATGGCTCCTCTTACAGCGGCTTGCATCGTTT
TCGCCGCAATCTGCGCGATTAGCGAGTGCAAATCGGTGTGTCCAGAAGGCAACGTGCCCTGTCCCTTGAA
TGACGGGACAATCAGCTGCTGCAACGAAGGATACTTCTGTGGAAGCAACGGTACTTGTCTTCAGTGGAAC
GACGCGAGCAATGTTATATGTCCTGATGGACAGTCTGAATGTCCGGCTGGAAATACTTGTTGCAAATTGT
CTAGTGGACAATGGGGATGCTGTCCTCTTCCAAATGCAGTCTGTTGCTCTAACGGAGAGAATTGCTGTCC
ATCAGGATACACTTGTGATCTATCAGCGGGCACTTGCACGAAAGGAGACATTGCCATAGCTATGATTGAA
AAAAGGCCTGCTGCTTTGAGAGAAGGAGACACATGTCCTGATGGACAGTCTGAATGTCCGACTGGAAATA
CATGTTGCAAACTGTCTAGTGGAGAATGGGGATGCTGTCCTCTTCCAAATGCAGTCTGTTGCTCTGACGG
AGAACACTGCTGTCCATCAGGATACACTTGTGATCTATCAGCGGGCACTTGCACAAAAGGAGACATTGCC
ATAGCTATGATTGAAAAAAGGCCTGCTGCTTTGAGAGAAGGAGACACATGTCCTGATGGACAGTCTGAAT
GTCCGACTGGAAATACATGTTGCAAACTGTCTAGTGGAGAATGGGGATGCTGTCCTCTTCCAAATGCAGT
CTGTTGCTCTGACGGAGAACACTGCTGTCCATCAGGA

Genomic data:
Note: There are no genomic sequence data for this species at this time.
No genomic sequence to show (or empty ID or entry).
No exon sequence to show (or no genomic sequence entry).
Genomic sequence note: A genomic sequence is not presented (see note 2 in header).

00010 **O. niloticus progranulin A** Short name: **O_nilA or tilapA** Date entered: 2014-03

Species: ***Oreochromis niloticus (Nile tilapia)***
Taxonomy (via NCBI): *Eukaryota; Metazoa; Chordata; Craniata; Vertebrata; Euteleostomi; Actinopterygii; Neopterygii; Teleostei; Euteleostei; Neoteleostei; Acanthomorpha; Acanthopterygii; Percomorpha; Perciformes; Labroidei; Cichlidae; African cichlids; Pseudocrenilabrinae; Oreochromini; Oreochromis*
General Comment:

Protein Sequence
MQKWVVICWAVLAVVGADVCPDGGLCKEGETCCNSPENGYGCCRYENAECCDDHIHCCPAGTVCNKALSG
CINTTLTIPWVKRTPADQPKLCKSFRMIKAYESQEDDNICPDLSRCPAEFSCLKGLTKFGCCPLTQGISC
PDGKHCCPEGHDCSSDSRACIKKEIVVTVLCSDGVSECPDGTTCCETTEGKWACCPLPKAVCCADKIHCC
SEGTTCDVEHSKCIHSSTKKEMPMWAKLPARVRAAWENQKEVPVEAVNNTGTENAPKGTTANLLPPSAND
VPCDDTSACPDGTTCCKTQEGGWACCPMPEAVCCEDFIHCCPKGKKCNLEAQTCEDGLTSVPWAKKVPAI
IKQKVEEKKVPCDHTAACPDNTTCCKTKEGRWACCRMPEAVCCNDHKHCCPNGTTCDQTSMSCNGPSGST
PMLQKVPAFTTGAPTTVQPTTESPTTSSKAEEEDKEEEGMISCDAHTSCPQLTTCCYMKKFEKWGCCPLP
EAVCCEDGSHCCPKQYKCDNSRTSCIKGEVVIPWYTKIPAITSDEAEPSSVQCEGANQCPEQTSCCKLFT
GEWGCCPLKNAVCCPDKEHCCPQGYTCDILSRSCQKLIMMQLERVPLTPVYLLEPEPQLSPTKHKDIKCD
DKTDCGHDETCCRTSATSWGCCPSPNAVCCSDMKHCCPTGYTCTKEGSCIQNSKLYWPNWHVFLANKKRA
LIV*

Transcript:

Note: Incomplete direct cDNA sequence data. Limited est sequence data available at time of entry.

Predicted sequence:
Note:
Derivation: NCBI: GI: 542182094 XM_005469021.1
GTACAGCAACATTAAGGCACCTCCTCTCCCTCCCCGTTCGGGGTGTGTTCAAGAGTATGCGGGCTATCAC
TTGTGTCATTTTGTGGCGAGACTCTTGTGAAACACCTGCTCACAACAGCCCTTCTGTTCATTTTTTTCAA
CAAAGATGCAGAAGTGGGTTGTGATCTGCTGGGCCGTCCTGGCTGTGGTTGGTGCTGATGTGTGTCCAGA
TGGAGGGCTGTGTAAAGAAGGGGAAACCTGCTGCAACAGCCCAGAAAACGGCTACGGGTGTTGCCGATAT
GAAAACGCTGAGTGCTGTGATGATCACATCCACTGCTGTCCAGCGGGCACAGTCTGTAATAAAGCCTTAT
CTGGCTGTATTAATACCACCCTGACCATCCCATGGGTGAAAAGAACCCCTGCTGATCAGCCCAAACTCTG
TAAATCCTTCAGGATGATCAAGGCCTACGAGAGTCAGGAAGACGACAACATCTGCCCCGATCTGTCACGA
TGCCCTGCTGAGTTTTCCTGTCTGAAGGGTTTGACGAAGTTTGGCTGCTGTCCTCTAACTCAGGGAATCT
CCTGCCCCGATGGCAAACACTGCTGCCCTGAGGGCCACGACTGCAGCTCAGACAGCCGCGCCTGCATCAA
AAAAGAGATCGTGGTTACAGTTCTATGCAGCGATGGCGTTTCGGAGTGCCCAGATGGTACCACCTGCTGT
GAAACAACAGAAGGCAAATGGGCATGCTGTCCACTACCAAAGGCCGTGTGCTGCGCTGACAAGATACACT
GCTGCTCTGAAGGAACCACATGTGATGTCGAGCATTCCAAATGCATTCACTCTTCTACCAAGAAAGAGAT
GCCAATGTGGGCAAAGCTCCCTGCCAGAGTGAGAGCAGCTTGGGAAAACCAGAAAGAAGTGCCAGTTGAG
GCGGTGAATAACACAGGAACAGAAAACGCCCCCAAAGGCACCACAGCCAATCTGCTTCCTCCATCTGCCA
ACGATGTTCCATGTGACGATACATCAGCCTGTCCTGATGGCACCACCTGCTGTAAAACCCAAGAAGGTGG
CTGGGCCTGCTGTCCAATGCCAGAGGCTGTTTGCTGTGAAGACTTCATACACTGTTGTCCAAAAGGGAAG
AAATGTAACTTGGAGGCTCAAACCTGTGAGGACGGCCTGACCTCTGTGCCCTGGGCCAAGAAGGTTCCTG
CAATCATAAAGCAGAAAGTAGAGGAGAAGAAAGTTCCTTGTGATCACACAGCAGCCTGTCCTGATAACAC
CACCTGCTGTAAAACGAAAGAAGGTCGCTGGGCCTGCTGTCGAATGCCAGAGGCGGTCTGCTGCAACGAT
CACAAACACTGCTGCCCGAACGGCACCACCTGTGACCAGACCTCCATGTCCTGCAATGGCCCATCAGGCT
CAACTCCCATGCTACAGAAGGTACCTGCGTTCACTACTGGAGCACCTACTACTGTGCAGCCTACAACTGA
GAGTCCAACAACAAGCTCTAAAGCGGAGGAGGAGGACAAAGAAGAAGAAGGCATGATTAGCTGTGATGCC
CACACCAGCTGCCCTCAGTTGACCACCTGCTGCTACATGAAGAAGTTTGAAAAGTGGGGCTGCTGCCCGC
TGCCTGAGGCGGTGTGCTGTGAGGATGGGAGTCACTGTTGTCCTAAACAGTATAAATGTGACAATAGCCG
CACGTCATGCATAAAAGGAGAAGTGGTGATTCCATGGTACACCAAGATTCCAGCCATTACCAGCGACGAG
GCCGAGCCCTCCTCTGTCCAGTGCGAAGGTGCAAACCAGTGTCCTGAACAAACCAGCTGCTGCAAGCTGT
TCACAGGCGAGTGGGGCTGCTGTCCACTCAAAAATGCTGTTTGCTGTCCAGATAAGGAGCACTGTTGCCC
GCAGGGTTACACCTGTGACATCTTATCCAGATCCTGCCAGAAGCTCATCATGATGCAGCTGGAGAGAGTC
CCACTGACACCGGTGTATCTACTTGAGCCTGAACCCCAGCTCAGCCCAACAAAGCACAAAGACATCAAGT
GCGATGACAAGACCGATTGCGGACATGATGAAACTTGCTGCAGGACTTCTGCCACTTCCTGGGGCTGCTG
TCCATCTCCTAACGCGGTGTGCTGCAGCGACATGAAACACTGCTGCCCGACCGGGTACACCTGCACCAAA
GAAGGCTCCTGCATCCAGAACTCCAAGCTTTACTGGCCCAACTGGCACGTGTTCCTCGCCAACAAAAAAA
GAGCCCTAATTGTGTGAAAACACCACCTCTAAACTGTTACTAGTTTTAGTTTTTGCATGTAATGCACTAA
TGGAGAAAGAATTCTACATTAATTTCAAAATGCTTTGTATTTAAATGACACTTTGAAAACGATTTGCACC
TCAGTTGCTAAGATGTTTGCAATCTTGAAGTTGAACGTTTTGGTCTGAATGCACCTAATTATATAGATGA
CCATGTGACTAATGTATTTAAGGAGTTATCTTTGTTATTCCAAGCACACTCAGTTTGTCACTTGAAACTC
TACTCCATCCACAATAGAAGACAATCGAAACACTCCACTTGTCAGTCATCTGTTTCTTTAAGACTTGCTG
CAGCTGCCTTCTGTTGAAAAGGTTATTTCTTTATCAATAATAGTCAATCAGTCAAAGCCTGATTCAATAT
CTTTCATCATTCTGAACATTCAGTCTGATCATTTAATGATTTTGTGGCGTAATTACTCATTAAATCTTGG
GTTCATAATTTTGTGCATTGGATTAGTAATCTGTGTTAATGAGGCAAAGGTGAAGAAATTCATGCTTGGG
TCATCTTACCCTGTCAAACTGTCACATTATCCATTAAATCAATAAAAAAGAAGTGACATTGTTAAAA

cDNA data:
From: NCBI est: GI: 253924610
Note: This cDNA confirms exons encoding modules 4 and 5.
GGGGAAGGTTCCTGCAATCATAAAGCAGAAGGTAGAGGTGAAGGATGTTCCATGTGACGATACATCAGCC
TGTCCTGATGGCACCACCTGCTGTAAAACCCAAGAAGGTGGCTGGGCTTGCTGTCCAATGCCAGAGGCTG
TTTGCTGTGAAGACTTCATACACTGTTGTCCAAAAGGGAAGAAATGTAACTTGGAGGCTCAAACCTGTGA
GGACGGCCTGATCTCTGTGCCCTGGGCCAAGAAGGTTCCTGCAATCATAAAGCAGAAAGTAGAGGAGAAG
AAAGTTCCTTGTGATCACACAGCAGCCTGTCCTGATAACACCACCTGCTGTAAAACGAAAGAAGGTCGCT
GGGCCTGCTGTCGAATGCCAGAGGCGGTCTGCTGCAACGATCACAAACACTGCTGCCCGAACGGCACCAC
CTGTGACCAGACCTCCATGTCCTGCAATGGCCCATCAGGCTCAACTCCCAT

From: NCBI est: GI: (253937433, 253896546, 253926090, 253922782, 253884649, 253887892, 253892436,

253915043, 253915453, 253888770)
Note: A cDNA contig of est sequences at the 3' end of the transcript confirming exons encoding the last last two modules (modules 7 and 8)
GGGGCTGTCCTAAACAGTATAAATGTGACAATAGCCGCACGTCATGCATAAAAGGGGAAGTGGTGATTCC
GTGGTACACCAAGATTCCAGCCATTACCAGCGACGAGGCCGAGCCCTCCTCTGTCCAGTGCGAAGGTGCA
AACCAGTGTCCTGAACAAACCAGCTGCTGCAAGCTGTTCACAGGCGAGTGGGGCTGCTGTCCACTCAAAA
ATGCTGTTTGCTGTCCAGATAAGGAGCACTGTTGCCCGCAGGGTTACACCTGTGACGTCTTATCCAGATC
CTGCCAGAAGCTCATCATGATGCAGCTGGAGAGAGTCCCACTGACACCGGTGTATCTACTTGAGCCTGAA
CCCCAGCTCAGCCCAACAAAGCACAAAGACATCAAGTGCGATGAACAGACCGATTGCGGACATGATGAAA
CTTGCTGCAGGACTTCTGCCACTTCCTGGGGCTGCTGTCCATCTTCTAACGCGGTGTGCTGCAGCGACAT
GAAACACTGCTGCCCGACCGGGCACACCTGCACCAAAGAAGGCTCCTGCATCCAAAACTCCAAGCTTTAC
TGGCCCAACTGGCACGTGTTCCTCGCCAACAAAAAAAGAGCCCTAATTGTGTGAAAACACCACCTCTAAA
CTGTTACTAGTTTTAGTTTTTGCATGTAATGCACTAATGGAGAAAGAATTCTACATTAATTTCAAAATGC
TTTGTATTTAAATGACACTTTGAAAACGATTTGCACCTCAGTTGCTAAGATGTTTGCAATCTTGAAGTTG
AACGTTTTGGTCTGAATGCACCTAATTATATAGATGACCATGTGACTAATGTATTTAAGGAGTTATCTTT
GTTATTCCAAGCACACTCAGTTTGTCACTTGAAACTCTACTCCATCCACAATAGAAGACAATCGAAACAC
TCCACTTGTCAGTCATCTGTTTCTTTAAGACTTGCTGCAGCTGCCTTCTGTTGAAAAGGTTATTTCTTTA
TCAATAATAGTCAATCAGTCAAAGCCTGATTCAGTATCTTTCATCATTCTGAACATTCAGTCTGATCATT
TAATGATTTTGTGGCGTAATTACTCATTAAATCTTGGGTTCATAATTTTGTGCATTGGATTAGTAATCTG
TGTTAATGAGGCAAAGGTGAAGAAATTCATGCTTGGGTCATCTTACCCTGTCAAACTGTCACATTATCCA
TTAAATCAATAAAAAAGAAGTGACATTGTTAAAAAAAAAAAAAAAAAAAAAAAAAAAAAAAAAAAAAAAA

Genomic data:
Note: Genomic data (exons). From linkage group LG4
From NCBI: GI: 537083217
Note: The coding exons are within the section 23339707-23321661
Coding exon 1 **Exon type: sn**
Note: from the start codon
ATGCAGAAGTGGGTTGTGATCTGCTGGGCCGTCCTGGCTGTGGTTGGTGCTGATGTGTGTCCAGATGGAG
GGCTGTGTAAAGAAGGGGAAACCTGCTGCAACAGCCCAGAAAACGGCTACGGGTGTTGCCGATATGAAAA
C

Coding exon 2 **Exon type: c**
GCTGAGTGCTGTGATGATCACATCCACTGCTGTCCAGCGGGCACAGTCTGTAATAAAGCCTTATCTGGCT
GTATTAATACCACCCTGACCATCCCATGGGTGAAAAGAACCCCTGCTGATCAGCCCAAACTCTGTAAA

Coding exon 3 **Exon type: n***
TCCTTCAGGATGATCAAGGCCTACGAGAGTCAGGAAGACGACAACATCTGCCCCGATCTGTCACGATGCC
CTGCTGAGTTTTCCTGTCTGAAGGGTTTGACGAAGTTTGGCTGCTGTCCTCTAACTCAG

Coding exon 4 **Exon type: c***
GGAATCTCCTGCCCCGATGGCAAACACTGCTGCCCTGAGGGCCACGACTGCAGCTCAGACAGCCGCGCCT
GCATCAAAAAAG

Coding exon 5 **Exon type: n**
AGAGATCGTGGTTACAGTTCTATGCAGCGATGGCGTTTCGGAGTGCCCAGATGGTACCACCTGCTGTGAA
ACAACAGAAGGCAAATGGGCATGCTGTCCACTACCAAAG

Coding exon 6 **Exon type: c**
GCCGTGTGCTGCGCTGACAAGATACACTGCTGCTCTGAAGGAACCACATGTGATGTCGAGCATTCCAAAT
GCATTCACTCTTCTACCAAGAAAGAGATGCCAATGTGGGCAAAGCTCCCTGCCAGAGTGAGAGCAGCTTG
GGAAAACCAGAAAG

Coding exon 7 **Exon type: j**
Note: encodes a short addition to the module joining sequence
AAGTGCCAGTTGAGGCGGTGAATAACACAGGAACAGAAAACGCCCCCAAAGGCACCACAGCCAATCTGCT
TCCTCCATCTG

Coding exon 8 **Exon type: n**
GCCAACGATGTTCCATGTGACGATACATCAGCCTGTCCTGATGGCACCACCTGCTGTAAAACCCAAGAAG

GTGGCTGGGCCTGCTGTCCAATGCCAGAG

Coding exon 9 **Exon type: cn**
GCTGTTTGCTGTGAAGACTTCATACACTGTTGTCCAAAAGGGAAGAAATGTAACTTGGAGGCTCAAACCT
GTGAGGACGGCCTGACCTCTGTGCCCTGGGCCAAGAAGGTTCCTGCAATCATAAAGCAGAAAGTAGAGGA
GAAGAAAGTTCCTTGTGATCACACAGCAGCCTGTCCTGATAACACCACCTGCTGTAAAACGAAAGAAGGT
CGCTGGGCCTGCTGTCGAATGCCAGAG

Coding exon 10 **Exon type: cn**
GCGGTCTGCTGCAACGATCACAAACACTGCTGCCCGAACGGCACCACCTGTGACCAGACCTCCATGTCCT
GCAATGGCCCATCAGGCTCAACTCCCATGCTACAGAAGGTACCTGCGTTCACTACTGGAGCACCTACTAC
TGTGCAGCCTACAACTGAGAGTCCAACAACAAGCTCTAAAGCGGAGGAGGAGGACAAAGAAGAAGAAGGC
ATGATTAGCTGTGATGCCCACACCAGCTGCCCTCAGTTGACCACCTGCTGCTACATGAAGAAGTTTGAAA
AGTGGGGCTGCTGCCCGCTGCCTGAG

Coding exon 11 **Exon type: cn**
GCGGTGTGCTGTGAGGATGGGAGTCACTGTTGTCCTAAACAGTATAAATGTGACAATAGCCGCACGTCAT
GCATAAAAGGAGAAGTGGTGATTCCATGGTACACCAAGATTCCAGCCATTACCAGCGACGAGGCCGAGCC
CTCCTCTGTCCAGTGCGAAGGTGCAAACCAGTGTCCTGAACAAACCAGCTGCTGCAAGCTGTTCACAGGC
GAGTGGGGCTGCTGTCCACTCAAAAAT

Coding exon 12 **Exon type: cn**
GCTGTTTGCTGTCCAGATAAGGAGCACTGTTGCCCGCAGGGTTACACCTGTGACATCTTATCCAGATCCT
GCCAGAAGCTCATCATGATGCAGCTGGAGAGAGTCCCACTGACACCGGTGTATCTACTTGAGCCTGAACC
CCAGCTCAGCCCAACAAAGCACAAAGACATCAAGTGCGATGACAAGACCGATTGCGGACATGATGAAACT
TGCTGCAGGACTTCTGCCACTTCCTGGGGCTGCTGTCCATCTCCTAAC

Coding exon 13 **Exon type: c**
Note: up to the stop codon
GCGGTGTGCTGCAGCGACATGAAACACTGCTGCCCGACCGGGTACACCTGCACCAAAGAAGGCTCCTGCA
TCCAGAACTCCAAGCTTTACTGGCCCAACTGGCACGTGTTCCTCGCCAACAAAAAAAGAGCCCTAATTGT
GTGA

Genomic sequence note: A genomic sequence is not presented (see note 2 in header).

00011 **O. niloticus progranulin B** Short name: **O_nilB or tilapB** Date entered: 2014-03

Species: ***Oreochromis niloticus (Nile tilapia)***
Taxonomy (via NCBI): *Eukaryota; Metazoa; Chordata; Craniata; Vertebrata; Euteleostomi; Actinopterygii; Neopterygii; Teleostei; Euteleostei; Neoteleostei; Acanthomorpha; Acanthopterygii; Percomorpha; Perciformes; Labroidei; Cichlidae; African cichlids; Pseudocrenilabrinae; Oreochromini; Oreochromis*
General Comment: Two variants, X1 and X2, have been predicted from genomic sequence data. These are minor splice variants in that the granulin modules themselves are not affected.

Protein Sequence
MTLQMGLLCLALLGLSSALVCPDGGICQGKNTCCKNTEGGYGCCPLPRAECCSDHLHCCYEGTLCDLVHE
KCVNKTVSLPWMTRLLTTPQLEEMVKAVVCPDQVSECPDGTTCCQLPDSSWGCCPLAKAVCCDDRRHCCP
EATKCDIAHLRCVSASLESFPLLVKLPARRRGNNPVSCPDGKLSCPTAFTCCLLPNGEYGCCPYPKATCC
EDHLHCCPANMECDLKHSQCRRGETVMPLMKKIPAVANDISCPDKKAFCPDLTTCCKMINDTYGCCPMPN
AVCCSDRVHCCPAGSECDLYHRTCVSTQRNPTMTLAEAATELLKVQRKVHSIPCNESVACADRSTCCKTL
KGDWACCPLPQAVCCEDHLHCCPHGTICNLKSSTCDASTGNAVTPMLGHVPVFPLLEDGNNRCDKSTTCP
GKSTCCKTASGSWACCPLLQAVCCDDHIHCCPHGQVCNLAAETCVDPWGFSPPVSWLTKVPALTLEVEDE
KCDEKTRCPWGSTCCKMNSGQWACCPLPQAVCCNDHKHCCPKGYKCNVAEGTCDKPGGLSVAWLQKIPAL
QEELGRTVSNPTRNMCDAQTSCPRDTTCCFMEKEHKWGCCPLPNAVCCKDGNHCCPSRHTCEPHHSSCSR
GSHVIPWFAKVSAVTEPGAVIDVKCDNKSSCASGTTCCKLKTGKWGCCPLVKAVCCTDHEHCCPQGYTCN
MQTGTCEKKQHYHRLSTIPQIEVLQSVKEAEDEEDVACDGRGEFRCSKRDTCCKISATEWSCCPSPRAVC
CFDSKHCCPAGYSCDLKAGGCTLQTQLTWDSWFGDRKRDTVPHGL*

Transcript:
Note: No useful cDNA est sequence data available at time of entry.

Predicted sequence:
Note:
Derivation: NCBI GI: 542230141 XM_003448705.2
TCAGGCGTCATGTGATATAAGTATTATCAGAGCCTGTTTGCTTTGTGTGTTGTGGGTGTTGCAGACGCTA
CATCAAACGGTATATGTATGTGACCGTTCAGACGAGGAGTATGCAGAAAAGTGCCCTGTGCTGATAACGA
ATAAACTCTTTCTGCCTGCTCTCAGGATTCCTGCTAATCATGACTCTGCAGATGGGGCTCTTGTGTTTGG
CCCTGCTGGGTCTGAGCTCAGCGCTGGTCTGTCCCGATGGAGGCATATGTCAGGGCAAAAACACCTGCTG
CAAGAATACTGAGGGGGGATACGGATGCTGCCCACTACCACGTGCAGAGTGCTGTTCAGACCACCTCCAC
TGCTGTTACGAGGGCACGCTGTGCGATCTGGTTCATGAGAAATGCGTCAATAAAACAGTTTCCCTGCCGT
GGATGACACGACTCCTGACCACACCTCAGCTCGAGGAGATGGTGAAGGCAGTCGTTTGTCCGGACCAGGT
GTCTGAGTGTCCGGATGGCACGACTTGCTGCCAGCTTCCTGACAGCTCCTGGGGCTGCTGCCCCTTAGCC
AAGGCGGTGTGTTGTGACGATAGGCGCCACTGCTGCCCTGAGGCAACAAAGTGTGATATCGCTCACTTGC
GGTGCGTCTCTGCCTCGCTGGAGTCCTTCCCCCTGCTGGTGAAACTACCTGCTAGAAGGAGAGGGAATAA
CCCAGTATCATGCCCTGATGGGAAACTCAGCTGCCCAACCGCTTTCACATGCTGTCTGCTACCCAACGGA
GAGTACGGCTGCTGCCCATACCCAAAGGCCACATGCTGTGAGGATCACCTTCACTGTTGCCCTGCCAACA
TGGAGTGTGACCTGAAGCACTCACAGTGTAGGCGTGGCGAGACCGTCATGCCCCTGATGAAGAAGATCCC
TGCTGTAGCCAATGACATCAGTTGTCCAGACAAGAAGGCATTTTGTCCTGATCTCACCACATGCTGCAAG
ATGATCAACGACACCTACGGCTGCTGCCCGATGCCCAATGCCGTCTGTTGTTCCGATCGCGTCCACTGCT
GCCCTGCAGGCTCAGAGTGCGACCTGTACCACAGGACCTGTGTGTCTACCCAGAGAAACCCCACCATGAC
TCTGGCAGAAGCTGCGACCGAGCTCCTTAAAGTGCAGAGAAAAGTCCATTCCATCCCCTGCAACGAGTCT
GTGGCCTGTGCTGACAGAAGCACCTGCTGTAAAACACTAAAAGGAGACTGGGCCTGCTGTCCGTTACCAC
AGGCTGTGTGCTGTGAGGACCACCTGCACTGCTGCCCCCACGGCACCATTTGTAACCTGAAAAGCTCCAC
GTGCGACGCCTCCACAGGTAACGCCGTGACTCCCATGCTTGGACACGTGCCGGTATTTCCTTTACTGGAA
GACGGTAACAACAGGTGCGACAAGTCAACGACATGTCCTGGGAAATCCACCTGCTGCAAGACTGCGAGTG
GCAGTTGGGCCTGCTGTCCGCTGCTTCAGGCTGTTTGCTGTGACGATCACATCCACTGCTGCCCTCACGG
CCAAGTCTGCAACTTGGCAGCCGAAACTTGCGTTGACCCTTGGGGCTTCTCTCCGCCCGTCTCCTGGCTG
ACAAAGGTACCAGCTCTGACCTTAGAGGTGGAGGATGAGAAATGTGACGAGAAAACCAGATGTCCGTGGG
GCTCCACCTGCTGTAAAATGAACTCTGGACAGTGGGCCTGCTGCCCTTTACCTCAGGCTGTGTGCTGCAA
CGATCACAAGCACTGCTGCCCCAAAGGCTACAAGTGCAATGTGGCCGAGGGGACCTGTGACAAACCCGGT
GGCCTCAGTGTGGCCTGGCTGCAGAAGATCCCGGCCCTGCAGGAGGAGCTCGGCCGGACTGTTTCCAATC
CAACTCGGAACATGTGCGATGCCCAGACCAGCTGTCCCAGAGACACGACCTGCTGCTTCATGGAAAAGGA
ACACAAATGGGGCTGCTGCCCTCTGCCGAACGCTGTCTGCTGCAAAGATGGAAACCACTGCTGCCCCAGC
AGACACACCTGTGAGCCCCACCACTCCTCCTGCTCCAGAGGTTCCCATGTTATACCGTGGTTCGCCAAAG
TAAGCGCTGTAACGGAGCCGGGCGCCGTGATCGACGTTAAATGTGACAACAAGAGCAGCTGCGCTTCAGG
GACGACCTGCTGTAAGCTGAAGACCGGAAAGTGGGGCTGCTGCCCGCTGGTCAAGGCTGTTTGCTGCACT
GACCATGAGCACTGCTGCCCTCAGGGCTACACCTGCAACATGCAGACAGGAACATGCGAGAAGAAACAAC
ACTACCACCGCCTCAGCACCATCCCTCAGATCGAAGTGCTACAGTCTGTGAAGGAGGCAGAAGACGAGGA
GGATGTGGCGTGTGACGGCAGGGGCGAGTTTCGCTGCTCCAAACGAGACACCTGCTGTAAGATCTCTGCC
ACGGAGTGGAGCTGCTGCCCCTCCCCCAGGGCCGTCTGCTGCTTTGACTCGAAGCACTGCTGTCCTGCTG
GGTACTCCTGCGACCTGAAGGCCGGAGGCTGCACCTTGCAGACCCAACTGACCTGGGACTCGTGGTTTGG
GGACCGAAAGAGAGACACTGTCCCCCACGGACTCTAATCCACTGAGGTGCAATGATGATCCCAATCTTTC
CGTTATATCTACTACTGGTGTTGTCTGTGGACAAGAAGGGCTTCTCATGTTTCATTTCATGGAGGTGTAT
TCAGAGTTTTTTGCACTCTTGAGTATTTTGAGTTTTGTGTGGTGGGGGGGGGCATTTGGTCCGCATATGC
AGGAAAAACATCTGTTTAATTGCTTTTTGCCATTGAAGGAATTTTAGTGAAGTTGAAGTAGATTTCAAGG
GGGAAGCAGGTCAGACCTAGCCTGGATGCTGGTTGAAAATATTTATTGCACTCCTTCAAAACCACTCCAC
ATTTAGGCTTTACAGTGCGTCTGTTAAAGGATTACTCCGGTCTGTTTCAACCTGCCGTGTTATCTGTTAA
TGTCAGATCTGTCATTAATGCACTCTGTAGTTTGCTTATAGGTAACAAATCCCATTTGAAAATAAACTTC
AATGAATTTGTCTCTTTTGAAAGTCGATTGTTTCTGCTGACTTTTTTTTTCTTTCTTTTTTCCTTCAGAG
TAGCTTTGCACGAGTGAACTTAAAATAATGAACCCACACAGGGAGCTTGGCACCTGCTCTGTTAATCCTG
TCTGAAAGTGGCTGCTTCACTCCTTTAGCTCCATGTATCTTCTGATTGGCTGCCCAAACCTTTAAACAAC
AGGTGGGTGCGGCGGGCGGAGAAAGCACTTTTAAACCTTTAAGATAGAGGAGCAGGTTTCTGTAAGCTTT
TGTACCAAGGTTGACAGTTCCTTTGAGTTTGTGGAACAAATCTTATGCATATTTTGATGGCATTTGTTGC
TACTTTCACGAAAAACTCCCTGATCATTAGTGTTTAAACGTGAGTGCACAGAGCTGGTCTACTTCTCTGG
AAGAGAATCATGAATGTACAAACTGCCTTTTTGTGAGCAACATTTCCTAAATAATCTGACTTTAACCTTT
CCTGATAGTTTATTCAGTAAAATGGCAAAGCCACAAATTAACCACTTTAACAGTGAGAAACCACAAATCT
GAACAAACTAACAAGTAGCCTTTAGATCCCCCCCAGTGTCTCTGAATTTGTTAATTTGACTGTTTCAGGC
TTAGAGGATGAAATCGAGCTGTCAGATTAGAACTGCTGGGATTGTGGGCGTAGACATTCACTTCATGGTA
AAGTGGTTTTGGTGTCTGGGGACTTGATTTTTCACTGACTCTTAAGAGTTTAAAAAAATGAAATCATACA
GCAGACTGTCAAACATTATTGTTGTACATACAAACTGAAATGTAGGCGATCCAAAGTCTGTAAAGGTGAG

AGGAAAATGACGGGACACGAACGAGCATTAAGAACAGTTTGTGCCTTCTTCAGTAAGAGTTACCTGTATC
CTTTGGAAAGTCTTTTGTTTTGTGCAGTTGTAACATTGATTTGCATCTTTGTCTTTATGGAAACGTAACA
CAGGCGCTCAGGAGAATCATGTGGAGTTACTTGTGAATTGAGACTTGGTCAGTGTCACAGTTGGTAATAT
GTTGTTTGTAGATTCAGGAGTTCCTAAAAAGCTGAACTCTCTTAAACTGAAGTCATTTCAATGATTTCTA
AATGACTGTCATGTAAAATGTTTTACAGATTGGCTCCATGTGAAAATAAAAGTTCATTTTGCTCCTGTCA

cDNA data:

cDNA sequence data: No cDNA data (or empty cDNA entry).

Genomic data:

Note: Genomic data (exons). tilapB coding exons are in gi|537083121:c24916646-24896014 Oreochromis niloticus isolate 000638D3DF linkage group LG8-24
From NCBI: GI: 537083121 (c24916646-24896014)

Coding exon 1 **Exon type: sn**
Note: From start codon
ATGGGGCTCTTGTGTTTGGCCCTGCTGGGTCTGAGCTCAGCGCTGGTCTGTCCCGATGGAGGCATATGTC
AGGGCAAAAACACCTGCTGCAAGAATACTGAGGGGGGATACGGATGCTGCCCACTACCACGT

Coding exon 2 **Exon type: c**
GCAGAGTGCTGTTCAGACCACCTCCACTGCTGTTACGAGGGCACGCTGTGCGATCTGGTTCATGAGAAAT
GCGTCAATAAAACAGTTTCCCTGCCGTGGATGACACGACTCCTGACCACACCTCAG

Coding exon 3 **Exon type: n**
CTCGAGGAGATGGTGAAGGCAGTCGTTTGTCCGGACCAGGTGTCTGAGTGTCCGGATGGCACGACTTGCT
GCCAGCTTCCTGACAGCTCCTGGGGCTGCTGCCCCTTAGCCAAG

Coding exon 4 **Exon type: c**
GCGGTGTGTTGTGACGATAGGCGCCACTGCTGCCCTGAGGCAACAAAGTGTGATATCGCTCACTTGCGGT
GCGTCTCTGCCTCGCTGGAGTCCTTCCCCCTGCTGGTGAAACTACCTGCTAGAAGGAGAGGGAATAACCC
AG

Coding exon 5 **Exon type: n**
TATCATGCCCTGATGGGAAACTCAGCTGCCCAACCGCTTTCACATGCTGTCTGCTACCCAACGGAGAGTA
CGGCTGCTGCCCATACCCAAAG

Coding exon 6 **Exon type: c**
GCCACATGCTGTGAGGATCACCTTCACTGTTGCCCTGCCAACATGGAGTGTGACCTGAAGCACTCACAGT
GTAGGCGTGGCGAGACCGTCATGCCCCTGATGAAGAAGATCCCTGCTGTAGCCAATGACA

Coding exon 7 **Exon type: n**
TCAGTTGTCCAGACAAGAAGGCATTTTGTCCTGATCTCACCACATGCTGCAAGATGATCAACGACACCTA
CGGCTGCTGCCCGATGCCCAAT

Coding exon 8 **Exon type: c**
GCCGTCTGTTGTTCCGATCGCGTCCACTGCTGCCCTGCAGGCTCAGAGTGCGACCTGTACCACAGGACCT
GTGTGTCTACCCAGAGAAACCCCACCATGACTCTGGCAGAAGCTGCGACCGAGCTCCTTAAAGTGCAGAG
AAAAG

Coding exon 9 **Exon type: n**
TCCATTCCATCCCCTGCAACGAGTCTGTGGCCTGTGCTGACAGAAGCACCTGCTGTAAAACACTAAAAGG
AGACTGGGCCTGCTGTCCGTTACCACAG

Coding exon 10 **Exon type: cn**
GCTGTGTGCTGTGAGGACCACCTGCACTGCTGCCCCCACGGCACCATTTGTAACCTGAAAAGCTCCACGT
GCGACGCCTCCACAGGTAACGCCGTGACTCCCATGCTTGGACACGTGCCGGTATTTCCTTTACTGGAAGA
CGGTAACAACAGGTGCGACAAGTCAACGACATGTCCTGGGAAATCCACCTGCTGCAAGACTGCGAGTGGC
AGTTGGGCCTGCTGTCCGCTGCTTCAG

Coding exon 11 **Exon type: cn**
GCTGTTTGCTGTGACGATCACATCCACTGCTGCCCTCACGGCCAAGTCTGCAACTTGGCAGCCGAAACTT
GCGTTGACCCTTGGGGCTTCTCTCCGCCCGTCTCCTGGCTGACAAAGGTACCAGCTCTGACCTTAGAGGT

GGAGGATGAGAAATGTGACGAGAAAACCAGATGTCCGTGGGGCTCCACCTGCTGTAAAATGAACTCTGGA
CAGTGGGCCTGCTGCCCTTTACCTCAG

Coding exon 12 **Exon type: cn**
GCTGTGTGCTGCAACGATCACAAGCACTGCTGCCCCAAAGGCTACAAGTGCAATGTGGCCGAGGGGACCT
GTGACAAACCCGGTGGCCTCAGTGTGGCCTGGCTGCAGAAGATCCCGGCCCTGCAGGAGGAGCTCGGCCG
GACTGTTTCCAATCCAACTCGGAACATGTGCGATGCCCAGACCAGCTGTCCCAGAGACACGACCTGCTGC
TTCATGGAAAAGGAACACAAATGGGGCTGCTGCCCTCTGCCGAAC

Coding exon 13 **Exon type: cn**
GCTGTCTGCTGCAAAGATGGAAACCACTGCTGCCCCAGCAGACACACCTGTGAGCCCCACCACTCCTCCT
GCTCCAGAGGTTCCCATGTTATACCGTGGTTCGCCAAAGTAAGCGCTGTAACGGAGCCGGGCGCCGTGAT
CGACGTTAAATGTGACAACAAGAGCAGCTGCGCTTCAGGGACGACCTGCTGTAAGCTGAAGACCGGAAAG
TGGGGCTGCTGCCCGCTGGTCAAG

Coding exon 14 **Exon type: cn**
GCTGTTTGCTGCACTGACCATGAGCACTGCTGCCCTCAGGGCTACACCTGCAACATGCAGACAGGAACAT
GCGAGAAGAAACAACACTACCACCGCCTCAGCACCATCCCTCAGATCGAAGTGCTACAGTCTGTGAAGGA
GGCAGAAGACGAGGAGGATGTGGCGTGTGACGGCAGGGGCGAGTTTCGCTGCTCCAAACGAGACACCTGC
TGTAAGATCTCTGCCACGGAGTGGAGCTGCTGCCCCTCCCCCAGG

Coding exon 15 **Exon type: c**
Note: up to stop codon
GCCGTCTGCTGCTTTGACTCGAAGCACTGCTGTCCTGCTGGGTACTCCTGCGACCTGAAGGCCGGAGGCT
GCACCTTGCAGACCCAACTGACCTGGGACTCGTGGTTTGGGGACCGAAAGAGAGACACTGTCCCCCACGG
ACTCTAA

Genomic sequence note: A genomic sequence is not presented (see note 2 in header).

00012 **O. niloticus progranulin C1** Short name: **O_nilC1 or tilapC1** Date entered: 2014-03

Species: ***Oreochromis niloticus (Nile tilapia)***
Taxonomy (via NCBI): *Eukaryota; Metazoa; Chordata; Craniata; Vertebrata; Euteleostomi; Actinopterygii; Neopterygii; Teleostei; Euteleostei; Neoteleostei; Acanthomorpha; Acanthopterygii; Percomorpha; Perciformes; Labroidei; Cichlidae; African cichlids; Pseudocrenilabrinae; Oreochromini; Oreochromis*
General Comment: Four Nile tilapia small form sequences are in the database. All within 56 kB on GI: 537082957 Oreochromis niloticus isolate 000638D3DF linkage group LG22.

Protein Sequence
MSRLTLWLSVGVFMWGFASGLITCPDGSTCSDIDTCCKTERGYGCCQYPKAVCCADMLHCCPSGYRCNLV
TQLCEKLDQPWLSIPMVKKDAAQKLTYPFLYKPALPSVPAFVIPMSEDTESMNEIPEPALTHVGSPEAQV
IRCDSLHYCQGGMSCCQASTGQWNCCPYPLGQCCADGLHCCEYGYTCDASSLKCRNSYS*

Transcript:
Note: No useful cDNA data.

Predicted sequence:
Note:
Derivation: NCBI: XM_003457268.2 GI:542252113
ACAGAAGTGCTGTGATGTGAAGTCAAGCTCTCAGCTGCTCCTCAGTGGGCGGGTCTTACCTGCACTATAA
AAAGCCATGCAAGTTTCAGTGATAGGGGAAGTGTGAAATATATGACAGGAAACTGTTGACTCTCTTCCCA
ATCTGAAGATGTCGAGGCTCACTCTGTGGTTGTCAGTCGGGGTGTTTATGTGGGGATTTGCTTCTGGCTT
AATCACGTGTCCTGATGGGAGTACTTGCTCAGACATCGATACCTGCTGCAAGACTGAGCGTGGATATGGC
TGCTGTCAGTATCCAAAAGCGGTGTGCTGCGCAGACATGCTCCACTGCTGTCCGTCAGGGTATCGCTGTA
ACCTGGTGACACAGCTGTGTGAGAAACTGGACCAGCCGTGGCTGAGCATTCCCATGGTGAAGAAGGATGC
TGCACAGAAACTCACATATCCTTTCCTCTATAAGCCAGCGCTGCCTTCAGTACCTGCCTTTGTCATCCCT
ATGTCAGAGGACACAGAGAGCATGAATGAGATACCAGAGCCAGCTCTAACACACGTCGGCTCCCCTGAGG
CCCAAGTCATTCGCTGTGATTCCCTACATTACTGCCAGGGGGGGATGTCTTGCTGCCAGGCATCCACAGG
CCAGTGGAACTGCTGTCCCTACCCACTGGGCCAGTGTTGTGCAGATGGTCTGCACTGCTGTGAGTATGGA
TACACCTGTGATGCCTCCTCCTTGAAATGCAGAAATTCTTACTCTTGAGATTTCTCAGGGCTGCACGAAT

GATGTTGAAACACGATGCTGAATGTTGAAACTGCACTGTAACAGTTTAGATCTGTTTTTATATTTTTTTA
AAATTTTTTAAATACTGTTACTTCAAGATTTGTCAAGATTTTGCTGAAAATTATTACACACACTGCCACA
TCTGAGATAGAAGCCGATCAGCCTCACAAGAAGACAGCGAGCGGGCAATTGATGTTATATCCACGTAAGA
TTACTTTAAGTATTCGTTGGCTGTTTTGTGTTTAAATTGATGCTGCTGCGTCACTGGACAGTCACCATTT
AACTTCACAAATTAGCTTCAAAAGATTTCCTGCAACCATTTATTTCCACTGAGGTCATGTTGAAAATGTC
TAATGTCTGGATTCTTTTCATAAACTGAAAAATCAAAGGCTTTTCTGCTATTTTCAAGTCAAATTGTGGC
GTTTTTGTATTGAAAGTTAAAAACATAAGAATATTTCCAAATAAAAATGATAGAAATGTGAGTTTTGATG
CCGCAAAATGTTGGTGTGTTGGTGTGACTGGTTGCAAGGTCAGACTGCCACATAAAGAAGTGGAGGACTG
GAAGGTGAAAGCTTTTTAAAAAAAATTTATTCATTTTTGTGTGTTTTATTCTTTCTTTTACTTTATTTGT
CTCCATTAACAATATAACTTGCTGACCACCTGGATATAGCAGCAGACATGCAGAGTTTGATACAAATCAG
GCACCACAGCAGGGTTTCTGCGGGTGGCAGCAGATTTAGAATTATGGAGAAATGAAGAAGCTGATGGTGC
AACACACTTAGATGAGGAAAGACGGTAACACAATAGTAATGGAGGAGGAATGGTAAAGTTATGGTAAGAA
AAAAAAAATAGGGAAGTGAAACAAAGACAGGCTCCATTTTACAGCCAACACTCAAAAACATGTCAAAAGG
AAGAAGTGTGGCAATAACCAGTTAAGACTGGGTCTATATGGAGAGAAAAGTCTTACAAAGTGTAAACTAA
GATTTCCAGTGTGCAGTGATTTGTGTGCATTTTTTTTTTTTTGAGAGAGAGAGATTCATAGATTTTTATT
TTGAGCTTGGATTAAAATATAAAGACATTAAAACAGTGCTTGACCGTAGACACAGTCTTAATATAAGTTA
AACTTAGTTAAGTTCAGTGAAGTAGGGTTGGTTTGGTCTTAAAAAAAAACTACATTTATGTATATATTGCT
TACACAAGATTAATAAAACCTTAACACCATAA

cDNA data:

cDNA sequence data: No cDNA data (or empty cDNA entry).

Genomic data:

Note: Genomic data (exons).
From NCBI: NC_022219.1 GI:537082957 (25024046..25028454)
Note: NC_022219 REGION: 25024046..25028454

Coding exon 1 **Exon type: sn**
ATGTCGAGGCTCACTCTGTGGTTGTCAGTCGGGGTGTTTATGTGGGGATTTGCTTCTGGCTTAATCACGT
GTCCTGATGGGAGTACTTGCTCAGACATCGATACCTGCTGCAAGACTGAGCGTGGATATGGCTGCTGTCA
GTATCCAAAA

Coding exon 2 **Exon type: c**
GCGGTGTGCTGCGCAGACATGCTCCACTGCTGTCCGTCAGGGTATCGCTGTAACCTGGTGACACAGCTGT
GTGAGAAACTGGACCAGCCGTGGCTGAGCATTCCCATGGTGAAGAAGGATGCTGCACAGAAACTCACATA
TCCTTTCCTCTATAAGCCAGCGCTGCCTTCAGTACCTGCCTTTGTCATCCCTATGTCAGAGGACACAGAG
AGCATGAATGAG

Coding exon 3 **Exon type: n**
ATACCAGAGCCAGCTCTAACACACGTCGGCTCCCCTGAGGCCCAAGTCATTCGCTGTGATTCCCTACATT
ACTGCCAGGGGGGGATGTCTTGCTGCCAGGCATCCACAGGCCAGTGGAACTGCTGTCCCTACCCACTG

Coding exon 4 **Exon type: c**
GGCCAGTGTTGTGCAGATGGTCTGCACTGCTGTGAGTATGGATACACCTGTGATGCCTCCTCCTTGAAAT
GCAGAAATTCTTACTCTTGA

Genomic sequence note: A genomic sequence is not presented (see note 2 in header).

00013 **O. niloticus progranulin C2** Short name: **O_nilC2 or tilapC2** Date entered: 2014-03

Species: ***Oreochromis niloticus (Nile tilapia)***
Taxonomy (via NCBI): *Eukaryota; Metazoa; Chordata; Craniata; Vertebrata; Euteleostomi; Actinopterygii; Neopterygii; Teleostei; Euteleostei; Neoteleostei; Acanthomorpha; Acanthopterygii; Percomorpha; Perciformes; Labroidei; Cichlidae; African cichlids; Pseudocrenilabrinae; Oreochromini; Oreochromis*
General Comment: Incomplete genomic data and incomplete cDNA data for this small-form progranulin gene. Four Nile tilapia small form sequences are in the database. All within 56 kB on GI: 537082957 Oreochromis niloticus isolate 000638D3DF linkage group LG22.

Protein Sequence

MLRITLCLSFGVFLWGFASCSITCPDGSTCPNTATCCKAKIGFACCPHAHAMCCADLVHCCPSGYRCNLV

TMKCQKLDQPWLTIPMVKKEAAEKPNTPELSGTPLQELKESQVPDQIKKTMVYCDSYTYCPDGTTCCRHP
KGGWLCCPYSPGKCCLDGYHCCPIGFDCDHTYTHCVREKLTYPFLRKPAVPSAPASLIP...

Transcript:

Note: Prediction from genomic data is incomplete. It stops in the second coding exon and provides a prediction up to just beyond the first granulin module. There is an EST sequence which displays an odd splice from just after the signal peptide coding to the next exon. But this cDNA sequence continues through the code for the second granulin module. It does not reach the stop codon, but is so similar to tilapC3 that we can expect just 8 more codons and a similar sn-cn-c exon structure.

Predicted sequence:

Note: Incomplete prediction based upon a combination of incomplete genomic and cDNA data. The stop codon is expected to be 8 codons beyond this prediction.
Derivation: NCBI: GI:537082957 from 25049779-25050482 and est GI:253894390.

```
ATGTTGAGGATCACTCTGTGTTTGTCATTCGGTGTGTTTCTGTGGGGATTTGCTTCATGCTCTATCACAT
GTCCTGATGGGAGTACTTGTCCAAACACCGCCACCTGCTGCAAGGCTAAAATCGGCTTTGCCTGCTGTCC
CCATGCACATGCCATGTGCTGTGCCGACCTGGTCCACTGCTGCCCTTCAGGATATCGCTGTAACCTGGTT
ACCATGAAATGTCAGAAATTAGACCAGCCGTGGCTGACCATACCCATGGTGAAGAAGGAGGCTGCGGAGA
AACCAAACACCCCTGAACTGTCTGGAACTCCACTCCAGGAGCTTAAAGAGAGCCAGGTCCCAGATCAAAT
AAAGAAAACAATGGTCTACTGTGACAGTTACACCTACTGTCCTGATGGCACTACTTGCTGCAGACACCCA
AAAGGAGGCTGGCTCTGTTGTCCCTACTCTCCTGGCAAATGTTGTCTGGATGGCTACCACTGCTGTCCAA
TTGGATTTGACTGTGACCATACCTACACGCACTGTGTGAGGGAAAAACTCACATATCCTTTCCTCCGCAA
GCCAGCAGTGCCTTCAGCACCTGCCTCCCTCATCCCA
```

cDNA data:

From: NCBI est: GI:253894390
Note: This cDNA sequence not reliable for the beginning of the transcript. Apparent splice donor artifact within the first coding exon.

```
GGGGAGGGAAAGCGTGAAAGGCATCACAGGAAGCTGTTGAATTTCTTCACTATCTAAAGATGTTGAAGAT
CACTCTTCTGTGTTTGTCATTCGGTGTGTTTTTGTGGGGATTTGCTTCATGCTCTATCACACGTCCTGAT
GCCATGTGCTGTGCCGATCTGCTCCACTGCTGCCCTTCAGGATATCGCTGTAACCTGGTTACCATGAAAT
GTCAGAAATTAGACCAGCCGTGGCTGACCATACCCATGGTGAAGAAGGAGGCTGCGGAGAAACCAAACAC
CCCTGAACTGTCTGGAACTCCACTCCAGGAGCTTAAAGAGAGCCAGGTCCCAGATCAAATAAAGAAAACA
ATGGTCTACTGTGACAGTTACACCTACTGTCCTGATGGCACTACTTGCTGCAGACACCCAAAAGGAGGCT
GGCTCTGTTGTCCCTACTCTCCTGGCAAATGTTGTCTGGATGGCTACCACTGCTGTCCAATTGGATTTGA
CTGTGACCATACCTACACGCACTGTGTGAGGGAAAAACTCACATATCCTTTCCTCCGCAAGCCAGCAGTG
CCTTCAGCACCTGCCTCCCTCATCCCA
```

Genomic data:

Note: Genomic data (exons). Incomplete: The second coding exon is interrupted by a gap of about 4 kb which would contain the rest of the gene.
From NCBI: NC_022219.1 GI:537082957 (25049779..25050574)
Note: NC_022219 REGION: 25049779..25050574
Coding exon 1 **Exon type: sn**
Note: from the start codon

```
ATGTTGAGGATCACTCTGTGTTTGTCATTCGGTGTGTTTCTGTGGGGATTTGCTTCATGCTCTATCACAT
GTCCTGATGGGAGTACTTGTCCAAACACCGCCACCTGCTGCAAGGCTAAAATCGGCTTTGCCTGCTGTCC
CCATGCACAT
```

Coding exon 2 **Exon type: cn**
Note: Incomplete. The cn exon type is expected based upon similarity with tilap3

```
GCCATGTGCTGTGCCGACCTGGTCCACTGCTGCCCTTCAGGATATCGCTGTAACCTGGTTACCATGAAAT
GTCAGAAATTAGACCAGCCGTGGCTGACCATACCCATGGTGAAGAAG...
```

Coding exon 3 **Exon type: c**
Note: Yet to be sequenced. The c exon type is expected based upon similarity with tilap3. Expected to be the final coding exon.
...

Genomic sequence note: A genomic sequence is not presented (see note 2 in header).

00014 **O. niloticus progranulin C3** Short name: **O_nilC3 or tilapC3** Date entered: 2014-03

Species: ***Oreochromis niloticus (Nile tilapia)***
Taxonomy (via NCBI): *Eukaryota; Metazoa; Chordata; Craniata; Vertebrata; Euteleostomi; Actinopterygii; Neopterygii; Teleostei; Euteleostei; Neoteleostei; Acanthomorpha; Acanthopterygii; Percomorpha; Perciformes; Labroidei; Cichlidae; African cichlids; Pseudocrenilabrinae; Oreochromini; Oreochromis*
General Comment: Four Nile tilapia small form sequences are in the database. All within 56 kB on GI: 537082957 Oreochromis niloticus isolate 000638D3DF linkage group LG22.

Protein Sequence

```
MLRITLCLSFGVFLWGFASSSITCPDGSTCSDSATCCKAKIGFGCCPFPNAMCCADLLHCCPSGYRCNLV
TMNCEKLDQPWLTIPMVKKEAAEKPAPPELPGTPLQELKESQVPDPIKSSVVYCDSYTYCPDGTTCCRHP
QGGWTCCPYSPGKCCLDGYHCCPIGFDCDRTYTHCVREKLTYPFLRKPAVPSAPASLIPTSEDKEN*
```

Transcript:

Note: EST data are good for tilapC3 except for the end of the coding sequence. That is why the predicted sequence is also presented.

Predicted sequence:

Note: The only cDNA sequence covering the end of the coding sequence has an extra G compared with the sequence predicted from the genomic data, which effects a frame shift at the fifth codon before the more reliably predicted stop.
Derivation: NCBI: GI: 525343820 NM_001279700.1

```
AACACAGGAAGCTGCTGAATTTCTTCACTGCCTAAAGATGTTGAGGATCACTCTGTGTTTGTCATTCGGT
GTGTTTCTGTGGGGATTTGCTTCATCCTCTATCACATGTCCTGATGGGAGTACTTGTTCAGACTCCGCCA
CCTGCTGCAAGGCTAAAATCGGCTTTGGCTGCTGCCCATTTCCAAATGCCATGTGCTGTGCCGATCTGCT
CCACTGCTGCCCTTCAGGATATCGCTGTAACCTGGTTACCATGAATTGTGAGAAATTAGACCAGCCGTGG
CTGACCATACCCATGGTGAAGAAGGAGGCTGCGGAGAAACCAGCCCCACCTGAACTGCCTGGAACTCCAC
TCCAGGAGCTTAAAGAGAGCCAGGTCCCAGATCCAATAAAGAGTTCAGTGGTCTACTGTGACAGTTACAC
CTACTGTCCTGATGGCACTACTTGCTGCAGACACCCACAAGGAGGCTGGACCTGTTGTCCCTACTCTCCT
GGCAAATGTTGTCTGGATGGCTACCACTGTTGTCCAATTGGATTTGACTGTGACCGCACCTACACGCACT
GTGTGAGGGAAAAACTCACATATCCTTTCCTCCGCAAGCCAGCAGTGCCTTCAGCACCTGCCTCCCTCAT
CCCAACTTCAGAGGACAAAGAAAACTAGGAGAAGGGACACAGGAAGCAGATTTTCAGCTGCTGTGACTAT
AGATGTACCTGCAACCCCTCCTGCGTTATGGTAACACATGCTGAGATGGCTTTAAAGTCCACTGTGATAG
ATTTTTTTGGTCATTTTTGTTAAAAATTGTCCTAAAATTCTTTTTGCCATGCTGAAGAATAGTACACAGG
TC
```

cDNA data:

From: NCBI est: contig from GI: (253904238, 253845176, 253882775, 253893385, 253932238, 253850426, 253904269, 253895156, 253829805, 253878523, 253836585, 253856859, 327414295, 327414093)
Note: In this cDNA contig, the last 214 bases are from only one sequence(gi:327414093).

```
GGGGAAAGTGTGAAAGGCAACACAGGAAGCTGCTGAATTTCTTCACTGCCTAAAGATGTTGAGGATCACT
CTGTGTTTGTCATTCGGTGTGTTTCTGTGGGGATTTGCTTCATCCTCTATCACATGTCCTGATGGGAGTA
CTTGTTCAGACTCCGCCACCTGCTGCAAGGCTAAAATCGGCTTTGGCTGCTGCCCATTTCCAAATGCCAT
GTGCTGTGCCGATCTGCTCCACTGCTGCCCTTCGGGATATCGCTGTAACCTGGTTACCATGAATTGTGAG
AAATTAGACCAGCCGTGGCTGACCATACCCATGGTGAAGAAGGAGGCTGCGGAGAAACCAGCCCCACCTG
AACTGCCTGGAACTCCACTCCAGGAGCTTAAAGAGAGCCAGGTCCCAGATCCAATAAAGAGTTCAGTGGT
CTACTGTGACAGTTACACCTACTGTCCTGATGGCACTACTTGCTGCAGACACCCACAAGGAGGCTGGACC
TGTTGTCCCTACTCTCCTGGCAAATGTTGTCTGGATGGCTACCACTGTTGTCCAATTGGATTTGACTGTG
ACCGCACCTACACGCACTGTGTGAGGGAAAAACTCACATATCCTTTCCTCCGCAAGCCAGCAGTGCCTTC
AGCACCTGCCTCCCTCATCCCAACTTCAGGAGGACAAGGAAAACTAGAAGGAGGGGACACAGGGAAGGCA
GGATTTTTCAGCTGCTGTGACTAATAGATGGTACCTGCAACCCCCTTCTGGCGTTATGTTACCACAATGC
TGAAAATGGGCTTTAAAGGTCCACTGGTGATAGAATTTTTTTGGGTCATTTTGNTAAAANTTGTGCCTAA
GATTCTTTTGGCCATGCTTGAAGAA
```

Genomic data:

Note: Genomic data (exons).

From NCBI: NC_022219.1 GI:537082957 (25068923..25071220)
Note: NC_022219 REGION: 25068923..25071220
Coding exon 1 **Exon type: sn**
Note: from start codon
ATGTTGAGGATCACTCTGTGTTTGTCATTCGGTGTGTTTCTGTGGGGATTTGCTTCATCCTCTATCACAT
GTCCTGATGGGAGTACTTGTTCAGACTCCGCCACCTGCTGCAAGGCTAAAATCGGCTTTGGCTGCTGCCC
ATTTCCAAAT

Coding exon 2 **Exon type: cn**
GCCATGTGCTGTGCCGATCTGCTCCACTGCTGCCCTTCAGGATATCGCTGTAACCTGGTTACCATGAATT
GTGAGAAATTAGACCAGCCGTGGCTGACCATACCCATGGTGAAGAAGGAGGCTGCGGAGAAACCAGCCCC
ACCTGAACTGCCTGGAACTCCACTCCAGGAGCTTAAAGAGAGCCAGGTCCCAGATCCAATAAAGAGTTCA
GTGGTCTACTGTGACAGTTACACCTACTGTCCTGATGGCACTACTTGCTGCAGACACCCACAAGGAGGCT
GGACCTGTTGTCCCTACTCTCCT

Coding exon 3 **Exon type: c**
Note: to stop codon
GGCAAATGTTGTCTGGATGGCTACCACTGTTGTCCAATTGGATTTGACTGTGACCGCACCTACACGCACT
GTGTGAGGGAAAAACTCACATATCCTTTCCTCCGCAAGCCAGCAGTGCCTTCAGCACCTGCCTCCCTCAT
CCCAACTTCAGAGGACAAAGAAAACTAG

Genomic sequence note: A genomic sequence is not presented (see note 2 in header).

00015 **O. niloticus progranulin C4** Short name: **O_nilC4 or tilapC4** Date entered: 2014-03

Species: ***Oreochromis niloticus (Nile tilapia)***
Taxonomy (via NCBI): *Eukaryota; Metazoa; Chordata; Craniata; Vertebrata; Euteleostomi; Actinopterygii; Neopterygii; Teleostei; Euteleostei; Neoteleostei; Acanthomorpha; Acanthopterygii; Percomorpha; Perciformes; Labroidei; Cichlidae; African cichlids; Pseudocrenilabrinae; Oreochromini; Oreochromis*
General Comment: Four Nile tilapia small form sequences are in the database. All within 56 kB on GI: 537082957 Oreochromis niloticus isolate 000638D3DF linkage group LG22.

Protein Sequence
MSGITLWLSVGMFVWGFASCSITCPDESLCPDDCTCCKTAHGYSCCLYPNAMCCADLRHCCPSGYRCNLV
TMKCQKLDQPWLTIPMVKKEAAEKPAPPELPGSPLQELKESRVPDQIKSSVVHCDSYTYCPDGTTCCRHP
KGGWFCCPYSPGKCCLDGYHCCPFGLDCDHTYTHCLREKLTYPFLYKPALPSAPASPIPKAKESRRANSA
*MNDLCEHGCSHSPPPSHAENSSLPLGHPRITETCLNVLWLFF

Transcript:
Note: No useful cDNA data.

Predicted sequence:
Note:
Derivation: NCBI: XM_005462011.1 GI:542252117
GAGAAGCTGCTGAAGCTCTTCACTTGCTGAAGATGTCGGGGATCACTCTGTGGCTGTCAGTTGGTATGTT
TGTGTGGGGATTTGCTTCATGCTCCATCACATGTCCTGATGAGAGTCTTTGTCCAGATGACTGCACCTGC
TGCAAGACTGCCCATGGATATAGCTGCTGTCTATATCCAAATGCCATGTGCTGTGCTGATCTGCGCCACT
GCTGCCCTTCGGGATATCGCTGTAACCTGGTTACCATGAAATGTCAGAAATTAGACCAGCCGTGGCTGAC
CATACCCATGGTGAAGAAGGAGGCTGCGGAGAAACCAGCCCCACCTGAACTGCCTGGATCTCCACTCCAG
GAGCTTAAAGAGAGCCGCGTCCCAGATCAAATAAAGAGTTCAGTGGTCCACTGTGACAGTTACACCTACT
GTCCTGATGGCACTACCTGCTGCAGACACCCAAAAGGAGGCTGGTTCTGTTGTCCCTACTCTCCCGGGAA
ATGTTGTCTCGATGGATACCACTGCTGTCCATTTGGATTGGACTGTGATCACACCTACACGCACTGTCTG
AGGGAAAAACTCACATATCCCTTCCTCTATAAGCCAGCGCTGCCTTCAGCACCTGCTTCTCCCATCCCAA
AGGCAAAGGAAAGTAGGAGGGCCAATTCTGCGTAGATGAACGATCTCTGTGAGCATGGATGTAGCCACAG
CCCTCCTCCTTCTCATGCAGAAAACTCGAGCCTACCCTTGGGACATCCTAGGATCACAGAAACATGCCTA
AATGTGCTGTGGCTATTCTTTTGAATAGTTTTTTCTTTTATAAGAGTTTCCTATTATTATTATTATGGTC
AGAAATCTGTTGAAATGCATTTTGCATCACATCTGATATATAATCAGATCAGTCTTGGGAGAAGACAGTT
CCTGGACAAATGATCTGACAAGTGCACAAGATTGTTTTTAATGTTTGCTGTTTCTTTGCTTTTAGACTAA
TGGGTGTGCATAGTTGGCTGTTGCAATGGACAGTCACCAGTCATCCTAACAAACCAGCTTTGCAAGATTT

CCTGCTGCCTGCGTAACTAGATATCACTGAATGTAGACTAGAAAATAAAATGTAAGGCTGAATGATTATT
AAAATGTCTTTATTGTTTCTTTCTTTGTTTGAATGTTTTCTATCTTGTGTCACCAATAAAGACCTGTTGT
GACCTGTGTAAA

cDNA data:

cDNA sequence data: No cDNA data (or empty cDNA entry).

Genomic data:

Note: Genomic data (exons).
From NCBI: NC_022219.1 GI:537082957 (25077392..25079923)
Note: NC_022219 REGION: 25077392..25079923
Coding exon 1 **Exon type: sn**
Note: from start codon
ATGTCGGGGATCACTCTGTGGCTGTCAGTTGGTATGTTTGTGTGGGGATTTGCTTCATGCTCCATCACAT
GTCCTGATGAGAGTCTTTGTCCAGATGACTGCACCTGCTGCAAGACTGCCCATGGATATAGCTGCTGTCT
ATATCCAAAT

Coding exon 2 **Exon type: cn**
GCCATGTGCTGTGCTGATCTGCGCCACTGCTGCCCTTCGGGATATCGCTGTAACCTGGTTACCATGAAAT
GTCAGAAATTAGACCAGCCGTGGCTGACCATACCCATGGTGAAGAAGGAGGCTGCGGAGAAACCAGCCCC
ACCTGAACTGCCTGGATCTCCACTCCAGGAGCTTAAAGAGAGCCGCGTCCCAGATCAAATAAAGAGTTCA
GTGGTCCACTGTGACAGTTACACCTACTGTCCTGATGGCACTACCTGCTGCAGACACCCAAAAGGAGGCT
GGTTCTGTTGTCCCTACTCTCCC

Coding exon 3 **Exon type: c**
Note: to stop codon
GGGAAATGTTGTCTCGATGGATACCACTGCTGTCCATTTGGATTGGACTGTGATCACACCTACACGCACT
GTCTGAGGGAAAAACTCACATATCCCTTCCTCTATAAGCCAGCGCTGCCTTCAGCACCTGCTTCTCCCAT
CCCAAAGGCAAAGGAAAGTAGGAGGTAG

Genomic sequence note: A genomic sequence is not presented (see note 2 in header).

00038 **P. lividus progranulin** Short name: **P_liv** Date entered: 2014-03

Species: ***Paracentrotus lividus (common urchin)***
Taxonomy (via NCBI): *Eukaryota; Metazoa; Echinodermata; Eleutherozoa; Echinozoa; Echinoidea; Euechinoidea; Echinacea; Echinoida; Echinidae; Paracentrotus.*
General Comment: With still only partial EST cDNA data and no genomic data, this species was included in the analysis to test how representative S. purpuratus was for sea urchin progranulin. The EST data provide the beginning of the coding sequence, then some middle repeat sequence, and a contig of 11 EST sequences corresponds with S_pur_22 to p, except that a full module is in place of the paragranulin.

Protein Sequence

Protein sequence not yet prepared. May be too incomplete to show.

Transcript:

Note: Incomplete. Three disconnected fragments. beginning of the coding sequence but no end at this time.
The cDNA data define the transcript well (below), so no predicted sequence is required.

cDNA data:

From: NCBI est: AM512207.1 GI:139272915
Note: This cDNA fragment encodes a signal peptide, one full granulin module, and most of a second module.
GTCGTCTAAGATATAGGATTTAGGCCATTCACGATTCGACTGTGAGCTGTGTGAACCATAACTACCGATT
GATGTTGGTGGTTGATTGAAATTACAAAGCTAAGACAGGAGCAAAATTTGCGCTGAGATAGGGTTTTCTT
TCTCACTCAGTCACTGTTATTCTGTGTGGCTTGCCTGCAGACAGTGAAAAAGGATGAAGCTGGTGGTTAT
CATTGTGGCTTCTCTGGTGTCGCTGGCACTTGGAGGACCCCTGAAAATATCAGAGGAACCATCCTCATTG
GTGGAGAAACTTACCAAAGCTACAGTTTGCAAAAGTGGAAAGACATGTGGTGCTACCTCGACTTGCTGTG
ATGGCTTGAACGATGAAACCGGATGCTGTGCTTACCCTAATGCAGTGTGCTGCTCTGATGGTCGTCATTG
TTGTCCTGCTGGATATGAATGTGATGTCATCAACAACATTTGCAAGAGCAGAGAAAGTGTTAAGTTTATG

GCCCATGTAACAAATGGTGAATCTAATGTGATTTGCCCTGGTGGACAGTCTGAATGTCCAGACGGGAATA
CATGTTGCAGACTTCCTACTGGTCAGTACGGTTGCTGTCCTCTACCAAACGCAGTTTGTTGTAGTGATGG
CGAGCACTGCTGTCCATCCGGTTTAACTTGGTCGTCTAAGGTCGTCTAAGGTT

From: NCBI est: AM599110.1 GI:139242821
Note: This cDNA fragment encodes 2 full granulin modules between most of a C-half and about 70% of another module. It is part of the repeat.
TGTTGTAGTGATGGTGAGCACTGCTGTCCATCAGGCTATACTTGTGATCCATCAGCTGGAACTTGTTCAA
GAGGGGAGGATGTTGTGCTGTGGTCTGAAAAGTCCTCTGCTACTCCTGTACATTTGGGAAGTGTAATCTG
TCCTGGTGGACAGGCTGAATGTCCAGATGGGAATACATGTTGCAGACTTCCCTCTGGTCAATATGGTTGT
TGTCCTCTACCAAATGCAGTGTGTTGTAGTGATGGCGAGCACTGCTGTCCATCAGGTTATACTTGCGATG
TATCAGCTGGAACTTGTTCACAAGGGGAGGATGTTGTGTTGTGGTCTGAAAAGTCCTCTGCAACTCCTGC
GCATTTGGAAAATGTAGTATGTCCTGGAGGACAGGCTCAATGTCCAGATGGGAATACATGTTGTAGACTT
CCTACTGGTCAGTACGGTTGTTGTCCTCTACCAAATGCAGTGTGTTGTAGTGATGGCGAGCACTGCTGTC
CATCAGGTTATACTTGCGATGTATCAGCTGGAACTTGTTCAAGAGGGGAGGATATTGTGTTGTGGTCTGA
AAAGTCCTCTGCCACTCCTGTGCATTTGGAAAATGTAGTCTGTCCTGGAGGACAGTCTCAATGTCCAGAT
GGGAATACATGTTGCAGACTTCCTACTGGTCAGTATGGTTGTTGTCCTCCTCCAAACGCAGTGTGTTGTA
GTGATGGCGAGCACTGCTGTTGTTGTAGTGTGTTGTAGTGTGTTGTAGTGGG

From: NCBI est: Contig of GI:(139242821, 138486394, 139301968, 139229200, 139349007, 139362091, 139309851, 139303794, 138476016, 138480889, 139275101, 89472664)
Note: This cDNA fragment encodes most of a C-half and 5 full modules corresponding to the S. purpuratus sequence near the end of the large repeat (exon 2) through exons 3 and 4. The N-half of the last module encoded in this contig corresponds to the S_pur paragranulin.
TGTTGTAGTGATGGTGAGCACTGCTGTCCATCAGGCTATACTTGTGATCCATCAGCTGGAACTTGTTCAA
AAGGGGAGGATGTTGTGTTGTGGTCTGAAAAGTCCTCTGCAACTCCTGTGCATTTGGAAAGTGTAATCTG
TCCTGGTGGACAGGCTGAATGTCCAGATGGGAATACATGTTGCAGACTTCCTTCGGGTCAATATGGTTGT
TGTCCTCTACCAAATGCAGTTTGTTGTAGTGATGGTGAGCACTGCTGTCCATCAGGTTATACTTGTGATG
TATCAGCTGGAACTTGTTCACAGGGGGAGGATGTTGTGTTGTGGTCTGAAAAGTCCTCTGCAACTCCTGT
GCATTTGGAAAGTGTAATCTGTCCTGGTGGACAGGCTGAATGTCCAGATGGGAATACATGTTGCAGACTT
CCTTCGGGTCAATATGGTTGTTGTCCTCTACCAAATGCAGTTTGTTGTAGTGATGGTGAGCACTGCTGTC
CATCAGGTTATACTTGTGATGTATCAGCTGGAACTTGTTCACAAGGGGAGGATGTTGTGTTGTGGTCTGA
AAAGTCCTCTGCAACTCCTGTGCATTTGGAAAGTGTAATCTGTCCTGGTGGACAGGCTGAATGTCCAGAT
GGGAATACATGTTGCAGACTTCCTTCTGGTCAATATGGTTGTTGTCCTCTACCAAATGCAGTGTGTTGTA
GTGATGGTGAGCACTGCTGTCCATCAGGTTATACTTGCGACGTATCAGCTGGCACCTGCACACAGGGTTC
CAGTATCTTGGAATGGAGTTCTAAGTCTAATCCTCACAAGAAAACCCAGAATGTTCAAAATCTTGCAACT
GTTGGATCAGTTATAATGTGCCCAGATAAGAAGACCTCATGTCCACAAGCAAACACCTGTTGCCCTCTAA
TGACATCAGGGGAATGGGGCTGCTGCCCAGTACAAAACGCTATTTGCTGCGATGACCACAAGCACTGCTG
TCCGTCAGGCTACACCTGTGGAACCAACTCCTGCCAGAAGGGCTCCAAGCACCTGCCTCTTCTCACCAAG
GCTGCTGGCCTCCTCAAACTTACATACCACAGCCAAGAAAAGCAAGAGGTCATAATTCACAACAAGAAGC
ACCTGTTATTCTCACCAGATGAACCAAAGACAGGATCTGTAATCTGCCCTGGTGGTCAATCTGAATGTCC
AGATGGAACCATCTGTTGTCTATTATCCAGCGGCCAATATGGATGTTGCCCTCTCCCTCAGGCGGTGTGC
TGTGCTGACAAAGAGCATTGCTGTCCAAATGACTATACCTGTGACCCCACCTCATCCACCTGTATGCGGG
GTATACACCATCTACCATGGTTGAGTAAAGTCCCATCAACTCCACT

Genomic data:

Note: There are no genomic data for proganulin in this species at this time.
No genomic sequence to show (or empty ID or entry).
No exon sequence to show (or no genomic sequence entry).
Genomic sequence note: A genomic sequence is not presented (see note 2 in header).

00031 **Paralabidochromis chilotes progranulin C** Short name: **P_chiC (obsolete Pc1)** Date entered: 2014-03

Species: ***Paralabidochromis chilotes (Victoria big-lipped Hap). AKA Haplochromis chilotes.***
Taxonomy (via NCBI): *Eukaryota; Metazoa; Chordata; Craniata; Vertebrata; Euteleostomi; Actinopterygii; Neopterygii; Teleostei; Euteleostei; Neoteleostei; Acanthomorpha; Acanthopterygii; Percomorpha; Perciformes; Labroidei; Cichlidae; African cichlids; Pseudocrenilabrinae; Haplochromini; Paralabidochromis.*
General Comment: Abundant EST data. No genomic data at this time.

Protein Sequence

MLRISLCLSFGVFLWGFASCSMCPDGSTCSDTATCCKAYIGYGCCPYQNAMCCADRVHCCPSGYRCNLVT
MKCEKLDQPWLTIPMVKKEAAEKPAPPELPGTPLQELKESHVPDQIKTSVVYCDSYTYCPDRTTCCRHPQ
GGWTCCPYSPGKCCLDGYHCCPIGFDCDRTYQHCVREKLTYPFLRKPALPSAPASFIPTSEDKEN*

Transcript:

Note: Transcript given as a contig of 99 est sequences from both 5' and 3' reads, so that this small transcript is very well defined.
The cDNA data define the transcript well (below), so no predicted sequence is required.

cDNA data:

From: NCBI est: Contig of 99 sequences. Representative est sequences are from the 5' end by BJ674509.1 GI:46515273, and from the 3' end by BJ681837.1 GI46524958.
Note: cDNA sequence data:
CTTTTTGTTTTTTCGGGAAAGTGTGAAAGGCATCACAGGAAGCTGCTGAATTTCTTCACTGCCTAAAGAT
GTTGAGGATCAGTCTGTGTTTGTCATTCGGTGTGTTTCTGTGGGGATTTGCTTCATGCTCTATGTGTCCT
GATGGGAGTACTTGTTCAGACACCGCCACCTGCTGCAAGGCTTATATCGGATATGGCTGCTGCCCATATC
AAAATGCCATGTGCTGTGCCGACCGGGTCCACTGCTGCCCTTCAGGATATCGCTGTAACCTGGTTACCAT
GAAATGTGAGAAATTAGACCAGCCGTGGCTGACCATACCCATGGTGAAGAAGGAAGCTGCAGAGAAACCA
GCCCCCCCTGAACTGCCTGGAACTCCACTCCAGGAGCTTAAAGAGAGCCACGTCCCAGATCAAATAAAGA
CTTCAGTGGTCTACTGTGACAGTTACACCTACTGTCCTGATCGCACTACTTGCTGCAGACACCCACAAGG
AGGCTGGACCTGTTGTCCCTACTCTCCTGGCAAATGTTGTCTGGATGGCTACCACTGCTGTCCAATTGGA
TTTGACTGTGACCGCACCTACCAGCACTGTGTGAGGGAAAAACTCACATATCCTTTCCTCCGCAAGCCAG
CACTACCTTCAGCACCTGCGTCCTTCATCCCAACTTCAGAGGACAAAGAAAACTAGAAGAAGGGACACAG
GAAGCAGATTTTCAGCTGCTGTGACTATAGATGTACCTACAACCCCTCTTGCGGTATGGTAACACATGCT
GAGATGGCTTTAAAGTCCACTGTGATAGATTTTTTTTGGTTATTTTTGTTAAAAATTGTCCTAAAATTAT
TTTTGCCATGCTGAAGAATAGTACACAGGTGTAAAAAATTGTGAAAATTAAATCTTAAGCTAATGTTACT
GCATATTTTATAATGGAGAGTCACCAATTATTGCTTTAGTAATGGAAATGTGGTTTTTATCTATTCCATT
GTCTTATATTGCCCCTGTGACTGAGTGTCAGTGAGTATAGACCAGCATGTTGAATTTAAGGTAGTAAGGT
GAAAGGCTTCTGATTTGTTTTATGGTTTCTTCCTTCCTTTGTGTACTTTTCACACTTGACCAATAAAAAT
AACATCTTCTTCCAAAAAAAACAAAAAAAAGGATCCATAATC

Genomic data:

Note: No genomic data. Expect exon structure as in other closely related cichlids like Rhamphochromis esox.
No genomic sequence to show (or empty ID or entry).
No exon sequence to show (or no genomic sequence entry).
Genomic sequence note: A genomic sequence is not presented (see note 2 in header).

00044 **Petromyzon marinus Grn-related S1** Short name: **P_marS1** Date entered: 2014-11

Species: ***Petromyzon marinus (sea lamprey)***
Taxonomy (via NCBI): *Eukaryota; Metazoa; Chordata; Craniata; Vertebrata; Cyclostomata; Hyperoartia; Petromyzontiformes; Petromyzontidae; Petromyzon.*
General Comment: An interesting Grn variety: Contains a half-module (paragranulin) at the beginning, which is most similar (by protein BLAST) to mammalian paragranulins. Then a reduced motif module with only 3-double-Cys. It is most like (via protein Blast) a module with similar motif in a platyhelminth (Echinococcus granulosus). Finally, there is a module with typical Cys motif, which has a best Blastn hit with rabbit module 4 and the second of 2 modules in a puffer fish small form (different from the ggg small form). Furthermore, in comparison with the Coelacanth A and C progranulins, the module is most similar to the repeated modules in L_chaC, again sugggesting a closer relationship with many fish small-form genes.

Protein Sequence

MALPRPLMFVLVASGLCALVSADRCPDGRFCVSRCCWNLSRYTCCWVGEGEGQGETQGALATVPAQAISA
TVSQVTSCAGSVCSANGESRCCPLSEGSCCGDGLSCCGKGSTCTTFRGLNLCLPDAEFQDPVATFLSREQ
QQQQDGCVDSSGCPAGSDCHGASGDHRGQCVSSAALTPWVEKEAALSGPVGAHVVYCGSGQYCRDGQTCC
RLATGSWGCCNIPHAICCSDGIHCCPAGHFCLTASGLCARSTLALPAVEGV*

Transcript:

Note: Very well defined by EST data. No prediction necessary.
The cDNA data define the transcript well (below), so no predicted sequence is required.

cDNA data:

From: NCBI est: Contig of GI:(51801419, 51801297, 51801208, 51801306, 51801280, 51801428, 51801407, 51801143, 51801359, 51801331, 51801443, 51801227, 51801256, 109187956, 51798656, 166894134, 109188626, 109183559, 51797424, 166886616, 166872921, 90815534, 51799995, 51800505, 51801161, 51800021, 51800020, 51801167, 51800902, 166886602, 51801347, 51801307, 116021539, 166894944, 166892750, 51801316, 51801324)
Note:

```
TTAGTGATTAGCGTGGTCGCGGCCGAGGTACGCGGGGACATGACCTGGTGAAGGTCGCCGATAAGCTGGT
GGGCTCATGCCAGCGTGACGCTTTATAAGGGACAGCAGCAGCAGCAACGCGGGGGTCGATCAGCGCTGTG
TTCTCACTTGGGCTCAGCGAGGAGGCGATCGCGATGGCTCTTCCACGTCCGCTGATGTTCGTGCTGGTGG
CGTCCGGGCTATGTGCCCTCGTGTCTGCCGACCGTTGCCCTGATGGTCGATTCTGCGTATCGCGTTGTTG
CTGGAACTTATCCCGATACACTTGCTGCTGGGTAGGAGAAGGTGAAGGCCAGGGCGAGACACAGGGGGCA
TTGGCCACAGTTCCAGCTCAGGCCATCTCTGCCACAGTCTCACAGGTCACCTCCTGCGCAGGCTCCGTCT
GCTCGGCCAATGGAGAATCTCGCTGCTGCCCCTTGTCTGAGGGCTCGTGTTGTGGTGACGGCCTGTCGTG
CTGTGGAAAAGGATCCACCTGCACCACCTTCCGAGGGCTCAACCTTTGCCTCCCCGATGCGGAATTTCAG
GACCCTGTGGCCACCTTCCTGAGCCGTGAGCAGCAGCAGCAGCAGGACGGCTGCGTGGACAGCAGCGGCT
GCCCCGCGGGGAGCGACTGCCACGGGGCGAGTGGCGACCACAGGGGGCAGTGCGTGAGCTCGGCCGCGCT
CACCCCCTGGGTGGAGAAGGAGGCGGCGCTCTCAGGCCCTGTGGGAGCTCACGTTGTGTACTGCGGGAGC
GGGCAGTACTGCCGGGACGGCCAGACCTGCTGTCGCCTCGCCACCGGCAGCTGGGGCTGCTGCAACATCC
CGCACGCGATTTGCTGCTCTGACGGGATTCACTGCTGTCCGGCTGGGCACTTCTGTCTCACGGCGTCGGG
TCTCTGCGCTCGGTCCACACTGGCACTGCCCGCTGTGGAGGGTGTCTGAGATGCGTGCCCCCCCCCCCCC
CAGTGAGCCTTAGAGTGCTCGGTGCACGAAGCAAAAAAAAACCACGCCGTGCGTTGCACCACCCCATCGA
TGTGCTTAACTTAGATGTTATGCATTCGGTATGCTTTTTACTAGCTTCTTTTATTGCAGCCATTCGCTAT
TTAAACACCTTAAAATATATATTTTTCGCTATCAGCATGATGGAATGAGACAGATAAACCATTCGGATAT
TTCCCAGAGCAGTAACAACAGTAGCAACCCTTAGGTGTTTGAGAAGAGCTCACGGAAGCAGATGTCGCAA
CACTACCTCTGAAATCTCCTAATGTGACATCTGGTCCACACATCACAGGGCAGCACAATATTCTCACATA
TTAACGGCACATTATTCAAATTAGGGGTATTGTATTAGCGTTTTGTATTATTTAATCTTCATTAAATTAT
AACCCCTGAAAGTTTAATAAGAAGCAGGTGTCGCTACAATTTCACCCACGAAGTATAATCATGATACACT
GACCAGCGCTAATAATTCAAACAGCTGAAGCTCGCACGGTGTTTTAAATATCGGGATGTGTAAATGCACT
CGTAATACGCTTAAAATAACAGGCTGATGCCAACATCCACTGATTTAATACTGTTCTTCAAAAGGGACAG
TGTTGTAATCGATGCCATTCTCTACACTGACTGTACACCTGTACTTTAATGGACGTTGTCATTTCTTTCT
GCTATTGTAAACAAAATGGCTGCAATAAACCTGTCCCCCTTTCGAAAAAAAAAAAAAAAAAAAAAAAAA
```

Genomic data:

Note: The reverse complement of gi:308016283, gb:AEFG01008630.1, has the first 3 exons near its end. Exon 4 was found in a contig of 3 wgs sequences from the NCBI trace archive. The last exon is in gi:308016284, gb:AEFG01008629.1.
From NCBI wgs: gi:308016283, gb:AEFG01008630.1
Note: First 3 coding exons are near the end of the reverse complement. There are some minor differences from the more reliable EST contig sequence.
Coding exon 1 **Exon type: sn**
Note: Also has 5'ut sequence.

```
CACATGACCTGGTGAAGGTCGCCGATAAGCTGGTGGGCTCATGCCAGCGTGACGCTTTATAAGGGACAGC
AGCAGCAGCCTGTCTGTCGATCAGCGCTGTGTTCTCACTTGGGCTCAGCGAGGAGGCGATCGCGATGGCT
CTTCCACGTCCGCTGATGTTCGTGCTGGTGGCGTCCGGGCTATGTGCCCTCGTGTCTGCCGACCGTTGCC
CTGATGGTCGATTCTGCGTATCGCGTTGTTGCTGGAACTTATCCCGATACACTTGCTGCTGGGTAGGAGA
A
```

Coding exon 2 **Exon type: n''**
Note: Encodes an incomplete N-half module which lacks the first double Cys, and the spacing of the 2 single Cys is aberrant. Some EST data show a splice 3 bases upstream adding an AAG (Lysine codon) at the beginning of this sequence.

```
GGTGAAGGCCAGGGCGAGACACAGGGGGCATTGGCCACAGTTCCAGCTCAGGCCATCTCTGCCACAGTCT
CACAGGTCACCTCCTGCGCAGGCTCCGTCTGCTCGGCCAATGGAGAATCTCGCTGCTGCCCCTTGTCTGA
G
```

Coding exon 3 **Exon type: c**

GGCTCGTGTTGTGGTGACGGCCTGTCGTGCTGTGGAAAAGGATCCACCTGCACCACCTTCCGAGGGCTCA
ACCTTTGCCTCCCCGATGCGGAATTTCAGGACCCTG

From NCBI Trace: Contig of gnl|ti|1483540875, gnl|ti|1193824538 and gnl|ti|1180101663.
Note: Note to NCBI: the trace archive is very useful at times.
Coding exon 3 **Exon type:** xn
Note: The x denotes a sequence unlike a Grn half module, but having 4 Cys in it.
TGGCCACCTTCCTGAGCCGTGAGCAGCAGCAGCAGGACGGCTGCGTGGACAGCAGCGGCTGCCCCGC
GGGGAGCGACTGCCACGGGGCGAGTGGCGACCACAGGGGGCAGTGCGTGAGCTCGGCCGCGCTCACCCCC
TGGGTGGAGAAGGAGGCGGCGCTCTCAGGCCCTGTGGGAGCTCACGTTGTGTACTGCGGGAGCGGGCAGT
ACTGCCGGGACGGCCAGACCTGCTGTCGCCTCGCCACCGGCAGCTGGGGCTGCTGCAACATCCCGCAC

From NCBI wgs: gi|308016284|gb|AEFG01008629.1|
Note: Last exon starts about 330 bases into the reverse complement.
Coding exon 4 **Exon type:** c
Note: Includes also all 3'ut.
gcgatttgctgctctgacgggattcactgctgtccggctgggcacttctgtctcacggcgtcgggtctct
gcgctcggtccacactggcactgcccgctgtggagggtgtctgagatgcgtgcccccccccccccagtg
agccttagagtgctcggtgcacgaagcaaaaaaaaacacgccgtgcgttgcgtcaccccttcgatgtgct
taacttagatgttatgcattcggtatgctttttactagcttctttattgcagccattcgctatttaaaca
ccttaaatatatattttcgctatcagcatgatggaatgagacagataaaccattcggatatttcccaga
gcagtaacaacagtagcaacccttaggtgtttgagaagagctcacggaagcagatgtcgcaacactacct
ctgaaatctcctaatgtgacatctggtccacacatcacagggcagcacaatattctcacatattaacggc
acattattcaaattaggggtattgtattagcgttttgtattatttaatcttcattaaattataacccctg
aaagtttaataagaagcaggtgtcgctacaatttcacccacgaagtataatcatgatacactgaccagcg
ctaataattcaaacagctgaagctcgcacggtgttttaaatatcgggatgtgtaaatgcactcgtaatac
gcttaaaataacaggctgatgccaacatccactgatttaatactgttcttcaaaagggacagtgttgtaa
tcgatgccattctctacactgactgtacacctgtactttaatggacgttgtcatttctttctgctattgt
aaacaaaatggctgcaataaacctgt

Genomic sequence note: A genomic sequence is not presented (see note 2 in header).

00045 **Petromyzon marinus Grn-related S2** Short name: **P_marS2** Date entered: 2014-11

Species: ***Petromyzon marinus (sea lamprey)***
Taxonomy (via NCBI): *Eukaryota; Metazoa; Chordata; Craniata; Vertebrata; Cyclostomata; Hyperoartia; Petromyzontiformes; Petromyzontidae; Petromyzon.*
General Comment: This is closely related to Pmar_S1. It encodes a paragranulin and a module lacking the first double Cys. It does not include a full Grn module.

Protein Sequence
MALPRPLMFVLVASGLCALVSADRCPDGRYCASRCCWKFSRYTCCWAGEGKGQGETQRALATVPAEAISV
KVTSCAGSVCSANGESRCCPLSEGSCCGDGLSCCGKGSTCTTFQGLNVCLPNAKFQDRGNMLL*

Transcript:
Note: Quite well defined by and EST contig.
The cDNA data define the transcript well (below), so no predicted sequence is required.

cDNA data:
From: NCBI est: Contig of GI:(51801150, 51801371, 51801396, 51801203, 51801444, 166891179)
Note:
ACGCGGGGATCAGCGCTGTGTTCTCACTTGGGCTCGGCGAGGAGGCGATCGCGATGGCTCTTCCACGTCC
GCTGATGTTCGTGCTGGTGGCGTCCGGGCTATGTGCCCTCGTGTCTGCCGACCGTTGCCCCGATGGTCAA
TACTGCGCATCGCACTGCTGCTGGAAATTTTCCCGATACACTTGCTGCTGGGCAGGAGAGGGTAAAGGCC
AGGGCGAGACACAGAGGGCATTGGCCACAGTTCCAGCTGAGGCCATCTCTGTCAAGGTCACCTCCTGCGC
AGGCTCCGTCTGCTCGGCCAACGGCGAATCTCGCTGCTGCCCCTTGTCTGAGGGCTCGTGCTGCGGTGAC
GGCCTGTCGTGCTGTGGAAAAGGATCCACCTGCACCACCTTCCAGGGGCTCAACGTTTGCCTGCCCAATG
CAAAATTCCAGGACCGTGGCAATATGCTGCTCTGACGGGATCCTCTGCTGTCCGGCTGGGCACTTCTGCA
TTGGATCTGTGCGCTCGGTCCACGCTGTGGAGGGTGTCTGAGATGTGTGCCCAGTGGGCATCACAGTGCT

GCGTGCACGAAGCACTGTGCATTGCACCCTTCGACTGCTCTTTGTGTGATTTTTTTATTTGCAGCAATTC
ACATAAAAAAACACGAATAGCACCTTGCTCTTTATAGTCTTGGTTCTTTCGGTAAAGTCAAGTAGAGGGG
AAACAATCAAGTAATAAAAATGTTGAACGATAAAATTCTCGCGACGTTTCGACTGCGGCGCGGGGCGGTC
ATCATCAGGTGTGAAGGCCACAACAAGGAAAATGTGGGAAGAAGTGGCGGTTTTTTTTGGCCCTCAA

Genomic data:

Note: The coding exons, the last including much 3'ut sequence, are in the reverse complement of gi:308016282, gb:AEFG01008631.1.
From NCBI wgs: gi:308016282, gb:AEFG01008631.1
Note: In the reverse complement.
Coding exon 1 **Exon type: sn**
Note: From the start codon.
ATGGCTCTTCCACGTCCGCTGATGTTCGTGCTGGTGGCGTCCGGGCTATGTGCCCTCGTGTCTGCCGACC
GTTGCCCCGATGGTCGATACTGCGCATCGCGCTGCTGCTGGAAATTTTCCCGATACACTTGCTGCTGGGC
AGGAGAG

Coding exon 2 **Exon type: n''**
Note: Here n'' denotes encoding of a 4 Cys variant of an N-half, which lacks the first double Cys.
GGTAAAGGCCAGGGCGAGACACAGAGGGCATTGGCCACAGTTCCAGCTGAGGCCATCTCTGTCAAGGTCA
CCTCCTGCGCAGGCTCCGTCTGCTCGGCCAACGGCGAATCTCGCTGCTGCCCCTTGTCTGAG

Coding exon 3 **Exon type: c**
GGCTCGTGCTGCGGTGACGGCCTGTCGTGCTGTGGAAAAGGATCCACCTGCACCACCTTCCAGGGGCTCA
ACGTTTGCCTGCCCAATGCAAAATTCCAGGACCGTG

Coding exon 4 **Exon type: termination and 3'ut**
Note: Has the last 5 codons then 3'ut. But it seems that frame-shifts disrupted a Grn C-half sequence. It is seen in another reading frame AICCSDGILCCPAGHFCIGS... but is lost again by mutation.
GCAATATGCTGCTCTGACGGGATCCTCTGCTGTCCGGCTGGGCACTTCTGCATTGGATCTGTGCGCTCGG
TCCACGCTGTGGAGGGTGTCTGAGATGTGTGCCCAGTGGGCATCACAGTGCTGCGTGCACGAAGCACTGT
GCATTGCACCCTTCGACTGCTCTTTGTGTGATTTTTTTATTTGCAGCAATTCACATAAAAAAACACGAAT
AGCACCTTGCTCTTTATAGTCTTGGTTCTTTCGGTAAAGTCAAGTAGAGGGGAAACAATCAAGTAATAAA
AATGTTGAACGATAAAATTCTCGCGACGTTTCGACTGC

Genomic sequence note: A genomic sequence is not presented (see note 2 in header).

00046 **Petromyzon marinus Grn-related S3** Short name: **P_marS3** Date entered: 2014-11

Species: ***Petromyzon marinus (sea lamprey)***
Taxonomy (via NCBI): *Eukaryota; Metazoa; Chordata; Craniata; Vertebrata; Cyclostomata; Hyperoartia; Petromyzontiformes; Petromyzontidae; Petromyzon.*
General Comment: Has no full module sequence. Has the same 10 Cys form (with just 3 double Cys) as found in Pmar_S1 and Pmar_S2, which shows the close relationship between these genes. The paragranulin-like beginning is less like a typical N-half module.

Protein Sequence

MAFPRALLLLLVVAGLASACPCGRRRCCVRGLNVYCCFADKGENGVMQLSMVPAQAVSPYNAHARSCTGS
VCSANGESRCCPLSEGSCCGDGKSCCGKGTTCTMYGGVNLCLPHAELSFPVATMLNREQQGGCVEDSGCP
AGSKCHGVTDDTEGQCVSSAVPIPEEVL*

Transcript:

Note: Very well defined by EST data.
The cDNA data define the transcript well (below), so no predicted sequence is required.

cDNA data:

From: NCBI est: Contig from GI:(51801314, 51801340, 51801339, 51801387, 51801216, 51801166, 51801388, 51801281, 51801330, 51801361, 51801493, 51801305, 51801409, 51801399, 51801315, 51801159, 51801169, 51801335, 51801185, 51801196, 51801323, 51801350, 51795109, 114312690, 51801234, 90816872, 90816337)
Note:

ACGCGGGGGCACACAACACGTTTCGCAACCAAGCTTTAACATCCTTTGTGAAACATTTCTTGTCTAACCG
TCCAACAAAAATTTCATCTCAGGATGCCTGCATGACTTGATTAGAGGTGCGGATTAGTTGGCTCATGTCG
CGTGGGGGGCTTTATAAGGAGGGGACCCTGCATACCCAGGGGAGCTCGCATCCTCACCTGGACTCAACGA
AGACGCAACCGTCATGGCTTTTCCTCGTGCACTCTTGCTGCTGTTGGTGGTGGCCGGGCTCGCGTCCGCT
TGCCCCTGTGGCAGAAGGAGGTGTTGCGTTCGAGGCTTGAACGTTTACTGCTGTTTTGCAGACAAGGGTG
AAAACGGAGTCATGCAGTTGAGCATGGTGCCGGCGCAAGCCGTGTCTCCCTACAACGCACACGCTCGCTC
CTGCACGGGCTCCGTGTGCTCGGCCAATGGAGAATCTCGCTGCTGCCCCTTGTCTGAGGGTTCATGCTGT
GGAGATGGGAAGTCATGCTGTGGCAAAGGGACAACCTGCACTATGTATGGAGGGGTCAACCTTTGCCTTC
CCCATGCTGAATTAAGCTTCCCAGTGGCCACGATGTTGAACCGTGAGCAACAGGGAGGCTGCGTGGAGGA
CAGCGGCTGCCCCGCGGGCAGCAAGTGTCACGGGGTGACTGACGACACCGAGGGGCAGTGCGTGAGCTCG
GCCGTGCCCATCCCTGAGGAGGTGCTGTAGTGCCATTTCCTAGGTGATGGGCACTGCTGTCAGTGATGCC
TGCTGCTCTCTGATCGATAGCAGCCTGCTTACATCGCCAGGTCTCTGTGCTTGCAACAGCAAGGCATTGC
ATGCGTACTTTCTTGATTATACATGATGCAACAGATTACACGTTACATTGGGTACACTGGCTGATCAGGT
TAACTGCAATGTGTACGTTCTTGACATTGTACACGGTACACTGCACACCATGCCTTGCAAGCTGGGACAC
TGCATTTGGCAAAGTTCTTTGATCCACATTCTGCCGGAATCTCATAATATTCCAGTTAAATAGTGGTCGA
GTAGTCATATTTACCACTTATACAAAACAGATAGAGTTCCCATCTCTTGAAAGGTGATGTGTCTTGAAGC
CTTACTGCTGAACAAATTTTTTTTTTAATGATTTCATGCCCTCA

Genomic data:

Note: First 2 coding exons are in gi:308016281, gb:AEFG01008632.1 (from about 5670). The rest of the gene is in gi:308016280, gb:AEFG01008633.1, which also has the beginning of Pmar_S4.

From NCBI wgs: gi:308016281, gb:AEFG01008632.1

Coding exon 1 **Exon type: sn'**

Note: Here the difference indicated by n' is an unusual spacing of the 2 single Cys, which are separated by a single Pro. Note also that the splice donor is AAGgcaag. Sequence starts at earliest correspondence with the cDNA sequence.

cacacaacacgtttcgcaaccaagctttaacatcctttgtgaaacatttcttgtctaaccgtccaacaaa
aatttcatctcaggatgcctgcatgacttgattagaggtgcggattagttggctcatgtcgcgtgggggg
ctttataaggaggggaccctgcatgccgaggtgagctcgcatcctcacctggactcaacgaagacgcaac
cgtcatggcttttcctcgtgcactcttgctgctgctggtggtggccgggctcgcgtccgcttgcccctgt
ggcagaaggaggtgttgcgttcgaggcttgaacgtttactgctgttttgcagacaag

Coding exon 2 **Exon type: n''**

Note: Here n'' denotes encoding of a 4 Cys variant of an N-half, which lacks the first double Cys.

ggtgaaaacggagtcatgcagttgagcatggtgccggcgcaagccgtgtctccctacaacgcacacgctc
gctcctgcacgggctccgtgtgctcggccaatggagaatctcgctgctgccccttgtctgag

From NCBI wgs: gi:308016280, gb:AEFG01008633.1

Coding exon 3 **Exon type: c**

ggttcatgctgtggagatgggaagtcatgctgtggcaaagggacaacctgcactatgtatggaggggtca
acctttgccttccccatgctgaattaagcttcccag

Coding exon 4 **Exon type: x and termination.**

Note: Last coding exon encodes a polypeptide with 4 single Cys (x). The next exon containing 3'ut sequence is 1694 downstream in this contig.

tggccacgatgttgaaccgtgagcaacagggaggctgcgtggaggacagcggctgccccgcgggcagcaa
gtgtcacggggtgactgacgacaccgaggggcagtgcgtgagctcggccgtgcccatccctgaggaggtg
ctgtagtgccatttcc

Genomic sequence note: A genomic sequence is not presented (see note 2 in header).

00047 **Petromyzon marinus Grn-related S4** Short name: **P_marS4** Date entered: 2014-11

Species: ***Petromyzon marinus (sea lamprey)***

Taxonomy (via NCBI): *Eukaryota; Metazoa; Chordata; Craniata; Vertebrata; Cyclostomata; Hyperoartia; Petromyzontiformes; Petromyzontidae; Petromyzon.*

General Comment: Distinct from Pmar_S1, but with similarities. It begins with a paragranulin, has the granulin-related module with only 3 double Cys, and ends with a module having an almost normal granulin motif. In this case, there is a triple Cys in place of the normal first double Cys, which aligns with the CCC in the plant granulin-related module, although the motif is otherwise more typical of animal granulin.

Protein Sequence

MPAWQQRLLLLLLVPCVVLVPVDADVCLDGKHCGAAQCCLYSSGWDCCRWEKIAEQSDVIPAVALLPAES
EDSIDCSGPICLHSGEPLCCPAPAGVCCTDGRACCAANNTCITVEDMHVCYPEGGWKRTGQPTSASPKQH
QEGCMDDRGCPKDKRCRGVSGSHLGQCQLSAEVNPWAKMEVALPTLMTTAAAVVTKAASLPWEKQVNSVS
CANRRYCPGDSTCCCLPAGSWGCCGVPNAVCCADGVHCCPAGHVCMEKYCMKSSEENSVLVKP*

Transcript:

Note: Only one EST found for this gene. A longer read (into poor quality data) was present in the NCBI trace archive. It was useful for discovering the genomic sequence. A predicted sequence is given based upon the genomic data in order to complete the sequence for the beginning of the first coding exon and all of the last coding exon.

Predicted sequence:

Note: Confirmed from 42 to 711 by the cDNA data.
Derivation: EST gi:109185376 and genomic sequence as given below.

atgcctgcgtggcagcagcggttgctgctgctgctgctcgtgccttgtgttgtattagtgccggtggatg
ccgatgtttgtctcgatggaaagcactgcggcgctgcccaatgctgcctctattcgtccgggtgggattg
ctgccgctgggagaagattgctgaacaaagtgacgtcattccagcagtcgctctccttcctgcggaatct
gaagactccatcgactgctccggccccatctgcttgcacagtggagaaccgctctgctgtcctgccccag
caggtgtttgctgcaccgatggaagagcatgctgcgctgcaaacaacacatgcatcactgttgaggacat
gcacgtgtgttaccctgaaggtggctggaaacgcacaggtcagccgacaagtgcaagcccaaaacagcat
caggaaggctgcatggatgacagaggctgtccgaaggacaaacgttgccggggagtgagtggcagtcatc
tgggccagtgccaactctcagccgaggtcaacccctgggcaaagatggaggtggcactgcccacccttat
gacaactgctgcagctgttgtcaccaaggctgcttccctgccctgggagaagcaagtgaacagtgtctca
tgtgcaaatcgccggtactgccccggcgacagcacatgctgctgcctccctgctggatcgtggggatgct
gcggtgtgccaaacgcggtgtgctgtgccgacggagtgcactgctgtccagctgggcacgtgtgcatgga
aaaatactgtatgaagtccagcgaggaaaattctgttttggtgaaaccataaagatttcatactggc

cDNA data:

From: NCBI est and trace archive: gi|109185376|gb|EC383207.1|EC383207, and gnl|ti|1309476743, PMAH-aab17h09.g1
Note: First 39 bases may be from vector. The additiional sequence provided by the longer read in the trace archive is in lower-case.

CGACCCACGCGTCCGCGGACGCGTGGGTGGACGCGTGGGGCCTTGTGTTGTATTAGTGCCGGTGGATGCC
GATGTTTGTCTCGATGGAAAGCACTGCGGCGCTGCCCAATGCTGCCTCTATTCGTCCGGGTGGGATTGCT
GCCGCTGGGAGAAGATTGCTGAACAAAGTGACGTCATTCCAGCAGTCGCTCTCCTTCCTGCGGAATCTGA
AGACTCCATCGACTGCTCCGGCCCCATCTGCTTGCACAGTGGAGAACCGCTCTGCTGTCCTGCCCCAGCA
GGTGTTTGCTGCACCGATGGAAGAGCATGCTGCGCTGCAAACAACACATGCATCACTGTTGAGGACATGC
ACGTGTGTTACCCTGAAGGTGGCTGGAAACGCACAGGTCAGCCGACAAGTGCAAGCCCAAAACAGCATCA
GGAAGGCTGCATGGATGACAGAGGCTGTCCGAAGGACAAACGTTGCCGGGGAGTGAGTGGCAGTCATCTG
GGCCAGTGCCAACTCTCAGCCGAGGTCAACCCCTGGGCAAAGATGGAGGTGGCACTGCCCACCCTTATGA
CAACTGCTGCAGCTGTTGTCACCAAGGCTGCTTCCCTGCCCTGGGAGAAGCAAGTGAACAGTGTCTCATG
TGCAAATCGCCGGTACTGCCCCGGCGACAGCACATGCTGCTGCCTCCCTGCTGGATCGTGGGGATGCTGC
GGTGTGCCAaacgcggtgtgctgtgccgacggagtgcactgctgtccagctgggcacgtgtgcatggaaa
atactgtatgaagtccagcgaggaaattctgttttggtgaaaccataaagattcatactggcagcctaac
ttctccaacagctacatccctatg

Genomic data:

Note: This gene starts downstream of Pmar_S3 in gi:308016280, gb:AEFG01008633.1. It continues in the reverse complement of gi:307990109, gb:AEFG01034804.1. The last coding exon was located in wgs sequences from the NCBI trace archive.
From NCBI wgs: gi:308016280, gb:AEFG01008633.1
Note: Near the end of the contig which has 2 coding exons and the last 3'ut exon for Pmar_S3 upstream.
Coding exon 1 **Exon type:** **sn**
Note: Beginning at the start codon.

atgcctgcgtggcagcagcggttgctgctgctgctgctcgtgccttgtgttgtattagtgccggtggatg
ccgatgtttgtctcgatggaaagcactgcggcgctgcccaatgctgcctctattcgtccgggtgggattg
ctgccgctgggagaag

From NCBI wgs: gi:307990109, gb:AEFG01034804.1

Note: In the reverse complement of this contig.
Coding exon 2 **Exon type: n''**
Note: Encodes a polypeptide with 2 single Cys and one double Cys, distinct from, but similar to the 4 Cys forms in S1, 2 and 3. They all have ..CCXXXX at the end to splice normally into a C-half, so it appears that the sequence including the first double Cys has been deleted. If the genomic contig is good, this exon is over 14400 downstream of exon 1.

```
attgctgaacaaagtgacgtcattccagcagtcgctctccttcctgcggaatctgaagactccatcgact
gctccggccccatctgcttgcacagtggagaaccgctctgctgtcctgccccagca
```

Coding exon 3 **Exon type: c**

```
ggtgtttgctgcaccgatggaagagcatgctgcgctgcaaacaacacatgcatcactgttgaggacatgc
acgtgtgttaccctgaaggtggctggaaacgcacag
```

Coding exon 4 **Exon type: xn'**
Note: Encodes another polypeptide with 4 single Cys (x), and then an N-half variant with a tripple Cys in place of the usual first double Cys (n').

```
gtcagccgacaagtgcaagcccaaaacagcatcaggaaggctgcatggatgacagaggctgtccgaagga
caaacgttgccggggagtgagtggcagtcatctgggccagtgccaactctcagccgaggtcaacccctgg
gcaaagatggaggtggcactgcccacccttatgacaactgctgcagctgttgtcaccaaggctgcttccc
tgccctgggagaagcaagtgaacagtgtctcatgtgcaaatcgccggtactgccccggcgacagcacatg
ctgctgcctccctgctggatcgtggggatgctgcggtgtgccaaac
```

From NCBI Trace archive: Contig of gnl|ti:(1163855881, 1289728257, 1437921831, 1483859236, 1378711347, 1209757388)
Coding exon 5 **Exon type: c**
Note: Last coding exon continuing into 3'ut as far as confirmed by cDNA data.

```
gcggtgtgctgtgccgacggagtgcactgctgtccagctgggcacgtgtgcatggaaaaatactgtatga
agtccagcgaggaaaattctgttttggtgaaaccataaagatttcatactggcagcctaacttctcaaca
gctacatccctatg
```

Genomic sequence note: A genomic sequence is not presented (see note 2 in header).

00034 **Petromyzon marinus progranulin L (Large or Long form)** Short name: **P_marL**
Date entered: 2014-03

Species: ***Petromyzon marinus (sea lamprey)***
Taxonomy (via NCBI): *Eukaryota; Metazoa; Chordata; Craniata; Vertebrata; Cyclostomata; Hyperoartia; Petromyzontiformes; Petromyzontidae; Petromyzon.*
General Comment: This large form progranulin sequence as predicted encodes 10 granulin modules. The N-terminal and 6 modules are supported by cDNA data. There are smaller granulin-related genes in this species. Preliminary analysis showed the 4 such genes we found are not typical of any fish small form, and have diverged too much to be usefully included in our current analysis.

Protein Sequence

```
MMRPAVVAALLTAVAGSIMCPDGHHCPDADTCCLSAEQGYGCCPLPAAVCCSDGLHCCPSGTVCDLAHGM
CQKGKISVPWLEKTPTFTPRNKQCPDGTSCGDDATCCPLASGQYGCCSLEDAVCCSDYLHCCPKNTMCDL
KLGKCVHGKLSIPWVSKYPSKQAASDVQCDAFTSCSDQNTCCILPSGQWGCCPLPKAVCCDDKEHCCPEG
TICNVTSQTCNHAALSLPWSTKTPALRMTGEPNDVQCDDLVGCPDGNTCCKTPGGDWACCPLVKAVCCDD
HEHCCPQGTTCNLPAQTCDQGTLSLPWSTKTPALRVAGEPNVVICDDQSSCPDGNTCCKTPGGDWACCPL
VKAVCCDDHEHCCPQGTTCNLPAQTCDQGTLSLPWSTKTPALRVAGEPNVVICDDQSSCPDGNTCCKMPS
GDWGCCPLPKAVCCDDHQHCCPEGTTCNLPVERCDKGTLSLPWSTKTPALRVAGEPNVVICDDQFSCPDG
NTCCKLPSGDWGCCPLVKAVCCDDHEHCCPQGTTCNLPAQTCDQGTLSLPWSTKTPALRVAGEPNVVICD
DQFSCPDGDTCCKMPSGDWGCCPLVKAVCCDDHEHCCPQGTTCNLPAQTCDKGALSLPWSTKTPALRVAG
EPNDVKCDDQVSCPDQTTCCKTGTGDWGCCPLVKAVCCDDHEHCCPEGTTCNVPAGSCDQGQLSLPWSSK
TPARTRLTALLPASAAARGGSVRCDSAYACPDGTTCCQLPQGQWGCCPLPLATCCSDFVHCCPSGYTCDL
AQASCIKKPHFLTWAPKAPAKARPAQVFDLL*
```

Transcript:
Note: EST data confirm the beginning and sequence for the first six of the 10 modules.

Predicted sequence:

Note: Prediction is from genomic data supported up to the 6th granulin module inclusively. Sequence is given from start codon to stop codon.

Derivation: NCBI trace gnl|ti|1154271397 and wgs AEFG01039898.1

ATGATGCGTCCGGCGGTCGTGGCCGCGCTGCTGACTGCCGTGGCCGGCAGCATCATGTGCCCCGACGGAC
ACCACTGCCCCGACGCTGACACGTGCTGCTTGTCCGCCGAGCAGGGATACGGCTGCTGCCCCCTGCCCGC
GGCCGTGTGCTGCTCGGACGGGCTGCACTGCTGCCCTTCGGGGACCGTTTGCGATTTGGCCCACGGGATG
TGCCAGAAGGGGAAAATATCCGTGCCCTGGCTGGAGAAGACGCCGACGTTTACGCCACGAAATAAGCAGT
GTCCGGACGGGACGTCGTGTGGGGACGACGCCACCTGCTGCCCGTTGGCCTCGGGACAATACGGCTGCTG
CTCCCTGGAGGACGCTGTCTGCTGCAGCGACTACCTGCACTGCTGCCCAAAAAACACGATGTGTGACTTG
AAGCTTGGAAAGTGCGTTCACGGCAAGTTGTCCATTCCGTGGGTGAGCAAGTACCCTTCCAAGCAGGCGG
CTTCTGACGTGCAGTGCGATGCCTTCACCAGCTGCTCCGATCAGAACACCTGTTGCATACTCCCGTCAGG
CCAATGGGGCTGCTGCCCTCTTCCAAAGGCCGTGTGCTGCGACGATAAGGAGCACTGCTGCCCAGAAGGC
ACGATATGCAACGTGACGTCGCAGACGTGCAACCATGCCGCCCTCAGCCTGCCCTGGAGCACCAAGACGC
CGGCCCTCAGAATGACGGGAGAGCCCAACGATGTGCAGTGCGACGATCTGGTCGGCTGTCCCGACGGGAA
CACCTGCTGCAAGACGCCGGGGGGAGACTGGGCCTGCTGCCCTCTCGTGAAGGCCGTGTGCTGCGACGAC
CACGAGCACTGCTGCCCGCAAGGCACCACGTGTAACCTGCCGGCGCAGACGTGCGATCAAGGCACCCTCA
GCCTCCCCTGGAGCACCAAGACGCCGGCCCTCAGAGTGGCGGGAGAGCCCAACGTCGTGATTTGTGACGA
TCAGTCCAGCTGTCCGGACGGGAACACGTGCTGCAAGACGCCGGGGGGAGACTGGGCCTGCTGCCCTCTT
GTGAAGGCCGTGTGCTGCGACGACCACGAGCACTGCTGCCCGCAAGGCACCACGTGTAACCTGCCGGCGC
AGACGTGCGATCAAGGCACCCTCAGCCTCCCCTGGAGCACCAAGACGCCGGCCCTCAGAGTGGCGGGAGA
GCCCAACGTCGTGATTTGTGACGATCAGTCCAGCTGTCCCGACGGGAACACGTGCTGCAAGATGCCCAGC
GGAGATTGGGGCTGCTGCCCTCTGCCAAAGGCCGTGTGCTGCGACGACCACCAGCACTGCTGCCCGGAAG
GCACCACGTGTAACCTGCCAGTTGAACGCTGCGACAAAGGCACCCTCAGCCTGCCCTGGAGCACCAAGAC
TCCGGCCCTCAGAGTGGCGGGAGAGCCGAACGTCGTGATTTGTGACGATCAGTTCAGCTGTCCCGACGGG
AACACGTGCTGCAAGTTGCCGAGCGGAGACTGGGGCTGCTGCCCTCTTGTGAAGGCCGTGTGCTGCGACG
ACCACGAGCACTGCTGCCCGCAAGGCACCACGTGTAACCTGCCGGCGCAGACGTGCGATCAAGGCACCCT
CAGCCTGCCCTGGAGCACCAAGACGCCGGCCCTCAGAGTGGCGGGAGAGCCCAACGTCGTGATTTGTGAC
GATCAGTTCAGCTGTCCTGACGGGGACACGTGCTGCAAGATGCCCAGCGGAGATTGGGGCTGCTGCCCTC
TCGTGAAGGCCGTGTGCTGCGACGACCACGAGCACTGCTGCCCGCAAGGCACCACGTGTAACCTGCCGGC
GCAGACGTGCGACAAAGGTGCCCTCAGCCTGCCCTGGAGCACCAAGACGCCGGCCCTCAGAGTGGCGGGA
GAGCCCAACGATGTGAAGTGCGACGATCAGGTCAGCTGTCCTGACCAAACCACGTGCTGCAAGACGGGAA
CCGGAGACTGGGGCTGCTGCCCTCTCGTGAAGGCCGTCTGCTGCGACGACCACGAGCACTGCTGCCCCGA
AGGCACGACGTGCAATGTGCCGGCGGGCTCGTGCGACCAGGGGCAGCTCTCCCTGCCCTGGAGCTCCAAG
ACTCCGGCCAGGACGCGTCTCACCGCGCTGCTGCCCGCGTCTGCCGCGGCGCGCGGGGGCAGCGTGCGGT
GCGACTCGGCCTACGCCTGCCCCGACGGCACCACCTGCTGCCAGCTCCCGCAGGGGCAGTGGGGCTGCTG
CCCCTTACCGCTGGCCACCTGCTGCAGCGACTTTGTGCACTGCTGCCCGAGCGGTTACACGTGTGACCTG
GCACAGGCGAGCTGCATCAAGAAGCCCCATTTCCTCACCTGGGCCCCCAAAGCCCCTGCCAAGGCCAGGC
CCGCTCAGGTCTTTGACCTGCTGTAA

cDNA data:

From: NCBI est: Contig of GI:(166881187, 83671489, 51789102, 51795078)

Note: In this cDNA contig, gi:51795078 chosen over long-range gi:83671489 to preserve the correct reading frame.

GGCAAGTGGAAGACGTCGTGGTGGTGTCGAGCCTTGAAAAACTGTCCCCCTCCTCACGTCTCCCAAGATG
ATGCGTCCGGCGGTCGTGGCCGCGCTGCTGACTGCCGTGGCCGGCAGCATCATGTGCCCCGACGGACAGC
AGTGCCCCGACGGCAACACGTGCTGCTTGTCCGCCGAGCAGGGATACGGCTGCTGCCCCCTGCCCGCGGC
CGTGTGCTGCTCGGACGGGCTGCACTGCTGCCCTTCGGGGACCGTTTGCGATTTGGCCCACGGGATGTGC
CAGAAGGGGAAAATATCCGTGCCCTGGCTGGAGAAGACGCCGACGTTTATGCCACGAAATAAGCAGTGTC
CGGACGGGACGTCGTGTGGGGACGACGCCACCTGCTGCCCGTTGGCCTCGGGACAATACGGCTGCTGCTC
CCTGGAGGACGCTGTCTGCTGCAGCGACTACCTGCACTGCTGCCCAAAAAACACGATGTGTGACTTGAAG
CTTGGAAAGTGCGTTCACGGCAAGTTGTCCATTCCGTGGGTGAGCAAGTACCCTTCCAAGCAGGCGGCTT
CTGACGTGCAGTGCGATGCCTTCACCAGCTGCTCCGATCAGAACACCTGTTGCATACTCCCGTCAGGCCA
ATGGGGCTGCTGCCCTCTTCCAAAGGCCGTCTGCTGCGACGATAAGGAGCACTGCTGCCCAGAAGGCACG
ATATGCAACGTGACGTCGCAGACGTGCAACCATGCCGCCCTCAGCCTGCCCTGGAGCACCAAGACGCCGG
CCCTCAGAATGACGGGAGAGCCCAACGATGTGCAGTGCGACGATCTGGTCGGCTGTCCCGACGGGAACAC
CTGCTGCAAGACGCCGGGGGGAGACTGGGCCTGCTGCCCTCTCGTGAAGGCCGTGTGCTGCGACGACCAC
GAGCACTGCTGCCCGCAAGGCACCACGTGTAACCTGCCGGCGCAGACGTGCGATCAAGGCACCCTCAGCC
TCCCCTGGAGCACCAAGACGCCGGCCCTCAGAGTGGCGGGAGAGCCCAACGTCGTGATTTGTGACGATCA
GTCCAGCTGTCCGGACGGGAACACGTGCTGCAAGACGCCGGGGGGAGACTGGGCCTGCTGCCCTCTTGTG

AAGGCCGTGTGCTGCGACGACCACGAGCACTGCTGCCCGCAAGGCACCACGTGTAACCTGCCGGCGCAGA
CGTGCGATCAAGGCACCATCAGCCTCCCCTGGAGCACCAAGACGCCGGCCCTCAGAGTGGCGGGAGAGCC
CAACGTCGTGATTTGTGACGATCAGTCCAGCTGTCCCGACGGGAACACGTGCTGCAAGATGCCCAGCGGA
GATTGGGGCTGCTGCCCTCTGCCAAAGGCCGTGTGCTGCGACGACCACCAGCACTGCTGCCCGGAAGGCA
CCACGTGTAACCTGCCAGTTGAACGCTGCGACAAAGGCACCCTCAGCCTGCCCTGGAACACCAAGACTCC
GGCCCCTC

Genomic data:

Note: Genomic sequences mostly in gi:307985015 gb:AEFG01039898.1. First coding exon in trace archive sequence gnl|ti|1154271397 PMAC-aan86d01.b1.

From NCBI trace: gnl|ti|1154271397 PMAC-aan86d01.b1

Coding exon 1 **Exon type: sn**

Note: from the start codon within that exon.

ATGATGCGTCCGGCGGTCGTGGCCGCGCTGCTGACTGCCGTGGCCGGCAGCATCATGTGCCCCGACGGAC
ACCACTGCCCCGACGCTGACACGTGCTGCTTGTCCGCCGAGCAGGGATACGGCTGCTGCCCCCTGCCCGC
G

From NCBI wgs: AEFG01039898.1 GI:307985015

Coding exon 2 **Exon type: c**

GCCGTGTGCTGCTCGGACGGGCTGCACTGCTGCCCTTCGGGGACCGTTTGCGATTTGGCCCACGGGATGT
GCCAGAAGGGGAAAATATCCGTGCCCTGGCTGGAGAAGACGCCGACGTTTACGCCACGAAATAAGCAG

Coding exon 3 **Exon type: n**

TGTCCGGACGGGACGTCGTGTGGGGACGACGCCACCTGCTGCCCGTTGGCCTCGGGACAATACGGCTGCT
GCTCCCTGGAGGAC

Coding exon 4 **Exon type: cn**

GCTGTCTGCTGCAGCGACTACCTGCACTGCTGCCCAAAAAACACGATGTGTGACTTGAAGCTTGGAAAGT
GCGTTCACGGCAAGTTGTCCATTCCGTGGGTGAGCAAGTACCCTTCCAAGCAGGCGGCTTCTGACGTGCA
GTGCGATGCCTTCACCAGCTGCTCCGATCAGAACACCTGTTGCATACTCCCGTCAGGCCAATGGGGCTGC
TGCCCTCTTCCAAAG

Coding exon 5 **Exon type: cn**

Note: Splice donor aag|gcgagt supported by cDNA data.

GCCGTGTGCTGCGACGATAAGGAGCACTGCTGCCCAGAAGGCACGATATGCAACGTGACGTCGCAGACGT
GCAACCATGCCGCCCTCAGCCTGCCCTGGAGCACCAAGACGCCGGCCCTCAGAATGACGGGAGAGCCCAA
CGATGTGCAGTGCGACGATCTGGTCGGCTGTCCCGACGGGAACACCTGCTGCAAGACGCCGGGGGGAGAC
TGGGCCTGCTGCCCTCTCGTGAAG

Coding exon 6 **Exon type: cn**

Note: Splice donor aag|gcgagt supported by cDNA data.

GCCGTGTGCTGCGACGACCACGAGCACTGCTGCCCGCAAGGCACCACGTGTAACCTGCCGGCGCAGACGT
GCGATCAAGGCACCCTCAGCCTCCCCTGGAGCACCAAGACGCCGGCCCTCAGAGTGGCGGGAGAGCCCAA
CGTCGTGATTTGTGACGATCAGTCCAGCTGTCCGGACGGGAACACGTGCTGCAAGACGCCGGGGGGAGAC
TGGGCCTGCTGCCCTCTTGTGAAG

Coding exon 7 **Exon type: cn**

GCCGTGTGCTGCGACGACCACGAGCACTGCTGCCCGCAAGGCACCACGTGTAACCTGCCGGCGCAGACGT
GCGATCAAGGCACCCTCAGCCTCCCCTGGAGCACCAAGACGCCGGCCCTCAGAGTGGCGGGAGAGCCCAA
CGTCGTGATTTGTGACGATCAGTCCAGCTGTCCCGACGGGAACACGTGCTGCAAGATGCCCAGCGGAGAT
TGGGGCTGCTGCCCTCTGCCAAAG

Coding exon 8 **Exon type: cn**

Note: cDNA data does not yet go beyond the middle of this exon.

GCCGTGTGCTGCGACGACCACCAGCACTGCTGCCCGGAAGGCACCACGTGTAACCTGCCAGTTGAACGCT
GCGACAAAGGCACCCTCAGCCTGCCCTGGAGCACCAAGACTCCGGCCCTCAGAGTGGCGGGAGAGCCGAA
CGTCGTGATTTGTGACGATCAGTTCAGCTGTCCCGACGGGAACACGTGCTGCAAGTTGCCGAGCGGAGAC
TGGGGCTGCTGCCCTCTTGTGAAG

Coding exon 9 **Exon type: cn**

GCCGTGTGCTGCGACGACCACGAGCACTGCTGCCCGCAAGGCACCACGTGTAACCTGCCGGCGCAGACGT

GCGATCAAGGCACCCTCAGCCTGCCCTGGAGCACCAAGACGCCGGCCCTCAGAGTGGCGGGAGAGCCCAA
CGTCGTGATTTGTGACGATCAGTTCAGCTGTCCTGACGGGGACACGTGCTGCAAGATGCCCAGCGGAGAT
TGGGGCTGCTGCCCTCTCGTGAAG

Coding exon 10 **Exon type: cn**
GCCGTGTGCTGCGACGACCACGAGCACTGCTGCCCGCAAGGCACCACGTGTAACCTGCCGGCGCAGACGT
GCGACAAAGGTGCCCTCAGCCTGCCCTGGAGCACCAAGACGCCGGCCCTCAGAGTGGCGGGAGAGCCCAA
CGATGTGAAGTGCGACGATCAGGTCAGCTGTCCTGACCAAACCACGTGCTGCAAGACGGGAACCGGAGAC
TGGGGCTGCTGCCCTCTCGTGAAG

Coding exon 11 **Exon type: cn**
GCCGTCTGCTGCGACGACCACGAGCACTGCTGCCCCGAAGGCACGACGTGCAATGTGCCGGCGGGCTCGT
GCGACCAGGGGCAGCTCTCCCTGCCCTGGAGCTCCAAGACTCCGGCCAGGACGCGTCTCACCGCGCTGCT
GCCCGCGTCTGCCGCGGCGCGCGGGGGCAGCGTGCGGTGCGACTCGGCCTACGCCTGCCCCGACGGCACC
ACCTGCTGCCAGCTCCCGCAGGGGCAGTGGGGCTGCTGCCCCTTACCGCTG

Coding exon 12 **Exon type: c**
Note: up to the stop codon.
GCCACCTGCTGCAGCGACTTTGTGCACTGCTGCCCGAGCGGTTACACGTGTGACCTGGCACAGGCGAGCT
GCATCAAGAAGCCCCATTTCCTCACCTGGGCCCCCAAAGCCCCTGCCAAGGCCAGGCCCGCTCAGGTCTT
TGACCTGCTGTAA

Genomic sequence note: A genomic sequence is not presented (see note 2 in header).

00030 **R. esox progranulin C** Short name: **R_esoC (obsolete Re1)** Date entered: 2014-03

Species: ***Rhamphochromis esox (Tigerfish)***
Taxonomy (via NCBI): *Eukaryota; Metazoa; Chordata; Craniata; Vertebrata; Euteleostomi; Actinopterygii; Neopterygii; Teleostei; Euteleostei; Neoteleostei; Acanthomorpha; Acanthopterygii; Percomorpha; Perciformes; Labroidei; Cichlidae; African cichlids; Pseudocrenilabrinae; Haplochromini; Rhamphochromis.*
General Comment: No cDNA data, but defined by genomic sequence. Typical of several African cichlid small form progranulins for which cDNA sequence data are available. Our old name for it, Re1, followed from the early Dr1, 2 foe zebrafish small forms. Now the relationship to the small fish C forms is more evident.

Protein Sequence
MSGITLWLSVGMFVWGFASCSITCPDESLCPDDCTCCKTAHGYSCCLYPNAMCCADLLHCCPSGYRCNLV
TMNCEKLDQPWLIIPMVKKEAAEKPAPPELSGTPLQELKESHVPDQIKKSVVYCDSYTYCPDGTTCCRHP
QGGWTCCPYSPGKCCLDGYHCCPIGFDCDRTYQHCVREKLTYPFLRKPALPSGPASFIPTSEDKEN*

Transcript:
Note: The prediction is well supported by cDNA data from closely related African cichlids (many of which have no genomic data).

Predicted sequence:
Note: from start codon to stop codon.
Derivation:
ATGTCGGGGATCACTCTGTGGCTGTCAGTTGGTATGTTTGTGTGGGGATTTGCTTCATGCTCCATCACAT
GTCCTGATGAGAGTCTTTGTCCAGATGACTGTACCTGCTGCAAGACTGCCCATGGATATAGCTGCTGTCT
ATATCCAAATGCCATGTGCTGCGCCGACCTGCTCCACTGCTGCCCTTCGGGATATCGCTGTAACCTGGTT
ACCATGAATTGTGAGAAATTAGACCAGCCGTGGCTGATCATACCCATGGTGAAGAAGGAGGCTGCGGAGA
AACCAGCCCCCCCTGAACTGTCTGGAACTCCACTCCAGGAGCTTAAAGAGAGCCACGTCCCAGATCAAAT
AAAGAAATCAGTGGTCTACTGTGACAGTTACACCTACTGTCCTGATGGCACTACTTGCTGCAGACACCCA
CAAGGAGGCTGGACCTGTTGTCCCTACTCTCCTGGCAAATGTTGTCTGGATGGCTACCACTGCTGTCCAA
TTGGATTTGACTGTGACCGAACCTACCAGCACTGTGTGAGGGAAAAACTCACATATCCTTTCCTCCGCAA
GCCAGCACTACCTTCAGGACCTGCGTCCTTCATCCCAACTTCAGAGGACAAAGAAAACTAG

cDNA data:
cDNA sequence data: No cDNA data (or empty cDNA entry).

Genomic data:

Note: Genomic data (exons).
From NCBI: ABPN01000900.1 GI:192381634
Note: Good sequence for exons 1 and 2 starting at base 4678
Coding exon 1 **Exon type:** sn
ATGTCGGGGATCACTCTGTGGCTGTCAGTTGGTATGTTTGTGTGGGGATTTGCTTCATGCTCCATCACAT
GTCCTGATGAGAGTCTTTGTCCAGATGACTGTACCTGCTGCAAGACTGCCCATGGATATAGCTGCTGTCT
ATATCCAAAT

Coding exon 2 **Exon type:** cn
GCCATGTGCTGCGCCGACCTGCTCCACTGCTGCCCTTCGGGATATCGCTGTAACCTGGTTACCATGAATT
GTGAGAAATTAGACCAGCCGTGGCTGATCATACCCATGGTGAAGAAGGAGGCTGCGGAGAAACCAGCCCC
CCCTGAACTGTCTGGAACTCCACTCCAGGAGCTTAAAGAGAGCCACGTCCCAGATCAAATAAAGAAATCA
GTGGTCTACTGTGACAGTTACACCTACTGTCCTGATGGCACTACTTGCTGCAGACACCCACAAGGAGGCT
GGACCTGTTGTCCCTACTCTCCT

From NCBI: ABPN01057103.1 GI:191618541
Note: This wgs sequence overlaps ABPN01000900.1 and extends it far enough to capture the last coding exon. The overlap includes coding exon 2, but is not as reliable as ABPN01000900.1 in that region.
Coding exon 3 **Exon type:** c
Note: Up to the stop codon
GGCAAATGTTGTCTGGATGGCTACCACTGCTGTCCAATTGGATTTGACTGTGACCGAACCTACCAGCACT
GTGTGAGGGAAAAACTCACATATCCTTTCCTCCGCAAGCCAGCACTACCTTCAGGACCTGCGTCCTTCAT
CCCAACTTCAGAGGACAAAGAAAACTAG

Genomic sequence note: A genomic sequence is not presented (see note 2 in header).

00048 **S. kowalevskii progranulin** Short name: **S_kow** Date entered: 2015-03

Species: ***Saccoglossus kowalevskii (***
Taxonomy (via NCBI): *Eukaryota; Metazoa; Hemichordata; Enteropneusta; Harrimaniidae; Saccoglossus.*
General Comment: The current prediction in the NCBI database, gi|585702646|ref|XM_002739948.2| is incomplete and includes an error. Separately, genomic and cDNA data are insufficient to provide a likely complete transcript sequence prediction, but combined data appear to define the full coding sequence. The first half of the gene encodes 5 nearly identical modules. Since there are large gaps in genomic sequence, it is possible that one has been missed. The cDNA sequence from EST data picks up most of the last 2 repeats, and continues to the poly-A tail. It provides some data missing in a genomic gap, and offers a convincing overlap..

Protein Sequence

MILLFITFMVLSHAIASPLYFTPSELNSMLCPDGKSQCPDGSTCCKLTSGEWGCCPMLKAVCCSDGLHCC
PNGTTCDVSRGKCNKGEFSMQWVKKTRSIPADSILCPDGKSQCPDGNTCCKLKTGEYGCCPLPKAVCCSD
GLHCCPNGTTCDVSQGKCNKGDFSMPWVKKTLATPTNSILCPDGQSQCPDGSTCCKLSTGEWGCCPLPKA
VCCSDGLHCCPSGTTCDVSQGKCNKGDFSMPWVKKTLATPTNSILCPDGQSQCPDGSTCCKLSTGQWGCC
PLPKAACCSDGLHCCPNGTTCDVSQGKCNKGDFSMPWVKKTLATPTNSILCPDGQSQCPDGSTCCKLSTG
EWGCCPLPKAVCCSDGLHCCPSGTTCDVSQGKCNKGDFSMPWVKKTLATPTNSILCPDGQSQCPDGSTCC
KLSTGQWGCCPLPKAVCCSDGLHCCPSGTTCDDSQGKCNKGDFSMPWVKKTLALRNVDVISSVNDVISSV
NDVISSVNDVISSVNDVTCKGGKQKCPDGNTCCEEASGLFGCCPFPDAVCCVDQQHCCPQGFTCDLQQTK
CIKGSLGLPLMKKTMAQNVEVVVCPGGKQECPSQMTCCSVHRDLYGCCPVPDAVCCDDKIHCCPKDYTCK
DSRCLNGDINLPMLERVSAKYTHKVESVMCPDGQSECPDGSTCCILASGQYGCCPLPKAVCCDDHQHCCP
NGYKCDTAGGSCQKGDDAIPWLKKHNSIPMSGNVKCQDGSECPSGSTCCKLPTGDYGCCPYLDAVCCNDG
LHCCPKGYTCNTKEGTCQKASINMLPWVKSSEGLVDLVTCPDKAKCPDKTTCCQLPSDKYACCPLEHAIC
CEDKIHCCPDYLTCDMPSGKCVRKSIISIPWMEKIPTLIELCDVDVCPDGLTGCLSGQTCTATTSGQYNC
CPMPNAVSCDGGEHCCPHGYACDKHQGCLEMDNVPLSSVQLP*

Transcript:

Note: Sequence assembly indicates variability in the length of a short repeat encoding DVISSVNDVISSVNDVISSVNDVISSVN after the 6th module.

Predicted sequence:

Note: Transcript prediction is from combined genomic and EST data
Derivation: Genomic WGS and transcript EST data further defined in this entry.
ATGATTTTGTTATTCATTACATTCATGGTGCTGTCGCATGCTATTGCTTCTCCTTTGTATTTTACCCCGA
GTGAATTAAACTCTATGTTATGTCCGGATGGAAAGTCTCAATGTCCAGATGGAAGTACATGTTGTAAATT
GACCAGTGGAGAATGGGGATGCTGTCCAATGCTAAAGGCAGTATGCTGCTCTGATGGTCTACATTGCTGT
CCTAATGGTACAACATGTGATGTTTCTCGGGGTAAATGTAATAAGGGTGAATTTAGTATGCAATGGGTCA
AGAAAACACGATCAATCCCCGCTGACTCTATCTTGTGTCCTGATGGTAAATCCCAATGTCCAGATGGCAA
CACGTGCTGTAAGCTGAAAACTGGAGAGTATGGGTGTTGTCCTTTACCCAAGGCTGTATGCTGCTCTGAT
GGTCTACATTGCTGTCCTAATGGTACAACATGTGATGTTTCTCAGGGTAAATGTAATAAGGGTGACTTCA
GTATGCCGTGGGTCAAGAAAACTTTAGCCACACCAACAAATTCTATATTGTGTCCTGATGGTCAGTCTCA
ATGTCCAGATGGAAGCACTTGTTGCAAACTGTCGACGGGTGAATGGGGATGTTGTCCCTTACCCAAGGCT
GTATGCTGCTCTGATGGTCTACATTGCTGTCCTAGTGGTACAACATGTGATGTTTCTCAGGGTAAATGTA
ATAAGGGTGACTTCAGTATGCCATGGGTCAAGAAAACTTTAGCCACACCAACAAATTCTATATTGTGTCC
TGATGGTCAGTCTCAATGTCCAGATGGAAGCACTTGTTGCAAACTGTCGACTGGTCAATGGGGATGTTGT
CCATTACCCAAGGCTGCATGCTGCTCTGATGGTCTACATTGCTGTCCTAATGGTACAACATGTGATGTTT
CTCAGGGTAAATGTAATAAGGGTGACTTCAGTATGCCATGGGTCAAGAAAACTTTAGCCACACCAACAAA
TTCTATATTGTGTCCTGATGGTCAGTCTCAATGTCCAGATGGAAGCACTTGTTGCAAACTGTCGACGGGT
GAATGGGGATGTTGTCCCTTACCCAAGGCTGTATGCTGCTCTGATGGTCTACATTGCTGTCCTAGTGGTA
CAACATGTGATGTTTCTCAGGGTAAATGTAATAAGGGTGACTTCAGTATGCCATGGGTCAAGAAAACTTT
AGCCACACCAACAAATTCTATATTGTGTCCTGATGGTCAGTCTCAATGTCCAGATGGGAGCACTTGTTGC
AAACTGTCAACCGGTCAATGGGGATGTTGTCCTTTACCCAAGGCTGTATGCTGCTCTGATGGTCTACATT
GCTGTCCTAGTGGTACAACATGTGATGATTCTCAGGGTAAATGTAATAAGGGTGACTTCAGTATGCCATG
GGTCAAGAAAACATTGGCATTAAGAAATGTTGATGTCATCTCTAGTGTTAATGATGTCATCTCTAGTGTT
AATGATGTCATCTCTAGTGTTAATGATGTCATCTCTAGTGTTAATGATGTCACATGTAAAGGAGGCAAGC
AGAAGTGCCCTGACGGGAATACGTGTTGTGAAGAAGCAAGTGGTTTGTTTGGTTGTTGTCCATTTCCAGA
TGCCGTTTGTTGTGTTGATCAACAACATTGTTGTCCACAAGGATTTACATGTGATTTACAACAAACAAAG
TGTATTAAAGGGTCTTTAGGATTGCCCCTAATGAAGAAAACCATGGCTCAGAATGTTGAAGTTGTTGTAT
GCCCTGGTGGTAAACAGGAATGCCCCTCACAGATGACTTGTTGTTCAGTACACAGAGATTTATATGGATG
TTGTCCGGTGCCTGATGCGGTTTGTTGTGATGACAAAATTCACTGCTGTCCCAAAGACTATACATGTAAG
GATAGCCGCTGTCTTAATGGAGATATTAACTTGCCGATGTTGGAACGTGTCTCGGCTAAATACACCCACA
AAGTAGAGTCAGTGATGTGCCCAGATGGTCAGTCTGAGTGTCCAGATGGGAGCACATGTTGTATATTAGC
CAGTGGTCAATATGGATGCTGTCCTTTACCAAAGGCTGTATGTTGTGATGATCACCAACACTGTTGTCCT
AATGGCTACAAATGTGACACGGCAGGAGGATCATGTCAGAAAGGAGATGACGCCATACCCTGGTTAAAGA
AACACAATTCTATTCCGATGTCTGGTAACGTAAAGTGCCAGGATGGTTCAGAGTGTCCTAGTGGTAGTAC
ATGCTGTAAACTGCCTACTGGGGACTATGGCTGCTGCCCATATCTTGATGCTGTCTGCTGCAACGATGGT
CTACATTGTTGTCCTAAAGGTTATACTTGTAATACCAAGGAAGGAACGTGTCAAAAGGCATCAATTAATA
TGTTGCCCTGGGTGAAGAGTTCAGAAGGTCTGGTTGATCTGGTTACCTGCCCTGACAAGGCAAAATGTCC
TGATAAAACGACATGTTGTCAGTTGCCATCTGACAAGTATGCCTGCTGTCCATTAGAACATGCTATTTGT
TGCGAGGATAAGATACACTGCTGTCCAGATTATTTGACATGTGACATGCCATCTGGAAAGTGTGTTCGCA
AATCCATCATATCTATTCCATGGATGGAGAAAATCCCTACACTTATAGAATTATGTGATGTGGATGTTTG
TCCAGATGGTCTGACAGGTTGTCTGAGTGGGCAGACTTGCACAGCTACAACCTCTGGACAATATAACTGC
TGTCCTATGCCAAATGCTGTCTCTTGTGATGGTGGTGAACACTGTTGTCCCCATGGTTACGCATGTGATA
AACACCAAGGATGTTTAGAAATGGATAATGTTCCCCTATCTTCAGTACAGCTACCATAGTAACATCTGTT
TTTGCAAAATTGTCTTTATCGAGGGAACTAAATTTGAGAAGGCATAATTGTATATTTTTTGTGATGATAA
TTTTTTTTACATTTTTGGCATTATTTTCTGTGCAAATTAGACTATTGTAGTTATTCAGAATGTTGCACTA
AATTTTTGAGCATATCTGGGACTTCTTATGTTGCAGTACAGCACCCTCAGGTGTTTAATTGTGTAATTAC
TTTGTTTCTGTTGTACCTTAAGGTGGTATTGTGAACATTAGACAAATCACTGCTAAAGATTAACCATTCC
CAAAATATTCACTTCCTGTTTTTAAAAAAAATGTAATAAAGTAATCCATCAATTAAAATTGTACAATCTT
CGATATCGAGATCCATGTTAAGAATGTTGGCATTCAGATATGATCACATGACATGTATATAATATCGAAA
AATTATGCATTCCAAATAAACCATGGAGGGAATACTTTTGTTGTATATTGAGGGCTCGCTACTACATACC
ATAGCATGTCCTGTTTTGATCAATAAGCTTCCCATGTGTATTGATATTTGGTAATGAATTTTCCATATAC
AAATAAAGGTGACTTGGACAATCTATCAAGAAAAGTAGCTTAATATTCTGTCTAGTGTTCCACTCTTTAT
GCATGCCACTAATACCTGGAATATGTGGTGTTCTACACTCCACTAATGCCTAAAATCTATGTGGTGATCT
ATAGTATACACACAACTAATGCCTAGAATCTGTGATGTTCCACCCTCAATATGCCAAAACACACAAGATT
CTGTGCCATGATTGGTCAATACGTAAACACGCAAGGTTGTATAGAATTACACGGTCACCCTTGCATAATG
AACTAATGGAAATAGTCAACAGATTATTCCTCATTGCATATCGAAAATTCCATGTTTTCTACCTTTAGCA
TGTCCATGTTTTGACCGATTTAGTATCTGGTGAGGATTTACAAATGTGATAAAAACTAATAAATATTTTG
TTTTAAATTTGTGGNCATTAAAAAAAAAAAAAAA

cDNA data:

From: NCBI est: Contig of GI:(187124962, 187100237, 187162181, 187051231, 187141477, 187167824, 187118922, 187202484, 187195250, 187094534, 187182439, 187220035, 187094072, 187092896, 187048843, 187071460, 187064262, 187162180, 187195249, 187066975, 187100236, 187096627, 187047420, 187167823)

Note: Provides a partial transcript missing the beginning. One Cap 3 assembly ambiguity showed an additional 2 codons in some transcripts. The translations affect the amino acid sequence PWMEKIPTLIDYV(NV)DVCP, where the difference is in parenthesis. This contig has the shorter VISSVND repeat. The transcript with a longer repeat is represented by a contig which includes EST sequences GI:187167982, 187051231.

```
CTTGTTGCAAACTGTCGACGGGTGAATGGGGATGTTGTCCCTTACCCAAGGCTGTATGCTGCTCTGATGG
TCTACATTGCTGTCCTAGTGGTACAACATGTGATGTTTCTCAGGGTAAATGTAATAAGGGTGACTTCAGT
ATGCCATGGGTCAAGAAAACTTTAGCCACACCAACAAATTCTATATTGTGTCCTGATGGTCAGTCTCAAT
GTCCAGATGGGAGCACTTGTTGCAAACTGTCAACCGGTCAATGGGGATGTTGTCCTTTACCCAAGGCTGT
ATGCTGCTCTGATGGTCTACATTGCTGTCCTAGTGGTACAACATGTGATGTTTCTCAGGGTAAATGTAAT
AAGGGTGACTTCAGTATGCCATGGGTCAAGAAAACATTGGCATTAAGAAATGTTGATGTCATCTCTAGTG
TTAATGATGTCATCTCTAGTGTTAATGATGTCATCTCTAGTGTTAATGATGTCACATGTAAAGGAGGCAA
GCAGAAGTGCCCTGACGGGAATACGTGTTGTGAAGAAGCAAGTGGTTTGTTTGGTTGTTGTCCATTTCCA
GATGCCGTTTGTTGTGTTGATCAACAACATTGTTGTCCACAAGGATTTACATGTGATTTACAACAAACAA
AGTGTATTAAAGGGTCTTTAGGATTGCCCCTAATGAAGAAAACCATGGCTCAGAATGTTGAAGTTGTTGT
ATGCCCTGGTGGTAAACAGGAATGCCCCTCACAGATGACTTGTTGTTCAGTACACGGAGATTTATATGGA
TGTTGTCCGGTGCCTGATGCGGTTTGTTGTGATGACAAAATTCACTGCTGTCCCAAAGACTATACGTGTA
AGGATAGCCGCTGTCTTAATGGAGATATTAACTTGCCGATGTTGGAACGTGTCTCGGCTAAATACACCCA
CAAAGTAGAGTCAGTGATGTGCCCAGATGGTCAGTCTGAGTGTCCAGATGGGAGCACATGTTGTATATTA
GCCAGTGGTCAATATGGATGCTGTCCTTTACCAAAGGCTGTATGTTGTGATGATCACCAACACTGTTGTC
CTAATGGCTACAAATGTGACACGGCAGGAGGATCATGTCAGAAAGGAGATGACGCCATACCCTGGTTAAA
GAAACACAATTCTATTCCGATGTCTGGTAACGTAAAGTGCCAGGATGGTTCAGAGTGTCCTAGTGGTAGT
ACATGCTGTAAACTGCCTACTGGGGACTATGGCTGCTGCCCATATCTTGATGCTGTCTGCTGCAACGATG
GTCTACATTGTTGTCCTAAAGGTTATACTTGTAATACCAAGGAAGGAACGTGTCAAAAGGCATCAATTAA
TATGTTGCCCTGGGTGAAGAGTTCAGAAGGTCTGGTTGATCTGGTTACCTGCCCTGACAAGGCAAAATGT
CCTGATAAAACGACATGTTGTCAGTTGCCATCTGACAAGTATGCCTGCTGTCCATTAGAACATGCTATTT
GTTGCGAGGATAAGATACACTGCTGTCCAGATTATTTGACATGTGACATGCCATCTGGAAAGTGTGTTCG
CAAATCCATCATATCTATTCCATGGATGGAGAAAATCCCTACACTTATAGAATTATGTGATGTGGATGTT
TGTCCAGATGGTCTGACAGGTTGTCTGAGTGGGCAGACTTGCACAGCTACAACCTCTGGACAATATAACT
GCTGTCCTATGCCAAATGCTGTCTCTTGTGATGGTGGTGAACACTGTTGTCCCCATGGTTACGCATGTGA
TAAACACCAAGGATGTTTAGAAATGGATAATGTTCCCCTATCTTCAGTACAGCTACCATAGTAACATCTG
TTTTTGCAAAATTGTCTTTATCGAGGGAACTAAATTTGAGAAGGCATAATTGTATATTTTTTGTGATGAT
AATTTTTTTTACATTTTTGGCATTATTTTCTGTGCAAATTAGACTATTGTAGTTATTCAGAATGTTGCAC
TAAATTTTTGAGCATATCTGGGACTTCTTATGTTGCAGTACAGCACCCTCAGGTGTTTAATTGTGTAATT
ACTTTGTTTCTGTTGTACCTTAAGGTGGTATTGTGAACATTAGACAAATCACTGCTAAAGATTAACCATT
CCCAAAATATTCACTTCCTGTTTTTAAAAAAAATGTAATAAAGTAATCCATCAATTAAAATTGTACAATC
TTCGATATCGAGATCCATGTTAAGAATGTTGGCATTCAGATATGATCACATGACATGTATATAATATCGA
AAAATTATGCATTCCAAATAAACCATGGAGGGAATACTTTTGTTGTATATTGAGGGCTCGCTACTACATA
CCATAGCATGTCCTGTTTTGATCAATAAGCTTCCCATGTGTATTGATATTTGGTAATGAATTTTCCATAT
ACAAATAAAGGTGACTTGGACAATCTATCAAGAAAAGTAGCTTAATATTCTGTCTAGTGTTCCACTCTTT
ATGCATGCCACTAATACCTGGAATATGTGGTGTTCTACACTCCACTAATGCCTAAAATCTATGTGGTGAT
CTATAGTATACACACAACTAATGCCTAGAATCTGTGATGTTCCACCCTCAATATGCCAAAACACACAAGA
TTCTGTGCCATGATTGGTCAATACGTAAACACGCAAGGTTGTATAGAATTACACGGTCACCCTTGCATAA
TGAACTAATGGAAATAGTCAACAGATTATTCCTCATTGCATATCGAAAATTCCATGTTTTCTACCTTTAG
CATGTCCATGTTTTGACCGATTTAGTATCTGGTGAGGATTTACAAATGTGATAAAAACTAATAAATATTT
TGTTTTAAATTTGTGGNCATTAAAAAAAAAAAAAAA
```

Genomic data:

Note: Gaps in the S. kowalevskii progranulin gene sequence data from the main NCBI WGS database were partially filled by data from the Trace archive. Although the full genomic sequence was not assembled, we obtained sequence which includes all known exons.

From NCBI wgs: GI:281441650:128282-148110 unplaced genomic scaffold

Note: These first 6 exons look good. Downstream are large gaps, and an incomplete granulin exon. The presence of at least one exon in the gaps after the sixth coding exon is shown by cDNA data, which also overlaps the partial exon sequence at the end of this entry.

Coding exon 1 **Exon type: sn**

ATGATTTTGTTATTCATTACATTCATGGTGCTGTCGCATGCTATTGCTTCTCCTTTGTATTTTACCCCGA
GTGAATTAAACTCTATGTTATGTCCGGATGGAAAGTCTCAATGTCCAGATGGAAGTACATGTTGTAAATT
GACCAGTGGAGAATGGGGATGCTGTCCAATGCTAAAG

Coding exon 2 **Exon type: cn**
Note: Coding exons 2, 3,4, 5,6 are nearly identical repeats.
GCAGTATGCTGCTCTGATGGTCTACATTGCTGTCCTAATGGTACAACATGTGATGTTTCTCGGGGTAAAT
GTAATAAGGGTGAATTTAGTATGCAATGGGTCAAGAAAACACGATCAATCCCCGCTGACTCTATCTTGTG
TCCTGATGGTAAATCCCAATGTCCAGATGGCAACACGTGCTGTAAGCTGAAAACTGGAGAGTATGGGTGT
TGTCCTTTACCCAAG

Coding exon 3 **Exon type: cn**
Note: Coding exons 2, 3,4, 5,6 are nearly identical repeats.
GCTGTATGCTGCTCTGATGGTCTACATTGCTGTCCTAATGGTACAACATGTGATGTTTCTCAGGGTAAAT
GTAATAAGGGTGACTTCAGTATGCCGTGGGTCAAGAAAACTTTAGCCACACCAACAAATTCTATATTGTG
TCCTGATGGTCAGTCTCAATGTCCAGATGGAAGCACTTGTTGCAAACTGTCGACGGGTGAATGGGGATGT
TGTCCCTTACCCAAG

Coding exon 4 **Exon type: cn**
Note: Coding exons 2, 3,4, 5,6 are nearly identical repeats.
GCTGTATGCTGCTCTGATGGTCTACATTGCTGTCCTAGTGGTACAACATGTGATGTTTCTCAGGGTAAAT
GTAATAAGGGTGACTTCAGTATGCCATGGGTCAAGAAAACTTTAGCCACACCAACAAATTCTATATTGTG
TCCTGATGGTCAGTCTCAATGTCCAGATGGAAGCACTTGTTGCAAACTGTCGACTGGTCAATGGGGATGT
TGTCCATTACCCAAG

Coding exon 5 **Exon type: cn**
Note: Coding exons 2, 3,4, 5,6 are nearly identical repeats.
GCTGCATGCTGCTCTGATGGTCTACATTGCTGTCCTAATGGTACAACATGTGATGTTTCTCAGGGTAAAT
GTAATAAGGGTGACTTCAGTATGCCATGGGTCAAGAAAACTTTAGCCACACCAACAAATTCTATATTGTG
TCCTGATGGTCAGTCTCAATGTCCAGATGGAAGCACTTGTTGCAAACTGTCGACGGGTGAATGGGGATGT
TGTCCCTTACCCAAG

Coding exon 6 **Exon type: cn**
Note: Coding exons 2, 3,4, 5,6 are nearly identical repeats.
GCTGTATGCTGCTCTGATGGTCTACATTGCTGTCCTAGTGGTACAACATGTGATGTTTCTCAGGGTAAAT
GTAATAAGGGTGACTTCAGTATGCCATGGGTCAAGAAAACTTTAGCCACACCAACAAATTCTATATTGTG
TCCTGATGGTCAGTCTCAATGTCCAGATGGGAGCACTTGTTGCAAACTGTCAACCGGTCAATGGGGATGT
TGTCCTTTACCCAAG

From NCBI Trace archive of Saccoglossus kowalevskii wgs sequences.: Contig of TI:1752672055, 1713392136.
Note: This contig overlaps GI:281441650:128282-148110, fills some of the last gap, and completely defines exon 7. Two similar contigs were assembled. This one contains the coding exon 7 with the longer TGACATCATTAACACTAGAGA repeat encoding VISSVND four times. The allelic exon with just three repeats is represented by a contig of TI:1746992008, 1702121647.
Coding exon 7 **Exon type: cnc**
Note: The longer coding exon 7 (see comment above).
GCTGTATGCTGCTCTGATGGTCTACATTGCTGTCCTAGTGGTACAACATGTGATGTTTCTCAGGGTAAAT
GTAATAAGGGTGACTTCAGTATGCCATGGGTCAAGAAAACATTGGCATTAAGAAATGTTGATGTCATCTC
TAGTGTTAATGATGTCATCTCTAGTGTTAATGATGTCATCTCTAGTGTTAATGATGTCATCTCTAGTGTT
AATGATGTCACATGTAAAGGAGGCAAGCAGAAGTGCCCTGACGGGAATACGTGTTGTGAAGAAGCAAGTG
GTTTGTTTGGTTGTTGTCCATTTCCAGATGCCGTTTGTTGTGTTGATCAACAACATTGTTGTCCACAAGG
ATTTACATGTGATTTACAACAAACAAAGTGTATTAAAGGGTCTTTAGGATTGCCCCTAATGAAGAAAACC
ATGGCTCAG

From NCBI WGS: GI:255177688 (GB:ACQM01074953.1) Saccoglossus kowalevskii Contig75118, whole genome shotgun sequence.
Note: This contig contains 4 exons.
Coding exon 8 **Exon type: n**
AATGTTGAAGTTGTTGTATGCCCTGGTGGTAAACAGGAATGCCCCTCACAGATGACTTGTTGTTCAGTAC
ACAGAGATTTATATGGATGTTGTCCGGTGCCTGAT

Coding exon 9 **Exon type: c**
GCGGTTTGTTGTGATGACAAAATTCACTGCTGTCCCAAAGACTATACATGTAAGGATAGCCGCTGTCTTA
ATGGAGATATTAACTTGCCGATGTTGGAACGTGTCTCGGCTAAATACACCCACAAA

Coding exon 10 **Exon type: n**
GTAGAGTCAGTGATGTGCCCAGATGGTCAGTCTGAGTGTCCAGATGGGAGCACATGTTGTATATTAGCCA
GTGGTCAATATGGATGCTGTCCTTTACCAAAG

Coding exon 11 **Exon type: cncn**
GCTGTATGTTGTGATGATCACCAACACTGTTGTCCTAATGGCTACAAATGTGACACGGCAGGAGGATCAT
GTCAGAAAGGAGATGACGCCATACCCTGGTTAAAGAAACACAATTCTATTCCGATGTCTGGTAACGTAAA
GTGCCAGGATGGTTCAGAGTGTCCTAGTGGTAGTACATGCTGTAAACTGCCTACTGGGGACTATGGCTGC
TGCCCATATCTTGATGCTGTCTGCTGCAACGATGGTCTACATTGTTGTCCTAAAGGTTATACTTGTAATA
CCAAGGAAGGAACGTGTCAAAAGGCATCAATTAATATGTTGCCCTGGGTGAAGAGTTCAGAAGGTCTGGT
TGATCTGGTTACCTGCCCTGACAAGGCAAAATGTCCTGATAAAACGACATGTTGTCAGTTGCCATCTGAC
AAGTATGCCTGCTGTCCATTAGAACAT

From NCBI Trace archive of Saccoglossus kowalevskii wgs sequences.: Contig of TI:1701193238, 1659660105, 1721786828, 1661741634, 1747024700.
Note: This contig overlaps GI:255177688 and also has coding exon 11.
Coding exon 12 **Exon type: cn***
Note: Where n* represents a 5 Cys N-half module.
GCTATTTGTTGCGAGGATAAGATACACTGCTGTCCAGATTATTTGACATGTGACATGCCATCTGGAAAGT
GTGTTCGCAAATCCATCATATCTATTCCATGGATGGAGAAAATCCCTACACTTATAGATTATGTGGATGT
TTGTCCAGATGGTCTGACAGGTTGTCTGAGTGGGCAGACTTGCACAGCTACAACCTCTGGACAATATAAC
TGCTGTCCTATGCCAAAT

From NCBI WGS: GI:255177690 (GB:ACQM01074951.1) Saccoglossus kowalevskii Contig75116, whole genome shotgun sequence.
Note: This has part of exon 12, so it overlaps with the previous contig of trace archive sequences.
Coding exon 13 **Exon type: c***
Note: Ending at the stop codon. Here c* indicates a 5 Cys C-half module. The exon also contains all 3'ut which is not shown here.
GCTGTCTCTTGTGATGGTGGTGAACACTGTTGTCCCCATGGTTACGCATGTGATAAACACCAAGGATGTT
TAGAAATGGATAATGTTCCCCTATCTTCAGTACAGCTACCATAG

Genomic sequence note: A genomic sequence is not presented (see note 2 in header).

00037 **S. purpuratus progranulin** Short name: **S_pur** Date entered: 2014-03

Species: ***Strongylocentrotus purpuratus (purple sea urchin)***
Taxonomy (via NCBI): *Eukaryota; Metazoa; Echinodermata; Eleutherozoa; Echinozoa; Echinoidea; Euechinoidea; Echinacea; Echinoida; Strongylocentrotidae; Strongylocentrotus.*
General Comment: The first exon encoding the signal peptide is followed by a large exon encoding 25 modules containing a repeat of 23 nearly identical modules. The first encoded module is different, as is the C-half of the last module. The gene ends with 4 more normal exons n-cn-n-c, so the product would include a paragranulin. Limited EST data confirm splicing from the repeat through the last 4 exons. Three other EST sequences cover short parts of the repeat interior. NOTE: The gene reported as NW_003580203.1 GI:346417161 is wrong. The DNA sequence starts within the repeat of exon 2, while translation is recorded from an internal Met codon in exon 3.

Protein Sequence

MKLVTILVVASLVSLAFGGPLKVEELAKENTCKDGCATTSTCCNDLNNESGCCTYPNAVCCSDGRHCCPT
GYECDVTSNTCIKGNDVMRMLITDLTSNASAVICPDGQSECPDGNTCCKLASGQYGCCPLPNAVCCSDHL
HCCPSGYTCGVSSGTCTQGESVVPWYEKSSATPVHVENVVCPDGQSECPDGNTCCTLASGQYGCCPLPNA
VCCSDHLHCCPSGYTCGVSSGTCTQGESVVPWYEKSSATPVHVESVVCPDGQSECPDGNTCCTLASGQYG
CCPLPNAVCCSDHLHCCPSGYTCGVSSGTCTRGESVVLWYEKSSATPVHVESVVCPDGQSECPDGNTCCK
LASGQYGCCPLPNAVCCSDHLHCCPSGYTCGVSSGTCTQGESVVPWYEKSSATPVHVESVVCPDGQSECP
DGNTCCTLASGQYGCCPLPNAVCCSDHLHCCPSGYTCGVSSGTCTQGESVVPWYEKSSATPVHEENVVCP
DGQSECPDGNTCCKLASGQYGCCPLPNAVCCSDHLHCCPSGYTCGVSSGTCTQGESVVPWYEKSSATPVH
EENVVCPDGQSECPDGNTCCKLASGQYGCCPLPNAVCCSDHLHCCPSGYTCGVSSGTCTQGESVVPWYEK

SSATPVLVENVVCPDGQSECPDGNTCCTLASGQYGCCPLPNAVCCSDHLHCCPSGYTCGVSSGTCTRGES
VVLWYEKSSATPVQVENVVCPDGQSECPDGNTCCKLASGQYGCCPLPNAVCCSDHLHCCPSGYTCGVSSG
TCTQGESVVPWYEKSSATPVHVENVVCPDGQSECPDGNTCCTLASGQYGCCPLPNAVCCSDHLHCCPSGY
TCGVSSGTCTQGESVVPWYEKSSATPVHVENVVCPDGQSECPDGNTCCKLASGQYGCCPLPNAVCCSDHL
HCCPSGYTCGVSSGTCTQGESVVSWYEKSAATPVQVKSVICPGGQAQCPDGNTCCKLASGQYGCCPLPNA
VCCSDHLHCCPSGYTCGVSAGTCTRGESVVLWYEKSSATPVQVENVVCPDGQSECPDGNTCCTLASGQYG
CCPLPNAVCCSDHLHCCPSGYTCGVSSGTCTQGESVVLWFEKSAATPVQVESVICPGGQSECPDGNTCCK
LASGQYGCCPLPNAVCCSDHLHCCPSGYTCGVSSGTCTQGESVVPWYEKSSATPVHVENVVCPDGQSECP
DGNTCCKLASGQYGCCPLPNAVCCSDHLHCCPSGYTCGVSSGTCTQGESVVSWYEKSAATPVQVESVICP
GGQAQCPDGNTCCKLASGQYGCCPLPNAVCCSDHLHCCPSGYTCGVSAGTCTRGESVVLWYEKSSATPVQ
VENVVCPDGQSECPDGNTCCKLASGQYGCCPLPNAVCCSDHLHCCPSGYTCGVSSGTCTQGESVVLWYEK
SAATPVQVESVLCPGGQSECPDGNTCCKLASGQYGCCPLPNAVCCSDHLHCCPSGYTCGVSSGTCTQGES
VVPWYEKSSATPVHVENVVCPDGQSECPDGNTCCKLASGQYGCCPLPNAVCCSDHLHCCPSGYTCGVSSG
TCTQGESVVPWYEKSSATPVQVDSVICPGGQAQCPDGNTCCKLPSGQYGCCPLPNAVCCSDGEHCCPSGY
TCDVSAGTCTQGESVVLWYEKSSATPVQVESVVCPGGQAQCPDGNTCCKLPSGQYGCCPLPNAVCCSDGE
HCCPSGYTCDVSAGTCTQGESVVPWYEKSSATPVQIESVVCPDGQSECPDGSTCCKLASGQYGCCPIPSA
VCCSDHLHCCPSGYTCDVSAGTCTQGESVVPWYEKSSATPVQIESVVCPDGQSECPDGSTCCKLASGQYG
CCPLPNAVCCSDGQHCCPSGYTCDVSAGTCTQGESVVPWYEKSSATPVYVESVVCPDGQSECPDGNTCCK
LASGQYGCCPLPNAVCCSDHLHCCPSGYTCGVSSGTCTRGSSILEWSSKSSARSNVDKLQITATEPVSIC
PDSKTSCPSSSTCCPLMASGEWGCCPVHNAVCCDDHIHCCPSGYSCGTNSCQKGSMHLPLLSKSTGLLKL
TFEGQEEPKPEVKVHDNKHLLFQPDEPRTGSVICPGGKIECQDGNACCLLAGGDYSCCPLPQAVSPHEES
GDKMMCPDEISSCPTGSTCCQTPGNIYGCCPYQAAICCSDLIHCCPSDYSCNSSTGTCTKDDHIMPWVGK
IPPL*

Transcript:

Note: Limited cDNA data from EST sequences confirm the exons from the end of the large repeat to the 3' end. The beginning and much of the repeat exon are unconfirmed.

Predicted sequence:

Note: Predicted from AAGJ04101346.1 (2855-13696, stop-start) reverse complement.

Derivation: AAGJ04101346.1 GI:335681100 range:2855-13696

ATGAAGCTGGTGACTATCCTTGTGGTGGCTTCTCTTGTGTCACTGGCATTTGGAGGACCCCTGAAGGTGG
AAGAACTTGCCAAAGAGAATACTTGTAAAGATGGATGTGCTACTACATCAACGTGTTGTAACGACTTGAA
CAATGAATCAGGATGTTGCACATACCCAAATGCAGTATGCTGCTCTGATGGTCGCCACTGTTGTCCTACA
GGCTACGAATGTGATGTCACCAGCAACACATGCATAAAAGGAAATGATGTAATGAGGATGTTGATCACAG
ATTTAACTAGTAATGCATCTGCTGTAATTTGTCCTGATGGCCAATCAGAATGTCCCGATGGTAATACATG
TTGCAAACTTGCTTCTGGCCAATATGGTTGTTGCCCTCTACCAAATGCAGTTTGTTGTAGTGATCATCTT
CACTGCTGTCCATCAGGATACACTTGTGGTGTATCATCTGGCACATGCACTCAAGGAGAGTCTGTTGTCC
CTTGGTATGAAAAGTCATCTGCCACTCCTGTGCACGTAGAAAATGTTGTATGTCCTGATGGCCAATCAGA
ATGTCCAGATGGTAATACATGTTGCACACTTGCTTCTGGCCAATATGGTTGTTGTCCTCTGCCAAATGCA
GTGTGTTGTAGTGATCATCTTCACTGCTGTCCATCAGGTTACACTTGTGGTGTTTCATCTGGCACATGCA
CACAGGGAGAGTCTGTTGTCCCTTGGTATGAAAAGTCATCTGCCACTCCTGTGCACGTAGAAAGTGTTGT
ATGCCCAGATGGCCAATCAGAATGTCCAGATGGTAATACATGTTGCACACTTGCTTCTGGCCAATATGGT
TGTTGTCCTCTGCCAAATGCAGTGTGTTGTAGTGATCATCTTCACTGCTGTCCATCAGGTTACACTTGTG
GTGTATCATCTGGCACATGCACACGAGGAGAGTCTGTTGTCTTATGGTATGAAAAGTCATCTGCCACTCC
TGTGCACGTAGAAAGTGTTGTATGTCCTGATGGCCAATCAGAATGTCCAGATGGTAATACATGTTGCAAA
CTTGCTTCTGGCCAATATGGTTGTTGTCCTCTACCAAATGCAGTGTGTTGTAGTGATCATCTTCACTGCT
GTCCATCAGGATACACTTGTGGTGTATCATCTGGCACATGCACTCAAGGAGAGTCTGTTGTCCCTTGGTA
TGAAAAGTCATCTGCCACTCCTGTGCACGTAGAAAGTGTTGTATGTCCTGATGGCCAATCAGAATGTCCA
GATGGTAATACATGTTGCACACTTGCTTCTGGCCAATATGGTTGTTGTCCTCTACCAAATGCAGTGTGTT
GTAGTGATCATCTTCACTGCTGTCCATCAGGATACACTTGTGGTGTATCATCTGGCACATGCACTCAAGG
AGAGTCTGTTGTCCCTTGGTATGAAAAGTCATCTGCCACTCCTGTGCACGAAGAAAATGTTGTATGTCCT
GATGGCCAATCAGAATGTCCAGATGGTAATACATGTTGCAAACTTGCTTCTGGCCAATATGGTTGTTGTC
CTCTACCAAATGCAGTTTGTTGTAGTGATCATCTTCACTGCTGTCCATCAGGATACACTTGTGGTGTATC
ATCTGGCACATGCACTCAAGGAGAGTCTGTTGTCCCTTGGTATGAAAAGTCATCTGCCACTCCTGTGCAC
GAAGAAAATGTTGTATGTCCTGATGGCCAATCAGAATGTCCAGATGGTAATACATGTTGCAAACTTGCTT
CTGGCCAATATGGTTGTTGTCCTCTACCAAATGCAGTGTGTTGTAGTGATCATCTTCACTGCTGTCCATC
AGGTTACACTTGTGGTGTTTCATCTGGCACATGCACACAGGGAGAGTCTGTTGTCCCTTGGTATGAAAAG
TCATCTGCCACTCCTGTGCTTGTAGAAAATGTTGTATGTCCTGATGGCCAATCAGAATGTCCAGATGGTA

ATACATGTTGCACACTTGCTTCTGGCCAATATGGTTGTTGTCCTCTGCCAAATGCAGTGTGTTGTAGTGA
TCATCTTCACTGCTGTCCATCAGGATACACTTGTGGTGTATCATCTGGCACATGCACACGAGGAGAGTCT
GTTGTCTTATGGTATGAAAAATCATCTGCCACTCCAGTGCAAGTAGAAAATGTTGTATGTCCTGATGGCC
AATCAGAATGTCCAGATGGTAATACATGTTGCAAACTTGCTTCTGGCCAATATGGTTGTTGTCCTCTACC
AAATGCAGTGTGTTGTAGTGATCATCTTCACTGCTGTCCATCAGGATACACTTGTGGTGTATCATCTGGC
ACATGCACTCAAGGAGAGTCTGTTGTCCCTTGGTATGAAAAGTCATCTGCCACTCCTGTGCACGTAGAAA
ATGTTGTATGCCCTGATGGCCAATCAGAATGTCCAGATGGTAATACATGTTGCACACTTGCTTCTGGCCA
ATATGGTTGTTGTCCTCTACCAAATGCAGTTTGTTGTAGTGATCATCTTCACTGCTGTCCATCAGGATAC
ACTTGTGGTGTATCATCTGGCACATGCACTCAAGGAGAGTCTGTTGTCCCTTGGTATGAAAAGTCATCTG
CCACTCCTGTGCACGTAGAAAATGTTGTATGTCCTGATGGCCAATCAGAATGTCCAGATGGTAATACATG
TTGCAAACTTGCTTCTGGCCAATATGGTTGTTGTCCTCTACCAAATGCAGTTTGTTGTAGTGATCATCTT
CACTGCTGTCCATCAGGATACACTTGTGGTGTATCATCTGGCACATGCACTCAAGGAGAGTCTGTTGTCT
CTTGGTATGAAAAGTCAGCTGCCACTCCTGTGCAAGTAAAGAGTGTTATCTGTCCAGGAGGCCAAGCACA
ATGCCCAGATGGTAATACATGTTGCAAACTTGCTTCTGGCCAATATGGTTGTTGTCCTCTGCCAAATGCA
GTGTGTTGTAGTGATCATCTTCACTGCTGTCCATCAGGATACACTTGCGGTGTTTCTGCAGGAACCTGCA
CACGAGGAGAGTCTGTTGTCTTATGGTATGAAAAATCATCTGCCACTCCAGTGCAAGTAGAAAATGTTGT
ATGTCCTGATGGCCAATCAGAATGTCCAGATGGTAATACATGTTGCACACTTGCTTCTGGCCAATATGGT
TGTTGTCCTCTACCAAATGCAGTTTGTTGTAGTGATCATCTTCACTGCTGTCCATCAGGATACACTTGTG
GTGTATCATCTGGTACATGCACACAGGGAGAGTCTGTGGTCTTATGGTTTGAAAAATCAGCTGCCACTCC
TGTGCAAGTAGAGAGTGTTATCTGTCCAGGAGGCCAATCCGAATGTCCAGATGGTAATACATGTTGCAAA
CTTGCTTCTGGTCAGTATGGTTGTTGCCCTCTACCAAATGCAGTTTGTTGTAGTGATCATCTTCACTGCT
GTCCATCAGGATACACTTGTGGTGTATCATCTGGCACATGCACTCAAGGAGAGTCTGTTGTCCCTTGGTA
TGAAAAGTCATCTGCCACTCCTGTGCACGTAGAAAATGTTGTTTGTCCTGATGGCCAATCAGAATGTCCA
GATGGTAATACATGTTGCAAACTTGCTTCTGGCCAATATGGTTGTTGTCCTCTACCAAATGCAGTTTGTT
GTAGTGATCATCTTCACTGCTGTCCATCAGGATACACTTGTGGTGTATCATCTGGCACATGCACTCAAGG
AGAGTCTGTTGTCTCTTGGTATGAAAAGTCAGCTGCCACTCCTGTGCAAGTAGAGAGTGTTATCTGTCCA
GGAGGCCAAGCACAATGCCCAGATGGTAATACATGTTGCAAACTTGCTTCTGGCCAATATGGTTGTTGTC
CTCTGCCAAATGCAGTGTGTTGTAGTGATCATCTTCACTGCTGTCCATCAGGATACACTTGTGGTGTTTC
TGCAGGAACCTGCACACGAGGAGAGTCTGTTGTCTTATGGTATGAAAAATCATCTGCCACTCCAGTGCAA
GTAGAAAATGTTGTATGTCCTGATGGCCAATCAGAATGTCCAGATGGTAATACATGTTGCAAGCTTGCTT
CTGGCCAATATGGTTGTTGTCCTCTACCAAATGCAGTTTGTTGTAGTGATCATCTTCACTGCTGTCCATC
AGGATACACTTGTGGTGTATCATCTGGTACATGCACACAGGGAGAGTCTGTGGTCTTATGGTATGAAAAG
TCAGCTGCCACTCCTGTGCAAGTAGAGAGTGTTCTCTGTCCAGGAGGCCAATCCGAATGTCCAGATGGTA
ATACATGTTGCAAACTTGCTTCTGGCCAGTATGGTTGTTGCCCTCTACCAAATGCAGTTTGTTGTAGTGA
TCATCTACACTGCTGTCCATCAGGATACACTTGTGGTGTATCATCTGGCACATGCACTCAAGGAGAGTCT
GTTGTCCCTTGGTATGAAAAGTCATCTGCCACTCCTGTGCACGTGGAAAATGTTGTATGTCCTGATGGCC
AATCAGAATGTCCAGATGGTAATACATGTTGCAAACTTGCTTCTGGCCAATATGGTTGTTGTCCTCTACC
AAATGCAGTGTGTTGTAGTGATCATCTTCACTGCTGTCCATCAGGATACACTTGTGGTGTATCATCTGGC
ACATGCACTCAAGGAGAGTCTGTTGTCCCTTGGTATGAAAAGTCATCTGCCACTCCTGTGCAAGTAGACA
GTGTTATCTGTCCAGGAGGCCAAGCACAATGCCCAGATGGTAATACATGTTGCAAACTTCCTTCTGGTCA
ATATGGTTGTTGTCCTTTACCAAATGCAGTGTGTTGTAGTGATGGTGAGCACTGCTGTCCGTCAGGATAC
ACTTGCGATGTTTCTGCGGGAACCTGCACACAGGGAGAGTCTGTGGTCTTATGGTATGAAAAGTCATCTG
CCACTCCAGTGCAAGTAGAGAGTGTTGTATGTCCAGGAGGCCAAGCACAATGCCCAGATGGTAATACATG
TTGCAAACTTCCTTCTGGTCAATATGGTTGTTGTCCTTTACCAAATGCAGTGTGTTGTAGTGATGGTGAG
CACTGCTGTCCGTCAGGATACACTTGCGATGTTTCTGCAGGAACCTGCACACAAGGAGAGTCTGTTGTTC
CTTGGTATGAAAAGTCATCTGCCACTCCTGTGCAGATAGAGAGTGTTGTATGCCCTGATGGCCAATCAGA
ATGCCCAGATGGTAGTACATGTTGCAAACTTGCTTCTGGCCAATATGGTTGTTGTCCTATACCAAGTGCA
GTGTGTTGTAGTGATCATCTTCACTGCTGTCCATCAGGATACACTTGCGATGTTTCTGCAGGAACCTGCA
CACAAGGAGAGTCTGTTGTTCCTTGGTATGAAAAGTCATCTGCCACTCCTGTGCAGATAGAGAGTGTTGT
ATGCCCTGATGGCCAATCAGAATGCCCAGATGGTAGTACATGTTGCAAACTTGCTTCTGGTCAATATGGT
TGTTGTCCTTTACCAAATGCAGTGTGTTGTAGTGATGGTCAGCACTGCTGTCCATCAGGATACACTTGCG
ATGTTTCTGCAGGAACCTGCACACAAGGAGAGTCTGTTGTCCCTTGGTATGAAAAGTCATCTGCCACTCC
AGTGTACGTAGAGAGTGTTGTATGTCCTGATGGCCAATCAGAATGCCCAGATGGTAATACATGTTGCAAG
CTTGCTTCTGGCCAATATGGTTGTTGTCCTCTACCAAATGCAGTTTGTTGTAGTGATCATCTTCACTGTT
GTCCATCAGGATACACTTGTGGTGTATCATCTGGCACATGTACAAGGGGTAGCAGCATCTTGGAATGGAG
TTCAAAGTCAAGTGCACGTTCTAATGTAGACAAACTTCAGATTACGGCCACTGAACCAGTTAGCATCTGC
CCAGATAGCAAGACATCATGCCCTTCATCAAGCACTTGTTGCCCCCTCATGGCTTCAGGAGAATGGGGTT
GCTGCCCCGTACATAATGCTGTGTGCTGTGATGACCACATTCACTGTTGCCCATCAGGCTACAGTTGCGG
AACCAACTCCTGCCAGAAGGGCTCCATGCACCTACCCCTTCTCTCCAAGTCCACTGGCCTCCTTAAGCTT
ACCTTCGAGGGCCAGGAAGAACCAAAGCCAGAGGTCAAAGTTCATGATAATAAGCATCTCCTATTTCAGC

CAGATGAACCACGGACAGGATCCGTCATCTGCCCAGGAGGCAAAATAGAATGTCAAGATGGAAACGCTTG
TTGCCTGTTAGCCGGCGGTGATTATTCTTGTTGCCCTCTCCCTCAGGCGGTGTCCCCCCATGAGGAATCA
GGTGATAAGATGATGTGTCCTGATGAGATCTCATCCTGTCCCACTGGATCCACCTGCTGTCAGACACCAG
GAAACATCTACGGCTGCTGCCCCTACCAAGCAGCTATCTGCTGCTCTGATCTGATACACTGCTGTCCAAG
TGACTACTCCTGTAACAGCTCTACAGGAACATGCACCAAAGATGACCATATCATGCCATGGGTGGGAAAG
ATTCCACCTCTCTAA

cDNA data:

From: NCBI est: CD320922.1 GI:34792983
Note: This cDNA fragment encodes the last 2 modules from exon2 and confirms the splice into exon 3. It overlaps by 6 bases with an EST contig defining the rest of exon 3 to the last coding and 3'ut exon 6.
ACCACGCGTCCGGTTTCTGCAGAACCTGCACACAAGGAGAGTCTGTTGTTCCTTGGTATGAAAAGTCATC
TGCCACTCCTGTGCAGATAGAGAGTGTTGTATGCCCTGATGGCCAATCAGAATGCCCAGATGGTAGTACA
TGTTGCAAACTTGCTTCTGGCCAATATGGTTGTTGTCCTATACCAAGTGCAGTGTGTTGTAGTGATCATC
TTCACTGCTGTCCATCAGGATACACTTGCGATGTTTCTGCAGGAACCTGCACACAAGGAGAGTCTGTTGT
CCCTTGGTATGAAAAGTCATCTGCCACTCCAGTGTACGTAGAGAGTGTTGTATGTCCTGATGGCCAATCA
GAATGCCCAGATGGTAATACATGTTGCAAACTTGCTTCTGGCCAATATGGTTGTTGTCCTCTACCAAATG
CAGTTTGTTGTAGTGATCATCTTCACTGTTGTCCATCAGGATACACTTGTGGTGTATCATCTGGCACATG
TACAAGGGGTAGCAGCATCTTGGAATGGAGTTCANAGTCAAGTGCACGTTCTAATGTAGANCAACTTCAG
ATTACGGCCACTGAACCAGTTAGCATCTGCCCAGATAGCAAGACATCATGCCCTTCATCAAGCACTTT

From: NCBI est: Contig of GI:(57581490, 109403311, 34806747, 56845941, 56592915, 61235384)
Note: This cDNA EST contig overlaps by 6 bases with CD320922.1 GI:34792983 and defines the rest of exon 3 to the last coding and 3'ut exon 6.
CACTTTTTGCCCCCTCATGGCTTCAGGAGAATGGGGTTGCTGCCCCGTACATAATGCTGTGTGCTGTGAT
GACCACATTCACTGTTGCCCATCAGGCTACAGTTGCGGAACCAACTCCTGCCAGAAGGGCTCCATGCACC
TACCCCTTCTCTCCAAGTCCACTGGCCTCCTTAAGCTTACCTTCGAGGGCCAGGAAGAACCAAAGCCAGA
GGTCAAAGTTCATGATAATAAGCATCTCCTATTTCAGCCAGATGAACCACGGACAGGATCCGTCATCTGC
CCAGGAGGCAAAATAGAATGTCAAGATGGAAACGCTTGTTGCCTGTTAGCCGGCGGTGATTATTCTTGTT
GCCCTCTCCCTCAGGCGGTGTCCCCCCATGAGGAATCAGGTGATAAGATGATGTGTCCTGATGAGATCTC
ATCCTGTCCCACTGGATCCACCTGCTGTCAGACACCAGGAAACATCTACGGCTGCTGCCCCTACCAAGCA
GCTATCTGCTGCTCTGATCTGATACACTGCTGTCCAAGTGACTACTCCTGTAACAGCTCTACAGGAACAT
GCACCAAAGATGACCATATCATGCCATGGGTGGGAAAGATTCCACCTCTCTAATCCATTGAGCTTCAAGG
ATGACATGGCTAAGGAGACTTTACACAAGATCAACAAATTTCTAGACAAAAATATGATTTTGCTTTTAAA
GTACCTATCAGTTGGAGAGACTTATTTTTAGTACTATGAGTATGCTTCTTAAAGCAATTTATCTGTAAGA
TAAGCTTGATTGATGTATGGTTATATTTATGTCAGTTTTAAAATATAAATTAAAAATTTATTAACTTACA
AAATTTGAAATTTGGGTTGTGTTACTACATAAAGAGTGTTTTGAGATTAAGAAGTATTCCATGGGAAAGA
TTCCACACCACTTCCAGCTTGAGTAGTTGTGGGTAAGAATTTCTTATTTTTACCTCACTGAAAAAACATT
ATAACATGTATTATTATGTAAAAATGGTGAAGTAGTGGTGACAATCCTCGATTTGATGAATTTATTTTAT
GCAAATTCAGATTGTATGTAATTCTAGTACCATATCAGTTTTGATTTATACATTTTTTCATATATAGATT
AGAGCAGTCACTGTACATAAATTGAAGGAAGGAGGAAAATATTAATCAGGCTGTCTTGAATCTAATCCAG
TTATNGTTACTAGAGATATATTTTGAGAATATTGTTGATGGACTTTAATCACTATGTATGTCATCCTGAC
TGTATG

Genomic data:

Note: Genomic data for our prediction was from NW_001286694.1 GI:115655586, since replaced by gi: 335681100 gb: AAGJ04101346.1 (gene in reverse orientation).
From NCBI wgs: AAGJ04101346.1 GI:335681100 (2190..13760)
Note: The range given is sufficient to include the beginning of coding exon 1 and the 3'ut confirmed by EST cDNA data.
Coding exon 1 **Exon type: s**
Note: From the start codon.
ATGAAGCTGGTGACTATCCTTGTGGTGGCTTCTCTTGTGTCACTGGCATTTGGAGGACCCCTGAAG

Coding exon 2 **Exon type: ncncncncncncncncncncncncncncncncncncncncncncncncncnc**
Note: repetitive
GTGGAAGAACTTGCCAAAGAGAATACTTGTAAAGATGGATGTGCTACTACATCAACGTGTTGTAACGACT
TGAACAATGAATCAGGATGTTGCACATACCCAAATGCAGTATGCTGCTCTGATGGTCGCCACTGTTGTCC
TACAGGCTACGAATGTGATGTCACCAGCAACACATGCATAAAAGGAAATGATGTAATGAGGATGTTGATC

ACAGATTTAACTAGTAATGCATCTGCTGTAATTTGTCCTGATGGCCAATCAGAATGTCCCGATGGTAATA
CATGTTGCAAACTTGCTTCTGGCCAATATGGTTGTTGCCCTCTACCAAATGCAGTTTGTTGTAGTGATCA
TCTTCACTGCTGTCCATCAGGATACACTTGTGGTGTATCATCTGGCACATGCACTCAAGGAGAGTCTGTT
GTCCCTTGGTATGAAAAGTCATCTGCCACTCCTGTGCACGTAGAAAATGTTGTATGTCCTGATGGCCAAT
CAGAATGTCCAGATGGTAATACATGTTGCACACTTGCTTCTGGCCAATATGGTTGTTGTCCTCTGCCAAA
TGCAGTGTGTTGTAGTGATCATCTTCACTGCTGTCCATCAGGTTACACTTGTGGTGTTTCATCTGGCACA
TGCACACAGGGAGAGTCTGTTGTCCCTTGGTATGAAAAGTCATCTGCCACTCCTGTGCACGTAGAAAGTG
TTGTATGCCCAGATGGCCAATCAGAATGTCCAGATGGTAATACATGTTGCACACTTGCTTCTGGCCAATA
TGGTTGTTGTCCTCTGCCAAATGCAGTGTGTTGTAGTGATCATCTTCACTGCTGTCCATCAGGTTACACT
TGTGGTGTATCATCTGGCACATGCACACGAGGAGAGTCTGTTGTCTTATGGTATGAAAAGTCATCTGCCA
CTCCTGTGCACGTAGAAAGTGTTGTATGTCCTGATGGCCAATCAGAATGTCCAGATGGTAATACATGTTG
CAAACTTGCTTCTGGCCAATATGGTTGTTGTCCTCTACCAAATGCAGTGTGTTGTAGTGATCATCTTCAC
TGCTGTCCATCAGGATACACTTGTGGTGTATCATCTGGCACATGCACTCAAGGAGAGTCTGTTGTCCCTT
GGTATGAAAAGTCATCTGCCACTCCTGTGCACGTAGAAAGTGTTGTATGTCCTGATGGCCAATCAGAATG
TCCAGATGGTAATACATGTTGCACACTTGCTTCTGGCCAATATGGTTGTTGTCCTCTACCAAATGCAGTG
TGTTGTAGTGATCATCTTCACTGCTGTCCATCAGGATACACTTGTGGTGTATCATCTGGCACATGCACTC
AAGGAGAGTCTGTTGTCCCTTGGTATGAAAAGTCATCTGCCACTCCTGTGCACGAAGAAAATGTTGTATG
TCCTGATGGCCAATCAGAATGTCCAGATGGTAATACATGTTGCAAACTTGCTTCTGGCCAATATGGTTGT
TGTCCTCTACCAAATGCAGTTTGTTGTAGTGATCATCTTCACTGCTGTCCATCAGGATACACTTGTGGTG
TATCATCTGGCACATGCACTCAAGGAGAGTCTGTTGTCCCTTGGTATGAAAAGTCATCTGCCACTCCTGT
GCACGAAGAAAATGTTGTATGTCCTGATGGCCAATCAGAATGTCCAGATGGTAATACATGTTGCAAACTT
GCTTCTGGCCAATATGGTTGTTGTCCTCTACCAAATGCAGTGTGTTGTAGTGATCATCTTCACTGCTGTC
CATCAGGTTACACTTGTGGTGTTTCATCTGGCACATGCACACAGGGAGAGTCTGTTGTCCCTTGGTATGA
AAAGTCATCTGCCACTCCTGTGCTTGTAGAAAATGTTGTATGTCCTGATGGCCAATCAGAATGTCCAGAT
GGTAATACATGTTGCACACTTGCTTCTGGCCAATATGGTTGTTGTCCTCTGCCAAATGCAGTGTGTTGTA
GTGATCATCTTCACTGCTGTCCATCAGGATACACTTGTGGTGTATCATCTGGCACATGCACACGAGGAGA
GTCTGTTGTCTTATGGTATGAAAAATCATCTGCCACTCCAGTGCAAGTAGAAAATGTTGTATGTCCTGAT
GGCCAATCAGAATGTCCAGATGGTAATACATGTTGCAAACTTGCTTCTGGCCAATATGGTTGTTGTCCTC
TACCAAATGCAGTGTGTTGTAGTGATCATCTTCACTGCTGTCCATCAGGATACACTTGTGGTGTATCATC
TGGCACATGCACTCAAGGAGAGTCTGTTGTCCCTTGGTATGAAAAGTCATCTGCCACTCCTGTGCACGTA
GAAAATGTTGTATGCCCTGATGGCCAATCAGAATGTCCAGATGGTAATACATGTTGCACACTTGCTTCTG
GCCAATATGGTTGTTGTCCTCTACCAAATGCAGTTTGTTGTAGTGATCATCTTCACTGCTGTCCATCAGG
ATACACTTGTGGTGTATCATCTGGCACATGCACTCAAGGAGAGTCTGTTGTCCCTTGGTATGAAAAGTCA
TCTGCCACTCCTGTGCACGTAGAAAATGTTGTATGTCCTGATGGCCAATCAGAATGTCCAGATGGTAATA
CATGTTGCAAACTTGCTTCTGGCCAATATGGTTGTTGTCCTCTACCAAATGCAGTTTGTTGTAGTGATCA
TCTTCACTGCTGTCCATCAGGATACACTTGTGGTGTATCATCTGGCACATGCACTCAAGGAGAGTCTGTT
GTCTCTTGGTATGAAAAGTCAGCTGCCACTCCTGTGCAAGTAAAGAGTGTTATCTGTCCAGGAGGCCAAG
CACAATGCCCAGATGGTAATACATGTTGCAAACTTGCTTCTGGCCAATATGGTTGTTGTCCTCTGCCAAA
TGCAGTGTGTTGTAGTGATCATCTTCACTGCTGTCCATCAGGATACACTTGCGGTGTTTCTGCAGGAACC
TGCACACGAGGAGAGTCTGTTGTCTTATGGTATGAAAAATCATCTGCCACTCCAGTGCAAGTAGAAAATG
TTGTATGTCCTGATGGCCAATCAGAATGTCCAGATGGTAATACATGTTGCACACTTGCTTCTGGCCAATA
TGGTTGTTGTCCTCTACCAAATGCAGTTTGTTGTAGTGATCATCTTCACTGCTGTCCATCAGGATACACT
TGTGGTGTATCATCTGGTACATGCACACAGGGAGAGTCTGTGGTCTTATGGTTTGAAAAATCAGCTGCCA
CTCCTGTGCAAGTAGAGAGTGTTATCTGTCCAGGAGGCCAATCCGAATGTCCAGATGGTAATACATGTTG
CAAACTTGCTTCTGGTCAGTATGGTTGTTGCCCTCTACCAAATGCAGTTTGTTGTAGTGATCATCTTCAC
TGCTGTCCATCAGGATACACTTGTGGTGTATCATCTGGCACATGCACTCAAGGAGAGTCTGTTGTCCCTT
GGTATGAAAAGTCATCTGCCACTCCTGTGCACGTAGAAAATGTTGTTTGTCCTGATGGCCAATCAGAATG
TCCAGATGGTAATACATGTTGCAAACTTGCTTCTGGCCAATATGGTTGTTGTCCTCTACCAAATGCAGTT
TGTTGTAGTGATCATCTTCACTGCTGTCCATCAGGATACACTTGTGGTGTATCATCTGGCACATGCACTC
AAGGAGAGTCTGTTGTCTCTTGGTATGAAAAGTCAGCTGCCACTCCTGTGCAAGTAGAGAGTGTTATCTG
TCCAGGAGGCCAAGCACAATGCCCAGATGGTAATACATGTTGCAAACTTGCTTCTGGCCAATATGGTTGT
TGTCCTCTGCCAAATGCAGTGTGTTGTAGTGATCATCTTCACTGCTGTCCATCAGGATACACTTGTGGTG
TTTCTGCAGGAACCTGCACACGAGGAGAGTCTGTTGTCTTATGGTATGAAAAATCATCTGCCACTCCAGT
GCAAGTAGAAAATGTTGTATGTCCTGATGGCCAATCAGAATGTCCAGATGGTAATACATGTTGCAAGCTT
GCTTCTGGCCAATATGGTTGTTGTCCTCTACCAAATGCAGTTTGTTGTAGTGATCATCTTCACTGCTGTC
CATCAGGATACACTTGTGGTGTATCATCTGGTACATGCACACAGGGAGAGTCTGTGGTCTTATGGTATGA
AAAGTCAGCTGCCACTCCTGTGCAAGTAGAGAGTGTTCTCTGTCCAGGAGGCCAATCCGAATGTCCAGAT
GGTAATACATGTTGCAAACTTGCTTCTGGCCAGTATGGTTGTTGCCCTCTACCAAATGCAGTTTGTTGTA
GTGATCATCTACACTGCTGTCCATCAGGATACACTTGTGGTGTATCATCTGGCACATGCACTCAAGGAGA
GTCTGTTGTCCCTTGGTATGAAAAGTCATCTGCCACTCCTGTGCACGTGGAAAATGTTGTATGTCCTGAT

GGCCAATCAGAATGTCCAGATGGTAATACATGTTGCAAACTTGCTTCTGGCCAATATGGTTGTTGTCCTC
TACCAAATGCAGTGTGTTGTAGTGATCATCTTCACTGCTGTCCATCAGGATACACTTGTGGTGTATCATC
TGGCACATGCACTCAAGGAGAGTCTGTTGTCCCTTGGTATGAAAAGTCATCTGCCACTCCTGTGCAAGTA
GACAGTGTTATCTGTCCAGGAGGCCAAGCACAATGCCCAGATGGTAATACATGTTGCAAACTTCCTTCTG
GTCAATATGGTTGTTGTCCTTTACCAAATGCAGTGTGTTGTAGTGATGGTGAGCACTGCTGTCCGTCAGG
ATACACTTGCGATGTTTCTGCGGGAACCTGCACACAGGGAGAGTCTGTGGTCTTATGGTATGAAAAGTCA
TCTGCCACTCCAGTGCAAGTAGAGAGTGTTGTATGTCCAGGAGGCCAAGCACAATGCCCAGATGGTAATA
CATGTTGCAAACTTCCTTCTGGTCAATATGGTTGTTGTCCTTTACCAAATGCAGTGTGTTGTAGTGATGG
TGAGCACTGCTGTCCGTCAGGATACACTTGCGATGTTTCTGCAGGAACCTGCACACAAGGAGAGTCTGTT
GTTCCTTGGTATGAAAAGTCATCTGCCACTCCTGTGCAGATAGAGAGTGTTGTATGCCCTGATGGCCAAT
CAGAATGCCCAGATGGTAGTACATGTTGCAAACTTGCTTCTGGCCAATATGGTTGTTGTCCTATACCAAG
TGCAGTGTGTTGTAGTGATCATCTTCACTGCTGTCCATCAGGATACACTTGCGATGTTTCTGCAGGAACC
TGCACACAAGGAGAGTCTGTTGTTCCTTGGTATGAAAAGTCATCTGCCACTCCTGTGCAGATAGAGAGTG
TTGTATGCCCTGATGGCCAATCAGAATGCCCAGATGGTAGTACATGTTGCAAACTTGCTTCTGGTCAATA
TGGTTGTTGTCCTTTACCAAATGCAGTGTGTTGTAGTGATGGTCAGCACTGCTGTCCATCAGGATACACT
TGCGATGTTTCTGCAGGAACCTGCACACAAGGAGAGTCTGTTGTCCCTTGGTATGAAAAGTCATCTGCCA
CTCCAGTGTACGTAGAGAGTGTTGTATGTCCTGATGGCCAATCAGAATGCCCAGATGGTAATACATGTTG
CAAGCTTGCTTCTGGCCAATATGGTTGTTGTCCTCTACCAAATGCAGTTTGTTGTAGTGATCATCTTCAC
TGTTGTCCATCAGGATACACTTGTGGTGTATCATCTGGCACATGTACAAGGGGTAGCAGCATCTTGGAAT
GGAGTTCAAAGTCAAGTGCACGTTCTAATGTAGACAAACTTCAG

Coding exon 3 **Exon type: n**
ATTACGGCCACTGAACCAGTTAGCATCTGCCCAGATAGCAAGACATCATGCCCTTCATCAAGCACTTGTT
GCCCCCTCATGGCTTCAGGAGAATGGGGTTGCTGCCCCGTACATAAT

Coding exon 4 **Exon type: cn**
GCTGTGTGCTGTGATGACCACATTCACTGTTGCCCATCAGGCTACAGTTGCGGAACCAACTCCTGCCAGA
AGGGCTCCATGCACCTACCCCTTCTCTCCAAGTCCACTGGCCTCCTTAAGCTTACCTTCGAGGGCCAGGA
AGAACCAAAGCCAGAGGTCAAAGTTCATGATAATAAGCATCTCCTATTTCAGCCAGATGAACCACGGACA
GGATCCGTCATCTGCCCAGGAGGCAAAATAGAATGTCAAGATGGAAACGCTTGTTGCCTGTTAGCCGGCG
GTGATTATTCTTGTTGCCCTCTCCCTCAG

Coding exon 5 **Exon type: n**
GCGGTGTCCCCCCATGAGGAATCAGGTGATAAGATGATGTGTCCTGATGAGATCTCATCCTGTCCCACTG
GATCCACCTGCTGTCAGACACCAGGAAACATCTACGGCTGCTGCCCCTACCAAGCA

Coding exon 6 **Exon type: c**
Note: Up to the stop codon. At least 660 bases of 3'ut continues this exon.
GCTATCTGCTGCTCTGATCTGATACACTGCTGTCCAAGTGACTACTCCTGTAACAGCTCTACAGGAACAT
GCACCAAAGATGACCATATCATGCCATGGGTGGGAAAGATTCCACCTCTCTAA

Genomic sequence note: A genomic sequence is not presented (see note 2 in header).

00026 **S. salar progranulin 1** Short name: **S_sal1 or salmo1** Date entered: 2014-03

Species: ***Salmo salar (Atlantic salmon)***
Taxonomy (via NCBI): *Eukaryota; Metazoa; Chordata; Craniata; Vertebrata; Euteleostomi; Actinopterygii; Neopterygii; Teleostei; Euteleostei; Protacanthopterygii; Salmoniformes; Salmonidae; Salmoninae; Salmo.*
General Comment: Very closely related to salmoC. With the exon form sn-n-c, it lacks exons corresponding to the 2nd and 3rd of salmoC, which has the form sn-cn-c-n-c.

Protein Sequence
MWNIAALVLVVAGSASCYITCPDGKVCSDQSTCCLTKEGYACCPVTHQVPWTALVQASDSTPQAGVIRCD
TKFYCPSGTSCCKGPTGKWGCCPFPLGKCCADGQHCCEYGYTCDSSFKCRKGYSQIPSGLRDDAKQD*

Transcript:
Note: The transcript is very well defined by over 80 EST entries. One is given here as representative of the cDNA sequences. It covers the whole coding sequence of this small transcript.
The cDNA data define the transcript well (below), so no predicted sequence is required.

cDNA data:
From: NCBI est: DW583582.1 GI:85055404 with additional 5' and 3' ut
Note: This represents the cDNA sequence defined very welll by over 80 EST sequences. What is presented here is a contig of these sequences extending the untranslated sequences of the representative DW583582.1
CCCATTGTGCTGTGTGTAACCCCATGACAGGAAGTTTTAAATCCCTGAGCTGTGACTAACTACAGGATGT
GGAACATAGCTGCATTGGTGTTAGTGGTGGCAGGGTCTGCCTCTTGCTACATCACCTGCCCTGATGGGAA
GGTCTGCTCTGATCAATCAACCTGCTGTTTGACTAAAGAAGGATACGCCTGCTGTCCAGTTACCCATCAG
GTTCCGTGGACAGCACTAGTTCAGGCCTCTGACAGCACCCCACAGGCTGGAGTCATTCGCTGTGACACAA
AATTCTACTGCCCTTCTGGAACCAGCTGCTGCAAGGGACCGACTGGCAAATGGGGCTGCTGCCCATTCCC
ACTGGGCAAGTGCTGTGCAGATGGCCAGCATTGCTGTGAATATGGATACACCTGTGACTCCTCATTCAAG
TGCAGGAAAGGTTACTCTCAGATTCCTTCAGGTCTGAGGGATGATGCTAAGCAGGACTGACAAGATGTCT
TATTAATTATAATGTTGTCACTGTACTGGTATTGGATTGAAGAAACAGATTTGCTAGTTTAGCTAACCAA
AACATCAGTCCTAGCTTGCTATTATGAAAATCGAATGCAACAATGTTTTCAATTCTAATTTTGCTTTGAA
AAGCAACTCAAACATAAAACATGTAAAAATGAACTATAACCTTTGAATTCTACCCTGCAGATATACCATG
GTTTATAGGGAAATAATGCACGCTCTAGAATGCCCTTCATGACAATCAGAAACGAGTGTTCAACAATGTC
ACGGTATAACATTACATGTTGAAAGAATGTTGAATCATGTCACCCACTCATTCATTGCATAAAAGACATA
TCAATACACTTGAAATAAATGACATTCTAAGAAAAAACTGTT

Genomic data:
Note: Genomic data (exons).
From NCBI: AGKD01000506 REGION: 31000..33600
Note: gb|AGKD01000506.1|:31000-33600 Salmo salar Contig_000506, whole genome shotgun sequence
Coding exon 1 **Exon type: sn**
Note: from the start codon (which is the beginning of this exon)
ATGTGGAACATAGCTGCATTGGTGTTAGTGGTGGCAGGGTCTGCCTCTTGCTACATCACCTGCCCTGATG
GGAAGGTCTGCTCTGATCAATCAACCTGCTGTTTGACTAAAGAAGGATACGCCTGCTGTCCAGTTACCCA
T

Coding exon 2 **Exon type: n**
GTTCCGTGGACAGCACTAGTTCAGGCCTCTGACAGCACCCCACAGGCTGGAGTCATTCGCTGTGACACAA
AATTCTACTGCCCTTCTGGAACCAGCTGCTGCAAGGGACCGACTGGCAAATGGGGCTGCTGCCCATTCCC
ACTG

Coding exon 3 **Exon type: c**
Note: up to the stop codon.
GGCAAGTGCTGTGCAGATGGCCAGCATTGCTGTGAATATGGATACACCTGTGACTCCTCATTCAAGTGCA
GGAAAGGTTACTCTCAGATTCCTTCAGGTCTGAGGGATGATGCTAAGCAGGACTGA

Genomic sequence note: A genomic sequence is not presented (see note 2 in header).

00023 **S. salar progranulin A** Short name: **S_salA or salmoA** Date entered: 2014-03

Species: ***Salmo salar (Atlantic salmon)***
Taxonomy (via NCBI): *Eukaryota; Metazoa; Chordata; Craniata; Vertebrata; Euteleostomi; Actinopterygii; Neopterygii; Teleostei; Euteleostei; Protacanthopterygii; Salmoniformes; Salmonidae; Salmoninae; Salmo.*
General Comment: Transcript well defined by cDNA and EST data. Genomic sequence data is complete for the gene, but not yet properly assembled.

Protein Sequence
MQRYLVLCMALLAQVSADDCPGGEACEEGNTCCKVPSDGYECCPMYQAECCEDHIHCCPEGTLCVVNESM
CVNGTVSLPWVERVSAKQSIYPKSFRMISANAGDEDDNICPDKSHCPEEFSCLKATTGYGCCPVAHAVPC
SDGKHCCPEGHQCNTDCSSCIKQTEPVVAVMCSDRESECPEGTTCCETPDGSWGCCPMPKAVCCEDKIHC
CPEGSTCDIKQSKCISTTNKEMPMWAKFPARKRAEWETQNEVAQVTTPIPDTTTSANRSPASLWEGTSPP
FIRTGDVPCDESTACLDGTTCCKTQEGGWACCPLPQAVCCSDFVHCCPHGKKCNLAAQTCDDPSGSPSEP
WLKKVPAVPREGKLPGDVTCDPTHMCPDNTTCCKTASGDWACCPLPEAVCCEDHEYCCPHGTTCDLAGLT
CDGPSGSEPMVGKVQALTTLAPDHEEAATDQQGVQVDDMELPTKDDKDVPCDESTACLDGTTCCKTQEGG
WACCPLPQAVCCSDFVHCCPHGKKCNLAAQTCDDPSGSPSEPWLKKVPAVPREGKLPGDVTCDPTHMCPD
NTTCCKTASGDWDCCPLPEAVCCEDHEHCCPHGTTCDLATLTCDGPSGPEPMVGKVPALTTLAPDHEEAP
ADHQGVQMDDMELPTEDDESDEDEELHRTQCDAHTSCPKDATCCFMKSTQKWGCCPLPQAVCCADGEHCC

PKDYICDMSKTSCSKGGVVIPWYNKLAAEPHDIALTTPLSVKCDTQNRCPEGSSCCQLSTGQWGCCPLRK
AVCCADEEHCCPQGYSCNMGSGTCQKLMFLQFQTVPLTRVSAPEPPTPQEKEIHCGGPFVCNNEETCCKA
SATTWACCPVPNAVCCSDMKHCCTTGYTCGEGGSCTQSTGFKWDHWQVFFSNKKRALHV*

Transcript:

Note: An EST contig of over 40 sequences defines the transcript well. Since the full coding sequence is also in a cDNA entry, for simplicity it is that which is included in this list.

The cDNA data define the transcript well (below), so no predicted sequence is required.

cDNA data:

From: NCBI: BT059209.1 GI:223648327

Note: cDNA sequence data:

ATTGTCAAGGAGCGCGGACACCTGTAAAGTCACTGAGATAGAGATGCAGAGGTATCTGGTGCTCTGTATG
GCCCTGCTGGCCCAGGTCTCTGCTGATGACTGCCCAGGAGGAGAGGCGTGTGAGGAGGGCAACACCTGCT
GCAAGGTCCCCTCCGATGGCTATGAATGTTGCCCAATGTATCAGGCTGAGTGCTGTGAGGATCATATCCA
CTGCTGCCCTGAGGGAACACTCTGCGTTGTGAATGAGTCCATGTGTGTGAATGGAACTGTCTCCCTACCA
TGGGTGGAGAGAGTCTCTGCTAAACAGTCCATATATCCTAAATCTTTCAGAATGATCAGTGCAAATGCCG
GGGATGAGGATGATAACATCTGCCCTGACAAGTCCCACTGCCCCGAAGAGTTCTCCTGTCTGAAGGCCAC
TACAGGCTATGGTTGCTGTCCTGTTGCTCATGCTGTGCCTTGCTCTGATGGGAAACATTGCTGTCCTGAA
GGCCACCAGTGCAACACAGACTGCAGCTCCTGCATCAAACAGACAGAGCCTGTTGTTGCAGTGATGTGCA
GTGACAGAGAGTCTGAGTGTCCAGAGGGAACCACATGCTGTGAGACTCCAGATGGGAGTTGGGGGTGCTG
CCCCATGCCCAAGGCTGTGTGCTGTGAGGATAAGATTCACTGTTGTCCTGAGGGCAGCACCTGTGACATC
AAGCAATCCAAGTGTATATCCACTACCAACAAGGAAATGCCCATGTGGGCTAAGTTCCCTGCCCGCAAGA
GGGCAGAGTGGGAAACCCAGAATGAAGTTGCCCAAGTGACAACACCTATTCCAGACACTACCACCTCAGC
CAATAGGAGTCCTGCTTCTCTTTGGGAGGGTACTTCTCCACCCTTCATTAGGACAGGAGACGTTCCCTGT
GATGAGTCAACGGCCTGTTTGGATGGAACCACATGTTGTAAAACACAGGAAGGAGGATGGGCATGCTGTC
CTCTGCCACAGGCAGTTTGCTGCAGTGACTTTGTCCACTGCTGCCCACATGGTAAGAAGTGCAACCTGGC
TGCCCAGACGTGTGACGACCCCTCAGGCTCCCCCTCTGAGCCCTGGCTGAAGAAGGTTCCTGCCGTGCCT
CGGGAGGGTAAATTGCCAGGAGATGTTACCTGTGACCCCACCCACATGTGTCCCGACAACACCACCTGCT
GCAAGACAGCGTCAGGAGACTGGGCCTGCTGCCCCTTGCCTGAGGCTGTTTGCTGTGAGGACCACGAGTA
CTGCTGCCCCCATGGCACCACCTGTGACCTGGCTGGCCTCACCTGTGATGGTCCCTCAGGGTCGGAGCCT
ATGGTGGGGAAGGTGCAAGCTCTCACAACCCTGGCACCTGATCATGAAGAGGCAGCCACAGACCAGCAAG
GGGTGCAGGTGGATGACATGGAGCTGCCTACAAAGGATGACAAAGACGTTCCCTGTGATGAGTCAACGGC
CTGTTTGGATGGAACCACATGTTGTAAAACACAGGAAGGAGGATGGGCATGCTGTCCTCTGCCACAGGCA
GTTTGCTGCAGTGACTTTGTCCACTGCTGCCCACATGGTAAGAAGTGCAACCTGGCTGCCCAGACGTGTG
ACGACCCCTCAGGCTCCCCCTCTGAGCCCTGGCTGAAGAAGGTTCCTGCCGTGCCTCGGGAGGGTAAATT
GCCAGGAGATGTTACCTGTGACCCCACCCACATGTGTCCCGACAACACCACCTGCTGCAAGACAGCGTCA
GGAGACTGGGACTGCTGCCCCTTGCCTGAGGCTGTTTGCTGTGAGGACCACGAGCACTGTTGCCCCCATG
GCACCACCTGTGACCTGGCTACCCTCACCTGTGATGGCCCCTCAGGGCCAGAGCCGATGGTGGGGAAGGT
GCCCGCCCTCACAACCCTGGCACCTGATCATGAGGAGGCACCTGCAGACCACCAGGGGGTGCAGATGGAC
GACATGGAGCTGCCTACAGAGGATGATGAAAGTGACGAAGACGAGGAGTTGCACAGGACACAGTGTGACG
CCCACACCAGCTGCCCAAAGGACGCCACCTGCTGCTTTATGAAATCCACCCAGAAATGGGGCTGCTGCCC
ACTGCCACAGGCAGTGTGCTGTGCTGATGGAGAGCACTGCTGTCCCAAAGACTACATATGTGATATGAGC
AAGACTTCCTGCTCTAAGGGTGGGGTGGTGATCCCATGGTACAACAAGCTGGCAGCTGAGCCACATGACA
TTGCCCTTACTACCCCCCTCTCTGTGAAGTGTGACACCCAGAACAGATGCCCTGAAGGCTCCAGCTGCTG
CCAACTTTCCACAGGACAGTGGGGCTGCTGCCCCCTGCGCAAGGCTGTGTGCTGTGCAGATGAGGAGCAC
TGCTGTCCACAGGGCTACAGCTGTAACATGGGCTCAGGGACTTGCCAGAAGCTAATGTTTCTTCAGTTCC
AGACGGTACCCCTAACCCGGGTATCTGCGCCTGAGCCCCCGACCCCACAGGAGAAGGAAATCCACTGTGG
GGGGCCGTTTGTCTGCAATAATGAGGAGACATGCTGCAAGGCCTCCGCCACTACATGGGCTTGCTGTCCC
GTTCCAAATGCGGTGTGCTGCAGTGACATGAAGCACTGCTGCACTACAGGCTACACCTGTGGTGAGGGAG
GGTCCTGCACCCAGTCCACTGGCTTCAAATGGGACCACTGGCAGGTGTTCTTCTCCAACAAGAAGAGAGC
CCTGCATGTGTGACAACATTGGGCCAACCGTTGGAGTGATAGGCCCAAACTCCACCACCAGACGCATTGC
ACTAATGAAGGGACGCAGGAACTTAATATCACTGTTACAGTATATCAAATTGTGTTTTTTACAGAGGAAA
AATATTGTGTGCATATTTAGGAAAGGGAAGTGAAAATATGTAACATTTTGCACCATTGGTAGGTGCTTGG
AATGTTACTACATTTACTTCAAGAGATACCCATGCTTGCTCATGAACAGAAACTATCAAGATTGTCACAG
AGGAGCATATAAATGACAAGAACTTAGTACTGTTTTACAATGTTTGATGTTACCTTTTAAACAGGAAGTG
TGTTGTTTTTTAGTTGATCGTGTGAAGTGCATTGTGCCTCATAGTTTCAGGTATACCTTTGAAATGCTTT
TTACATGACATTTACATGACATTTTTACCTTCATTTAACTAGGAAAGTCAGTTAAGAACAAATTTTTGTT
CAGGGGCAGAACGACAGATTTTCACCTTGTCAACTCGGGGATTCGATTTTGCAACCTTTCGGTCACTAGT

CCAACACTCTAACCACTAGGCTACCTGCCGCCCCATATGCCCTCTGTCTTGTTGCTACCACGTTTTAAGT
AAGACATGGATTATCCCACTTCCTTGTGTTTTACAAATTATGCAATTTTAAATAGTCCGTAATGATTCTT
GCTGTTGCTACCAAAGGGAATTAATTAGCTGTAAAATGTGAATAAAAAAGTCATTGCCAAATCAGTTGTA

Genomic data:

Note: Genomic sequences defining exon structure are in (reverse complements) AGKD01037414.1, AGKD01029393.1, AGKD01030149.1, although there are errors, including an assembly error in AGKD01029393.1 after coding exon 13.

From NCBI: AGKD01037414.1 GI:354421622

Note: Contains coding exons 1 to 7 in the reverse complement of the first 3500 bases.

Coding exon 1 **Exon type: sn**

Note: from start codon (which is at the beginning of this exon)

ATGCAGAAGTATCTGGTGCTCTGTATGGCCCTGCTGGGCCAGGTCTCTGCTGATGACTGCCCAGGAGGAG
AGGCATGTGAGGAGGGCAACACCTGCTGCAAGGTCCCCTCTGATGGCTATGAATGTTGCCCAATGTATCA
G

Coding exon 2 **Exon type: c**

GCTGAGTGCTGTGAGGATCATATCCACTGCTGCCCTGAGGGAACACTCTGCGTTGTGAATGAGTCCATGT
GTGTGAATGGAACTGTCTCCCTACCATGGGTGGAGAGAGTCTCTGCTAAACAGTCCATATATCCTAAA

Coding exon 3 **Exon type: n***

TCTTTCAGAATGATCAGTGCATATGCCGGGGATGAGGATGATAACATCTGTCCTGACAAGTCCCACTGCC
CCGAAGAGTTCTCCTGTCTGAAGGCCACTACAGGCTATGGTTGCTGTCCTGTTGCTCAT

Coding exon 4 **Exon type: c***

GCTGTGCCTTGCTCTGATGGGAAACATTGCTGTCCTGAAGGCCACCAGTGCAGCACAGACTGCAGCTCCT
GCATCAAACAGACAG

Coding exon 5 **Exon type: n**

AGCCTGTTGTTGCAGTGATGTGCAGTGACAGAGAGTCTGAGTGTCCAGAGGGAACCACATGCTGTGAGAC
TCCAGATGGGAGTTGGGGGTGCTGCCCCATGCCCAAG

Coding exon 6 **Exon type: c**

GCTGTGTGCTGTGAGGATAAGATTCACTGTTGTCCTGAGGGCAGCACCTGTGACATCAAGCAATCCAAGT
GTATATCCACTACCAACAAGGAAATGCCCATGTGGGCTAAGTTCCCTGCCCGCAAGAGGGCAGAGTGGGA
AACCCAGAATG

Coding exon 7 **Exon type: j**

AAGTTGCCCAAGTGACAACACCTATTCCAGACACTACCACCTCAGCCAATAGGAGTCCTGCTTCTCTTTG
GGAGGGTACTTCTCCACCCTTCATTAGGACAGGAG

From NCBI: AGKD01029393.1

Note: Contains, with some sequence errors, coding exons 2 to 13 in the reverse complement, and then hits an assembly error with no further granulin gene sequence beyond. Because of errors in exon 2 and several in exon 6, only exons 8 and 9 are given. The remaining exons are reported from AGKD01030149.1 following this.

Coding exon 8 **Exon type: n**

ACGTTCCCTGTGATGAGTCAACGGCCTGTTTGGATGGAACCACATGTTGTAAAACACAGGAAGGAGGATG
GGCATGCTGTCCTCTGCCACAG

Coding exon 9 **Exon type: cn**

GCAGTTTGCTGCAGTGACTTTGTCCACTGCTGCCCACATGGTAAGAAGTGCAACCTGGCTGCCCAGACGT
GTGACGACCCCTCAGGCTTCCCCTCTGAGCCCTGGCTGAAGAAGGTTCCTGCCGTGCCTCGGGAGGGTAA
ATTGCTAGGAGATGTTACCTGTGACCCCACCTACATGTGTCCCGACAACACCACCTGCTGCAAGACAGCG
TCAGGAGACTGGGCCTGCTGCCCTTTGCCTGAG

From NCBI: AGKD01030149.1

Note: Includes coding exons 10 to 16 in the reverse complement. This overlaps the progranulin gene sequence in AGKD01029393.1 reverse complement by 4 exons and correctly extends the sequence to the final coding exon of this gene.

Coding exon 10 **Exon type: c**

GCTGTTTGCTGTGAGGACCACGAGTACTGCTGCCCCCATGGCACCACCTGTGACCTGGCTGGCCTCACCT

GTGATGGTCCCTCAGGGTCGGAGCCTATGGTGGGGAAGGTGCAAGCTCTCACAACCCTGGCACCTGATCA
TGAAGAGGCAGCCACAGACCAGCAAGGGGTGCAGGTGGATGACATGGAGCTGCCTACAAAGGATGACAAA
G

Coding exon 11 **Exon type: n**
ACGTTCCCTGTGATGAGTCAACGGCCTGTTTGGATGGAACCACATGTTGTAAAACACAGGAAGGAGGATG
GGCATGCTGTCCTCTGCCACAG

Coding exon 12 **Exon type: cn**
GCAGTTTGCTGCAGTGACTTTGTCCACTGCTGCCCACATGGTAAGAAGTGCAACCTGGCTGCCCAGACGT
GTGACGACCCCTCAGGCTCCCCCTCTGAGCCCTGGCTGAAGAAGGTTCCTGCCGTGCCTCGGGAGGGTAA
ATTGCCAGGAGATGTTACCTGTGACCCCACCCACATGTGTCCCGACAACACCACCTGCTGCAAGACAGCG
TCAGGAGACTGGGACTGCTGCCCCTTGCCTGAG

Coding exon 13 **Exon type: cn**
GCTGTTTGCTGTGAGGACCACGAGCACTGTTGCCCCCATGGCAGCACCTGTGACCTGGCTACCCTCACCT
GTGATGGTCCCTCAGGGCCAGAGCCGATGGTGGGGAAGGTGCCCGCCCTCACAACCCTGGCACCTGATCA
TGAGGAGGCACCTGCAGACCACCAGGGGGTGCAGATGGACGACATGGAGCTGCCTACAGAGGATGATGAA
AGTGACGAAGACGAGGAGTTGCACAGGACACAGTGTGACGCCCACACCAGCTGCCCAAAGGACGCCACCT
GCTGCTTTATGAAATCCACCCAGAAATGGGGCTGCTGCCCACTGCCACAG

Coding exon 14 **Exon type: cn**
GCAGTGTGCTGTGCTGATGGAGAGCACTGCTGTCCCAAAGACTACATATGTGATATGAGCAAGACTTCCT
GCTCTAAGGGTGGGGTGGTGATCCCATGGTACAACAAGCTGGCAGCTGAGCCACATGACATTGCCCTTAC
TACCCCCCTCTCTGTGAAGTGTGACACCCAGAACAGATGCCCTGAAGGCTCCAGCTGCTGCCAACTTTCC
ACAGGACAGTGGGGCTGCTGCCCCCTGCGCAAG

Coding exon 15 **Exon type: cn**
GCTGTGTGCTGTGCAGATGAGGAGCACTGCTGTCCACAGGGCTACAGCTGTAACATGGGCTCAGGGACTT
GCCAGAAGCTAATGTTTCTTCAGTTCCAGACGGTACCCCTAACCCGGGTATCTGCGCCTGAGCCCCCGAC
CCCACAGGAGAAGGAAATCCACTGTGGGGGGCCGTTTGTCTGCAATAATGAGGAGACATGCTGCAAGGCC
TCCGCCACTACATGGGCTTGCTGTCCCGTTCCAAAT

Coding exon 16 **Exon type: c**
Note: up to the stop codon
GCGGTGTGCTGCAGTGACATGAAGCACTGCTGCACTACAGGCTACACCTGTGGTGAGGGAGGGTCCTGCA
CCCAGTCCACTGGCTTCAAATGGGACCACTGGCAGGTGTTCTTCTCCAACAAGAAGAGAGCCCTGCATGT
GTGA

Genomic sequence note: This Salmo salar progranulin A gene sequence including known transcript was generated by combining AGKD01037414.1:1..3500_rc, AGKD01029393.1:17314..17867_rc, AGKD01030149.1:9871..15124_rc in that order, where _rc denotes reverse complement sequence.
CAGCGCGTTCAGGAGTCGGGTACATTGTCAAGGAGCGCGGACACCTGTAAAGTCACTGAGATAGAGGTAA
GGTCAACTTTTTTTATTTATATAGTAGCAAGATCTCAATGACAAATATATTCAATTCTAAACTTCTCAAG
TGAAATTGCACCAAATGTTCTACTTCAAATTAAATTGCATATGTTGTTTATGCTATCCTTGTCATTGCTT
TTAAATTGTTGTCATTGTTCATTGTCTTTTGTGATAACAATTTCAAATCATTTAATATGGACCTAGGTGG
GTGGGTCCATCTTTTAAAATTCTAAATGCACATTATGACCAACGTGATTGAGTGTGTGCCAATATAAAAA
GAAGCATGGTGATATATCAGGGTCCACAGTTCTTCTGAAACTGTTAAAGTCCATTTTCTTCTCCTTTTGA
AACCACCTTGTGACAAAATACAGTTAGTGAACAGTACTGATTGTCAGGTGAATATTGTCTGTGGTTAATA
CACCTGTGTTCCCTATTGCTCTTGATACCACAATGAAGGAAAGTATCTCTTATGGGAGGTACTTGGCTCA
GATTAAGGGGAATTTTCCTTTCATAAAAACAGGTAAACCGAAAGTAGAAGTGCTGTATGCTGCATCATTT
TTGTGTCTAGTAGCCTAAACAACATTGAAGCAATGGTAGACTGCTTTAATGATCTCCCCTATGAAGCTCA
ACACTTAGTAACAGTATAATCACATCCCCATCCCTGCATCATAGATTCATTCCTTAAATGTGGTTGCATA
TGAGGAGGGCTTTGTATTGGTAAGAATGTGGAACTATTAACTGTCTGAAGCTCACCCCATGGAACATAAA
AGAGGAAGTGAATCTATGAAAATAGCTTGGGGAATTTTGATATACTTTAGTAGGCTACAGCAGTACCTCA
GGAGATTGTCTGAAAGTTACTCTAGTTAAGAGGAAAGTGAGAGTTCTGTCTAGATTTAGTTCTGATGACT
CCATTGTTGCGATTTTATGCGTTTCTGTACAAAAGAGCTTTTATAACATTTGTCAGTTATTGATTGTACA
ATGCATGTATATAATATACAGTTTGTGTTAGAAGTTATTTCTGTCTCTCTCTCTCCAGATGCAGAAGTAT
CTGGTGCTCTGTATGGCCCTGCTGGGCCAGGTCTCTGCTGATGACTGCCCAGGAGGAGAGGCATGTGAGG
AGGGCAACACCTGCTGCAAGGTCCCCTCTGATGGCTATGAATGTTGCCCAATGTATCAGGTATGTATGGG
GTGGTGCCTGCAGAGCTTAGCCCTTAGCTTTATTTACAGCCTGAACCCGAGTTCCCTAACCCTTGTACAC

TGAATTATATCTATAAGAATTGTACAAACCAAATAGGTAGACTATGGTGTCATTGTCTCTTACCCTGAAA
TAGATACCTCTTTATTGCACCCTTGTGCTAGCATTCTCTCGTATAATCTGTTTGGTCTGTAAGATGTGAG
ATTCTAACACACTTTGTTAATCAAACTACTCTCTTACCATTTATCTCAGGCTGAGTGCTGTGAGGATCAT
ATCCACTGCTGCCCTGAGGGAACACTCTGCGTTGTGAATGAGTCCATGTGTGTGAATGGAACTGTCTCCC
TACCATGGGTGGAGAGAGTCTCTGCTAAACAGTCCATATATCCTAAAGTAAGAATGTCATTGAATCCAGG
AAGAAAGTGTAGCTGACTATTGACAACGTTGAAATGCACTGTAACAATGACACCAGTTGACTTTCATGGC
AATATGTGTCCCTGTTTTCAATCATTCACATTGTTGAGGAATCAGGCAGAACTCCGTTATCAGCAATAAG
ATTTTATTCAAGCAATTGCAGGGACGATCACTTTCTGGATGAAACTGGAGAGGAACTTAAAAATGTCCAG
AATCACACAGTCATTTATATTCTTCATCTACACAAAGAACTGCTTACCCTTCTCTTAAAGCTATATACAA
ACAAGAATCGGTTCTAACCAGTTCTCACAGCCTAAGATAGGGGAGTGATAAGATCTTTAAAGCCTAAACA
GACAGAACTAGAATTCAGCCTGGTTACGCCTTTAGTGCCCCAGAGATAAGGGGCCAAAAACAAATCAATC
CTTTGTGCGGTGTGTGAAGAGGGTACTGAAAATTAACAGTGAAAAATACATACAGTACTATTCAACTCAA
GCATTTACATAGCATTAGACTGTAACAAACCATTTTGCCCCAACAACATAATTAACCTACATGAACGGAA
GGTAATCAAAAATACTATGATCAGTCATGTTTTTTGTAATTGAAAAAGTAAAATGTATTTTTTTCTACCC
CTCTAGTCTTTCAGAATGATCAGTGCATATGCCGGGGATGAGGATGATAACATCTGTCCTGACAAGTCCC
ACTGCCCCGAAGAGTTCTCCTGTCTGAAGGCCACTACAGGCTATGGTTGCTGTCCTGTTGCTCATGTAAT
AACCTAAACACTGTTGCAATCTAGCCTTTTCCGCCAGATTCATACTGTTATACCGTTCACATAAACATGC
ATTGGTGTCAATGGAAAAACTGGAGTTATGTGCACAGATATCAAGTTAGGATGCAGGCCTGAGTGAATAG
TAAATTGATCTAATGTACACACAGGCTGTGCCTTGCTCTGATGGGAAACATTGCTGTCCTGAAGGCCACC
AGTGCAGCACAGACTGCAGCTCCTGCATCAAACAGACAGGTTAGACTCTTGTGGAGTCACGTGTTGCAAT
CTTATGCTTCTGTTGCAACATCATCACTAACCCCTCTGCCTTCTTCCACCCCGGCAGAGCCTGTTGTTGC
AGTGATGTGCAGTGACAGAGAGTCTGAGTGTCCAGAGGGAACCACATGCTGTGAGACTCCAGATGGGAGT
TGGGGGTGCTGCCCCATGCCCAAGGTGGGGGGGCTATATGGAGGGGGCTAGGTGGTTACCATCATGGATT
TGCGAGTCAGGAATAATATTGGTCCTTTGAATGTGTGACATGTACACTCTCCAGTGTCTGTAATCACTCC
ATGGAGAGATGTCTAATCGAGGATTTGCATGGCCTATGTGCTAACCCTTTGTTTATATATGGTCATCTCA
TGAGGTGATTCTCTGTGTGTCTGTGTGCACTGACCAGGCTGTGTGCTGTGAGGATAAGATTCACTGTTGT
CCTGAGGGCAGCACCTGTGACATCAAGCAATCCAAGTGTATATCCACTACCAACAAGGAAATGCCCATGT
GGGCTAAGTTCCCTGCCCGCAAGAGGGCAGAGTGGGAAACCCAGAATGGTAGGTTTCACCCCCCTGCCCC
CCCTCCAAAAGGCATATTTTCCTGGTCTATTTTTGTCTGGATGTTAATGTGTATATGATTATTTTGCTGT
ACGTGCTTCCAGAAGTTGCCCAAGTGACAACACCTATTCCAGACACTACCACCTCAGCCAATAGGAGTCC
TGCTTCTCTTTGGGAGGGTACTTCTCCACCCTTCATTAGGACAGGAGGTATGACATTACACTTCTTCTTA
gaacaatacaatagtgttgtggaatatttgttagctcgtccagttgtgttgatcgctaacaccctgctg
tctctccagacgttccctgtgatgagtcaacggcctgtttggatggaaccacatgttgtaaaacacagga
aggaggatgggcatgctgtcctctgccacaggtatactatgtctcatactgtaggggcccattcactctc
ttcgctaatcaatttctttaagatagatcaacctgattgcatgtcttttgcttgtcccgtgacagcttcc
cctttttgtttgggttcttgtctcttccaggcagtttgctgcagtgactttgtccactgctgcccacatg
gtaagaagtgcaacctggctgcccagacgtgtgacgacccctcaggcttcccctctgagccctggctgaa
gaaggttcctgccgtgcctcgggagggtaaattgctaggagatgttacctgtgaccccacctacatgtgt
cccgacaacaccacctgctgcaagacagcgtcaggagactgggcctgctgccctttgcctgagGTAGGGG
ACAAATAAATATGGCTACTACTGCTGTGGATAGGCACATTAATTGAATGGCCTCTTAACTTGACTGCTCT
GCGATGCGGTGAGAGATTCTGTATGGCTAACAAAGAGGCACTTAGTTTTGGTCTTTACGACATCCCAGTA
TCACATCCTCATTCTTTACTCCTGTCAGCTTGCTGCTGCTTGTGATCTTAACATTTCTTCTGCCTTGTAA
AGGGGTTTTGGAGCTTGAACCACATGTTGTTTTATAGCAAGGGGGGCTATGTTTCATTGAAAAGTAGAAC
TGGGAAACTGGGTAATATAGGGAGTCACAACTTTTCTCTAACTTGCATAATCTCTTCTGCAACCAGCAAT
ACTTCGACATAATTTCTATAAAGGCTTGTGACAGTCTTAAATGACAGAAAATGTTTTGAGTGTCCTTTCC
CTTCCTCCATCCAGGCTGTTTGCTGTGAGGACCACGAGTACTGCTGCCCCCATGGCACCACCTGTGACCT
GGCTGGCCTCACCTGTGATGGTCCCTCAGGGTCGGAGCCTATGGTGGGGAAGGTGCAAGCTCTCACAACC
CTGGCACCTGATCATGAAGAGGCAGCCACAGACCAGCAAGGGGTGCAGGTGGATGACATGGAGCTGCCTA
CAAAGGATGACAAAGGTGATGAAGACGAGGAGGGGCCCAGGACACAGTATGACGCCCACACCACCTGCTG
CTTTATTAAATATTTTGTTAGCTCATCCAGTTGTGTTGATCGCTAACACCCTGCTGTCTCTCCAGACGTT
CCCTGTGATGAGTCAACGGCCTGTTTGGATGGAACCACATGTTGTAAAACACAGGAAGGAGGATGGGCAT
GCTGTCCTCTGCCACAGGTATACTATGTCTCATACTGTAGGGGCCCATTCACTCTCTTAGCTAATCAATT
TCTTCAAGAGAGATCAACCTGATTGCATGTCTTTTGCTTGTCCCGTGACAGCTTCCCCTTTTTGTTTGGG
TTCTTCTCTCTTCCAGGCAGTTTGCTGCAGTGACTTTGTCCACTGCTGCCCACATGGTAAGAAGTGCAAC
CTGGCTGCCCAGACGTGTGACGACCCCTCAGGCTCCCCCTCTGAGCCCTGGCTGAAGAAGGTTCCTGCCG
TGCCTCGGGAGGGTAAATTGCCAGGAGATGTTACCTGTGACCCCACCCACATGTGTCCCGACAACACCAC
CTGCTGCAAGACAGCGTCAGGAGACTGGGACTGCTGCCCCTTGCCTGAGGTAGGGACAAATAGATATGGC
TACTACTGCTGTGGATAGGCACATTAATTGAATGGCCTCTTAACTTGACTGCTCTGCGATGCGGTGAGAG
ATTCTGTATGGCTAACAAAGAGGCACTTAGTTTTGGTCTTTACGACATCCCATTATCACTTCCTCATTCT
TTACTCCTGTCAGCTTGCTGCTGCTTGTGATCTTAACCTTTCTTCTGCCTTGTAAAGGGGTTTTGGAGCT

TGAACCACATGTTGTTTTATAGCAAGGGGGGCTATGTTTCATTGAAAAGTAGAACTGGGAAACTGGGTAA
TATAGGGAGTCACAACTTTTCTCTAACTTGCATAATCTCTTCTGCAACCAGCAATACTTCTACATAATTT
CTATAAAGGCTTGTGACAGTCTTAAATGACAGAAAATGCTTGTTGTGTGTCCTTTCCCTTCCTTAAGGCT
GTTTGCTGTGAGGACCACGAGCACTGTTGCCCCCATGGCAGCACCTGTGACCTGGCTACCCTCACCTGTG
ATGGTCCCTCAGGGCCAGAGCCGATGGTGGGGAAGGTGCCCGCCCTCACAACCCTGGCACCTGATCATGA
GGAGGCACCTGCAGACCACCAGGGGGTGCAGATGGACGACATGGAGCTGCCTACAGAGGATGATGAAAGT
GACGAAGACGAGGAGTTGCACAGGACACAGTGTGACGCCCACACCAGCTGCCCAAAGGACGCCACCTGCT
GCTTTATGAAATCCACCCAGAAATGGGGCTGCTGCCCACTGCCACAGGTGAGTGTTACAGTAATAGTTAT
TGCTGAGCAATTGGTGCTTTTGAGGTCTGTTCAGTTTCGGTTCGATTATAAATAAATAATAACGGATTTC
GGTTTCGATTATTTGGGTTGAATGCTGTAACAGCACAGAATATAACAATACAAGTCCCATGGTGGAAGTG
ACTGCCCATTACTGCTTATCACTTATCACATTGCTTTACTTTAATAAAATATTACTTTTTGTGTATATTA
CATTTGTTTTAATTTAAGAACTTTATTTCATTCCAATTCATCATTTCATCTCTATAGAGCTGCTGCCTAT
GCTGTCTGACAAAATCAAAAACATTTTAGTTCTTCAACATAAATAAGGCATACTTTTATGACTGCTGAAT
ACCAACTATCAATTACTTTGATCATGTATTTTCAGGTAGAGATACCTCGCGAAGCAACAGCTGCTCCAAA
GACAGTACAGTATGAAGATAGGCAGAAACTGCTTCTCCAATAGAAATATCCAATCATGCTTGTAGGCGAT
GTCATGGCGACATTGGCTAGCTAAGCTCGTTCAGAAACGTCATTGGGTCTAACGGGCATCACGTGCCGAA
CTGCGCATGTTCATGCCATCAAATCAAAGGCACTTCTTCAATATAGTAATTTTTGATTAAAATGAAAACG
TGTAAGATTGTCACTTTGGAGTAATAACTTGTTCAACTATAAAAAGGCATTGGCTCGAATCTAGGTTGTA
CCTTTTAGATTTGGATGAAAAATGTAAGGACACTTTTTTTTTTTTACTTGTCCCATTGACTTCTCAAACC
CCAGCCTGGTCTGCTTCTCAAGCGCTCTCTATATCCCTTCCGTAGCAGGCATAAAAGAAACACAGACCGG
ACAAGTCGATGCGCAATGGATTATAGTCATTGTAGTTAATTACAATGTTTTATGCGCTAAACTATGTAGA
ATATTGGCCTGTTGGAAACAACTCCCTACAACATCTCACAGTTCAGGCTGGATCTGATTTATCTCTAAAG
GAACTGCAACGTGCGCATTGAGCTCATAAAAAAAATCTGAACAAAATGGAATTAAAATAATTGAACTGACA
TCGGTCAATTAGTTGTTTGATAACCAACATGTCAATCACTCAGCACTATTAATGGTGTTCCCAACTAGTG
AGGAAGGATGTTAGGGAATGAAAGCCCTTTCCCTCACTACTCGCTGTTCCCTCTTCCCCAGGCAGTGTGC
TGTGCTGATGGAGAGCACTGCTGTCCCAAAGACTACATATGTGATATGAGCAAGACTTCCTGCTCTAAGG
GTGGGGTGGTGATCCCATGGTACAACAAGCTGGCAGCTGAGCCACATGACATTGCCCTTACTACCCCCCT
CTCTGTGAAGTGTGACACCCAGAACAGATGCCCTGAAGGCTCCAGCTGCTGCCAACTTTCCACAGGACAG
TGGGGCTGCTGCCCCCTGCGCAAGGTGTGTGTTTGTGTGAGCGAGGGTGTGTGTTGGTATGCACATGCGT
ATAGAGAACCCTAGAAACAGTTTTGCTCTGCTATCTCACATGGGGAACCTTTCATGTCTGTACCACATCT
GGCCGTGATTGGGAGTCCCATAGGGCAGCGCACAATTGGCCCAGCGATTGTCCGGGTTTGGCCGGGGTAG
GCTGTCATTGTAAATAAGAATTTGTTCTTAACTGACTTGCCTAGTTAAATAAAACTAATTTGTCTCACTG
TATAGGCTGTGTGCTGTGCAGATGAGGAGCACTGCTGTCCACAGGGCTACAGCTGTAACATGGGCTCAGG
GACTTGCCAGAAGCTAATGTTTCTTCAGTTCCAGACGGTACCCCTAACCCGGGTATCTGCGCCTGAGCCC
CCGACCCCACAGGAGAAGGAAATCCACTGTGGGGGGCCGTTTGTCTGCAATAATGAGGAGACATGCTGCA
AGGCCTCCGCCACTACATGGGCTTGCTGTCCCGTTCCAAATGTATGATATACACCTCCGCCCTCTCACCA
CAGACACCACAAACGCTTCTAGAAATGAAAATGTCATACTAATGTTAGTGGAGTTCAAGACTAAGGCATC
TACTCTCCTTCTATTGAATAGTCAAATTCCTATATGATTTCTCTCATTGCTTCATCGATGTCTTTTTTTT
TACTCTCCCTTCCACCTTATCCCTGTCTTCCTCCACCTGTCCTCCTAGGCGGTGTGCTGCAGTGACATGA
AGCACTGCTGCACTACAGGCTACACCTGTGGTGAGGGAGGGTCCTGCACCCAGTCCACTGGCTTCAAATG
GGACCACTGGCAGGTGTTCTTCTCCAACAAGAAGAGAGCCCTGCATGTGTGACAACATTGGGCCAACCGT
TGGAGTGATAGGCCCAAACTCCACCACCAGACGCATTGCACTAATGAAGGGACGCAGGAACTTAATATCA
CTGTTACAGTATATCAAATTGTGTTTTTTACAGAGGAAAAATATTGTGTGCATATTTAGGAAAGGGAAGT
GAAAATATGTAACATTTTGCACCATTGGTAGGTGCTTGGAATGTTACTACATTTACTTCAAGAGATACCC
ATGCTTGCTCATGAACAGAAACTATCAAGATTGTCACAGAGGAGCATATAAATGACAAGAACTTAGTACT
GTTTTACAATGTTTGATGTTACCTTTTAAACAGGAAGTGTGTTGTTTTTTAGTTGATCGTGTGAAGTGCA
TTGTGCCTCATAGTTTCAGGTATACCTTTGAAATGCTTTTTACATGACATTTACATGACATTTTTACCTT
CATTTAACTAGGAAAGTCAGTTAAGAACAAATTCTTGTTCAGGGGCAGAACGACAGATTTTCACCTTGTC
AACTCGGGGATTCGATTTTGCAACCTTTCGGTCACTAGTCCAACACTCTAACCACTAGGCTACCTGCCGC
CCCATATGCCCTCTGTCTTGTTGCTACCACGTTTTAAGTAAGACATGGATTATCCCACTTCCTTGTGTTT
TACAAATTATGCAATTTTAAATAGTCCGTAATGATTCTTGCTGTTGCTACCAAAGGGAATTAATTAGCTG
TAAAATGTGAATAAAAAAGTCATTGCCAAATCAGTTGTACAATATGCACGTGTTGGTTTGTAATCAGTGT
TCACGCAAATCTGTGCCCAGGATGTAACTATAGTAGGCCTTGCAAGTAAAAAGGAAAAAACTCAAGAGGT
GATAAAGGTGAGTACTGAGTGGACTACAGTTGGAGAAAAACCACATTCAACGTCTGATACTAATTAC

00024 **S. salar progranulin B** Short name: **S_salB or salmoB** Date entered: 2014-03

Species: ***Salmo salar (Atlantic salmon)***
Taxonomy (via NCBI): *Eukaryota; Metazoa; Chordata; Craniata; Vertebrata; Euteleostomi; Actinopterygii; Neopterygii;*

Teleostei; Euteleostei; Protacanthopterygii; Salmoniformes; Salmonidae; Salmoninae; Salmo.
General Comment: Genomic sequences containing granulin exons predict two variants of the Salmo salar B form. The transcript for the one we worked with is fully defined by sequences in the NCBI EST database.

Protein Sequence

MIGVVCLALLGLTSALICPDGGMCDDENTCCKTPSGGYGCCPLPNAECCSDHLHCCYEGTVCDLVHSKCL
NKTVSLPWVRRVPAQHIISPLMVEGVRAVICPDGESECPDDTTCCQLPDGSWGCCPLAKAVCCEDKMHCC
PEGTKCDLAHSKCVSPTLETFAMREKGPAMKKQTVTCPGGTSRCPDRTTCCLLTIGDYGCCPYLEAVCCT
DKLHCCPGNTTCDLEHDICTSPNTQTPLAKKTPAVPNDVDCPDKVSSCPDETTCCLLGDRSYGCCPMPSA
VCCPDHIHCCPEGTTCDLEQSTCVSEHGQTSMAVKFPATLTTTSLQSTVNAVPCNDSVACADGTTCCKLL
NGEWACCPLPKAVCCDDHLHCCPHGTICNLAESTCDDPSSGSALVPMLDKVPAFSYVSQEEPLPNSICDK
STLCPGKSTCCKTTTGNWACCPLPNAVCCDDHLHCCPHGTVCNLEASSCDDPLGFNMPWVTKVPALTTQA
QLATEKCDEQTICPGGTTCCRQNSGQWACCPLPHAVCCDDHEHCCPKGYTCNVAEQTCDKAGTLSLPWIP
KVPALLLHRVLPQASAPSFPLAKNMCDPHTSCPKYTTCCFMNKSGKWGCCPLPKAVCCANGDHCCPSGYS
CDVHKTSCTKGPLTIPWYRKEKALTEGAMLKDVKCDDKSSCASGATCCKLPTGEWGCCPLVKAVCCTDHE
HCCPQGYSCNMQTGTCEKLVEAIVLSTVPLTKVAESQQRAAEMSIDVKCDSTGEYSCPKLETCCKTSPTE
WSCCPAPKAICCTDTRHCCPMGYTCDLERGGCSQQAELTWDMFYSHDKKKDFVPF*

Transcript:

Note: Transcript sequence is well supported by over 16 EST entries.
The cDNA data define the transcript well (below), so no predicted sequence is required.

cDNA data:

From: NCBI: NM_001140047.1 GI:213511241
Note: A representative cDNA sequence well supported by EST data (contig of GI:89867824, 213068489, 213069977, 213076305, 89857615, 213075008, 85053963, 24342034, 85019727, 213077774, 84971965, 85044879, 89853382, 45309780, 117848278, 85053563)
AGTTGGAGTGTCGCTTCGAATGAAAGATTATTTAACTAGTTTGAGCTTTTACATCGCACACTAACGAAGA
CTATATGAAGACTATGCATTGCAGTTCCCAACTTGAATAACACATCTGACGATATTCCCTCATCTGCCCT
CTTGCTCTTCTCAGATTCTCTGAACACAATGATAGGGGTGGTATGTCTGGCCCTCCTAGGCCTGACCTCA
GCCCTGATTTGTCCCGATGGTGGGATGTGTGACGACGAGAACACCTGCTGCAAGACCCCCAGCGGTGGAT
ATGGCTGCTGTCCATTACCAAATGCTGAGTGCTGTTCCGACCACCTGCATTGCTGCTATGAGGGGACAGT
GTGTGACTTAGTCCACTCCAAGTGCCTCAATAAAACAGTCTCTCTGCCGTGGGTCAGAAGAGTCCCCGCA
CAACACATCATCAGCCCTCTGATGGTGGAGGGAGTGAGGGCAGTAATTTGTCCCGACGGCGAGTCGGAGT
GTCCGGACGACACCACCTGCTGTCAACTCCCAGATGGCTCCTGGGGCTGCTGTCCGCTGGCCAAGGCAGT
GTGCTGTGAGGACAAGATGCACTGCTGTCCAGAGGGCACCAAGTGTGATCTAGCCCACTCCAAGTGTGTG
TCTCCCACCCTGGAGACCTTTGCCATGAGGGAAAAGGGGCCTGCAATGAAGAAGCAGACGGTGACCTGCC
CAGGTGGTACGAGCAGATGCCCAGACAGAACCACCTGTTGCCTGCTGACCATTGGCGACTATGGCTGCTG
CCCCTACCTGGAGGCTGTGTGCTGTACCGACAAACTCCACTGTTGCCCGGGCAATACAACCTGTGACCTG
GAGCATGATATATGCACTTCCCCCAACACACAGACCCCACTGGCTAAGAAGACCCCTGCCGTCCCCAACG
ATGTGGATTGCCCAGACAAGGTGTCATCGTGTCCTGATGAGACCACTTGCTGCCTGCTGGGGGATAGGAG
CTATGGCTGCTGCCCTATGCCAAGTGCTGTGTGTTGTCCAGACCACATCCACTGCTGTCCTGAGGGAACC
ACCTGTGACCTGGAACAGAGCACCTGTGTGTCTGAACACGGCCAAACTTCCATGGCTGTCAAATTCCCCG
CCACATTGACCACGACATCCCTACAGAGCACAGTCAATGCTGTGCCCTGCAACGACTCTGTGGCCTGCGC
TGATGGAACCACGTGCTGTAAATTACTAAATGGAGAATGGGCCTGCTGCCCGCTACCCAAGGCTGTGTGT
TGTGATGACCACCTCCACTGCTGCCCCCACGGGACCATCTGTAACCTGGCAGAGAGTACGTGTGATGACC
CCTCTTCGGGCTCTGCCCTGGTACCCATGCTGGACAAGGTGCCCGCCTTTAGCTATGTGTCGCAGGAGGA
GCCGCTGCCCAACAGCATATGTGACAAATCCACGTTGTGCCCGGGCAAATCCACGTGCTGCAAGACCACC
ACAGGAAACTGGGCATGCTGCCCCCTGCCCAATGCTGTGTGTTGCGATGACCACCTCCACTGCTGCCCCC
ACGGCACTGTGTGTAACCTGGAGGCCAGTAGCTGCGATGACCCCTTAGGCTTCAACATGCCGTGGGTCAC
CAAGGTGCCAGCACTCACCACCCAGGCACAACTGGCCACTGAGAAGTGTGACGAACAGACCATTTGCCCC
GGGGGCACCACCTGCTGCAGACAGAACTCAGGACAGTGGGCATGCTGTCCTCTACCTCATGCGGTGTGCT
GCGATGACCACGAGCACTGCTGCCCTAAAGGCTACACGTGTAATGTGGCCGAGCAGACCTGTGACAAAGC
TGGGACCCTCAGCTTGCCCTGGATCCCCAAGGTGCCAGCCCTGCTCCTGCACAGAGTGCTTCCTCAAGCC
AGTGCCCCCTCCTTCCCCCTAGCCAAGAACATGTGTGACCCCCACACCAGCTGTCCCAAATATACCACTT
GCTGCTTCATGAACAAGTCTGGCAAGTGGGGATGCTGCCCCCTGCCAAAGGCGGTGTGCTGTGCCAACGG
AGACCACTGCTGCCCCAGCGGCTACAGCTGTGATGTCCACAAGACCTCCTGCACTAAGGGCCCCCTGACC
ATTCCCTGGTACCGCAAAGAGAAGGCCCTGACTGAGGGAGCCATGTTGAAAGACGTAAAGTGTGACGACA
AGAGCAGCTGTGCCTCGGGGGCCACCTGCTGCAAGTTGCCCACGGGCGAATGGGGCTGCTGTCCCCTGGT

CAAGGCTGTTTGCTGTACAGACCATGAGCACTGCTGCCCCCAGGGCTACAGCTGCAACATGCAGACTGGG
ACCTGTGAGAAACTGGTAGAGGCTATAGTCCTCAGTACGGTGCCCCTGACAAAGGTAGCAGAGTCCCAGC
AGAGGGCAGCAGAGATGAGTATAGATGTGAAGTGTGACAGCACTGGAGAGTACAGCTGTCCCAAACTGGA
AACCTGCTGTAAGACCTCACCCACAGAATGGTCCTGCTGCCCTGCACCAAAGGCCATATGCTGCACGGAC
ACCAGGCACTGCTGCCCCATGGGATACACATGTGACCTGGAGCGGGGAGGCTGCTCCCAGCAGGCTGAGT
TGACCTGGGACATGTTCTACAGCCATGACAAAAAGAAAGACTTTGTTCCGTTTTGACTTTGAGCTAAATC
GGGAAGCACTGATCTGGACTCTGAGCCAATCAGGATGCAGCACTGATCTGATGTTCCTCACTCCAGCACA
CAACCTAACCTGCAGAACATTCCATAAGAGGAACAAACAACCTTATGGACACTGGACAAGGGACAGGAGA
TTTATTTTGTATGTTTTTTTTGTGTAAGTGATTTGATTTGGTTCAATTGGGGTAGAACAGGAAATAGAAA
TGGTTGGAAGTAGAGAATTCAGCATCTCCATCCAATGCCCATCCCAAAGTTTTATTTTTTATTATTTTAA
AGCAGCTAGCTTGTAGGTTTGTCTCAATGTCTTTACGCTTGTTACTACTGCCCCATATGCCATTTTCTCT
ATGCAAATGAGAAAGATGAATGTATTATACTCTCTTGGAACAAGAATTATCATTCTCCAGAATAAGCCTG
CTTTAAGATGTATCAAAAATGTATTTTATTCCAAACTCTTTCAAGAACACAGCTACATAGTACAAGAGAG
CTGATTTTTCATGTTTCTTTGCTGCAGGCACAACCTAGCATTAGTGAGCGTTAGTATATCGTTCTTTCTG
GATGCACTACAGAGCCATTGATCAGCCGACAGTGTTACTTTGATTGAACTTTTTGCTCGATTTAAAAAAA
AAAAAAAAAAATGTATGTATGTTTTGCCCTCTGGCCTGGGTTCATCACTTTCTGTGAACATCAAAATGGT
CCAGCCTCTGACAGTTTTCTGTCATTAAAATAACAAATACTGTCACTCAAACCCCTTAAGTCATAGCCAT
TTTTTCTTGATGTCATAATGCCCATTGTTGATAAACTTTTGATTTTCAGTATAGGAATTATTGGTAAACC
CCAGTTTAATGGTTTCTACATGCAATTTGTGGTGCTTGAACAGGTGATGGGGAAGTGAATCCAGTATGAT
TGGTTAGTACAATGTTAGTTGATAACAAAATTGATACTAAGCACTCTGTTCTACCAGCCGTTGTAATGCT
AACCAGTAATAATACTTTGAAAGTCAATGCAAACAATACCAGTGAGCACATAGCTCTCATCCCATTCCCG
ACTAATGTCCCAAATTTGTATCTGTACTCTATAGTTTTGTTTTGGATTTGTTTGTTTTTTTACATGAATG
TCATGTTAAACGAAGGACATGTTTCTTGGTTAATTATAGTTTCTTTATCATATATTTTATCTTTTGAAGG
GGAGTTCTGTGTCAGTGTATGCCACTGAGGTGAAATAAAATTGTTTTTGTTCTTCGGTAGGGAAAACCAT
TTTGTGTTGAGCTGCACTATAGTACAATAATAAACAATGGATGAGTCATGTTTTTGTCTGAAAAAAAAAA

Genomic data:

Note: Genomic structure and sequence from AGKD01009690.1 GI:354449353, AGKD01115372.1 GI:354343369, AGKD01153924.1 GI:354304604, and AGKD01010990.1 GI:354448053.

From NCBI: AGKD01009690.1 GI:354449353

Note: Contains the first 5 exons for the cDNA sequence in the forward direction at the end of the contig. The start codon is at the end of the second exon.

No exon sequence to show (or no genomic sequence entry).

Coding exon 1 Exon type: sn

ATAGGGGTGGTATGTCTGGCCCTCCTAGGCCTGACCTCAGCCCTGATTTGTCCCGATGGTGGGATGTGTG
ACGACGAGAACACCTGCTGCAAGACCCCCAGCGGTGGATATGGCTGCTGTCCATTACCAAAT

Coding exon 2 Exon type: c

GCTGAGTGCTGTTCCGACCACCTGCATTGCTGCTATGAGGGGACAGTGTGTGACTTAGTCCACTCCAAGT
GCCTCAATAAAACAGTCTCTCTGCCGTGGGTCAGAAGAGTCCCCGCACAACACATCATCAGCCCTCTG

Coding exon 3 Exon type: n

ATGGTGGAGGGAGTGAGGGCAGTAATTTGTCCCGACGGCGAGTCGGAGTGTCCGGACGACACCACCTGCT
GTCAACTCCCAGATGGCTCCTGGGGCTGCTGTCCGCTGGCCAAG

From NCBI: AGKD01115372.1 GI:354343369

Note: Contains coding exons 4 to 6.

Coding exon 4 Exon type: c

GCAGTGTGCTGTGAGGACAAGATGCACTGCTGTCCAGAGGGCACCAAGTGTGATCTAGCCCACTCCAAGT
GTGTGTCTCCCACCCTGGAGACCTTTGCCATGAGGGAAAAGGGGCCTGCAATGAAGAAGCAGACG

Coding exon 5 Exon type: n

GTGACCTGCCCAGGTGGTACGAGCAGATGCCCAGACAGAACCACCTGTTGCCTGCTGACCATTGGCGACT
ATGGCTGCTGCCCCTACCTGGAG

Coding exon 6 Exon type: c

GCTGTGTGCTGTACCGACAAACTCCACTGTTGCCCGGGCAATACAACCTGTGACCTGGAGCATGATATAT
GCACTTCCCCCAACACACAGACCCCACTGGCTAAGAAGACCCCTGCCGTCCCCAACGAT

From NCBI: AGKD01153924.1 GI:354304604

Note: Contains coding exons 7 to 13.
Coding exon 7 **Exon type: n**
GTGGATTGCCCAGACAAGGTGTCATCGTGTCCTGATGAGACCACTTGCTGCCTGCTGGGGGATAGGAGCT
ATGGCTGCTGCCCTATGCCAAGT

Coding exon 8 **Exon type: c**
GCTGTGTGTTGTCCAGACCACATCCACTGCTGTCCTGAGGGAACCACCTGTGACCTGGAACAGAGCACCT
GTGTGTCTGAACACGGCCAAACTTCCATGGCTGTCAAATTCCCCGCCACATTGACCACGACATCCCTACA
GAGCACA

Coding exon 9 **Exon type: n**
GTCAATGCTGTGCCCTGCAACGACTCTGTGGCCTGCGCTGATGGAACCACGTGCTGTAAATTACTAAATG
GAGAATGGGCCTGCTGCCCGCTACCCAAG

Coding exon 10 **Exon type: cn**
GCTGTGTGTTGTGATGACCACCTCCACTGCTGCCCCCACGGGACCATCTGTAACCTGGCAGAGAGTACGT
GTGATGACCCCTCTTCGGGCTCTGCCCTGGTACCCATGCTGGACAAGGTGCCCGCCTTTAGCTATGTGTC
GCAGGAGGAGCTGCTGCCCAACAGCATATGTGACAAATCCACGTTGTGCCCGGGCAAATCCACGTGCTGC
AAGACCACCACAGGAAACTGGGCATGCTGCCCCCTGCCCAAT

Coding exon 11 **Exon type: cn**
GCTGTGTGTTGCGATGACCACCTCCACTGCTGCCCCCACGGCACTGTGTGTAACCTGGAGGCCAGTAGCT
GCGATGACCCCTTAGGCTTCAACATGCCGTGGGTCACCAAGGTGCCAGCACTCACCACCCAGGCACAACT
GGCCACTGAGAAGTGTGACGAACAGACCATTTGCCCCGGGGGCACCACCTGCTGCAGACAGAACTCAGGA
CAGTGGGCATGCTGTCCTCTACCTCAT

Coding exon 12 **Exon type: cn**
GCGGTGTGCTGCGATGACCACGAGCACTGCTGCCCTAAAGGCTACACGTGTAATGTGGCCGAGCAGACCT
GTGACAAAGCTGGGACCCTCAGCTTGCCCTGGATCCCCAAGGTGCCAGCCCTGCTCCTGCACAGAGTGCT
TCCTCAAGCCAGTGCCCCCTCCTTCCCCCTAGCCAAGAACATGTGTGACCCCCACACCAGCTGTCCCAAA
TATACCACTTGCTGCTTCATGAACAAGTCTGGCAAGTGGGGATGCTGCCCCCTGCCAAAG

Coding exon 13 **Exon type: cn**
GCGGTGTGCTGTGCCAACGGAGACCACTGCTGCCCCAGCGGCTACAGCTGTGATGTCCACAAGACCTCCT
GCACTAAGGGCCCCCTGACCATTCCCTGGTACCGCAAAGAGAAGGCCCTGACTGAGGGAGCCATGTTGAA
AGACGTAAAGTGTGACGACAAGAGCAGCTGTGCCTCGGGGGCCACCTGCTGCAAGTTGCCCACGGGCGAA
TGGGGCTGCTGTCCCCTGGTCAAG

From NCBI: AGKD01010990.1 GI:354448053
Note: Contains coding exons 14 to 15 and comletes the salmoB progranulin gene.
Coding exon 14 **Exon type: cn**
GCTGTTTGCTGTACAGACCATGAGCACTGCTGCCCCCAGGGCTACAGCTGCAACATGCAGACTGGGACCT
GTGAGAAACTGGTAGAGGCTATAGTCCTCAGTACGGTGCCCCTGACAAAGGTAGCAGAGTCCCAGCAGAG
GGCAGCAGAGATGAGTATAGATGTGAAGTGTGACAGCACTGGAGAGTACAGCTGTCCCAAACTGGAAACC
TGCTGTAAGACCTCACCCACAGAATGGTCCTGCTGCCCTGCACCAAAG

Coding exon 15 **Exon type: c**
Note: up to the stop codon.
GCCATATGCTGCACGGACACCAGGCACTGCTGCCCCATGGGATACACATGTGACCTGGAGCGGGGAGGCT
GCTCCCAGCAGGCTGAGTTGACCTGGGACATGTTCTACAGCCATGACAAAAAGAAAGACTTTGTTCCGTT
TTGA

Genomic sequence note: A genomic sequence is not presented (see note 2 in header).

00025 **S. salar progranulin C** Short name: **S_salC or salmoC** Date entered: 2014-03

Species: ***Salmo salar (Atlantic salmon)***
Taxonomy (via NCBI): *Eukaryota; Metazoa; Chordata; Craniata; Vertebrata; Euteleostomi; Actinopterygii; Neopterygii; Teleostei; Euteleostei; Protacanthopterygii; Salmoniformes; Salmonidae; Salmoninae; Salmo.*
General Comment: There is a discrepancy in sequences through the second coding exon which is of the cn type. Two out of three EST sequences read through nicely, while the third has the same additional A found in three trace archive

wgs sequences which presumably contributed to the assembly of AGKD01126159.1. This extra A shifts the frame just before the second Cys codon for the N-half module, and would terminate translation about 23 codons further, despite there being good sequence from the c-n-c exons downstream. This could represent a mutant allele if it is not a sequencing artifact. We used the 2/3 majority EST cDNA sequence in the analysis.

Protein Sequence

```
KMWSIAALVLVLTGSASCYITCPGGKVCSDQSTCCLTKGGYACCPVPNAVCCSDMAHCCPSGFNCNAITQ
KCEKGDHPWSSVPMLNKVAAEEPSSPVSAPLQSDSSPVQSNAVESSMVGKVQCDNYYACPDGTTCCHHPT
GLWFCCPYSPGRCCLDGYHCCPYGYDCDPTYTKCVRYGNLRYPFAPRQAPSMIEAIKVSKPENKVHDQQV
SWTALVQAADSTPQAGVTHCDTKFYCPSATSCCKGPNGKWGCCPFPLAKCCADGLHCCEYGYTCDPNSYK
CRKWYSQIPSGLKDEANQD*
```

Transcript:

Note: The transcript is defined by an EST contig.
The cDNA data define the transcript well (below), so no predicted sequence is required.

cDNA data:

From: NCBI: EST contig from GI: (223564584, 29327546, 223564585, 24392971)
Note: cDNA sequence data:

```
TTGGCCAACTTTTTTGTACAAAGTTGTCCCCAACTGAGAACAATATCAAGGCTAAGATGTGGAGCATAGC
TGCATTGGTGTTAGTGCTGACAGGGTCTGCCTCTTGCTACATCACCTGCCCTGGTGGGAAGGTCTGCTCT
GATCAATCAACCTGTTGTTTGACTAAAGGAGGATACGCCTGCTGTCCAGTTCCCAATGCGGTGTGTTGCT
CTGACATGGCCCACTGTTGCCCGTCAGGCTTCAACTGCAATGCCATCACCCAGAAGTGTGAGAAAGGCGA
CCATCCATGGAGCAGTGTGCCCATGCTGAACAAGGTGGCTGCAGAGGAACCAAGCTCTCCTGTCTCTGCT
CCGCTCCAGTCTGACAGCAGCCCTGTCCAGAGCAATGCAGTGGAGAGCTCCATGGTCGGCAAGGTGCAAT
GTGACAACTATTATGCTTGTCCCGATGGCACCACCTGCTGCCACCACCCCACAGGCCTGTGGTTCTGCTG
TCCGTACTCTCCTGGTAGGTGCTGTCTAGATGGGTACCACTGCTGTCCCTACGGCTATGACTGTGACCCC
ACATACACTAAGTGTGTGAGGTACGGCAACCTGAGGTACCCTTTCGCTCCGAGGCAGGCCCCTTCAATGA
TCGAAGCCATCAAGGTCTCCAAACCGGAAAACAAGGTCCACGATCAGCAGGTCTCATGGACAGCTCTAGT
TCAGGCCGCTGACAGCACGCCACAGGCTGGAGTCACTCACTGTGACACTAAATTCTATTGTCCTTCTGCA
ACCAGCTGCTGCAAGGGACCTAATGGCAAATGGGGATGTTGCCCATTCCCACTGGCGAAGTGTTGTGCGG
ATGGCCTGCATTGCTGTGAATATGGATACACCTGTGACCCAAACTCATACAAGTGCAGGAAATGGTACTC
TCAGATTCCTTCAGGTCTGAAGGATGAGGCTAATCAGGACTGACACTATTTCAAATTGTTTATGATCTTG
TCACTGTACTGACATTGGATTGTACCTTTTGTGAATGCATTGTAATTTAACCTTGCACTGATGAATACAA
GAATGTTAATCAGTCACCCAATCATT
```

Genomic data:

Note: Genomic sequence is mostly in AGKD01126159.1 GI:354332369 (see general comment on sequene discrepancy), and ends in AGKD01460049.1 GI:353998421
From NCBI: AGKD01126159.1 GI:354332369
Note: Contains all but the last coding exon
Coding exon 1 **Exon type: sn**
Note: from the start codon (which is at the beginning of this exon).

```
ATGTGGAGCATAGCTGCATTGGTGTTAGTGCTGACAGGGTCTGCCTCTTGCTACATCACCTGCCCTGGTG
GGAAGGTCTGCTCTGATCAATCAACCTGTTGTTTGACTAAAGGAGGATACGCCTGCTGTCCAGTTCCCAA
T
```

Coding exon 2 **Exon type: cn**
Note: Important: The discrepant additional A has been removed to force this exon to conform with the majority cDNA sequence and a well-formed cn exon.

```
GCGGTGTGTTGCTCTGACATGGCCCACTGTTGCCCGTCAGGCTTCAACTGCAATGCCATCACCCAGAAGT
GTGAGAAAGGCGACCATCCATGGAGCAGTGTGCCCATGCTGAACAAGGTGGCTGCAGAGGAACCAAGCTC
TCCTGTCTCTGCTCCGCTCCAGTCTGACAGCAGCCCTGTCCAGAGCAATGCAGTGGAGAGCTCCATGGTC
GGCAAGGTGCAATGTGACAACTATTATGCTTGTCCCGATGGCACCACCTGCTGCCACCACCCCACAGGCC
TGTGGTTCTGCTGTCCGTACTCTCCT
```

Coding exon 3 **Exon type: c**

```
GGTAGGTGCTGTCTAGATGGGTACCACTGCTGTCCCTACGGCTATGACTGTGACCCCACATACACTAAGT
GTGTGAGGTACGGCAACCTGAGGTACCCTTTCGCTCCGAGGCAGGCCCCTTCAATGATCGAAGCCATCAA
```

GGTCTCCAAACCGGAAAACAAGGTCCACGATCAG

Coding exon 4 **Exon type:** **n**
CAGGTCTCATGGACAGCTCTAGTTCAGGCCGCTGACAGCCCGCCACAGGCTGGAGTCACTCACTGTGACA
CTAAATTCTATTGTCCTTCTGCAACCAGCTGCTGCAAGGGACCTAATGGCAAATGGGGATGTTGCCCATT
CCCACTG

From NCBI: AGKD01460049.1 GI:353998421
Note: Contains only the last exon and does not overlap with AGKD01126159.1.
Coding exon 5 **Exon type:** **c**
Note: up to the stop codon.
GCGAAGTGTTGTGCGGATGGCCTGCATTGCTGTGAATATGGATACACCTGTGACCCAAACTCATACAAGT
GCAGGAAATGGTACTCTCAGATTCCTTCAGGTCTGAAGGATGAGGCTAATCAGGACTGA

Genomic sequence note: A genomic sequence is not presented (see note 2 in header).

00007 **T. rubripes progranulin B** Short name: **T_rubB or fuguB** Date entered: 2014-03

Species: ***Takifugu rubripes (Fugu rubripes) (Japanese pufferfish)***
Taxonomy (via NCBI): *Eukaryota; Metazoa; Chordata; Craniata; Vertebrata; Euteleostomi; Actinopterygii; Neopterygii; Teleostei; Euteleostei; Neoteleostei; Acanthomorpha; Acanthopterygii; Percomorpha; Tetraodontiformes; Tetradontoidea; Tetraodontidae; Takifugu.*
General Comment:

Protein Sequence

MTALQTSILLLALLNLSAALVCPDGGTCEDRNTCCRNSAGGYSCCPLPHAECCSDHLHCCYQGTLCDLEH
HKCVNKTVSLPWLSRLPAAPRVQAGLTGVICPDSKTMCPDGATCCQLPSGSMGCCLFSNAVCCEDKLHCC
PEGTRCDLARSKCVSPSQQWFPMMHKVPATTRAEHPEASIICPDGKSRCQLGHTCCQLASGAYGCCPLQQ
AVCCSDHERCCPAGTRCDLEHDACVSGATPVPMLRIAAVPGEGATPVVPIKIDNNKCDESTTCPGDSTCC
RTLEGGWACCPLAQAVCCDDHVHCCPHDTICNLETQTCDGQSGGRPPLRWVEKVPASTSAVQCDEQTSCP
GGSTCCKMASGQWACCPLPEAVCCSDGEHCCPKGYRCNLAVQTCDVAGGDTRPWLRKVPARQEEPSGAAS
GPARPAGVMCDDRTSCPRDTSCCFMQETRRWGCCPVPNAVCCEDGDHCCPRGHRCDPHRRSCSKGPLVTP
WFTKLSAATRPGAVTDVPCDDRSSCPAGTTCCKLKSGGWGCCPLVKAVCCNDHEHCCPQGYSCNTETGTC
EKKDHGAPVFAVPQRRVLESRSRGAQGVLSCGGTGEFHCPKEDTCCPTSATEWACCPSPRAVCCSDQKHC
CPAGFSCDPSGGCVQDLSSWDAWFDRSARGGL*

Transcript:

Note: Incomplete direct cDNA sequence data. Limited est sequence data available at time of entry.

Predicted sequence:

Note:
Derivation: NCBI XM_003961109.1 GI:410895340 (also JGI: Takru4|622326|estExt_GW.C_1300089)
tccgccttggtttctcctgcgaaatttgcctttaggagcaacgttaatgtgacagctggacaaactgcgc
cggacaggttcggagtatgtacacttgtgccttttgttgctaatacctgaagttcttctactccaacaag
acagctgctaataatgacggctctgcagacgtcgatcctgcttctggctctcctcaacctgagcgcggcg
ctggtttgtcctgatggaggcacgtgtgaggacaggaacacctgctgcaggaactccgcaggtggataca
gctgctgcccgctaccgcatgctgaatgttgctccgaccatctgcactgctgctaccaggggacgctttg
tgacctggaacaccacaaatgtgtcaacaaaacggtttctttgccctggttgagccgacttcctgccgcc
cctcgggtccaggctgggctgactggggtcatctgtcctgattctaagaccatgtgtccggatggcgcca
cttgctgccagcttcccagtggttccatgggctgttgcctgttcagcaacgccgtgtgctgcgaggacaa
gctccactgttgcccggaaggaacgaggtgtgatctcgctcgctccaagtgcgtttcgccatcgcagcag
tggttccccatgatgcacaaagttccggccaccacgagagcagagcatccagaggcttctataatatgtc
ctgatggaaaaagccgctgccaactcggccacacatgctgccagctagccagcggcgcctacggctgctg
cccgctccaacaggctgtgtgctgcagcgatcacgagcgctgctgccctgcaggcacgcgctgcgacctg
gagcacgacgcctgtgtgtccggcgccacgccagtcccaatgctgaggatcgccgccgtccccggcgaag
gcgcgacgcctgttgttccaatcaagatcgacaacaacaagtgcgacgagtccacgacgtgtcccgggga
ctccacctgctgtaggacgctagaaggcggctgggcctgttgcccgttggcgcaggctgtgtgctgtgac
gatcacgtccactgctgccctcacgacaccatctgtaacctggagactcaaacctgcgacggccagtcgg
ggggtcgtcctcccctccgctgggttgaaaaggtgcctgcgtccacctcggcggtgcagtgcgacgagca

aacgtcgtgtccgggaggctccacctgctgcaaaatggcgtcgggtcagtgggcctgctgcccgctgcct
gaggccgtgtgctgcagcgatggcgagcactgctgtcctaaaggctaccgatgtaacctggcggtccaga
cctgtgacgtggccggcggagacactcggccgtggctgcggaaggttccggcccggcaggaggagcccag
cggggcggcgtccggtccggctcggccagccggggtcatgtgtgacgaccggaccagctgccctcgagac
accagctgctgcttcatgcaggagacgcgccgatggggctgctgccccgtcccgaacgcggtttgttgtg
aggacggggaccactgctgccccaggggggcaccgctgtgacccccaccgccgttcctgctcaaagggccc
cctggttacgccctggttcaccaaactgagcgccgcgactcggccgggcgccgttacggacgtcccgtgt
gacgaccggagcagctgtccggcaggaaccacctgctgcaagctgaagagcggagggtggggctgctgcc
cgctggtcaaggcggtgtgctgcaacgaccacgagcactgctgtcctcagggctacagctgcaacacgga
gacgggcacgtgcgagaagaaggaccacggcgcgcccgtctttgctgtcccccagaggagggtgctagag
tccaggtccagaggagcccaaggcgtcctgtcatgtggaggcacgggggaattccactgtcccaaggagg
acacttgctgtccaacatcagccacagagtgggcctgctgcccctcaccgagggccgtgtgctgctcaga
ccagaagcactgctgtcccgctggattctcctgtgacccgtctggaggctgcgtccaggacctgtcctcc
tgggacgcctggtttgataggtccgcccgaggtggactttaa

cDNA data:

From: NCBI est: GI: (25134870, 26775899, 21879548, 22020914)

Note: A cDNA contig encoding the signal peptide and first 4 modules.

TCCGCCTTGGTTTCTCCTGCGAAATTTGCCTTTAGGAGCAACGTTAATGTGACAGCTGGACAAACTGCGC
CGGACAGGTTCGGAGTATGTACACTTGTGCCTTTTGTTGCTAATACCTGAAGTTCTTCTACTCCAACAAG
ACAGCTGCTAATAATGACGGCTCTGCAGACGTCGATCCTGCTTCTGGCTCTCCTCAACCTGAGCGCGGCG
CTGGTTTGTCCTGATGGAGGCACGTGTGAGGACAGGAACACCTGCTGCAGGAACTCCGCAGGTGGATACA
GCTGCTGCCCGCTACCGCATGCTGAATGTTGCTCCGACCATCTGCACTGCTGCTACCAGGGGACGCTCTG
TGACCTGGAACACCACAAATGTGTCAACAAAACGCATTCTGTGCCCTGGTTGAGCCGACTTCCTGCCGCC
CCTCGGGTCCAGGCTGGGCTGACTGGGGTCATCTGTCCTGATTCTAAGACCATGTGTCCGGATGGCGCCA
CTTGCTGCCAGCTTCCCAGTGGTTCCATGGGCTGTTGCCTGTTCAGCAACGCCGTGTGCTGCGAGGACAA
GCTCCACTGTTGCCCGGAAGGAACGAGGTGTGATCTCGCTCGCTCCAAGTGCGTTTCGCCATCGCAGCAG
TGGTTCCCCATGATGCACAAAGTTCCGGCCACCACGAGAGCAGAGCATCCAGAGGCTTCTATAATATGTC
CTGATGGAAAAAGCCGCTGCCAACTCGGCCACACATGCTGCCAGCTAGCCAGCGGCGCCTACGGCTGCTG
CCCGCTCCAACAGGCTGTGTGCTGCAGCGATCACGAGCGCTGCTGCCCTGCAGGCACGCGCTGCGACCTG
GAGCACGACGCCTGCGTGTCCGGCGCCACGCCAGTCCCAATGCTGAGGATCGCCGCCGTCCCCGGCGAAG
GCGCGACGCCTGTTGTCCCAATCAAGATCGACAACAACAAGTGCGACGAGTCCACGACGTGTCCCGGGGA
CTCCACCTGCTGTAGGACGCTAGAAGGAGGCTGGGCCTGTTGCCCGTTGGCGCAGGCTGTGTGCTGTGAC
GATCACGTCCACTGCTGCCCTCACGACACCATCTGTAACCTGGAGACTCAAACCTGCG

From: NCBI est: GI: 22020841

Note: This cDNA fragment includes module 5.

AACCTGGAGACTCAAACCTGCGACGGCCAGTCGGGGGGGCGGCCTCCCCTCCGCTGGGTTGAAAAGGCGC
CTGCAGCCACCTCGGCGGTGCAGTGCGACGAGCAAACGTCGTGTCCGGGAGTCTCCACCTGCTGCAAAAT
GGCGTCGGGTCAGTGGGCCTGCTGCCCGCTGCCTGAGGCCGTGTGCTGCAGCGATGGCGAGCACTGCTGC
TCTAAAGGCTACCGATGTAACCTGGCGGGCCAGACCCTGACGGGGCCG

From: NCBI est: GI: 24550509

Note: Reverse complement of the cDNA fragment. It encodes most of the last module and has 3'ut

TTTTTTTTCAATCTCCCAAAGGCAGATTTGCCGATTTTATTACTGGATGAAAACTGCAAATAAAAAAATA
AACCTTTAAAAACACAAACCAACCCAATGCTCAGAAAAAAAAAGAAAAACTTTAAAAATTACACAGATAC
TTTGCCATCAATGTTTTATAACAAAAGACAATTAGAACCACATTGAAAGAGTTCTGGAATGGTTTTCCCA
GGAGAAAATATTCAGAACTCTTCAACTTCCTGTTCACCAATACAGCACGTCTATATGGAGTGCAGAAGCG
TCCCAGAGCGAAGGAAAGAGGGAGTTTAGAGCCTTCAACCATCTGGTTTTAAAGTCCACCTCGGGCGGAC
CTATCAAACCAGGCGTCCCAGGAGGACAGGTCCTGGACGCAGCCTCCAGACGGGTCACAGGAGAATCCAG
CGGGACAGCAGTGCTTCTGGTCTGAGCAGCACACGGCCCTCGGTGAGGGGCAGCAGGCCCACTCTGTGGC
TGATGTTGGACAGCAAGTGTCCTCCTTGGGACA

Genomic data:

Note: Genomic data for coding exon structure is in gi|409211590:1774463-1780861 Takifugu rubripes chromosome 1

From NCBI: GI: 409211590

Note: Progranulin coding sequences contained within the range 1774463-1780861.

No exon sequence to show (or no genomic sequence entry).

Coding exon 1 **Exon type:** sn

GCTCTGCAGACGTCGATCCTGCTTCTGGCTCTCCTCAACCTGAGCGCGGCGCTGGTTTGTCCTGATGGAG
GCACGTGTGAGGACAGGAACACCTGCTGCAGGAACTCCGCAGGTGGATACAGCTGCTGCCCGCTACCGCA
T

Coding exon 2 **Exon type: cn**
GCTGAATGTTGCTCCGACCATCTGCACTGCTGCTACCAGGGGACGCTTTGTGACCTGGAACACCACAAAT
GTGTCAACAAAACGGTTTCTTTGCCCTGGTTGAGCCGACTTCCTGCCGCCCCTCGGGTCCAGGCTGGGCT
GACTGGGGTCATCTGTCCTGATTCTAAGACCATGTGTCCGGATGGCGCCACTTGCTGCCAGCTTCCCAGT
GGTTCCATGGGCTGTTGCCTGTTCAGCAAC

Coding exon 3 **Exon type: c**
GCCGTGTGCTGCGAGGACAAGCTCCACTGTTGCCCGGAAGGAACGAGGTGTGATCTCGCTCGCTCCAAGT
GCGTTTCGCCATCGCAGCAGTGGTTCCCCATGATGCACAAAGTTCCGGCCACCACGAGAGCAGAGCATCC
AG

Coding exon 4 **Exon type: n**
AGGCTTCTATAATATGTCCTGATGGAAAAAGCCGCTGCCAACTCGGCCACACATGCTGCCAGCTAGCCAG
CGGCGCCTACGGCTGCTGCCCGCTCCAACAG

Coding exon 5 **Exon type: c**
GCTGTGTGCTGCAGCGATCACGAGCGCTGCTGCCCTGCAGGCACGCGCTGCGACCTGGAGCACGACGCCT
GTGTGTCCGGCGCCACGCCAGTCCCAATGCTGAGGATCGCCGCCGTCCCCGGCGAAG

Coding exon 6 **Exon type: n**
GCGCGACGCCTGTTGTTCCAATCAAGATCGACAACAACAAGTGCGACGAGTCCACGACGTGTCCCGGGGA
CTCCACCTGCTGTAGGACGCTAGAAGGCGGCTGGGCCTGTTGCCCGTTGGCGCAG

Coding exon 7 **Exon type: cn**
GCTGTGTGCTGTGACGATCACGTCCACTGCTGCCCTCACGACACCATCTGTAACCTGGAGACTCAAACCT
GCGACGGCCAGTCGGGGGGTCGTCCTCCCCTCCGCTGGGTTGAAAAGGTGCCTGCGTCCACCTCGGCGGT
GCAGTGCGACGAGCAAACGTCGTGTCCGGGAGGCTCCACCTGCTGCAAAATGGCGTCGGGTCAGTGGGCC
TGCTGCCCGCTGCCTGAG

Coding exon 8 **Exon type: cn**
GCCGTGTGCTGCAGCGATGGCGAGCACTGCTGTCCTAAAGGCTACCGATGTAACCTGGCGGTCCAGACCT
GTGACGTGGCCGGCGGAGACACTCGGCCGTGGCTGCGGAAGGTTCCGGCCCGGCAGGAGGAGCCCAGCGG
GGCGGCGTCCGGTCCGGCTCGGCCAGCCGGGGTCATGTGTGACGACCGGACCAGCTGCCCTCGAGACACC
AGCTGCTGCTTCATGCAGGAGACGCGCCGATGGGGCTGCTGCCCCGTCCCGAAC

Coding exon 9 **Exon type: cn**
GCGGTTTGTTGTGAGGACGGGGACCACTGCTGCCCCAGGGGGCACCGCTGTGACCCCCACCGCCGTTCCT
GCTCAAAGGGCCCCCTGGTTACGCCCTGGTTCACCAAACTGAGCGCCGCGACTCGGCCGGGCGCCGTTAC
GGACGTCCCGTGTGACGACCGGAGCAGCTGTCCGGCAGGAACCACCTGCTGCAAGCTGAAGAGCGGAGGG
TGGGGCTGCTGCCCGCTGGTCAAG

Coding exon 10 **Exon type: cn**
GCGGTGTGCTGCAACGACCACGAGCACTGCTGTCCTCAGGGCTACAGCTGCAACACGGAGACGGGCACGT
GCGAGAAGAAGGACCACGGCGCGCCCGTCTTTGCTGTCCCCCAGAGGAGGGTGCTAGAGTCCAGGTCCAG
AGGAGCCCAAGGCGTCCTGTCATGTGGAGGCACGGGGGAATTCCACTGTCCCAAGGAGGACACTTGCTGT
CCAACATCAGCCACAGAGTGGGCCTGCTGCCCCTCACCGAGG

Coding exon 11 **Exon type: c**
GCCGTGTGCTGCTCAGACCAGAAGCACTGCTGTCCCGCTGGATTCTCCTGTGACCCGTCTGGAGGCTGCG
TCCAGGACCTGTCCTCCTGGGACGCCTGGTTTGATAGGTCCGCCCGAGGTGGACTTTAA

Genomic sequence note: A genomic sequence is not presented (see note 2 in header).

00008 **T. rubripes progranulin C** Short name: **fuguC** Date entered: 2014-03

Species: ***Takifugu rubripes (Fugu rubripes) (Japanese pufferfish)***
Taxonomy (via NCBI): *Eukaryota; Metazoa; Chordata; Craniata; Vertebrata; Euteleostomi; Actinopterygii; Neopterygii;*

Teleostei; Euteleostei; Neoteleostei; Acanthomorpha; Acanthopterygii; Percomorpha; Tetraodontiformes; Tetradontoidea; Tetraodontidae; Takifugu.

General Comment:

Protein Sequence

```
MSKLTLCLVAGAILLGVVACVKCPDGHQCTDKETCCQGTKGYNCCPYPDAVCCSDKTHCCPSGYTCNLAE
QMCEKQNLPWFRIPMVMEEAEKSSASVLPVYFSRGVEDSRVPAEEKSSVLCDNHYRCPDGTTCCRNLAGG
WFCCPYSPGSCCQDGYHCCPMGYHCDPTYRYCLQQGLRYPFTARKQPPVVPAALIVASEERSGLLERPMM
PLKKLSNDMQDAGGVQCDSTFVCPADMTCCKGPQGQWSCCPFRLGQCCADGQHCCEYGYTCSSSSVSCKK
SFSQ*
```

Transcript:

Note: Incomplete direct cDNA sequence data. Limited est sequence data available at time of entry.

Predicted sequence:

Note:

Derivation: NCBI: XM_003969392.1 GI:410911925 (also JGI: Takru4|553464|e_gw2.37.302.1)

```
ATGTCGAAGTTAACTCTGTGTCTGGTGGCCGGTGCGATCCTGCTGGGGGTTGTAGCCTGTGTCAAATGCC
CCGATGGGCATCAATGCACAGACAAAGAGACTTGCTGTCAGGGGACGAAAGGCTACAACTGCTGTCCATA
TCCAGATGCCGTGTGTTGCTCTGACAAGACCCACTGCTGCCCTTCAGGGTATACCTGTAACCTTGCCGAA
CAGATGTGTGAGAAACAGAACCTGCCGTGGTTTAGAATCCCCATGGTGATGGAAGAGGCGGAGAAATCGA
GTGCCTCCGTTCTACCCGTGTATTTCTCCCGAGGGGTTGAGGACAGCCGCGTCCCAGCTGAGGAGAAGAG
CTCGGTCCTCTGTGACAACCATTACCGATGTCCTGACGGCACCACCTGCTGCAGGAACCTGGCTGGTGGC
TGGTTCTGCTGTCCATATTCCCCTGGCAGCTGCTGTCAGGATGGCTACCACTGTTGTCCCATGGGCTACC
ACTGTGACCCTACTTACCGGTACTGTTTGCAGCAGGGCCTGAGGTATCCTTTCACAGCCAGGAAGCAACC
ACCTGTAGTCCCTGCTGCCCTCATTGTCGCCTCAGAGGAAAGAAGTGGACTTCTGGAGAGACCAATGATG
CCTCTCAAAAAACTCAGCAACGACATGCAAGACGCTGGAGGTGTTCAGTGTGATTCGACCTTTGTCTGCC
CAGCGGACATGACGTGCTGCAAAGGTCCCCAGGGCCAGTGGAGCTGTTGTCCATTTCGACTGGGCCAGTG
TTGCGCAGATGGTCAGCACTGCTGTGAATATGGATACACCTGCAGCAGCTCCTCCGTGTCCTGCAAAAAG
TCCTTCTCACAGTAG
```

cDNA data:

From: NCBI est: GI: 22020744

Note: Short poor cDNA sequence encoding part of signal and first module.

```
GTGTCTGGTGGCCGGTGCGATCCTGCTGGGGGTTGTAGCCTGTGTCAGAGGCCCCGATGGGCATCAATGC
ACAGACAAAGAGACTTGCTGTCAGGGGACGAAAGGCTACAACTGCTGTCCATATCCAGATGCCGTGTGTT
GCTCTGACAAGACCCACTGCTGCCCTTCAGGTATACCTGTAACCTTGCCGAACAGATGTGTGAGAAACAG
AACCTGCCGTGGTTTAGAATCCCCATGGTGATGGAAGAGGCGGAGAAATCGAGTGCCTCCGTTCTACCCG
TGTATTTCTCCCGAGGGGTTGAGGACAGCCGCCGCCCAGCTG
```

Genomic data:

Note: Genomic data for coding exons is in gi|409211579:9037901-9039687 Takifugu rubripes chromosome 12

From NCBI: GI: 409211579

Note: Progranulin coding sequences contained within the range 9037901-9039687

Coding exon 1 **Exon type: sn**

Note: from start codon

```
ATGTCGAAGTTAACTCTGTGTCTGGTGGCCGGTGCGATCCTGCTGGGGGTTGTAGCCTGTGTCAAATGCC
CCGATGGGCATCAATGCACAGACAAAGAGACTTGCTGTCAGGGGACGAAAGGCTACAACTGCTGTCCATA
TCCAGAT
```

Coding exon 2 **Exon type: cn**

```
GCCGTGTGTTGCTCTGACAAGACCCACTGCTGCCCTTCAGGGTATACCTGTAACCTTGCCGAACAGATGT
GTGAGAAACAGAACCTGCCGTGGTTTAGAATCCCCATGGTGATGGAAGAGGCGGAGAAATCGAGTGCCTC
CGTTCTACCCGTGTATTTCTCCCGAGGGGTTGAGGACAGCCGCGTCCCAGCTGAGGAGAAGAGCTCGGTC
CTCTGTGACAACCATTACCGATGTCCTGACGGCACCACCTGCTGCAGGAACCTGGCTGGTGGCTGGTTCT
GCTGTCCATATTCCCCT
```

Coding exon 3 **Exon type: c**

```
GGCAGCTGCTGTCAGGATGGCTACCACTGTTGTCCCATGGGCTACCACTGTGACCCTACTTACCGGTACT
```

GTTTGCAGCAGGGCCTGAGGTATCCTTTCACAGCCAGGAAGCAACCACCTGTAGTCCCTGCTGCCCTCAT
TGTCGCCTCAGAGGAAAGAAGTGGACTTCTGGAG

Coding exon 4 **Exon type: n**
AGACCAATGATGCCTCTCAAAAAACTCAGCAACGACATGCAAGACGCTGGAGGTGTTCAGTGTGATTCGA
CCTTTGTCTGCCCAGCGGACATGACGTGCTGCAAAGGTCCCCAGGGCCAGTGGAGCTGTTGTCCATTTCG
ACTG

Coding exon 5 **Exon type: c**
Note: up to stop codon
GGCCAGTGTTGCGCAGATGGTCAGCACTGCTGTGAATATGGATACACCTGCAGCAGCTCCTCCGTGTCCT
GCAAAAAGTCCTTCTCACAGTAG

Genomic sequence note: A genomic sequence is not presented (see note 2 in header).

00009 **T. rubripes progranulin D** Short name: **T_rubD or fuguD** Date entered: 2014-03

Species: ***Takifugu rubripes (Fugu rubripes) (Japanese pufferfish)***
Taxonomy (via NCBI): *Eukaryota; Metazoa; Chordata; Craniata; Vertebrata; Euteleostomi; Actinopterygii; Neopterygii; Teleostei; Euteleostei; Neoteleostei; Acanthomorpha; Acanthopterygii; Percomorpha; Tetraodontiformes; Tetradontoidea; Tetraodontidae; Takifugu.*
General Comment: This short unusual progranulin is apparently most evolutionarily connected with the fish progranulin A form.

Protein Sequence
MLSSVLFSGVLLVLVGADQCPGGRCDVCSEQEVLCPDGKRCCPEGHLCSSDGRSCIKTAVVPCSDTEACP
DNYTCCKDGSDKWTCCPLPQAVCCPDRNYCCPAQYRCDEQSTSCIKGDSVMSWFPKLPAALRMDAGLQVA
LRDVRCDEQTSCKDGQTCCRTSPTTWGCCPSPQAVCCSDMKHCCPTGYTCSEGGQCSLKTRRLRWFNWGD
EKLI*

Transcript:
Note: Incomplete direct cDNA sequence data. Limited est sequence data available at time of entry.

Predicted sequence:
Note:
Derivation: NCBI: XM_003965021.1 GI:410903176 (also JGI: Takru4|623961|estExt_GW.C_3070012)
ATGCTGTCCAGCGTGTTGTTCTCAGGGGTTCTGCTGGTGCTGGTCGGGGCAGATCAGTGCCCAGGAGGAA
GGTGTGATGTCTGCAGCGAGCAGGAAGTTCTCTGCCCTGATGGAAAACGCTGCTGTCCTGAAGGTCACCT
GTGCAGCTCGGACGGACGCTCCTGCATTAAAACAGCTGTCGTTCCATGCAGTGACACTGAGGCGTGTCCA
GATAATTACACCTGTTGTAAGGACGGAAGTGACAAATGGACATGTTGTCCTCTACCGCAGGCTGTGTGCT
GCCCAGATAGGAATTACTGCTGCCCCGCCCAGTACAGGTGTGACGAGCAGAGCACCTCCTGCATCAAGGG
CGATTCAGTGATGTCCTGGTTCCCAAAGCTTCCTGCTGCCCTTCGCATGGACGCCGGCCTCCAGGTGGCG
CTCCGAGACGTCCGGTGTGACGAGCAGACCAGCTGCAAAGACGGCCAGACCTGCTGCAGAACATCACCCA
CCACCTGGGGCTGCTGCCCCTCTCCACAAGCGGTGTGCTGCAGTGACATGAAGCACTGCTGTCCCACCGG
CTACACCTGCTCGGAAGGAGGACAATGTTCCCTGAAGACCCGCCGCCTTCGCTGGTTCAACTGGGGCGAC
GAGAAGCTGATCTGA

cDNA data:
From: NCBI est: GI: 25133181
Note: This cDNA fragment covers all but the last 9 codons and stop codon.
CACACCAGTCCGGCCCCCTATAAGATGCTGTCCAGCGTGTTGTTCTCAGGGGTTCTGCCTGTGCTGGTCG
GGGCAGATCAGTGCCCAGGAGGAAGGTGTGATGTCTGCAGCGAGCAGGAAGTTCTCTGCCCTGCTGGAAA
ACGCTGCTGTCCTGAAGGTCACCTGTGCAGCTCGGACGGACGCTCCTGCATTAAAACAGCTGTCGTTCCA
TGCAGTGACACTGAGGTGTGTCCAGATAATTACACCTGTTGTAAGGACGGAAGTGACAAATGGACATGTT
GTCCTCTACCGCAGGCTGTGTGCTGCCCAGATAGGAATCACTGCTGCCCCGCCCAGTACAGGTGTGACGA
GCAGAGCACCTCCTGCATCAAGGGCGATTCAGTGATGTCCTGGTTCCCAAAGCTTCCTGCTGCCCTTCGC
ATGGACGCCGGCCTCCAGGTGGCGCTCCGAGACGTCCGGTGTGACGAGCAGACCAGCTGCAAAGACGGCC
AGACCTGCTGCAGAACATCACCCACCACCTGGGGCTGCTGCCCCTCTCCACAAGCGGTGTGCTGCAGTGA
CATGAAGCACTGCTGTCCCACCGGCTACACCTGCTCGGAAGGAGGACAATGTTCCCTGAAGACCCGCCGC

CTTCGCTGGTT

Genomic data:

Note: Genomic data for coding exons is in gil409211586:11923025-11925211 Takifugu rubripes chromosome 5

From NCBI: GI: 409211586

Note: Progranulin coding sequences contained within the range 11923025-11925211

Coding exon 1 **Exon type: sx**

Note: From start codon. Where x encodes 14 aa containing 3 Cys.

ATGCTGTCCAGCGTGTTGTTCTCAGGGGTTCTGCTGGTGCTGGTCGGGGCAGATCAGTGCCCAGGAGGAA
GGTGTGATGTCTGCAGCGAGCAG

Coding exon 2 **Exon type: c***

Note: Encodes the C-half of a 10 Cys module.

GAAGTTCTCTGCCCTGATGGAAAACGCTGCTGTCCTGAAGGTCACCTGTGCAGCTCGGACGGACGCTCCT
GCATTAAAACAG

Coding exon 3 **Exon type: n**

CTGTCGTTCCATGCAGTGACACTGAGGCGTGTCCAGATAATTACACCTGTTGTAAGGACGGAAGTGACAA
ATGGACATGTTGTCCTCTACCGCAG

Coding exon 4 **Exon type: cn**

GCTGTGTGCTGCCCAGATAGGAATTACTGCTGCCCCGCCCAGTACAGGTGTGACGAGCAGAGCACCTCCT
GCATCAAGGGCGATTCAGTGATGTCCTGGTTCCCAAAGCTTCCTGCTGCCCTTCGCATGGACGCCGGCCT
CCAGGTGGCGCTCCGAGACGTCCGGTGTGACGAGCAGACCAGCTGCAAAGACGGCCAGACCTGCTGCAGA
ACATCACCCACCACCTGGGGCTGCTGCCCCTCTCCACAA

Coding exon 5 **Exon type: c**

Note: up to stop codon

GCGGTGTGCTGCAGTGACATGAAGCACTGCTGTCCCACCGGCTACACCTGCTCGGAAGGAGGACAATGTT
CCCTGAAGACCCGCCGCCTTCGCTGGTTCAACTGGGGCGACGAGAAGCTGATCTGA

Genomic sequence note: A genomic sequence is not presented (see note 2 in header).

00006 **X. tropicalis progranulin** Short name: **X_tro or frogXt** Date entered: 2014-03

Species: ***Xenopus tropicalis (western clawed frog)***

Taxonomy (via NCBI): *Eukaryota; Metazoa; Chordata; Craniata; Vertebrata; Euteleostomi; Amphibia; Batrachia; Anura; Mesobatrachia; Pipoidea; Pipidae; Xenopodinae; Xenopus; Silurana.*

General Comment: Good cDNA and genomic data. But there are discrepancies within the middle repetitive section. Additional genomic sequence is provided here which is consistent with the EST contig and shows evidence of this middle repeat.

Protein Sequence

MGPPWFLLLVVSTVSATLCPDGSTCGEKSLCCELPGKKGYGCCPAAEVVSRSLPMILSQTSCSGCPDEYS
CVNTPEGGTACCPLSEGKSCQDGHHCCSVGSYCSDDGHYCIPASNQSAVVCPDGRSECPTLTTCCMMSDM
SSWGCCPMPQAVCCDDHMHCCPHNSECDVQQGRCISNQDHIPWMSKLPARVKSEGLKLVGLGDEERRVPC
LDGTFCPDGSTCCEQVDHTYGCCSILSAVCCSDHLHCCPGGTTCDLVHKKCVSQTGEGPLLPQMPAIREE
SANQVPCDATTSCPDKNTCCHLSSEKWGCCPYAQAVCCDDHIHCCPSGFTCSGGSCVLAEHSIPWMRKTL
AQGLKTTRVQCDDTASCPEKETCCRLVSGKWGCCPVVKAVCCNDHLHCCPEGYTCSQGECSKMEHSIPWF
TKTPALTHEARDVECDDMYSCPDGQTCCRLASGDWGCCPIAQAVCCDDHEHCCPPGYTCSGGSCQKGELS
IPWFLKTPALKQKARDVQCDDMYSCADGQTCCRLASGDWGCCPIAQAVCCDDHEHCCPPGYTCSGGSCQQ
GVLSIPWFLKTPALKQNARDVQCDDMYSCADGQTCCRLASGDWGCCPIAQAVCCDDHEHCCPPGYTCSGG
QCQKGEHSIPLFSKTPALKQKARDVQCDEIYSCPDGQTCCRLASGDWGCCPIARAVCCDDHEHCCPHGYI
CSGGSCQQGELSIPWFLKTPALKQKARDVQCDDMYSCPDGQTCCRLASGDWGCCPIAQAVCCDDHEHCCP
PGYTCSGGQCQKGEHSIPLFSKTPALRQETKYVKCDDTHSCADGQTCCRLASGDWGCCPIAQAVCCDDHE
HCCPPGYTCSGSQCMIGEHSIPWFSKTPALKQKAKDVQCDDMYSCADGQTCCRLASGDWGCCPIAQAVCC
DDHEHCCPPGYTCSGGQCQKGEHSIPWYSKTPALKQEGNIVKCDDSFACKDGYTCCRMVSGVWGCCPIEK
AVCCSDHWHCCPQGFTCDARGTCVLGQFSIPWLTKVPALPFDGTHSICDDTHTCPSGTTCCPGEGGGWRC
CPVEEKEAIETSPSDTSVQGLRLDYVWCDSQYACFDGQTCCRGLGGVWNCCVYTQGVCCPDMVHCCPYGY
VCLNHGTSCSRSGSPRWDGQSGSPHWDGKQSPRDGKRPPFL*

Transcript:

Note: This database entry and our earlier assembly from EST data are in agreement.
The cDNA data define the transcript well (below), so no predicted sequence is required.

cDNA data:

From: NCBI: NM_001078701.1 GI:118403711
Note: cDNA sequence data:

```
GGCGGGAGTAATATTCCAAAGGGTTCGAGCGGCGTCCTGTTTTAATGGCCGGGGCCTATTCAGTTAGTGG
CGCAATTTAGTAGCTGCTTGATGTATATTATGGGTGCATATGGCAGCTTTCTGCCTGGATTTGATATCCC
AGCCCGGTGCCACCCCATCTATCAAAGTACCACAGCTCCTGTGTAGTTTATGTTCCCAGGTGCTGAGCTA
TTTTTCTCAGTCATAGTTGTGTGAACACAAAAGGGTGGAAGCAATCTGGTATAAAAATATTTTTACACAT
TCTGAAACAAAAGTCTCAGCAGACTCTGATTTTGGAGTGAGCGGGGTAAGTGGGCGGAGCGCAGCGCATA
AACCGAGGTCAATTGCTGGGAAGCTGTCACATGCACAGAGCTGGGCAGGATGGGTCCACCTTGGTTTCTT
CTGCTTGTGGTCTCCACTGTTAGTGCCACACTCTGCCCAGATGGCAGCACTTGTGGAGAAAAGAGTCTGT
GCTGTGAACTGCCTGGCAAGAAGGGGTATGGCTGTTGCCCTGCGGCAGAGGTTGTGTCTCGCTCCCTTCC
TATGATTCTTTCCCAGACCTCCTGCTCAGGCTGCCCTGATGAGTATTCCTGTGTGAATACTCCAGAAGGG
GGAACCGCTTGCTGTCCATTATCTGAGGGAAAATCTTGCCAAGATGGACATCACTGCTGTTCTGTAGGCT
CCTACTGCTCAGATGATGGCCACTACTGTATCCCAGCCTCTAACCAGTCTGCTGTAGTCTGCCCGGATGG
AAGGTCTGAATGCCCCACTCTCACCACCTGCTGTATGATGTCTGACATGTCGTCATGGGGGTGCTGCCCC
ATGCCACAGGCAGTTTGCTGTGATGATCATATGCACTGCTGTCCCCATAACTCTGAGTGTGATGTCCAGC
AAGGGCGCTGTATCTCCAACCAGGACCACATTCCCTGGATGAGCAAACTTCCAGCCCGTGTGAAATCAGA
GGGCTTGAAATTAGTTGGCTTGGGAGATGAAGAACGACGGGTTCCATGCCTTGATGGCACTTTCTGCCCA
GATGGCTCTACCTGCTGTGAACAAGTAGACCACACATATGGGTGCTGCTCCATATTGTCTGCTGTCTGCT
GTTCTGACCATCTTCACTGTTGCCCTGGTGGTACCACATGTGACCTTGTCCATAAGAAATGTGTATCCCA
AACTGGGGAGGGCCCCTTATTGCCACAGATGCCAGCTATCCGGGAGGAATCAGCCAACCAGGTCCCCTGC
GATGCCACCACTAGCTGCCCGGATAAGAACACCTGCTGCCACCTGTCATCTGAGAAGTGGGGTTGCTGCC
CTTATGCACAGGCAGTGTGTTGTGACGATCATATCCACTGCTGTCCCAGTGGGTTCACATGTTCTGGAGG
CAGCTGTGTGTTGGCAGAGCACTCTATCCCTTGGATGAGGAAGACTTTGGCTCAGGGGCTGAAAACTACC
AGAGTTCAGTGTGATGACACCGCCAGCTGCCCAGAAAAGGAAACCTGCTGTCGTCTGGTATCTGGCAAGT
GGGGTTGCTGTCCTGTAGTGAAGGCTGTGTGCTGTAATGATCATCTTCATTGCTGTCCTGAAGGTTATAC
ATGCTCCCAGGGCGAATGCTCAAAAATGGAACACTCCATCCCTTGGTTCACAAAAACTCCAGCTCTGACC
CATGAAGCCAGAGATGTCGAATGTGATGATATGTACAGCTGCCCAGATGGACAAACCTGCTGCCGCTTGG
CCTCAGGCGACTGGGGATGCTGCCCAATAGCACAGGCTGTGTGCTGTGATGACCATGAACACTGCTGCCC
TCCTGGTTACACATGTTCTGGAGGAAGTTGCCAGAAGGGGGAGCTCTCTATCCCATGGTTCCTCAAGACT
CCAGCTTTGAAACAGAAGGCCAGAGATGTCCAGTGTGATGATATGTACAGCTGCGCAGATGGGCAAACCT
GCTGCCGCCTGGCATCTGGGGACTGGGGATGCTGCCCTATAGCACAGGCTGTGTGCTGTGATGACCATGA
ACACTGCTGCCCTCCTGGATACACATGCTCTGGAGGAAGTTGCCAGCAGGGGGTGCTTTCAATCCCATGG
TTCCTCAAGACTCCAGCTTTGAAACAGAATGCCAGAGATGTCCAGTGTGATGATATGTATAGCTGCGCAG
ATGGGCAAACCTGCTGCCGCCTGGCATCTGGGGACTGGGGATGCTGCCCTATTGCACAGGCTGTGTGCTG
TGATGACCATGAGCACTGCTGCCCTCCTGGTTACACATGCTCCGGAGGACAGTGCCAGAAGGGGGAGCAC
TCCATCCCATTGTTCAGCAAGACTCCAGCTTTGAAACAGAAGGCCAGAGATGTCCAGTGTGATGAAATTT
ACAGCTGCCCAGATGGGCAAACCTGCTGCCGCTTGGCCTCTGGCGACTGGGGATGCTGCCCAATAGCACG
GGCTGTGTGCTGTGATGACCATGAACACTGCTGCCCTCATGGATACATATGCTCTGGAGGAAGTTGCCAG
CAGGGGGAGCTCTCTATCCCATGGTTCCTCAAGACTCCAGCTTTGAAACAGAAGGCCAGAGATGTCCAGT
GTGATGATATGTACAGTTGCCCAGATGGACAAACCTGCTGCCGCTTGGCCTCTGGTGACTGGGGATGCTG
CCCAATAGCACAGGCTGTGTGCTGTGATGACCATGAACACTGCTGCCCTCCTGGTTACACCTGCTCTGGA
GGACAGTGCCAGAAGGGGGAGCACTCCATCCCATTGTTCAGCAAGACTCCAGCTCTGAGACAGGAGACCA
AATATGTCAAATGTGATGATACCCACAGCTGTGCAGATGGGCAAACCTGCTGCCGCCTGGCATCTGGGGA
CTGGGGATGCTGCCCTATAGCACAGGCTGTGTGCTGTGATGACCATGAACACTGCTGCCCCCCTGGATAT
ACCTGCTCTGGGTCACAGTGCATGATTGGGGAGCACTCCATCCCATGGTTCAGCAAGACCCCGGCTTTAA
AACAGAAGGCCAAAGATGTCCAGTGTGATGATATGTACAGCTGCGCAGATGGGCAAACCTGCTGCCGCTT
GGCATCTGGGGACTGGGGATGCTGCCCTATAGCACAGGCTGTGTGTTGTGATGACCATGAACACTGCTGC
CCTCCTGGTTACACATGCTCTGGAGGACAATGCCAGAAGGGAGAGCACTCCATCCCATGGTACAGCAAGA
CTCCAGCTCTGAAGCAAGAAGGTAACATAGTGAAATGTGATGATTCCTTCGCCTGTAAAGATGGTTACAC
TTGTTGTCGCATGGTCTCTGGGGTGTGGGGATGCTGCCCTATAGAGAAGGCTGTGTGCTGCTCCGACCAC
TGGCACTGCTGCCCCCAGGGGTTTACCTGTGATGCCAGAGGTACCTGTGTACTAGGCCAGTTCTCTATCC
CATGGCTCACTAAGGTTCCAGCTTTGCCCTTTGATGGGACTCACAGTATTTGTGACGATACCCACACTTG
CCCTTCTGGTACCACATGCTGCCCTGGAGAGGGGGGAGGCTGGAGATGCTGCCCAGTGGAAGAGAAGGAG
GCTATCGAAACCTCCCCCAGTGACACCTCTGTCCAGGGATTACGTCTGGATTATGTCTGGTGTGATTCTC
```

AGTATGCCTGCTTTGATGGCCAAACTTGTTGTCGAGGACTCGGAGGAGTGTGGAACTGCTGTGTTTATAC
TCAGGGGGTGTGCTGCCCAGACATGGTGCACTGCTGTCCCTATGGGTATGTGTGTCTGAACCATGGAACG
TCATGTAGTCGTTCAGGAAGCCCACGTTGGGATGGTCAGTCGGGAAGCCCACATTGGGATGGAAAACAGT
CCCCACGGGATGGTAAACGGCCCCCTTTCCTATGAACACAATCCAAATCCACTTGATTGTTACTACTAAT
AAGATCCTTCTAAAAGCACTGGAATAAAATCAAATTATTTTTGTTTTATATGTTTAAAAAAAAAAAAAAA

Genomic data:

Note: Genomic data (exons). The exons are derived from two genomic sequences. The first should cover the whole gene, since it has exons encoding the first paragranulin and five modules and the last five and a half modules including the second paragranulin. But it misses exons necessary for the four repeat modules in the centre of the assembled transcript. The second is a shorter contig which has these missing exons along with two upstream and six downstream exons. Exons are numbered according to expectations from the cDNA seqence.

From NCBI: AAMC02035398.1 GI: 428718064

Note: An error in assembly of AAMC02035398.1 has joined high-identity sequences at the end of coding exon 12 and between coding exons 20 and 21, thus omiting 8 exons encoding 4 highly similar modules.

Coding exon 1 **Exon type: sn**

Note: from start codon

ATGGGTCCACCTTGGTTTCTTCTGCTTGTGGTCTCCACTGTTAGCGCCACACTATGCCCAGATGGCAGCA
CTTGTGGAGAAAAGAGTCTGTGCTGTGAACTGCCTGGCAAGAAGGGGTATGGCTGTTGCCCTGCGGCAGA
G

Coding exon 2 **Exon type: n***

Note: * Encodes the N-half of a 10 cys module

GTTGTGTCTCGCTCCCTTCCTATGATTCTTTCCCAGACCTCCTGCTCAGGCTGCCCTGATGAGTATTCCT
GTGTGAATACTCCAGAAGGGGGAACCGCTTGCTGTCCATTAGCTGAG

Coding exon 3 **Exon type: c***

Note: * Encodes the C-half of a 10 cys module

GGAAAATCTTGCCAAGATGGACATCACTGCTGTTCTGTAGGCTCCTACTGCTCAGATGATGGCCACTACT
GTATCCCAG

Coding exon 4 **Exon type: n**

CCTCTAACCAGTCTGCTGTAGTCTGCCCGGATGGAAGGTCTGAATGCCCCACTCTCACCACCTGCTGTAT
GATGTCTGACATGTCGTCATGGGGGTGCTGCCCCATGCCACAG

Coding exon 5 **Exon type: c**

GCAGTTTGCTGTGATGATCATATGCACTGCTGTCCCCATAACTCTGAGTGTGATGTCCAGCAAGGGCGCT
GTATCTCCAACCAGGACCACATTCCCTGGATGAGCAAACTTCCAGCCCGTGTGAAATCAGAGGGCTTGAA
ATTAGTTGGCTTGG

Coding exon 6 **Exon type: n**

GAGATGAAGAACGACGGGTTCCATGCCTTGATGGCACTTTCTGCCCAGATGGCTCTACCTGCTGTGAACA
AGTAGACCACACATATGGGTGCTGCTCCATATTGTCT

Coding exon 7 **Exon type: c**

GCTGTCTGCTGTTCTGACCATCTTCACTGTTGCCCTGGTGGTACCACATGTGACCTTGTCCATAAGAAAT
GTGTATCCCAAACTGGGGAGGGCCCCTTATTGCCACAGATGCCAGCTATCCGGGAGGAAT

Coding exon 8 **Exon type: n**

CAGCCAACCAGGTCCCCTGCGATGCCACCACTAGCTGCCCGGATAAGAACACCTGCTGCCACCTGTCATC
TGAGAAGTGGGGTTGCTGCCCTTATGCACAG

Coding exon 9 **Exon type: c**

GCAGTGTGTTGTGACGATCATATCCACTGCTGTCCCAGTGGGTTCACATGTTCTGGAGGCAGCTGTGTGT
TGGCAGAGCACTCTATCCCTTGGATGAGGAAGACTTTGGCTCAGGGGCTGAAAA

Coding exon 10 **Exon type: n**

CTACCAGAGTTCAGTGTGATGACACCGCCAGCTGCCCAGAAAAGGAAACCTGCTGTCGTCTGGTATCTGG
CAAGTGGGGTTGCTGTCCTGTAGTGAAG

Coding exon 11 **Exon type: c**

GCTGTGTGCTGTAATGATCATCTTCATTGCTGTCCTGAAGGTTATACATGCTCCCAGGGCGAATGCTCAA
AAATGGAACACTCCATCCCTTGGTTCACAAAAACTCCAGCTCTGACCCATGAAG

Coding exon 12 Exon type: n
Note: The end of this exon is approximately where duplications of about 450 to 600 bases resulted in the repeat of 4 nearly identical modules in the middle of the progranulin. It is where the wrong downstream sequence was incorporated by the sequence assembly process.
CCAGAGATGTCGAATGTGATGATATGTACAGTTGCCCAGATGGACAAACCTGCTGCCGCTTGGCCTCTGG
TGACTGGGGATGCTGCCCAATAGCACAG

Coding exon 21 Exon type: c
Note: This exon wrongly assembled to follow exon 12 in AAMC02035398.1. The intervening exons are presented in the companion genomic DNA contig in this entry.
GCTGTGTGCTGTGATGACCATGAACACTGCTGCCCTCCTGGTTACACCTGCTCTGGAGGACAGTGCCAGA
AGGGGGAGCACTCCATCCCATTGTTCAGCAAGACTCCAGCTCTGAGACAGGAGA

Coding exon 22 Exon type: n
CCAAATATGTCAAATGTGATGATACCCACAGCTGTGCAGATGGGCAAACCTGCTGCCGCCTGGCATCTGG
GGACTGGGGATGCTGCCCTATAGCACAG

Coding exon 23 Exon type: c
GCTGTGTGCTGTGATGACCATGAACACTGCTGCCCCCCTGGATATACCTGCTCTGGGTCACAGTGCATGA
TTGGGGAGCACTCCATCCCATGGTTCAGCAAGACCCCGGCTTTAAAACAGAAGG

Coding exon 24 Exon type: n
CCAAAGATGTCCAGTGTGATGATATGTACAGCTGCGCAGATGGGCAAACCTGCTGCCGCTTGGCATCTGG
GGACTGGGGATGCTGCCCTATAGCACAG

Coding exon 25 Exon type: c
GCTGTGTGTTGTGATGACCATGAACACTGCTGCCCTCCTGGTTACACATGCTCTGGAGGACAATGCCAGA
AGGGAGAGCACTCCATCCCATGGTACAGCAAGACTCCAGCTCTGAAGCAAGAAG

Coding exon 26 Exon type: n
GTAACATAGTGAAATGTGATGATTCCTTCGCCTGTAAAGATGGTTACACTTGTTGTCGCATGGTCTCTGG
GGTGTGGGGATGCTGCCCTATAGAGAAG

Coding exon 27 Exon type: cn
GCTGTGTGCTGCTCCGACCACTGGCACTGCTGCCCCCAGGGGTTTACCTGTGATGCCAGAGGTACCTGTG
TACTAGGCCAGTTCTCTATCCCATGGCTCACTAAGGTTCCAGCTTTGCCCTTTGATGGGACTCACAGTAT
TTGTGACGATACCCACACTTGCCCTTCTGGTACCACATGCTGCCCTGGAGAGGGGGGAGGCTGGAGATGC
TGCCCAGTGGAAGAG

Coding exon 28 Exon type: n
AAGGAGGCTATCGAAACCTCCCCCAGTGACACCTCTGTCCAGGGATTACGTCTGGATTATGTCTGGTGTG
ATTCTCAGTATGCCTGCTTTGATGGCCAAACTTGTTGTCGAGGACTCGGAGGAGTGTGGAACTGCTGTGT
TTATACTCAG

Coding exon 29 Exon type: c
Note: up to the stop codon (3'ut continues in the exon)
GGGGTGTGCTGCCCAGACATGGTGCACTGCTGTCCCTATGGGTATGTGTGTCTGAACCATGGAACGTCAT
GTAGTCGGTCAGGAAGCCCACGTTGGGATGGTCAGTCGGGAAGCCCACATTGGGATGGAAAACAGTCCCC
ACGGGATGGTAAACGGCCCCCTTTCCTATGA

From NCBI Trace Archive: TI in (443961751, 422295050, 450798742, 240880237, 479158468, 415700498, 416062972, 248893834, 391890786, 243494173, 418516449, 389713143, 242525690, 408119643, 391766295, 242742823, 414002177, 412560105, 414225439, 419262832, 423416245, 584721614, 389645324, 422621756, 241665798, 249207218, 415658536, 413407419, 391890402, 389681264, 242821231, 243494269, 569866661, 430948189, 475372848, 421849771, 413358776, 552245266, 418310929, 451013482, 242525306, 417339606, 415700879, 389785261, 418505765, 391397092, 416626534, 242742439, 431156322, 241665421, 412007748, 435260900)
Note: A contig assembled from these Trace archive sequences (via Cap3, including quality)

Coding exon 11 **Exon type: c**
GCTGTGTGCTGTAATGATCATCTTCATTGCTGTCCTGAAGGTTATACATGCTCCCAGGGCGAATGCTCAA
AAATGGAACACTCCATCCCTTGGTTCACAAAAACTCCAGCTCTGACCCATGAAG

Coding exon 12 **Exon type: n**
CCAGAGATGTCGAATGTGATGATATGTACAGTTGCCCAGATGGACAAACCTGCTGCCGCTTGGCCTCTGG
TGACTGGGGATGCTGCCCAATAGCACAG

Coding exon 13 **Exon type: c**
GCTGTGTGCTGTGATGACCATGAACACTGCTGCCCTCCTGGTTACACATGTTCTGGAGGAAGTTGCCAGA
AGGGGGAGCTCTCTATCCCATGGTTCCTCAAGACTCCAGCTTTGAAACAGAAGG

Coding exon 14 **Exon type: n**
CCAGAGATGTCCAGTGTGATGATATGTACAGCTGCGCAGATGGGCAAACCTGCTGCCGCCTGGCATCTGG
GGACTGGGGATGCTGCCCTATAGCACAG

Coding exon 15 **Exon type: c**
GCTGTGTGCTGTGATGACCATGAACACTGCTGCCCTCCTGGATACACATGCTCTGGAGGAAGTTGCCAGC
AGGGGGTGCTTTCAATCCCATGGTTCCTCAAGACTCCAGCTTTGAAACAGAATG

Coding exon 16 **Exon type: n**
CCAGAGATGTCCAGTGTGATGATATGTATAGCTGCGCAGATGGGCAAACCTGCTGCCGCCTGGCATCTGG
GGACTGGGGATGCTGCCCTATTGCACAG

Coding exon 17 **Exon type: c**
GCTGTGTGCTGTGATGACCATGAGCACTGCTGCCCTCCTGGTTACACATGCTCCGGAGGACAGTGCCAGA
AGGGGGAGCACTCCATCCCATTGTTCAGCAAGACTCCAGCTTTGAAACAGAAGG

Coding exon 18 **Exon type: n**
CCAGAGATGTCCAGTGTGATGAAATTTACAGCTGCCCAGATGGGCAAACCTGCTGCCGCTTGGCCTCTGG
CGACTGGGGATGCTGCCCAATAGCACGG

Coding exon 19 **Exon type: c**
GCTGTGTGCTGTGATGACCATGAACACTGCTGCCCTCATGGATACATATGCTCTGGAGGAAGTTGCCAGC
AGGGGGAGCTCTCTATCCCATGGTTCCTCAAGACTCCAGCTTTGAAACAGAAGG

Coding exon 20 **Exon type: n**
CCAGAGATGTCCAGTGTGATGATATGTACAGTTGCCCAGATGGACAAACCTGCTGCCGCTTGGCCTCTGG
TGACTGGGGATGCTGCCCAATAGCACAG

Coding exon 21 **Exon type: c**
GCTGTGTGCTGTGATGACCATGAACACTGCTGCCCTCCTGGTTACACCTGCTCTGGAGGACAGTGCCAGA
AGGGGGAGCACTCCATCCCATTGTTCAGCAAGACTCCAGCTCTGAGACAGGAGA

Coding exon 22 **Exon type: n**
CCAAATATGTCAAATGTGATGATACCCACAGCTGTGCAGATGGGCAAACCTGCTGCCGCCTGGCATCTGG
GGACTGGGGATGCTGCCCTATAGCACAG

Coding exon 23 **Exon type: c**
GCTGTGTGCTGTGATGACCATGAACACTGCTGCCCCCCTGGATATACCTGCTCTGGGTCACAGTGCATGA
TTGGGGAGCACTCCATCCCATGGTTCAGCAAGACCCCGGCTTTAAAACAGAAGG

Coding exon 24 **Exon type: n**
CCAAAGATGTCCAGTGTGATGATATGTACAGCTGCGCAGATGGGCAAACCTGCTGCCGCTTGGCATCTGG
GGACTGGGGATGCTGCCCTATAGCACAG

Coding exon 25 **Exon type: c**
GCTGTGTGTTGTGATGACCATGAACACTGCTGCCCTCCTGGTTACACATGCTCTGGAGGACAATGCCAGA
AGGGAGAGCACTCCATCCCATGGTACAGCAAGACTCCAGCTCTGAAGCAAGAAG

Coding exon 26 **Exon type: n**
GTAACATAGTGAAATGTGATGATTCCTTCGCCTGTAAAGATGGTTACACTTGTTGTCGCATGGTCTCTGG

GGTGTGGGGATGCTGCCCTATAGAGAAG

Genomic sequence note: This genomic sequence was obtained by splitting the portion of AAMC02035398.1 which contains progranulin coding exons and assembling it with the trace wgs contig specified in this entry to provide the sequence containing all predicted exons.

TGCTTTGTGTACAAGGTTTTTAACACTTTGCTGTCTGATTATTTTCCCCCCCTCAGGATGGGTCCACCTT
GGTTTCTTCTGCTTGTGGTCTCCACTGTTAGCGCCACACTATGCCCAGATGGCAGCACTTGTGGAGAAAA
GAGTCTGTGCTGTGAACTGCCTGGCAAGAAGGGGTATGGCTGTTGCCCTGCGGCAGAGGTAGGTAGAGGG
AAGCATGGGGGCTGCACAACTGCCACATAGAGTAGAAGATCTAGATAAATACCCTCAGCTGTGGGTTTTT
CAACATTAGGACATTATGGTGCCTAGAATGATTTATGGTGCTCTGTTGAACCAAACAAAAGTGCTACAAT
GACCCTTTCTCCTTTTAGGTTGTGTCTCGCTCCCTTCCTATGATTCTTTCCCAGACCTCCTGCTCAGGCT
GCCCTGATGAGTATTCCTGTGTGAATACTCCAGAAGGGGGAACCGCTTGCTGTCCATTAGCTGAGGTAAG
CATTGGTCATTGAATAAGGACCCTAGTGATACAGGAAACTTATTTGACAGTGACCACAGCTAAAGGAAGA
GACTCTGTTTTGGGAGTCTTGCTTTCATAAGACTGGTTCATGGGGCAAGTTTTCAGAAAGCCTTCATATA
GTAGGGCTTTTTCTCTGGGCACAAAGGGGGTTCTTTAAAATTCTGTTTAAAGGCTGCTGTATCCTTATAC
AGTGGCATGCTTGGTGGTGTTTTCGGTGTCTTGGTGCATGAAACCTCTCGGTTTGGCTCTCAAAGGAGTG
TTACTGGTTCAGACTCCAGTTTATTGCTCTATCGTGACATCAATTGAGATGTTAGGCCAGAGGTGGCCTT
GGGCTTTGCCATCAATATGCCCCCCTGCCCTTTCCTTTTTTTTTTTTTGTGGAATGGGTGACTGGCACCC
TATCTTGAGACCGGCACCCTATCCGGTGACAAGTATAAAATATATGGTTAGGTTAACTTTTCAGGAAGCA
GCAAGTCTTAACTTTCTGTAAATGACTTTTTGTTTTGTGATTTCTTGCAAAACTTAGGGAAAATCTTGCC
AAGATGGACATCACTGCTGTTCTGTAGGCTCCTACTGCTCAGATGATGGCCACTACTGTATCCCAGGTTA
GAGATCTCATCTGCTATAACTTCCCCCTTCCATCTTTGATAATTTCTACTTAAACATTGTGTTTTGTTTT
TGTTTTTTTTTACAGCCTCTAACCAGTCTGCTGTAGTCTGCCCGGATGGAAGGTCTGAATGCCCCACTCT
CACCACCTGCTGTATGATGTCTGACATGTCGTCATGGGGGTGCTGCCCCATGCCACAGGTAAGAGGACCC
AGTTCTTATGCCTACTGTTTTAAATTCCCATACTACAGACCCTTGACAAAAAAAAATCCGCTCCCAATAA
CCTCTAACTGCCAAATACTGGGAGATGTAGTAAAGCTTAAGGGTTCCAGGTTGTACATGTAAAGTTCAGG
CTTGAACTGGCCGCAACCGCCCCCCACCCCCAGTGCTCTTCAAGGCAAGGGGGGGGGGGGGCAAAGGGTC
CTCCATCCACCTTTCCCTAATCACCTTTCCCTAATCTGCAGCTGCCAACATGATATATTTATGAAGAGTT
CACAGGGGGTCACGTTGAGTGTCCTTTTATTATTTATTTGATTATTGAAACTTGGCAGTAGCTGCTGCAT
TTCCCACCCTAGGCTTATACTCGAGTCAATAAGTTTTTCCAGTTTTCTTGGGTAAAATTAGGTACCTCGG
CTTATATTCGGATCGGTTTATACTCGAGTATATACGGTAACTTGAAATGAATAGATTTTTCCCCATGACT
CCTTAACTGACCAGAAAGCCCACATGGATCTTGCTGTGCCAGGGTTAATGTTCTCTCTGTCTGACCATTC
TGCAGGCAGTTTGCTGTGATGATCATATGCACTGCTGTCCCCATAACTCTGAGTGTGATGTCCAGCAAGG
GCGCTGTATCTCCAACCAGGACCACATTCCCTGGATGAGCAAACTTCCAGCCCGTGTGAAATCAGAGGGC
TTGAAATTAGTTGGCTTGGGTAAGCTCTACCAACTTGTTACAACTACCAACTTTTATTCTGTGGGGCCAA
GGTTTTCATAACTTCTTCTTGACAACTGGACTGACATAGATATTCTGCTTAAAATATGCTATTTATTTAA
AATTTTAATATCATAGTACATTTAAGGCTTATGCGTTTGGTGCCTATACTGGGCACTTAATCATAGGCCA
AGGATATAGGTATAGACCTCATAGTCAGGGTCACCTTTTTATTTTAGAGAAGCTTTCATTAAAGGAGAAG
GAAAGGCTAATAAAGAGTTAATCTCAAGCTGCATGCATACCTTCAGTTCTCTCAATAGTGCCCTTAAGTC
TCCCCATATTTCTCCCATTCAGATGATCAGAAGCCTCATAGGAAAAAAAAATACTCTGAGCTGTGTAAAG
AAATGCCTCGCTCCTGCACCAAGACCAAGACTGGTGTACATGCTCAGTGTGTAAGACTATATGATAAAGC
TTCCTGCTGATTGGCTCAGATCACACATTCCTAAGGGGGGGAGGGAGTTCTTGGCATTCTTGAGGGAGGG
GGGGAGCAGGAGAGAGGAGAGAGCTGCGTGTCTCTGGCAGAGGAAAACAAACAGACAATAAATCCTGTAT
CTTTTGAAAGAGAACTCCAGTGCAGCGTTTCTGTGTGTGCTTATGGCTGTATTTACATAGGCCTTTCTGA
TAAAGCTTACTAAAGGTCCCCATACACGGGCCGATTATAGCTGCCGATATCGGTCCCTTGGACCGATTCG
GCAGCTTATCGGCTCGTGTAGGGGCAGAACCAAGGGGCCTGGCCGACCGATATCTGGCCTGAAATTGGGC
AGATATCGATCGGCCAGGTTAGAAAATCCAGTCGGATCGGGGACCGCATGGGCTCGTCGATGCGGTCCCC
GAACCGACTGCCCCATTGCCACGTGTAGAATTCGATCCTTTGGCCCCAGGGCCAAACTATCGAATTCGCC
TACATGCCTCCCCGATATCGCCCACCCGTAGGTGGGGCTATCGGGTGAAGATCGCCAAGCGAGCGGATCT
GGCCGTGTATGGCCAGCTTTAGTTTTTACCTTTCCTTCTCCTTTAAATATAAGGAATAAGGGGCGAATTG
GAAACTTAGTGTTACTCAGCAGTACTGCTATTTTATTTTCTTTCTGGCATGTAGGAGATGAAGAACGACG
GGTTCCATGCCTTGATGGCACTTTCTGCCCAGATGGCTCTACCTGCTGTGAACAAGTAGACCACACATAT
GGGTGCTGCTCCATATTGTCTGTAAGTGTTTCCTCATTGCTTTATTAATATGTGCATAGAAACCTTTTCC
TCTGGTTAGTCCTAAGTACACTTTGCAATGCAATTAAAGGGGAAGTTCACCTTTTAGTATGTCATAGATG
ACTTTATTCTAAGTACTTTAGAAATGTATACCCCCTTTAAAAGAAAATATATCATGGTTTAAGCCCTGGG
CTGCCGACTACCTGTGTCTTTTTGGCCTTTTAAATCTGATTATCTAGTGTAAAGGGTCAGGACCACAACA
GATGAAAATCTGCACAATGCACAAATTCTTTCAGAAATTGTATGTAATTCCTGGAAAGCGTGATATTTTT
GTCATTACTGCTTGTATTTAAATTAAAGGCAGGCAGTACAGCCCTTCTCGCTGGCTTTGTGTGGTCTAGT
AACCCAGCAGTACAAAGTACCCAAATCTAGGAAATGCTCGTCATCAGCCACATAGTTCTCAATGAGAAAC
ATGTTGCCACAATCCTGTAACTCAAAGCAAACCAATCGGATGTTTGATTTCATGTGGTTATCTGGGCTGC

TATGGGCTACTGCACCAAGGCCAACATTTCTTGTAATGCTACATAACTCCCACAGTGGTAGCCCTTCATT
TCAGGTTAACTGCACTTTTACCTGATTGATGGTCATGGTATGTTTCTTTTAGGCTGTCTGCTGTTCTGAC
CATCTTCACTGTTGCCCTGGTGGTACCACATGTGACCTTGTCCATAAGAAATGTGTATCCCAAACTGGGG
AGGGCCCCTTATTGCCACAGATGCCAGCTATCCGGGAGGAATGTAAGTCCGTGTGGGGTTTAATTGCCCA
GTTTACGGCGCTTTAACTGAATTTCTTTAGTACTTTGCATGCACTTGTTGGCAGGGGAAGAAAGGTAGGG
GAAGAGGCACACAGGGGTTAAGATCTGTAGATTGAAATTCCATATAGGCCAATGAGAGAAACCACTGTTC
TAATTCCTAAAACTGTGGCAGAATGGGCAATACCCAGATGTTTGCTATTAACTGTAACCATGTTACAAGC
TTAGGGGACCTCCACCAAAGATGCCAAAGATGGACCTCCATGGGAGGGGAGGGCTTGAAATTTAAACCTT
TGACAACTATTGGATTAATGGAATTAAAATGCAAGATGTTTGCCCTGCAAGGCATTGAGGCAAAGTAATT
TTGTTTGTGGGAGTGTGGCACCTTGGGTACCTGTGGTTTGGGTGTGGCAGAGCATTCCGATCTACTGTAA
ATTTAGCACCACTTCTTCCTTCCTTCTCAGCTGCTGCAACCTGCAATTACATGGCAGATATCTCTGTATG
TACCATGACTGTCCAGAACAAATTGGACTACACAGGGAAATAGCGTGGTTAGCTTTGATAGATCATGTAT
ATGGCCAAATAGCAGGATTTAATATTATGAGAGCTGACTCTTAAGTGGTGTAATGGTTACAACGCTATAA
AGATAGTAGCTTGCTGCTCCTTGGGAGTGTAGAAGAAATGTAGTGCCTTCATGGATGAATTGTGCTCCGT
CTCAGCAGCCAACCAGGTCCCCTGCGATGCCACCACTAGCTGCCCGGATAAGAACACCTGCTGCCACCTG
TCATCTGAGAAGTGGGGTTGCTGCCCTTATGCACAGGTATAAACTAATGAAGTCTGCTGGTTCTTACGGC
TTAATAGTGTGAACATTCAGAGCTACAATACTGAGACATGTTCATTCTCCTGTCCCCACTTAGGCAGTGT
GTTGTGACGATCATATCCACTGCTGTCCCAGTGGGTTCACATGTTCTGGAGGCAGCTGTGTGTTGGCAGA
GCACTCTATCCCTTGGATGAGGAAGACTTTGGCTCAGGGGCTGAAAAGTATGTAGTCATAACATTCATCC
CTCTCTAGCTCTAACTTTAAGTGTGAGGCCATGTAAAGATTTTTGAGTCTAACCTTTTCTTTTTTTTTTT
TTTTTGTTTAGCTACCAGAGTTCAGTGTGATGACACCGCCAGCTGCCCAGAAAAGGAAACCTGCTGTCGT
CTGGTATCTGGCAAGTGGGGTTGCTGTCCTGTAGTGAAGGTGCAATGCCTTGTATTTGTTTTTCAGAATT
TGTATGTATTTGCATACTCCTTGGCCTCCTGCTTTCTGGAATTGGACTAGCTTTTCCTTTGCAAAGTCCT
CATGATATGCTGTAAATGTCTCCAACACAGAGTAGAAGTAGCCAACGTCATTCCAACCATATGAAGTGTA
TAAAGGTTAAATGTTGGGCTTGTCCTCAGTTTGGTAGTTCCAATGACAGATCATCTTATCTGAGCAGGAA
TATATATCTACATCATGACCTGTTATGGAGCTGCATTATATGGTCAGGCAGTTTCAGTGAGTAATAGTCT
GATCTTATTGATATAGTCTTAAGTTCTGCATGCTCATAACTGGCAGCAACAGCCCCTCCCATCAGCAAGT
GGATCGTATTAAAACTTTCTATTGGTACTTTGTCCCAATTATACAATATCCAGGCTGAGTACAGCAAATT
TCCCTTATAGTTGCTGCCTTTAGTCTAGTGTTACTCATGGAATTTAGGTAACCTTGTGACTTCCCAGCAG
GCTTGGACTGGCAGTTTTTGGATTTTGGCAAATGCCAGTGGGGCTGCTATAATTTGCCGTAAACGGTCAC
TATGTATTGGGCCTGTGGCGGGCTGTTTGAGCTTTTGTGTACTTCAACTGCCAGAAACTATTTTGAATCC
CAACCCAGACTTGCTTCTCCGCAAGATAAGACATGGTCATGGTCATTTAAAAATAAAATTTCTTACAATA
CCTCCCTTAAATTGTCAAATGCACTTATCTGTAGCCACATAATGTAAAATGTTTGCACTTCACATCTTTT
TTTGAGAATTTATTAGCAACTTGGAGGGAAAGCCTCTATGTAGTCTGGTTTTCTTATTAATTCCTTATAT
GAAATGTTTTATTTAGTCATTTTCCTCCTTTCCCAGGCTGTGTGCTGTAATGATCATCTTCATTGCTGTC
CTGAAGGTTATACATGCTCCCAGGGCGAATGCTCAAAAATGGAACACTCCATCCCTTGGTTCACAAAAAC
TCCAGCTCTGACCCATGAAGGTAAGTGGTGAAACTTGGGTTGGAGTGTTGAAGGGTCTTCCAATCTGCAG
GTGTTAACCTAAACTTGGCTTCACATGGCAATATGCCTGAGAATGGTATAGAGAGATCAGTTGATGCACT
GTTTGCTTGCTGGAGCAATCTACTAATTACATTATGGACTTGGGCAGGTGGTAAATATGGAACTCCTTTG
TACCTTTTGCATTTGTACTATACAAGACCAAGCTACAATAGCAGCCAAGGTGAATAGTAAGGGCATTGGA
CCAGCAGTATGGAATAAATCTAAGAAACTGGCAGTGGCTGCCCATACTAGATGGTTTTCTTTCTCTCACT
TTCCCTTCTCTCCATTCTCCCTGTTGTATCTGTAGCCAGAGATGTCGAATGTGATGATATGTACAGCTGC
CCAGATGGACAAACCTGCTGCCGCTTGGCCTCAGGCGACTGGGGATGCTGCCCAATAGCACAGGTATGAC
AGCTTGTTTGGTACCAACCAACCAGAAGTCTCTCGCCCTCTGAGGGAAGAGTGTGTGGTTACTATATGTT
GTATATCTTAATCCCTTTCCAACAATTCCTTTTGTTCCACCCCAGGCTGTGTGCTGTGATGACCATGAAC
ACTGCTGCCCTCCTGGTTACACATGTTCTGGAGGAAGTTGCCAGAAGGGGGAGCTCTCTATCCCATGGTT
CCTCAAGACTCCAGCTTTGAAACAGAAGGGTAAGTGGCAAAATCAGGATCTAGACTTCATGTGTGTTGCA
AAACACACAAATAGGAGAATCCAAGACCATTTTATAAGACATGGCCCATTAAACATGTTCTCTAGTGGTT
TTGTTTTTTCTTCATGATTGACTCTCCTTTCCAATCCCTGTTTTCTATAGCCAGAGATGTCCAGTGTGAT
GATATGTACAGCTGCGCAGATGGGCAAACCTGCTGCCGCCTGGCATCTGGGGACTGGGGATGCTGCCCTA
TAGCACAGGTGTGAGAATTTGCGGTCTTATATTATAATTTCTCAAGAGTCAACTTTTTGTCAACCTGTCC
TTGGCTTCCACTTCTGACCCATTCCAAGACTTCCAATTCACCTTTACTTTACAACATTAACTTCTCTCAC
CCTCTGAGGAAAGAGTGTGTGGTCACTATATGTTGTATATCTTGTTCCCTTTCCAACAATTCCTTTTGTT
CCCACCCCAGGCTGTGTGCTGTGATGACCATGAACACTGCTGCCCTCCTGGATACACATGCTCTGGAGGA
AGTTGCCAGCAGGGGGTGCTTTCAATCCCATGGTTCCTCAAGACTCCAGCTTTGAAACAGAATGGTAAGT
GGCGAAATCAGGGGAGACTGGATCTAGACTTCATGTGTGTGACAAAACACACAAATAGGAGAATCCAAGA
CCATTTTACAAGACATGGCCCATTAAACATGTTCTCTAGTGGCTTTGTGTTTTCTTCATGGTTGACCCTC
CTTTCCAATCCCCGTTTTCTGTAGCCAGAGATGTCCAGTGTGATGATATGTATAGCTGCGCAGATGGGCA
AACCTGCTGCCGCCTGGCATCTGGGGACTGGGGATGCTGCCCTATTGCACAGGTATGAACACTTGTTAGG
TACCAACCCATAAATCGCTCGCTTTCTGAGGGAAGAGTGTGTGGTCACTATGTGTATCCTGTTCAAATAA

TTTCTTCTGTCCCCACCCCAGGCTGTGTGCTGTGATGACCATGAGCACTGCTGCCCTCCTGGTTACACAT
GCTCCGGAGGACAGTGCCAGAAGGGGGAGCACTCCATCCCATTGTTCAGCAAGACTCCAGCTTTGAAACA
GAAGGGTAAGTGGCGAAATCAGGATCTAGACTTCATGTGTGTTGCAAAACACACAAATAGGAGAATCCAA
GACCATATTACAAGACATGGCCCATTAAACATGTTCACTAGTGGTTTTGTTTTTCCTTCATGAATGACCC
TCCTTTCCAATCCCCATTTTCAATAGCCAGAGATGTCCAGTGTGATGAAATTTACAGCTGCCCAGATGGG
CAAACCTGCTGCCGCTTGGCCTCTGGCGACTGGGGATGCTGCCCAATAGCACGGGTATGACAGCTTGTTT
GTTACCAACCCATAAGTCTCTCGCCCTCTGATGGAAGAGTGGGTGGTCACTTTATGTATCCTGTTCCAAC
AATTTCTTCCGTCTCCACCCCAGGCTGTGTGCTGTGATGACCATGAACACTGCTGCCCTCATGGATACAT
ATGCTCTGGAGGAAGTTGCCAGCAGGGGGAGCTCTCTATCCCATGGTTCCTCAAGACTCCAGCTTTGAAA
CAGAAGGGTAAGTGGCAAAATCAGGGGAGACTGGATCTAGACTTAATGAGTGTTACAAAACACACACAAA
TAGGAGATTCCAAGACCATTTTACAAGACATGGCCCATTAAACATGTTCTCTAGTGGCTTTGTTTTTTCT
TCATGATTGACTCTCCTTTCCATTCCCCATTTTCAATAGCCAGAGATGTCCAGTGTGATGATATGTACAG
TTGCCCAGATGGACAAACCTGCTGCCGCTTGGCCTCTGGTGACTGGGGATGCTGCCCAATAGCACAGGTA
TGACAGCTTGTTTGGTACCAACCCATAAGTCTCTTGCCCTCTGAGGGAACAGTGGGTGGTCACTATATGT
ATCCTGTTCCAACAATTTCTTCTGTCCCCACCCCAGGCTGTGTGCTGTGATGACCATGAACACTGCTGCC
CTCCTGGTTACACCTGCTCTGGAGGACAGTGCCAGAAGGGGGAGCACTCCATCCCATTGTTCAGCAAGAC
TCCAGCTCTGAGACAGGAGAGTAAGTTCTGAAAACCTTTTACATTAGTTTATCAGGTGCCTGGGATTCAG
TTCCTCCAAACAGAAGTTCCCAAACTTGTTCTGGTATCCTTGTTACATGGGATTTATTGTGCATTTATTG
TGTGATGTGAATGATGTATAATCATGTGTGGGTGAGCAAATAAATAGGATCACATGAGGATCAGTATGTG
CTTGCGGCATTAAATTCTGGCTGAAGCTTCTGTGTTTACCAGTCGGGCTGTTACTGAACTACTTACCTGT
GTAGACAACCAAAATATCACGCTCAAGTCCTATAAACATGACCAGTATATCTGGCAATGAAATCGCCAAA
TTCTAATGGTAGCTGCCTGTGCCAGAAGATGTGGTGATGATGAATATCCATTTCCTCTGTGCCTACTTTT
CTCTCCCTTCATCTGTCCTTTTTCTGTAGCCAAATATGTCAAATGTGATGATACCCACAGCTGTGCAGAT
GGGCAAACCTGCTGCCGCCTGGCATCTGGGGACTGGGGATGCTGCCCTATAGCACAGGTATGCGAATTTG
GTAGTCTTTCTCAAGTCCAACTTCCATTTTGTTAGACTTGCATTGGCTTCCGCTTGTGAAACATTCCAAG
ACTTCCAATTCAGCTTTACTTTACAACATTAACTTCATCTGTCCCTGCTCCAGGCTGTGTGCTGTGATGA
CCATGAACACTGCTGCCCCCCTGGATATACCTGCTCTGGGTCACAGTGCATGATTGGGGAGCACTCCATC
CCATGGTTCAGCAAGACCCCGGCTTTAAAACAGAAGGGTAAGTGGCAAAATCATGGGAGACAGGATCTAG
ACTACATGTGTGTGTTACAAAACACACAAATAGGAGAAAGACCTGGCCCATTAAACATGTTCTCTAGTGG
CTTTGTTTTTTCTTTATGATTGACTCTTCTTTCCAATCCCCGTTTTCTATAGCCAAAGATGTCCAGTGTG
ATGATATGTACAGCTGCGCAGATGGGCAAACCTGCTGCCGCTTGGCATCTGGGGACTGGGGATGCTGCCC
TATAGCACAGGTGTGAGAATTTGTGGTCTTATATTGTAATTTCTCAAGAGTCAACTTTTTGCCAACCTCT
TCTTGGCTTCCACTTCTGACCCATTCTAAGACTTCCAATTCAACTTTACTTTACAACATTAACTTCATCT
GTCCCTGCTCCAGGCTGTGTGTTGTGATGACCATGAACACTGCTGCCCTCCTGGTTACACATGCTCTGGA
GGACAATGCCAGAAGGGAGAGCACTCCATCCCATGGTACAGCAAGACTCCAGCTCTGAAGCAAGAAGGTA
AGTGTAGACCGGGGTAAAGGAAAGACCAGGCCTACAAAAGCCGTAGCTTGTACACTGCCTGGAAAGCGTT
TAGTTATCAGCTGACTTGCCAGTTACTGGTGATATGGCATCAATTGTAAACATGTTTATGTCTCTTGAGA
CATCAGTAGAATTTGGCAGCCAATTATCTCATTGCACCAATTTTCATAAGGCAATTTGGCTAACACTCCT
GTCAATGTTTTTGCAGGTAACATAGTGAAATGTGATGATTCCTTCGCCTGTAAAGATGGTTACACTTGTT
GTCGCATGGTCTCTGGGGTGTGGGGATGCTGCCCTATAGAGAAGGTATGAGCTGGCGTCATTAACCTACA
GAAGGGGATATCCAATATGGTTATGGAAACTTTGCATGTGAAAGGTTCATACTGAGAGGATGGAACACAT
CGTATTCTTGTTCTAATTGGGATGCACCGAATCCAAAATTCTGCCAAGATTCTGTCTTTTTCAGCAGGAT
ATGGAGTCGGCTGAATCCACGCTCCTGGCCAATCCGAATCCCTAAAATCATGTGACTTTGTCACACACTT
TTTTTCGCCAAATGCTTTGCACATGCAAATTAGGATTCAGGTTTGGTATTCGGCCAAATTTTTTACAAAG
TATTCGGGGTTGAATCCAAAAATAGTGGATTCGGTTCCTCCCGACTCTGAACATTCTAATACTCTGGCTG
CAGATATGCTTTAGTCTGGCCCACCTAAAAGTTGGCTACACAGTCATGTATACCCCATAAAAAAAAGATT
GAAAAGAATCTGGGTAAGCAGAATGAGTCAAGGTACAGCTGTCTTGTGAAGTCTTAAATTAAGCATTGGC
CTTTGTCATATCAGATGTGTAAGATACCTTATCTTATTAATGTGACTGGATCATTTAGGTATCAATTCCT
TCCGGAACAAGCTTTCGTAGCCCTTTCCTGTCCCTGTGGTCACTACTGTCCCCTTCCTTGTCAATGTTTT
CCTCTGTTCTCAGTCCTGTCACATCCTTAAGTACTCTTTTCCATAGTTTGTTTTCATTGTGGTCCCCCAA
ATCCCACACACATAACCCAACAGTGTGCCCTGCAAGATCCCTAGGATGCAGTCAGAGGGTGGCCTTCAGG
TCTAAACAGGGAGTCAAAATAGGCCCATGGAATTCTAAGTACATAGAGGCCCAAACAGCCCAATAAATAA
TGACTGTCTATGGCATTTGCCAGAACCCACAGATTAGGCCAGTCCAGGCCTGGTGGGAGTCTAAAAGTTT
AAGTTCAAGACACATAGGAAGATTAGTTTTAAAACGACTAATCGCTGTGATTTTGCTACCATAGATTAAT
ATACAAGTCGGTGCAACTCATCACCCTTTGTGTCTTCACTAGTGATGAGCGAATCTGAATCTGTTTATTC
GCTTCGCATAAAATTCGCAAAACGGCGAAAACGCATTTGACGCGCTTATATTTTTTTTGACGCGACTGCG
CCCTTTTACGCAATCGCGCCCGTTTTGACACGCAACAATTTTTTTTTCCGCAGTGAATTTTCACAGAAGT
TTCACGAAACAATACGCCAATGGCGAAATGTGGAAATTCGTTCTTCACTAGTCTTCACCCTATAAGTGAA
GACACAGAGCAGCTGGTCATCTTCCCACCCTCCAACTCTGTTTCCCTGCCTGACATCACTGTCTGCGTGG
AAAATATTAATATAATCCCAGTTCCACACGCTCGCTGTCTAGGAGTCACATTTGACTCTGCTCTCTTTCA

```
CTCCCCACATTCAAACACACTCTAAATCCTGTCGATTTCACCTCAAAAACATTGCATGCATTCGACCTCC
CCTCACTCATGAGACTGCCAAAATTCTCATCCACGCCCTTGTCATCTCGCTAAGACTACTGCAACTCACT
GCTCTGTGGGCTTCCCCTATCTAAACTGACCCCACTCCAATCCATCATGAACAGTGCTGCCAGACTCATA
CACCTCTCCTTCCGCTCCAAAAGTGCTGCCCCTCTCTGCCAATCCTTGCACTGGCTACCTGTCACGTACA
GGATTCAGTTCAAGATTCTTGCACTCGCCTTCAAAGCACTCACTGGTGCCGCACCGCTCTATTTAACCAC
CCTAATCCCCAAGTACACCCCCAGACGCAACCTTCGCTCAACGCATAACCTCCTTCTCTCCTCCTCACTC
ATCACTTCCTCTCATTCCCGCATCCAGGACTTCTCACGTGCTGCACCCATCCTCTGGAACGCCCTTCCCA
GTCCCATTAGGTATTGCCAGACTCTCCAAGCCTTTAAATGGTCTCTTAAAACTCACCTTTTCACTCAAAC
CTATAACCTAAACCCCCAAACATTCACTAACCACCAGTTTTCACCTCTCACTTACTGTCACTTTACACAC
CTACATGAACTCTAATGCCATGCTAGTTACCCTAACCTTATGTCTCACCCCTCCCTCTTAGAATGTAAGC
TTTGCGAGCAGGGCCCTCCCCCTAGTGTCTCCGATCCTCATCCAATGCAACTGCAAACCATTTTTGTGAA
CTGCTTATATGTACATGTTAACGGTTATGTCTTTTGTACCCCTCTATTCTGTAAAGCGCTGCGTAAATTG
ATGGCGCTATATAAATAATAATAATAGACAACTGTTTGTGTGTTTTAGCTGATGCAGTATTATCCGTAAA
TGATCAACATTGTCTATTTTTGTAGCCATGACAAGTAGCTGCTACTAGTAGCTCCGTGTGTCTTCACCCT
AAATCTGCCTCTGAAAGGGGTACTTGACCTTTTGGTTTGCTTGTATTGTGTTAGATTGTCCTGTTAGAAA
TGTACCCTATAGCTGAGAGGAAACAGATTGTGATGCTGTCTTTCCCTCCTTAGGCTGTGTGCTGCTCCGA
CCACTGGCACTGCTGCCCCCAGGGGTTTACCTGTGATGCCAGAGGTACCTGTGTACTAGGCCAGTTCTCT
ATCCCATGGCTCACTAAGGTTCCAGCTTTGCCCTTTGATGGGACTCACAGTATTTGTGACGATACCCACA
CTTGCCCTTCTGGTACCACATGCTGCCCTGGAGAGGGGGGAGGCTGGAGATGCTGCCCAGTGGAAGAGGT
CAGCAAAATGGTTAAACGAACTGTTATTTTATTGGCTACAGCTTGTATTGTAACCCTTGTATCTTTTAGC
TTTTGCCCTGATATTATTATTTATTATGCCTTGCAGAAGGAGGCTATCGAAACCTCCCCCAGTGACACCT
CTGTCCAGGGATTACGTCTGGATTATGTCTGGTGTGATTCTCAGTATGCCTGCTTTGATGGCCAAACTTG
TTGTCGAGGACTCGGAGGAGTGTGGAACTGCTGTGTTTATACTCAGGTGAGCCCCACTGCAAGCTGACAG
TTCTGTCATGGATATATGTGCCTATTGTAACTTAGAGCAACCTATACTGGCACTGTGTAGCAGAGAATGC
AGCTCTTATTCCACATGCAATGTCTTCCCTGCAGGGGGTGTGCTGCCCAGACATGGTGCACTGCTGTCCC
TATGGGTATGTGTGTCTGAACCATGGAACGTCATGTAGTCGGTCAGGAAGCCCACGTTGGGATGGTCAGT
CGGGAAGCCCACATTGGGATGGAAAACAGTCCCCACGGGATGGTAAACGGCCCCCTTTCCTATGAACACA
ATCCAAATCCACTTGATTGTTACTACTAAGATCCTTCTAAAAGCACTGGAATAAAATCAAATTATTTTTG
TTTTATATGTTTAAACTAATGAGTTTTTTTTTTGTGCTGGGGGGGGGGGGTGAAAGCTCACTTGCCTTTT
ATTTATTTCCTTTAAAAAGAGCCTCTGGGCCAAACGCTTGGGTTCTACTTGATATCCTAT
```

— Compiled, scripted, styled, and updated by R.G.E. Palfree —
