# Supplementary material for: The Evolution of the Secreted Regulatory Protein Progranulin
Source: PLoS One. 2015 Aug 6;10(8):e0133749. doi: 10.1371/journal.pone.0133749 (PMC4527844; doi:10.1371/journal.pone.0133749)
Supplement: S3 Table — Polypeptide sequences of granulin modules obtained from the genomes of premetazoan unicellular organisms including choanoflagellates, filasterea, apusozoa, amoebazoa and unicellular plants. (DOC) [file pone.0133749.s009.doc]

**Supplementary Table 3. The polypeptide sequences for the granulin modules of unicellular organisms** (intervening sequences between modules have been omitted).

| Organism and gene  (ID from NCBI databases unless otherwise stated) | Granulin module sequence |
| --- | --- |
|  |  |
| ***Choanoflagellate*** |  |
| MONBRDRAFT_28514.2  Monosiga brevicollis MX1: Supercontig null: 610394-611860  Progranulin | VTCGGGVAKCPTNNTCCKLSSDGYGCCPYLDGVCCKDGAHCCPANHTCNPSNGQCIS  DICPDHRSECSPEHTCCLTTDGSYGCCPFFDAQCCDNNHCCPEGNECAPDGTCNT  VVCPDKSQCAESTTCCSAPNGGYGCCPLQDANCCADQMHCCPQGTVCNATAGGCTR  QACSAEGTACPMNTTCCKLEYGAQACCPLPNATCCGDVEGHCCPGDRECDLTSMTCLS  ILCRLLDVNFVCPASAQCCADPRETLLRGTSADAALRAACCDSQGVAIADPWFM |
| MONBRDRAFT_11382.2  Monosiga. brevicollis MX1: Supercontig null: 461288-463285 +  GRN domain with lipase_GDSL_2 | VSQCPAGGSCCALADGHYGCCATSSGVCCAGSATCCPSGYTCHATDGSCLA |
| PTSG_05769.1  Salpingoeca. rosetta: Supercontig 13: 1541708-1542857 +  Progranulin | ERCSEGAKCPSHNTCCKMDGGRFGCCPYANATCCSNFCCPPNSQCAEDGRSCDTG  IVCPDGSYCPDFNTCCQIPGGYGCCSLSDGVCCPDGAFCCPSGMTCGQRSCYAE |
| PTSG_09523.1  Salpingoeca. Rosetta: Supercontig 26: 229626-232592 +  GRN domain with lipase-like protein | CTGTSMYNATFCNDNSTCCPYKWSPNGYGCCTMPNAVCCSNGYTCCPEGTECRDSGTSWDVITSCV |
| PTSG_10760.1  Salpingoeca. rosetta: Supercontig 34: 463086-465653 –  GRN domain with lipase-like protein | ITCPSHNTSTTADALECPSGSTCCDNGCCPSSHAVCCHDGKHCCAHGYHCAGSRCEA |
| ***Filasterea*** |  |
| CAOG_01098.2  Capsaspora owczarzaki ATCC 30864: Supercontig 1: 3524252-3525990 –  Progranulin | ALCPDGITSCPSSSTCCVTASGWYGCCPLPSATCCADHVHCCPNGYICNTTQSICTP  IECGDGITYCSSSQTCCLLSGGSYGCCPLPNATCCADHVHCCPNGYTCDTTLNTCKL  VVCPDNSQCSATQTCCKMSNGSYGCCPLPNASCCADHLHCCPSGFTCDVTDHTCNN |
|  |  |
| ***Amoebazoa***  Thesequences marked by * were obtained from NCBI nucleotide databases searched by Blast-X using the the Dictyostelium discoideum sequence. These Grn-module containing genes lack discrete NCBI ID accession numbers:- Incomplete modules indicated by dashed lines |  |
| Dictyostelium discoideum AX4  XP_638956 | kcpdgslcpnsntccsasdgsyaccptpnaqccsdkqhccpyqftcgnggnick |
| Dictyostelium fasciculatum EGG24629 | qcqdgsfcpagntccpsanagysccpannavcckdlqhccpqnfscssggkici |
| Dictyostelium purpureum  XP_003290312 | kcndgsecpalntcclisdgsyaccptpngvccnnnhcctqgfhcgsggnici |
| Polysphondylium pallidum  EFA85154 | ecpdgsycdsgstccpsgnggysccpsagasccsdfkhccpagftcnrsdkkhD |
| *Dictyostelium intermedium strain PJ-11 Contig2565, whole genome shotgun sequence ID: gb|AJWI01002565.1 | VKCPDGSLCPNSNTCCPASNDSYACCPTPNAQCCSDKQHCCPYQFQCGNGGNICKP |
| *Polysphondylium violaceum strain QSvi11 Contig231, whole genome shotgun sequence ID: gb|AJWJ01000116.1 | KCGDGSVCPSDNTCCLVQGGVYACCPTPQGTCCSDSPNQQSQSNSQHCCPQGFSCGSTGQICI |
| *Dictyostelium firmibasis strain TNS-C-0014 Contig92, whole genome shotgun sequence ID: gb|AJWH01000092.1 | …………………………………………………………………. AQCCSDKQHCCPYQFSCGNGGNICKP |
| Hartmannella vermiformis  Taxonomically Broad  EST database:  Cluster HVL00000901 | .....ELSDGQYGCCPYPQAVCCSDHSSCCPNGYTCDVQKSQCV  SCADGSSCPSTDTCCQLQSGEYGCCPYPQAVCCSDKESCCPNGYTCDLTNKQCV  NCADGSQCNDGSTCCQLQSGGYGCCPYVNAVCCSDKQSCCPNGYTCDLQNKQCD |
| ***Apusozoa*** |  |
|  |  |
| AMSG_08884.2 Broad Institute Origins of Multicellularity database  Thecamonas trahens ATCC 50062: Supercontig 49: 125998-129421  bacterial permeability-increasing protein domain and GRN module containing protein | APCGGAPGGVCAVHNVCCSWGCCATDSGVCCPTSGLCCPAGWICTSTGCSQ |
|  |  |
| Unicellular green plants |  |
| Chlamydomonas reinhardtii  XP_001700766.1 | MCDMFGWTECGVGNTCSCSFSLFGWLCLWHDCCPLADAVSCPDLKHCCPAGTTCNAAQGACIA |
| Chlamydomonas reinhardtii  XP_001697622.1 | VKCDDDNECPNGSTCCCVNEIFNMCFQWGCCPMPKATCCDDHEHCCPADLPVCDTDAGRCLPSAG |
| Volvox carteri f. nagariensis  XP_002947177.1 | ICDIFGWTECPVGNSCSCSFSFFGFLCLWHDCCPLAGGVTCPDLKHCCPSGTNCDQRQGVCVS |
| Volvox carteri f. nagariensis  XP_002954352.1 | VKCDDDNECPAGSTCCCVMEFFNMCFQWGCCPMPKATCCSDNQHCCPADLPVCDTVGGRCLPAG |
| Chlorella variabilis  EFN58681.1 | CDDTTQCPPDNTCCCMREFFGFCFTWACCPLPKATCCDDQQHCCPEDLPVCDTVAGRCLA |
| Coccomyxa subellipsoidea C-169  EIE26881.1 | CDTATSCPPASTCCCMREFFGYCFTWACCPLKEATCCDDHEHCCPSNLPVCDTVAGRCLS |
| Asterochloris sp  e_gw1.00063.81.1*  (DOE Joint Genome Institute) | CDQTGKTQCPNGSTCCCMRDFFGFCFTWACCPLPEATCCNDHQHCCPSNLPVCDTTAGRCL |
