# Supplementary material for: The Evolution of the Secreted Regulatory Protein Progranulin
Source: PLoS One. 2015 Aug 6;10(8):e0133749. doi: 10.1371/journal.pone.0133749 (PMC4527844; doi:10.1371/journal.pone.0133749)
Supplement: S4 Table — Schematic representations of granulin protein and gene structures from a representative selection of fish. (DOC) [file pone.0133749.s010.doc]

Supplementary table 4. The protein and genomic structure of Grn genes in a representative selection of fish families. Symbols: g, canonical 12 cysteine granulin module; g*, 10 cysteine granulin module; p, six cysteine N-half granulin module (paragranulin); q, six cysteine C-terminal half granulin module; x, non-granulin sequences; sn, exon encoding signal peptide and N-terminal half granulin; n, exon encoding N-terminal half granulin; c exons encoding C-terminal half granulin; cn exon encoding a C-terminal half granulin module, a spacer and an N-terminal half granulin module. Other special notations for particular cases are explained in the table.

| **Teleostei, Ostariophysi** | | | | |
| --- | --- | --- | --- | --- |
| **Species** | **name** | **Progranulin Form** | **Exon Structure for granulin modules** | **Data note** |
| **Danio rerio**  (zebrafish) | D_rerA | gg*gggggggg | sn-c-n*-c*-n-c--n-c-n-c-n-c-n-cn-cn-cn-c | See Table S1 |
|  | D_rerB | ggggggggg | sn-c-n-c-n-c-n-cn-cn-cn-cn-cn-c | See Table S1 |
|  | D_rer1,2 | gp | sn-c-n | See Table S1 |
| **Cyprinus carpio**  (common carp) | C_carA | gg*gggggggg | sn-c-n*-c*-n-c--n-c-n-c-n-c-n-cn-cn-cn-c | Genomic GI:685042152, LG7, no EST data |
|  | C_carB | gggggggggggg | sn-c-n-c-n-c-n-c-n-c-n-c-n-c-n-c-n-cn-cn-cn-c | Genomic GI:685042184, LG5, no EST data |
|  | C_car1 | gp | sn-c-n  (cDNA and genomic DNA show at least 5 variations in sequence) | Abundant EST data. Genomic in GI:685042563 & GI:685042152. |
| **Astyanax mexicanus**  (Mexican tetra) | A_mexA | gg*g(g)ggggg | sn-c-n*-c*-n-c-(n)-c-n-c-n-cn-cn-cn-c | Genomic in GI:597754467. Incomplete. No EST data. |
|  | A_mexB | ggggggggg | sn-c-n-c-n-c-n-cn-cn-cn-cn-cn-c | Genomic in GI:594227355. No EST data for coding region. |
|  | small form (tentative) | ggg(g?)g | sn-cn-c-n-c-(?)-n-c | Incomplete genomic prediction GI:597794435 & GI:597794433. EST entries (5) may span the gene. Uncertain repeat number. |
| **Carassius auratus**  (goldfish) | C_aur1, etc. | gp | No genomic data | cDNA as GI:86558937. TSA sequence GI:749632732. Several similar in <20 EST sequences. |
|  | C_aurB1 | ggggggg | No genomic data | TSA sequence GI:749618242 |
|  | C_aurB2 | (?)gggg(?) | No genomic data | TSA sequence GI:749614252. Incomplete both ends. Might be an A form, but more similar to C_aurB1 than to C_carA or B. |
|  | C_aurB3 | (?)ggg | No genomic data | TSA sequence GI:749624452. Last 3 modules and 3'ut. Might be an A frm, but more similarity with C_carB than C_carA. |
| **Pimephales promelas**  (fathead minnow) | P_proA | gg*ggggggggg | sn-c-n*-c*-n-c-n-(c)-n-c-n-(c)-n-c-n-cn-cn-cn-c | Genomic in GI:650755918 with gaps. |
|  | P_proB | ggggggggg | sn-c-n-c-n-c-n-cn-cn-cn-cn-cn-c | Genomic starts in GI:651191275, ends in GI:650985677, with gaps. All but the last 3 modules covered by EST data. |
|  | P_pro1 | gp | sn-c-n | Transcript defined by EST data. Genomic in GI:651002570 |

| **Teleostei, Euteleostei, Protacanthopterygii** | | | | |
| --- | --- | --- | --- | --- |
| **Species** | **name** | **Progranulin Form** | **Exon Structure for granulin modules** | **Data note** |
| Salmo salar  (Atlantic salmon) | S_salA | gg*gggggggg | sn-c-n*-c*-n-c-n-cn-c-n-cn-cn-cn-cn-c | See Table S1 |
|  | S_salB | gggggggggg | sn-c-n-c-n-c-n-c-n-cn-cn-cn-cn-cn-c | See Table S1 |
|  | S_salC | ggg | sn-cn-c-n-c | See Table S1 |
|  | S_sal1 | pg | sn-n-c | See Table S1 |
| Oncorhynchus tshawytscha  (chinook salmon) | O_tsh1 | pg |  | EST data |
|  |  |  |  | Database not recently searched for additions. |
| Oncorhynchus mykiss  (rainbow trout) | O_myk1 | pg |  | EST data |
|  | O_mykC | ggg |  | EST data |
|  |  |  |  | Database not recently searched for additions. |
| Salvelinus fontinalis  (brook trout) | S_fon1 | pg |  | EST data |
|  |  |  |  | Database not recently searched for additions. |

| **Teleostei, Euteleostei, Neoteleostei, Acanthomorpha, Acanthopterygii** | | | | |
| --- | --- | --- | --- | --- |
| **Species** | **name** | **Progranulin Form** | **Exon Structure for granulin modules** | **Data note** |
| Gasterostus aculeatus  (stickelback) | G_acuA | gg*gggggg | sn-c-n*-c*-n-c-n-c*n-cn-cn-cn-c | See Table S1 |
|  | G_acuB | ggggggggg | sn-c-n-c-n-c-n-c-n-cn-cn-cn-cn-c | See Table S1 |
|  | G_acuC | ggg | sn-cn-c-n-c | See Table S1 |
| Oreochromis niloticus  (Nile tilapia) | O_nilA | gg*gggggg | sn-c-n*-c*-n-c-n-cn-cn-cn-cn-c | See Table S1 |
|  | O_nilB | gggggggggg | sn-c-n-c-n-c-n-c-n-cn-cn-cn-cn-cn-c | See Table S1 |
|  | O_nilC1 | gg | sn-c-n-c | See Table S1 |
|  | O_nilC2,3,4 | gg | sn-cn-c | See Table S1 |
| Takifugu rubripes  (Japanese puffer) | T_rubB | gggggggg | sn-cn-c-n-c-n-cn-cn-cn-cn-c | See Table S1 |
|  | T_rubC | ggg | sn-cn-c-n-c | See Table S1 |
|  | T_rubD | q*gg | sx-c*-n-cn-c | See Table S1. Related to the A form. |
| Tetraodon nigroviridis  (green spotted puffer) | T_nigB | gggggggg | sn-cn-c-n-c-n-cn-cn-cn-cn-c | Genomic GI:47217605 |
|  | T_nigC | ggg | sn-cn-c-n-c | Ample EST data. Genomic in GI:47222198 |
|  | T_nigD | q*gg | sx-c*-n-cn-c | GI:56309590 and EST data. Genomic in GI:47217921 |
| Paralabidochromis chilotes (Victoria big-lipped Hap) | P_chiC | gg |  | Abundant EST data. |
| Rhamphochromis esox (Tigerfish) | R_esoC | gg | sn-cn-c | Genomic in GI:192381634 & GI:191618541 |

| **Teleostei, Euteleostei, Neoteleostei, Acanthomorpha, Paracanthopterygii** | | | | |
| --- | --- | --- | --- | --- |
| **Species** | **name** | **Progranulin Form** | **Exon Structure for granulin modules** | **Data note** |
| Gadus morhua  (Atlantic cod) | G_morC | gpp (gpg but for a stop codon) |  | Abundant EST data for this small form. See Table S1. |
|  | large forms |  | Insufficient genomic data as yet. | No cDNA data for large forms |

| **Chondrichthyes** | | | | |
| --- | --- | --- | --- | --- |
| **Species** | **name** | **Progranulin Form** | **Exon Structure for granulin modules** | **Data note** |
| Callorhinchus milii  (elephantfish) | C_mil1 | gqgggggggg | sn-c-c-n-c-n-cn-cn-cn-cn-cn-cn-c | See Table S1 |
|  | C_mil2 | ggg'g'g'gg | sn-cn-cn-c'n-c'n-c'n-cn-c  (c' encodes a C-half lacking the last 2 single Cys of the normal motif) | See Table S1 |

| **Hyperoartia & Hyperotreti** | | | | |
| --- | --- | --- | --- | --- |
| **Species** | **name** | **Progranulin Form** | **Exon Structure for granulin modules** | **Data note** |
| Petromyzon marinus  (sea lamprey) | P_marL | gggggggggg | sn-c-n-cn-cn-cn-cn-cn-cn-cn-cn-c | See Table S1 |
|  | P_marS1 | pg”xg | sn-n”-c-xn-c  (n" encodes a 4 Cys N-half: C-C-CC) | See Table S1 |
|  | P_marS2 | pg” | sn-n"-c | See Table S1 |
|  | P_marS3 | pg”x | sn-n"-c-x | See Table S1 |
|  | P_marS4 | p-g”-x-ğ | sn-n”-c-xň-c  (ň encodes an N-half similar to that in plants) | See Table S1 |
| Eptatretus burgeri  (inshore hagfish) | E_bur1 | ggg+g | (g+ denotes a module with an unusual N-half Cys arrangement CXC-C-CXCC) | From a 10 EST contig. |
