# Supplementary material for: The Evolution of the Secreted Regulatory Protein Progranulin
Source: PLoS One. 2015 Aug 6;10(8):e0133749. doi: 10.1371/journal.pone.0133749 (PMC4527844; doi:10.1371/journal.pone.0133749)
Supplement: S5 Table — (DOCX) [file pone.0133749.s011.docx]

**Table 5: The genomic environment immediately flanking human *GRN* and coelacanth *GrnA (L_ChaA)***

| **Human Chromosome 17** | **Coelacanth scaffold Ensembl scaffold JH126680.1** |
| --- | --- |
|  |  |
| ITGA3 | ITGA3 |
| PDK2 | PDK2 |
| SAMD14 | SAMD14 |
| PPIR9B | PPIR9B |
| SGCA | SGCA |
| COL1A1 | COL1A1 |
|  |  |
| Gap Approximately 6300kb |  |
|  | MLL |
|  | RETSAT |
| MPP2 | MPP2 |
| FAM215A |  |
| LLRC37A10P |  |
| PPY |  |
| PYY | PYY |
| TMEM101 | TMEM101 |
| NAGS | NAGS |
| LSM12 | LSM12 |
| G6PC3 |  |
| HDAC5 | HDAC5 |
| C170RF53 |  |
| ASB16 | ASB16 |
|  | TMUMB |
| UBTF | UBTF |
| SHC1P2 |  |
| SLC4A1 | SLC4A1 |
| RUND3CA | RUND3CA |
| SLC25A39 | SLC25A39 |
| **GRN** | **GRN (*L_ChaA*)** |
| FAM171A2 | FAM171A2 |
| ITGA2 |  |
| GPATCH | GPATCH |
| FZD2 | FZD2 |
|  |  |

Human *GRN* (highlighted in bold) and coelacanth *GrnA* on Ensmbl scaffold JH126680.1 show synteny (gene abbreviations are as given in NCBI). The human genes fall into two groups on chromosome 17 separated by a large gap.
