# Supplementary material for: The Evolution of the Secreted Regulatory Protein Progranulin
Source: PLoS One. 2015 Aug 6;10(8):e0133749. doi: 10.1371/journal.pone.0133749 (PMC4527844; doi:10.1371/journal.pone.0133749)
Supplement: S6 Table — Genes immediately flanking the short-form Grn genes of Danio rerio (D_rer1 and D_rer2 in Figs 5 and 6) were subjected to synteny analysis against the human genome using the Syntenty Database program. 40 of 46 Danio genes flanking Grn1 and Grn2 have orthologues in the human genome in two major synteny groups. Short-form Grns were probably lost from the tetrapod lineage in a discrete event rather than as a block deletion of multiple genes. (DOC) [file pone.0133749.s012.doc]

**Supplementary Table 6. Loss of the Grn C (short-form) Grn gene in tetrapods.** Conserved gene position around zebrafish short-form *Grn*-genes *Grn1* and *Grn2* (*D_rer1* and *D_rer2* in fig 5 and 6, bold in table) and orthologous genes on human chromosomes indicate that the short form GrnC genes of fish were lost in the tetrapod line by a discrete genetic event rather than large scale deletion of a block of genes. Synteny groups were assigned using the Synteny Database.

| **Danio Chromosome 19 GENE** | **Synteny groups** | **Human chromosomal assignment** |
| --- | --- | --- |
|  |  |  |
| [angpt2](http://www.ncbi.nlm.nih.gov/entrez/query.fcgi?db=gene&cmd=retrieve&dopt=full_report&list_uids=114408) | 278899 | **h8** |
| [csmd2](http://www.ncbi.nlm.nih.gov/entrez/query.fcgi?db=gene&cmd=retrieve&dopt=full_report&list_uids=565460) | 280144 | **h1** |
| [cx30.9](http://www.ncbi.nlm.nih.gov/entrez/query.fcgi?db=gene&cmd=retrieve&dopt=full_report&list_uids=402821) | 280143 | **h1** |
| Pef1 [zgc:100787](http://www.ncbi.nlm.nih.gov/entrez/query.fcgi?db=gene&cmd=retrieve&dopt=full_report&list_uids=445249) | 280143 | **h1** |
| zgc:103571 C1orf212 homolog | 280143 | **h1** |
| [cx39.4](http://www.ncbi.nlm.nih.gov/entrez/query.fcgi?db=gene&cmd=retrieve&dopt=full_report&list_uids=557275) (connexin 39.4) | not found |  |
| [dlgap3](http://www.ncbi.nlm.nih.gov/entrez/query.fcgi?db=gene&cmd=retrieve&dopt=full_report&list_uids=557556) | 280144 | **h1** |
| [ilf2](http://www.ncbi.nlm.nih.gov/entrez/query.fcgi?db=gene&cmd=retrieve&dopt=full_report&list_uids=406517) | 27983 | **h1** |
| [si:ch211-250g4.3](http://www.ncbi.nlm.nih.gov/entrez/query.fcgi?db=gene&cmd=retrieve&dopt=full_report&list_uids=557772) | not found |  |
| [thsd7a](http://www.ncbi.nlm.nih.gov/entrez/query.fcgi?db=gene&cmd=retrieve&dopt=full_report&list_uids=557991) | 280319 | **h7** |
| [nxph1](http://www.ncbi.nlm.nih.gov/entrez/query.fcgi?db=gene&cmd=retrieve&dopt=full_report&list_uids=437006) | 280319 | **h7** |
| [phf14](http://www.ncbi.nlm.nih.gov/entrez/query.fcgi?db=gene&cmd=retrieve&dopt=full_report&list_uids=555505) | 280320 | **h7** |
| [smap2](http://www.ncbi.nlm.nih.gov/entrez/query.fcgi?db=gene&cmd=retrieve&dopt=full_report&list_uids=556132) | 280144 | **h1** |
| [col9a2](http://www.ncbi.nlm.nih.gov/entrez/query.fcgi?db=gene&cmd=retrieve&dopt=full_report&list_uids=321212) | 280143 | **h1** |
| LOC100535782  collagen alpha-1(XIX) chain-like |  |  |
| [bai2](http://www.ncbi.nlm.nih.gov/entrez/query.fcgi?db=gene&cmd=retrieve&dopt=full_report&list_uids=556993) | 280144 | **h1** |
| LOC100536444  (XP_003200675.1) | Not found |  |
| [sfpq](http://www.ncbi.nlm.nih.gov/entrez/query.fcgi?db=gene&cmd=retrieve&dopt=full_report&list_uids=406564) | 280143 | **h1** |
| ZMYM4 [si:ch211-173p18.1](http://www.ncbi.nlm.nih.gov/entrez/query.fcgi?db=gene&cmd=retrieve&dopt=full_report&list_uids=327248) zinc finger protein 262 | 280143 | **h1** |
| si:ch211-173p18.2 | not found |  |
| ZMYM4 si:ch211-173p18.3 | 280143 | **h1** |
| illr4 | 281417 | **h12** |
| Rbm48 [dkfzp564o0523](http://www.ncbi.nlm.nih.gov/entrez/query.fcgi?db=gene&cmd=retrieve&dopt=full_report&list_uids=402897) | 280845 | **h7** |
| efcab1[si:ch211-173p18.9](http://www.ncbi.nlm.nih.gov/entrez/query.fcgi?db=gene&cmd=retrieve&dopt=full_report&list_uids=558421) | 280177 | **h8** |
| **GRN1 (*D_rer1*)** |  |  |
| **GRN2 (*D_rer2*)** |  |  |
| Cdk6 [si:ch211-234f14.1](http://www.ncbi.nlm.nih.gov/entrez/query.fcgi?db=gene&cmd=retrieve&dopt=full_report&list_uids=100034507) | 285297 | **h7** |
| Fam133b [si:ch211-287b5.3](http://www.ncbi.nlm.nih.gov/entrez/query.fcgi?db=gene&cmd=retrieve&dopt=full_report&list_uids=790942) | 285297 | **h7** |
| Hepacam2 LOC558612 |  | **h7** |
| ccd132 isoform a [si:ch211-287b5.1](http://www.ncbi.nlm.nih.gov/entrez/query.fcgi?db=gene&cmd=retrieve&dopt=full_report&list_uids=100034394) |  | **h7** |
| calcr | 285297 | **H7 (7q21.3)** |
| [mir489](http://www.ncbi.nlm.nih.gov/entrez/query.fcgi?db=gene&cmd=retrieve&dopt=full_report&list_uids=100033722) | not found |  |
| [tfpi2](http://www.ncbi.nlm.nih.gov/entrez/query.fcgi?db=gene&cmd=retrieve&dopt=full_report&list_uids=560339) | 285297 | **h7** |
| [gngt1](http://www.ncbi.nlm.nih.gov/entrez/query.fcgi?db=gene&cmd=retrieve&dopt=full_report&list_uids=335656) | 287265 | **h7** |
| bet1 | 285297 | **h7** |
| [col1a2](http://www.ncbi.nlm.nih.gov/entrez/query.fcgi?db=gene&cmd=retrieve&dopt=full_report&list_uids=336471) | 285297 | **h7** |
| [casd1](http://www.ncbi.nlm.nih.gov/entrez/query.fcgi?db=gene&cmd=retrieve&dopt=full_report&list_uids=692268) | 285297 | **h7** |
| [sgce](http://www.ncbi.nlm.nih.gov/entrez/query.fcgi?db=gene&cmd=retrieve&dopt=full_report&list_uids=368230) | 285297 | **h7** |
| [ppp1r9a](http://www.ncbi.nlm.nih.gov/entrez/query.fcgi?db=gene&cmd=retrieve&dopt=full_report&list_uids=560862) | 285297 | **h7** |
| [asb4](http://www.ncbi.nlm.nih.gov/entrez/query.fcgi?db=gene&cmd=retrieve&dopt=full_report&list_uids=550321) | 285297 | **h7** |
| [si:rp71-57j15.4](http://www.ncbi.nlm.nih.gov/entrez/query.fcgi?db=gene&cmd=retrieve&dopt=full_report&list_uids=561007) | 287777 | **h17** |
| [shfm1](http://www.ncbi.nlm.nih.gov/entrez/query.fcgi?db=gene&cmd=retrieve&dopt=full_report&list_uids=322269) | 285297 | **h7** |
| [dlx6a](http://www.ncbi.nlm.nih.gov/entrez/query.fcgi?db=gene&cmd=retrieve&dopt=full_report&list_uids=30586) | 285297 | **h7** |
| [dlx5a](http://www.ncbi.nlm.nih.gov/entrez/query.fcgi?db=gene&cmd=retrieve&dopt=full_report&list_uids=30569) | 285297 | **h7** |
| [zgc:110753](http://www.ncbi.nlm.nih.gov/entrez/query.fcgi?db=gene&cmd=retrieve&dopt=full_report&list_uids=503606) | 286447 | **h8** |
| [wu:fb99g09](http://www.ncbi.nlm.nih.gov/entrez/query.fcgi?db=gene&cmd=retrieve&dopt=full_report&list_uids=799532) | 284395 | **h1** |
| [si:dkeyp-66g8.1](http://www.ncbi.nlm.nih.gov/entrez/query.fcgi?db=gene&cmd=retrieve&dopt=full_report&list_uids=562045) | 279835 | **h1** |
| [tinagl1](http://www.ncbi.nlm.nih.gov/entrez/query.fcgi?db=gene&cmd=retrieve&dopt=full_report&list_uids=562116) | 280144 | **h1** |
|  |  |  |
