# Supplementary material for: The Evolution of the Secreted Regulatory Protein Progranulin
Source: PLoS One. 2015 Aug 6;10(8):e0133749. doi: 10.1371/journal.pone.0133749 (PMC4527844; doi:10.1371/journal.pone.0133749)
Supplement: S1 Text — (DOCX) [file pone.0133749.s013.docx]

**Supplementary text 1**

***Bikonts other than Viridiplantae* *lack genes encoding easily identifiable granulin modules*.**

Searching by BLASTx using the the *Dictyostelium* sequence as the query identified a possible *Grn* sequence in downy mildew *Pseudoperonospora cubensis* psc_contig_26682, whole genome shotgun sequence, GenBank: AHJF01024289.1 with an open reading frame that encodes a granulin module. BLASTp analysis of the predicted translated sequence gave 89% identity with a cysteine protease gb|ABK24495.1 from *Picea sitchensis* (Spruce tree). We conclude therefore that the sequence in *Ps cubensis* resulted from contamination with plant DNA.

Searching BLASTn with the *Capsaspora* Grn nucleotide sequence using the “somewhat dissimilar” setting detected a putative transcript, GenBank: GAGR01016315.1 in the transcriptome shotgun assembly of *Nannochloropsis gaditana*, a heterokont alga. Translation provides a potential open reading frame with at least two granulin modules. We have not identified similar genes in any other heterokonts and in the absence of supporting data believe it may be a contaminant.
