# Supplementary material for: The Evolution of the Secreted Regulatory Protein Progranulin
Source: PLoS One. 2015 Aug 6;10(8):e0133749. doi: 10.1371/journal.pone.0133749 (PMC4527844; doi:10.1371/journal.pone.0133749)
Supplement: S2 Text — (DOCX) [file pone.0133749.s014.docx]

***Supplementary Text 2. Variations on the granulin motif***

Phylogenetic analysis revealed the presence of variants of the granulin module motif. These variants may provide insight into the structural constraints that underlie the granulin fold and the extent to which it can be modified. By far the most common granulin module structure contains 12 cysteines aligned as in fig 1 of the main text (“normal motif” in the table below). Progranulins that belong to the GrnA subgroup start with an amino terminal half module of 6 cysteines, called paragranulin, followed by a 10-cyteine module. Less frequent variants occur some of which are discussed below.

Table showing the variations on the normal Cysteine motifs.

| **Found in** |  | **Motif of resulting module (N-half / C-half)** | **Comment** |
| --- | --- | --- | --- |
| Normal motif | n/c | **C――C――CC――CC- / -CC――CC――C――C** |  |
| Common 10 Cys | n*/c* | **C――C――C.――CC- / -.C――CC――C――C** |  |
| Elephant Shark C_mil2 | n/c' | **C――C――CC――CC- / -CC――CC――.――** . | mod 3,4,5 (rpt) |
| Sea anemone N_vec | n/c” | **C――C――CC――CC- / -CC-** (deletion) | mod 9 |
| Lamprey P_marS1,2,3,4 | n”/c | **C――C―**del **―CC- / -CC――CC――C――C** | mod 1 |
| Lamprey P_marS4 | n'/c | **C――C――CCC―CC- / -CC――CC――C――C** | mod 2 |

**Notes:** A dot stands for some other residue than C. Abbreviations: “mod” = module (the number gives the position in the sequence of full modules); “rpt” = belonging to a repeat of nearly identical sequences; “del” = deletion. Cysteines are schematically aligned, but spacing does vary in modules. The / indicates the position of the intron that separates N and C terminal sequences in the corresponding nucleotide sequences. The notation for half-module variants is used to draw attention to differences but it may mean different forms of variant when used in different contexts in the supplementary sequence data document.

The disulphide bridging pattern for the normal motif is well characterized (Hrabal et al, 1996 , Tolkatchev et al, 2008):-

C_1_—C_2_—C_3_C_4_—C_5_C_6_- / -C_7_C_8_—C_9_C_10_—C_11_—C_12_

In the common 10 Cys module (n*/c*), which occurs as the first full module of all genes in the GrnA synteny group, one disulfide bond, C_4_C_7_, is lost:-

C_1_—C_2_—C_3_ x—C_5_C_6_- / - x C_8_—C_9_C_10_—C_11_—C_12_

The missing two cysteines in the variant found in 3 modules in *C. Milii* progranulin 2 and four cysteines missing in one module of the *N. vectensis* progranulin would have more effect on the structure. Either there would be two free sulfhydryl groups (C_10_ and C_8_ for *C_mil2* and C_6_ and C_8_ for *N Vec*) with the possibility of their disulfide bonding with polypeptide outside the module or there would be rearrangement of the disulfide bonding within the variant module. For the elephant shark C_mil2 rearrangement of the disulphide bridging with the maximum retention of the normal motif could be postulated as :-

C_1_—C_2_—C_3_C_4_—C_5_C_6_- / -C_7_C_8_—C_9_C_10_

where C_10_C_12_ is lost and there is rearrangement of C_8_C_11_ to C_8_C_10._ For the sea anemone *N_vec* internal bridging with maximum retention of the normal motif can be hypothesized as:-

C_1_—C_2_—C_3_C_4_—C_5_C_6_- / -C_7_C_8_

where there is loss of C_8_C_11_, C_10_C_12_ and rearrangement of C_6_C_9_ to C_6_C_8_.

The lamprey (*Petromyzon marinus*) *Grn* genes show the greatest diversity among module variants. In this case it is the N-half which is affected. The long form progranulin (*P_marL*) was included in the analysis for figures 3, 4 & 5 in the manuscript. Four short forms also were found in this species (*P_marS1, S2, S3, S4*). They are included only in the table 1 of the manuscript, as their peculiarities required separate consideration. At the beginning of the lamprey small forms is a paragranulin. It is followed by a shortened module lacking a stretch of residues which include the first double Cys. This is shown below in an alignment with the normal granulin of P_marS1.

P_marS1_01 TS**C**-AGSV**C**--------SANGESR**CC**PLSEGS**CC**GDGLS**CC**GKGST**C**TTFRGLNL**C**LP

P_marS2_01 TS**C**-AGSV**C**--------SANGESR**CC**PLSEGS**CC**GDGLS**CC**GKGST**C**TTFQGLNV**C**LP

P_marS3_01 RS**C**-TGSV**C**--------SANGESR**CC**PLSEGS**CC**GDGKS**CC**GKGTT**C**TMYGGVNL**C**LP

P_marS4_01 ID**C**-SGPI**C**--------LHSGEPL**CC**PAPAGV**CC**TDGRA**CC**AANNT**C**ITVEDMHV**C**YP

P_marS1_02 VY**C**GSGQY**C**RDGQT**CC**RLATGSWG**CC**NIPHAI**CC**SDGIH**CC**PAGHF**C**LTASGL--**C**AR

Because of the deletion, the N-half can not be compared with others in phylogenetic trees. When the C-half DNA sequences were included in the phylogenetic analysis, they were grouped together and placed in Csub01 of the manuscript figure 4.

This kind of module has been found also in platyhelminths *Hymenolepis microstoma* and *Echinococcus granulosus*, in which they occur within a string of normal modules. In these sequences the variants are modules 2 and 6, and they are shown aligned with the lamprey modules below.

H. microstoma 2 KS**C**---LST**C**---GD-L**CC**PFPKGV**CC**EDGEH**CC**PAEYK**C**DV--TTRS**C**RL

H. microstoma 6 SK**C**RPDWTS**C**SANGRTG**CC**PLKDAV**CC**SDGLH**CC**LKGST**C**LD---NGT**C**LV

E. granulosus 2 ES**C**-P-AT-**C**---GD-L**CC**PFEGGV**CC**NDGEH**CC**PPGYE**C**DI--LTKS**C**RL

E. granulosus 6 GA**C**FPKATP**C**SGNGKTG**CC**PLENAV**CC**SDGLH**CC**PKDSV**C**TA---SGW**C**LM

P_marS1_01 TS**C**--AGSV**C**SANGESR**CC**PLSEGS**CC**GDGLS**CC**GKGST**C**TTFRGLNL**C**LP

P_marS2_01 TS**C**--AGSV**C**SANGESR**CC**PLSEGS**CC**GDGLS**CC**GKGST**C**TTFQGLNV**C**LP

P_marS3_01 RS**C**--TGSV**C**SANGESR**CC**PLSEGS**CC**GDGKS**CC**GKGTT**C**TMYGGVNL**C**LP

P_marS4_01 ID**C**--SGPI**C**LHSGEPL**CC**PAPAGV**CC**TDGRA**CC**AANNT**C**ITVEDMHV**C**YP

The alignment above includes data from *H. microstoma* GI:674590324, to which we added sequence from an overlooked exon, and *E. granulosus* GI: 674561510.

Considering that this type of module is found in multiple copies, in several genes, and in at least both lamprey and platyhelminth, it is most likely they have acquired useful functions, and possibly functions which are related to those of some normal modules, but biased toward the role of the C-half of the module. Three possible disulphide arrays may be hypothesized if, to maximize retention of the normal module structure, all the disulphide bridges in the C-terminal half are aligned as they are in the normal 12 cysteine motif.

C_1_—C_2_— del —C_5_C_6_- / -C_7_C_8_—C_9_C_10_—C_11_—C_12_

Here disulphide bridges C_1_C_3_, C_4_C_7_ are lost, there is rearrangement of C_1_C_3_ and C_4_C_7_ to

C_1_C_7_ and 4 out of the 5 disulphde bonds are the same as in the normal motif.

C_1_—C_2_— del —C_5_C_6_- / -C_7_C_8_—C_9_C_10_—C_11_—C_12_

Here there is loss of C_1_C_3_, C_4_C_7_, rearrangement of C_1_C_3_ to C_1_C_2_, and C_4_C_7_ to

C_5_C_7_ and 3 out of 5 disulphide bonds are the same as in the normal motif.

C_1_—C_2_— del —C_5_C_6_- / -C_7_C_8_—C_9_C_10_—C_11_—C_12_

Here there is loss of C_1_C_3_, C_2_C_5_, and C_4_C_7_, rearrangement of C_1_C_3_ to C_1_C_5_, and C_2_C_5_ and C_4_C_7_ to C_2_C_7_, and 3 out the 5 disulphide bridges are the same as in the normal motif.

P_marS1 has one normal granulin module and P_marS4 has an almost normal module, distinguished by a triple Cys in place of the first double Cys of the motif. They are shown below aligned with the fourth module of coelacanth progranulin C.

P_marS1_02 VY**C**GSGQY**C**RDGQT**CC**RLATGSWG**CC**NIPHAI**CC**SDGIH**CC**PAGHF**C**LTASGL**C**AR

VY**C**G+ Y**C** DG T**CC**+L +GSWG**CC** PHAI**CC** DG H**CC**P G+F**C** S **C** +

L_chaC_04 VY**C**GNQYY**C**PDGNT**CC**KLPSGSWG**CC**PHPHAI**CC**RDGYH**CC**PYGYF**C**DFTSTK**C**TK

V **C** N Y**C**P T**CC** LP GSWG**CC** P A+**CC** DG H**CC**P G+ **C** K

P_marS4_02 VS**C**ANRRY**C**PGDST**CCC**LPAGSWG**CC**GVPNAV**CC**ADGVH**CC**PAGHV**C**M--EKY**C**MK

The triple Cys in P_marS4_02 is reminiscent of the granulin motif variation found in plants. To show the similarity, it is aligned below with the module from *Populus euphratica,* the Euphrates poplar tree.

Poplar SD**C**GDFSY**C**PSDET**CCC**ILKVFDY**C**LVYG**CC**AYENAV**CC**ADSVY**CC**PSDYPI**C**DVEEGL**C**IK

P_mar_S4_02 VS**C**ANRRY**C**PGDST**CCC**LP-----AGSWG**CC**GVPNAV**CC**ADGVH**CC**PAGH-V**C**M--EKY**C**MK

.*.: ***.*.****: . :***. *******.*:***:.: :* * *:*

Although the triple Cys aligns perfectly, and there is a good level of sequence similarity elsewhere, the lack of an additional single Cys in a longer loop before the following double Cys ensures that it falls in more naturally with the granulins of animals.

When the DNA sequences encoding the N- and C-half modules are included in the phylogenetic tree, the N-halves of both are grouped with fish small form half modules, and specifically module 2 of the progranulin C type (3 to 7 in the case of coelacanth). The C-halves, however, group with the C-halves of the final modules of fish long forms and of *X. tropicalis*. The sub-trees are shown below with the lamprey labels in italics.

**N-Half (DNA) tree (Fig. 3, 05b) C-half (DNA) tree (Fig. 4, 07b)**


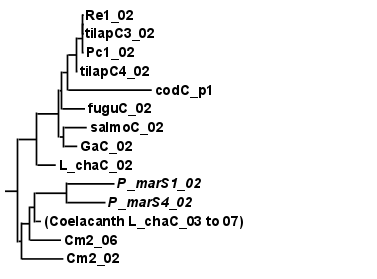

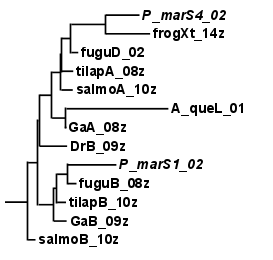


**References.**

Hrabal, R., Z. Chen, S. James, H. P. Bennett, and F. Ni. 1996. The hairpin stack fold, a novel protein architecture for a new family of protein growth factors. Nat Struct Biol 3: 747-752.

Tolkatchev, D., P. Xu, and F. Ni. 2001. A peptide derived from the C-terminal part of a plant cysteine protease folds into a stack of two beta-hairpins, a scaffold present in the emerging family of granulin-like growth factors. J Pept Res 57: 227-233.
