# Supplementary material for: The Evolution of the Secreted Regulatory Protein Progranulin
Source: PLoS One. 2015 Aug 6;10(8):e0133749. doi: 10.1371/journal.pone.0133749 (PMC4527844; doi:10.1371/journal.pone.0133749)
Supplement: S3 Text — (DOCX) [file pone.0133749.s015.docx]

***Supplementary text 3: Progranulin exon structures***

The underlying exon structure for *Grn* genes from representative species is shown in the table below. Features include a first exon sn with a signal peptide and a sequence encompassing the N-terminal 6 cysteines, the common c, n and cn exons as defined in the main text, examples of modified granulin motifs (n*, c*, c’, n’ etc) which differ from the canonical 12 cysteine granulin array, and large exons encoding multiple granulin modules. All members of the GrnA synteny group have the modified 10 cyteine motif as the first full granulin module (n*-c*).

Exon structures for some *Grn* genes (including the species presented in figure 5); the synteny group (A, B or C) as defiend in the text is shown where known.

Mammal sn-n*-c*-n-c-n-c-n-cn-cn-cn-c GrnA

Anolis (A_car) sn-n*-c*-n-c-n-c-n-cn-cn-cn-cn-cn-c GrnB

Frog (X_tro) sn-n*-c*-n-c-n-c-n-c-n-c-**n-c-n-c-n-c-n-c-n-c-n-c-n-c**-n-cn-n-c GrnA

Coelacanth (L_chaA) sn-n*-c*-n-c-n'-**cn-cn-cn-cn**-cn-cn-cn-c GrnA

Coelacanth (L_chaC) sn-cn-**cn-cn-cn-cn-cn-c**... (uncertain if this is the last exon) GrnC

Tilapia (O_nilA) sn-c-n*-c*-n-c-n-cn-cn-cn-cn-c GrnA

Tilapia (O_nilB) sn-c-n-c-n-c-n-c-n-cn-cn-cn-cn-cn-c GrnB

Tilapia (O_nilC1) sn-c-n-c GrnC

Tilapia (O_nilC2,3,4) sn-cn-c GrnC

Elephant Shark C_mil1 sn-c-c-n-c-n-**cn-cn-cn-cn**-cn-cn-c

Elephant Shark C_mil2 sn-cn-c**n-c'n-c'n-c'n-cn-c** GrnC

Lamprey (P_marL) sn-c-n-cn-**cn-cn-cn-cn-cn-cn-c**n-c

Lancelet (B_flo) sn-cn-cn-c-n-cn-c'-|nc|-n'cnc**ncncncncncnc{***ill-defined* ***repeats*}-nc**-n-c-n-c-n-c

Sea squirt (C_intL) s-n-c-**n-c-n-c-n-c-n-c-n-c-n**-c

Sea urchin (S_pur) s-nc**ncncncncncncncncncncncncncncncncncncncncncncncnc**-n-cn-n-c

Sea anemone (N_vec) snc-nc**ncncncnc**ncncnc”nc-ncncnc

Sponge (A_queL) snc-ncncncncncncncncncncncncnc (Highly repetitive)

Special notation (e.g. n' or c”) is defined for a species in the supplementary document of sequences used in this study.Otherwise, the notation is as in table 1 of the manuscript. Bold indicates stretches of repeats of nearly identical sequence.The repeated sequences in (not bolded) A_queL fall mostly into 2 closely-related groups.

*Signal peptide and first half-module encoded in same exon*

Typically, the exon encoding the first N-half module includes most, and most frequently all, of the signal peptide. This is highly conserved as shown by the grouping of the first N-half module from fish progranulins (both large and small form), the mammalian, anole, coelacanth and frog paragranulins, the lancelet *Branchiostoma* (B_flo), and even the sea anemone *Nematostella* in sub-tree Nsub03 of figure 3. The sea urchins (*Strongylocentrotus purpuratus* & *Paracentrotus lividus*) differ because their signal peptide is encoded in a separate upstream exon, nevertheless, as with the sn exons above their first N-half modules are grouped in Nsub03. Exceptions are provided by the lamprey, sea squirt and sponge which all have the signal peptide and first N-half module encoded in the same exon, but fall into the outgroup subtree Nsub01.

*Intron loss*

The exonic structure of the *Grn* genes suggest two types of intron loss as having occurred. 1) The loss of a single intron to produce a -cn- exon from c and n exons. 2. The loss of several neighbouring introns to produce extraordinarily large exons encoding several modules (as in sea urchin, sea anemone, sponge, and the middle repetitive section of the lancelet *B. floridae*).

Among the proposed mechanisms for intron loss, reverse transcriptase-mediated intron loss (RTMIL) (Niu et al. 2005; Fink, 1987) is the most attractive for explaining the origin of large exons. The insertion through homologous recombination would occur after the accurate splicing which is necessary to produce well-formed granulin modules and result in exons encoding multiple granulin modules. This mechanism does not explain the preponderance of cn exons as, if the RTMIL process had removed introns from between c and n exons, it should have been equally effective in removing them from between n and c exons. : Intron loss between half-module exons could have occurred via mechanisms reported for the introduction of deletions in DNA and shortening of introns, such as non-homologous end joining (NHEJ) following double strand breaks, and an error-prone homology-based repair (Pontier & Tijsterman, 2009; van Schendel and Tijsterman, 2013). There are a number of possible reasons why cn exons are common and nc exons rare, none of which can, at this stage be definitively excluded.

1: All the cn exons may have arisen from only one or two original events, and have populated the various progranulin genes via duplications.

2: cn exons may exert a positive selective advantage compared to nc exons. One potenitla advantage of the cn type exons may related to their ability to increase the number of unique granulin module sequences that been encoded from a single gene. The c, n and cn exons are in phase such that skipping a cn exon generates a new hybrid granulin module (Bhandari and Bateman, 1992). In the example below, skipping the C2N3 exon in line (a) results in a new hybrid module, N2C3 (underlined), increasing, in principle, the number of possible granulin modules that be encoded in a single gene (Bhandari and Bateman, 1992). Alternately transcribed transcripts that contain hybrid granulin modules have been observed (Plowman et al, 1992). Skipping a hypothetical nc exon, for example, N2C2 in line (b) results only in the loss of granulin modules without generating novel hybrid granulin module sequences.


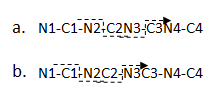


3: But addition or removal of codons at the junction of N-half and C-half modules would compromise the module structure and be unlikely to survive selection, whereas the joining sequences between modules, being variable in composition and length, would be more likely to accommodate such changes. This would account for cn exons being common but nc exons rare.

*Other unusual exon-intron features.*

There is an unusual exon structure immediately before the tandem repeat sequence. It is represented in the table above as -c'-|nc|-n'(cnc repeat). The |nc| exon encodes a granulin module which lacks the beginning containing the first Cys and the end containing the last Cys. But these ends are supplied by the flanking exons. Thus:

560 base intron 760 base intron

...VKDVP**C**PDGK / SA**C**EEDYT**CC**KNSTGYG**CC**PFSRAV**CC**NDTVH**CC**PQHYT**C**NSGAP / (G)T**C**IHNFL...repeat

sequence

The introns contain no recognizable trace of granulin sequence. However this peculiar distribution of a module sequence between 3 exons arose, it's symmetry is intriguing, and it implies the influence of selection pressure to preserve a normal granulin module motif.

**References:**

Bhandari, V., and A. Bateman, 1992. Structure and chromosomal location of the human granulin gene. *Biochem Biophys Res Commun* **188**: 57-63.

G.R. Fink, 1987. Pseudogenes in yeast? *Cell* 49:5-6

Deng-Ke Niu, Wen-Ru Hou, and Shu-Wei Li , 2005. mRNA-Mediated Intron Losses: Evidence from Extraordinarily Large Exons . *Mol. Biol. Evol.* **22**:1475–1481

Daphne B. Pontier and Marcel Tijsterman, 2009. A Robust Network of Double-Strand Break Repair Pathways Governs Genome Integrity during C.elegans Development

*Current Biology* **19:**1384–1388

Plowman, G. D., J. M. Green, M. G. Neubauer, S. D. Buckley, V. L. McDonald, G. J. Todaro, and M. Shoyab. 1992. The epithelin precursor encodes two proteins with opposing activities on epithelial cell growth. *J Biol Chem* **267:** 13073-13078

Robin van Schendel and Marcel Tijsterman, 2013. Microhomology-Mediated Intron Loss during Metazoan Evolution. *Genome Biol Evol* **5**:1212-1219
